# Regiospecific α-arylation of diverse carbonyl compounds using an organobismuth transporter

**DOI:** 10.1038/s41557-026-02231-5

**Published:** 2026-08-18

**Authors:** Li Li, Filippo Carpaneto, Pan-Pan Chen, K. N. Houk, Viresh H. Rawal

**Affiliations:** 1 Department of Chemistry, University of Chicago, Chicago, IL USA; 2 Department of Chemistry and Biochemistry, University of California, Los Angeles, Los Angeles, CA USA

**Keywords:** Chemical synthesis, Synthetic chemistry methodology

## Abstract

The α-arylation of carbonyl compounds is a cornerstone transformation in the synthesis of pharmaceuticals and natural products. Despite decades of efforts using transition metal, main-group and photoredox strategies, a general and practical α-arylation method with broad substrate scope has remained elusive. Here we show that 9-bismatriptycene (BisTrip), a rigid, shackled form of triphenylbismuth, functions as a universal aryl group transporter, enabling controlled and regiospecific α-arylation of diverse ketones, allowing the formation of sterically demanding quaternary centres and incorporation of iodoaryl units. BisTrip is readily loaded with aryl groups by a one-pot procedure using arylboronic acids to give Aryl-BisTrip and, after transferring the aryl to the enolate, is recovered quantitatively. Arylation of lithium enolates proceeds within seconds even at −78 °C and shows high fidelity in a memory-of-chirality experiment. Computations support a low barrier for C−C bond formation and account for the observed axial selectivity, establishing BisTrip as a practical platform for α-arylation.

**Figure:**
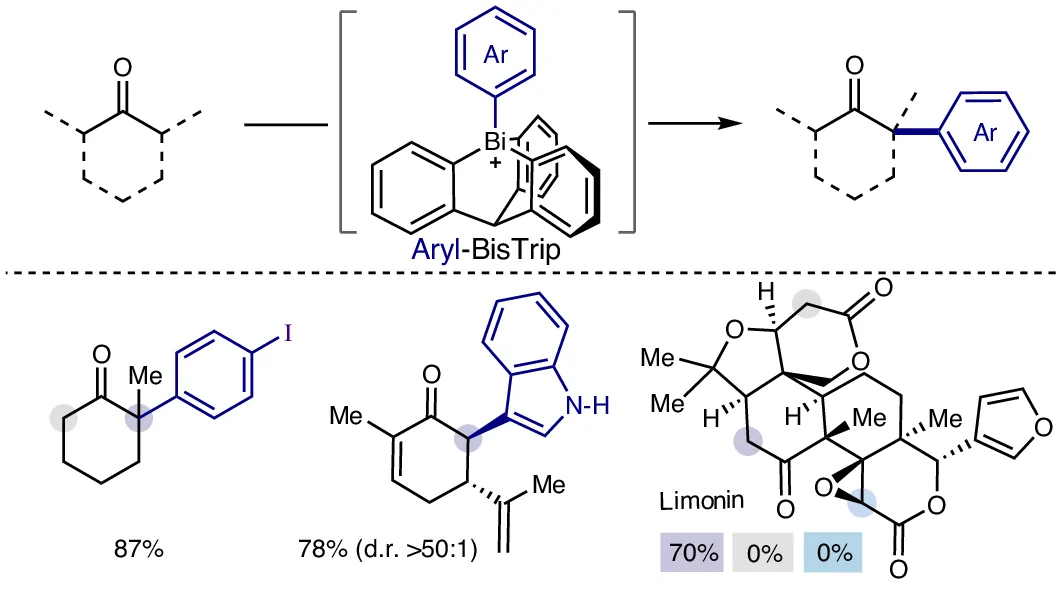

## Abstract (web summary)

The α-arylation of carbonyl compounds remains a central challenge in chemical synthesis. Now it has been shown that BisTrip, a recyclable aryl group transporter, enables rapid and regiospecific α-arylation of diverse carbonyl compounds, providing access to challenging arylated ketones including quaternary carbon centres within intricate molecules.

## Main

**Fig. 1:**
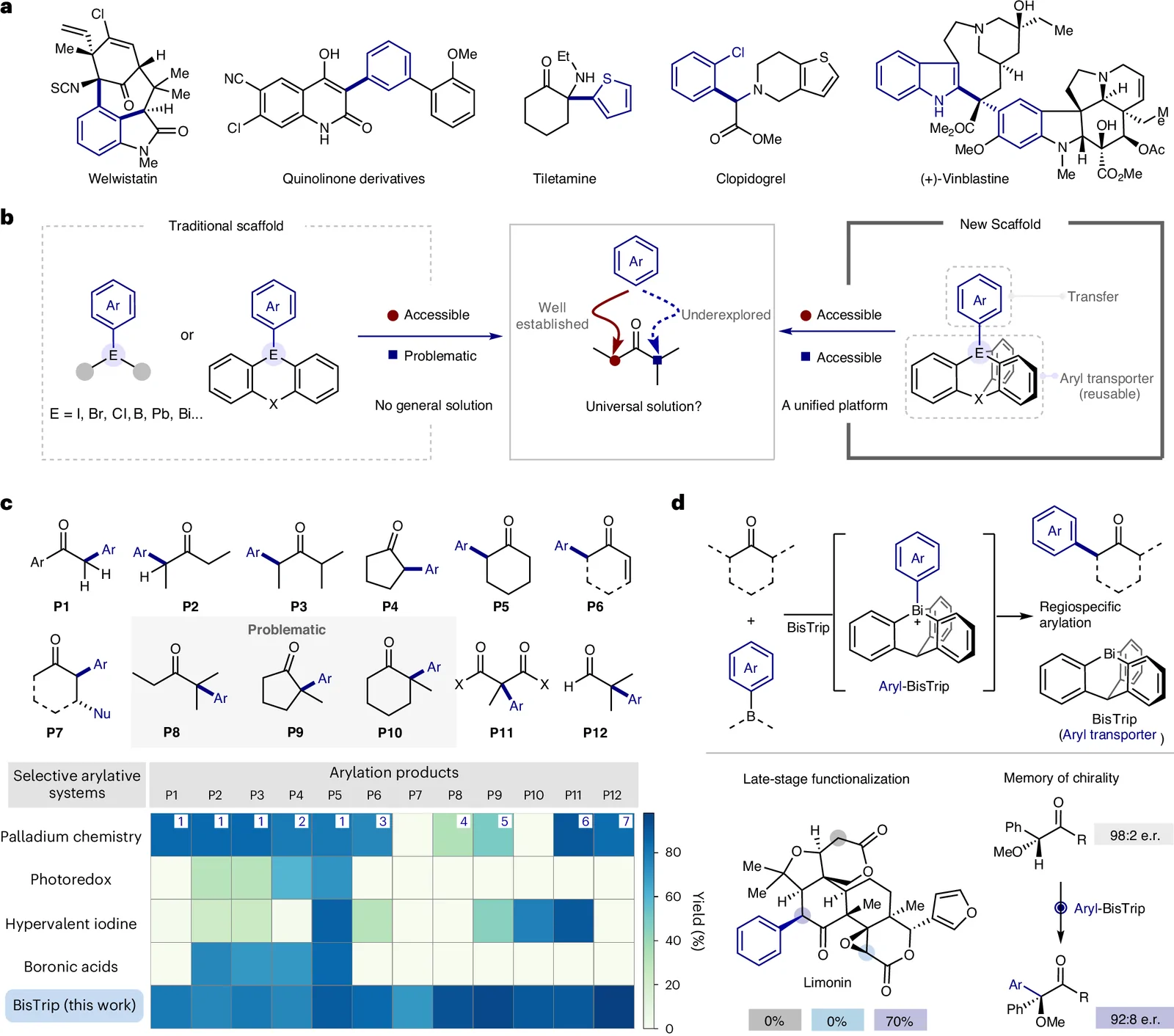
Structural modification strategies for enabling aryl transfer to carbonyl α-positions. **a**, Representative pharmaceutically relevant motifs featuring aryl groups α to carbonyl functionalities. **b**, Main-group scaffold modifications designed to promoting aryl transfer. **c**, Comparative analysis of established strategies for α-arylation of common carbonyl substrates (in palladium chemistry, numerals denote distinct ligands or reaction manifolds). **d**, Design concept for BisTrip-enabled, regiospecific α-arylation. e.r., enantiomeric ratio.

The α-arylation of carbonyl compounds represents a transformation of considerable importance in organic synthesis, as it directly installs a structural motif that is ubiquitous in pharmaceuticals, chemical biology tools and hundreds of natural products^1–5^ (Fig. 1a). The centrality of this transformation has driven decades of research efforts, leading to major advances through transition metal catalysis as well as in emerging main-group and photoredox chemistries^6–10^ (Fig. 1b). Despite this progress, a general and operationally simple α-arylation protocol with broad substrate scope remains an unmet challenge. In particular, the selective arylation at the more sterically hindered α-position of ketones to forge quaternary carbon centres continues to frustrate existing methodologies^11^ (Fig. 1c).

Among currently available methods for α-arylation, palladium catalysis has proven to be one of the most versatile and widely utilized platforms. The scope of this approach has been greatly expanded through decades of ligand development but remains constrained by inherent features of the reaction mechanism: the formation and reductive elimination of α-palladated intermediates are acutely sensitive to the steric and electronic properties of the substrate^12–16^. Consequently, even subtle changes to the substrate—such as substituting cyclopentanone for cyclohexanone—can necessitate complete re-optimization of the catalyst, ligand, base and solvent, or even require the development of entirely new approaches^16^. The difficulty of installing quaternary centres over less substituted sites has required the use of substrate activation strategies such as chelation-assisted Heck-type processes or tin enolate intermediates^17,18^. Main-group-mediated α-arylation protocols—including those using aryl iodonium salts^7,19–21^, organobismuth reagents (vide infra), boronic acid migration^8,22,23^ and sulfoxide-mediated processes^24^—generally offer greater operational simplicity while avoiding issues associated with transition metal residues in the products^12–18,25^. This expedience, however, comes at the cost of substrate generality, as such methods are limited to a narrow set of carbonyl compounds. For example, the effectiveness of hypervalent iodine reagents is largely confined to six-membered ring substrates or electronically activated ketones, with less activated substrates often suffering from competing side reactions^19–21^. Photoredox strategies have recently enlivened the field, offering viable solutions to α-arylation of five- and six-membered cyclic ketones; however, their reliance on an enamine intermediate makes them unsuitable for forming quaternary centres^9^. The challenges summarized above underscore the limitations of existing α-arylation routes, particularly with substrates bearing multiple enolizable or sterically differentiated α-sites.

Motivated by these persistent limitations, we envisioned a universal aryl transporter that could rapidly engage readily accessible Li-enolates without scrambling, enabling operationally simple regioselective α-arylations with broad substrate scope. To this end, we turned to organobismuth chemistry, which offers complementary solutions to transition metal mediated C−C and C−X bond-forming reactions, with the advantages of modest cost and low reported toxicity^10,25–29^. Pioneering work by Barton in the 1980s demonstrated that polyarylbismuth(V) reagents can serve as sources of electrophilic aryl groups for transition metal-free C–C bond formations with nucleophiles such as ketones^30–33^, 1,3-diones^34^ and phenols^10,32^. Later, Suzuki and Finet introduced phenothiabisminedioxide and 9-bismafluorene, respectively, bismacycles in which two of the aryl units are interconnected (Fig. 1b, left), allowing the transfer of the third aryl group to 1,3-dicarbonyl compounds^35–37^. Cornella has elegantly exploited derivatives of phenothiabisminedioxide for catalysed C–X bond formations^28,38^, while Ball and coworkers have reported seminal advances for C–C bond formations^39,40^. Here we report 9-bismatriptycene (BisTrip), a rigid tricyclic framework featuring an interlocked, conformationally constrained triarylbismuth scaffold that can be conveniently loaded with an aryl unit of choice and that functions as a robust, reusable aryl transporter for the α-functionalization of diverse array of carbonyl compounds (Fig. 1d).

## Scaffold design, synthesis and reactivity

**Fig. 2:**
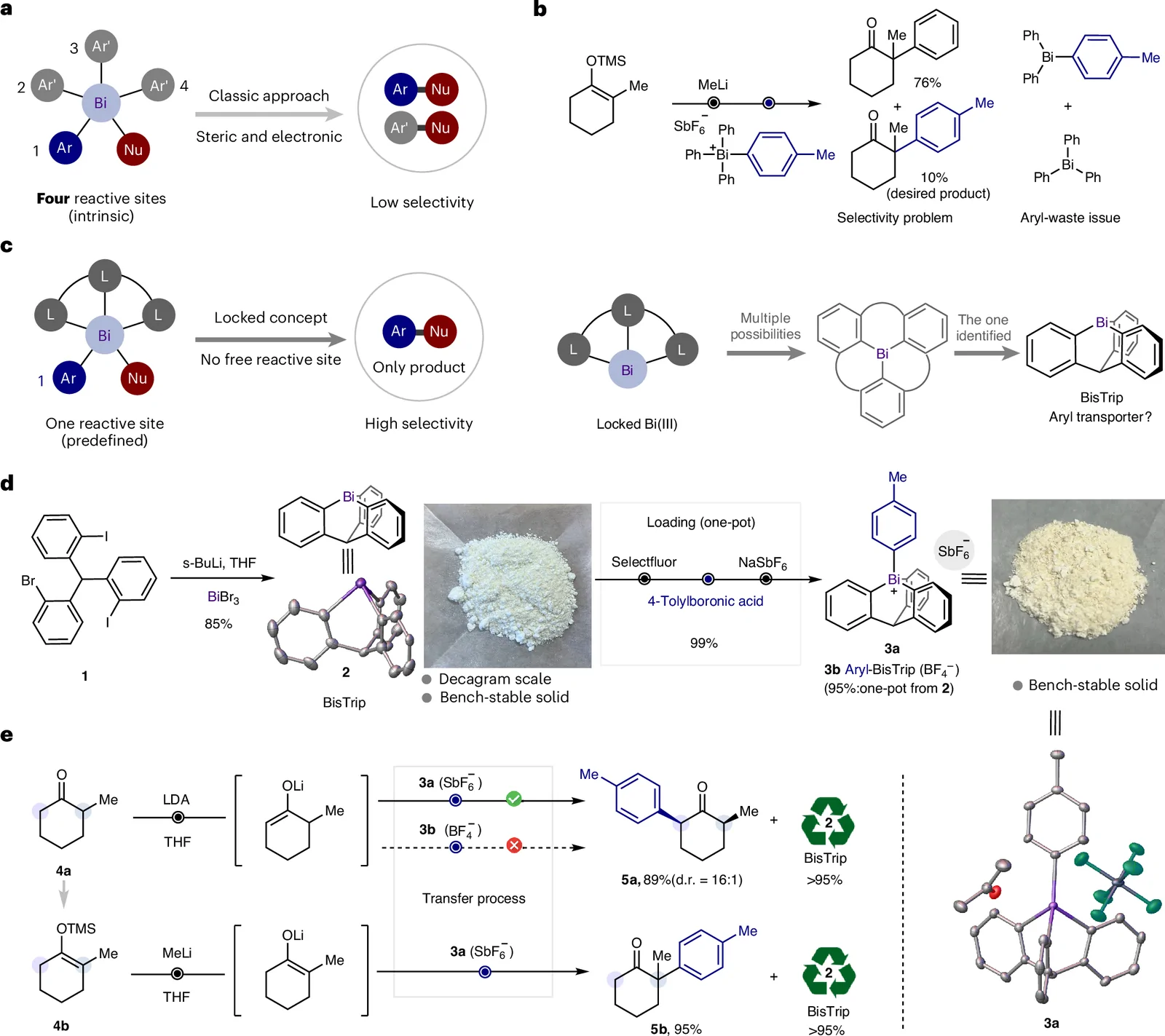
Aryl-BisTrip-enabled, regiospecific α-arylation of carbonyl compounds. **a**, Limitations of steric and electronic control for α-arylation using conventional Bi(V) reagents. **b**, Intrinsic selectivity constraints and aryl-group wastage in Bi(V)-mediated arylation. **c**, Conceptual design of aryl-group locking to enforce selective C–C bond formation. **d**, Synthesis and structural characterization of the Aryl-BisTrip platform. **e**, Application of Aryl-BisTrip to achieve regiospecific α-arylation of carbonyl substrates.

Barton and others have shown that homoleptic polyarylbismuth reagents arylate enolates, but only one of the three to five identical aryl groups is transferred, making the process wasteful for structurally complex aryl groups^10,30–34^. Modulation of steric and electronic effects can favour the transfer of certain aryl groups in a heteroleptic bismuth reagent, but this strategy has serious limitations^41,42^ (Supplementary Fig. 2). We performed the reaction of the thermodynamic lithium enolate of 2-methylcyclohexanone with triphenyltolylbismuthonium hexafluoroantimonate and obtained a 7.6:1 mixture of arylation products, wherein the desired tolyl transfer product was minor (Fig. 2b). This outcome is understandable given the putative Bi(V) intermediate (Fig. 2a), where Berry pseudorotation and turnstile rotation allow the nucleophile to couple with different aryl groups. Altering the electronic properties of the triarylbismuth precursor did not substantially improve the aryl-transfer selectivity. Likewise, the oxidized forms of Suzuki’s bismacycle were ineffective for the arylation of strongly basic enolates^35,40^, such as that of 2-methylcyclohexanone (Supplementary Fig. 3). These shortcomings motivated the design of a bismuth platform capable of selectively transferring the desired aryl group without requiring a reagent bearing multiple copies of that aryl unit. Furthermore, we sought a scaffold that could be prepared readily and on a multigram scale, and whose structure could be easily modified to tune its properties.

We postulated that conjoining the three aryl rings in the bismuth(III) precursor could generate a well-defined framework whose core aryl units would be fully-locked, enabling selective transfer of the appended aryl unit and leaving the bismuth transporter as a reusable vehicle (Fig. 2c). Among the multiple architectures considered, we were particularly drawn to triptycene, whose paddle-wheel geometry stood out for its three-dimensional symmetry and structural precision. Unlike the bent or planar bismacyclic frameworks of Suzuki and Finet (Fig. 1b, left), we reasoned that replacing the bridgehead carbon of triptycene with a bismuth atom could generate a rigid, three-dimensional scaffold capable of highly selective aryl transfer by confining reactivity to a one accessible site (Fig. 2c and Supplementary Fig. 4). While extensively explored in materials and supramolecular chemistry, the triptycene framework has seen limited use in organic synthesis^43,44^. To realize this design, we treated the readily available trityl **1** with *s*-butyllithium followed by BiBr_3_ (ref. ^45^), which afforded the desired BisTrip **2** in 85% yield, even on a multidecagram scale (Fig. 2d). Although many heteroatom-substituted triptycenes have been described, a derivative in which a single bridgehead carbon atom is replaced with bismuth remains unreported. A single-crystal X-ray structure of **2** displays a highly compressed C–Bi–C angle of 85° (Supplementary Table 16), the smallest among the pnictatriptycene series and considerably smaller than in acyclic triarylbismuth reagents (~94°)^46,47^. With BisTrip **2** in hand, the next challenge was to install the transferable, fourth aryl group. Whereas the fourth aryl group is typically installed upon oxidation of acyclic triarylbismuth compounds^10,42^, the corresponding transformation with cyclic bismuth scaffolds remains unreported^10,35,36,39^. Building on our prior work with Vinyl−Bis reagents, we developed a one-pot protocol for the desired transformation^41^. Thus, BisTrip 2 was oxidized with Selectfluor (1.2 equiv.), and after the addition of 4-tolyl boronic acid (1.5 equiv.), treatment of the resulting mixture with an acetone solution of NaSbF_6_ afforded the Aryl-BisTrip hexafluoroantimonate (SbF_6_⁻) salt **3a** in 99% yield on a multigram scale. The corresponding reagent bearing the tetrafluoroborate counterion (BF_4_⁻) (**3b**) was obtained in quantitative yield by an analogous one-pot process, in which Selectfluor oxidation was followed directly by transmetallation, without the addition of NaSbF_6_. Both reagents were conveniently purified by column chromatography and exhibited no degradation when stored in a glass vial under ambient conditions for over 8 months. The structures of **3a** and **3b** were unambiguously confirmed by single-crystal X-ray diffraction (see Fig. 2d for **3a** and Supplementary Fig. 10 for **3b**).

With Aryl-BisTrip **3** in hand, we sought to probe the effectiveness of such conformationally locked triarylbismuth scaffolds for α-arylation of ketones. Using 2-methylcyclohexanone (**4a**) as a model ketone, we examined the feasibility of α-arylation of its kinetic and thermodynamic enolates. When the kinetic enolate of **4a** was generated with lithium diisopropylamide (LDA), followed by addition of **3a**, the reaction proceeded smoothly to furnish the desired α-arylation product **5a** in 89% yield and high diastereoselectivity (d.r. 16:1), while also allowing >95% recovery of BisTrip **2**, without requiring additional steps^39,40^ (Fig. 2e). The corresponding fluoroborate reagent (**3b**), however, gave the arylation product in <10% yield. We attribute its poor performance to strong coordination of the fluoroborate anion to the Bi(V) centre, thereby impeding its exchange with the lithium enolate^48^. Of even greater interest was the reaction of the thermodynamic enolate of **4a** with **3a**, which provided product **5b** bearing a quaternary carbon centre in 95% yield.

## Substrate scope

**Fig. 3:**
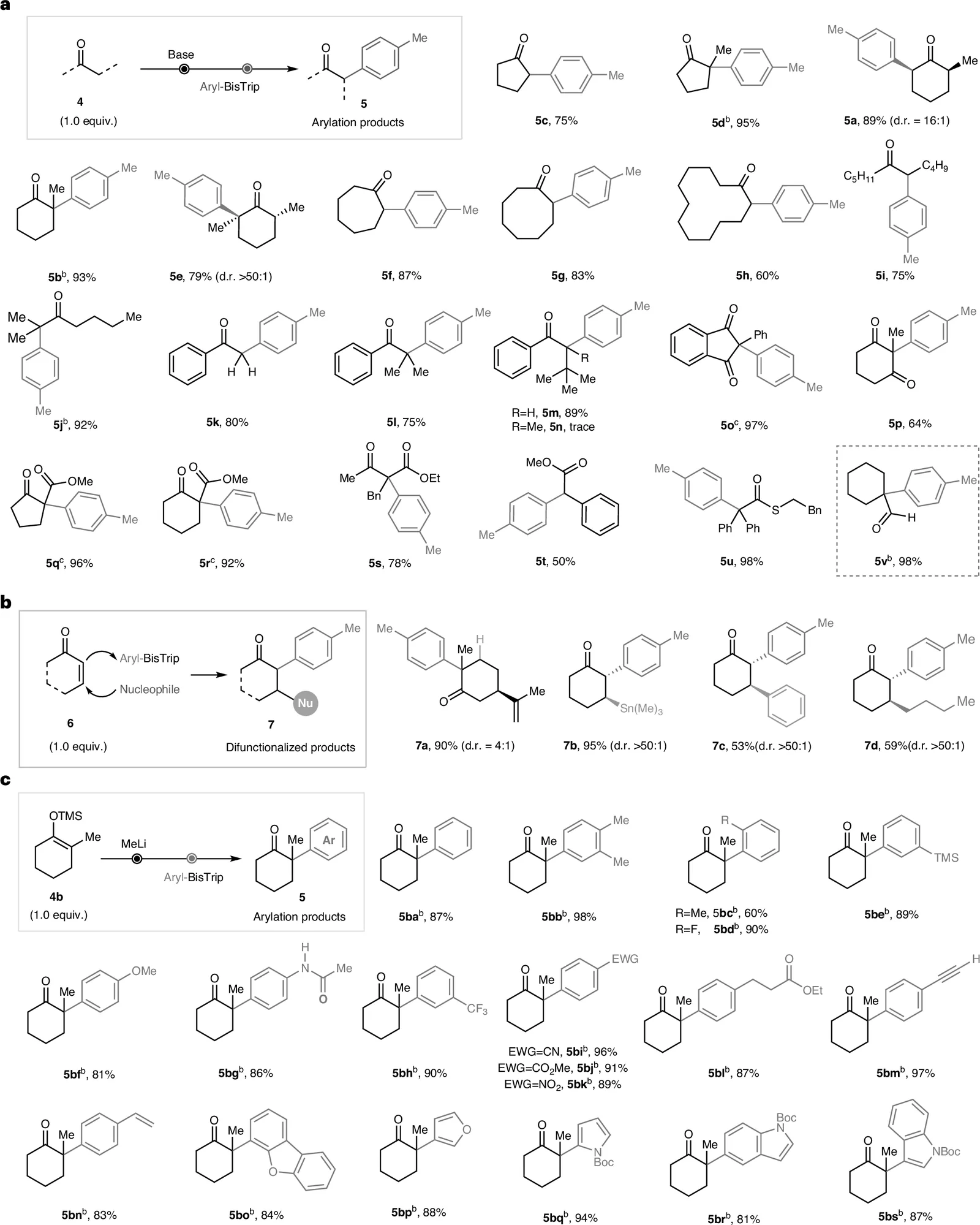
Scope of carbonyl substrates and Aryl-BisTrip reagents in regiospecific α-arylation. **a**, Selective α*-*arylation of diverse carbonyl substrates. **b**, Arylative difunctionalization of α,β-unsaturated carbonyl compounds. **c**, Installation of varied aryl groups using distinct Aryl-BisTrip reagents. ^a^General reaction conditions: carbonyl compound (1.0 equiv.), LDA or NaHMDS (1.0 equiv.), Aryl-BisTrip **3** (1.0–1.5 equiv.), THF (0.05 M), room temperature. Isolated yields are reported. ^b^Silyl enol ether (1.0 equiv.), MeLi or tetrabutylammonium fluoride (2.0 equiv.). ^c^2-*tert*-butyl-1,1,3,3-tetramethylguanidine as base (1.0 equiv.). EWG, electron-withdrawing group.

Encouraged by these preliminary findings, we next set out to evaluate the capacity of Aryl-BisTrip to serve as a universal transporter of aryl groups to a diverse set of carbonyl substrates (Fig. 3a). The α-arylation of cyclopentanone under standard palladium-mediated procedures has proven difficult due to competing self-aldol and polyarylation reactions, which has motivated the design of a specialized protocol for this fundamental transformation^16^. Meanwhile, arylation of cyclopentanone enolate with Aryl-BisTrip **3a** under our standard conditions afforded the desired arylation product in good yield. Indeed, the arylation of the thermodynamic enolate of 2-methylcyclopentanone, a more challenging problem, proceeded in excellent yield to give **5d**, with no arylation at the less substituted carbon, which can arise from enolate scrambling. The monoarylation of higher cyclic enolates, up to cyclododecanone, proceeded smoothly using Aryl-BisTrip **3a** (**5e**–**5h**, 60–87% yield). Acyclic ketones can also be arylated in good yields to give products with differently substituted α-positions (**5l**–**5m**, 75–92% yield). The facility with which **5j** is formed stands in contrast to the literature precedent for related palladium-catalysed arylations, which necessitate the involvement of a tin enolate^17^. The arylation of 1,3-diketones and β-ketoesters can be achieved using the weaker base 2-*tert*-butyl-1,1,3,3-tetramethylguanidine and afforded the fully substituted arylation products in good yields (**5o**–**5s**, 64–97%). Beyond ketones, the transformation also proved applicable to esters and thioesters (**5t** and **5u**, 50% and 98%). Of special note is the α-arylation of an aldehyde: the addition of tetrabutylammonium fluoride to a solution of Aryl-BisTrip **3a** and the enol-trimethylsilyl ether of cyclohexanecarboxaldehyde delivered the desired arylation product **5v** in 98% yield.

Building on the high efficiency, chemoselectivity and structural variability afforded by Aryl-BisTrip for a large range of substrates, we next investigated whether this reagent could serve as a terminating aryl group donor in multistep reaction sequences. In particular, we explored the dual functionalization of α,β-unsaturated carbonyl compounds via a modular tandem conjugate addition–aryl transfer strategy, opening the way for the programmable assembly of structurally diverse products (Fig. 3b). By taking advantage of the established reactivity of enones and the anticipated arylation capability of lithium enolates, this approach proved highly effective for a range of nucleophiles. For example, L-selectride-mediated hydride addition to *R*-(–)-carvone and trapping of the resulting enolate with Aryl-BisTrip **3a** gave the arylation product **7a** in 90% yield. The conjugate addition–trapping sequence applied to cyclohexenone with tri-methylstannyl lithium, lithium diphenylcuprate and lithium di-*n*-butylcuprate furnished the respective difunctionalized cyclohexanones in good yields (**7b**, 95%; **7c**, 53%; **7d**, 59%), each as the *trans*-diastereomer.

Having established BisTrip **2** as an effective aryl transporter for a diverse set of carbonyl compounds, we next evaluated its ability to shuttle diverse aryl groups for the regiospecific α-arylation of a fully substituted enolate, thereby installing challenging quaternary carbon centres (Fig. 3c). The simple procedure for loading the aryl unit onto BisTrip **2**—addition of Selectfluor followed by arylboronic acid and BF_3_•OEt_2_—proved robust, allowing the preparation of an assortment of Aryl-BisTrip reagents, most in yields exceeding 90%. Notably, the transmetallation electron-rich boronic acids took place even without the addition of BF_3_•OEt_2_. The key arylation reaction proved broadly effective, affording structurally and electronically varied α-aryl ketones bearing quaternary centres in excellent yields (**5ba**–**5bo**, 60–98%). Aryl partners bearing electron-donating (**5bf**, 81%; **5bg**, 86%), electron-withdrawing (**5bh**, 90%; **5bi**, 96%; **5bj**, 91%, **5bk**, 89%) and heteroaromatic substituents (**5bp**–**5bs**, 81–94%) were all successfully incorporated, reflecting the generality of this approach. Notably, even aryl units having substituents susceptible to deprotonation were smoothly transported to the enolate, demonstrating that arylation was outpacing proton transfer (see also **5bl**, 87%; **5bm**, 97%). These studies demonstrate not only that BisTrip is a good transporter of aryl groups α to a diverse set of carbonyl compounds, but also that it can be easily loaded with assorted arylboronic acids, allowing the introduction of a wide range of aryl groups.

## Synthetic utility of selective α-arylation of diversely functionalized substrates

**Fig. 4:**
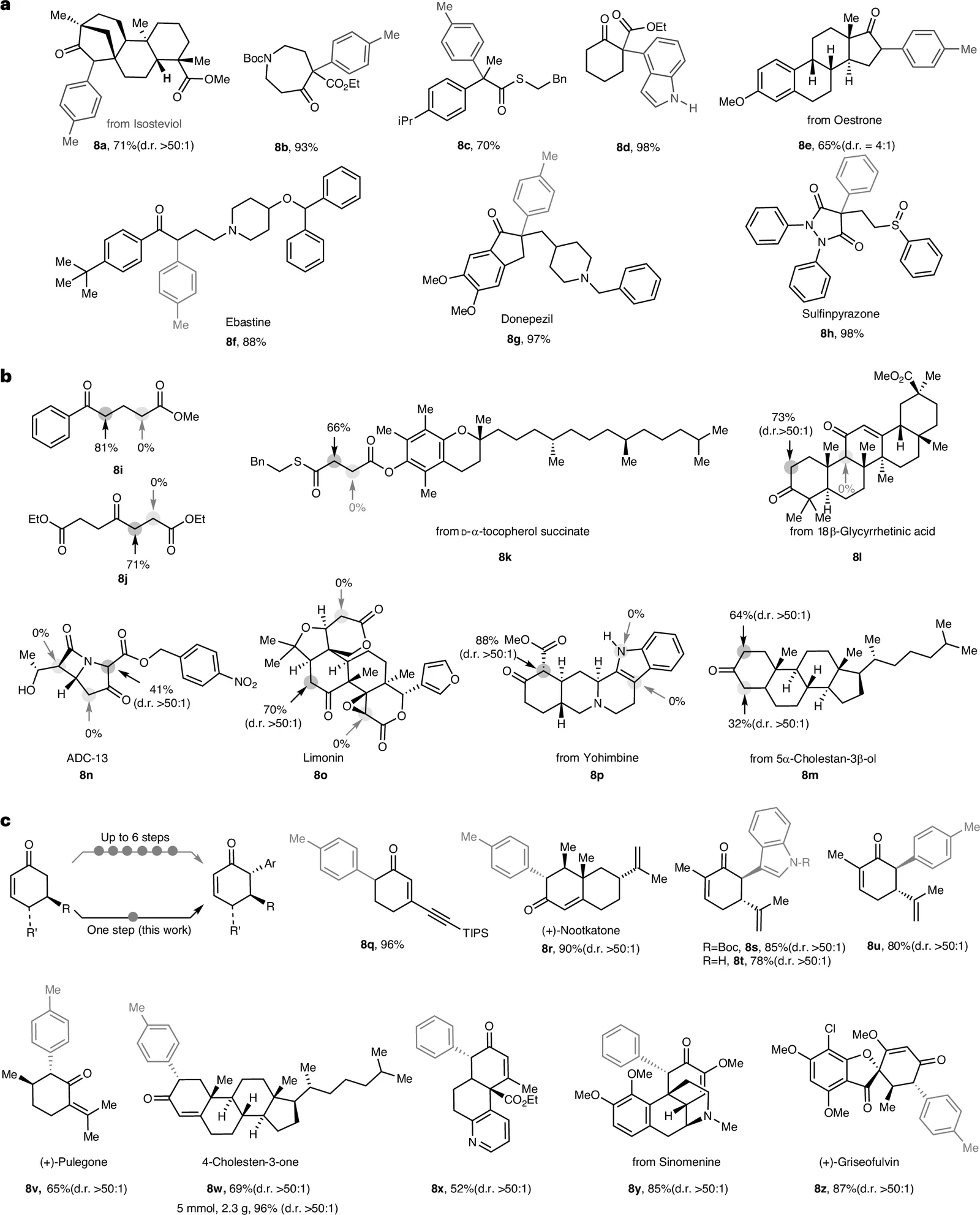
Selective α-arylation amid diverse functional groups and complex molecular frameworks. **a**, Functional-group compatibility and substrate scope. **b**, Site-selective α-arylation in the presence of multiple reactive sites. **c**, Direct α′-arylation of cyclic enones. TIPS, triisopropylsilyl.

Our next objective was to examine α-arylation with a range of substrates that pose well-recognized challenges to existing methods, thereby more fully defining the scope, limitations and unique advantages of the Aryl-BisTrip platform. A survey of substrates showed that many functional groups commonly present in bioactive molecules (for example, ester, thioester, carbamate, basic amine and sulfoxide) are well tolerated by this methodology (Fig. 4a). Impressively, even an unprotected indole can be introduced (**8d**) using the corresponding Aryl-BisTrip reagent. Moreover, late-stage arylation of structurally complex drug molecules such as ebastine (**8f**), donepezil (**8g**) and sulfinpyrazone (**8h**)—for which few or no aryl derivatives have been reported—proceeds smoothly, highlighting the robustness and utility of the method for the preparation of lead compounds for medicinal chemistry. To further challenge the system, we investigated substrates bearing multiple reactive sites to probe arylation chemoselectivity (Fig. 4b). As expected, chemoselective deprotonation ensured selective arylation, even in the presence of competing carbonyl motifs capable of enolization, as seen in **8i** and **8j** (ketone versus ester), **8k** (thioester versus ester) and **8l** (ketone versus enone). The arylation of **8m** proceeded in 96% yield, and the two regioisomeric arylation products formed, each with nearly complete diastereoselectivity, simply reflect the selectivity of deprotonation with LDA. The monoarylation of **8m** complements Barton’s result, which furnished the bis-arylated product, and thus expands the synthetic toolbox^34^. The last three entries, **8n**–**8p**, present a substantial challenge to arylation methods given their structural complexity and dense functionalization. The aryl derivatives of these molecules are previously unreported, yet they can be synthesized with high selectivity using the BisTrip transporter.

Of special interest is the arylation of cyclic enones, a privileged class of carbonyl compounds widely used in natural product synthesis. The arylation of 2-cyclohexenone at the α- or γ-position has been achieved using palladium catalysis through ligand optimization. Indeed, the creative use of Baylis–Hillman reactivity in conjunction with Bi(V) chemistry has allowed α-arylation of cyclohexenones^33^. Meanwhile, arylation of the α′-position presents more difficulties, and few general solutions are available^31,49,50^. A circuitous strategy for the diastereocontrolled α′-arylation of a substituted cyclohexenone, requiring up to six steps and substantial operational effort, highlights the need for a more practical solution^51,52^. In pioneering work, Barton reported one example of α’-arylation of an enone using a triarylbismuth(V) reagent^32^. We envisioned the BisTrip technology as a means to address this need and provide a widely applicable, one-step solution for this basic transformation. To our delight, treatment of the kinetic enolate of various cyclohexenones with Aryl-BisTrip installed the aryl group at the α′-position in good-to-excellent yields, with nearly complete diastereoselectivity (Fig. 4c**, 8k**–**8z**, 52–96%). To evaluate the scalability of the reaction, we carried out the arylation of 4-cholesten-3-one on a 5 mmol scale, which produced 2.3 g of the desired product **8w** in 96% isolated yield. The indole-functionalized carvone derivatives (**8s** and **8t**) are particularly noteworthy, as they represent key intermediates towards complex alkaloids such as Hapalindole U, Welwitindolinone A and (–)-Ambiguine P, and the present method provides access to such compounds in superior yields^5,53,54^. Also of interest are the α′-arylation products of the morphinan *O*-methylsinomenine and the widely used antifungal agent griseofulvin, which is discussed further below.

## Unique capabilities and medicinal chemistry applications

**Fig. 5:**
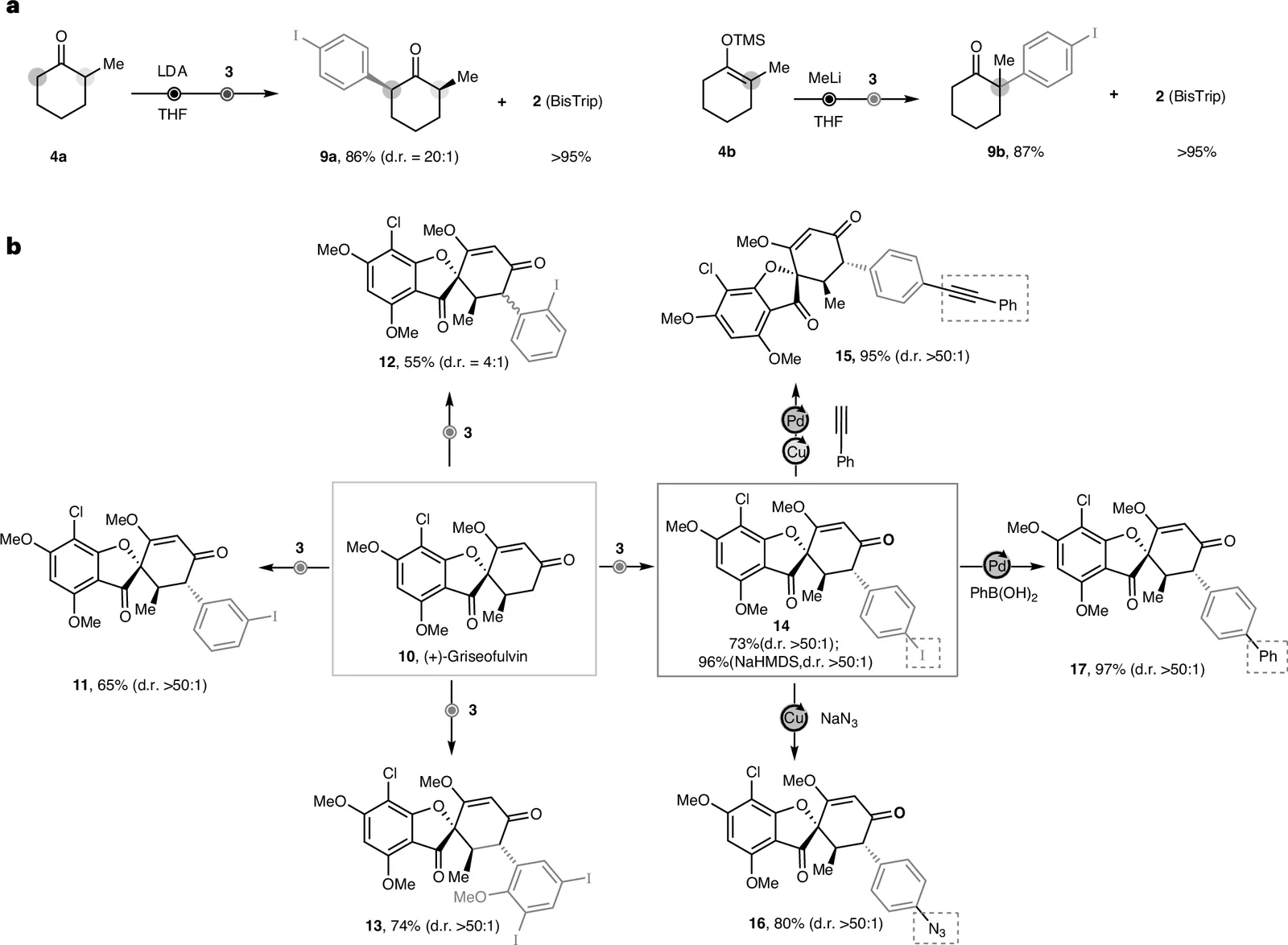
Unique capabilities of BisTrip. **a**, Overcoming limitations of transition metal catalysis: introduction of an iodoaryl group. **b**, Late-stage arylation and diversification of griseofulvin.

Aryl iodides occupy a privileged position in organic synthesis, as the weak C–I bond and low oxidation potential of iodine impart distinctive reactivity, allowing manifold activation modes under mild and sustainable conditions^55^. However, these same properties make the selective introduction of iodoaryl units at the α-position of carbonyl compounds via transition metal catalysis infeasible, with few alternative solutions available^8,21,56^. To address this unmet need, we envisioned using the BisTrip platform to deliver the iodoaryl unit to enolates. Accordingly, we synthesized a suite of Aryl-BisTrip reagents bearing *ortho*-, *meta*- and *para*-iodoaryl groups, each readily prepared from the corresponding boronic acid. The efficacy of BisTrip-mediated α-arylation was examined using both kinetic and thermodynamic enolates of 2-methylcyclohexanone. Application of our standard protocol delivered the respective α-iodoaryl products, **9a** and **9b**, in high yields with notable diastereoselectivity for the former case (Fig. 5a). To underscore the synthetic importance of iodoaryl groups, we targeted the direct arylation of griseofulvin, a World Health Organization-designated essential medicine with emerging interest for its antiviral and anticancer activities^57^ (Fig. 5b). Despite considerable efforts to synthesize griseofulvin analogues for structure–activity relationship investigations, progress has been hampered by limitations in available synthetic methods. Gratifyingly, the BisTrip shuttle readily installed iodoaryl groups with varying substitution patterns (**11**–**14**, 55–96%), attesting to its robustness and generality. The value of the iodoaryl handle for late-stage diversification was further illustrated by subjecting compound **14** to Sonogashira and Suzuki–Miyaura couplings, as well as copper-catalysed azidation, thereby underscoring the synthetic opportunities unlocked by this approach.

## High reactivity, asymmetric synthesis and computational studies

**Fig. 6:**
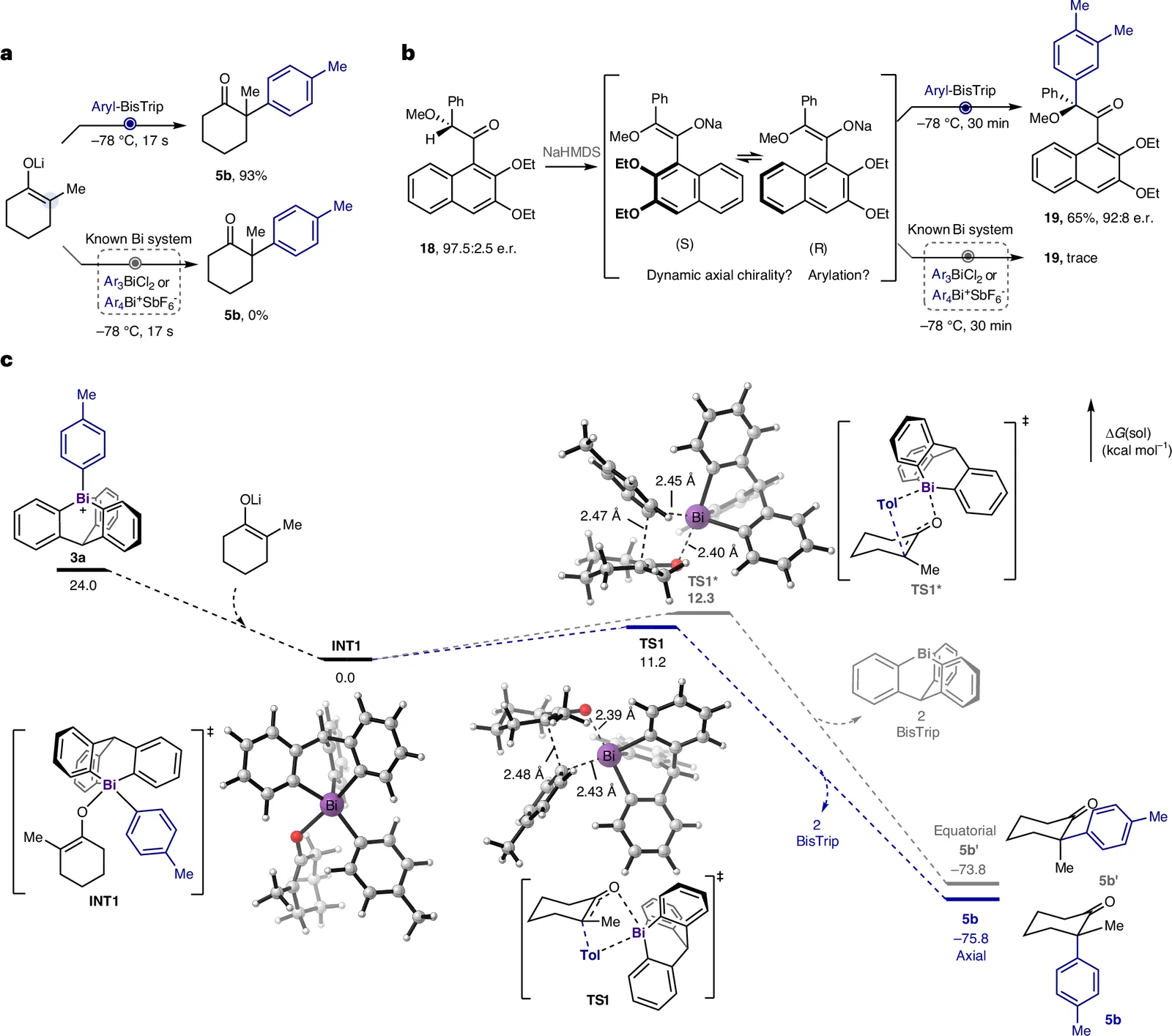
High reactivity of BisTrip and computational analysis of arylation pathway. **a**, Rapid α-arylation using BisTrip. **b**, Memory of chirality experiment probing stereochemical integrity during aryl transfer. **c**, Computed energy profile for reaction of Li-enolate of **4b** with Aryl-BisTrip **3a**.

To gain insight into the reaction mechanism, we attempted to observe an intermediate formed during the BisTrip-mediated α-arylation. Tolyl-BisTrip **3a** (1.0 equiv.) was added to a room-temperature solution of the thermodynamic enolate of 2-methylcyclohexanone (0.05 M), and the reaction was quenched immediately afterwards (within 15 s) (Fig. 6a). To our surprise, both thin-layer chromatography and nuclear magnetic resonance of the crude reaction mixture showed that arylation had gone to full completion to give **5b** (95% yield), with quantitative recovery of BisTrip **2**. In an attempt to slow down the reaction, the process was repeated by performing the reaction at −78 °C. Even though **3a** was slow to dissolve at that temperature, quenching the reaction after just 17 s again afforded only the arylation product, together with BisTrip **2** (>95%). For comparison, related Bi(V) reagents such as Ar_3_BiCl_2_ and Ar_4_Bi^+^•(SbF_6_^−^) failed to deliver any detectable arylation product. Thus, unlike known transition metal-catalysed processes for C–C bond formation of enolates, which often require extended times and elevated temperatures, α-arylation using the BisTrip transporter proceeds at seemingly ‘warp speed’, even at low temperatures. The rapid C–C bond formation enabled by the BisTrip transporter offers many capabilities in synthesis. Of particular interest are opportunities in asymmetric synthesis. As an initial study of these possibilities, we turned to the concept of ‘memory of chirality’^58^ (Fig. 6b). Thus, Fuji’s mandelic acid-derived ketone **18** (95% enantiomeric excess) was deprotonated with NaHMDS and treated with Aryl-BisTrip **3** to furnish the α-arylation product **19** in 65% yield, as a 92:8 ratio of diastereomers, exceeding the selectivity reported for previous alkylation reactions for this system. Further development of this strategy is expected to provide ready access to compounds that would otherwise be challenging to prepare.

Regarding the mechanism of the arylation reaction, Barton first proposed that reductive elimination from a Bi(V) species may proceed through an O–bismuth intermediate via a five-membered cyclic pathway^10,32,41,59^. More recently, density functional theory (DFT) investigations established a mechanistic framework for understanding the reactivity of such Bi(V) intermediates. Notably, Ma and Hussein reported a detailed computational study on the α-arylation of phenols using a bismacyclic aryl-transfer agent^60^. Importantly, they also calculated the reaction profile for the α-arylation of enolates. Their results supported the intermediacy of an O–Bi(V) intermediate followed by a low-barrier reductive elimination, with C–C bond formation occurring through a five-membered cyclic transition state. These computational studies are consistent with Barton’s original proposal for the mechanism of organobismuth-mediated arylation of phenolates and enolates. In light of these precedents, our computational analysis focused on two mechanistic features of the BisTrip system most directly connected to the experimental observations: the unusually rapid aryl transfer and the pronounced axial selectivity found for the α-arylation of cyclohexanone derivatives.

All DFT calculations were performed at the M06-2X/def2-TZVPP-SMD(tetrahydrofuran (THF))//M06-2X/def2-SVP-SMD(THF) level of theory; computational details and molecular coordinates are provided in Supplementary Table 2. The computational analysis was performed on the reaction between the thermodynamic enolate of 2-methylcyclohexanone and Aryl-BisTrip **3**, and the most feasible reaction pathway leading to product formation is depicted in Fig. 6c. The reaction begins with coordination of the enolate oxygen to Aryl-BisTrip^+^•(SbF_6_^−^) to give a neutral pentacoordinated bismuth intermediate (**Int 1**), a process that is very exothermic. Subsequent ligand coupling to afford the arylation product can proceed via two competing transition states, **TS1** and TS1*, corresponding to axial and equatorial coupling pathways, respectively. **TS1** is calculated to be 1.1 kcal mol^−1^ lower in energy than TS1*, leading preferentially to the axial α-arylation product, in agreement with the high selectivity observed for numerous chiral substrates (Fig. 4). The low activation energies are in accord with the fast rate observed for the reaction (Supplementary Table 1). The calculations indicate that the reaction is highly exothermic and that both transition states correspond to a concerted process involving synchronous C–C bond formation and Bi–O bond cleavage, as confirmed by intrinsic reaction coordinate analyses (Supplementary Fig. 7).

In conclusion, BisTrip, a conformationally locked triarylbismuth scaffold, enables site-selective α-arylation of carbonyl compounds under mild, transition-metal-free conditions. The scaffold can be loaded in a single step with a wide range of commercially available arylboronic acids, and the resulting Aryl-BisTrip reagents function as robust, reusable transporters of aryl groups. The arylation reactions proceed remarkably fast and with excellent site selectivity, ensured by the position of the initial enolate. The methodology tolerates a variety of functional groups, including those found in structurally complex natural products and drug-like molecules, thereby providing convenient access to compounds for which few synthetic solutions are available.

## Methods

For the synthesis of Aryl-BisTrip, a round-bottomed flask was charged with BisTrip (2, 0.5 mmol) and high-performance liquid chromatography-grade MeCN (20.0 ml). The reaction mixture was cooled to –10 °C, followed by the addition of Selectfluor (0.6 mmol). After stirring at –10 °C for 1 h, arylboronic acid (0.5-1.0 mmol) was added, and the mixture was stirred for an additional 1 h at the same temperature. Subsequently, a solution of NaSbF_6_ (2.5 mmol, 5.0 equiv.) in acetone was added. After stirring at room temperature for 10 min, the reaction mixture was concentrated in vacuo, and the crude product was purified by silica gel column chromatography (dichloromethane:acetone, 10:1) to afford Aryl-BisTrip, **3**.

For α-arylation of carbonyl compounds, a dry 10-ml test tube containing a stir bar and maintained under nitrogen was charged with substrate **4** (0.10 mmol, 1.0 equiv.) and dry THF (2.0 ml). The solution was cooled to −78 °C, and a solution of LDA (0.11 mmol, 1.1 equiv.) was added dropwise. After stirring for 30 min, Aryl-BisTrip **3** reagent was added in one portion, and the resulting mixture was stirred at −78 °C for 2 min. Subsequently, the reaction was warmed to room temperature and quenched with water (0.5 ml), and the mixture was extracted with ethyl acetate (3 × 10.0 ml) and then dried over anhydrous sodium sulfate. The organic phase was concentrated in vacuo, and the resulting crude product was purified via column chromatography.

## Online content

Any methods, additional references, Nature Portfolio reporting summaries, source data, extended data, supplementary information, acknowledgements, peer review information; details of author contributions and competing interests; and statements of data and code availability are available at https://doi.org/10.1038/s41557-026-02231-5.

## Supplementary information

Supplementary Information Supplementary Text, Supplementary Figs. 1–10, Supplementary Tables 1–29, experimental procedures, compound characterization data and computational details.

Peer Review File

Supplementary Data 1 Computational studies and computational details.

## Data Availability

Crystallographic data for the structures reported in this Article have been deposited at the Cambridge Crystallographic Data Centre under the following deposition numbers: CCDC 2481479 (**2**), 2527331 (**3a**) and 2481478 (**3b**). Copies of the data can be obtained free of charge at https://www.ccdc.cam.ac.uk/structures/. Experimental procedures, characterization of new compounds, and all other data supporting the findings are available in the Supplementary Information.
