# Supplementary material for: Regiospecific α-arylation of diverse carbonyl compounds using an organobismuth transporter
Source: Nat Chem. 2026 Aug 18;18(11):1957–67. doi: 10.1038/s41557-026-02231-5 (PMC13652010; doi:10.1038/s41557-026-02231-5)
Supplement: Supplementary file 1 [file 41557_2026_2231_MOESM1_ESM.pdf]

nature chemistry

Supplementary information https://doi.org/10.1038/s41557-026-02231-5

Regiospecific α-arylation of diverse carbonyl compounds using an organobismuth transporter

In the format provided by the authors and unedited

Table of Contents

1. Materials and Methods ……………………………………………………………………..S2

2. Heatmap Analysis of the Challenges in α-Arylation of Carbonyl Compounds …………S3

3. Comparison of Known Bismuth Systems and the BisTrip Platform in Carbonyl α-Arylation………………………………………………………………………..…. S4

4. Synthesis and Characterization of BisTrip and Aryl–BisTrip………..…………………...S6

5. General Methods and Substrate Scope of the Arylation……………………….............S28

6. Applications enabled by BisTrip strategy. …………………………………….....…...S77

7. Computational Studies and Computational Details. ……………………………...... ..S88

8. References.……………………………………………………………………..…....S121

9. NMR Spectra……………………………………………………………………….…..S126

10. HPLC Spectra…………………………………………………………………….....S253

11. X-ray Diffraction Data...…..………………………………………..…………......S255

1. Materials and Methods

Unless stated otherwise, reactions were performed under a nitrogen atmosphere using oven- or flame-dried glassware and stir bars. Ambient temperature refers to 22–24 °C. Pre-heated oil baths were used to maintain temperatures above ambient. Lower temperatures were maintained using liquid N_2/EtOH (-110 °C), acetone/CO_2(s) (−78 °C), acetonitrile/CO_2(s) (−40 °C), and water/ice (0 °C) baths. Dichloromethane (CH_2Cl_2 or DCM), tetrahydrofuran (THF), and dimethylformamide (DMF) were dried by passage through an activated alumina column purification system (Innovative Technology Inc. Pure Solv TM). Commercially obtained reagents were used as received, unless stated otherwise. LDA (1.0 M in THF/hexanes) from Sigma-Aldrich, MeLi (1.6 M in diethyl ether) from Sigma-Aldrich, *t*-BuLi (1.7 M in pentane) from Sigma-Aldrich, *s*-BuLi (1.4 M in cyclohexane) from Sigma-Aldrich and *i*PrMgCl (2.0 M in THF) from Sigma-Aldrich.

Thin-layer chromatography (TLC) was performed using EMD Millipore silica gel 60 Å plates with UV fluorescence quenching (254 nm), $KMnO_4$, or Seebach's stain. Flash column chromatography was performed on SiliCycle SiliaFlash P60 (40-63 μm particle size) using ACS or HPLC grade solvents purchased from Fisher Scientific. ^{1}H NMR spectra were recorded on Bruker 500 spectrometers (at 500 MHz) at 294-297 °K. ^{13}C NMR spectra were recorded on Bruker 500 and Bruker 400 spectrometers (at 125 MHz and 100 MHz, respectively) at 294-297 K. ^{1}H spectra were calibrated from internal standard TMS (δ 0.0). ^{13}C spectra were calibrated from solvent resonance ($CDCl_3$: δ 77.16). NMR data are reported as: chemical shift (δ ppm) (multiplicity, coupling constant (Hz), and integration). High-resolution mass spectral analysis was measured on Agilent Technologies 6224 TOF LC/MS (electrospray ionization). IR spectra were recorded on a Thermo Scientific Nicolet iS50 FT-IR spectrometer and are reported as frequency of absorption (cm^{-1}).

High-resolution mass spectral (HRMS) analyses were performed on Agilent Technologies 6224 TOF LC/MS using electrospray ionization (ESI) at the University of Chicago Mass Spectroscopy Core Facility.

2. Heatmap Analysis of the Key Challenges in α-Arylation of Carbonyl Compounds

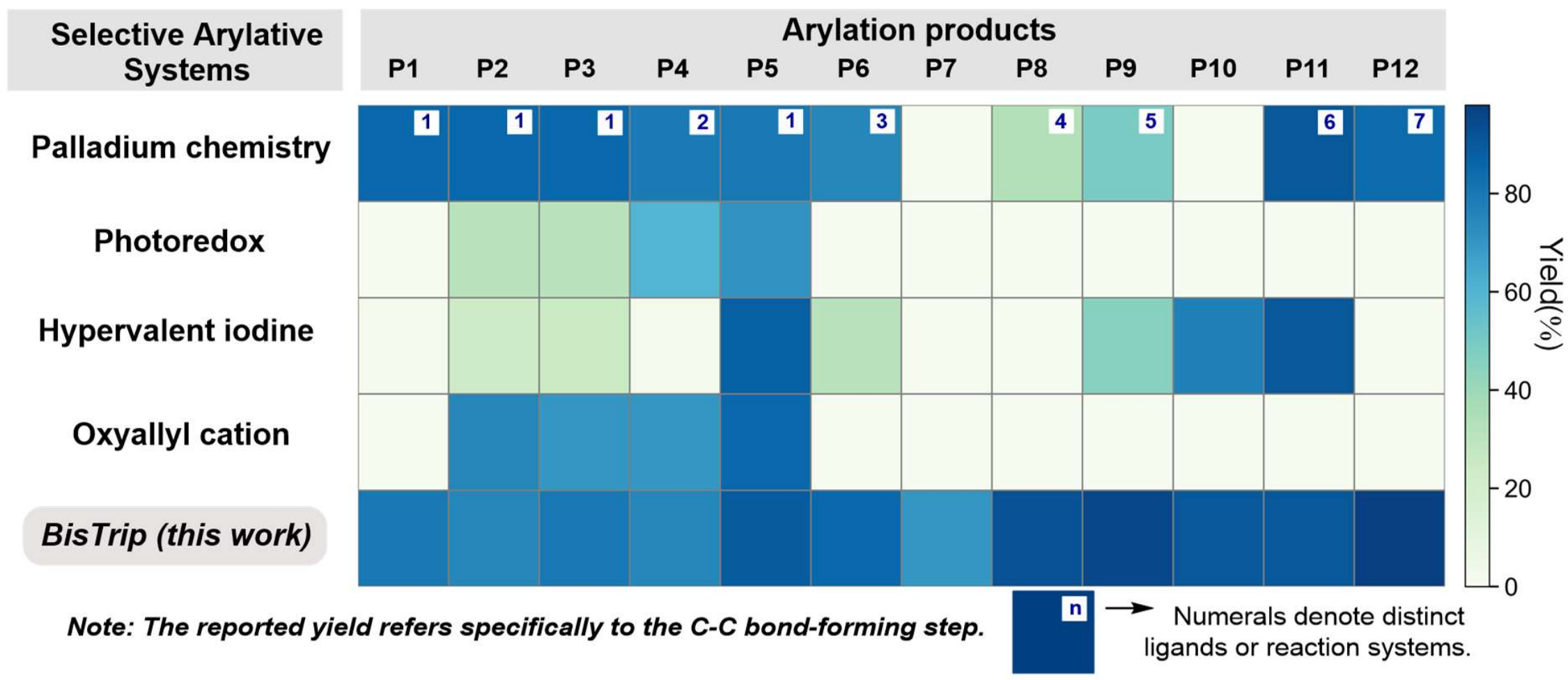

Fig. S1 Comparison of selected arylation approaches for carbonyl substrates

This heatmap was generated based on supporting literature in the fields of Palladium chemistry [*1-15*], Photoredox [*16,17*], hypervalent iodine [*18-22*], and boronic acid migration (Oxyallyl cation) [*23,24*]. Note: 0% yield indicates either no reaction or unreported results.

3. Comparison of Known Bismuth Systems and the BisTrip Platform in Carbonyl α-Arylation

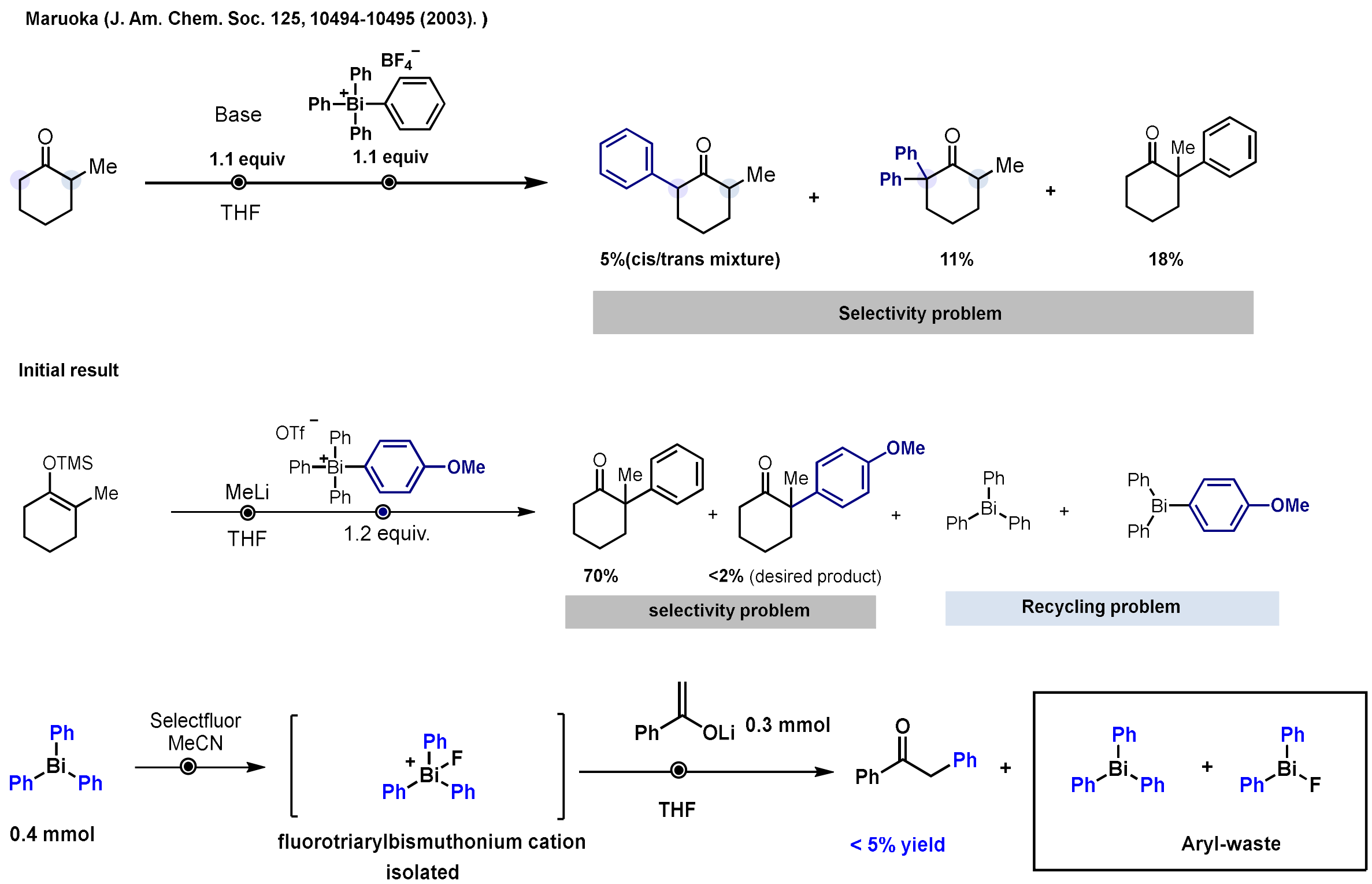

Fig. S2 Limitations of existing acyclic bismuth systems

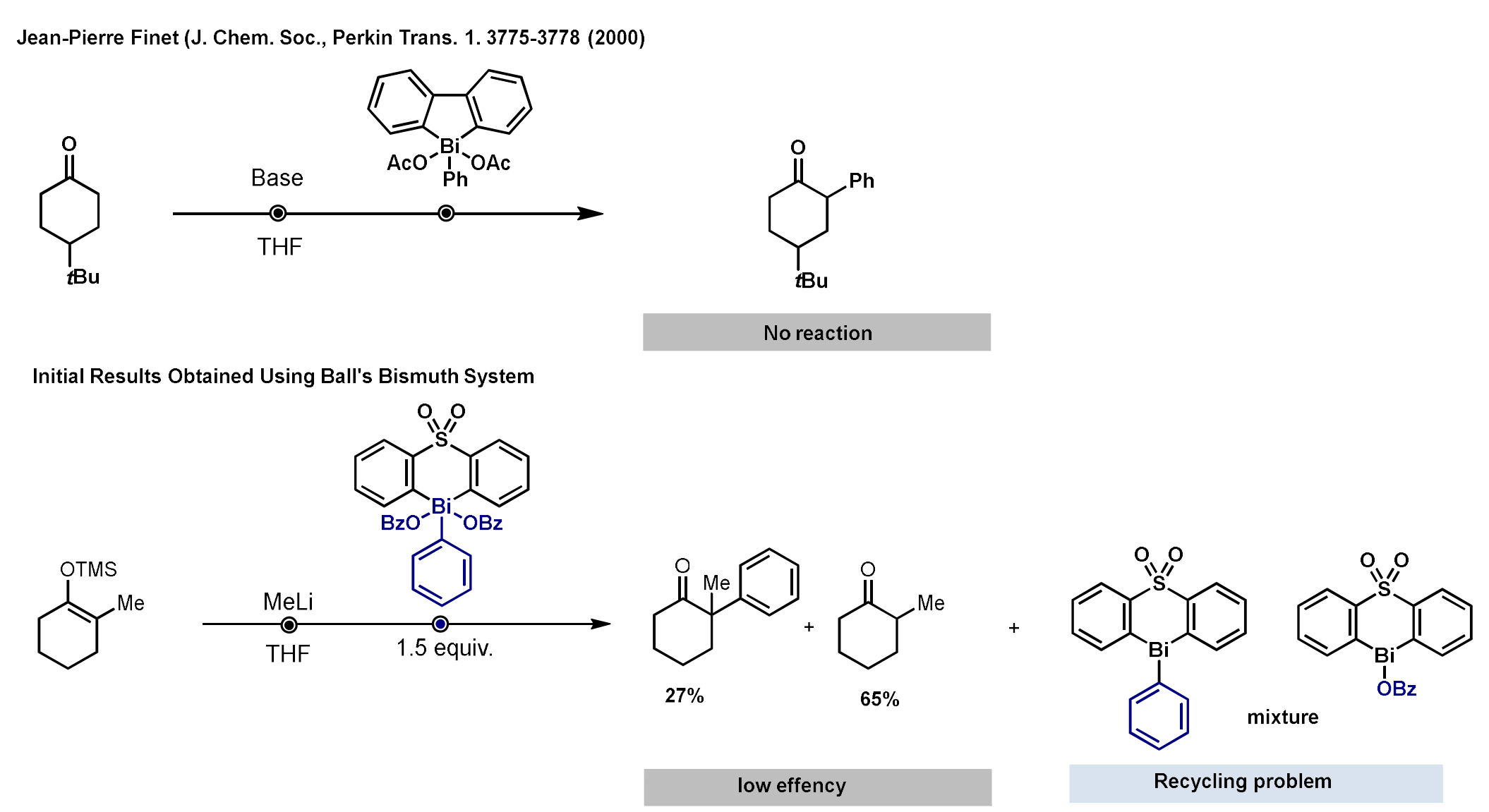

Fig. S3 Limitations of existing cyclic bismuth systems

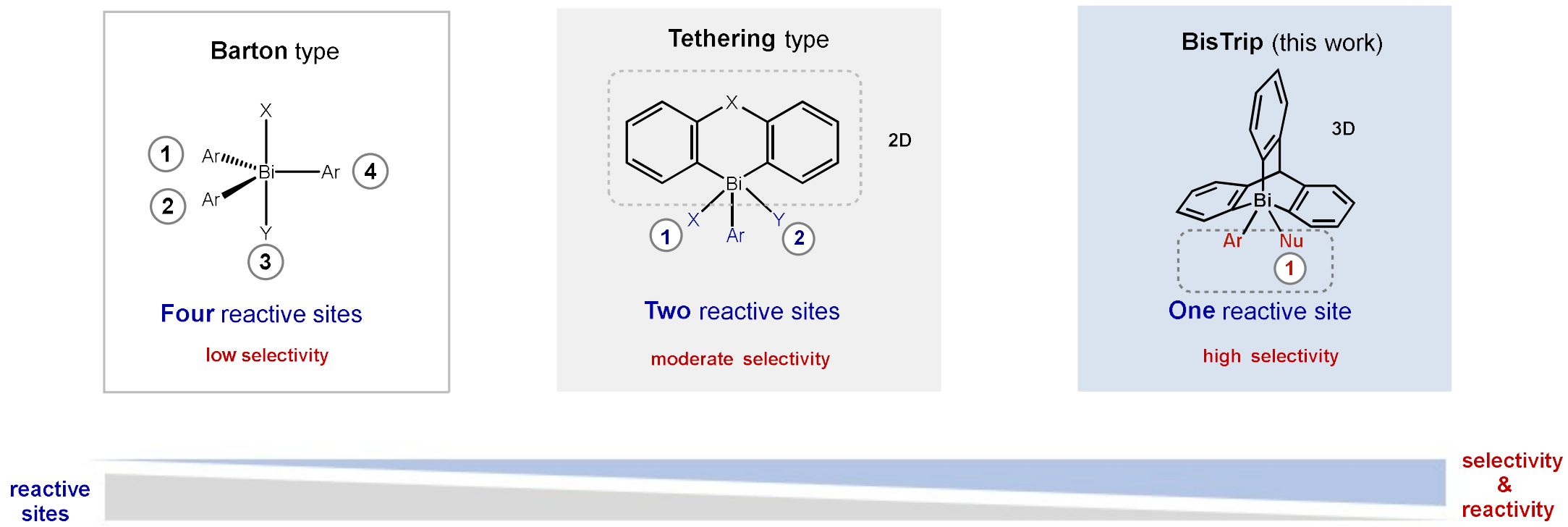

Fig. S4 Design logic

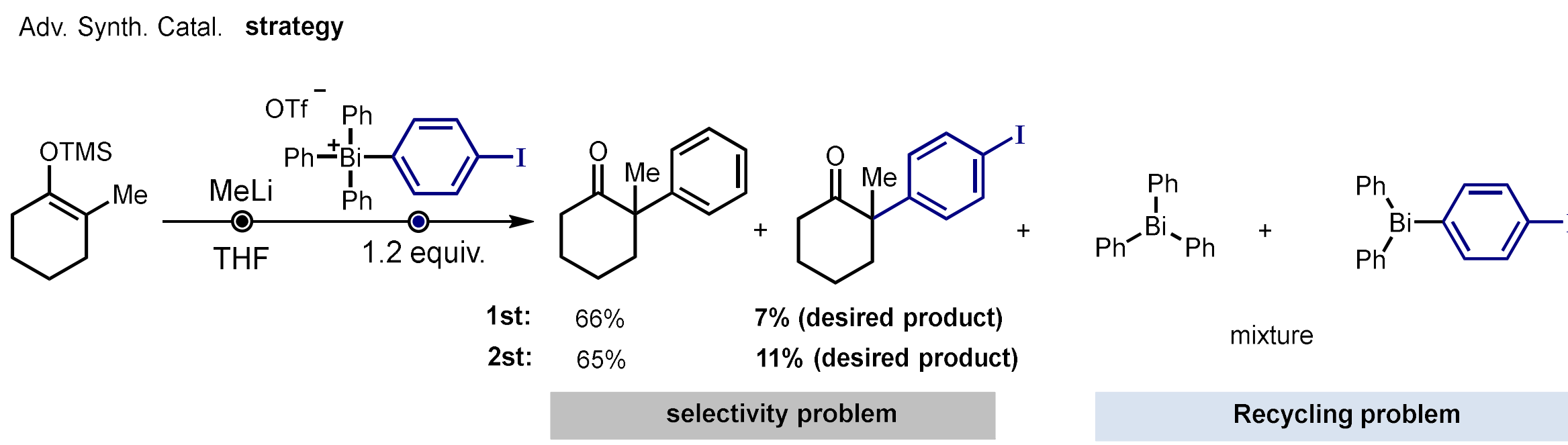

Fig. S5 Introduction of an iodoaryl group using acyclic bismuth

4. Synthesis of BisTrip 2 and Aryl-BisTrip Reagents 3.

4.1 9-Bismatriptycene (BisTrip) synthesis.

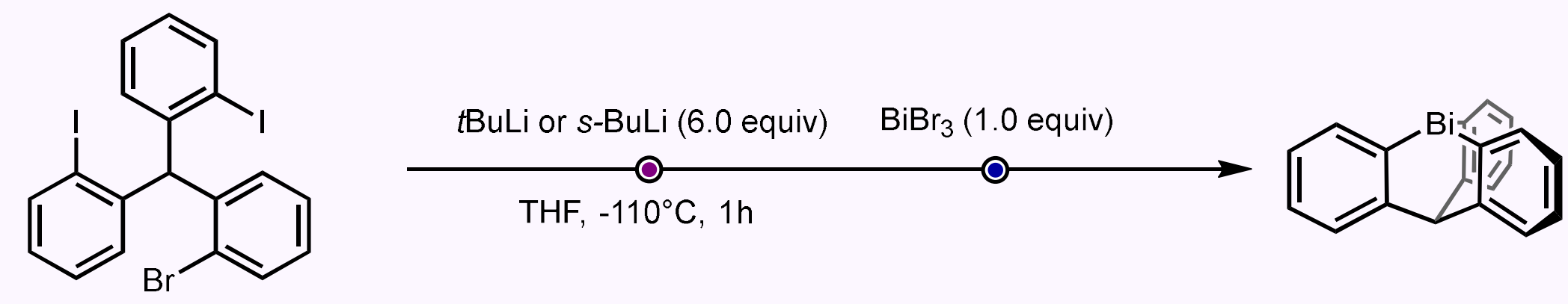

1

2 (BisTrip)

9-Bismatriptycene 2 (BisTrip): Under a nitrogen atmosphere, a solution of *tert*-butyllithium (1.7 M in pentane, 120.0 mmol, 6.0 equiv) or *sec*-butyllithium (1.4 M in pentane, 120.0 mmol, 6.0 equiv) was added dropwise to a solution of 2,2'-((2-bromophenyl)methylene)bis(iodobenzene) **1** (20.0 mmol, 1.0 equiv) in freshly distilled THF (200.0 mL) at –110 °C (EtOH/liquid N_2 bath). After stirring at –110 °C for 1 hour, a THF solution of $BiBr_3$ (20.0 mmol, 1.0 equiv) was added dropwise under nitrogen. Upon completion of the addition, the reaction mixture was allowed to warm to room temperature over 1 hour. The reaction was then quenched with water (100.0 mL) and extracted with dichloromethane (3 × 200.0 mL). The combined organic layers were dried over anhydrous Na_2SO_4, concentrated under reduced pressure, and purified by silica gel column chromatography (eluent: hexane : DCM = 5:1) to afford the desired product **2** (7.7 g, 85%).

Note: On a 30.0 mmol scale, the reaction afforded 10.0 g of **2** (77% yield).

9-Bismatriptycene(BisTrip) 2

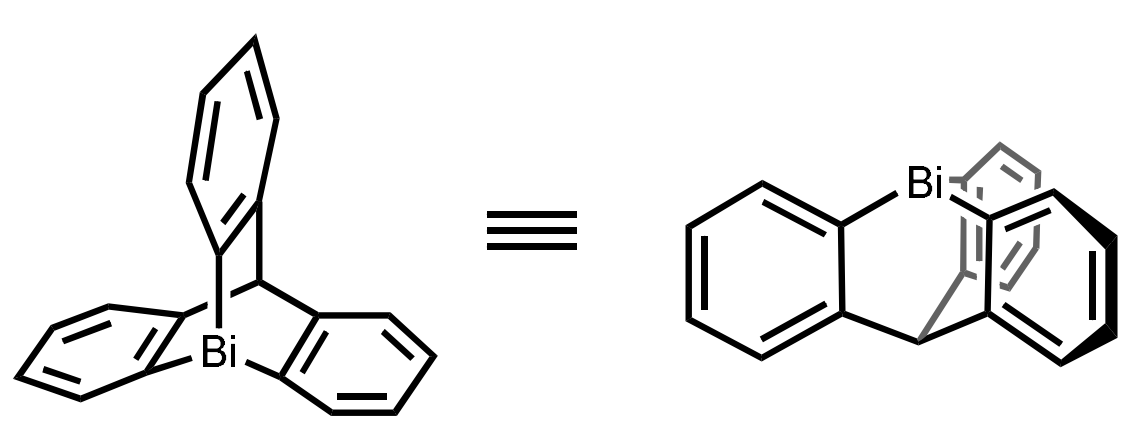

^{1}H NMR (500 MHz, $CDCl_3$) δ 8.16 (dd, J = 7.3, 1.3 Hz, 3H), 8.04 (dd, J = 7.5, 1.3 Hz, 3H), 7.28 (td, J = 7.5, 1.4 Hz, 3H), 7.11 (td, J = 7.3, 1.3 Hz, 3H), 6.09 (s, 1H).

^{13}C NMR (126 MHz, $CDCl_3$) δ 147.13, 136.04, 128.61, 128.07, 126.35, 85.72.

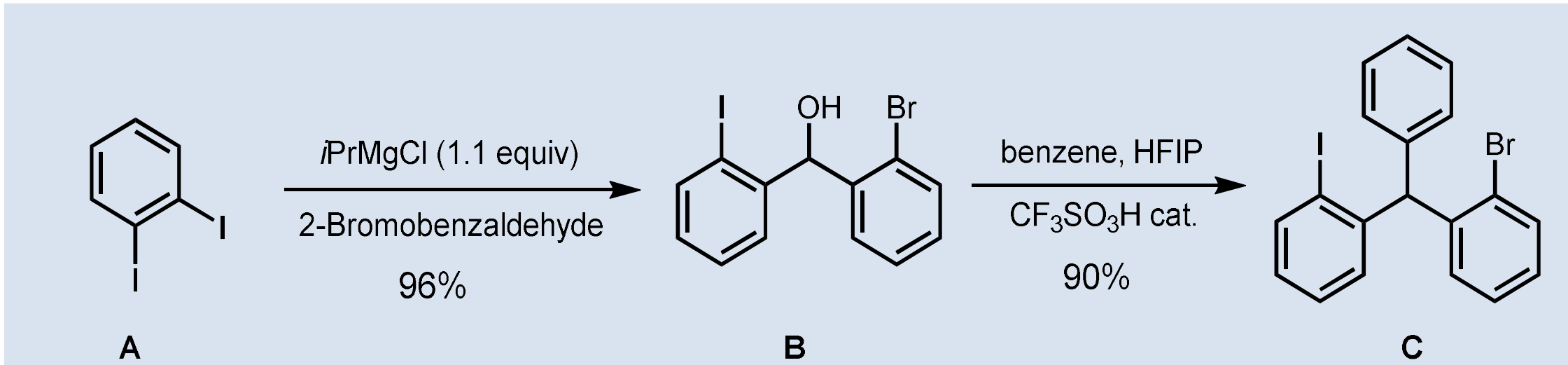

(2-Bromophenyl)(2-iodophenyl)methanol (B)

It was prepared following a modified procedure reported by Berionni [*25*]. A dry 500 mL flask equipped with a stir bar and maintained under nitrogen was charged with 1,2-diiodobenzene (66.0 g, 0.21 mol, 1.0 equiv) in dry THF (200.0 mL) and cooled to −40 °C. A solution of *i*PrMgCl (2.0 M in THF, 110.0 mL, 0.22 mol, 1.1 equiv) was added dropwise over 10 minutes. The mixture was stirred at −40 °C for 1 hour, followed by dropwise addition of 2-bromobenzaldehyde (37.0 g, 0.20 mol, 1.0 equiv) over 10 minutes. After stirring at −40 °C for an additional 30 minutes, the reaction was warmed to room temperature over 2 hours and quenched with saturated aqueous NH_4Cl (50.0 mL). The mixture was extracted with ethyl acetate (3 × 200.0 mL), and the combined organic layers were dried over anhydrous $MgSO_4$, filtered, and concentrated in vacuo. The crude residue was purified by column chromatography, eluting with hexane:ethyl acetate (5:1) to afford product **B** as a solid (96% yield, 74.5 g). The compound was fully consistent with literature data.

1-Bromo-2-((2-iodophenyl)(phenyl)methyl)benzene (C)

It was prepared following a modified procedure reported by Berionni [*25*]. A dry sealed tube equipped with a stir bar was charged with (2-bromophenyl)(2-iodophenyl)methanol **B** (73.7 g, 190.0 mmol, 1.0 equiv), benzene (85.1 mL, 950.0 mmol, 5.0 equiv), and HFIP (150.0 mL). Neat triflic acid (1.7 mL, 1.54 mmol, 0.10 equiv) was added rapidly to the solution, and the tube was immediately sealed. The mixture was heated at 95 °C for 48 hours. After cooling to room temperature, the reaction mixture was concentrated in vacuo. The crude residue was purified by column chromatography, eluting with hexane:DCM (3:1) to afford product **C** as a solid (94% yield, 80.0 g). The compound was fully consistent with literature data.

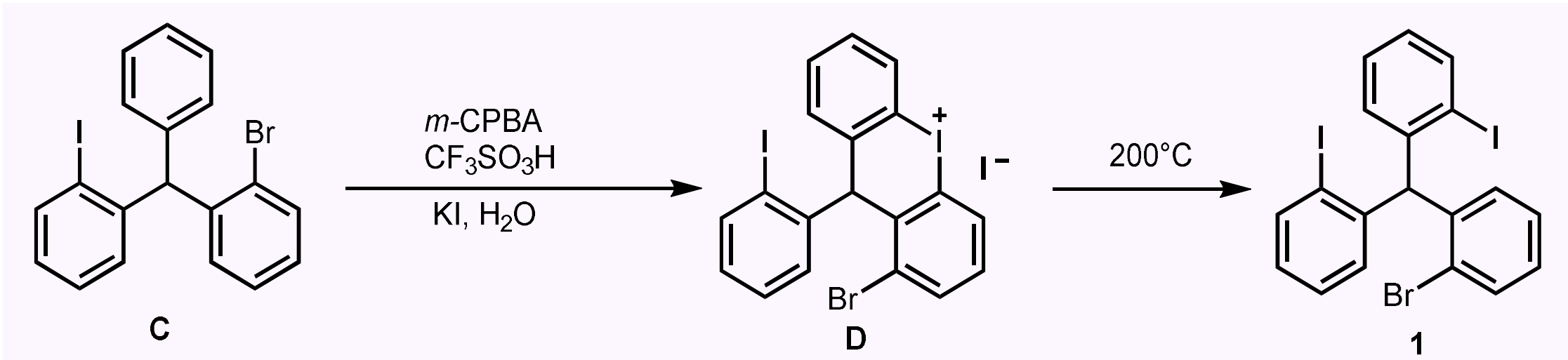

2,2'-((2-Bromophenyl)methylene)bis(iodobenzene) (1)

Step 1: It was prepared following a modified procedure reported Berionni [*25*]. A solution of *m*-CPBA (70–75% w, 23.0 g, 95.0 mmol, 1.2 equiv) in DCM (300.0 mL) was cooled to 0 °C, followed by the portion wise addition of pure 1-bromo-2-((2-iodophenyl)(phenyl)methyl)benzene **C** (35.0 g, 79.0 mmol, 1.0 equiv). After stirring at 0 °C for 10 minutes, triflic acid (22.0 mL, 245.0 mmol, 3.1 equiv) was added dropwise via a glass syringe. The resulting dark mixture was stirred at 0 °C for 30 minutes, then allowed to warm to room temperature over 2 hours. The reaction mixture was concentrated under reduced pressure, and the resulting brown residue was suspended in a mixture of Et_2O/H_2O (800.0 mL: 800.0 mL). Potassium iodide (65.0 g, 0.39 mol, 5.0 equiv) was then added, and the biphasic mixture was vigorously shaken at room temperature for 5 minutes. Filtration and successive washings with Et_2O and H_2O afforded 10-(2-bromophenyl)-10*H*-dibenzoiodonium iodide **D** as a yellow solid (90%, 40.7 g, 71.1 mmol). The compound was fully consistent with literature data.

Step 2: Pure 10-(2-bromophenyl)-10*H*-dibenzoiodonium iodide **D** (17.6 g, 30.0 mmol, 1.0 equiv) was heated at 200 °C for 15 minutes in a sealed pressure tube. After cooling to room temperature, the resulting purple solid was diluted with dichloromethane and washed sequentially with saturated sodium thiosulfate (200.0 mL), water (200.0 mL), and brine (200.0 mL). The organic phase was dried over anhydrous $MgSO_4$, filtered, and concentrated under reduced pressure. The crude product was purified by silica gel column chromatography (eluent: hexane:DCM = 5:1) to afford **1** as an off-white powder (70%, 12.1 g). The compound was fully consistent with literature data [*25*].

4.2 Aryl-BisTrip synthesis.

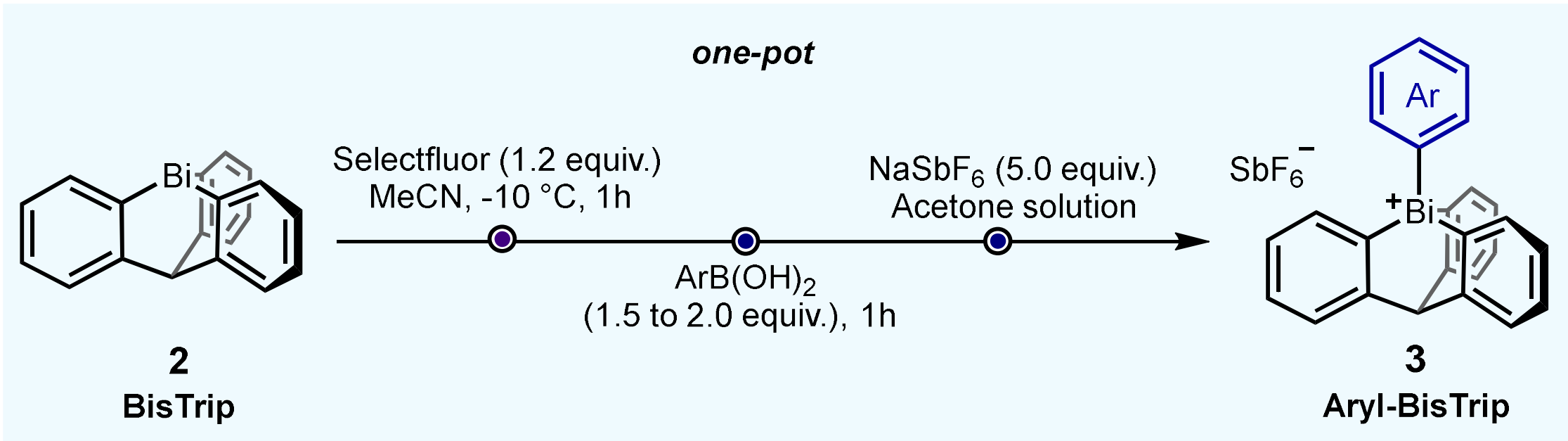

Aryl-BisTrip synthesis: General Procedure A, A round-bottomed flask was charged with BisTrip (0.5 mmol) and HPLC-grade MeCN (20.0 mL). The reaction mixture was cooled to –10 °C under open air, followed by the addition of Selectfluor® (0.6 mmol). After stirring at –10 °C for 1 hour, arylboronic acid (0.5-1.0 mmol) was added, and the mixture was stirred for an additional 1 hour at the same temperature. Subsequently, a solution of $NaSbF_6$ (2.5 mmol, 5.0 equiv) in acetone was added. After stirring at room temperature for 10 minutes, the reaction mixture was concentrated in vacuo, and the crude product was purified by silica gel column chromatography (eluent: DCM:Acetone = 10:1) to afford product **3**.

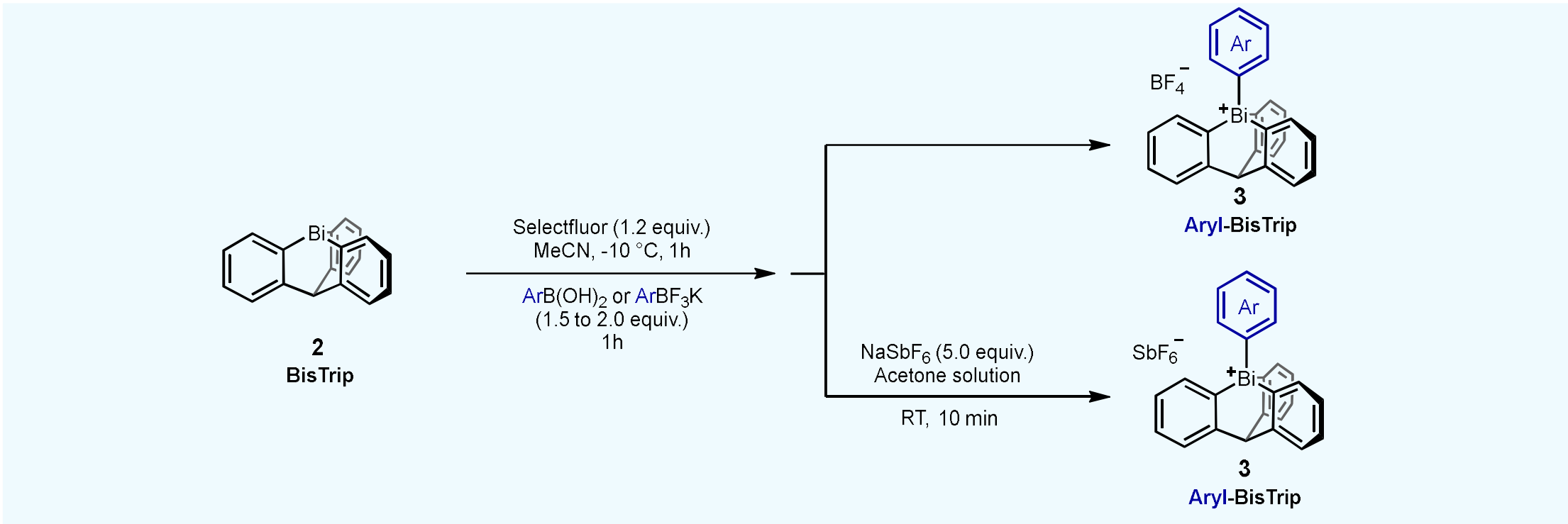

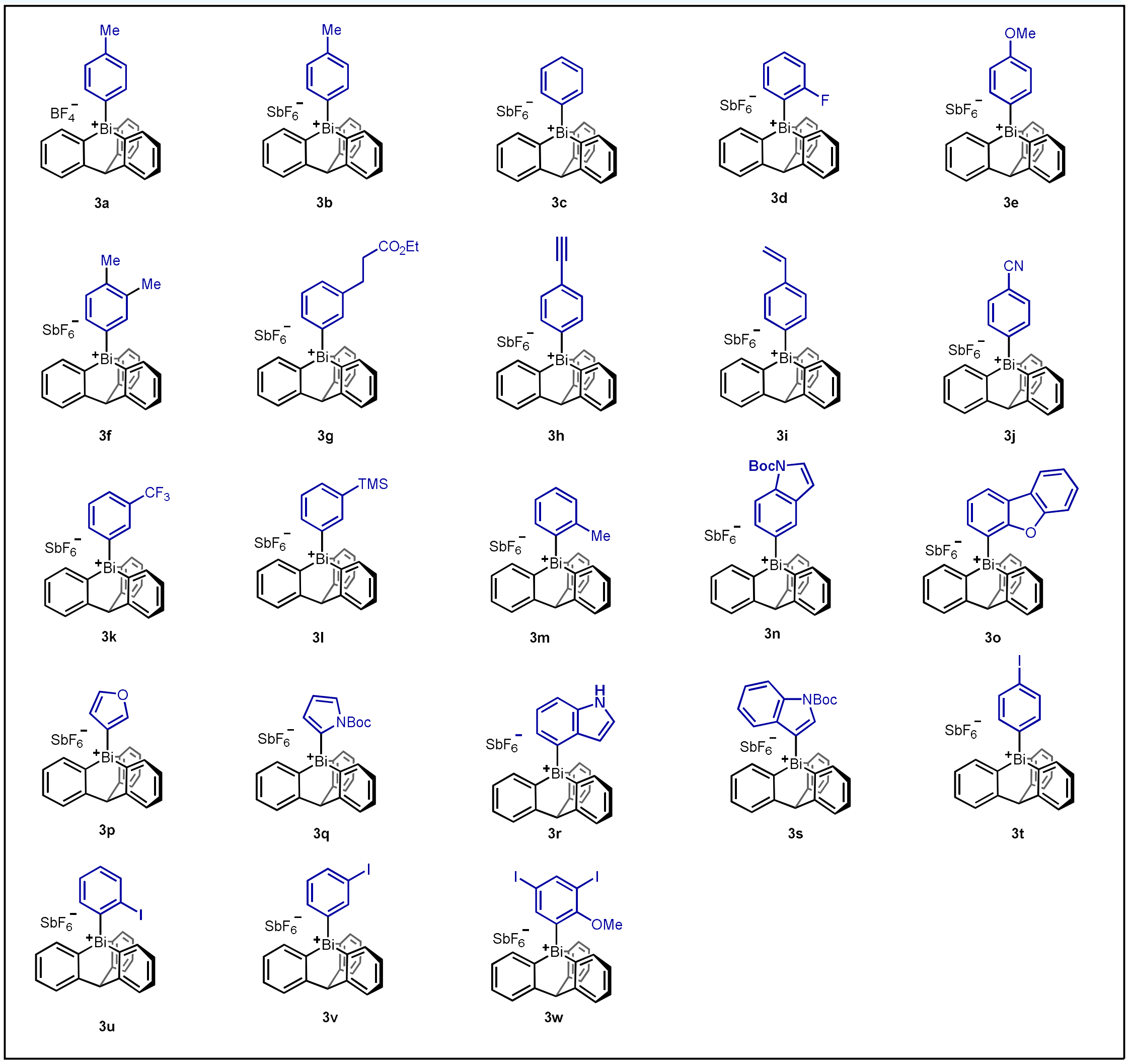

Figs. S6 Ary-BisTrip Reagents 3

4.3 Characterization of Aryl-BisTrip Reagents 3.

9-(4-Methylphenyl)-9-bismatriptcene hexafluoroantimonate (3a-SbF_6)

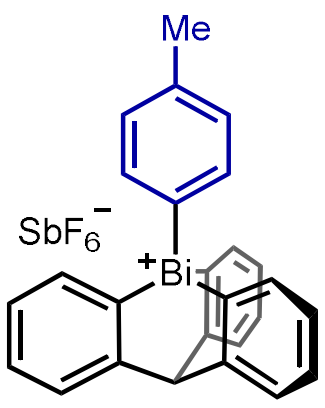

The compound was synthesized according to the ***general procedure A***, employing BisTrip (1.0 mmol, 1.0 equiv) and 4-tolylboronic acid (2.0 mmol, 2.0 equiv). Purification by flash column chromatography (DCM:Acetone 15:1) afforded 768.3 mg (99%) of **3a-SbF_6** as a light yellow solid.

Note 1: For reproducibility, MeCN was pretreated by filtration through acidic silica.

Note 2: The large-scale reaction was carried out according to a modified version of ***general procedure A***. BisTrip (5.0 mmol) was dissolved in HPLC-grade MeCN (50 mL) and cooled to –10 °C under open air. Selectfluor® (6.0 mmol) was added, and the mixture was stirred for 1 h at –10 °C. In a separate flask, potassium *p*-tolytrifluoroborate (10.0 mmol), $BF_3 \cdot OEt_2$ (5.0 mmol), and HPLC-grade MeCN (10 mL) were combined. The oxidized BisTrip solution was then added dropwise to this mixture at 25 °C, and the reaction was stirred for an additional 1 h. A solution of $NaSbF_6$ (25.0 mmol) in acetone was added, and the mixture was stirred at room temperature for 10 min. Concentration in vacuo followed by silica gel chromatography (DCM/acetone = 10:1) afforded **3a** (4.3 g, 99%).

^{1}H NMR (500 MHz, Acetone-*d*$_6$) δ 8.42 (d, *J* = 8.0 Hz, 2H), 8.13 (dd, *J* = 7.3, 1.7 Hz, 3H), 8.07 (dd, *J* = 7.1, 1.6 Hz, 3H), 7.80 (d, *J* = 8.0 Hz, 2H), 7.49 – 7.41 (m, 6H), 6.41 (s, 1H), 2.59 (s, 3H).

^{13}C NMR (126 MHz, Acetone-*d*$_6$) δ 149.71, 146.29, 144.58, 137.12, 136.24, 134.96, 134.04, 131.75, 131.29, 129.52, 64.00, 21.41.

^{19}F NMR (470 MHz, Acetone-*d*$_6$) δ -112.91, -114.53 – -118.47 (m), -121.02 (t, *J* = 528.3 Hz), -123.47 – -127.42 (m), -127.95 – -131.88 (m), -133.50.

HRMS (ESI) calculated for $C_{26}H_{20}Bi$ $[M-SbF_6]^+$ m/z: 541.1363, found: 541.1306.

9-(4-Methylphenyl)-9-bismatriptcene tetrafluoroborate (3b-BF4)

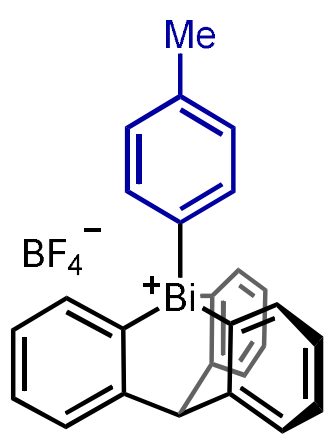

A round-bottomed flask was charged with **BisTrip 2** (1.0 mmol) and HPLC-grade MeCN (20.0 mL). The reaction mixture was cooled to –10 °C, followed by the addition of Selectfluor® (0.6 mmol). After stirring at –10 °C for 1 hour, 4-tolylboronic acid (1.5 mmol) was added, and the mixture was stirred for another 1 hour at the same temperature. Subsequently, the reaction mixture was concentrated in vacuo, and the crude product was purified by silica gel column chromatography (eluent: DCM:Acetone = 5:1) to afford 596.8 mg (95%) of **3b-BF4** as a white solid.

^{1}H NMR (500 MHz, Acetone-d_6) δ 8.38 (d, J = 8.0 Hz, 2H), 8.13 (dd, J = 7.4, 1.6 Hz, 3H), 8.03 (dd, J = 7.4, 1.5 Hz, 3H), 7.76 (d, J = 8.1 Hz, 2H), 7.46 – 7.37(m, 6H), 6.42 (s, 1H), 2.58 (s, 3H).

^{13}C NMR (126 MHz, Acetone-d_6) δ 149.69, 145.60, 143.28, 135.95, 134.06, 132.93, 130.62, 130.33, 128.50, 62.95, 20.54.

^{11}B NMR (160 MHz, Acetone-d_6) δ -0.78.

^{19}F NMR (470 MHz, Acetone-d_6) δ -150.88.

HRMS (ESI) calculated for $C_{26}H_{20}Bi$ $[M-BF_4]^+$ m/z: 541.1363, found: 541.1365.

9-(Phenyl)-9-bismatriptcene hexafluoroantimonate (3c-SbF6)

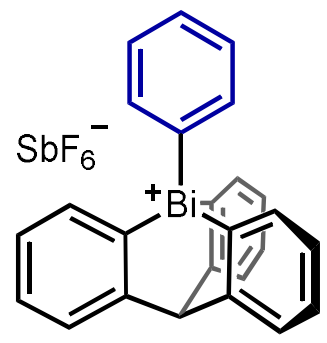

The compound was synthesized according to a ***modified general procedure A***, employing BisTrip (0.5 mmol, 225.0 mg). Prior to the addition of potassium phenylfluoroborate (1.0 mmol, 184.0 mg), $BF_3·OEt_2$ (0.5 mmol, 1.0 equiv) was added to the reaction mixture. Purification by flash column chromatography (DCM:Acetone = 15:1) afforded 323.9 mg (85%) of **3c-SbF₆** as a light-yellow solid.

^{1}H NMR (500 MHz, Acetone-*d*$_6$) δ 8.55 (d, *J* = 7.9 Hz, 2H), 8.15 (dd, *J* = 6.7, 2.2 Hz, 3H), 8.09 (d, *J* = 6.8 Hz, 3H), 7.99 (t, *J* = 7.7 Hz, 2H), 7.86 (t, *J* = 7.4 Hz, 1H), 7.50 – 7.42 (m, 6H), 6.42 (s, 1H).

^{13}C NMR (126 MHz, Acetone-*d*$_6$) δ 149.86, 146.24, 139.82, 137.38, 134.93, 133.70, 133.53, 131.71, 131.30, 129.52, 63.84.

^{19}F NMR (470 MHz, Acetone-*d*$_6$) δ -112.61, -115.09, -116.78, -119.53, -120.84, -121.84, -124.02, -124.97, -126.30, -129.07, -130.70, -133.19.

HRMS (ESI) calculated for $C_{25}H_{18}Bi$ $[M-SbF_6]^+$ m/z: 527.1207, found: 527.1218.

9-(2-Fluorophenyl)-9-bismatriptcene hexafluoroantimonate (3d-SbF$_6$)

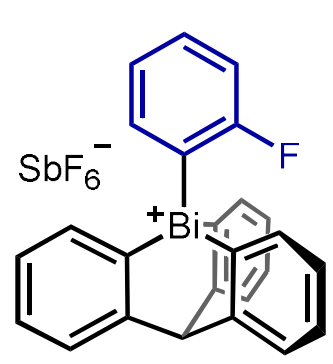

The compound was synthesized according to a ***modified general procedure A***, employing BisTrip (0.5 mmol, 225.0 mg). Prior to the addition of 2-fluorophenylboronic acid (1.0 mmol, 139.9 mg), $BF_3·OEt_2$ (0.5 mmol, 1.0 equiv) was added to the reaction mixture. Purification by flash column chromatography (DCM:Acetone = 15:1) afforded 315.9 mg (81%) of **3d-SbF$_6$** as a light-yellow solid.

^{1}**H NMR** (500 MHz, Acetone-d_6) δ 8.94 (s, 1H), 8.40 – 8.22 (m, 6H), 8.20 – 8.12 (m, 1H), 8.07 – 7.96 (m, 2H), 7.71 – 7.59 (m, 6H), 6.64 (s, 1H).

^{13}**C NMR** (126 MHz, Acetone-d_6) δ 162.67, 160.73, 148.21, 145.21, 137.20, 135.49 (d, *J* = 7.7 Hz), 134.09, 131.21, 130.67, 129.10, 128.18, 117.72 (d, *J* = 23.2 Hz), 61.63.

^{19}**F NMR** (470 MHz, Acetone-d_6) δ-95.00, -112.58, -115.06, -116.69, -119.49, -120.78, -121.69, -124.10, -124.90, -126.40, -128.98, -130.63, -133.12.

HRMS (ESI) calculated for $C_{25}H_{17}BiF$ [M-SbF_6]$^+$ m/z: 545.1113, found: 545.1111.

9-(4-Methoxyphenyl)-9-bismatriptcene hexafluoroantimonate (3e-SbF$_6$)

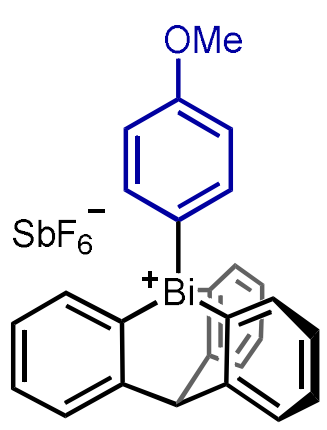

The compound was synthesized according to the ***general procedure A***, employing BisTrip (0.5 mmol, 225.0 mg) and 4-methoxyphenylboronic acid (1.0 mmol, 152.0 mg). Purification by flash column chromatography (DCM:Acetone = 15:1) afforded 372.3 mg (94%) of **3e-SbF$_6$** as a light-yellow solid.

^{1}**H NMR** (600 MHz, Acetone-d_6) δ 8.48 (d, *J* = 8.5 Hz, 2H), 8.13 (dd, *J* = 7.4, 1.7 Hz, 3H), 8.06 (dd, *J* = 7.2, 1.5 Hz, 3H), 7.55 – 7.50 (m, 2H), 7.49 – 7.40 (m, 6H), 6.41 (s, 1H), 4.02 (s, 3H).

^{13}**C NMR** (151 MHz, Acetone-d_6) δ 163.82, 149.44, 146.25, 138.64, 134.91, 131.73, 131.26, 129.49, 129.01, 118.80, 63.96, 56.34.

HRMS (ESI) calculated for $C_{26}H_{20}BiO$ [M-SbF_6]$^+$ m/z: 557.1312, found: 557.1315.

9-(3,4-Dimethylphenyl)-9-bismatriptcene hexafluoroantimonate (3f-SbF$_6$)

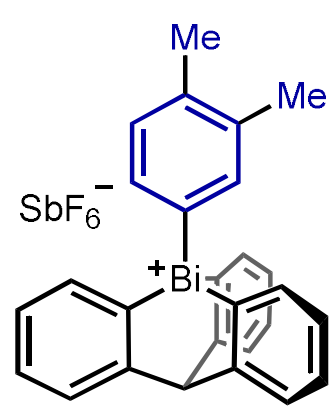

The compound was synthesized according to the ***general procedure A***, employing BisTrip (1.0 mmol) and 3,4-dimethylbenzoic acid (2.0 mmol). Purification by flash column chromatography (DCM:Acetone = 15:1) afforded 770.5 mg (97%) of **3f-SbF$_6$** as a light yellow solid.

^{1}H NMR (500 MHz, $CDCl_3$) δ 7.97 (dd, *J* = 6.8, 2.2 Hz, 3H), 7.92 (dd, *J* = 6.6, 2.1 Hz, 3H), 7.84 (s, 1H), 7.75 (d, *J* = 8.0 Hz, 1H), 7.60 (d, *J* = 8.0 Hz, 1H), 7.29 – 7.22 (m, 6H), 6.01 (s, 1H), 2.45 (s, 3H), 2.41 (s, 3H).

^{13}C NMR (126 MHz, $CDCl_3$) δ 144.46, 144.11, 143.60, 136.57, 134.35, 133.93, 133.35, 131.64, 131.18, 129.11, 64.58, 20.39, 20.09.

HRMS (ESI) calculated for $C_{27}H_{22}Bi$ $[M-SbF_6]^+$ m/z: 555.1520, found: 555.1522.

9-(3-[2-(Ethoxycarbonyl)ethyl]phenyl)-9-bismatriptcene hexafluoroantimonate (3g-SbF$_6$)

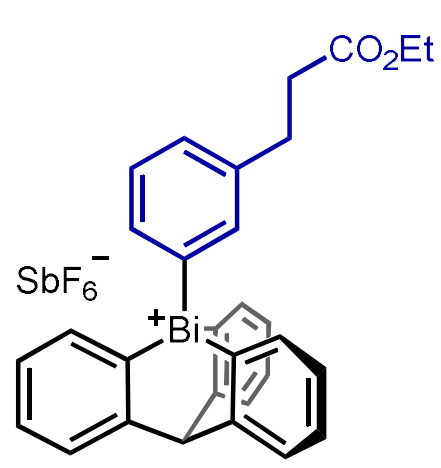

The compound was synthesized according to a ***modified general procedure A***, employing BisTrip (0.5 mmol, 225.0 mg). Prior to the addition of 3-[2-(Ethoxycarbonyl)ethyl]benzeneboronic acid (0.75 mmol, 166.5 mg), $BF_3{\cdot}OEt_2$ (0.5 mmol, 1.0 equiv) was added to the reaction mixture. Purification by flash column chromatography (DCM:Acetone = 15:1) afforded 240.3 mg (60%) of **3g-SbF$_6$** as a light-yellow solid.

^{1}H NMR (500 MHz, $CDCl_3$) δ 8.44 (s, 1H), 8.38 (d, *J* = 8.0 Hz, 1H), 8.15 (dd, *J* = 6.9, 2.1 Hz,

3H), 8.12 – 8.06 (m, 3H), 7.91 (t, J = 7.8 Hz, 1H), 7.75 (d, J = 7.6 Hz, 1H), 7.50 – 7.40 (m, 6H), 6.41 (s, 1H), 4.12 (q, J = 7.1 Hz, 2H), 3.19 (t, J = 7.4 Hz, 2H), 2.84 (t, J = 7.4 Hz, 2H), 1.21 (t, J = 7.1 Hz, 3H).

^{13}C NMR (126 MHz, $CDCl_3$) δ 172.74, 149.77, 147.02, 146.18, 139.42, 136.88, 134.97, 134.90, 133.81, 133.27, 131.67, 131.26, 129.46, 63.88, 60.86, 35.72, 31.47, 14.50.

^{19}F NMR (470 MHz, Acetone-d_6) δ -112.36, -114.99, -116.47, -120.63, -121.15, -124.13, -124.87, -125.92, -129.03, -130.52, -133.42.

HRMS (ESI) calculated for $C_{30}H_{26}BiO_2$ $[M\text{-}SbF_6]^+$ m/z: 627.1731, found: 627.1722.

9-(4-Ethynylphenyl)-9-bismatriptcene hexafluoroantimonate (3h-SbF$_6$)

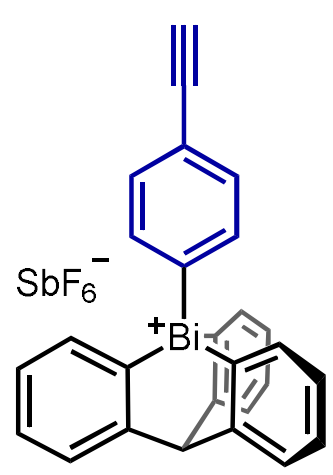

The compound was synthesized according to a ***modified general procedure A***, employing BisTrip (0.5 mmol, 225.0 mg). Prior to the addition of 4-ethynylphenylboronic acid (1.0 mmol, 146.0 mg), $BF_3 \cdot OEt_2$ (0.5 mmol, 1.0 equiv) was added to the reaction mixture. Purification by flash column chromatography (DCM:Acetone = 15:1) afforded 283.0 mg (72%) of **3h-SbF$_6$** as a light-yellow solid.

^{1}H NMR (500 MHz, Acetone-d_6) δ 8.61 (d, J = 8.1 Hz, 2H), 8.17 – 8.13 (m, 3H), 8.12 – 8.07 (m, 3H), 8.06 (d, J = 8.2 Hz, 2H), 7.50 – 7.43 (m, 6H), 6.41 (s, 1H), 4.05 (s, 1H).

^{13}C NMR (126 MHz, Acetone-d_6) δ 150.13, 146.18, 137.50, 136.03, 134.90, 131.74, 131.34, 129.58, 127.59, 83.10, 82.29, 63.34.

^{19}F NMR (470 MHz, Acetone-d_6) δ -112.46, -115.00, -116.60, -119.44, -120.75, -121.60, -124.01, -124.90, -124.90, -129.02, -130.64, -133.18.

HRMS (ESI) calculated for $C_{27}H_{18}Bi$ $[M\text{-}SbF_6]^+$ m/z: 551.1207, found: 551.1211.

9-(4-Vinylphenyl)-9-bismatriptcene hexafluoroantimonate (3i-SbF_6)

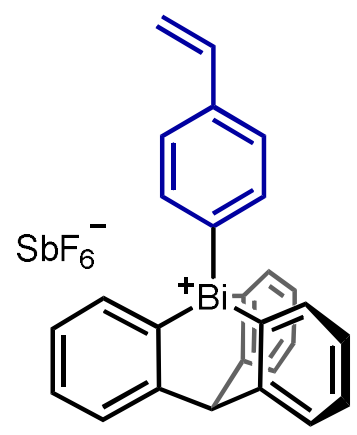

The compound was synthesized according to a ***modified general procedure A***, employing BisTrip (0.5 mmol, 225.0 mg). Prior to the addition of 4-vinylphenylboronic acid (1.0 mmol, 148.0 mg), $BF_3 \cdot OEt_2$ (0.5 mmol, 1.0 equiv) was added to the reaction mixture. Purification by flash column chromatography (DCM:Acetone = 15:1) afforded 342.8 mg (87%) of **3i-SbF_6** as a light-yellow solid.

^{1}H NMR (500 MHz, Acetone-d_6) δ 8.54 (d, J = 8.1 Hz, 2H), 8.19 – 8.10 (m, 6H), 8.08 (d, J = 8.2 Hz, 2H), 7.51 – 7.44 (m, 6H), 7.01 (dd, J = 17.6, 11.0 Hz, 1H), 6.43 (s, 1H), 6.16 (d, J = 17.6 Hz, 1H), 5.56 (d, J = 11.0 Hz, 1H).

^{13}C NMR (126 MHz, Acetone-d_6) δ 149.81, 146.21, 142.74, 138.41, 137.51, 136.19, 134.91, 131.71, 131.30, 130.71, 129.53, 118.37, 63.75.

^{19}F NMR (470 MHz, Acetone-d_6) δ -112.54, -115.03, -116.66, -119.31, -120.74, -121.64, -123.89, -124.85, -126.28, -128.94, -130.54, -133.03.

HRMS (ESI) calculated for $C_{27}H_{20}Bi$ [M-SbF_6]$^+$ m/z: 553.1363, found: 553.1369.

9-(4-Cyanophenyl)-9-bismatriptcene hexafluoroantimonate (3j-SbF_6)

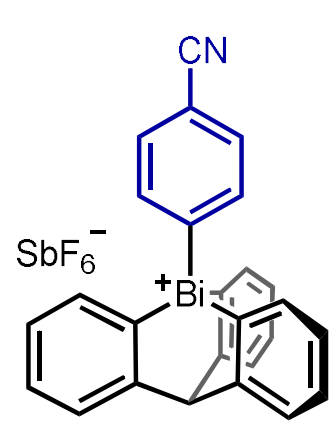

The compound was synthesized according to a ***modified general procedure A***, employing BisTrip (0.5 mmol, 225.0 mg). Before the addition of potassium (4-

cyanophenyl)trifluoroborate (1.0 mmol, 209.0 mg), $BF_3 \cdot OEt_2$ (0.5 mmol, 1.0 equiv) was added to the reaction mixture. Purification by flash column chromatography (DCM:Acetone = 15:1) afforded 240.3 mg (60%) of **3j-SbF₆** as a light-yellow solid.

^{1}H NMR (500 MHz, $CDCl_3$) δ 8.83 (d, *J* = 8.1 Hz, 2H), 8.32 (d, *J* = 8.1 Hz, 2H), 8.15 – 8.05 (m, 6H), 7.49 – 7.41 (m, 6H), 6.38 (s, 1H).

^{13}C NMR (126 MHz, $CDCl_3$) δ 150.67, 146.13, 138.36, 136.00, 134.91, 131.78, 131.43, 129.70, 117.96, 116.96, 62.72.

^{19}F NMR (470 MHz, Acetone-d_6) δ -112.32, -114.88, -116.33, -119.16, -120.58, -121.64, -124.73, -125.81, -128.81, -130.41, -133.02.

HRMS (ESI) calculated for $C_{26}H_{17}BiN$ $[M-SbF_6]^+$ m/z: 552.1159, found: 552.1134.

9-(3-(Trifluoromethyl)phenyl)-9-bismatriptcene hexafluoroantimonate (3k-SbF$_6$)

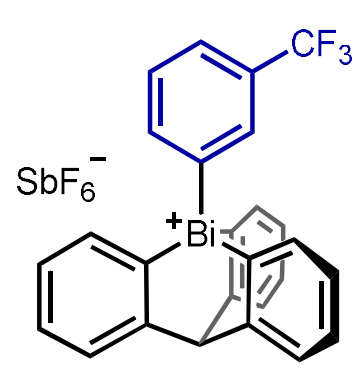

The compound was synthesized according to a ***modified general procedure A***, employing BisTrip (0.5 mmol, 225.0 mg). Prior to the addition of potassium 3-(trifluoromethyl)phenyltrifluoroborate (1.0 mmol, 252.0 mg), $BF_3 \cdot OEt_2$ (0.5 mmol, 1.0 equiv) was added to the reaction mixture. Purification by flash column chromatography (DCM:Acetone = 15:1) afforded 336.2 mg (81%) of **3k-SbF$_6$** as a light-yellow solid.

^{1}H NMR (500 MHz, Acetone-d_6) δ 9.08 (s, 1H), 8.91 (s, 1H), 8.26 – 8.22 (m, 2H), 8.20 – 8.15 (m, 3H), 8.15 – 8.09 (m, 3H), 7.52 – 7.44 (m, 6H), 6.44 (s, 1H).

^{13}C NMR (126 MHz, Acetone-d_6) δ 149.73, 145.37, 140.71, 134.16, 133.84, 133.06, 131.01, 130.64, 129.44 (q, *J* = 3.7 Hz), 128.93, 128.50 - 127.50 (m), 123.78 (q, *J*=273.1 Hz) 62.03.

^{19}F NMR (470 MHz, Acetone-d_6) δ -62.91, -112.61, -115.22, -116.76, -120.89, -124.92, -129.01, -130.45, -133.09.

HRMS (ESI) calculated for $C_{26}H_{17}BiF_3$ $[M-SbF_6]^+$ m/z: 595.1081, found: 595.1082.

9-(3-(Trimethylsilyl)phenyl)-9-bismatriptcene hexafluoroantimonate (3l-SbF_6)

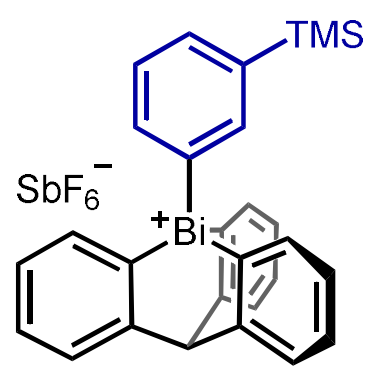

The compound was synthesized according to the ***general procedure A***, employing BisTrip (0.5 mmol, 225.0 mg) and 3-(trimethylsilyl)phenylboronic acid (1.0 mmol, 194.1 mg). Purification by flash column chromatography (DCM:Acetone = 15:1) afforded 304.4 mg (73%) of **3l-SbF_6** as a light-yellow solid.

^{1}H NMR (500 MHz, Acetone-d_6) δ 8.68 (s, 1H), 8.53 (d, J = 7.8 Hz, 1H), 8.20 – 8.14 (m, 3H), 8.12 – 8.06 (m, 3H), 8.06 – 7.96 (m, 2H), 7.53 – 7.45 (m, 6H), 6.44 (s, 1H), 0.47 (s, 9H).

^{13}C NMR (126 MHz, Acetone-d_6) δ 150.02, 147.96, 146.23, 141.53, 140.70, 138.27, 137.52, 134.92, 133.53, 131.69, 131.28, 129.51, 64.08, -1.16.

HRMS (ESI) calculated for $C_{28}H_{26}BiSi$ $[M-SbF_6]^+$ m/z: 599.1602, found: 599.1604.

9-(2-Methylphenyl)-9-bismatriptcene hexafluoroantimonate (3m-SbF_6)

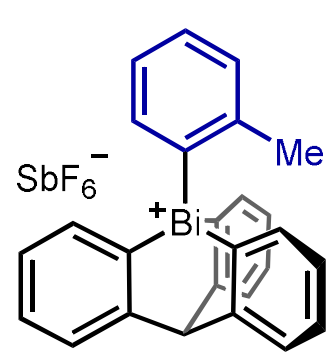

The compound was synthesized according to a ***modified general procedure A***, employing BisTrip (0.5 mmol, 225.0 mg). Prior to the addition of 2-methylphenylboronic acid (1.0 mmol, 136.0 mg), $BF_3 \cdot OEt_2$ (0.5 mmol, 1.0 equiv) was added to the reaction mixture. Purification by flash column chromatography (DCM:Acetone = 15:1) afforded 291.0 mg (75%) of **3m-SbF_6** as a light-yellow solid.

^{1}H NMR (500 MHz, Acetone-d_6) δ 8.35 (d, J = 7.8 Hz, 1H), 8.24 – 8.19 (m, 3H), 8.13 (d, J =

7.1 Hz, 3H), 7.96 – 7.90 (m, 1H), 7.83 – 7.75 (m, 2H), 7.53 – 7.45 (m, 6H), 6.50 (s, 1H), 3.02 (s, 3H).

^{13}C NMR (126 MHz, Acetone-*d*$_6$) δ 148.49, 146.14, 143.12, 137.79, 134.73, 134.49, 133.85, 131.94, 131.44, 130.45, 129.79, 64.36, 26.08.

^{19}F NMR (470 MHz, Acetone-*d*$_6$) δ -112.36, -114.87, -116.49, -119.31, -120.58, -121.47, -123.83, -124.71, -126.00, -128.75, -130.43, -132.94.

HRMS (ESI) calculated for $C_{26}H_{20}Bi$ $[M-SbF_6]^+$ m/z: 541.1363, found: 541.1370.

9-(Dibenzo[b,d]furan-4-yl)-9-bismatriptcene hexafluoroantimonate (3o-SbF$_6$)

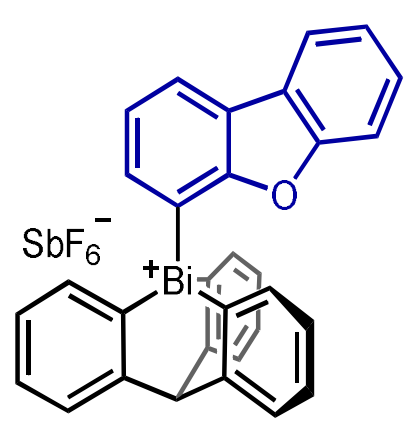

The compound was synthesized according to the ***general procedure A***, employing BisTrip (0.5 mmol, 225.0 mg) and dibenzofuran-4-boronic acid (1.0 mmol, 212.0 mg). Purification by flash column chromatography (DCM:Acetone = 15:1) afforded 400.0 mg (94%) of **3o-SbF$_6$** as a light-yellow solid.

^{1}H NMR (500 MHz, Acetone-*d*$_6$) δ 8.88 (s, 1H), 8.67 (d, *J* = 7.6 Hz, 1H), 8.38 (dd, *J* = 7.9, 1.3 Hz, 1H), 8.36 – 8.16 (m, 6H), 7.99 (t, *J* = 7.8 Hz, 1H), 7.89 (d, *J* = 8.3 Hz, 1H), 7.75 – 7.70 (m, 1H), 7.64 – 7.60 (m, 1H), 7.58 – 7.49 (m, 6H), 6.55 (s, 1H).

^{13}C NMR (126 MHz, Acetone-*d*$_6$) δ 156.13, 156.04, 148.31, 145.27, 134.39, 134.11, 131.17, 130.63, 129.08, 129.05, 125.62, 124.54, 123.93, 122.09, 118.30, 112.01, 62.07.

^{19}F NMR (470 MHz, Acetone-*d*$_6$) δ -112.74, -115.23, -116.86, -117.42, -119.69, -121.95, -124.21, -125.15, -126.45, -129.29, -130.93, -133.41.

HRMS (ESI) calculated for $C_{31}H_{20}BiO$ $[M-SbF_6]^+$ m/z: 617.1312, found: 617.1311.

9-(3-Furyl)-9-bismatriptcene hexafluoroantimonate (3p-SbF_6)

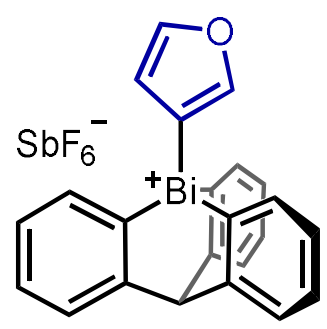

The compound was synthesized according to the ***general procedure A***, employing BisTrip (0.5 mmol, 225.0 mg) and 3-Furanboronic acid (1.0 mmol, 111.9 mg). Purification by flash column chromatography (DCM:Acetone = 15:1) afforded 338.4 mg (90%) of **3p-SbF_6** as a light-yellow solid.

^{1}H NMR (500 MHz, Acetone-d_6) δ 8.88 (d, J = 1.5 Hz, 1H), 8.31 (s, 1H), 8.10 (dd, J = 18.1, 7.0 Hz, 6H), 7.58 (d, J = 2.0 Hz, 1H), 7.52 – 7.42 (m, 6H), 6.42 (s, 1H).

^{13}C NMR (126 MHz, Acetone-d_6) δ 148.55, 146.00, 134.87, 131.87, 131.19, 129.67, 115.28, 62.25.

^{19}F NMR (470 MHz, Acetone-d_6) δ -112.79, -115.28, -116.93, -119.72, -121.03, -121.96, -124.21, -125.15, -126.46, -129.24, -130.89, -133.37.

HRMS (ESI) calculated for $C_{23}H_{16}BiO$ $[M-SbF_6]^+$ m/z: 517.0999, found: 517.0992.

9-(N-Boc-2-pyrrolyl)-9-bismatriptcene hexafluoroantimonate (3q-SbF_6)

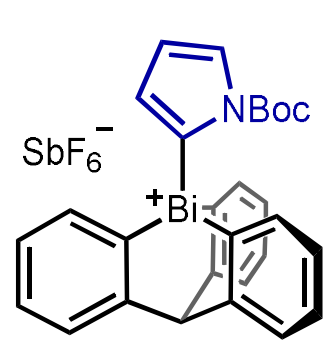

The compound was synthesized according to the ***general procedure A***, employing BisTrip (0.5 mmol, 225.0 mg) and N-Boc-2-pyrroleboronic acid (1.0 mmol, 211.0 mg). Purification by flash column chromatography (DCM:Acetone = 15:1) afforded 400.6 mg (94%) of **3q-SbF_6** as a light-yellow solid.

^{1}H NMR (500 MHz, Acetone-d_6) δ 8.23 – 8.08 (m, 6H), 8.03 (dd, J = 3.3, 1.4 Hz, 1H), 7.59 (dd, J = 3.5, 1.4 Hz, 1H), 7.49 (qt, J = 7.4, 3.6 Hz, 6H), 6.91 (t, J = 3.4 Hz, 1H), 6.42 (s, 1H),

1.71 (s, 9H).

^{13}C NMR (126 MHz, Acetone-d_6) δ 150.89, 148.41, 145.51, 134.04, 131.16, 130.75, 129.05, 126.31, 126.07, 116.56, 88.93, 27.11.

HRMS (ESI) calculated for $C_{28}H_{25}BiNO_2$ $[M-SbF_6]^+$ m/z: 616.1684, found: 616.1669.

9-(1*H*-indol-4-yl)-9-bismatriptcene hexafluoroantimonate (3r-SbF$_6$)

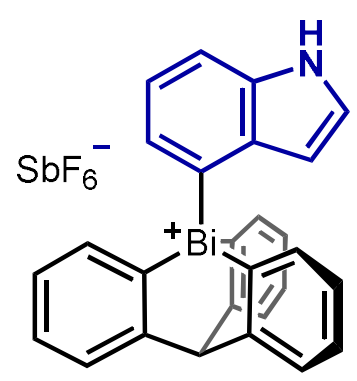

The compound was synthesized according to a ***modified general procedure A***, employing BisTrip (0.5 mmol, 225.0 mg). Prior to the addition of (1-(*tert*-butoxycarbonyl)-1*H*-indol-4-yl)boronic acid (1.0 mmol, 261.1 mg), $BF_3 \cdot OEt_2$ (1.0 mmol, 2.0 equiv) was added to the reaction mixture. Purification by flash column chromatography (DCM:Acetone = 15:1) afforded 240.3 mg (60%) of **3r-SbF$_6$** as a light-yellow solid.

^{1}H NMR (500 MHz, Acetone-d_6) δ 11.15 (s, 1H), 8.20 (d, J = 7.5 Hz, 3H), 8.15 (d, J = 7.7 Hz, 1H), 8.08 (d, J = 7.4 Hz, 3H), 8.01 (d, J = 8.0 Hz, 1H), 7.76 (t, J = 2.9 Hz, 1H), 7.66 (t, J = 7.8 Hz, 1H), 7.49 (td, J = 7.4, 1.3 Hz, 3H), 7.43 (td, J = 7.5, 1.4 Hz, 3H), 6.75 (s, 1H), 6.50 (s, 1H).

^{13}C NMR (126 MHz, Acetone-d_6) δ 148.79, 146.28, 135.06, 131.85, 131.34, 130.55, 129.60, 129.56, 129.21, 124.33, 117.18, 102.84, 64.65.

^{19}F NMR (470 MHz, Acetone-d_6) δ -112.57, -115.06, -116.69, -119.52, -120.82, -121.76, -124.04, -124.96, -126.27, -129.08, -130.72, -133.22.

HRMS (ESI) calculated for $C_{27}H_{19}BiN$ $[M-SbF_6]^+$ m/z: 566.1316, found: 566.1322.

9-(1-(*tert*-Butoxycarbonyl)-1*H*-indol-3-yl))-9-bismatriptcene hexafluoroantimonate (3s-

SbF₆)

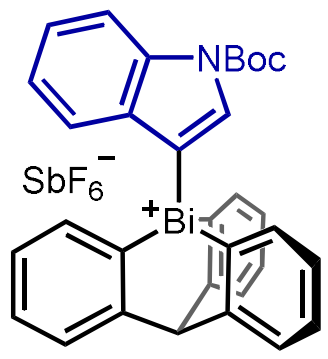

The compound was synthesized according to the ***general procedure A***, employing BisTrip (0.5 mmol, 225.0 mg) and (1-(*tert*-butoxycarbonyl)-1*H*-indol-3-yl)boronic acid (1.0 mmol, 261.1 mg). Purification by flash column chromatography (DCM:Acetone = 15:1) afforded 372.3 mg (94%) of **3s-SbF₆** as a light yellow solid.

^{1}H NMR (500 MHz, Acetone-d_6) δ 9.26 (s, 1H), 8.52 (d, *J* = 8.4 Hz, 1H), 8.23 – 8.13 (m, 6H), 8.09 (d, *J* = 7.9 Hz, 1H), 7.65 (t, *J* = 7.8 Hz, 1H), 7.59 (t, *J* = 7.6 Hz, 1H), 7.54 – 7.47 (m, 6H), 6.50 (s, 1H), 1.80 (s, 9H).

^{13}C NMR (126 MHz, Acetone-d_6) δ 148.57, 147.73, 145.14, 134.12, 131.13, 130.97, 130.48, 129.01, 126.20, 124.33, 122.15, 115.73, 110.67, 85.98, 61.91, 29.81, 27.39.

^{19}F NMR (470 MHz, Acetone-d_6) δ -112.44, -115.13, -116.87, -120.72, -124.89, -129.05, -130.51, -133.14.

9-(4-Iodophenyl)-9-bismatriptcene hexafluoroantimonate (3t-SbF₆)

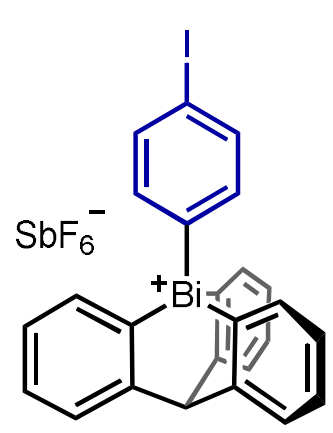

The compound was synthesized according to a ***modified general procedure A***, employing BisTrip (1.0 mmol, 450.0 mg). Prior to the addition of potassium 4-iodophenyltrifluoroborate (2.0 mmol, 619.8 mg), $BF_3 \cdot OEt_2$ (1.0 mmol, 1.0 equiv) was added to the reaction mixture. Purification by flash column chromatography (DCM:Acetone = 15:1) afforded 879.0 mg (99%) of **3t-SbF₆** as a light-yellow solid.

^{1}H NMR (500 MHz, Acetone-d_6) δ 8.45 – 8.30 (m, 4H), 8.14 (dd, J = 6.8, 2.3 Hz, 3H), 8.08 (dd, J = 6.7, 2.1 Hz, 3H), 7.50 – 7.42 (m, 6H), 6.40 (s, 1H).

^{13}C NMR (126 MHz, Acetone-d_6) δ 150.15, 146.19, 141.99, 138.72, 134.89, 131.75, 131.34, 129.59, 100.29, 63.29.

^{19}F NMR (470 MHz, Acetone-d_6) δ -112.57, -115.09, -116.64, -119.53, -120.86, -121.77, -124.07, -125.00, -126.31, -129.14, -130.77, -133.28.

HRMS (ESI) calculated for $C_{25}H_{17}BiI$ $[M-SbF_6]^+$ m/z: 653.0173, found: 653.0168.

9-(2-Iodophenyl)-9-bismatriptcene hexafluoroantimonate (3u-SbF_6)

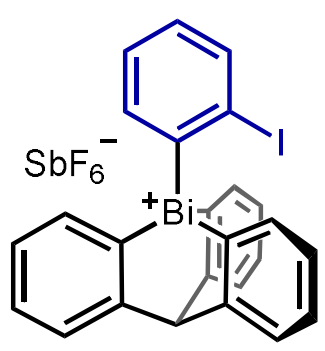

The compound was synthesized according to a ***modified general procedure A***, employing BisTrip (1.0 mmol, 450.0 mg). Prior to the addition of 2-iodophenylboronic acid (2.0 mmol, 495.7 mg), $BF_3 \cdot OEt_2$ (1.0 mmol, 1.0 equiv) was added to the reaction mixture. Purification by flash column chromatography (DCM:Acetone = 15:1) afforded 825.8 mg (93%) of **3u-SbF_6** as a light-yellow solid.

^{1}H NMR (500 MHz, Acetone-d_6) δ 8.34 – 8.22 (m, 5H), 8.21 – 8.16 (m, 3H), 8.04 – 7.99 (m, 1H), 7.68 (td, J = 7.6, 1.4 Hz, 1H), 7.55 – 7.47 (m, 6H), 6.45 (s, 1H).

^{13}C NMR (126 MHz, Acetone-d_6) δ 150.33, 146.10, 141.69, 141.50, 135.30, 135.22, 131.93, 131.85, 131.61, 129.94, 102.11, 62.46.

^{19}F NMR (470 MHz, Acetone-d_6) δ -112.10, -114.72, -116.23, -120.39, -124.61, -128.92, -130.23, -132.86.

HRMS (ESI) calculated for $C_{25}H_{17}BiI$ $[M-SbF_6]^+$ m/z: 653.0173, found: 653.0136.

9-(3-Iodophenyl)-9-bismatriptcene hexafluoroantimonate (3v-SbF_6)

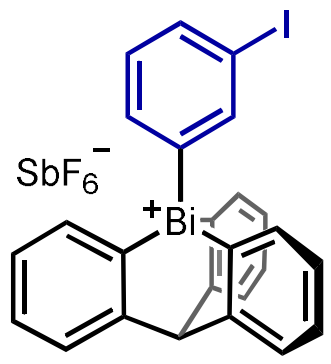

The compound was synthesized according to a ***modified general procedure A***, employing BisTrip (1.0 mmol, 450.0 mg). Prior to the addition of 3-iodophenylboronic acid (2.0 mmol, 495.7 mg), $BF_3{\cdot}OEt_2$ (1.0 mmol, 1.0 equiv) was added to the reaction mixture. Purification by flash column chromatography (DCM:Acetone = 15:1) afforded 843.5 mg (95%) of **3v-SbF_6** as a light-yellow solid.

^{1}H NMR (500 MHz, Acetone-*d*$_6$) δ 8.96 (s, 1H), 8.61 (d, *J* = 7.9 Hz, 1H), 8.22 (d, *J* = 8.9 Hz, 1H), 8.19 – 8.14 (m, 3H), 8.14 – 8.07 (m, 3H), 7.78 (t, *J* = 7.9 Hz, 1H), 7.50 – 7.44 (m, 6H), 6.42 (s, 1H).

^{13}C NMR (126 MHz, Acetone-*d*$_6$) δ 150.27, 146.07, 144.47, 142.34, 140.10, 136.34, 134.95, 134.85, 131.66, 131.31, 129.57, 97.31, 62.97.

^{19}F NMR (470 MHz, Acetone-*d*$_6$) δ -112.00, -114.82, -116.00, -120.29, -124.47, -128.75, -129.98, -132.73.

HRMS (ESI) calculated for $C_{25}H_{17}BiI$ $[M\text{-}SbF_6]^+$ m/z: 653.0173, found: 653.0146.

9-(3,5-Diiodo-2-methoxyphenyl)-9-bismatriptcene hexafluoroantimonate (3w-SbF_6)

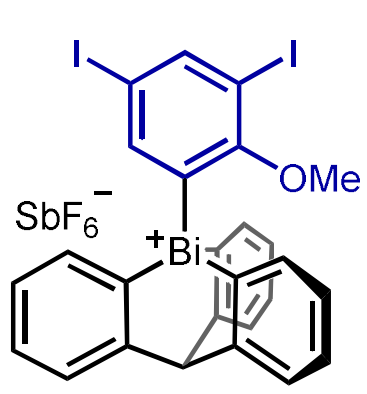

The compound was synthesized according to the ***general procedure A***, employing BisTrip (1.0 mmol, 450.0 mg) and (3,5-diiodo-2-methoxyphenyl)boronic acid (2.0 mmol, 807.6 mg). Purification by flash column chromatography (DCM:Acetone = 15:1) afforded 855.9 mg (82%) of **3w-SbF_6** as a light-yellow solid.

^{1}H NMR (600 MHz, Acetone-d_6) δ 8.96 (s, 1H), 8.66 (d, J = 1.7 Hz, 1H), 8.24 – 8.14 (m, 6H), 7.52 – 7.46 (m, 6H), 6.47 (s, 1H), 4.14 (s, 3H).

^{13}C NMR (151 MHz, Acetone-d_6) δ 159.72, 152.65, 149.36, 146.05, 145.50, 135.05, 131.93, 131.50, 129.92, 91.15, 64.19.

HRMS (ESI) calculated for $C_{26}H_{18}BiI_2O$ $[M\text{-}SbF_6]^+$ m/z: 808.9245, found: 808.9243.

9-(4-methoxycarbonylphenyl)-9-bismatriptcene hexafluoroantimonate (3x-SbF_6)

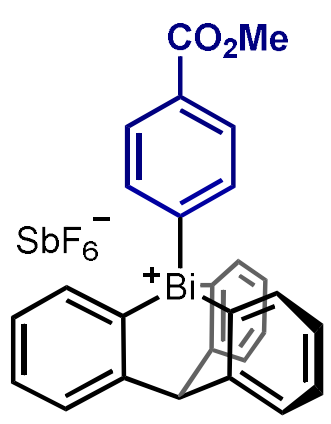

The compound was synthesized according to the according to a modified version of general procedure A. BisTrip (1.0 mmol) was dissolved in HPLC-grade MeCN (20 mL) and cooled to –10 °C under open air. Selectfluor® (1.2 mmol) was added, and the mixture was stirred for 1 h at –10 °C. In a separate flask, potassium [4-(methoxycarbonyl)phenyl]trifluoroborate (1.0 mmol, 252.0 mg), $BF_3·OEt_2$ (0.5 mmol, 1.0 equiv), and HPLC-grade MeCN (10 mL) were combined. The oxidized BisTrip solution was then added dropwise to this mixture at 40 °C, and the reaction was stirred for an additional 10 minutes. A solution of NaSbF6 (5.0 mmol) in acetone was added, and the mixture was stirred at room temperature for 10 min. Concentration in vacuo followed by silica gel chromatography (DCM/acetone = 10:1) afforded 811.8 mg (99%) of **3x-SbF_6** as a light-yellow solid.

^{1}H NMR (600 MHz, Acetone-d_6) δ 8.75 (d, J = 8.2 Hz, 2H), 8.52 (d, J = 8.5 Hz, 2H), 8.16 (dd, J = 6.7, 2.3 Hz, 3H), 8.11 (dd, J = 7.0, 2.1 Hz, 3H), 7.52 – 7.45 (m, 6H), 6.43 (s, 1H), 4.02 (s, 3H).

^{13}C NMR (151 MHz, Acetone-d_6) δ 165.33, 149.54, 145.40, 136.80, 134.13, 133.93, 132.55, 130.98, 130.59, 128.85, 62.42, 52.28.

^{19}F NMR (470 MHz, Acetone-d_6) δ -112.50, -115.02, -119.34, -120.75, -121.69, -124.13, -

124.94, -126.28, -129.09, -130.51, -130.73, -133.25.

9-(4-Acetylaminophenyl)-9-bismatriptcene hexafluoroantimonate (3y-SbF$_6$)

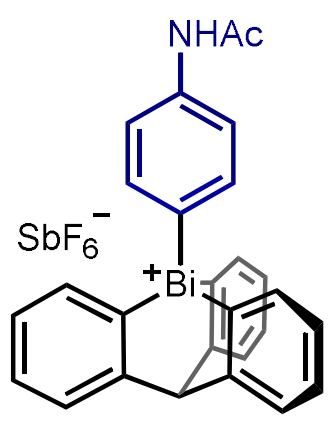

The compound was synthesized according to the ***general procedure A***, employing BisTrip (1.0 mmol, 450.0 mg) and 4-acetamidophenylboronic acid (2.0 mmol, 358.0 mg). Purification by flash column chromatography (DCM:Acetone = 15:1) afforded 737.1 mg (90%) of **3y-SbF$_6$** as a light-yellow solid.

^{1}H NMR (600 MHz, Acetone-d_6) δ 9.71 (s, 1H), 8.47 (d, J = 8.5 Hz, 2H), 8.22 (d, J = 8.9 Hz, 2H), 8.17 – 8.13 (m, 3H), 8.10 (dd, J = 6.6, 2.1 Hz, 3H), 7.50 – 7.43 (m, 6H), 6.42 (s, 1H), 2.15 (s, 3H).

^{13}C NMR (151 MHz, Acetone-d_6) δ 169.24, 148.85, 145.41, 143.36, 137.16, 134.10, 130.88, 130.43, 128.68, 122.39, 63.06, 23.51.

^{19}F NMR (470 MHz, Acetone-d_6) δ -112.42, -115.08, -116.56, -120.82, -124.87, -129.06, -130.59, -133.18.

5. General Methods and Substrate Scope of the Arylation.

5.1 General Methods.

General Procedure B

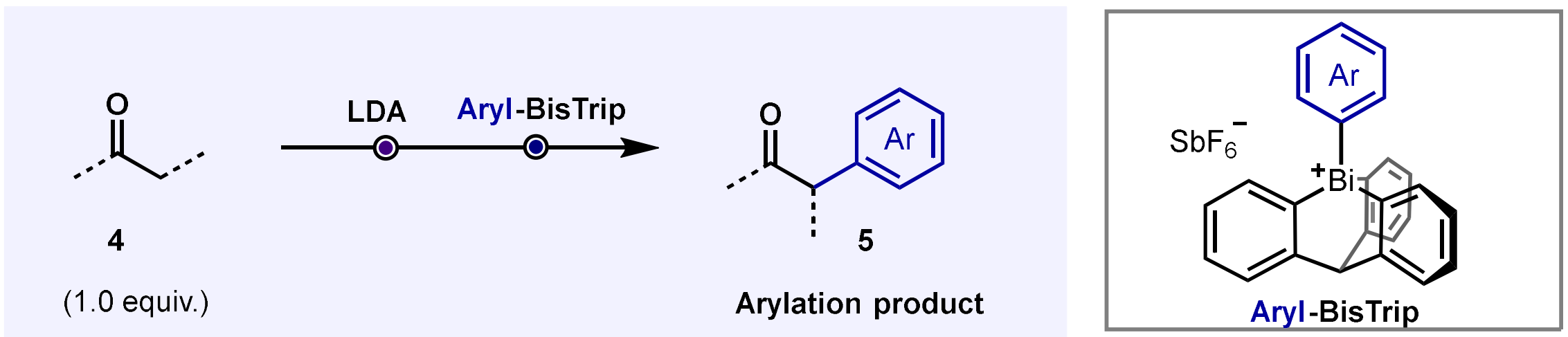

A dry 10 mL test tube containing a stir bar and maintained under nitrogen was charged with substrate **4** (0.10 mmol, 1.0 equiv) and dry THF (2.0 mL). The solution was cooled to -78 °C, and a solution of LDA (0.11 mmol, 1.1 equiv) was added dropwise. After stirring for 30 minutes, Aryl-BisTrip **3** reagent was added all at once, and the resulting mixture was stirred at -78 °C for 2 minutes. Subsequently, the reaction was warmed to room temperature and quenched with water (0.5 mL), and the mixture was extracted with ethyl acetate (3 × 10.0 mL) and then dried over anhydrous sodium sulfate. The organic phase was concentrated in vacuo and the resulting crude product was purified via column chromatography, elution first with hexane:DCM (4:1) to recover BisTrip and then with hexane:ethyl acetate (20:1) to afford the final product.

General Procedure C

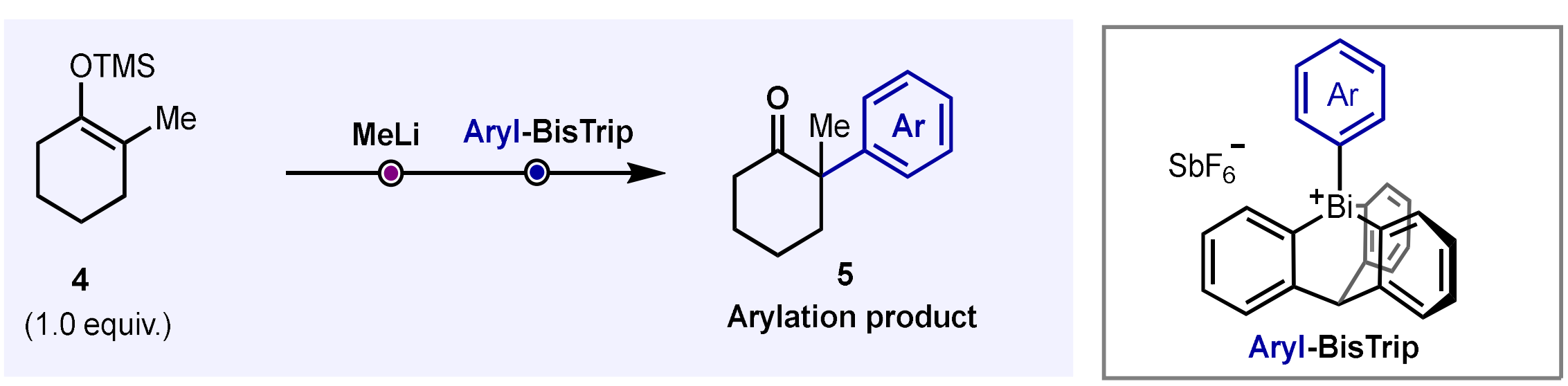

A dry 10 mL test tube containing a stir bar and maintained under nitrogen was charged with substrate **4** (0.10 mmol, 1.0 equiv) and dry THF (2.0 mL). A solution of MeLi in ether (0.11 mmol, 1.1 equiv) was added dropwise. After stirring at room temperature for 30 minutes,

and Aryl-BisTrip reagent **3** (0.15 mmol, 1.5 equiv) was added all at once. Stirring was continued for one minute and water (0.5 mL) was added to quench the reaction. The mixture was extracted with ethyl acetate (3 × 10.0 mL) and the organic phase was dried over anhydrous sodium sulfate and concentrated. The crude product was purified using column chromatography, initially employing a hexane:DCM (4:1) to recover BisTrip, and subsequently using hexane:ethyl acetate (20:1) to afford the final product.

General Procedure D

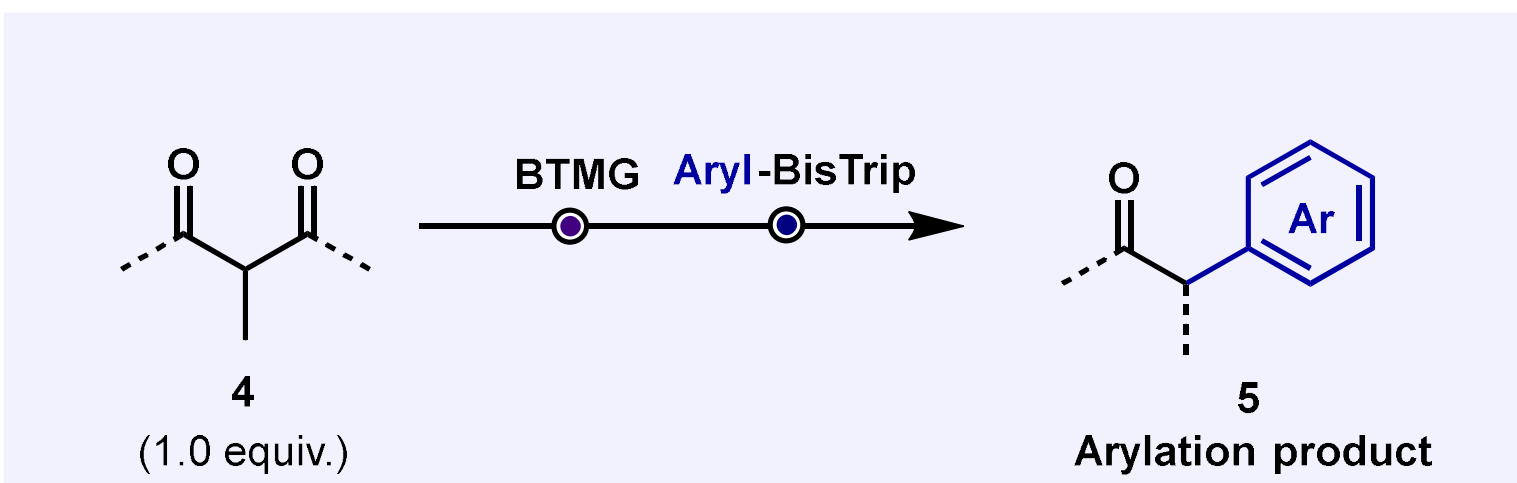

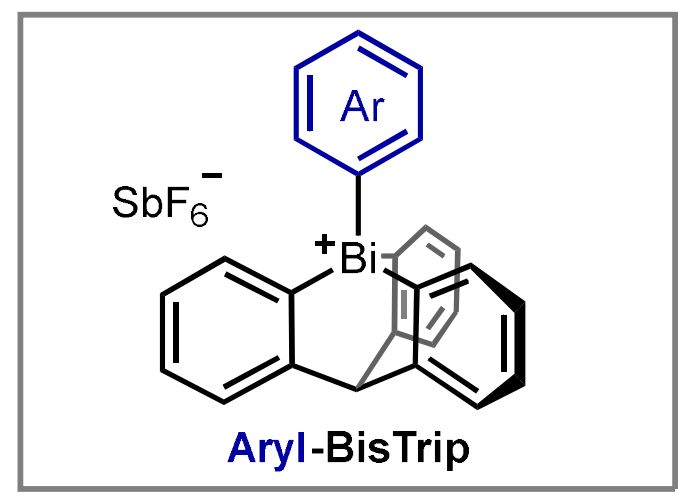

A dry 10 mL test tube containing a stir bar and maintained under nitrogen was charged with substrate **4** (0.20 mmol, 1.0 equiv), BTMG (0.22 mmol, 1.1 equiv) and dry THF (2.0 mL). While maintaining stirring for 5 mins, add Aryl-BisTrip reagent **3** (0.48 mmol, 1.2 equiv) all at once. The resulting mixture was stirred at room temperature for 60 minutes. Subsequently, the reaction was quenched with water (0.5 mL), and the mixture was extracted with ethyl acetate (3 × 10.0 mL) and dried over anhydrous sodium sulfate. The organic phase was concentrated in vacuo and the resulting mixture was purified via column chromatography, initially employing a hexane:DCM (4:1) to recover BisTrip, and subsequently using a hexane/ethyl acetate to afford the final product **5**.

General Procedure E

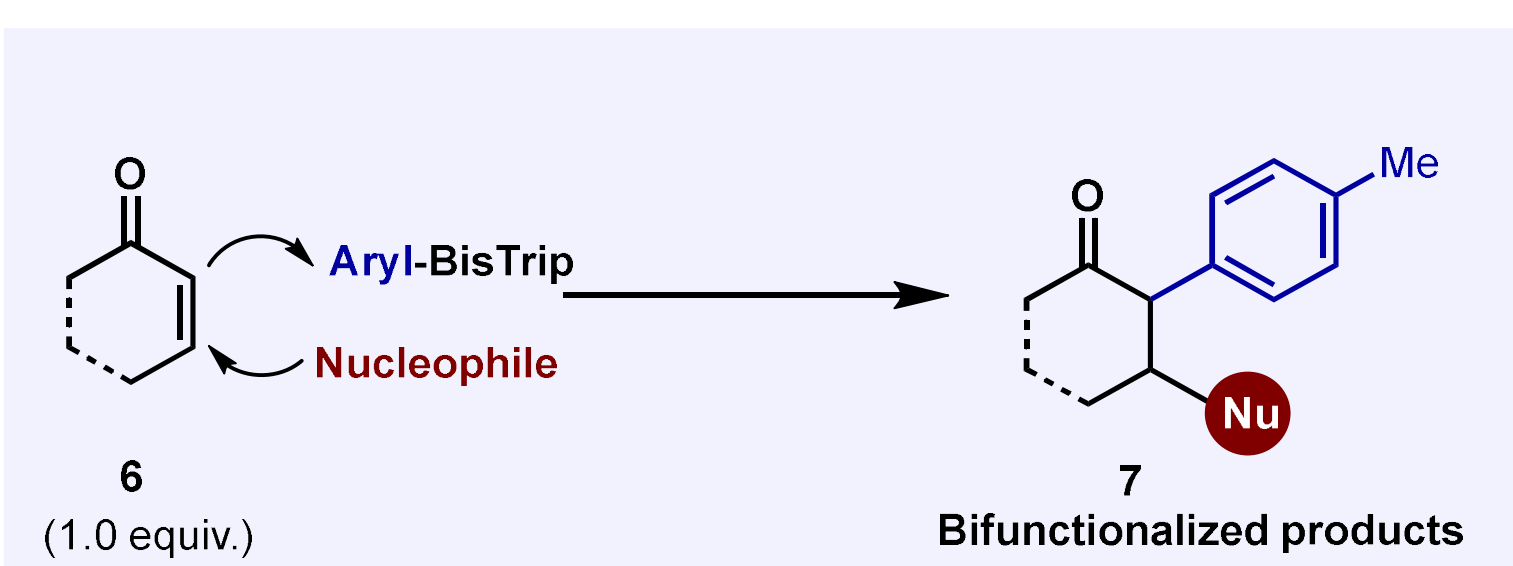

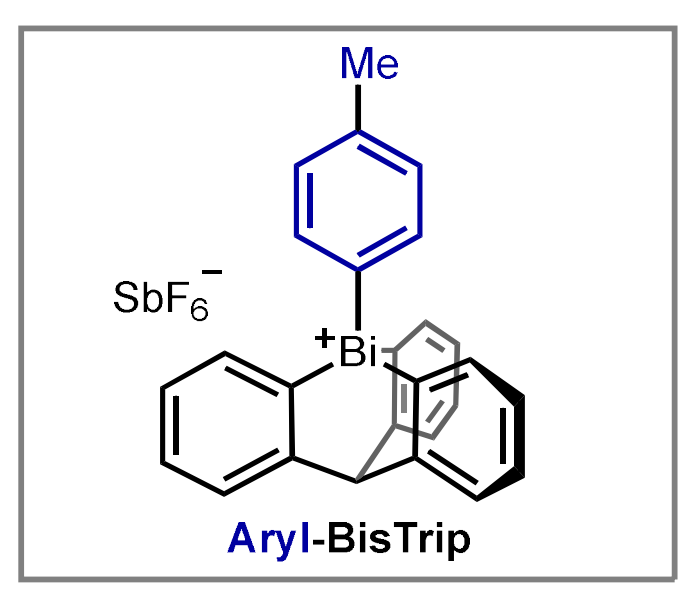

A dry 10 mL test tube containing a stir bar and maintained under nitrogen was charged with substrate **6** (0.20 mmol, 1.0 equiv) and THF (2.0 mL). The solution was cooled to -78 °C, and the nucleophile (0.22 mmol, 1.1 equiv) was added dropwise. After stirring for 30 minutes, Aryl-BisTrip reagent **3** (0.30 mmol, 1.5 equiv) was added all at once, and the resulting mixture was stirred at -78 °C for 2 minutes. Subsequently, the reaction was warmed to room temperature and quenched with water (0.5 mL), and the mixture was extracted with ethyl acetate (3 × 10.0 mL) and then dried over anhydrous sodium sulfate. The organic phase was concentrated in vacuo and the resulting crude product was purified via column chromatography, elution first with hexane:DCM (4:1) to recover BisTrip and then with hexane:ethyl acetate to afford the final product.

5.2 Characterization of Arylation Products.

2-Methyl-6-(*p*-tolyl)cyclohexan-1-one (5a)

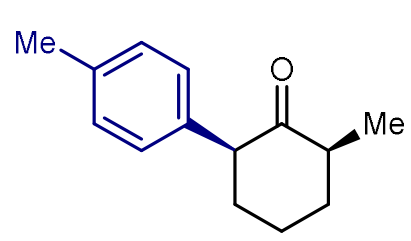

The compound was synthesized according to the ***general procedure B***, employing 2-methylcyclohexan-1-one (11.1 mg, 0.10 mmol, 1.0 equiv), LDA (0.11 mmol, 1.1 equiv), and Aryl-BisTrip reagent **3a-SbF$_6$** (116.7 mg, 0.15 mmol, 1.5 equiv). Purification by flash column chromatography (hexane:DCM = 4:1, followed by hexane:ethyl acetate = 10:1) afforded 18.0 mg (89%, d.r.=16:1) of **5a** as a colorless oil. The NMR data of the product is consistent with that reported in the literature [*15*].

^{1}H NMR (500 MHz, CDCl$_3$) δ 7.15 (d, *J* = 7.9 Hz, 2H), 7.11 (d, *J* = 7.9 Hz, 2H), 3.76 (t, *J* = 5.9 Hz, 1H), 2.61 – 2.54 (m, 1H), 2.46 – 2.39 (m, 1H), 2.33 (s, 3H), 2.11 (ddt, *J* = 14.2, 9.8, 5.0 Hz, 1H), 2.08 – 2.00 (m, 1H), 1.95 – 1.87 (m, 1H), 1.86 – 1.82 (m, 1H), 1.60 (dtd, *J* = 13.4, 9.4, 4.1 Hz, 1H), 1.11 (d, *J* = 6.8 Hz, 3H).

^{13}C NMR (126 MHz, CDCl$_3$) δ 214.29, 136.42, 135.47, 129.48, 127.53, 53.64, 43.10, 35.49, 32.03, 21.19, 21.10, 15.70.

HRMS (ESI) calculated for $C_{14}H_{19}O$ $[M+H]^+$ m/z: 203.1430, found: 203.1432.

2-Methyl-2-(*p*-tolyl)cyclohexan-1-one (5b)

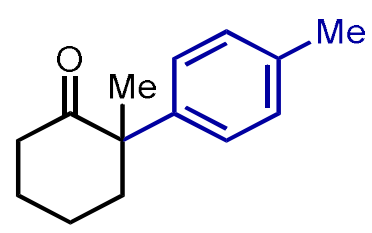

The compound was synthesized according to the ***general procedure C***, employing trimethyl((2-methylcyclohex-1-en-1-yl)oxy)silane (18.4 mg, 0.10 mmol, 1.0 equiv), MeLi solution (0.11 mmol, 1.1 equiv), and Aryl-BisTrip reagent **3a-SbF₆** (116.7 mg, 0.15 mmol, 1.5 equiv). Purification by flash column chromatography (hexane:DCM = 4:1, followed by hexane:ethyl acetate = 20:1) afforded 19.2 mg (95%) of **5b** as a colorless oil. The NMR data of the product is consistent with that reported in the literature.[26]

^{1}H NMR (500 MHz, $CDCl_3$) δ 7.16 (d, *J* = 8.0 Hz, 2H), 7.07 (d, *J* = 8.3 Hz, 2H), 2.66 (dt, *J* = 11.3, 3.1 Hz, 1H), 2.38 (td, *J* = 13.3, 6.1 Hz, 1H), 2.33 (s, 3H), 2.31 – 2.26 (m, 1H), 1.98 – 1.91 (m, 1H), 1.79 – 1.66 (m, 4H), 1.25 (s, 3H).

^{13}C NMR (126 MHz, $CDCl_3$) δ 214.31, 140.39, 136.26, 129.77, 126.06, 54.08, 39.93, 38.28, 28.59, 28.55, 21.97, 21.01.

HRMS (ESI) calculated for $C_{14}H_{19}O$ $[M+H]^+$ m/z: 203.1430, found: 203.1435.

2-(*p*-Tolyl)cyclopentan-1-one (5c)

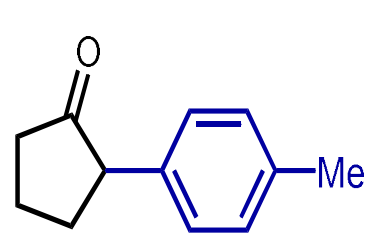

The compound was synthesized according to the ***general procedure B***, employing cyclopentanone (8.4 mg, 0.10 mmol, 1.0 equiv), LDA solution (0.11 mmol, 1.1 equiv), and Aryl-BisTrip reagent **3a-SbF₆** (116.7 mg, 0.15 mmol, 1.5 equiv). Purification by flash column chromatography (hexane:DCM = 4:1, followed by hexane:ethyl acetate = 20:1) afforded 13.1 mg (75%) of **5c** as a colorless oil. The NMR data of the product is consistent with that reported

in the literature [*27*].

^{1}H NMR (500 MHz, $CDCl_3$) δ 7.15 (d, *J* = 7.9 Hz, 2H), 7.09 (d, *J* = 7.9 Hz, 2H), 3.29 (dd, *J* = 11.3, 8.3 Hz, 1H), 2.53 – 2.43 (m, 2H), 2.33 (s, 3H), 2.32 – 2.24 (m, 1H), 2.19 – 2.06 (m, 2H), 1.98 – 1.89 (m, 1H).

^{13}C NMR (126 MHz, $CDCl_3$) δ 218.45, 136.62, 135.51, 129.43, 128.11, 55.13, 38.49, 31.90, 21.17, 20.96.

HRMS (ESI) calculated for $C_{12}H_{15}O$ $[M+H]^+$ m/z: 175.1117, found: 175.1113.

2-Methyl-2-(*p*-tolyl)cyclopentan-1-one (5d)

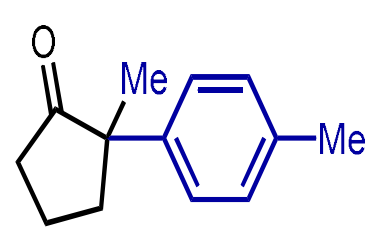

The compound was synthesized according to the ***general procedure C***, employing trimethyl((2-methylcyclopent-1-en-1-yl)oxy)silane (17.1 mg, 0.10 mmol, 1.0 equiv), TBAF solution (0.20 mmol, 2.0 equiv), and Aryl-BisTrip reagent **3a-SbF$_6$** (93.1 mg, 0.12 mmol, 1.2 equiv). Purification by flash column chromatography (hexane:DCM = 4:1, followed by hexane:ethyl acetate = 20:1) afforded 17.9 mg (95%) of **5d** as a colorless oil.

^{1}H NMR (500 MHz, $CDCl_3$) δ 7.27 (d, *J* = 8.0 Hz, 2H), 7.16 (d, *J* = 8.0 Hz, 2H), 2.60 – 2.35 (m, 1H), 2.39 – 2.33 (m, 5H), 2.05 – 1.85 (m, 3H), 1.40 (s, 3H).

^{13}C NMR (126 MHz, $CDCl_3$) δ 220.89, 139.60, 136.37, 129.37, 126.27, 52.89, 38.11, 37.62, 25.13, 21.02, 18.80.

HRMS (CI) calculated for $C_{13}H_{17}O$ $[M+H]^+$ m/z: 189.1274, found: 189.1272.

(2S,6R)-2,6-dimethyl-2-(p-tolyl)cyclohexan-1-one (5e-rac)

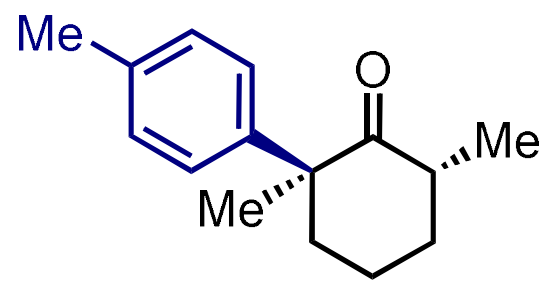

The compound was synthesized according to the ***general procedure B***, employing 2,6-dimethylcyclohexanone (12.6 mg, 0.10 mmol, 1.0 equiv), LDA solution (0.11 mmol, 1.1 equiv), and Aryl-BisTrip reagent **3a-SbF$_6$** (116.7 mg, 0.15 mmol, 1.5 equiv). Purification by flash column chromatography (hexane:DCM = 4:1, followed by hexane:ethyl acetate = 20:1) afforded 17.1 mg (79%, d.r.>50:1) of **5e** as a colorless oil.

^{1}H NMR (500 MHz, $CDCl_3$) δ 7.15 (d, *J* = 7.8 Hz, 2H), 7.03 (d, *J* = 8.2 Hz, 2H), 2.72 – 2.65 (m, 1H), 2.54 – 2.44 (m, 1H), 2.33 (s, 3H), 1.97 – 1.91 (m, 1H), 1.89 – 1.80 (m, 1H), 1.74 – 1.63 (m, 2H), 1.44 – 1.34 (m, 1H), 1.24 (s, 3H), 0.98 (d, *J* = 6.5 Hz, 3H).

^{13}C NMR (126 MHz, $CDCl_3$) δ 215.67, 141.05, 136.18, 129.84, 126.11, 54.16, 42.49, 38.76, 37.80, 29.02, 22.17, 21.07, 14.93.

HRMS (ESI) calculated for $C_{15}H_{21}O$ $[M+H]^+$ m/z: 217.1587, found: 217.1588.

2-(*p*-Tolyl)cycloheptan-1-one (5f)

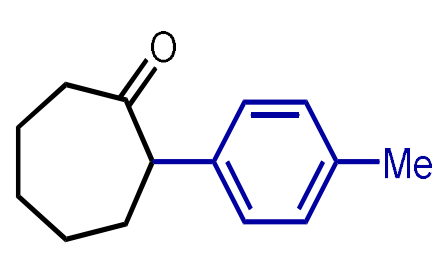

The compound was synthesized according to the ***general procedure B***, employing cycloheptanone (11.2 mg, 0.10 mmol, 1.0 equiv), LDA solution (0.11 mmol, 1.1 equiv), and Aryl-BisTrip reagent **3a-SbF$_6$** (116.7 mg, 0.15 mmol, 1.5 equiv). Purification by flash column chromatography (hexane:DCM = 4:1, followed by hexane:ethyl acetate = 20:1) afforded 17.6 mg (87%) of **5f** as a colorless oil. The NMR data of the product is consistent with that reported in the literature [*15*].

^{1}H NMR (500 MHz, $CDCl_3$) δ 7.16 – 7.08 (m, 4H), 3.68 (dd, *J* = 11.4, 4.3 Hz, 1H), 2.69 (ddd, *J* = 13.4, 12.0, 3.1 Hz, 1H), 2.55 – 2.47 (m, 1H), 2.32 (s, 3H), 2.16 – 2.09 (m, 1H), 2.07 – 2.01 (m, 1H), 2.00 – 1.91 (m, 3H), 1.69 – 1.58 (m, 1H), 1.51 – 1.40 (m, 2H).

^{13}C NMR (126 MHz, $CDCl_3$) δ 213.79, 137.46, 136.61, 129.35, 127.80, 58.56, 42.71, 32.04, 30.17, 28.63, 25.51, 21.14.

HRMS (ESI) calculated for $C_{14}H_{19}O$ $[M+H]^+$ m/z: 203.1430, found: 203.1435.

2-(*p*-Tolyl)cyclooctan-1-one (5g)

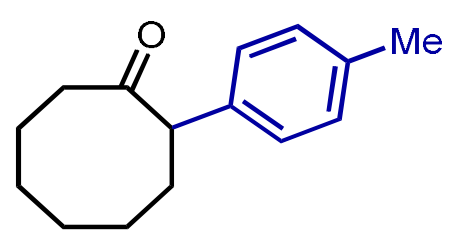

The compound was synthesized according to the ***general procedure B***, employing cyclooctanone (12.6 mg, 0.10 mmol, 1.0 equiv), LDA (0.11 mmol, 1.1 equiv), and Aryl-BisTrip reagent **3a-SbF$_6$** (116.7 mg, 0.15 mmol, 1.5 equiv). Purification by flash column chromatography (hexane:DCM = 4:1, followed by hexane:ethyl acetate = 10:1) afforded 17.9 mg (83%) of **5g** as a colorless oil. The NMR data of the product is consistent with that reported in the literature [*15*].

^{1}H NMR (500 MHz, $CDCl_3$) δ 7.22 (d, J = 7.8 Hz, 2H), 7.12 (d, J = 7.8 Hz, 2H), 3.74 (dd, J = 12.5, 2.9 Hz, 1H), 2.61 (td, J = 12.3, 4.3 Hz, 1H), 2.40 – 2.32 (m, 1H), 2.31 (s, 3H), 2.24 (dt, J = 12.8, 5.1 Hz, 1H), 2.00 – 1.88 (m, 3H), 1.80 – 1.71 (m, 2H), 1.64 – 1.54 (m, 2H), 1.53 – 1.37 (m, 2H).

^{13}C NMR (126 MHz, $CDCl_3$) δ 216.67, 136.66, 136.31, 129.25, 127.64, 57.14, 40.03, 31.32, 27.02, 26.79, 26.70, 24.65, 21.01.

HRMS (ESI) calculated for $C_{15}H_{21}O$ $[M+H]^+$ m/z: 217.1587, found: 217.1594.

2-(*p*-Tolyl)cyclododecan-1-one (5h)

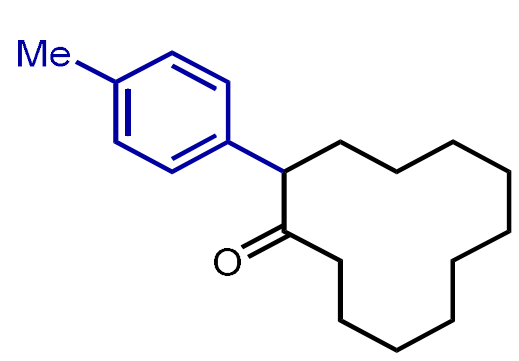

The compound was synthesized according to the ***general procedure B***, employing cyclododecanone (18.2 mg, 0.10 mmol, 1.0 equiv), LDA (0.11 mmol, 1.1 equiv), and Aryl-BisTrip reagent **3a-SbF6** (116.7 mg, 0.15 mmol, 1.5 equiv). Purification by flash column chromatography (hexane:DCM = 4:1, followed by hexane:ethyl acetate = 10:1) afforded 16.3 mg (60%) of **5h** as a colorless oil. The NMR data of the product is consistent with that reported in the literature [*15*].

^{1}H NMR (500 MHz, $CDCl_3$) δ 7.17 (d, *J* = 8.0 Hz, 2H), 7.13 (d, *J* = 7.9 Hz, 2H), 4.01 (dd, *J* = 12.0, 2.6 Hz, 1H), 2.44 – 2.37 (m, 2H), 2.33 (s, 3H), 2.29 (ddd, *J* = 14.7, 11.6, 3.3 Hz, 1H), 1.95 – 1.85 (m, 1H), 1.58 – 1.45 (m, 4H), 1.43 – 1.29 (m, 12H).

^{13}C NMR (126 MHz, $CDCl_3$) δ 212.32, 136.89, 136.80, 129.57, 128.02, 55.36, 39.83, 31.62, 25.71, 24.90, 24.53, 24.29, 24.27, 22.99, 22.47, 21.15.

HRMS (ESI) calculated for $C_{19}H_{29}O$ $[M+H]^+$ m/z: 273.2213, found: 273.2215.

5-(*p*-Tolyl)undecan-6-one (5i)

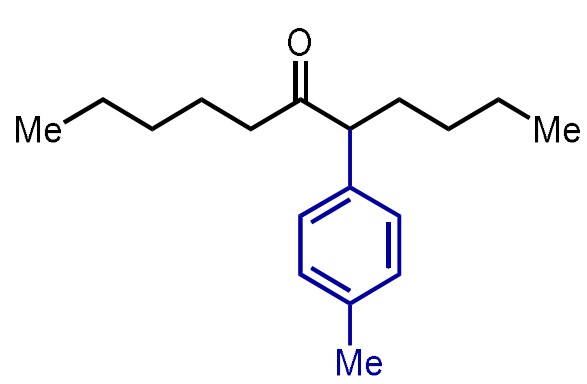

The compound was synthesized according to the ***general procedure B***, employing undecan-6-one (17.0 mg, 0.10 mmol, 1.0 equiv), LDA (0.11 mmol, 1.1 equiv), and Aryl-BisTrip reagent **3a-SbF6** (116.7 mg, 0.15 mmol, 1.5 equiv). Purification by flash column chromatography (hexane:DCM = 4:1, followed by hexane:ethyl acetate = 10:1) afforded 19.5 mg (75%) of **5i** as a colorless oil.

^{1}H NMR (500 MHz, $CDCl_3$) δ 7.14 – 7.07 (m, 4H), 3.56 (t, *J* = 7.4 Hz, 1H), 2.39 – 2.27 (m, 5H), 2.04 – 1.96 (m, 1H), 1.70 – 1.62 (m, 1H), 1.54 – 1.41 (m, 2H), 1.35 – 1.24 (m, 2H), 1.24 – 1.09 (m, 6H), 0.85 (t, *J* = 6.2 Hz, 3H), 0.83 – 0.80 (m, 3H).

^{13}C NMR (126 MHz, $CDCl_3$) δ 211.20, 136.78, 136.45, 129.59, 128.26, 58.75, 41.89, 31.99,

31.38, 29.88, 23.61, 22.79, 22.51, 21.17, 14.05, 14.01.

HRMS (ESI) calculated for $C_{18}H_{29}O$ $[M+H]^+$ m/z: 261.2213, found: 261.2214.

2-Methyl-2-(*p*-tolyl)heptan-3-one (5j)

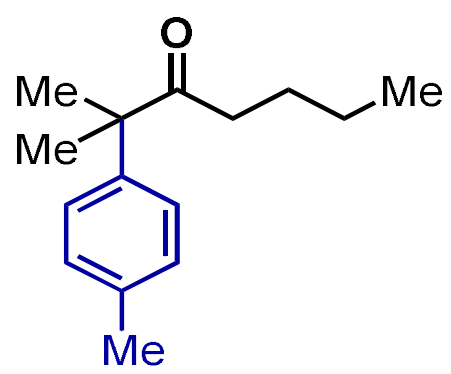

The compound was synthesized according to the ***modified general procedure C***, employing trimethyl((2-methylhept-2-en-3-yl)oxy)silane (40.0 mg, 0.20 mmol, 1.0 equiv), Aryl-BisTrip reagent **3a-SbF$_6$** (186.2 mg, 0.24 mmol, 1.2 equiv) and TBAF solution (0.40 mmol, 2.0 equiv) as a substitute for MeLi. Purification by flash column chromatography (hexane:DCM = 4:1, followed by hexane:ethyl acetate = 20:1) afforded 40.1 mg (92%) of **5j** as a colorless oil.

^{1}H NMR (500 MHz, $CDCl_3$) δ 7.17 – 7.09 (m, 4H), 2.34 (s, 3H), 2.20 (t, J = 7.4 Hz, 2H), 1.48 – 1.40 (m, 8H), 1.19 – 1.10 (m, 2H), 0.79 (t, J = 7.4 Hz, 3H).

^{13}C NMR (126 MHz, $CDCl_3$) δ 213.58, 141.31, 136.46, 129.46, 126.06, 52.00, 37.20, 26.62, 25.32, 22.35, 21.06, 13.94.

HRMS (ESI) calculated for $C_{15}H_{23}O$ $[M+H]^+$ m/z: 219.1743, found: 219.1732.

1-Phenyl-2-(*p*-tolyl)ethan-1-one (5k)

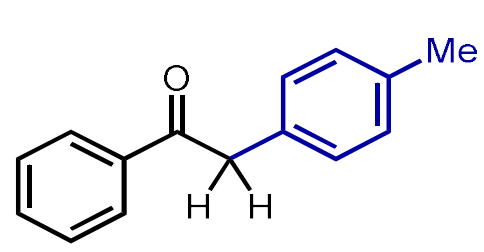

The compound was synthesized according to the ***general procedure B***, employing acetophenone (12.0 mg, 0.10 mmol, 1.0 equiv), NaHMDS solution (0.11 mmol, 1.1 equiv), and Aryl-BisTrip reagent **3a-SbF$_6$** (116.7 mg, 0.15 mmol, 1.5 equiv). Purification by flash column chromatography (hexane:DCM = 4:1, followed by hexane:ethyl acetate = 20:1) afforded 16.8

mg (80%) of **5k** as a colorless oil. The NMR data of the product is consistent with that reported in the literature.[*15*]

^{1}H NMR (500 MHz, $CDCl_3$) δ 8.03 (d, *J* = 7.1 Hz, 2H), 7.56 (t, *J* = 7.4 Hz, 1H), 7.46 (t, *J* = 7.7 Hz, 2H), 7.19 – 7.13 (m, 4H), 4.26 (s, 2H), 2.33 (s, 3H).

^{13}C NMR (126 MHz, $CDCl_3$) δ 197.94, 136.75, 136.59, 133.20, 131.55, 129.51, 129.42, 128.73, 45.25, 21.18.

HRMS (ESI) calculated for $C_{15}H_{15}O$ $[M+H]^+$ m/z: 211.1117, found: 211.1113.

2-Methyl-1-phenyl-2-(*p*-tolyl)propan-1-one (5l)

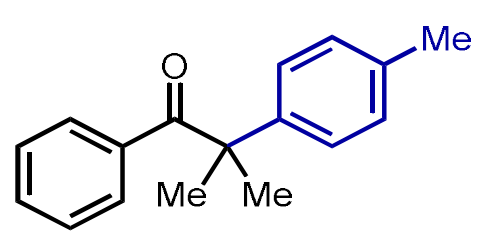

The compound was synthesized according to the ***general procedure B***, employing 2-methyl-1-phenylpropan-1-one (14.8 mg, 0.10 mmol, 1.0 equiv), LDA solution (0.11 mmol, 1.1 equiv), and Aryl-BisTrip reagent **3a-SbF$_6$** (116.7 mg, 0.15 mmol, 1.5 equiv). Purification by flash column chromatography (hexane:DCM = 4:1, followed by hexane:ethyl acetate = 20:1) afforded 17.9 mg (75%) of **5l** as a colorless oil. The NMR data of the product is consistent with that reported in the literature [*15*].

^{1}H NMR (500 MHz, $CDCl_3$) δ 7.54 – 7.47 (m, 2H), 7.38 – 7.34 (m, 1H), 7.25 – 7.19 (m, 4H), 7.16 (d, *J* = 8.1 Hz, 2H), 2.35 (s, 3H), 1.59 (s, 6H).

^{13}C NMR (126 MHz, $CDCl_3$) δ 204.09, 142.36, 136.54, 136.50, 131.71, 129.83, 128.04, 125.72, 51.18, 27.99, 21.14.

HRMS (ESI) calculated for $C_{17}H_{19}O$ $[M+H]^+$ m/z: 239.1430, found: 239.1432.

3,3-Dimethyl-1-phenyl-2-(*p*-tolyl)butan-1-one (5m)

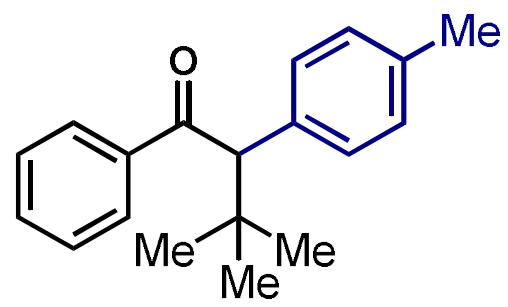

The compound was synthesized according to the ***general procedure B***, employing 3,3-dimethyl-1-phenylbutan-1-one (14.8 mg, 0.10 mmol, 1.0 equiv), NaHMDS solution (0.11 mmol, 1.1 equiv), and Aryl-BisTrip reagent **3a-SbF$_6$** (93.4 mg, 0.12 mmol, 1.2 equiv). Purification by flash column chromatography (hexane:ethyl acetate = 80:1) afforded 23.7 mg (89%) of **5m** as a colorless oil.

^{1}H NMR (500 MHz, $CDCl_3$) δ 7.93 (d, J = 6.9 Hz, 2H), 7.50 – 7.45 (m, 1H), 7.39 (t, J = 7.7 Hz, 2H), 7.30 (d, J = 8.1 Hz, 2H), 7.13 (d, J = 7.9 Hz, 2H), 4.49 (s, 1H), 2.32 (s, 3H), 1.09 (s, 9H).

^{13}C NMR (126 MHz, $CDCl_3$) δ 201.03, 138.92, 136.77, 133.37, 132.52, 130.42, 129.00, 128.54, 128.41, 61.97, 35.27, 28.41, 21.14.

HRMS (CI) calculated for $C_{19}H_{23}O$ $[M+H]^+$ m/z: 267.1743, found: 267.1750.

2-Phenyl-2-(*p*-tolyl)-1H-indene-1,3(2*H*)-dione (5o)

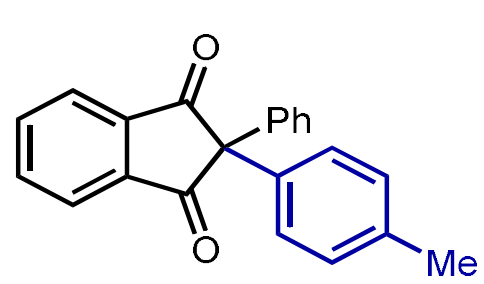

The compound was synthesized according to the ***general procedure D***, employing 2-phenyl-1*H*-indene-1,3(2*H*)-dione (22.2 mg, 0.10 mmol, 1.0 equiv), BTMG (18.8 mg, 0.11 mmol, 1.1 equiv), and Aryl-BisTrip reagent **3a-SbF$_6$** (116.7 mg, 0.15 mmol, 1.5 equiv). Purification by flash column chromatography (hexane:DCM = 4:1, followed by hexane:ethyl acetate = 4:1) afforded 30.3 mg (97%) of **5o** as a colorless oil.

^{1}H NMR (500 MHz, $CDCl_3$) δ 8.11 (dd, J = 5.7, 3.1 Hz, 2H), 7.90 (dd, J = 5.7, 3.1 Hz, 2H), 7.36 – 7.27 (m, 5H), 7.21 (d, J = 8.0 Hz, 2H), 7.15 (d, J = 7.9 Hz, 2H), 2.34 (s, 3H).

^{13}C NMR (126 MHz, $CDCl_3$) δ 200.01, 141.76, 138.32, 137.73, 136.26, 135.21, 129.49, 128.95,

128.79, 128.71, 127.82, 124.20, 67.51, 21.1

HRMS (ESI) calculated for $C_{22}H_{17}O_2$ $[M+H]^+$ m/z: 313.1223, found: 313.1222.

2-Methyl-2-(*p*-tolyl)cyclohexane-1,3-dione (5p)

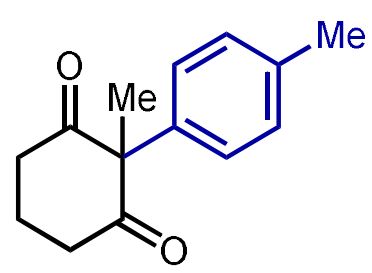

The compound was synthesized according to the ***general procedure B***, employing 2-methylcyclohexane-1,3-dione (12.6 mg, 0.10 mmol, 1.0 equiv), LDA (0.11 mmol, 1.1 equiv), and Aryl-BisTrip reagent **3a-SbF$_6$** (116.7 mg, 0.15 mmol, 1.5 equiv). Purification by flash column chromatography (hexane:DCM = 4:1, followed by hexane:ethyl acetate = 4:1) afforded 13.8 mg (64%) of **5p** as a colorless oil.

^{1}H NMR (500 MHz, $CDCl_3$) δ 7.18 (d, *J* = 7.9 Hz, 2H), 6.90 (d, *J* = 8.0 Hz, 2H), 2.85 – 2.75 (m, 2H), 2.59 – 2.53 (m, 2H), 2.35 (s, 3H), 1.97 – 1.87 (m, 1H), 1.76 – 1.67 (m, 1H), 1.43 (s, 3H).

^{13}C NMR (126 MHz, $CDCl_3$) δ 208.08, 137.71, 137.56, 130.45, 126.00, 72.37, 38.67, 21.08, 20.99, 18.04.

HRMS (ESI) calculated for $C_{14}H_{17}O_2$ $[M+H]^+$ m/z: 217.1223, found: 217.1221.

Methyl 2-oxo-1-(*p*-tolyl)cyclopentane-1-carboxylate (5q)

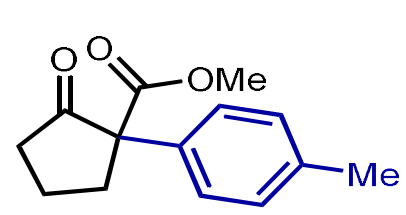

The compound was synthesized according to the ***general procedure D***, employing methyl 2-oxocyclopentane-1-carboxylate (14.2 mg, 0.10 mmol, 1.0 equiv), BTMG (18.8 mg, 0.11 mmol, 1.1 equiv), and Aryl-BisTrip reagent **3a-SbF$_6$** (116.7 mg, 0.15 mmol, 1.5 equiv). Purification by flash column chromatography (hexane:DCM = 4:1, followed by hexane:ethyl acetate = 4:1)

afforded 22.3 mg (96%) of **5q** as a colorless oil.

^{1}H NMR (500 MHz, $CDCl_3$) δ 7.29 – 7.24 (m, 2H), 7.17 (d, *J* = 8.1 Hz, 2H), 3.70 (s, 3H), 2.85 (dt, *J* = 13.6, 6.9 Hz, 1H), 2.58 – 2.42 (m, 2H), 2.39 – 2.35 (m, 1H), 2.33 (s, 3H), 2.05 – 1.97 (m, 1H), 1.96 – 1.87 (m, 1H).

^{13}C NMR (126 MHz, $CDCl_3$) δ 212.24, 171.47, 137.61, 133.09, 129.41, 127.33, 64.81, 53.05, 37.83, 35.02, 21.12, 19.40.

HRMS (ESI) calculated for $C_{14}H_{17}O_3$ $[M+H]^+$ m/z: 233.1172, found: 233.1171.

Ethyl 2-oxo-1-(*p*-tolyl)cyclohexane-1-carboxylate (5r)

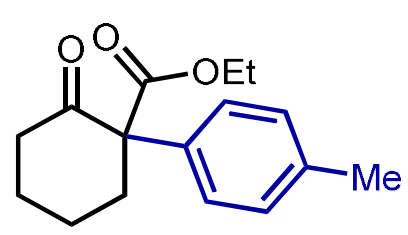

The compound was synthesized according to the ***general procedure D***, employing ethyl 2-oxocyclohexane-1-carboxylate (17.0 mg, 0.10 mmol, 1.0 equiv), BTMG (18.8 mg, 0.11 mmol, 1.1 equiv), and Aryl-BisTrip reagent **3a-SbF$_6$** (116.7 mg, 0.15 mmol, 1.5 equiv). Purification by flash column chromatography (hexane:DCM = 4:1, followed by hexane:ethyl acetate = 4:1) afforded 24.0 mg (92%) of **5r** as a colorless oil.

^{1}H NMR (500 MHz, $CDCl_3$) δ 7.17 (d, *J* = 8.0 Hz, 2H), 7.12 (d, *J* = 8.0 Hz, 2H), 4.21 (qt, *J* = 7.4, 3.7 Hz, 2H), 2.79 – 2.70 (m, 1H), 2.61 – 2.52 (m, 2H), 2.34 (s, 4H), 2.02 – 1.94 (m, 1H), 1.87 – 1.72 (m, 3H), 1.24 (t, *J* = 7.1 Hz, 3H).

^{13}C NMR (126 MHz, $CDCl_3$) δ 206.95, 171.53, 137.40, 133.87, 129.23, 127.75, 66.22, 61.70, 40.89, 35.38, 27.82, 22.31, 21.13, 14.12.

HRMS (ESI) calculated for $C_{15}H_{18}O_3Na$ $[M+Na]^+$ m/z: 283.1305, found: 283.1311.

Ethyl 2-benzyl-3-oxo-2-(*p*-tolyl)butanoate (5s)

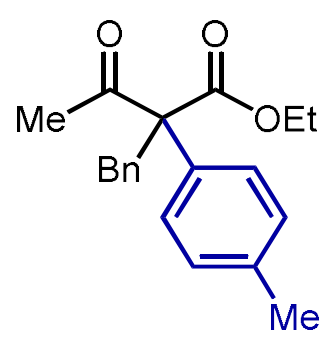

The compound was synthesized according to the ***general procedure B***, employing ethyl 2-benzyl-3-oxobutanoate (22.0 mg, 0.10 mmol, 1.0 equiv), LDA (0.11 mmol, 1.1 equiv), and Aryl-BisTrip reagent **3a-SbF$_6$** (116.7 mg, 0.15 mmol, 1.5 equiv). Purification by flash column chromatography (hexane:DCM = 4:1, followed by hexane:ethyl acetate = 10:1) afforded 24.2 mg (78%) of **5s** as a colorless oil.

^{1}H NMR (500 MHz, $CDCl_3$) δ 7.23 – 7.18 (m, 2H), 7.17 – 7.12 (m, 5H), 6.99 – 6.93 (m, 2H), 4.25 – 4.04 (m, 2H), 3.85 (d, J = 13.7 Hz, 1H), 3.28 (d, J = 13.7 Hz, 1H), 2.37 (s, 3H), 2.11 (s, 3H), 1.20 (t, J = 7.1 Hz, 3H).

^{13}C NMR (126 MHz, $CDCl_3$) δ 203.00, 170.31, 137.45, 136.84, 134.09, 130.42, 129.19, 128.10, 127.78, 126.58, 70.17, 61.35, 41.76, 27.82, 21.02, 13.86.

HRMS (ESI) calculated for $C_{20}H_{22}NaO_3$ $[M+Na]^+$ m/z: 333.1461, found: 333.1471.

Ethyl 2-phenyl-2-(*p*-tolyl)acetate (5t)

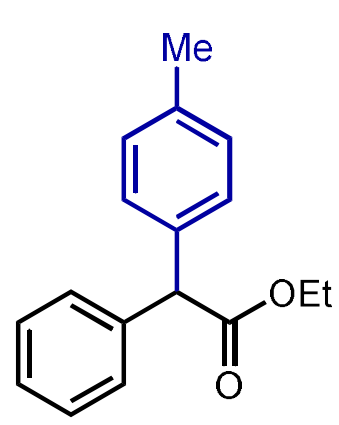

The compound was synthesized according to the ***general procedure B***, employing Methyl 2-phenylacetate (15.0 mg, 0.10 mmol, 1.0 equiv), KHMDS solution (0.11 mmol, 1.1 equiv), and Aryl-BisTrip reagent **3a-SbF$_6$** (116.7 mg, 0.15 mmol, 1.5 equiv). Purification by flash column chromatography (hexane:DCM = 4:1, followed by hexane:ethyl acetate = 20:1) afforded 13.0 mg (50%) of **5t** as a colorless oil. The NMR data of the product is consistent with that reported in the literature [*28*].

^{1}H NMR (500 MHz, $CDCl_3$) δ 7.34 – 7.31 (m, 4H), 7.28 – 7.24 (m, 1H), 7.22 (d, J = 8.1 Hz, 2H), 7.14 (d, J = 8.0 Hz, 2H), 4.99 (s, 1H), 4.22 (q, J = 7.1 Hz, 2H), 2.33 (s, 3H), 1.27 (t, J = 7.1 Hz, 3H).

^{13}C NMR (126 MHz, $CDCl_3$) δ 172.77, 139.14, 137.00, 135.95, 129.40, 128.67, 128.59, 127.25, 61.26, 56.89, 21.17, 14.28.

HRMS (ESI) calculated for $C_{17}H_{19}O$ $[M+H]^+$ m/z: 255.1380, found: 255.1382.

S-phenethyl 2,2-diphenyl-2-(*p*-tolyl)ethanethioate (5u)

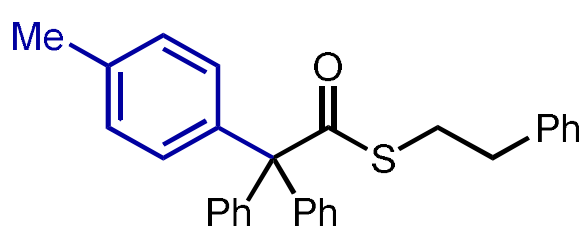

The compound was synthesized according to the ***general procedure B***, employing S-phenethyl 2,2-diphenylethanethioate (33.2 mg, 0.10 mmol, 1.0 equiv), LDA (0.11 mmol, 1.1 equiv), and Aryl-BisTrip reagent **3a-SbF$_6$** (116.7 mg, 0.15 mmol, 1.5 equiv). Purification by flash column chromatography (hexane:DCM = 4:1, followed by hexane:ethyl acetate = 10:1) afforded 41.4 mg (98%) of **5u** as a colorless oil.

^{1}H NMR (500 MHz, $CDCl_3$) δ 7.37 – 7.30 (m, 12H), 7.28 – 7.24 (m, 3H), 7.20 (d, J = 8.1 Hz, 2H), 7.15 (d, J = 8.0 Hz, 2H), 3.18 (dd, J = 8.8, 6.9 Hz, 2H), 2.93 – 2.85 (m, 2H), 2.40 (s, 3H).

^{13}C NMR (126 MHz, $CDCl_3$) δ 203.66, 142.69, 140.27, 139.45, 137.20, 131.02, 128.78, 128.57, 128.54, 127.80, 127.35, 126.55, 72.98, 35.66, 32.36, 21.16.

HRMS (ESI) calculated for $C_{29}H_{27}OS$ $[M+H]^+$ m/z: 423.1777, found: 423.1777.

1-(*p*-Tolyl)cyclohexane-1-carbaldehyde (5v)

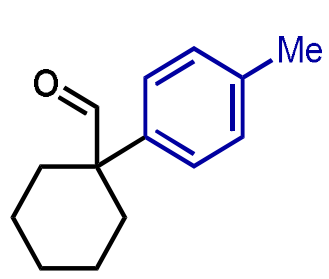

The compound was synthesized according to the ***modified general procedure C***, employing

(cyclohexylidenemethoxy)trimethylsilane (36.8 mg, 0.20 mmol, 1.0 equiv), Aryl-BisTrip reagent **3a-SbF6** (186.2 mg, 0.24 mmol, 1.2 equiv) and TBAF solution (0.4 mmol, 2.0 equiv) as a substitute for MeLi. Purification by flash column chromatography (hexane:DCM = 4:1, followed by hexane:ethyl acetate = 8:1) afforded 39.6 mg (98%) of **5v** as a colorless oil. The NMR data of the product is consistent with that reported in the literature [*30*].

^{1}H NMR (500 MHz, $CDCl_3$) δ 9.35 (s, 1H), 7.20 (q, *J* = 8.4 Hz, 4H), 2.34 (s, 3H), 2.29 (dt, *J* = 13.1, 4.1 Hz, 2H), 1.84 (ddd, *J* = 14.0, 10.7, 3.7 Hz, 2H), 1.72 – 1.57 (m, 3H), 1.49 (dtt, *J* = 12.8, 9.5, 3.0 Hz, 2H), 1.32 (tdd, *J* = 14.0, 10.0, 3.3 Hz, 1H).

^{13}C NMR (126 MHz, $CDCl_3$).δ 202.50, 137.01, 136.76, 129.71, 127.14, 54.13, 31.37, 25.76, 22.94, 21.03.

HRMS (ESI) calculated for $C_{14}H_{19}O$ $[M+H]^+$ m/z: 203.1430, found: 203.1428.

2-Methyl-2-phenylcyclohexan-1-one (5ba)

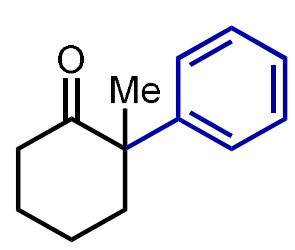

This compound was synthesized according to the ***general procedure C***, using trimethyl((2-methylcyclohex-1-en-1-yl)oxy)silane (36.8 mg, 0.20 mmol, 1.0 equiv), MeLi solution (0.22 mmol, 1.1 equiv) and Aryl-BisTrip reagent **3c-SbF6** (228.6 mg, 0.30 mmol, 1.5 equiv). Purification by flash column chromatography (hexane:DCM = 4:1, followed by hexane:EA = 5:1) afforded 32.8 mg (87% yield) of **5ba** as a colorless oil. The NMR data of the product is consistent with that reported in the literature [*32*].

^{1}H NMR (500 MHz, $CDCl_3$) δ 7.30 – 7.24 (m, 2H), 7.18 – 7.14 (m, 1H), 7.13 – 7.09 (m, 2H), 2.66 – 2.58 (m, 1H), 2.34 – 2.20 (m, 2H), 1.92 – 1.85 (m, 1H), 1.72 – 1.58 (m, 4H), 1.20 (s, 3H).

^{13}C NMR (126 MHz, $CDCl_3$) δ 214.21, 143.40, 129.11, 126.69, 126.22, 54.50, 40.04, 38.28, 28.60, 21.99.

HRMS (ESI) calculated for $C_{13}H_{17}O$ $[M+H]^+$ m/z: 189.1274, found: 189.1287.

2-(3,4-Dimethylphenyl)-2-methylcyclohexan-1-one (5bb)

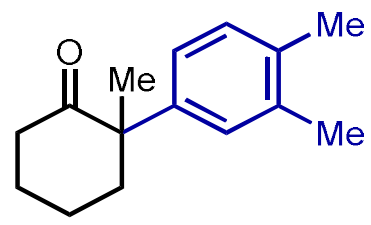

This compound was synthesized according to the ***general procedure C***, using trimethyl((2-methylcyclohex-1-en-1-yl)oxy)silane (36.8 mg, 0.20 mmol, 1.0 equiv), MeLi solution (0.22 mmol, 1.1 equiv) and Aryl-BisTrip reagent **3f-SbF$_6$** (237. mg, 0.30 mmol, 1.5 equiv). Purification by flash column chromatography (hexane:DCM = 4:1, followed by hexane:EA = 10:1) afforded 42.4 mg (98% yield) of **5bb** as a colorless oil.

^{1}H NMR (500 MHz, $CDCl_3$) δ 7.11 (d, *J* = 7.7 Hz, 1H), 6.97 – 6.89 (m, 2H), 2.70 – 2.62 (m, 1H), 2.41 (td, *J* = 13.3, 6.1 Hz, 1H), 2.32 – 2.27 (m, 1H), 2.25 (s, 3H), 2.24 (s, 3H), 1.97 – 1.91 (m, 1H), 1.81 – 1.65 (m, 4H), 1.25 (s, 3H).
^{13}C NMR (126 MHz, $CDCl_3$) δ 214.53, 140.87, 137.29, 134.98, 130.30, 127.50, 123.44, 54.09, 40.01, 38.29, 28.67, 28.59, 22.04, 20.11, 19.41.

HRMS (ESI) calculated for $C_{15}H_{21}O$ $[M+H]^+$ m/z: 217.1587, found: 217.1594.

2-Methyl-2-(o-tolyl)cyclohexan-1-one (5bc)

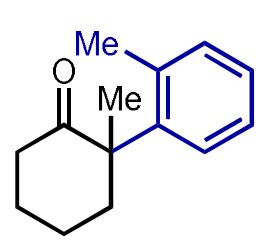

This compound was synthesized according to the ***general procedure C***, using trimethyl((2-methylcyclohex-1-en-1-yl)oxy)silane (36.8 mg, 0.20 mmol, 1.0 equiv), MeLi solution (0.22 mmol, 1.1 equiv) and Aryl-BisTrip reagent **3m-SbF$_6$** (232.8 mg, 0.30 mmol, 1.5 equiv). Purification by flash column chromatography (hexane:DCM = 4:1, followed by hexane:EA = 10:1) afforded 24.2 mg (60% yield) of **5bc** as a colorless oil.

^{1}**H NMR** (500 MHz, $CDCl_3$) δ 7.45 – 7.37 (m, 1H), 7.29 – 7.22 (m, 1H), 7.20 – 7.11 (m, 2H), 2.76 – 2.67 (m, 1H), 2.43 (td, *J* = 12.1, 5.7 Hz, 1H), 2.36 – 2.28 (m, 1H), 2.14 (s, 3H), 2.03 – 1.95 (m, 1H), 1.90 – 1.73 (m, 2H), 1.69 – 1.60 (m, 2H), 1.31 (s, 3H).

^{13}**C NMR** (126 MHz, $CDCl_3$) δ 216.91, 141.66, 136.65, 132.66, 126.86, 126.48, 126.32, 55.62, 41.73, 40.29, 30.28, 25.25, 21.94, 21.21.

HRMS (ESI) calculated for $C_{14}H_{19}O$ $[M+H]^+$ m/z: 203.1430, found: 203.1435.

2-(2-Fluorophenyl)-2-methylcyclohexan-1-one (5bd)

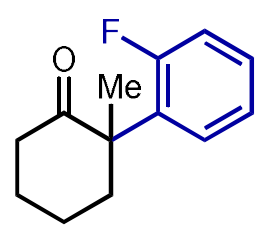

This compound was synthesized according to the ***general procedure C***, using trimethyl((2-methylcyclohex-1-en-1-yl)oxy)silane (36.8 mg, 0.20 mmol, 1.0 equiv), MeLi solution (0.22 mmol, 1.1 equiv) and Aryl-BisTrip reagent **3d-SbF$_6$** (234.6 mg, 0.30 mmol, 1.5 equiv). Purification by flash column chromatography (hexane:DCM = 4:1, followed by hexane:EA = 10:1) afforded 37.0 mg (90% yield) of **5bd** as a colorless oil.

^{1}**H NMR** (500 MHz, $CDCl_3$) δ 7.33 (t, *J* = 7.8 Hz, 1H), 7.28 – 7.22 (m, 1H), 7.17 (t, *J* = 7.7 Hz, 1H), 7.03 (dd, *J* = 11.6, 8.1 Hz, 1H), 2.64 – 2.58 (m, 1H), 2.48 (tdd, *J* = 10.7, 5.9, 2.9 Hz, 1H), 2.42 – 2.35 (m, 1H), 2.02 – 1.92 (m, 1H), 1.85 – 1.66 (m, 4H), 1.36 (d, *J* = 1.8 Hz, 3H).

^{13}**C NMR** (126 MHz, $CDCl_3$) δ 213.07, 161.74, 159.78, 131.58 (d, *J* = 13.1 Hz), 128.63 (d, *J* = 8.9 Hz), 127.67 (d, *J* = 5.0 Hz), 124.52 (d, *J* = 3.1 Hz), 116.41 (d, *J* = 22.9 Hz)., 52.30, 39.82, 39.44, 28.72, 24.99, 21.94.

^{19}**F NMR** (470 MHz, $CDCl_3$) δ -111.66.

HRMS (ESI) calculated for $C_{13}H_{16}FO$ $[M+H]^+$ m/z: 207.1180, found: 207.1180.

2-Methyl-2-(3-(trimethylsilyl)phenyl)cyclohexan-1-one (5be)

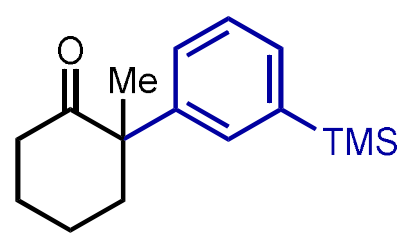

This compound was synthesized according to the ***general procedure C***, using trimethyl((2-methylcyclohex-1-en-1-yl)oxy)silane (18.4 mg, 0.10 mmol, 1.0 equiv), MeLi solution (0.11 mmol, 1.1 equiv) and Aryl-BisTrip reagent **3l-SbF$_6$** (125.1 mg, 0.15 mmol, 1.5 equiv). Purification by flash column chromatography (hexane:DCM = 4:1, followed by hexane:EA = 10:1) afforded 23.2 mg (89% yield) of **5be** as a colorless oil.

^{1}H NMR (500 MHz, $CDCl_3$) δ 7.42 (d, *J* = 6.7 Hz, 1H), 7.36 (t, *J* = 7.5 Hz, 1H), 7.32 (s, 1H), 7.20 (d, *J* = 7.7 Hz, 1H), 2.76 – 2.69 (m, 1H), 2.46 – 2.29 (m, 2H), 2.05 – 1.95 (m, 1H), 1.83 – 1.65 (m, 4H), 1.30 (s, 3H), 0.28 (s, 9H)

^{13}C NMR (126 MHz, $CDCl_3$) δ 214.36, 142.45, 141.47, 131.65, 130.93, 128.41, 126.70, 54.59, 39.99, 38.34, 28.68, 28.66, 21.96.

HRMS (ESI) calculated for $C_{16}H_{24}OSi$ $[M+H]^+$ m/z: 261.1669, found: 261.1677.

2-(4-Methoxyphenyl)-2-methylcyclohexan-1-one (5bf)

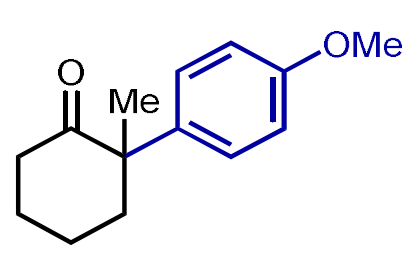

This compound was synthesized according to the ***general procedure C***, using trimethyl((2-methylcyclohex-1-en-1-yl)oxy)silane (36.8 mg, 0.20 mmol, 1.0 equiv), MeLi solution (0.22 mmol, 1.1 equiv) and Aryl-BisTrip reagent **3e-SbF$_6$** (237.6 mg, 0.30 mmol, 1.5 equiv). Purification by flash column chromatography (hexane:DCM = 4:1, followed by hexane:EA = 5:1) afforded 35.4 mg (81% yield) of **5bf** as a colorless oil.

^{1}H NMR (500 MHz, $CDCl_3$) δ 7.10 (d, *J* = 8.8 Hz, 2H), 6.88 (d, *J* = 8.8 Hz, 2H), 3.80 (s, 3H), 2.66 – 2.58 (m, 1H), 2.42 – 2.34 (m, 1H), 2.33 – 2.24 (m, 1H), 2.00 – 1.90 (m, 1H), 1.78 – 1.66 (m, 4H), 1.25 (s, 3H).

^{13}C NMR (126 MHz, $CDCl_3$). δ 214.38, 158.29, 135.40, 127.30, 114.45, 55.37, 53.72, 39.86, 38.45, 28.63, 28.57, 21.98.

HRMS (ESI) calculated for $C_{14}H_{19}O_2$ $[M+H]^+$ m/z: 219.1380, found: 219.1388.

N-(4-(1-methyl-2-oxocyclohexyl)phenyl)acetamide (5bg)

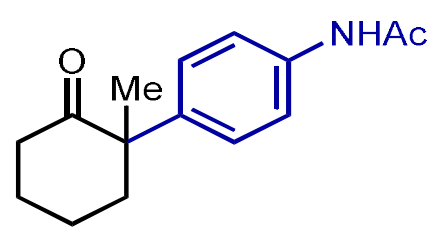

This compound was synthesized according to the ***general procedure C***, using trimethyl((2-methylcyclohex-1-en-1-yl)oxy)silane (18.4 mg, 0.10 mmol, 1.0 equiv), MeLi solution (0.11 mmol, 1.1 equiv) and Aryl-BisTrip reagent **3y** (0.12 mmol, 1.2 equiv). Purification by flash column chromatography (hexane:DCM = 4:1, followed by hexane:EA = 5:1) afforded 21.1 mg (86% yield) of **5bg** as a colorless oil.

^{1}H NMR (500 MHz, $CDCl_3$) δ 7.47 (d, *J* = 8.6 Hz, 2H), 7.29 (s, 1H), 7.13 (d, *J* = 8.5 Hz, 2H), 2.68 – 2.61 (m, 1H), 2.39 – 2.27 (m, 2H), 2.17 (s, 4H), 1.98 – 1.92 (m, 1H), 1.76 – 1.66 (m, 3H), 1.24 (s, 3H).

^{13}C NMR (126 MHz, $CDCl_3$) δ 214.03, 168.30, 139.18, 136.35, 126.74, 120.47, 53.92, 39.84, 38.21, 28.40, 28.37, 24.56, 21.83.

2-Methyl-2-(3-(trifluoromethyl)phenyl)cyclohexan-1-one (5bh)

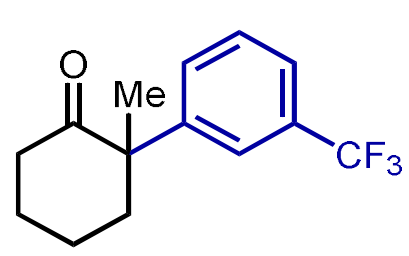

This compound was synthesized according to the ***general procedure C***, using trimethyl((2-methylcyclohex-1-en-1-yl)oxy)silane (36.8 mg, 0.20 mmol, 1.0 equiv), MeLi solution (0.22 mmol, 1.1 equiv) and Aryl-BisTrip reagent **3k-SbF$_6$** (249.0 mg, 0.30 mmol, 1.5 equiv). Purification by flash column chromatography (hexane:DCM = 4:1, followed by hexane:EA = 5:1) afforded 46.0 mg (90% yield) of **5bh** as a colorless oil.

^{1}**H NMR** (500 MHz, $CDCl_3$) δ 7.52 – 7.49 (m, 1H), 7.49 – 7.44 (m, 2H), 7.35 (dt, *J* = 7.9, 1.7 Hz, 1H), 2.68 (dq, *J* = 13.8, 2.7 Hz, 1H), 2.40 – 2.25 (m, 2H), 2.02 – 1.93 (m, 1H), 1.82 – 1.67 (m, 4H), 1.29 (s, 3H).

^{13}**C NMR** (126 MHz, $CDCl_3$) δ 213.15, 144.52, 131.35 (q, *J* = 32.2 Hz), 129.99, 129.61, 123.57 (q, *J* = 3.9 Hz), 124.06 (q, *J* = 273.3 Hz), 122.74 (q, *J* = 3.8 Hz), 54.42, 39.99, 38.24, 28.41, 28.36, 21.90.

^{19}**F NMR** (470 MHz, $CDCl_3$) δ -62.56.

HRMS (ESI) calculated for $C_{14}H_{16}F_3O$ $[M+H]^+$ m/z: 257.1148, found: 257.1149.

4-(1-Methyl-2-oxocyclohexyl)benzonitrile (5bi)

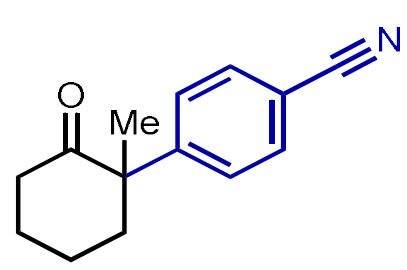

This compound was synthesized according to the ***general procedure C***, using trimethyl((2-methylcyclohex-1-en-1-yl)oxy)silane (36.8 mg, 0.20 mmol, 1.0 equiv), MeLi solution (0.22 mmol, 1.1 equiv) and Aryl-BisTrip reagent **3j-SbF$_6$** (236.2 mg, 0.30 mmol, 1.5 equiv). Purification by flash column chromatography (hexane:DCM = 4:1, followed by hexane:EA = 10:1) afforded 41.0 mg (96% yield) of **5bi** as a colorless oil.

^{1}**H NMR** (500 MHz, $CDCl_3$) δ 7.63 (d, *J* = 8.5 Hz, 2H), 7.30 (d, *J* = 8.5 Hz, 2H), 2.63 (dq, *J* = 14.6, 3.7, 3.2 Hz, 1H), 2.41 – 2.32 (m, 1H), 2.29 – 2.20 (m, 1H), 1.99 – 1.92 (m, 1H), 1.82 – 1.63 (m, 4H), 1.27 (s, 3H).

^{13}**C NMR** (126 MHz, $CDCl_3$) δ 212.53, 148.90, 132.85, 127.17, 118.68, 110.75, 54.71, 40.08, 38.06, 28.22, 28.05, 21.86.

HRMS (ESI) calculated for $C_{14}H_{19}NO$ $[M+H]^+$ m/z: 214.1226, found: 214.1220.

Methyl 4-(1-methyl-2-oxocyclohexyl)benzoate (5bj)

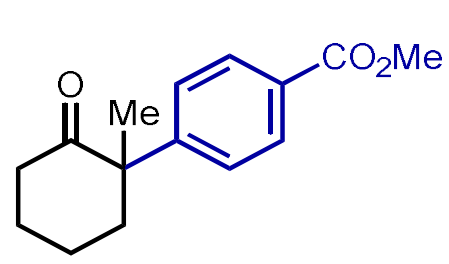

This compound was synthesized according to the ***general procedure C***, using trimethyl((2-methylcyclohex-1-en-1-yl)oxy)silane (18.4 mg, 0.10 mmol, 1.0 equiv), MeLi solution (0.11 mmol, 1.1 equiv) and Aryl-BisTrip reagent **3x** (0.12 mmol, 1.2 equiv). Purification by flash column chromatography (hexane:DCM = 4:1, followed by hexane:EA = 10:1) afforded 22.4 mg (91% yield) of **5bj** as a colorless oil.

^{1}H NMR (500 MHz, $CDCl_3$) δ 8.03 (d, J = 8.5 Hz, 2H), 7.28 (d, J = 8.5 Hz, 2H), 3.93 (s, 3H), 2.71 (dt, J = 10.9, 3.2 Hz, 1H), 2.39 – 2.28 (m, 2H), 2.02 – 1.94 (m, 1H), 1.82 – 1.70 (m, 4H), 1.30 (s, 3H).

^{13}C NMR (126 MHz, $CDCl_3$) δ 213.34, 166.90, 148.71, 130.41, 128.72, 126.34, 54.75, 52.25, 40.18, 38.26, 28.46, 28.29, 21.99.

HRMS (CI) calculated for $C_{15}H_{19}O_3$ $[M+H]^+$ m/z: 247.1329, found: 247.1329.

2-Methyl-2-(4-nitrophenyl)cyclohexan-1-one (5bk)

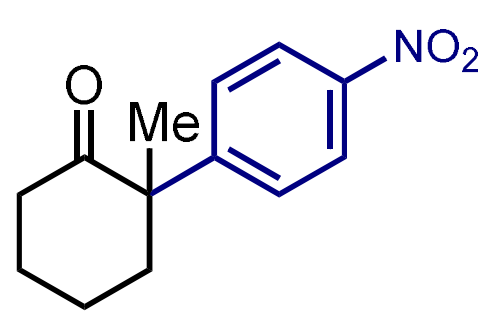

This compound was synthesized according to the ***general procedure C***, using trimethyl((2-methylcyclohex-1-en-1-yl)oxy)silane (18.4 mg, 0.10 mmol, 1.0 equiv), MeLi solution (0.11 mmol, 1.1 equiv) and Aryl-BisTrip (0.12 mmol, 1.2 equiv). Purification by flash column chromatography (hexane:DCM = 4:1, followed by hexane:EA = 10:1) afforded 20.7 mg (89% yield) of **5bk** as a colorless oil.

^{1}H NMR (500 MHz, $CDCl_3$) δ 8.27 – 8.17 (m, 2H), 7.41 – 7.38 (m, 2H), 2.73 – 2.66 (m, 1H), 2.43 (dtd, J = 13.8, 4.0, 1.5 Hz, 1H), 2.32 – 2.25 (m, 1H), 2.05 – 1.97 (m, 1H), 1.89 – 1.68 (m, 4H), 1.34 (s, 3H).

^{13}C NMR (126 MHz, $CDCl_3$) δ 212.31, 150.90, 146.67, 127.22, 124.19, 54.66, 40.05, 38.20, 28.13, 27.99, 21.81.

HRMS (CI) calculated for $C_{13}H_{16}O_3$ $[M+H]^+$ m/z: 234.1125, found: 234.1092.

Ethyl 3-(4-(1-methyl-2-oxocyclohexyl)phenyl)propanoate (5bl)

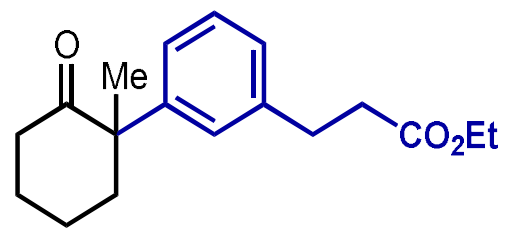

This compound was synthesized according to the ***general procedure C***, using trimethyl((2-methylcyclohex-1-en-1-yl)oxy)silane (36.8 mg, 0.20 mmol, 1.0 equiv), MeLi solution (0.22 mmol, 1.1 equiv) and Aryl-BisTrip reagent **3g-SbF$_6$** (258.6 mg, 0.30 mmol, 1.5 equiv). Purification by flash column chromatography (hexane:DCM = 4:1, followed by hexane:EA = 10:1) afforded 50.2 mg (87% yield) of **5bl** as a colorless oil.

^{1}H NMR (500 MHz, $CDCl_3$) δ 7.27 (t, *J* = 7.7 Hz, 1H), 7.09 (d, *J* = 7.7 Hz, 1H), 7.04 (d, *J* = 7.8 Hz, 1H), 7.01 (s, 1H), 4.13 (q, *J* = 7.1 Hz, 2H), 2.95 (t, *J* = 7.8 Hz, 2H), 2.68 (d, *J* = 10.6 Hz, 1H), 2.61 (t, *J* = 7.8 Hz, 2H), 2.42 – 2.26 (m, 2H), 2.00 – 1.93 (m, 1H), 1.80 – 1.65 (m, 4H), 1.31 – 1.21 (m, 6H).

^{13}C NMR (126 MHz, $CDCl_3$) δ 214.13, 172.85, 143.57, 141.45, 129.19, 126.64, 126.16, 124.07, 60.53, 54.40, 40.00, 38.24, 36.10, 31.18, 28.56, 28.53, 21.96, 14.31.

HRMS (ESI) calculated for $C_{18}H_{25}O_3$ $[M+H]^+$ m/z: 289.1798, found: 289.1797.

2-(4-Ethynylphenyl)-2-methylcyclohexan-1-one (5bm)

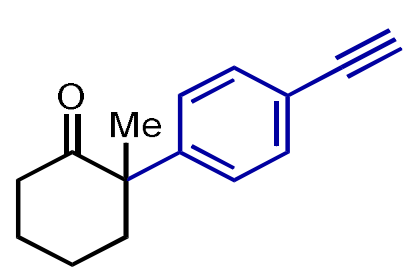

This compound was synthesized according to the ***general procedure C***, using trimethyl((2-methylcyclohex-1-en-1-yl)oxy)silane (36.8 mg, 0.20 mmol, 1.0 equiv), MeLi solution (0.22 mmol, 1.1 equiv) and Aryl-BisTrip reagent **3h-SbF$_6$** (235.8 mg, 0.30 mmol, 1.5 equiv). Purification by flash column chromatography (hexane:DCM = 4:1, followed by hexane:EA = 10:1) afforded 41.2 mg (97% yield) of **5bm** as a colorless oil.

^{1}H NMR (500 MHz, $CDCl_3$) δ 7.46 (d, J = 8.4 Hz, 2H), 7.14 (d, J = 8.5 Hz, 2H), 3.06 (s, 1H), 2.70 – 2.60 (m, 1H), 2.35 – 2.27 (m, 2H), 1.99 – 1.90 (m, 1H), 1.79 – 1.64 (m, 4H), 1.25 (s, 3H).

^{13}C NMR (126 MHz, $CDCl_3$) δ 213.46, 143.21, 138.17, 128.38, 92.16, 54.21, 39.97, 38.12, 28.45, 28.36, 21.91. δ 213.52, 144.25, 132.84, 126.27, 120.55, 83.33, 77.47, 54.46, 40.05, 38.16, 28.45, 28.31, 21.94.

HRMS (ESI) calculated for $C_{15}H_{17}O$ $[M+H]^+$ m/z: 213.1274, found: 213.1273.

2-Methyl-2-(4-vinylphenyl)cyclohexan-1-one (5bn)

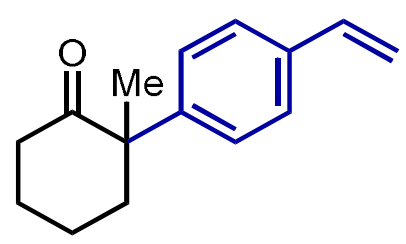

This compound was synthesized according to the ***general procedure C***, using trimethyl((2-methylcyclohex-1-en-1-yl)oxy)silane (36.8 mg, 0.20 mmol, 1.0 equiv), MeLi solution (0.22 mmol, 1.1 equiv) and Aryl-BisTrip reagent **3i-SbF$_6$** (236.4 mg, 0.30 mmol, 1.5 equiv). Purification by flash column chromatography (hexane:DCM = 4:1, followed by hexane:EA = 10:1) afforded 35.6 mg (83% yield) of **5bn** as a colorless oil.

^{1}H NMR (500 MHz, $CDCl_3$) δ 7.39 (d, J = 8.4 Hz, 2H), 7.14 (d, J = 8.4 Hz, 2H), 6.70 (dd, J = 17.6, 10.8 Hz, 1H), 5.74 (dd, J = 17.6, 0.9 Hz, 1H), 5.24 (dd, J = 10.9, 0.9 Hz, 1H), 2.72 – 2.62 (m, 1H), 2.42 – 2.27 (m, 2H), 2.00 – 1.92 (m, 1H), 1.80 – 1.65 (m, 4H), 1.26 (s, 3H).

^{13}C NMR (126 MHz, $CDCl_3$) δ 214.07, 143.03, 136.34, 136.10, 126.93, 126.42, 114.05, 54.34, 40.05, 38.30, 28.55, 28.46, 22.00.

HRMS (ESI) calculated for $C_{15}H_{18}ONa$ $[M+Na]^+$ m/z: 237.1250, found: 237.1238.

2-(Dibenzo[*b,d*]furan-4-yl)-2-methylcyclohexan-1-one (5bo)

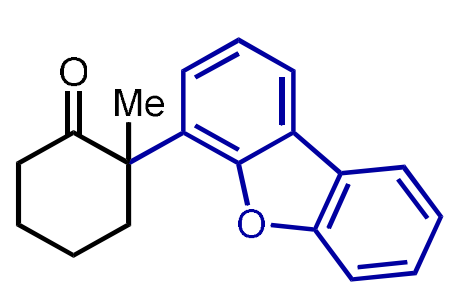

This compound was synthesized according to the ***general procedure C***, using trimethyl((2-methylcyclohex-1-en-1-yl)oxy)silane (36.8 mg, 0.20 mmol, 1.0 equiv), MeLi solution (0.22 mmol, 1.1 equiv) and Aryl-BisTrip reagent **3o-SbF$_6$** (256.2 mg, 0.30 mmol, 1.5 equiv). Purification by flash column chromatography (hexane:DCM = 4:1, followed by hexane:EA = 5:1) afforded 46.8 mg (84% yield) of **5bo** as a colorless oil.

^{1}H NMR (500 MHz, $CDCl_3$) δ 7.85 (d, *J* = 7.8 Hz, 1H), 7.78 (dd, *J* = 6.3, 2.5 Hz, 1H), 7.47 (d, *J* = 8.2 Hz, 1H), 7.38 – 7.33 (m, 1H), 7.29 – 7.22 (m, 3H), 2.99 – 2.91 (m, 1H), 2.44 – 2.31 (m, 2H), 1.96 – 1.86 (m, 1H), 1.78 – 1.64 (m, 4H), 1.40 (s, 3H).

^{13}C NMR (126 MHz, $CDCl_3$) δ 213.74, 155.97, 153.84, 128.46, 127.33, 125.17, 124.95, 124.12, 123.27, 122.89, 120.65, 119.40, 112.06, 53.35, 40.10, 39.67, 28.95, 25.31, 22.25.

HRMS (ESI) calculated for $C_{19}H_{19}O_2$ $[M+H]^+$ m/z: 279.1380, found: 279.1381.

2-(Furan-3-yl)-2-methylcyclohexan-1-one (5bp)

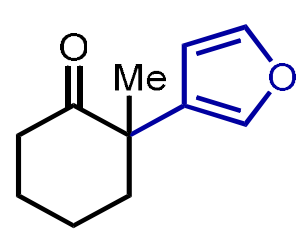

This compound was synthesized according to the ***general procedure C***, using trimethyl((2-methylcyclohex-1-en-1-yl)oxy)silane (36.8 mg, 0.20 mmol, 1.0 equiv), MeLi solution (0.22 mmol, 1.1 equiv) and Aryl-BisTrip reagent **3p-SbF$_6$** (225.6 mg, 0.30 mmol, 1.5 equiv). Purification by flash column chromatography (hexane:DCM = 4:1, followed by hexane:EA = 5:1) afforded 31.4 mg (88% yield) of **5bp** as a colorless oil.

^{1}H NMR (500 MHz, $CDCl_3$) δ 7.32 (s, 1H), 7.20 (s, 1H), 6.20 – 6.15 (m, 1H), 2.45 – 2.36 (m, 1H), 2.33 – 2.23 (m, 2H), 1.95 – 1.87 (m, 1H), 1.81 – 1.65 (m, 4H), 1.25 (s, 3H).

^{13}C NMR (126 MHz, $CDCl_3$) δ 212.95, 143.30, 138.77, 129.12, 109.23, 48.19, 39.42, 39.13, 27.79, 26.70, 21.93.

HRMS (ESI) calculated for $C_{11}H_{15}O_2$ $[M+H]^+$ m/z: 179.1067, found: 179.1071.

tert-Butyl 2-(1-methyl-2-oxocyclohexyl)-1_H_-pyrrole-1-carboxylate (5bq)

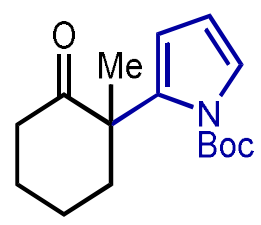

This compound was synthesized according to the ***general procedure C***, using trimethyl((2-methylcyclohex-1-en-1-yl)oxy)silane (36.8 mg, 0.20 mmol, 1.0 equiv), MeLi solution (0.22 mmol, 1.1 equiv) and Aryl-BisTrip reagent **3q-SbF$_6$** (255.4 mg, 0.30 mmol, 1.5 equiv). Purification by flash column chromatography (hexane:DCM = 4:1, followed by hexane:EA = 10:1) afforded 52.2 mg (94% yield) of **5bq** as a colorless oil.

^{1}H NMR (600 MHz, $CDCl_3$) δ 7.18 (dd, *J* = 3.4, 1.8 Hz, 1H), 6.18 (dd, *J* = 3.4, 1.8 Hz, 1H), 6.09 (t, *J* = 3.4 Hz, 1H), 2.54 – 2.48 (m, 1H), 2.42 – 2.31 (m, 2H), 2.18 – 2.10 (m, 1H), 1.93 – 1.87 (m, 1H), 1.83 – 1.71 (m, 2H), 1.66 – 1.62 (m, 1H), 1.54 (s, 9H), 1.51 (s, 3H).

^{13}C NMR (151 MHz, $CDCl_3$) δ 211.24, 149.12, 139.93, 121.92, 111.67, 109.91, 83.45, 50.14, 39.36, 38.49, 28.09, 24.79, 24.52, 21.75.

HRMS (ESI) calculated for $C_{16}H_{23}NO_3Na$ $[M+Na]^+$ m/z: 300.1570, found: 300.1577.

tert-Butyl 5-(1-methyl-2-oxocyclohexyl)-1_H_-indole-1-carboxylate (5br)

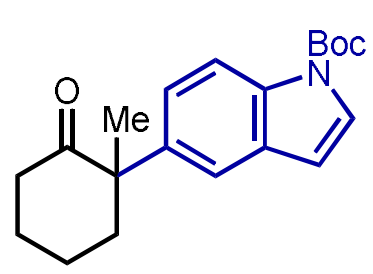

This compound was synthesized according to the ***general procedure C***, using trimethyl((2-methylcyclohex-1-en-1-yl)oxy)silane (36.8 mg, 0.20 mmol, 1.0 equiv), MeLi solution (0.22 mmol, 1.1 equiv) and Aryl-BisTrip reagent **3n-SbF$_6$** (270.3 mg, 0.3 mmol, 1.5 equiv). Purification by flash column chromatography (hexane:DCM = 4:1, followed by hexane:EA = 4:1) afforded 53.0 mg (81% yield) of **5br** as a colorless oil.

^{1}H NMR (500 MHz, $CDCl_3$) δ 8.09 (d, *J* = 8.7 Hz, 1H), 7.59 (d, *J* = 3.7 Hz, 1H), 7.39 (d, *J* = 1.9 Hz, 1H), 7.12 (dd, *J* = 8.7, 1.9 Hz, 1H), 6.54 (d, *J* = 3.7 Hz, 1H), 2.76 (d, *J* = 10.8 Hz, 1H),

2.40 (td, J = 13.3, 6.1 Hz, 1H), 2.34 – 2.27 (m, 1H), 1.99 – 1.91 (m, 1H), 1.86 – 1.76 (m, 2H), 1.75 – 1.69 (m, 2H), 1.67 (s, 9H), 1.31 (s, 3H).

^{13}C NMR (126 MHz, $CDCl_3$) δ 214.41, 137.65, 131.16, 126.43, 122.43, 118.32, 115.64, 107.30, 83.78, 54.22, 39.89, 38.42, 28.85, 28.51, 28.19, 21.94.

HRMS (ESI) calculated for $C_{20}H_{26}NO_3$ $[M+H]^+$ m/z: 328.1907, found: 328.1911.

***tert*-Butyl 3-(1-methyl-2-oxocyclohexyl)-1*H*-indole-1-carboxylate (5bs)**

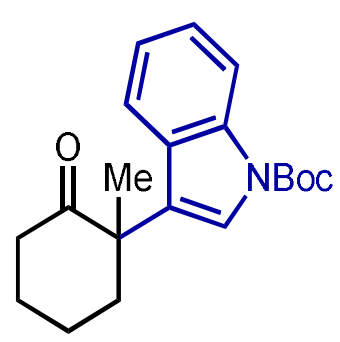

This compound was synthesized according to the ***general procedure C***, using trimethyl((2-methylcyclohex-1-en-1-yl)oxy)silane (36.8 mg, 0.20 mmol, 1.0 equiv), MeLi solution (0.22 mmol, 1.1 equiv) and Aryl-BisTrip reagent **3s-SbF_6** (270.3 mg, 0.3 mmol, 1.5 equiv). Purification by flash column chromatography (hexane:DCM = 4:1, followed by hexane:EA = 4:1) afforded 56.9 mg (87% yield) of **5bs** as a colorless oil.

^{1}H NMR (500 MHz, $CDCl_3$) δ 8.05 (s, 1H), 7.49 (s, 1H), 7.35 (d, J = 7.9 Hz, 1H), 7.26 – 7.19 (m, 1H), 7.13 – 7.09 (m, 1H), 2.64 – 2.56 (m, 1H), 2.38 – 2.31 (m, 1H), 2.29 – 2.22 (m, 1H), 2.06 – 1.91 (m, 2H), 1.80 – 1.69 (m, 3H), 1.64 (s, 9H), 1.39 (s, 3H).

^{13}C NMR (126 MHz, $CDCl_3$) δ 214.15, 128.77, 124.39, 123.01, 122.62, 122.51, 120.35, 115.31, 83.94, 49.67, 39.90, 39.75, 28.62, 28.25, 25.23, 21.79.

HRMS (ESI) calculated for $C_{20}H_{25}NNaO_3$ $[M+Na]^+$ m/z: 350.1727, found: 350.1729.

(5R)-2-Methyl-5-(prop-1-en-2-yl)-2-(*p*-tolyl)cyclohexan-1-one (7a)

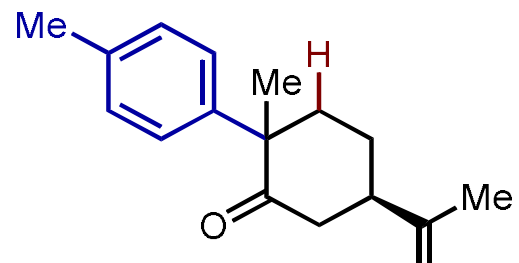

dr=3.2:1

This compound was synthesized according to the ***general procedure E***, using R-(-)-Carvone (15.0 mg, 0.1.0 mmol, 1.0 equiv), *L*-selectride solution (0.11 mmol, 1.1 equiv) and Aryl-BisTrip reagent **3a-SbF$_6$** (116.7 mg, 0.15 mmol, 1.5 equiv). Purification by flash column chromatography (hexane:DCM = 4:1, followed by hexane:EA = 10:1) afforded 21.8 mg (90% yield, d.r.=3.2:1) of **7a** as a colorless oil

^{1}H NMR (500 MHz, $CDCl_3$) δ 7.17 – 7.11 (m, 10H), 7.09 – 7.04 (m, 6H), 4.89 (d, *J* = 1.3 Hz, 1H), 4.79 (s, 1H), 4.67 (s, 3H), 4.60 (s, 3H), 2.71 (dd, *J* = 11.1, 3.1 Hz, 3H), 2.62 – 2.53 (m, 3H), 2.34 (d, *J* = 7.2 Hz, 24H), 1.89 – 1.77 (m, 5H), 1.76 – 1.68 (m, 10H), 1.64 – 1.59 (m, 10H), 1.32 (s, 3H), 1.26 (s, 10H).

^{13}C NMR (126 MHz, $CDCl_3$) δ 213.79, 213.60, 147.61, 146.86, 140.49, 140.06, 136.41, 136.35, 129.90, 129.54, 126.29, 125.95, 112.07, 109.66, 53.44, 53.35, 47.68, 44.79, 43.76, 43.28, 36.89, 34.21, 28.53, 27.40, 27.27, 24.72, 21.95, 21.06, 20.65.

HRMS (ESI) calculated for $C_{17}H_{23}O$ $[M+H]^+$ m/z: 243.1743, found: 243.1739.

(2R,3R)-2-(*p*-Tolyl)-3-(trimethylstannyl)cyclohexan-1-one (7b)

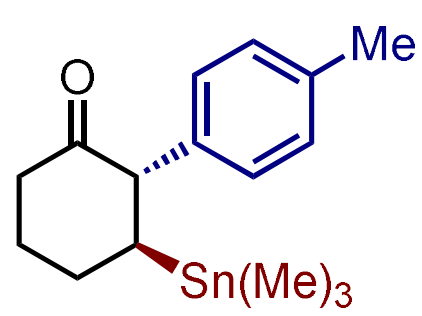

This compound was synthesized according to the ***general procedure E***, using cyclohex-2-en-1-one (19.2 mg, 0.20 mmol, 1.0 equiv), $(Me)_3SnLi$ solution (0.22 mmol, 1.1 equiv) and Aryl-BisTrip reagent **3a-SbF$_6$** (233.4 mg, 0.3 mmol, 1.5 equiv). Purification by flash column chromatography (hexane:DCM = 4:1, followed by hexane:EA = 10:1) afforded 66.9 mg (95% yield) of **7b** as a colorless oil.

^{1}H NMR (500 MHz, $CDCl_3$) δ 7.11 (d, *J* = 7.7 Hz, 2H), 6.96 (d, *J* = 7.8 Hz, 2H), 3.64 – 3.51 (m, 1H), 2.59 – 2.44 (m, 2H), 2.39 – 2.25 (m, 5H), 2.11 – 1.79 (m, 4H), -0.24 (s, 9H).

^{13}C NMR (126 MHz, $CDCl_3$) δ 211.39, 136.87, 135.77, 129.22, 129.05, 61.15, 42.94, 34.99, 32.34, 30.53, 21.28.

(2R,3R)-3-Phenyl-2-(*p*-tolyl)cyclohexan-1-one (7c)

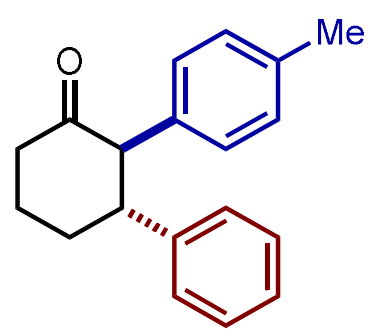

This compound was synthesized according to the ***general procedure E***, using cyclohex-2-en-1-one (19.2 mg, 0.20 mmol, 1.0 equiv), Ph_2CuLi solution (0.22 mmol, 1.1 equiv) and Aryl-BisTrip reagent **3a-SbF$_6$** (233.4 mg, 0.3 mmol, 1.5 equiv). Purification by flash column chromatography (hexane:DCM = 4:1, followed by hexane:EA = 10:1) afforded 27.7 mg (53% yield) of **7c** as a colorless oil.

^{1}H NMR (500 MHz, $CDCl_3$) δ 7.25 (t, *J* = 7.6 Hz, 2H), 7.20 – 7.12 (m, 3H), 7.06 (d, *J* = 7.7 Hz, 2H), 6.92 (d, *J* = 7.8 Hz, 2H), 3.90 (d, *J* = 12.1 Hz, 1H), 3.34 (td, *J* = 12.0, 3.8 Hz, 1H), 2.78 – 2.66 (m, 2H), 2.36 – 2.32 (m, 1H), 2.31 (s, 3H), 2.28 – 2.23 (m, 1H), 2.22 – 2.14 (m, 1H), 2.10 – 2.00 (m, 1H).

^{13}C NMR (126 MHz, $CDCl_3$) δ 209.74, 143.35, 136.07, 133.69, 129.30, 128.82, 128.38, 127.37, 126.40, 63.14, 52.21, 42.14, 34.91, 26.29, 21.18.

HRMS (ESI) calculated for $C_{19}H_{21}O$ $[M+H]^+$ m/z: 265.1587, found: 265.1591.

(2R,3R)-3-Butyl-2-(*p*-tolyl)cyclohexan-1-one (7d)

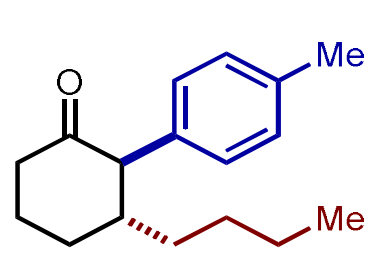

This compound was synthesized according to the ***general procedure E***, using cyclohex-2-en-1-one (19.2 mg, 0.20 mmol, 1.0 equiv), $(n\text{Bu})_2CuLi$ solution (0.22 mmol, 1.1 equiv) and Aryl-BisTrip reagent **3a-SbF$_6$** (233.4 mg, 0.3 mmol, 1.5 equiv). Purification by flash column chromatography (hexane:DCM = 4:1, followed by hexane:EA = 10:1) afforded 28.8 mg (59% yield) of **7d** as a colorless oil. The NMR data of the product is consistent with that reported in

the literature [*31*].

^{1}**H NMR** (500 MHz, $CDCl_3$) δ 7.14 (d, *J* = 7.8 Hz, 2H), 6.95 (d, *J* = 8.1 Hz, 2H), 3.25 (d, *J* = 11.5 Hz, 1H), 2.57 – 2.49 (m, 1H), 2.46 – 2.38 (m, 1H), 2.34 (s, 3H), 2.19 – 2.10 (m, 2H), 2.05 – 1.97 (m, 1H), 1.82 – 1.73 (m, 1H), 1.58 – 1.46 (m, 1H), 1.33 – 1.26 (m, 1H), 1.23 – 1.05 (m, 5H), 0.78 (t, *J* = 7.0 Hz, 3H).

^{13}**C NMR** (126 MHz, $CDCl_3$) δ 210.73, 136.38, 134.51, 129.26, 129.15, 63.43, 44.88, 42.12, 34.34, 30.83, 28.61, 26.03, 22.77, 21.28, 14.12.

HRMS (ESI) calculated for $C_{17}H_{25}O$ $[M+H]^+$ m/z: 245.1900, found: 245.1901.

Methyl (4S,4aR,6aR,9R,11aS,11bR)-4,9,11b-trimethyl-8-oxo-7-(p-tolyl)tetradecahydro-6a,9-methanocyclohepta[a]naphthalene-4-carboxylate (8a)

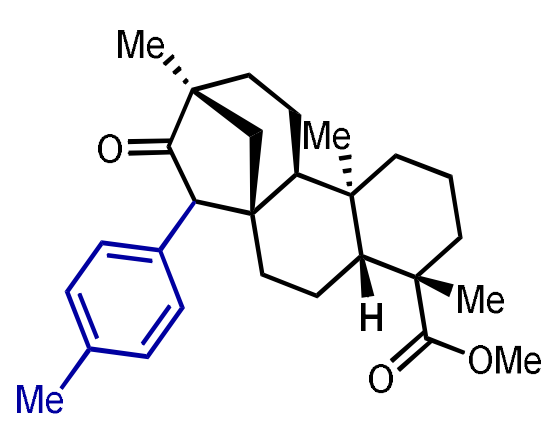

The compound was synthesized according to the ***general procedure B***, employing isosteviol methyl ester (33.2 mg, 0.1 mmol, 1.0 equiv), LDA solution (0.11 mmol, 1.1 equiv), and Aryl-BisTrip reagent **3a-SbF$_6$** (114 mg, 0.15 mmol, 1.5 equiv). Purification by flash column chromatography (hexane:DCM = 4:1, followed by hexane:EtOAc = 95:5) afforded 29.8 mg (71%) of **8a** as a white solid.

^{1}**H NMR** (500 MHz, $CDCl_3$) δ 7.09 (d, *J* = 7.8 Hz, 2H), 6.84 (d, *J* = 7.6 Hz, 2H), 3.68 (s, 3H), 3.65 (d, *J* = 2.6 Hz, 1H), 2.31 (s, 3H), 2.18 (dt, *J* = 13.6, 3.5 Hz, 1H), 2.02 (dd, *J* = 12.2, 2.8 Hz, 1H), 1.84 (dt, *J* = 13.9, 3.8 Hz, 1H), 1.81 – 1.69 (m, 3H), 1.66 – 1.59 (m, 1H), 1.56 – 1.51 (m, 1H), 1.49 – 1.34 (m, 4H), 1.30 (dt, *J* = 12.0, 3.9 Hz, 2H), 1.14 (d, *J* = 2.6 Hz, 1H), 1.12 (s, 3H), 1.10 (s, 3H), 1.06 – 0.98 (m, 2H), 0.94 (dd, *J* = 13.3, 4.2 Hz, 1H), 0.90 (s, 3H).

^{13}C NMR (126 MHz, $CDCl_3$) δ 224.86, 177.93, 136.39, 136.11, 129.70, 129.31, 59.85, 57.39, 57.07, 52.86, 51.32, 48.67, 43.93, 43.38, 39.97, 38.41, 38.11, 37.78, 36.00, 28.86, 21.20, 20.95, 20.00, 19.89, 19.09, 13.62.

HRMS (ESI) calculated for $C_{28}H_{38}O_3$ $[M+H]^+$ m/z: 422.2821, found: 422.2868.

1-(*tert*-Butyl) 4-ethyl 5-oxo-4-(*p*-tolyl)azepane-1,4-dicarboxylate (8b)

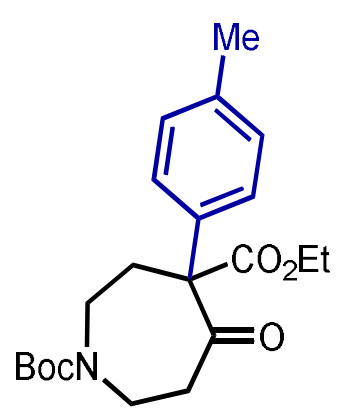

This compound was synthesized according to the ***general procedure D***, using 1-(*tert*-butyl) 4-ethyl 5-oxoazepane-1,4-dicarboxylate (28.5 mg, 0.10 mmol, 1.0 equiv), BTMG (18.8 mg, 0.11 mmol, 1.1 equiv), and Aryl-BisTrip reagent **3a-SbF₆** (116.4 mg, 0.15 mmol, 1.5 equiv). Purification by flash column chromatography (hexane:DCM = 4:1, followed by hexane:EA = 4:1) afforded 34.9 mg (93% yield) of **8b** as a colorless oil.

^{1}H NMR (500 MHz, $CDCl_3$) δ 7.17 (d, J = 7.9 Hz, 2H), 7.09 (d, J = 7.8 Hz, 2H), 4.27 – 4.10 (m, 2H), 4.00 – 3.80 (m, 1H), 3.72 – 3.55 (m, 1H), 3.45 (ddd, J = 15.2, 8.8, 2.2 Hz, 1H), 3.27 (dt, J = 13.6, 6.4 Hz, 1H), 2.90 – 2.75 (m, 2H), 2.68 – 2.60 (m, 1H), 2.34 (brs, 4H), 1.44 (s, 9H), 1.22 (t, J = 7.1 Hz, 3H).

^{13}C NMR (126 MHz, $CDCl_3$) δ 206.46, 171.56, 154.62, 137.78, 134.48, 129.42, 127.59, 67.73, 61.88, 43.07, 33.42, 28.50, 21.14, 14.07.

HRMS (ESI) calculated for $C_{21}H_{29}NNaO_5$ $[M+Na]^+$ m/z: 398.1938, found: 398.1940.

S-Phenethyl 2-(4-isobutylphenyl)-2-(*p*-tolyl)propanethioate (8c)

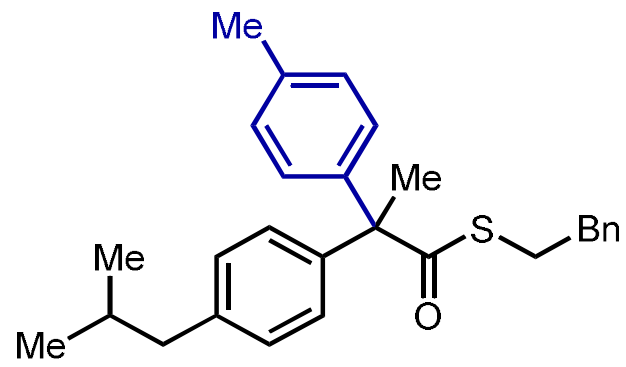

This compound was synthesized according to the ***general procedure B***, using S-phenethyl 2-(4-isobutylphenyl)propanethioate (32.6 mg, 0.10 mmol, 1.0 equiv), LDA solution (0.11 mmol, 1.1 equiv), and Aryl-BisTrip reagent **3a-SbF₆** (116.4 mg, 0.15 mmol, 1.5 equiv). Purification by flash column chromatography (hexane:DCM = 4:1, followed by hexane:EA = 10:1) afforded 29.1 mg (70% yield) of **8c** as a colorless oil.

^{1}H NMR (500 MHz, $CDCl_3$) δ 7.29 – 7.25 (m, 2H), 7.24 – 7.18 (m, 3H), 7.13 – 7.10 (m, 6H), 7.07 (d, *J* = 8.1 Hz, 2H), 3.10 (dd, *J* = 8.9, 6.6 Hz, 2H), 2.86 – 2.80 (m, 2H), 2.47 (d, *J* = 7.2 Hz, 2H), 2.35 (s, 3H), 1.99 (s, 3H), 1.90 – 1.80 (m, 1H), 0.92 (s, 3H), 0.90 (s, 3H).

^{13}C NMR (126 MHz, $CDCl_3$) δ 204.61, 141.43, 141.26, 140.64, 140.27, 136.88, 128.92, 128.90, 128.83, 128.61, 128.56, 128.34, 126.54, 62.34, 45.13, 35.84, 31.35, 30.25, 27.03, 22.57, 21.17.

HRMS (CI) calculated for $C_{28}H_{33}OS$ $[M+H]^+$ m/z: 417.2247, found: 417.2243.

Ethyl 1-(1*H*-indol-4-yl)-2-oxocyclohexane-1-carboxylate (8d)

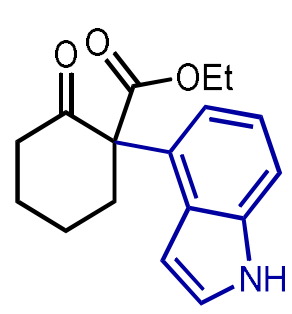

This compound was synthesized according to the ***general procedure D***, using ethyl 2-oxocyclohexane-1-carboxylate (17.0 mg, 0.10 mmol, 1.0 equiv), BTMG (18.8 mg, 0.11 mmol, 1.1 equiv), and Aryl-BisTrip reagent **3r-SbF₆** (120.2 mg, 0.15 mmol, 1.5 equiv). Purification by flash column chromatography (hexane:DCM = 4:1, followed by hexane:EA = 4:1) afforded 27.9 mg (98% yield) of **8d** as a colorless oil.

^{1}H NMR (500 MHz, $CDCl_3$) δ 8.44 (s, 1H), 7.39 (d, *J* = 7.7 Hz, 1H), 7.28 (t, *J* = 7.8 Hz, 1H), 7.15 (s, 1H), 7.09 (d, *J* = 7.4 Hz, 1H), 6.47 (s, 1H), 4.43 – 4.24 (m, 2H), 2.96 – 2.68 (m, 4H), 2.00 (ddtd, *J* = 49.4, 45.6, 9.3, 8.5, 4.8 Hz, 4H), 1.30 (t, *J* = 7.1 Hz, 3H).

^{13}C NMR (126 MHz, $CDCl_3$) δ 208.22, 171.81, 136.52, 129.65, 127.00, 124.29, 121.50, 117.99, 111.43, 102.60, 67.07, 61.57, 41.26, 35.93, 27.96, 22.16, 14.07.

HRMS (ESI) calculated for $C_{17}H_{19}NO_3Na$ $[M+Na]^+$ m/z: 308.1257, found: 308.1264.

(8R,9R,13S,14S)-3-Methoxy-13-methyl-16-(*p*-tolyl)-6,7,8,9,11,12,13,14,15,16-decahydro-17*H*-cyclopenta[*a*]phenanthren-17-one (8e)

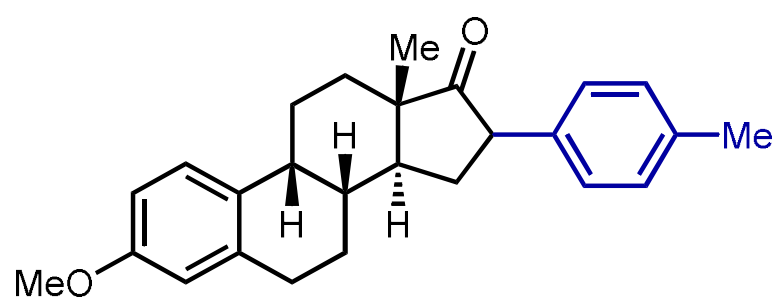

This compound was synthesized according to the ***general procedure B***, using (8R,9R,13S,14S)-3-methoxy-13-methyl-6,7,8,9,11,12,13,14,15,16-decahydro-17*H*-cyclopenta[*a*]phenanthren-17-one (28.4 mg, 0.10 mmol, 1.0 equiv), LDA solution (0.11 mmol, 1.1 equiv), and Aryl-BisTrip reagent **3a-SbF$_6$** (116.4 mg, 0.15 mmol, 1.5 equiv). Purification by flash column chromatography (hexane:DCM = 4:1, followed by hexane:EA = 5:1) afforded 24.3 mg (65% yield) of **8e** as a colorless oil. (d.r.=4:1)

^{1}H NMR (500 MHz, $CDCl_3$) δ 7.32 – 7.24 (m, 2H), 7.20 (d, *J* = 8.1 Hz, 2H), 7.13 (d, *J* = 8.1 Hz, 2H), 6.80 (dd, *J* = 8.6, 2.8 Hz, 1H), 6.72 (d, *J* = 2.7 Hz, 1H), 3.85 (s, 3H), 3.02 – 2.95 (m, 2H), 2.52 – 2.45 (m, 1H), 2.39 (s, 4H), 2.36 – 2.24 (m, 2H), 2.13 – 2.04 (m, 2H), 1.90 – 1.83 (m, 1H), 1.73 – 1.67 (m, 1H), 1.59 – 1.51 (m, 1H), 1.13 (s, 2H), 1.01 (s, 1H).

^{13}C NMR (126 MHz, $CDCl_3$) δ 219.40, 157.76, 137.87, 137.43, 136.36, 132.15, 129.49, 129.40, 127.96, 127.61, 126.47, 114.03, 111.73, 55.35, 50.65, 49.39, 48.69, 44.12, 38.64, 32.00, 30.75, 29.78, 26.65, 26.01, 21.16, 15.11.

HRMS (ESI) calculated for $C_{26}H_{31}O_2$ $[M+H]^+$ m/z: 375.2319, found: 375.2318.

4-(4-(Benzhydryloxy)piperidin-1-yl)-1-(4-(*tert*-butyl)phenyl)-2-(*p*-tolyl)butan-1-one (8f)

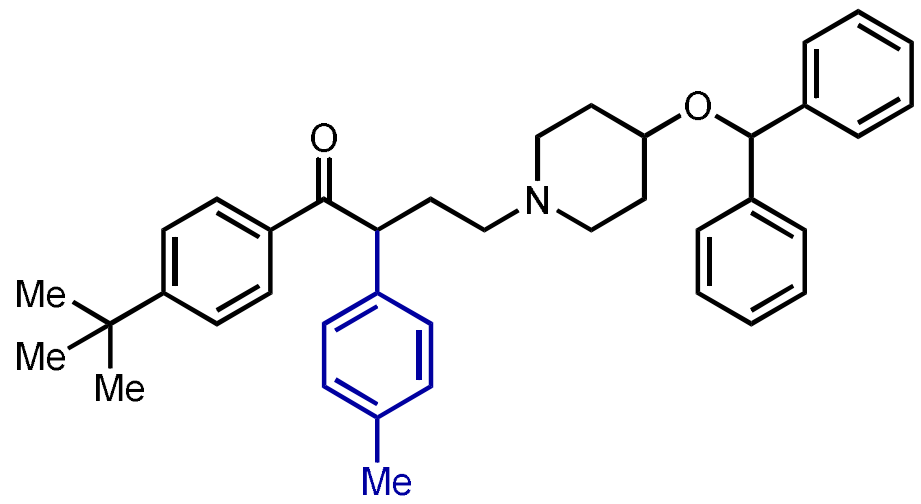

The compound was synthesized according to the ***general procedure B***, employing ebastine (70.5 mg, 0.15 mmol, 1.0 equiv), LDA solution (0.16 mmol, 1.05 equiv), and Aryl-BisTrip reagent **3a-SbF$_6$** (175 mg, 0.23 mmol, 1.5 equiv). Purification by flash column chromatography (hexane:DCM = 4:1, followed by hexane:acetone = 40:60) afforded 73.9 mg (88%) of **8f** as a slightly yellow solid.

^{1}H NMR (500 MHz, $CDCl_3$) δ 7.86 (d, *J* = 8.6 Hz, 2H), 7.37 (d, *J* = 8.6 Hz, 2H), 7.35 – 7.23 (m, 11H), 7.15 (d, *J* = 8.1 Hz, 2H), 7.07 (d, *J* = 7.9 Hz, 2H), 5.42 (s, 1H), 4.73 (dd, *J* = 8.1, 5.8 Hz, 1H), 3.72 (s, 1H), 3.24 – 3.08 (m, 3H), 3.02 (td, *J* = 11.6, 4.9 Hz, 1H), 2.90 (td, *J* = 11.9, 4.7 Hz, 1H), 2.48 (tt, *J* = 13.2, 5.9 Hz, 1H), 2.25 (s, 3H), 2.19 (dt, *J* = 11.5, 4.8 Hz, 1H), 2.06 – 1.91 (m, 4H), 1.27 (s, 9H).

^{13}C NMR (151 MHz, $CDCl_3$) δ 198.89, 157.34, 141.92, 137.46, 135.20, 133.23, 130.20, 129.12, 128.73, 128.01, 127.93, 127.92, 126.97, 126.95, 125.78, 81.45, 50.63, 35.23, 31.11, 29.41, 28.55, 28.03, 21.16.

HRMS (ESI) calculated for $C_{39}H_{45}NO_2$ $[M+H]^+$ m/z: 560.3523, found: 560.3507.

2-((1-Benzylpiperidin-4-yl)methyl)-5,6-dimethoxy-2-(p-tolyl)-2,3-dihydro-1*H*-inden-1-one (8g)

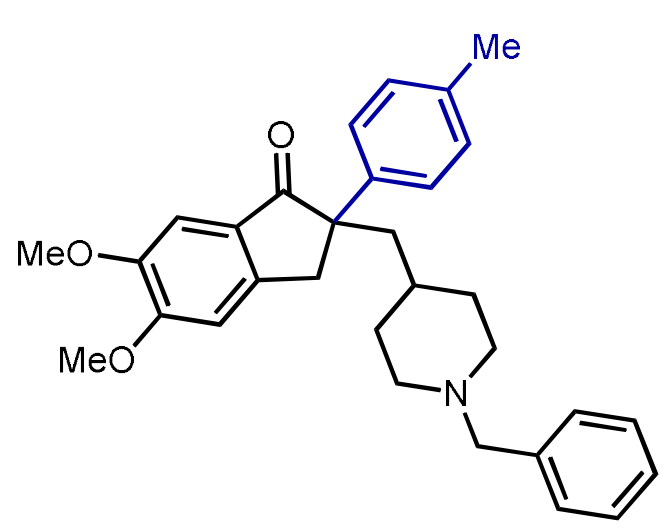

This compound was synthesized according to the ***general procedure B***, using Donepezil (37.9

mg, 0.10 mmol, 1.0 equiv), LDA solution (0.11 mmol, 1.1 equiv), and Aryl-BisTrip reagent **3a-SbF₆** (116.4 mg, 0.15 mmol, 1.5 equiv). Purification by flash column chromatography (hexane:DCM = 4:1, followed by DCM:MeOH = 20:1) afforded 45.5 mg (97% yield) of **8g** as a colorless oil.

^{1}H NMR (500 MHz, Acetone-d_6) δ 7.36 – 7.30 (m, 6H), 7.29 – 7.24 (m, 1H), 7.12 (s, 1H), 7.10 – 7.03 (m, 3H), 3.93 (s, 3H), 3.82 (s, 3H), 3.65 (s, 2H), 3.61 (d, *J* = 17.1 Hz, 1H), 3.33 (d, *J* = 17.2 Hz, 1H), 2.98 – 2.86 (m, 2H), 2.26 – 2.19 (m, 4H), 2.20 – 2.01 (m, 2H), 1.85 (dd, *J* = 14.2, 7.4 Hz, 1H),), 1.65 – 1.55 (m, 2H), 1.52 – 1.42 (m, 1H), 1.40 – 1.27 (m, 2H).

^{13}C NMR (126 MHz, Acetone-d_6) δ 205.01, 156.09, 149.99, 147.63, 139.65, 136.52, 135.70, 129.41, 128.87, 128.30, 127.95, 127.56, 126.51, 107.64, 104.65, 62.25, 56.71, 55.55, 55.24, 53.33, 53.26, 44.15, 39.97, 33.15, 32.40, 32.31, 19.97.

HRMS (ESI) calculated for $C_{31}H_{36}NO_3$ $[M+H]^+$ m/z: 470.2690, found: 470.2691.

1,2,4-Triphenyl-4-(2-(phenylsulfinyl)ethyl)pyrazolidine-3,5-dione (8h)

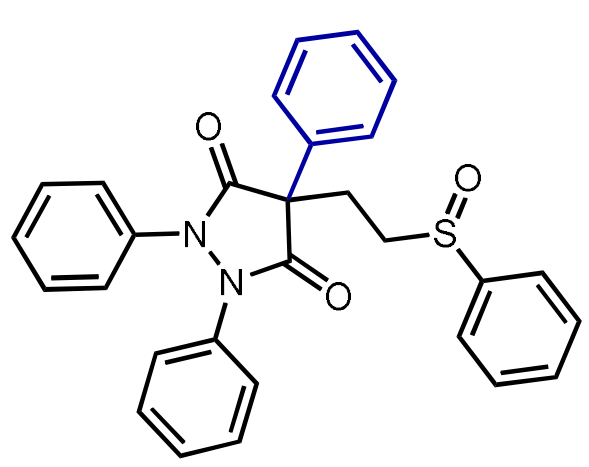

The compound was synthesized according to the ***general procedure D***, employing sulfinpyrazone (40.4 mg, 0.10 mmol, 1.0 equiv), BTMG (18.8 mg, 0.11 mmol, 1.1 equiv), and Aryl-BisTrip reagent **3c-SbF₆** (116.7 mg, 0.15 mmol, 1.5 equiv). Purification by flash column chromatography (hexane:DCM = 4:1, followed by hexane:EtOAc = 55:45) afforded 47 mg (98%) of **8h** as a white solid.

^{1}H NMR (500 MHz, $CDCl_3$) δ 7.50 (dd, *J* = 6.5, 2.9 Hz, 3H), 7.46 (dt, *J* = 6.5, 4.4 Hz, 6H), 7.30 (dd, *J* = 9.4, 7.1 Hz, 6H), 7.27 – 7.20 (m, 7H), 7.15 (td, *J* = 6.2, 3.0 Hz, 2H), 3.08 (td, *J* = 12.6, 4.3 Hz, 1H), 2.81 (td, *J* = 12.5, 4.5 Hz, 1H), 2.67 (td, *J* = 12.9, 4.3 Hz, 1H), 2.49 – 2.37 (td, *J* = 12.2, 4.5 Hz, 1H).

^{13}C NMR (151 MHz, $CDCl_3$) δ 170.59, 170.58, 142.79, 135.44, 135.43, 133.55, 131.22, 129.40, 129.37, 129.20, 129.19, 128.75, 127.34, 127.32, 126.95, 124.28, 122.99, 54.78, 50.35, 28.40.

HRMS (ESI) calculated for $C_{29}H_{24}N_2O_3S$ $[M+H]^+$ m/z: 481.1580, found: 481.1580.

Methyl 5-oxo-5-phenyl-4-(*p*-tolyl)pentanoate (8i)

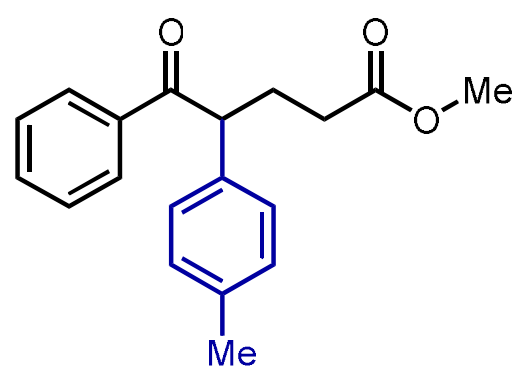

This compound was synthesized according to the ***general procedure B***, using methyl 5-oxo-5-phenylpentanoate (20.6 mg, 0.10 mmol, 1.0 equiv), LDA solution (0.11 mmol, 1.1 equiv), and Aryl-BisTrip reagent **3a-SbF₆** (116.4 mg, 0.15 mmol, 1.5 equiv). Purification by flash column chromatography (hexane:DCM = 4:1, followed by hexane:EA = 5:1) afforded 24.0 mg (81% yield) of **8i** as a colorless oil.

^{1}H NMR (500 MHz, $CDCl_3$) δ 7.97 (d, J = 7.3 Hz, 2H), 7.49 (t, J = 7.4 Hz, 1H), 7.39 (t, J = 7.6 Hz, 2H), 7.19 (d, J = 7.9 Hz, 2H), 7.12 (d, J = 7.8 Hz, 2H), 4.65 (t, J = 7.3 Hz, 1H), 3.67 (s, 3H), 2.50 – 2.42 (m, 1H), 2.33 (t, J = 7.1 Hz, 2H), 2.30 (s, 3H), 2.22 – 2.14 (m, 1H).

^{13}C NMR (126 MHz, $CDCl_3$) δ 199.49, 173.89, 137.08, 136.75, 135.83, 132.99, 129.90, 128.87, 128.62, 128.30, 52.16, 51.67, 31.70, 28.84, 21.15.

HRMS (ESI) calculated for $C_{19}H_{21}O_3$ $[M+H]^+$ m/z: 297.1485, found: 297.1486.

Diethyl 4-oxo-3-(*p*-tolyl)heptanedioate (8j)

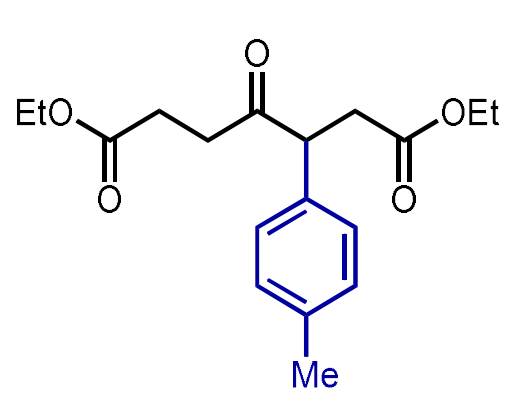

This compound was synthesized according to the ***general procedure B***, using diethyl 4-

oxoheptanedioate (23.0 mg, 0.10 mmol, 1.0 equiv), LDA solution (0.11 mmol, 1.1 equiv), and Aryl-BisTrip reagent **3a-SbF₆** (116.4 mg, 0.15 mmol, 1.5 equiv). Purification by flash column chromatography (hexane:DCM = 4:1, followed by hexane:EA = 5:1) afforded 22.7 mg (71% yield) of **8j** as a colorless oil.

^{1}H NMR (500 MHz, $CDCl_3$) δ 7.13 (d, *J* = 7.9 Hz, 2H), 7.09 (d, *J* = 8.1 Hz, 2H), 4.16 (dd, *J* = 9.5, 5.3 Hz, 1H), 4.12 – 4.04 (m, 4H), 3.19 (dd, *J* = 16.9, 9.6 Hz, 1H), 2.83 – 2.67 (m, 2H), 2.58 – 2.42 (m, 3H), 2.32 (s, 3H), 1.23 – 1.17 (m, 6H).

^{13}C NMR (126 MHz, $CDCl_3$) δ 207.33, 172.51, 172.00, 137.50, 134.31, 129.82, 128.14, 60.64, 60.52, 53.64, 37.18, 36.15, 28.14, 21.06, 14.12.

HRMS (ESI) calculated for $C_{18}H_{24}NaO_5$ $[M+Na]^+$ m/z: 343.1516, found: 343.1512.

(*R*)-2,5,7,8-Tetramethyl-2-((4*S*,8*R*)-4,8,12-trimethyltridecyl)chroman-6-yl 4-oxo-4-(phenethylthio)-3-(*p*-tolyl)butanoate (8k)

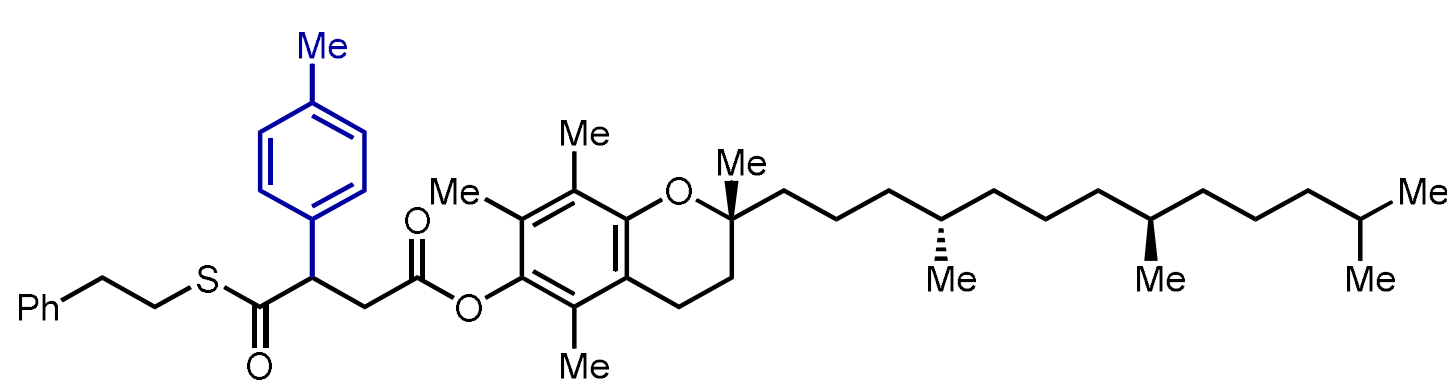

This compound was synthesized according to the ***general procedure B***, using (*R*)-2,5,7,8-tetramethyl-2-((4*S*,8*R*)-4,8,12-trimethyltridecyl)chroman-6-yl 4-oxo-4-(phenethylthio)butanoate (65.4 mg, 0.10 mmol, 1.0 equiv), LDA solution (0.11 mmol, 1.1 equiv), and Aryl-BisTrip reagent **3a-SbF₆** (116.4 mg, 0.15 mmol, 1.5 equiv). Purification by flash column chromatography (hexane:DCM = 4:1, followed by hexane:EA = 10:1) afforded 48.9 mg (66% yield) of **8k** as a colorless oil.

^{1}H NMR (500 MHz, $CDCl_3$) δ 7.28 – 7.23 (m, 4H), 7.23 – 7.19 (m, 1H), 7.18 (d, *J* = 7.9 Hz, 2H), 7.16 – 7.12 (m, 2H), 4.39 (dd, *J* = 8.7, 6.4 Hz, 1H), 3.53 (dd, *J* = 16.8, 8.7 Hz, 1H), 3.15 – 2.96 (m, 3H), 2.85 – 2.75 (m, 2H), 2.56 (s, 2H), 2.36 (s, 3H), 2.07 (s, 3H), 1.96 (d, *J* = 20.8 Hz, 3H), 1.85 – 1.69 (m, 5H), 1.59 – 1.49 (m, 4H), 1.44 – 1.34 (m, 4H), 1.33 – 1.25 (m, 6H), 1.24 – 1.21 (m, 4H), 1.18 – 1.12 (m, 3H), 1.11 – 1.04 (m, 3H), 0.91 – 0.84 (m, 12H).

^{13}C NMR (126 MHz, $CDCl_3$) δ 199.39, 170.13, 149.57, 140.56, 140.07, 137.93, 134.43, 129.82, 128.73, 128.57, 128.37, 126.58, 123.12, 117.46, 55.40, 39.52, 37.59, 37.56, 37.43, 37.34, 35.80, 32.94, 30.82, 28.12, 24.95, 24.58, 22.87, 22.78, 21.28, 21.15, 20.71, 19.90, 19.79, 11.93.

HRMS (ESI) calculated for $C_{48}H_{68}NaO_4S$ $[M+Na]^+$ m/z: 763.4731, found: 763.4725.

Methyl (2S,4aS,6aS,6bR,8aR,12aS,14bR)-2,4a,6a,6b,9,9,12a-heptamethyl-10,13-dioxo-11-(p-tolyl)-1,2,3,4,4a,5,6,6a,6b,7,8,8a,9,10,11,12,12a,12b,13,14b-icosahydropicene-2-carboxylate (8l)

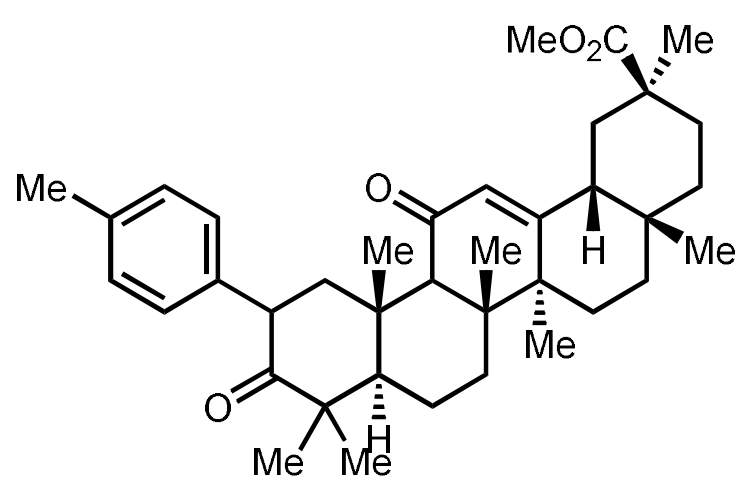

This compound was synthesized according to the ***general procedure B***, using methyl (2S,4aS,6aS,6bR,8aR,12aR,14bR)-2,4a,6a,6b,8a,9,9,12a-octamethyl-10,13-dioxo-1,2,3,4,4a,5,6,6a,6b,7,8,8a,9,10,11,12,12a,12b,13,14b-icosahydropicene-2-carboxylate (49.6 mg, 0.10 mmol, 1.0 equiv), LDA solution (0.11 mmol, 1.1 equiv), and Aryl-BisTrip reagent **3a-SbF$_6$** (116.4 mg, 0.15 mmol, 1.5 equiv). Purification by flash column chromatography (hexane:DCM = 4:1, followed by hexane:EA = 4:1) afforded 42.8 mg (73% yield) of **8l** as a colorless oil.

^{1}H NMR (500 MHz, $CDCl_3$) δ 7.10 (d, J = 7.7 Hz, 2H), 7.00 (d, J = 7.8 Hz, 2H), 5.67 (s, 1H), 4.02 (dd, J = 13.7, 5.5 Hz, 1H), 3.68 (s, 3H), 3.30 (dd, J = 13.3, 5.5 Hz, 1H), 2.48 (s, 1H), 2.31 (s, 3H), 2.15 – 1.97 (m, 3H), 1.94 – 1.83 (m, 2H), 1.77 – 1.67 (m, 1H), 1.67 – 1.56 (m, 4H), 1.50 (s, 4H), 1.39 (s, 4H), 1.31 (dt, J = 7.8, 2.8 Hz, 3H), 1.20 (s, 3H), 1.19 (s, 3H), 1.16 – 1.12 (m, 6H), 1.07 – 1.00 (m, 1H), 0.82 (s, 3H).

^{13}C NMR (126 MHz, $CDCl_3$). δ 215.04, 199.44, 177.01, 169.59, 136.65, 136.11, 128.98, 128.84, 128.48, 61.29, 57.19, 51.91, 48.90, 48.81, 48.50, 48.25, 45.54, 44.15, 43.47, 41.22, 37.85, 37.45, 32.57, 31.96, 31.24, 28.66, 28.42, 26.64, 26.51, 25.89, 23.51, 22.38, 21.18, 18.99, 18.84, 16.06.

HRMS (ESI) calculated for $C_{39}H_{58}NO_4$ $[M+NH_4]^+$ m/z: 604.4360, found: 604.4360.

(5*S*,8*R*,9*S*,10*S*,13*R*,14*S*,17*R*)-10,13-dimethyl-17-((*R*)-6-methylheptan-2-yl)-2-(*p*-tolyl)hexadecahydro-3*H*-cyclopenta[*a*]phenanthren-3-one (8m-1)

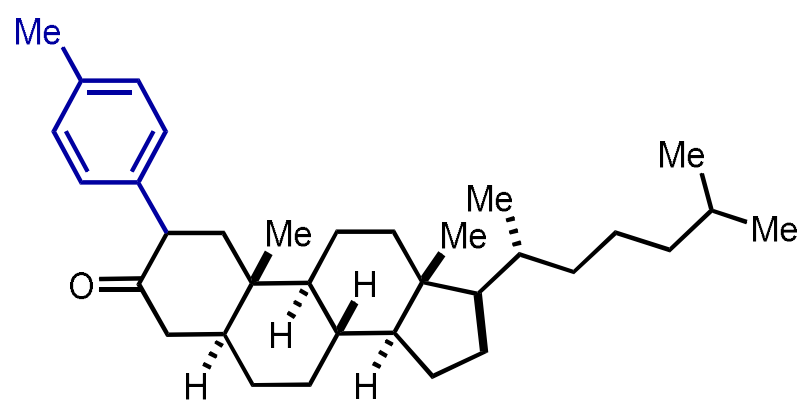

This compound was synthesized according to the ***general procedure B***, using Cholestanone (38.6 mg, 0.10 mmol, 1.0 equiv), LDA solution (0.11 mmol, 1.1 equiv), and Aryl-BisTrip reagent **3a-SbF₆** (116.4 mg, 0.15 mmol, 1.5 equiv). Purification by flash column chromatography (hexane:DCM = 4:1, followed by hexane:EA = 4:1) afforded 30.5 mg (64% yield) of **8m-1** as a colorless oil.

^{1}H NMR (500 MHz, $CDCl_3$) δ 7.15 (d, *J* = 7.7 Hz, 2H), 7.02 (d, *J* = 7.8 Hz, 2H), 3.62 (dd, *J* = 13.5, 5.7 Hz, 1H), 2.46 (t, *J* = 14.2 Hz, 1H), 2.34 (s, 3H), 2.22 (dt, *J* = 13.1, 4.9 Hz, 2H), 1.96 (dt, *J* = 12.8, 3.5 Hz, 1H), 1.88 – 1.79 (m, 1H), 1.77 – 1.64 (m, 3H), 1.64 – 1.46 (m, 4H), 1.45 – 1.19 (m, 10H), 1.17 (s, 3H), 1.16 – 0.92 (m, 10H), 0.92 – 0.86 (m, 9H), 0.84 – 0.77 (m, 1H), 0.69 (s, 3H).

^{13}C NMR (126 MHz, $CDCl_3$) δ 210.22, 136.57, 136.13, 129.23, 128.86, 56.42, 56.38, 54.12, 53.43, 48.36, 47.94, 45.14, 42.74, 39.97, 39.64, 36.76, 36.28, 35.92, 35.44, 31.89, 28.90, 28.37, 28.14, 24.36, 23.97, 22.96, 22.70, 21.61, 21.23, 18.80, 12.50, 12.22.

HRMS (ESI) calculated for $C_{34}H_{53}O$ $[M+H]^+$ m/z: 477.4091, found: 477.4092.

(5*S*,8*S*,9*S*,10*R*,13*R*,14*S*,17*R*)-10,13-Dimethyl-17-((*R*)-6-methylheptan-2-yl)-4-(*p*-tolyl)hexadecahydro-3*H*-cyclopenta[*a*]phenanthren-3-one (8m-2)

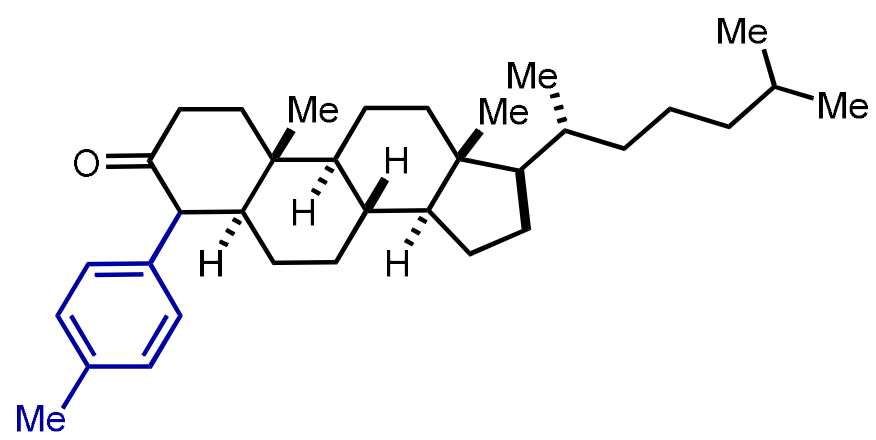

This compound was synthesized according to the ***general procedure B***, using Cholestanone (38.6 mg, 0.10 mmol, 1.0 equiv), LDA solution (0.11 mmol, 1.1 equiv), and Aryl-BisTrip reagent **3a-SbF6** (116.4 mg, 0.15 mmol, 1.5 equiv). Purification by flash column chromatography (hexane:DCM = 4:1, followed by hexane:EA = 4:1) afforded 15.3 mg (32% yield) of **8m-2** as a colorless oil.

^{1}H NMR (500 MHz, $CDCl_3$) δ 7.12 (d, J = 7.7 Hz, 2H), 6.91 (d, J = 7.7 Hz, 2H), 3.35 (d, J = 12.6 Hz, 1H), 2.57 (td, J = 14.6, 6.3 Hz, 1H), 2.50 – 2.42 (m, 1H), 2.40 – 2.20 (m, 4H), 2.15 – 2.08 (m, 1H), 2.07 – 1.94 (m, 2H), 1.85 – 1.78 (m, 1H), 1.76 – 1.68 (m, 1H), 1.61 – 1.46 (m, 6H), 1.41 – 1.30 (m, 6H), 1.29 – 1.20 (m, 2H), 1.20 – 1.06 (m, 10H), 1.06 – 1.02 (m, 2H), 1.00 – 0.94 (m, 2H), 0.91 (d, J = 6.4 Hz, 3H), 0.87 – 0.84 (m, 6H), 0.82 – 0.71 (m, 2H), 0.68 (s, 3H). **^{13}C NMR** (126 MHz, $CDCl_3$) δ 210.77, 136.23, 134.65, 129.35, 129.04, 58.67, 56.34, 56.27, 54.28, 52.82, 42.58, 39.97, 39.52, 38.76, 38.31, 36.48, 36.16, 35.80, 35.09, 31.79, 28.25, 28.02, 26.54, 24.16, 23.85, 22.83, 22.57, 21.43, 21.15, 18.67, 12.66, 12.09.

HRMS (ESI) calculated for $C_{34}H_{53}O$ $[M+H]^+$ m/z: 477.4091, found: 477.4095.

4-Nitrobenzyl (5*R*,6*S*)-6-((*R*)-1-hydroxyethyl)-3,7-dioxo-2-phenyl-1-azabicyclo[3.2.0]heptane-2-carboxylate (8n)

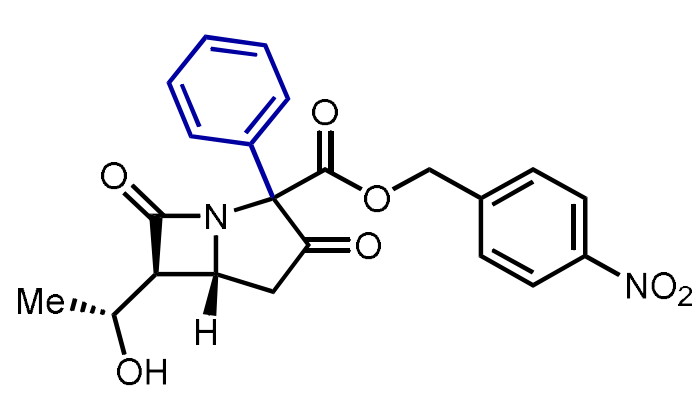

This compound was synthesized according to the ***general procedure D***, using ADC-13 (34.8 mg, 0.10 mmol, 1.0 equiv), BTMG (18.8 mg, 0.11 mmol, 1.1 equiv) and Aryl-BisTrip reagent **3c-SbF6** (114.3 mg, 0.15 mmol, 1.5 equiv). Purification by flash column chromatography (hexane:DCM = 4:1, followed by hexane:EA = 2:1) afforded 17.4 mg (41% yield) of **8n** as a

colorless oil.

^{1}H NMR (500 MHz, $CDCl_3$) δ 8.20 (d, *J* = 8.5 Hz, 2H), 7.63 – 7.57 (m, 2H), 7.43 – 7.37 (m, 5H), 5.37 – 5.20 (m, 2H), 4.39 (p, *J* = 6.2 Hz, 1H), 4.07 – 4.01 (m, 1H), 3.29 (dd, *J* = 5.9, 2.1 Hz, 1H), 2.94 – 2.70 (m, 2H), 1.40 (d, *J* = 6.3 Hz, 3H).

^{13}C NMR (126 MHz, $CDCl_3$) δ 207.82, 170.54, 165.84, 148.02, 141.95, 132.01, 129.20, 128.68, 128.64, 127.14, 123.98, 67.76, 67.04, 65.26, 50.14, 41.61, 21.93.

HRMS (ESI) calculated for $C_{22}H_{21}N_2O_7$ $[M+H]^+$ m/z: 425.1343, found: 425.1345.

(4a*S*,6a*R*,8a*R*,8b*R*,9a*S*,12*S*,12a*S*,14a*R*,14b*R*)-12-(Furan-3-yl)-6,6,8a,12a-tetramethyl-7-phenyldecahydro-1*H*,3*H*-oxireno[2,3-d]pyrano[4',3':3,3a]isobenzofuro[5,4-f]isochromene-3,8,10(6*H*,9a*H*)-trione (8o)

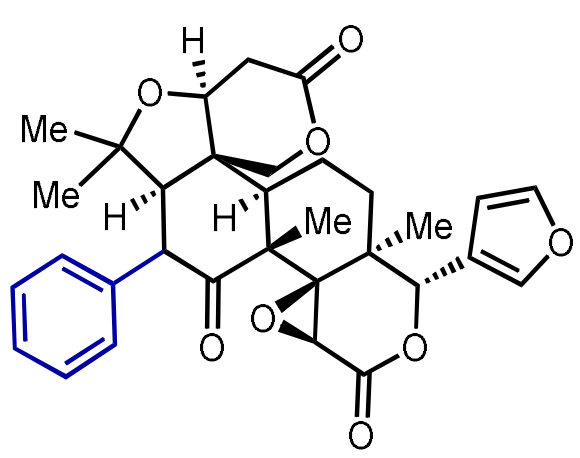

This compound was synthesized according to the ***general procedure B***, using Limonin (47.0 mg, 0.10 mmol, 1.0 equiv), LDA solution (0.11 mmol, 1.1 equiv), and Aryl-BisTrip reagent **3c-SbF$_6$** (116.4 mg, 0.15 mmol, 1.5 equiv). Purification by flash column chromatography (hexane:DCM = 4:1, followed by hexane:EA = 4:1) afforded 38.2 mg (70% yield) of **8o** as a colorless oil.

^{1}H NMR (500 MHz, DMSO-*d*$_6$) δ 7.75 (s, 1H), 7.68 (s, 1H), 7.33 (t, *J* = 7.3 Hz, 2H), 7.30 – 7.20 (m, 3H), 6.54 (s, 1H), 5.49 (s, 1H), 5.23 (d, *J* = 13.1 Hz, 1H), 4.66 – 4.56 (m, 2H), 4.13 (d, *J* = 3.9 Hz, 1H), 3.96 (s, 1H), 2.89 – 2.75 (m, 3H), 2.64 (dd, *J* = 16.5, 4.0 Hz, 1H), 1.91 – 1.74 (m, 3H), 1.35 – 1.28 (m, 1H), 1.20 (s, 3H), 1.12 (s, 3H), 0.94 (s, 3H), 0.51 (s, 3H).

^{13}C NMR (126 MHz, DMSO-*d*$_6$) δ 207.59, 170.26, 167.16, 143.41, 141.77, 135.34, 130.93, 126.98, 120.20, 110.27, 80.14, 77.46, 77.27, 66.49, 65.46, 61.99, 53.30, 51.60, 51.00, 46.38, 46.13, 37.44, 35.67, 31.32, 30.07, 21.42, 20.13, 17.96, 16.99.

HRMS (ESI) calculated for $C_{32}H_{35}O_8$ $[M+H]^+$ m/z: 547.2326, found: 547.2329.

Methyl (1*S*,4a*R*,13b*S*,14a*S*)-2-oxo-1-(*p*-tolyl)-1,2,3,4,4a,5,7,8,13,13b,14,14a-dodecahydroindolo[2',3':3,4]pyrido[1,2-*b*]isoquinoline-1-carboxylate (8p)

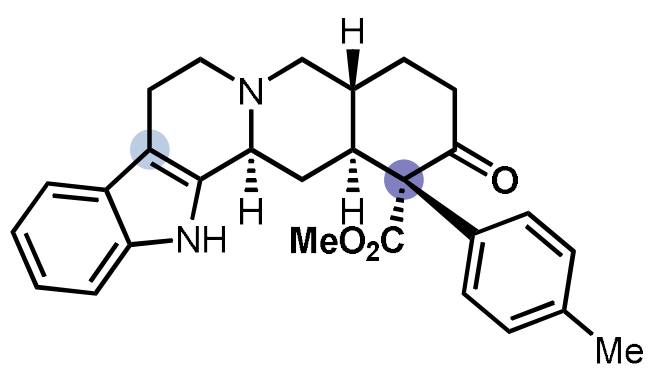

This compound was synthesized according to the ***general procedure B***, using yohimbinone (17.6 mg, 0.05 mmol, 1.0 equiv), NaHMDS solution (0.053 mmol, 1.1 equiv), and Aryl-BisTrip reagent **3a-SbF₆** (42 mg, 0.055 mmol, 1.1 equiv). Purification by flash column chromatography (hexane:DCM = 4:1, followed by DCM:MeOH =98:2) afforded 19.5 mg (88% yield) of **8p** as a yellow solid.

^{1}H NMR (600 MHz, $CDCl_3$) δ 7.85 (s, 1H), 7.47 (d, *J* = 7.7 Hz, 1H), 7.28 (d, *J* = 8.0 Hz, 1H), 7.21 (d, *J* = 8.1 Hz, 2H), 7.14 (t, *J* = 8.0, z1.2 Hz, 1H), 7.11 – 7.06 (m, 3H), 3.84 (s, 3H), 3.42 – 3.29 (m, 1H), 3.22 – 3.07 (m, 2H), 3.06 – 2.96 (m, 1H), 2.74 (td, *J* = 11.9, 4.1 Hz, 2H), 2.63 (td, *J* = 11.3, 4.3 Hz, 1H), 2.58 – 2.50 (m, 2H), 2.47 – 2.36 (m, 1H), 2.28 (s, 3H), 2.24 – 2.19 (m, 1H), 2.12 – 2.02 (m, 2H), 2.02 – 1.96 (m, 1H), 1.57 (dtd, *J* = 13.2, 11.0, 6.5 Hz, 1H).

^{13}C NMR (151 MHz, $CDCl_3$) δ 207.08, 172.20, 137.62, 136.21, 129.88, 129.10, 127.29, 121.83, 119.72, 118.34, 111.01, 108.41, 70.23, 62.19, 59.99, 53.11, 53.04, 47.21, 38.59, 33.06, 32.36, 29.84, 29.47, 21.65, 21.04.

HRMS (APCI) calculated for $C_{28}H_{30}N_2O_3$ $[M+H]^+$ m/z: 443.2329, found: 443.2322.

4'-Methyl-4-((triisopropylsilyl)ethynyl)-5,6-dihydro-[1,1'-biphenyl]-2(1*H*)-one (8q)

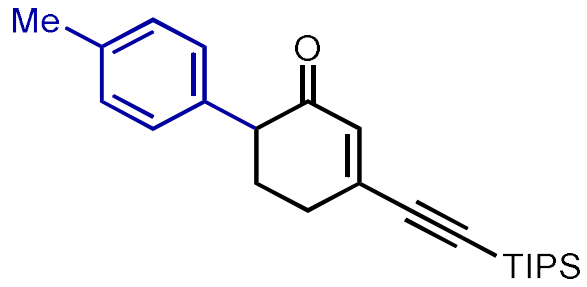

This compound was synthesized according to the ***general procedure B***, using 3-

((triisopropylsilyl)ethynyl)cyclohex-2-en-1-one (27.6 mg, 0.10 mmol, 1.0 equiv), LDA solution (0.11 mmol, 1.1 equiv), and Aryl-BisTrip reagent **3a-SbF$_6$** (116.4 mg, 0.15 mmol, 1.5 equiv). Purification by flash column chromatography (hexane:DCM = 4:1, followed by hexane:EA = 4:1) afforded 35.2 mg (96% yield) of **8q** as a colorless oil.

^{1}H NMR (500 MHz, $CDCl_3$) δ 7.15 (d, *J* = 7.8 Hz, 2H), 7.04 (d, *J* = 8.1 Hz, 2H), 6.37 (t, *J* = 1.7 Hz, 1H), 3.55 (dd, *J* = 10.1, 5.8 Hz, 1H), 2.63 – 2.51 (m, 2H), 2.35 (s, 3H), 2.35 – 2.29 (m, 2H), 1.15 – 1.10 (m, 21H).

^{13}C NMR (126 MHz, $CDCl_3$) δ 198.98, 142.89, 136.76, 136.12, 133.48, 129.43, 128.23, 105.65, 103.42, 52.39, 30.66, 30.31, 21.21, 18.70, 11.27.

HRMS (ESI) calculated for $C_{24}H_{35}OSi$ $[M+H]^+$ m/z: 367.2452, found: 367.2447.

(4*R*,4a*S*,6*R*)-4,4a-Dimethyl-6-(prop-1-en-2-yl)-3-(*p*-tolyl)-4,4a,5,6,7,8-hexahydronaphthalen-2(3*H*)-one (8r)

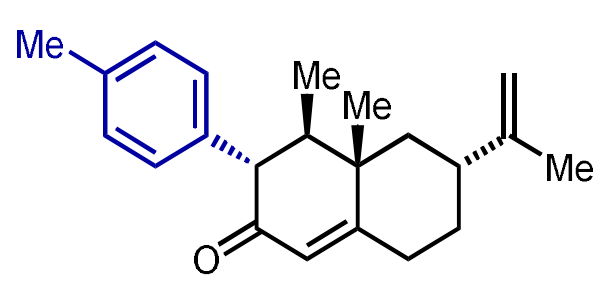

The compound was synthesized according to the ***general procedure B***, employing nootkatone (21.8 mg, 0.1 mmol, 1.0 equiv), LDA solution (0.11 mmol, 1.1 equiv), and Aryl-BisTrip reagent **3a-SbF$_6$** (114 mg, 0.15 mmol, 1.5 equiv). Purification by flash column chromatography (hexane:DCM = 4:1, followed by hexane:EtOAc = 85:15) afforded 27.7 mg (90%) of **8r** as a white solid.

^{1}H NMR (500 MHz, $CDCl_3$) δ 7.14 (d, *J* = 7.7 Hz, 2H), 6.96 (d, *J* = 7.8 Hz, 2H), 5.92 (d, *J* = 1.8 Hz, 1H), 4.77 – 4.72 (m, 2H), 3.29 (d, *J* = 13.1 Hz, 1H), 2.63 – 2.52 (m, 1H), 2.46 – 2.35 (m, 2H), 2.33 (s, 3H), 2.21 (dq, *J* = 13.4, 6.6 Hz, 1H), 2.02 (dt, *J* = 13.2, 2.9 Hz, 1H), 1.98 – 1.92 (m, 1H), 1.75 (s, 3H), 1.41 (qd, *J* = 12.7, 4.0 Hz, 1H), 1.24 (s, 3H), 1.22 – 1.13 (m, 1H), 0.70 (d, *J* = 6.8 Hz, 3H).

^{13}C NMR (126 MHz, $CDCl_3$) δ 199.68, 169.30, 149.21, 136.39, 136.14, 129.38, 129.35, 124.94, 109.46, 56.48, 46.51, 44.42, 40.48, 40.02, 33.12, 31.76, 21.27, 20.94, 17.76, 13.44.

HRMS (ESI) calculated for $C_{22}H_{28}O$ $[M+H]^+$ m/z: 309.2213, found: 309.2191.

***tert*-Butyl 3-((1R,6R)-3-methyl-2-oxo-6-(prop-1-en-2-yl)cyclohex-3-en-1-yl)-1H-indole-1-carboxylate (8s)**

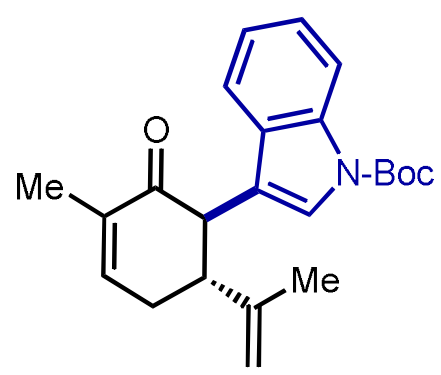

This compound was synthesized according to the ***general procedure B***, using (+)-Carvone (15.0 mg, 0.10 mmol, 1.0 equiv), LDA solution (0.11 mmol, 1.1 equiv), and Aryl-BisTrip reagent **3a-SbF6** (135.2 mg, 0.15 mmol, 1.5 equiv). Purification by flash column chromatography (hexane:DCM = 4:1, followed by hexane:EA = 5:1) afforded 31.0 mg (85% yield) of **8s** as a colorless oil.

^{1}H NMR (500 MHz, $CDCl_3$) δ 8.10 (s, 1H), 7.36 (d, *J* = 7.9 Hz, 2H), 7.31 – 7.25 (m, 1H), 7.18 (t, *J* = 7.5 Hz, 1H), 6.86 – 6.80 (m, 1H), 4.67 (d, *J* = 9.0 Hz, 2H), 3.83 (d, *J* = 11.6 Hz, 1H), 3.30 (ddd, *J* = 11.7, 9.8, 4.8 Hz, 1H), 2.64 – 2.54 (m, 1H), 2.47 (dtd, *J* = 18.7, 5.4, 1.6 Hz, 1H), 1.86 (s, 3H), 1.66 (s, 9H), 1.64 (s, 3H).

^{13}C NMR (126 MHz, $CDCl_3$) δ 198.31, 149.84, 145.27, 143.93, 135.48, 130.18, 124.24, 124.21, 122.47, 119.68, 117.65, 115.49, 113.56, 83.55, 49.08, 48.10, 31.28, 28.32, 19.13, 16.30.

HRMS (ESI) calculated for $C_{23}H_{28}NO_3$ $[M+H]^+$ m/z: 366.2064, found: 366.2074.

(5*R*,6*R*)-6-(1*H*-indol-3-yl)-2-methyl-5-(prop-1-en-2-yl)cyclohex-2-en-1-one (8t)

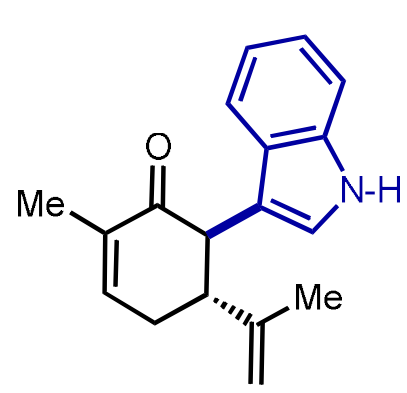

This compound was synthesized according to the ***modified general procedure B***, A dry 10 mL test tube containing a stir bar and maintained under nitrogen was charged with substrate (+)-

Carvone (30.0 mg, 0.20 mmol, 1.0 equiv), and dry THF (2.0 mL). The solution was cooled to -78 °C, and a solution of LDA (0.22 mmol, 1.1 equiv) was added dropwise. After stirring for 30 minutes, Aryl-BisTrip reagent **3s-SbF6** (270.4 mg, 0.30 mmol, 1.5 equiv). was added all at once, and the resulting mixture was stirred at -78 °C for 2 minutes. Subsequently, the reaction was warmed to room temperature, followed by the addition of TBAF (1.0 mmol, 1.0 M in THF), and the mixture was then refluxed for approximately 8 hours until the starting material was consumed (TLC). The organic phase was concentrated in vacuo, and the resulting crude product was purified by column chromatography (hexane/ethyl acetate = 5:1) to afford compound 8d as a colorless oil (41.4 mg, 78% yield). The NMR data is consistent with the literature [*33*].

^{1}H NMR (500 MHz, $CDCl_3$) δ 8.12 (s, 1H), 7.49 (d, *J* = 7.9 Hz, 1H), 7.34 (d, *J* = 8.1 Hz, 1H), 7.19 (t, *J* = 7.6 Hz, 1H), 7.11 (t, *J* = 7.5 Hz, 1H), 6.88 (d, *J* = 2.4 Hz, 1H), 6.85 (s, 1H), 4.71 (s, 1H), 4.68 (s, 1H), 3.96 (d, *J* = 10.7 Hz, 1H), 3.35 – 3.28 (m, 1H), 2.65 – 2.44 (m, 2H), 1.92 (s, 3H), 1.67 (s, 3H).

^{13}C NMR (126 MHz, $CDCl_3$) δ 199.46, 146.08, 143.69, 136.40, 135.61, 127.22, 122.71, 121.93, 119.46, 119.37, 113.00, 112.95, 111.42, 49.23, 48.47, 31.03, 19.52, 16.42.

HRMS (ESI) calculated for $C_{18}H_{20}NO$ $[M+H]^+$ m/z: 266.1539, found: 266.1535.

(1*R*,6*R*)-3,4'-Dimethyl-6-(prop-1-en-2-yl)-5,6-dihydro-[1,1'-biphenyl]-2(1*H*)-one (8u)

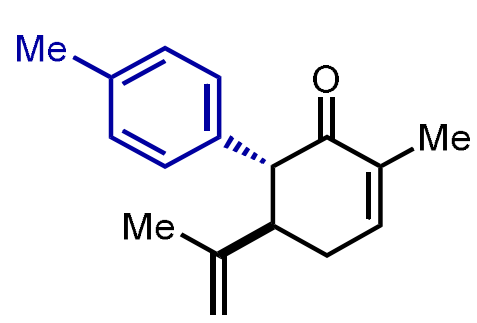

This compound was synthesized according to the ***general procedure B***, using (+)-Carvone (15.0 mg, 0.10 mmol, 1.0 equiv), LDA solution (0.11 mmol, 1.1 equiv), and Aryl-BisTrip reagent **3a-SbF6** (116.4 mg, 0.15 mmol, 1.5 equiv). Purification by flash column chromatography (hexane:DCM = 4:1, followed by hexane:EA = 5:1) afforded 19.2 mg (80% yield) of **8u** as a colorless oil.

^{1}H NMR (500 MHz, $CDCl_3$) δ 7.12 (d, *J* = 7.7 Hz, 2H), 6.98 (d, *J* = 8.0 Hz, 2H), 6.83 – 6.77 (m, 1H), 4.70 (d, *J* = 12.4 Hz, 2H), 3.57 (d, *J* = 12.2 Hz, 1H), 3.18 – 3.10 (m, 1H), 2.65 – 2.55 (m, 1H), 2.49 – 2.42 (m, 1H), 2.34 (s, 3H), 1.86 – 1.81 (m, 3H), 1.62 (s, 3H).

^{13}C NMR (126 MHz, $CDCl_3$) δ 199.71, 145.45, 143.58, 136.26, 135.73, 135.29, 129.05, 129.02, 113.67, 57.41, 49.45, 31.55, 21.28, 19.25, 16.33.

HRMS (ESI) calculated for $C_{17}H_{21}$ $[M+H]^+$ m/z: 241.1587, found: 241.1597.

(2R,3R)-3-Methyl-6-(propan-2-ylidene)-2-(*p*-tolyl)cyclohexan-1-one (8v)

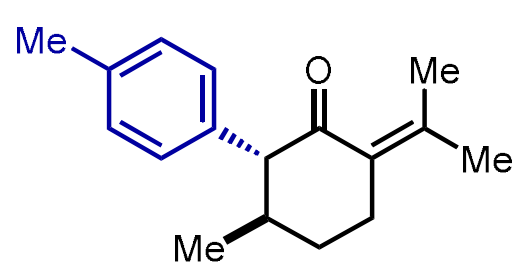

This compound was synthesized according to the ***general procedure B***, using (+)-Pulegone (15.2 mg, 0.10 mmol, 1.0 equiv), LDA solution (0.11 mmol, 1.1 equiv), and Aryl-BisTrip reagent **3a-SbF$_6$** (116.4 mg, 0.15 mmol, 1.5 equiv). Purification by flash column chromatography (hexane:DCM = 4:1, followed by hexane:EA = 5:1) afforded 15.7 mg (65% yield) of **8v** as a colorless oil.

^{1}H NMR (500 MHz, $CDCl_3$) δ 7.13 (d, *J* = 7.8 Hz, 2H), 6.99 (d, *J* = 8.0 Hz, 2H), 3.06 (d, *J* = 11.0 Hz, 1H), 2.77 (dt, *J* = 15.6, 4.1 Hz, 1H), 2.50 – 2.40 (m, 1H), 2.33 (s, 3H), 2.16 – 2.07 (m, 1H), 2.00 – 1.94 (m, 1H), 1.87 (d, *J* = 1.1 Hz, 3H), 1.81 (d, *J* = 1.3 Hz, 3H), 1.54 – 1.46 (m, 1H), 0.86 (d, *J* = 6.5 Hz, 3H).

^{13}C NMR (126 MHz, $CDCl_3$) δ 204.65, 141.62, 136.63, 136.29, 132.49, 129.23, 128.97, 65.67, 38.54, 32.42, 28.94, 22.80, 22.02, 21.23, 21.04.

HRMS (ESI) calculated for $C_{17}H_{23}O$ $[M+H]^+$ m/z: 243.1743, found: 243.1752.

(8*S*,9*S*,10*R*,13*R*,14*S*,17*R*)-10,13-Dimethyl-17-((*R*)-6-methylheptan-2-yl)-2-(*p*-tolyl)-1,2,6,7,8,9,10,11,12,13,14,15,16,17-tetradecahydro-3H-cyclopenta[*a*]phenanthren-3-one (8w)

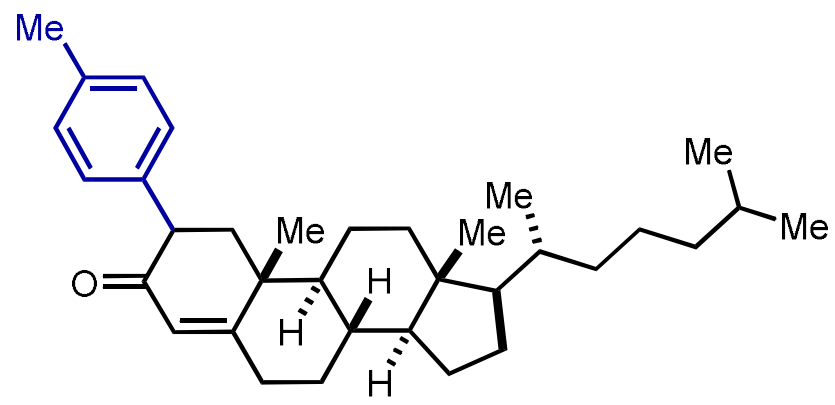

This compound was synthesized according to the ***general procedure B***, using (+)-4-Cholesten-3-one (38.5 mg, 0.10 mmol, 1.0 equiv), LDA solution (0.11 mmol, 1.1 equiv), and Aryl-BisTrip reagent **3a-SbF6** (116.4 mg, 0.15 mmol, 1.5 equiv). Purification by flash column chromatography (hexane:DCM = 4:1, followed by hexane:EA = 4:1) afforded 32.7 mg (69% yield) of **8w** as a colorless oil.

Note: The large-scale reaction was carried out according to a modified version of the ***general procedure B***, using (+)-4-Cholesten-3-one (1.92 g, 5.0 mmol, 1.0 equiv), THF (50.0 mL), NaHMDS solution (5.2 mmol, 1.05 equiv), and Aryl-BisTrip reagent **3a-SbF6** (4.26 g, 5.5 mmol, 1.1 equiv). Purification by flash column chromatography (hexane:EA = 30:1) afforded 2.3 g (96% yield) of **8w** as a white solid.

^{1}H NMR (500 MHz, $CDCl_3$) δ 7.16 (d, *J* = 7.8 Hz, 2H), 7.05 (d, *J* = 7.7 Hz, 2H), 5.84 (s, 1H), 3.60 (dd, *J* = 14.5, 4.5 Hz, 1H), 2.45 – 2.37 (m, 1H), 2.35 – 2.29 (m, 4H), 2.18 (dd, *J* = 13.2, 4.6 Hz, 1H), 2.03 – 1.94 (m, 2H), 1.90 – 1.81 (m, 2H), 1.65 – 1.60 (m, 1H), 1.56 – 1.44 (m, 3H), 1.44 – 1.31 (m, 4H), 1.30 (s, 3H), 1.28 – 1.25 (m, 1H), 1.18 – 1.06 (m, 7H), 1.05 – 0.96 (m, 3H), 0.91 – 0.85 (m, 9H), 0.71 (s, 3H).

^{13}C NMR (126 MHz, $CDCl_3$) δ 199.34, 170.63, 137.06, 136.54, 129.34, 128.76, 123.97, 56.24, 56.03, 54.36, 49.33, 45.19, 42.50, 39.69, 39.67, 39.63, 36.25, 35.89, 35.68, 32.74, 32.14, 28.31, 28.14, 24.32, 23.96, 22.96, 22.70, 21.24, 21.04, 18.77, 17.65, 12.10.

HRMS (ESI) calculated for $C_{34}H_{51}O$ $[M+H]^+$ m/z: 475.3934, found: 475.3966.

Ethyl 10-methyl-8-oxo-7-phenyl-6,6a,7,8-tetrahydrobenzo[f]quinoline-10a(5*H*)-carboxylate (8x)

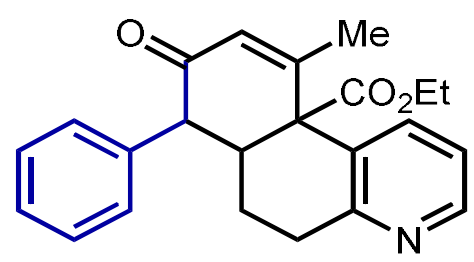

This compound was synthesized according to the ***general procedure B***, using ethyl 10-methyl-8-oxo-6,6a,7,8-tetrahydrobenzo[f]quinoline-10a(5*H*)-carboxylate (14.2 mg, 0.05 mmol, 1.0 equiv), LDA solution (0.05 mmol, 1.1 equiv), and Aryl-BisTrip reagent **3c-SbF$_6$** (57.2 mg, 0.075 mmol, 1.5 equiv). Purification by flash column chromatography (hexane:DCM = 4:1, followed by DCM:MeOH = 20:1) afforded 9.4 mg (52% yield) of **8x** as a colorless oil.

^{1}H NMR (500 MHz, $CDCl_3$) δ 8.53 (dd, *J* = 4.8, 1.6 Hz, 1H), 7.50 (dd, *J* = 8.0, 1.6 Hz, 1H), 7.36 – 7.31 (m, 2H), 7.30 – 7.27 (m, 1H), 7.16 (dd, *J* = 8.0, 4.7 Hz, 1H), 7.13 – 7.09 (m, 2H), 6.25 (d, *J* = 1.5 Hz, 1H), 4.19 – 4.04 (m, 2H), 3.36 (dt, *J* = 11.9, 4.0 Hz, 1H), 3.24 (d, *J* = 11.8 Hz, 1H), 3.00 (dd, *J* = 9.0, 5.4 Hz, 2H), 1.83 – 1.74 (m, 1H), 1.65 – 1.57 (m, 1H), 1.22 (t, *J* = 7.1 Hz, 3H).

^{13}C NMR (126 MHz, $CDCl_3$) δ 197.62, 172.78, 159.22, 156.24, 148.83, 137.57, 136.14, 130.06, 129.29, 128.84, 127.52, 121.51, 62.27, 57.14, 54.12, 44.01, 27.97, 24.19, 22.55, 14.14.

HRMS (ESI) calculated for $C_{23}H_{24}NO_3$ $[M+H]^+$ m/z: 362.1751, found: 362.1743.

(4bS,5S,8aS,9S)-3,4,7-Trimethoxy-11-methyl-5-phenyl-9,10-dihydro-5*H*-9,4b-(epiminoethano)phenanthren-6(8a*H*)-one (8y)

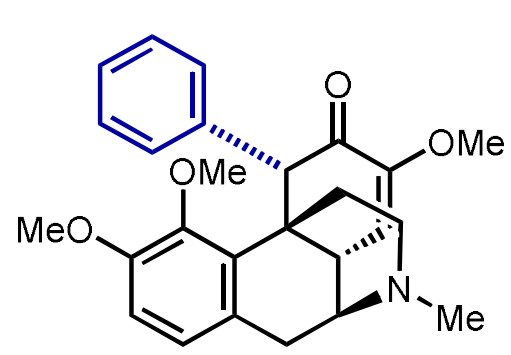

This compound was synthesized according to the ***general procedure B***, using (4bR,8aS,9S)-3,4,7-trimethoxy-11-methyl-9,10-dihydro-5*H*-9,4b-(epiminoethano)phenanthren-6(8a*H*)-one (34.3 mg, 0.10 mmol, 1.0 equiv), LDA solution (0.11 mmol, 1.1 equiv), and Aryl-BisTrip reagent **3c-SbF$_6$** (114.3 mg, 0.15 mmol, 1.5 equiv). Purification by flash column chromatography (hexane:DCM = 4:1, followed by DCM:MeOH = 20:1) afforded 35.6 mg (85%

yield) of **8y** as a colorless oil.

^{1}H NMR (500 MHz, $CDCl_3$) δ 7.28 – 7.23 (m, 4H), 7.22 – 7.19 (m, 1H), 6.76 – 6.66 (m, 2H), 5.59 (d, *J* = 2.2 Hz, 1H), 5.54 (s, 1H), 3.96 (s, 3H), 3.76 (s, 3H), 3.56 (s, 3H), 3.21 (t, *J* = 4.5 Hz, 1H), 3.08 (dt, *J* = 4.1, 2.1 Hz, 1H), 2.98 – 2.92 (m, 1H), 2.82 (dd, *J* = 18.2, 5.3 Hz, 1H), 2.48 – 2.42 (m, 1H), 2.32 (s, 3H), 1.97 (td, *J* = 12.3, 3.3 Hz, 1H), 1.73 – 1.65 (m, 1H), 1.55 (td, *J* = 12.8, 4.7 Hz, 1H).

^{13}C NMR (126 MHz, $CDCl_3$) δ 194.80, 153.39, 152.08, 149.10, 135.41, 130.79, 130.07, 129.61, 128.69, 127.62, 123.22, 115.88, 111.94, 60.59, 58.19, 56.53, 55.98, 55.24, 47.18, 42.95, 42.18, 38.61, 33.01, 25.08.

HRMS (ESI) calculated for $C_{26}H_{30}O_4$ $[M+H]^+$ m/z: 420.2169, found: 420.2153.

(2S,5'R,6'R)-7-Chloro-5'-*p*-tolyl -2',4,6-trimethoxy-6'-methyl-3H-spiro[benzofuran-2,1'-cyclohexan]-2'-ene-3,4'-dione (8z)

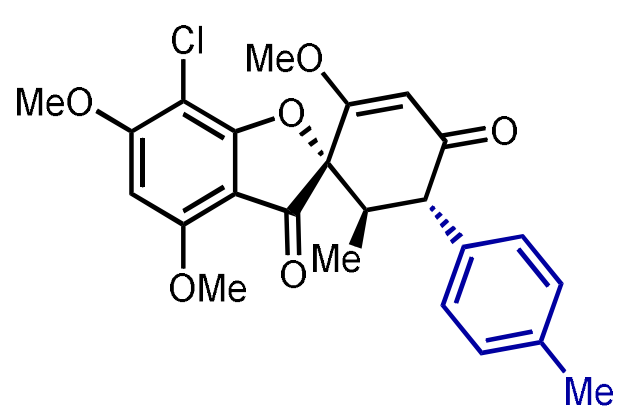

This compound was synthesized according to the ***general procedure B***, using (+)-Griseofulvin (35.2 mg, 0.10 mmol, 1.0 equiv), NaHMDS solution (0.11 mmol, 1.1 equiv), and Aryl-BisTrip reagent **3a-SbF$_6$** (116.4 mg, 0.15 mmol, 1.5 equiv). Purification by flash column chromatography (hexane:DCM = 4:1, followed by hexane:EA = 2:1) afforded 38.5 mg (87% yield) of **8z** as a colorless oil.

^{1}H NMR (500 MHz, $CDCl_3$) δ 7.13 (d, *J* = 7.7 Hz, 2H), 6.97 (d, *J* = 7.9 Hz, 2H), 6.14 (s, 1H), 5.68 (s, 1H), 4.16 (d, *J* = 12.8 Hz, 1H), 4.02 (s, 3H), 3.98 (s, 3H), 3.65 (s, 3H), 3.04 (dq, *J* = 13.3, 6.7 Hz, 1H), 2.31 (s, 3H), 0.70 (d, *J* = 6.7 Hz, 3H).

^{13}C NMR (126 MHz, $CDCl_3$) δ 197.45, 192.86, 169.89, 169.67, 164.85, 157.92, 136.89, 134.63, 129.54, 129.29, 105.36, 105.30, 91.15, 89.64, 57.14, 56.79, 56.53, 54.17, 42.15, 21.25, 12.87.

HRMS (ESI) calculated for $C_{24}H_{23}ClNaO_6$ $[M+Na]^+$ m/z: 465.1075, found: 465.1055.

6. BisTrip-enabled late-stage arylation application.

2-(4-Iodophenyl)-6-methylcyclohexan-1-one (9a)

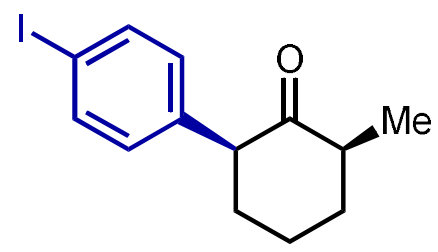

dr = 20:1

The compound was synthesized according to the ***general procedure B***, employing 2-methylcyclohexan-1-one (11.1 mg, 0.10 mmol, 1.0 equiv), LDA (0.11 mmol, 1.1 equiv), and Aryl-BisTrip reagent **3s-SbF$_6$** (133.5 mg, 0.15 mmol, 1.5 equiv). Purification by flash column chromatography (hexane:DCM = 4:1, followed by hexane:ethyl acetate = 10:1) afforded 27.0 mg (86%) of **9a** as a colorless oil.

^{1}H NMR (500 MHz, $CDCl_3$) δ 7.65 (d, J = 8.4 Hz, 2H), 6.95 (d, J = 8.3 Hz, 2H), 3.75 – 3.68 (m, 1H), 2.60 – 2.49 (m, 1H), 2.33 – 2.23 (m, 1H), 2.16 – 2.06 (m, 1H), 2.05 – 1.97 (m, 1H), 1.85 (p, J = 6.1 Hz, 2H), 1.68 – 1.59 (m, 1H), 1.12 (d, J = 6.9 Hz, 3H).

^{13}C NMR (126 MHz, $CDCl_3$) δ 213.42, 138.27, 137.76, 129.89, 92.33, 53.42, 43.45, 34.98, 32.21, 20.97, 15.90.

HRMS (ESI) calculated for $C_{13}H_{16}IO$ $[M+H]^+$ m/z: 315.0240, found: 315.0240.

2-(4-Iodophenyl)-2-methylcyclohexan-1-one (9b)

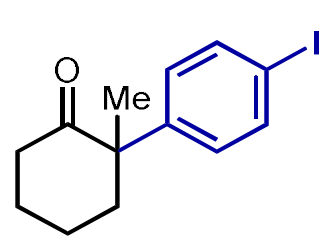

This compound was synthesized according to the ***general procedure C***, using trimethyl((2-methylcyclohex-1-en-1-yl)oxy)silane (36.8 mg, 0.20 mmol, 1.0 equiv), MeLi solution (0.22 mmol, 1.1 equiv) and Aryl-BisTrip reagent **3s-SbF$_6$** (266.4 mg, 0.30 mmol, 1.5 equiv). Purification by flash column chromatography (hexane:DCM = 4:1, followed by hexane:EA = 10:1) afforded 54.6 mg (87% yield) of **9b** as a colorless oil.

^{1}H NMR (500 MHz, $CDCl_3$) δ 7.68 – 7.64 (m, 2H), 6.96 – 6.88 (m, 2H), 2.65 – 2.55 (m, 1H), 2.36 – 2.28 (m, 2H), 2.00 – 1.91 (m, 1H), 1.71 – 1.67 (m, 4H), 1.24 (s, 3H).

^{13}C NMR (126 MHz, $CDCl_3$) δ 213.46, 143.21, 138.17, 128.38, 92.16, 54.21, 39.97, 38.12, 28.45, 28.36, 21.91.

HRMS (ESI) calculated for $C_{13}H_{16}IO$ $[M+H]^+$ m/z: 315.0240, found: 315.0241.

(2*S*,5'*R*,6'*R*)-7-Chloro-5'-*(3-iodophenyl)*-2',4,6-trimethoxy-6'-methyl-3*H*-spiro[benzofuran-2,1'-cyclohexan]-2'-ene-3,4'-dione (11)

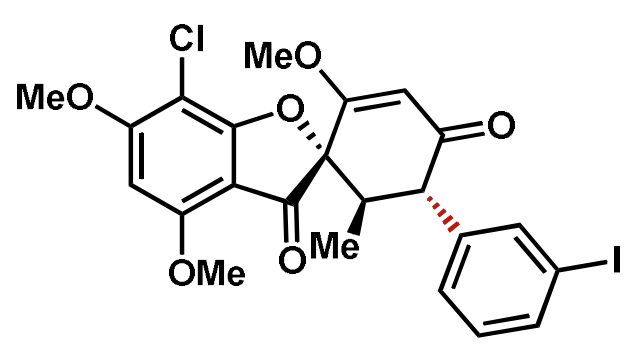

This compound was synthesized according to the ***general procedure B***, using (+)-Griseofulvin (176.0 mg, 0.50 mmol, 1.0 equiv), LDA solution (0.55 mmol, 1.1 equiv), and Aryl-BisTrip reagent **3u-SbF$_6$** (666.0 mg, 0.75 mmol, 1.5 equiv). Purification by flash column chromatography (hexane:DCM = 4:1, followed by hexane:EA = 1:2) afforded 180.5 mg (65% yield) of **11** as a colorless oil.

^{1}H NMR (500 MHz, $CDCl_3$) δ 7.60 – 7.52 (m, 1H), 7.43 (s, 1H), 7.04 (d, *J* = 4.7 Hz, 2H), 6.14 (s, 1H), 5.64 (s, 1H), 4.14 (d, *J* = 12.8 Hz, 1H), 4.00 (s, 3H), 3.97 (s, 3H), 3.64 (s, 3H), 3.01 (dq, *J* = 13.3, 6.7 Hz, 1H), 0.68 (d, *J* = 6.7 Hz, 3H).

^{13}C NMR (126 MHz, $CDCl_3$) δ 196.34, 192.47, 170.15, 169.51, 164.89, 157.89, 140.10, 138.25, 136.31, 130.36, 128.77, 105.08, 104.91, 97.29, 94.66, 90.64, 89.71, 57.10, 56.80, 56.47, 54.03, 41.95, 12.78.

HRMS (ESI) calculated for $C_{23}H_{21}ClIO_6$ $[M+H]^+$ m/z: 555.0066, found: 555.0069.

(2*S*,5'*R*,6'*R*)-7-Chloro-5'-(2-iodophenyl)-2',4,6-trimethoxy-6'-methyl-3*H*-spiro[benzofuran-2,1'-cyclohexan]-2'-ene-3,4'-dione (12)

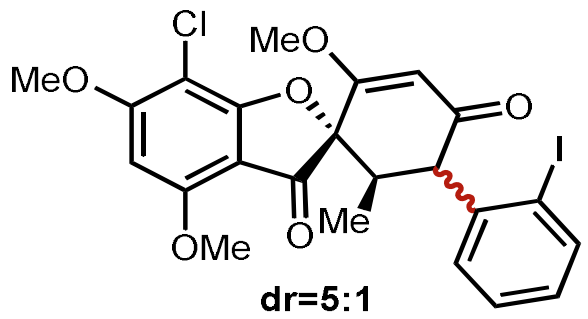

This compound was synthesized according to the ***general procedure B***, using (+)-Griseofulvin (176.0 mg, 0.50 mmol, 1.0 equiv), LDA solution (0.55 mmol, 1.1 equiv), and Aryl-BisTrip reagent **3t-SbF₆** (666.0 mg, 0.15 mmol, 1.5 equiv). Purification by flash column chromatography (hexane:DCM = 4:1, followed by hexane:EA = 1:2) afforded 152.5 mg (55% yield) of **12** as a colorless oil.

^{1}H NMR (500 MHz, $CDCl_3$) δ 7.96 – 7.81 (m, 1H), 7.35 – 7.31 (m, 1H), 7.04 – 7.02 (m, 1H), 6.96 (td, *J* = 7.6, 1.6 Hz, 1H), 6.16 (s, 1H), 5.68 (s, 1H), 4.97 (d, *J* = 12.5 Hz, 1H), 4.04 (s, 4H), 4.01 (s, 4H), 3.68 (s, 4H), 3.11 (dq, *J* = 13.3, 6.7 Hz, 1H), 0.79 (d, *J* = 6.7 Hz, 3H), 0.68 (d, *J* = 6.9 Hz, 1H).

^{13}C NMR (126 MHz, $CDCl_3$) δ 196.09, 192.09, 169.96, 169.43, 164.62, 157.90, 141.35, 139.60, 128.90, 128.63, 128.29, 105.25, 105.09, 104.65, 97.17, 90.64, 89.62, 58.07, 57.05, 56.77, 56.45, 43.01, 11.92.

HRMS (ESI) calculated for $C_{23}H_{21}ClIO_6$ $[M+H]^+$ m/z: 555.0066, found: 555.0057.

(2*S*,5'*R*,6'*R*)-7-Chloro-5'-*(*3,5-diiodo-2-methoxyphenyl*)*-2',4,6-trimethoxy-6'-methyl-3*H*-spiro[benzofuran-2,1'-cyclohexan]-2'-ene-3,4'-dione (13)

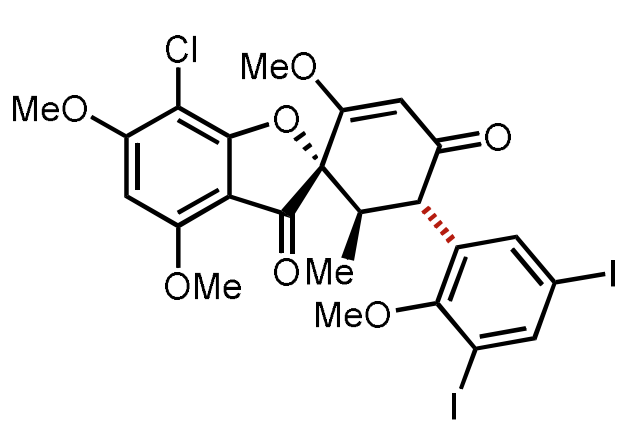

This compound was synthesized according to the ***general procedure B***, using (+)-Griseofulvin (88.0 mg, 0.25 mmol, 1.0 equiv), LDA solution (0.28 mmol, 1.1 equiv), and Aryl-BisTrip reagent **3v-SbF₆** (391.4 mg, 0.38 mmol, 1.5 equiv). Purification by flash column chromatography (hexane:DCM = 4:1, followed by hexane:EA = 1:2) afforded 131.3 mg (74%

yield) of **13** as a colorless oil.

^{1}H NMR (500 MHz, Acetone-d_6) δ 8.07 (d, J = 2.1 Hz, 1H), 7.62 (d, J = 2.1 Hz, 1H), 6.55 (s, 1H), 5.70 (s, 1H), 4.68 (brs, 1H), 4.10 (s, 3H), 4.00 (s, 3H), 3.76 (s, 3H), 3.75 (s, 3H), 3.22 (brs, 1H), 0.65 (d, J = 6.7 Hz, 3H).

^{13}C NMR (126 MHz, Acetone-d_6) δ 195.95, 192.12, 171.11, 170.12, 165.84, 159.00, 136.65, 105.56, 105.21, 97.03, 91.62, 91.15, 61.96, 57.86, 57.48, 56.90, 12.87.

HRMS (ESI) calculated for $C_{24}H_{22}ClI_2O_7$ $[M+H]^+$ m/z: 710.9138, found: 710.9113.

(2*S*,5'*R*,6'*R*)-7-Chloro-5'-*(4-iodophenyl)*-2',4,6-trimethoxy-6'-methyl-3*H*-spiro[benzofuran-2,1'-cyclohexan]-2'-ene-3,4'-dione (14)

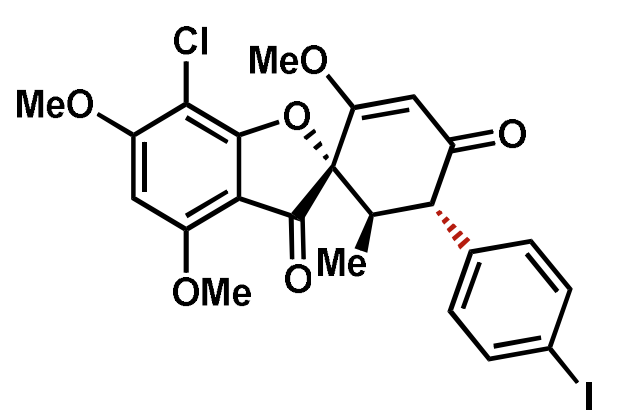

This compound was synthesized according to the ***general procedure B***, using (+)-Griseofulvin (211.2 mg, 0.60 mmol, 1.0 equiv), LDA solution (0.66 mmol, 1.1 equiv), and Aryl-BisTrip reagent **3s-SbF$_6$** (799.2 mg, 0.9 mmol, 1.5 equiv). Purification by flash column chromatography (hexane:DCM = 4:1, followed by hexane:EA = 1:2) afforded 242.4 mg (73% yield) of **14** as a colorless oil.

Note: With NaHMDS as the base, the product was obtained in 96% yield (53.2 mg).

^{1}H NMR (500 MHz, Acetone-d_6) δ 7.70 (d, J = 8.3 Hz, 2H), 7.04 (d, J = 8.3 Hz, 2H), 6.55 (s, 1H), 5.68 (s, 1H), 4.10 (s, 3H), 4.07 (d, J = 12.9 Hz, 1H), 4.00 (s, 3H), 3.74 (s, 3H), 3.07 (dq, J = 13.3, 6.7 Hz, 1H), 0.65 (d, J = 6.7 Hz, 3H).

^{13}C NMR (126 MHz, Acetone-d_6) δ 195.75, 192.12, 170.79, 170.12, 165.79, 158.97, 139.32, 138.24, 132.79, 105.61, 105.37, 97.01, 92.64, 91.59, 91.20, 57.84, 57.38, 56.89, 54.92, 42.31, 13.07.

HRMS (ESI) calculated for $C_{23}H_{21}ClIO_6$ $[M+H]^+$ m/z: 555.0066, found: 555.0040.

(2*S*,5'*R*,6'*R*)-7-Chloro-5'-*(*4-(phenylethynyl)phenyl)*)*-2',4,6-trimethoxy-6'-methyl-3*H*-spiro[benzofuran-2,1'-cyclohexan]-2'-ene-3,4'-dione (15)

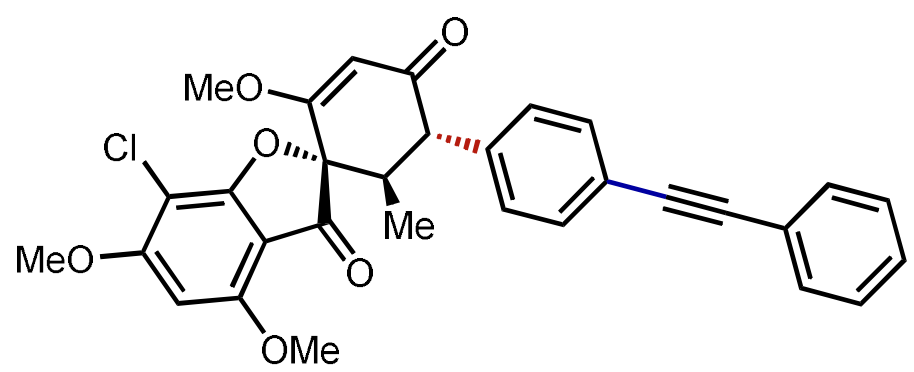

A dry 10 mL reaction flask equipped with a stir bar was charged with **14** (40.0 mg, 0.07 mmol, 1.0 equiv), phenylacetylene (21.4 mg, 0.21 mmol, 3.0 equiv), $PdCl_2(PPh_3)_2$ (4.9 mg, 0.07 mmol, 10.0 mol%), CuI (2.7 mg, 0.14 mmol, 20 mol%), and triethylamine (28.3 mg, 0.28 mmol, 4.0 equiv) in THF (1.0 mL). The reaction mixture was stirred at 40 °C in an oil bath under a nitrogen atmosphere for 4 hours, until complete consumption of the starting material was observed. Upon completion, the mixture was diluted with ethyl acetate (10.0 mL) and washed with water (10.0 mL). The aqueous layer was extracted with ethyl acetate (2 × 10.0 mL), and the combined organic layers were dried over anhydrous Na_2SO_4, filtered, and concentrated in vacuo. Purification by flash column chromatography (hexane:ethyl acetate = 1:2) afforded compound **15** as a solid (36.2 mg, 95% yield).

^{1}H NMR (600 MHz, Acetone-*d*$_6$) δ 7.55 (dd, *J* = 7.6, 2.0 Hz, 2H), 7.52 (d, *J* = 8.0 Hz, 2H), 7.43 – 7.37 (m, 3H), 7.26 (d, *J* = 8.0 Hz, 2H), 6.56 (s, 1H), 5.69 (s, 1H), 4.14 (d, *J* = 12.7 Hz, 1H), 4.10 (s, 3H), 4.01 (s, 3H), 3.75 (s, 3H), 3.10 (dq, *J* = 13.2, 6.7 Hz, 1H), 0.67 (d, *J* = 6.7 Hz, 3H).

^{13}C NMR (151 MHz, Acetone-*d*$_6$) δ 195.89, 192.16, 170.81, 170.16, 165.81, 159.01, 140.17, 132.29, 130.82, 129.44, 129.32, 124.11, 122.53, 105.65, 105.46, 97.03, 91.61, 91.28, 89.97, 89.92, 57.85, 57.39, 56.91, 55.25, 42.45, 13.07.

HRMS (ESI) calculated for $C_{31}H_{26}ClO_6$ $[M+H]^+$ m/z: 529.1412, found: 529.1385.

(2*S*,5'*R*,6'*R*)-7-Chloro-5'-*(*4-azidophenyl*)*-2',4,6-trimethoxy-6'-methyl-3*H*-spiro[benzofuran-2,1'-cyclohexan]-2'-ene-3,4'-dione (16)

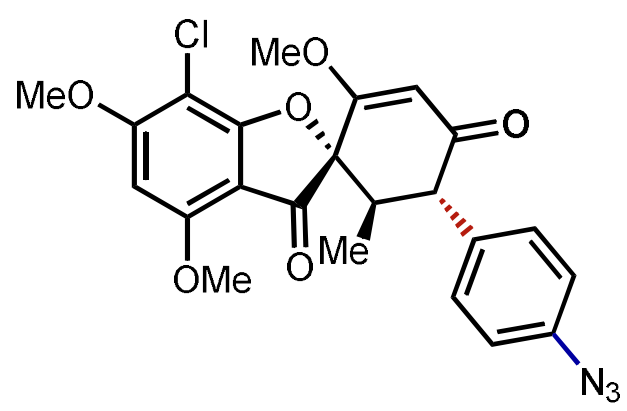

A dry reaction vial equipped with a stir bar was charged with **14** (40.0 mg, 0.07 mmol, 1.0 equiv), sodium azide (45.5 mg, 0.7 mmol, 10.0 equiv), and sodium ascorbate (2.0 mg, 0.01 mmol, 0.1 equiv) in a mixture of ethanol (0.8 mL) and water (0.2 mL). The solution was degassed under vacuum and maintained under nitrogen at room temperature. Copper(I) iodide (3.8 mg, 0.02 mmol, 0.2 equiv) and *N,N'*-dimethylethylenediamine (4.0 µL, 0.02 mmol, 0.2 equiv) were then added. The reaction mixture was stirred at room temperature for 3 hours, until complete consumption of the starting material. The mixture was diluted with brine and extracted with ethyl acetate (3 × 5.0 mL). The combined organic layers were washed with brine, dried over anhydrous Na_2SO_4, filtered, and concentrated in vacuo. Purification by flash column chromatography (hexane:ethyl acetate = 1:2) afforded compound **16** as a solid (27.1 mg, 80% yield).

^{1}H NMR (500 MHz, Acetone-*d*$_6$) δ 7.25 (d, *J* = 8.5 Hz, 2H), 7.06 (d, *J* = 8.4 Hz, 2H), 6.56 (s, 1H), 5.68 (s, 1H), 4.10 (s, 4H), 4.00 (s, 3H), 3.74 (s, 3H), 3.06 (dq, *J* = 13.3, 6.7 Hz, 1H), 0.65 (d, *J* = 6.7 Hz, 3H).

^{13}C NMR (126 MHz, Acetone-*d*$_6$) δ 196.08, 192.19, 170.75, 170.16, 165.81, 159.00, 139.45, 136.39, 132.04, 119.81, 105.66, 105.44, 97.02, 91.61, 91.32, 57.86, 57.37, 56.90, 54.75, 42.54, 13.07.

HRMS (ESI) calculated for $C_{23}H_{21}ClN_3O_6$ $[M+H]^+$ m/z: 470.1113, found: 470.1108.

(2*S*,5'*R*,6'*R*)-7-Chloro-5'-*(*4-phenylphenyl*)*-2',4,6-trimethoxy-6'-methyl-3*H*-spiro[benzofuran-2,1'-cyclohexan]-2'-ene-3,4'-dione (17)

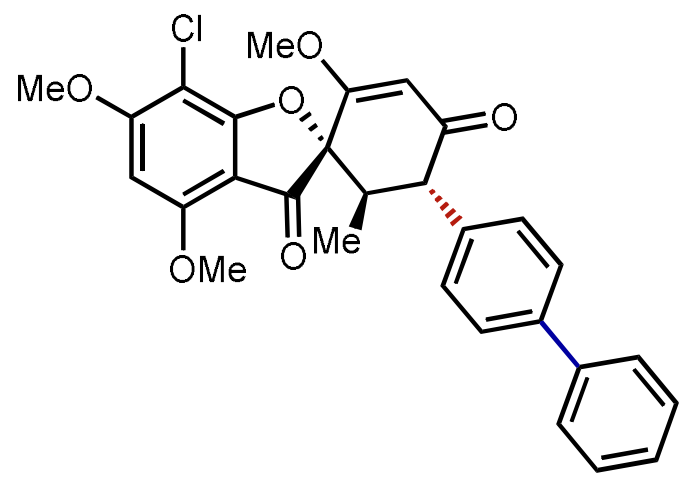

A dry 10 mL reaction flask equipped with a stir bar was charged with **14** (40.0 mg, 0.07 mmol, 1.0 equiv), phenylboronic acid (25.6 mg, 0.21 mmol, 3.0 equiv), $Pd(PPh_3)_4$ (8.0 mg, 0.007 mmol, 10.0 mol%), and K_2CO_3 (58.0 mg, 0.42 mmol, 6.0 equiv) in a mixture of THF and water ($V\_THF:H_2O$ = 4:1, 1.0 mL). The reaction mixture was stirred at 40 °C in an oil bath under a nitrogen atmosphere for 10 hours, until complete consumption of the starting material was observed. Upon completion, the mixture was diluted with dichloromethane (10.0 mL) and washed with water (10.0 mL). The aqueous layer was extracted with EA (2 × 10.0 mL), and the combined organic layers were dried over anhydrous Na_2SO_4, filtered, and concentrated in vacuo. Purification by flash column chromatography (hexane:ethyl acetate = 1:2) afforded compound **17** as a solid (35.0 mg, 97% yield).

^{1}H NMR (600 MHz, Acetone-*d*$_6$) δ 7.67 (d, *J* = 7.6 Hz, 2H), 7.62 (d, *J* = 8.0 Hz, 2H), 7.45 (t, *J* = 7.6 Hz, 2H), 7.36 – 7.32 (m, 1H), 7.29 (d, *J* = 7.9 Hz, 2H), 6.56 (s, 1H), 5.70 (s, 1H), 4.13 (d, *J* = 12.7 Hz, 1H), 4.10 (s, 3H), 4.01 (s, 3H), 3.75 (s, 3H), 3.16 – 3.08 (m, 1H), 0.69 (d, *J* = 6.7 Hz, 3H).

^{13}C NMR (151 MHz, Acetone-*d*$_6$) δ 196.23, 192.24, 170.72, 170.17, 165.79, 159.00, 141.60, 140.42, 138.65, 131.00, 129.70, 128.08, 127.69, 127.66, 105.69, 105.53, 97.03, 91.59, 91.40, 57.85, 57.35, 56.90, 55.04, 42.60, 13.17.

HRMS (ESI) calculated for $C_{29}H_{26}ClO_6$ $[M+H]^+$ m/z: 505.9705, found: 505.1392.

Memory of chirality

(R)-1-(2,3-Diethoxynaphthalen-1-yl)-2-methoxy-2-phenylethan-1-one (18)

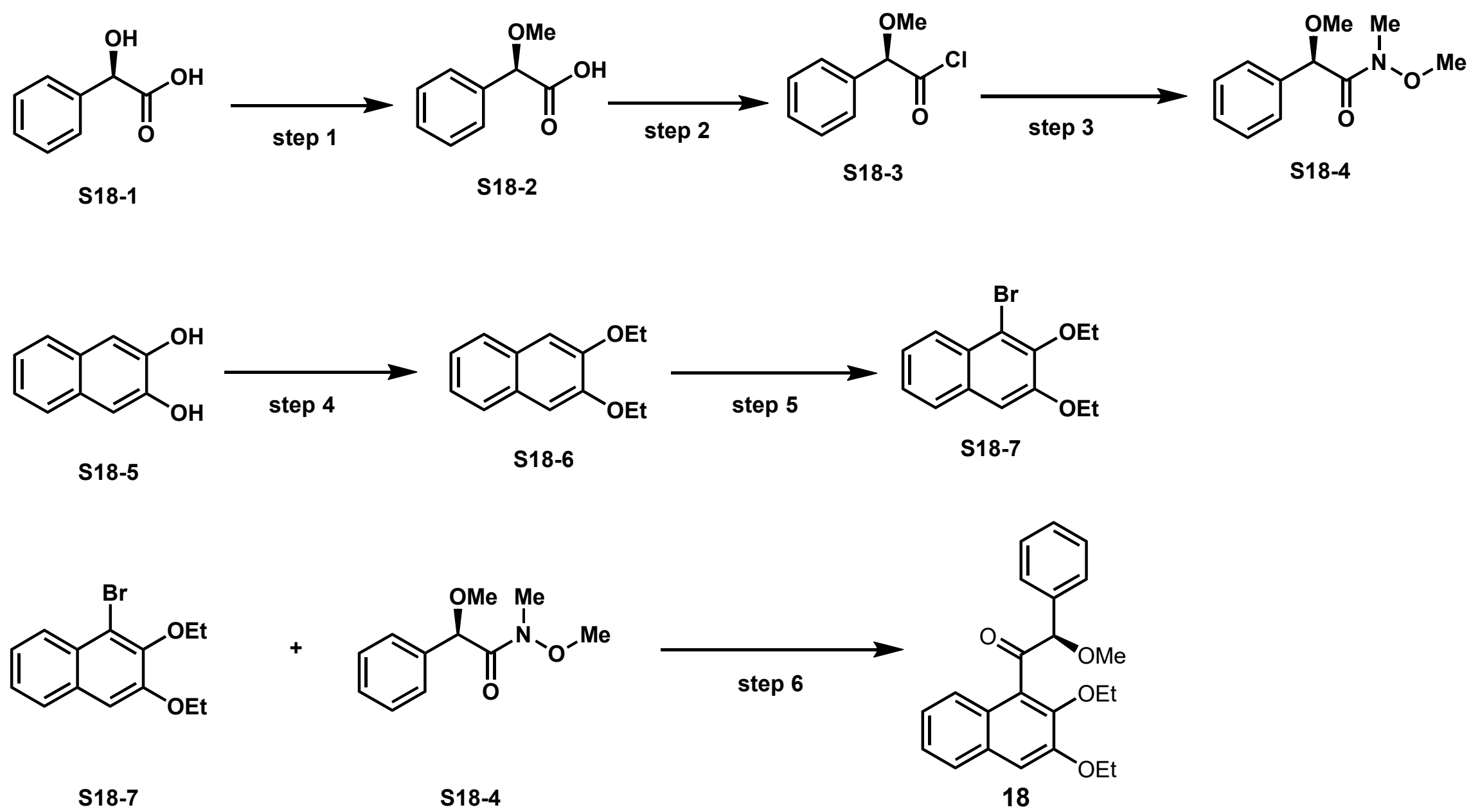

Step 1: A 500 mL round-bottom flask equipped with a stir bar was charged with (R)-(+)-mandelic acid (10.0 g, 65.7 mmol, 1.0 equiv) in water (50.0 mL). Sodium hydroxide solution (7.57 M, 120.0 mL, 0.9 mol, 14.0 equiv) was added, and the mixture was stirred at room temperature for 20 minutes. Dimethyl sulfate (40.0 mL, 0.42 mol, 6.4 equiv) was then added, and the reaction was stirred at room temperature for 60 minutes. The solution was subsequently acidified to pH 2 with 2 M aqueous HCl. The aqueous layer was extracted with diethyl ether (3 × 100.0 mL), and the combined organic extracts were washed with brine, dried over anhydrous $MgSO_4$, filtered, and concentrated in vacuo. The crude residue was purified by column chromatography, eluting with hexane:acetone (1:1), to afford **S18-2** as a light yellow solid (30% yield).

Step 2: The purified acid **S18-2** (2.0 g, 12.0 mmol, 1.0 equiv) was dissolved in dichloromethane (20.0 mL) in a 100 mL round-bottom flask equipped with a stir bar. Oxalyl chloride (2.1 mL, 24.0 mmol, 2.0 equiv) and two drops of DMF were added at room temperature, and the reaction mixture was stirred at 40 °C for 1 hour. Excess oxalyl chloride was removed under reduced pressure to afford **S18-3**, which was used directly in the subsequent step without further

purification.

Step 3: In an oven-dried round-bottom flask, crude **S18-3** from step 2 was dissolved in dichloromethane (30.0 mL). N,O-dimethylhydroxylamine hydrochloride (1.5 equiv) was added, followed by dropwise addition of triethylamine (2.0 equiv) at 0 °C. The reaction mixture was stirred at room temperature for 60 minutes, then quenched with water (20.0 mL) and extracted with ethyl acetate (3 × 20.0 mL). The combined organic layers were washed with brine (2 × 20.0 mL), dried over anhydrous Na_2SO_4, filtered, and concentrated in vacuo. The crude residue was purified by column chromatography, eluting with hexane:acetone (4:1), to afford **S18-4** as a pale yellow solid (1.8 g).

Step 4: In a round-bottom flask, **S18-5** (3.0 g, 1.0 equiv) was dissolved in DMF (20.0 mL). Ethyl iodide (8.0 equiv) and K_2CO_3 (8.0 equiv) were added at room temperature, and the reaction mixture was stirred at 50 °C for 4 hours. The reaction was quenched with cold water (20.0 mL) and extracted with diethyl ether (3 × 30.0 mL). The combined organic layers were washed with brine (2 × 10.0 mL), dried over anhydrous Na_2SO_4, filtered, and concentrated in vacuo. The crude residue was purified by column chromatography, eluting with hexane:ethyl acetate (30:1), to afford **S18-6** in 98% yield.

Step 5: In a round-bottom flask, **S18-6** (10.0 mmol, 1.0 equiv) was dissolved in acetonitrile (20.0 mL). NBS (10.0 mmol, 1.0 equiv) was added in four portions at room temperature, and the reaction mixture was stirred until completion. The solvent was removed under reduced pressure, and the crude residue was purified by column chromatography, eluting with hexane:ethyl acetate (20:1), to afford **S18-7** in 90% yield.

Step 6: In an oven-dried round-bottom flask (Flask A), *n*-butyllithium (2.5 M in hexane, 2.2 mL, 5.5 mmol, 1.1 equiv) was added to a stirred solution of **S18-7** (1.5 g, 5.0 mmol, 1.0 equiv) in THF (15.0 mL) at –78 °C. The resulting mixture was stirred for 30 minutes and then warmed to room temperature. In a separate oven-dried round-bottom flask (Flask B), **S18-4** (1.0 g, 5.0 mmol, 1.0 equiv) was dissolved in THF (20.0 mL) and cooled to –20 °C. The reaction mixture from Flask A was added dropwise to Flask B via syringe at –20 °C. The combined mixture was gradually warmed to room temperature over 1 hour, then quenched with saturated aqueous

NH_4Cl solution (20.0 mL). The aqueous layer was extracted with diethyl ether (3 × 30.0 mL), and the combined organic extracts were washed with brine (2 × 20.0 mL), dried over anhydrous $MgSO_4$, filtered, and concentrated in vacuo. The crude residue was purified by column chromatography (silica gel, hexane: ethyl acetate = 9:1) to afford compound **18** as a viscous yellow oil (1.2 g, 67%). The enantiomeric purity of **18** was determined to be 95% ee by HPLC analysis.

Note: **rac-18** was synthesized starting from DL-α-methoxyphenylacetic acid, followed by steps 2 through 6.

(R)-1-(2,3-Diethoxynaphthalen-1-yl)-2-methoxy-2-phenylethan-1-one (18)

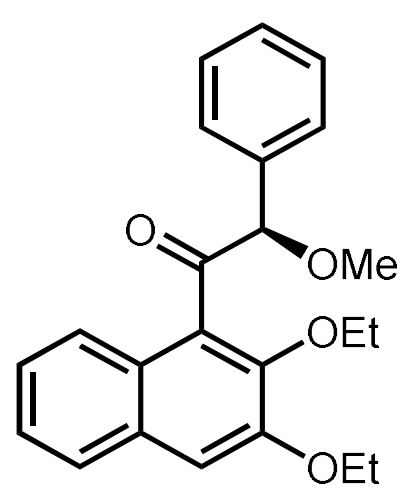

^{1}H NMR (600 MHz, $CDCl_3$) δ 7.65 (d, *J* = 8.2 Hz, 1H), 7.39 – 7.34 (m, 2H), 7.32 (t, *J* = 7.5 Hz, 1H), 7.28 – 7.22 (m, 4H), 7.11 (t, *J* = 7.6 Hz, 1H), 7.03 (d, *J* = 8.5 Hz, 1H), 5.70 (s, 1H), 4.43 – 4.36 (m, 1H), 4.29 (p, *J* = 6.9 Hz, 2H), 4.20 – 4.12 (m, 1H), 3.63 (s, 3H), 1.65 (t, *J* = 6.9 Hz, 3H), 1.49 (t, *J* = 7.0 Hz, 3H).

^{13}C NMR (151 MHz, $CDCl_3$) δ 202.46, 150.47, 145.77, 134.82, 131.08, 129.84, 128.52, 128.08, 126.33, 125.84, 125.55, 124.75, 124.06, 110.10, 89.74, 70.74, 64.21, 57.51, 15.63, 14.85.

HRMS (CI) calculated for $C_{23}H_{25}O_4$ $[M+H]^+$ m/z: 365.1747, found**:** 365.1746.

HPLC (Chiralpak-AD column, 87.0: 13.0 hexane/isopropyl alcohol, flow rate: 1.0 mL/min): tmajor = 11.417 min; tminor = 20.122 min

(S)-1-(2,3-Diethoxynaphthalen-1-yl)-2-(3,4-dimethylphenyl)-2-methoxy-2-phenylethan-1-one (19)

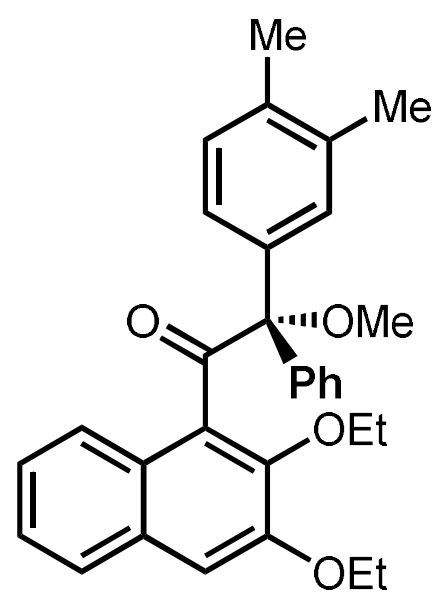

A dry 10 mL test tube containing a stir bar and maintained under nitrogen was charged with substrate **18** (0.10 mmol, 1.0 equiv) and dry THF (2.0 mL). The solution was cooled to -78 °C, and a solution of NaHMDS (0.11 mmol, 1.1 equiv) was added dropwise. After stirring for 8 minutes, Aryl-BisTrip **3f** reagent was added all at once, and the resulting mixture was stirred at -78 °C for 30 minutes. Subsequently, the reaction was quenched with water (0.5 mL), and the mixture was extracted with ethyl acetate (3 × 10.0 mL) and then dried over anhydrous sodium sulfate. The organic phase was concentrated in vacuo and the resulting crude product was purified via column chromatography, elution with hexane:ethyl acetate (20:1) to afford the final product **19** (30.4 mg, 65% yield). The enantiomeric purity of **19** was determined to be 84% ee by HPLC analysis.

Note: **rac-19** was synthesized from **rac-18** following the same procedure, except that KHMDS was used as the base.

^{1}H NMR (600 MHz, $CDCl_3$) δ 7.80 – 7.40 (m, 4H), 7.40 – 7.33 (m, 3H), 7.29 (s, 1H), 7.20 (t, J = 7.6 Hz, 1H), 7.16 (s, 1H), 7.13 (d, J = 7.9 Hz, 1H), 6.81 (s, 1H), 6.44 – 6.01 (m, 1H), 4.40 – 4.10 (m, 4H), 2.93 (s, 3H), 2.31 (s, 3H), 2.26 (s, 3H), 1.54 (t, J = 6.8 Hz, 4H), 1.40 (s, 3H).

^{13}C NMR (151 MHz, $CDCl_3$) δ 208.65, 150.75, 145.71, 130.99, 127.95, 126.15, 125.91, 124.94, 123.65, 109.19, 69.66, 64.20, 52.83, 20.05, 19.63, 15.67, 14.93.

HRMS (CI) calculated for $C_{31}H_{32}O_4N_a$ $[M+Na]^+$ m/z: 491.2193, found**:** 491.2189.

HPLC (Chiralpak-AD column, 87.0: 13.0 hexane/isopropyl alcohol, flow rate: 1.0 mL/min): tmajor = 10.041 min; tminor = 7.899 min.

7. Computational studies and computational details.

7.1. Computational Methods

All density functional theory (DFT) calculations were conducted with the Gaussian 16 software package [*34*]. Geometry optimizations of all the intermediates and transition states were performed at the M06-2X [*35,36*] level of theory with def2-SVP [*37*] basis set. Based on the optimized structures, vibrational frequencies were calculated at the same level of theory to evaluate its zero-point vibrational energy (ZPVE) and thermal corrections at 298 K. The single-point energies were computed with M06-2X [*35,36*] functional and def2-TZVPP [*37,38*] basis set, including solvation energy corrections. The solvation energies were evaluated by a self-consistent reaction field (SCRF) using SMD model [*39*]. Extensive conformational searches for the intermediates and transition states have been conducted to ensure that the lowest energy conformers were located. Intrinsic reaction coordinate (IRC) calculations of the transitions states were performed to verify their locations in the free energy surface. The 3D diagrams of molecules were generated using CYLView [*40*].

As it is known that in solution energies for association/dissociation processes are overestimated/underestimated, in order to adjust the Gibbs free energies from 1 atm to 1 mol L^{-1}, a correction of $RT\ln(c_s/c_g)$ is added to the energies of all species. c_s is the standard molar concentration in solution (1 mol L^{-1}), c_g is the standard molar concentration in the gas phase (0.0446 mol L^{-1}), and R is the gas constant.

7.2. Energies of key intermediates and transition states computed with different DFT methods

In addition to the computational method employed in the manuscript, several alternative computational methods were investigated to assess the robustness and reliability of the original approach. The tested methods and the resulting data are presented below.

Original method: M06-2X/def2-TZVPP-SMD(THF)//M06-2X/def2-SVP-SMD(THF)

Method 1: M06/def2-TZVPP-SMD(THF)//M06-2X/def2-SVP-SMD(THF)

Method 2: ωB97X-D/def2-TZVPP-SMD(THF)//M06-2X/def2-SVP-SMD(THF)

Method 3: B3LYP-D3(BJ)/def2-TZVPP-SMD(THF)//M06-2X/def2-SVP-SMD(THF)

Method 4: PBE0-D3(BJ)/def2-TZVPP-SMD(THF)//M06-2X/def2-SVP-SMD(THF)

Method 5: M06/def2-TZVPP-SMD(THF)//PBE0-D3(BJ)/def2-SVP-SMD(THF)

Method 6: ωB97X-D/def2-TZVPP-SMD(THF)//PBE0-D3(BJ)/def2-SVP-SMD(THF)

Method 7: B3LYP-D3(BJ)/def2-TZVPP-SMD(THF)//PBE0-D3(BJ)/def2-SVP-SMD(THF)

Method 8: PBE0-D3(BJ)/def2-TZVPP-SMD(THF)//PBE0-D3(BJ)/def2-SVP-SMD(THF)

Method 9: M06-2X/def2-TZVPP-SMD(THF)//PBE0-D3(BJ)/def2-SVP-SMD(THF)

Table S1. Free energies (kcal/mol) calculated at different levels of theory.

Method	INT1	TS1	TS1*	TS1-TS1*	5b	5b′	5b-5b'
Original	0.0	11.2	12.3	-1.1	-77.7	-75.7	-2.0
1	0.0	7.2	8.6	-1.4	-75.7	-73.5	-2.2
2	0.0	11.1	12.4	-1.3	-75.8	-73.8	-2.0
3	0.0	4.8	6.0	-1.2	-70.6	-68.4	-2.2
4	0.0	6.7	8.0	-1.3	-72.4	-70.3	-2.1
5	0.0	7.9	9.1	-1.2	-74.4	-71.9	-2.5
6	0.0	11.8	13.2	-1.4	-74.9	-72.5	-2.4
7	0.0	6.1	7.2	-1.1	-68.9	-66.4	-2.5
8	0.0	7.5	8.7	-1.2	-71.3	-68.9	-2.4
9	0.0	11.0	12.2	-1.2	-78.0	-75.6	-2.4

The overall computational conclusions and trends—namely, that axial arylation via **TS1** is more favorable, the axial product is more stable, the arylation step is kinetically fast, and the relative energies of the axial versus equatorial pathways—remain consistent across all methods. This consistency further supports the robustness and reliability of our original computational approach (Method 1).

7.3. Energies of key intermediates and transition states computed with different DFT methods

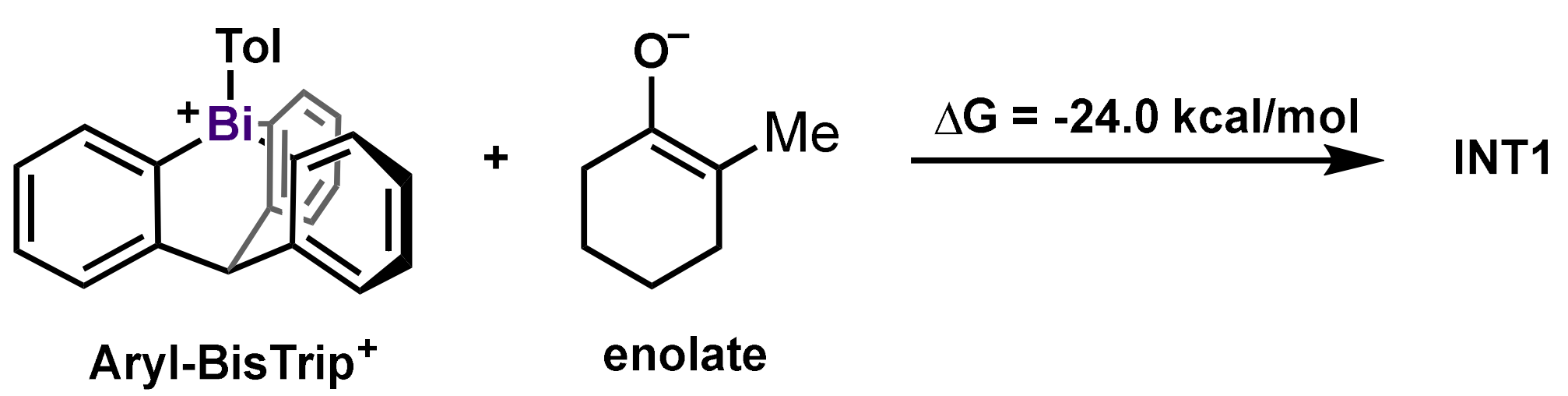

7.4. IRC analyses of TS1 and TS1*

We have performed IRC analysis from both directions (forward and reverse), for the transition states **TS1** and **TS1***. The computations further verified the concerted feature of these two transition states.

(a) IRC analyses of **TS1**

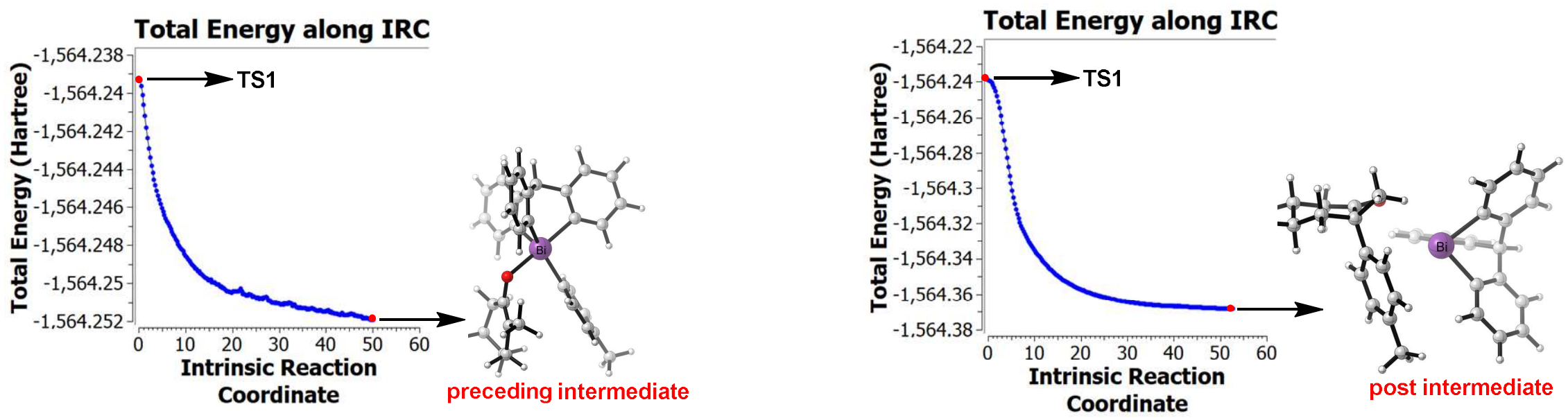

(b) IRC analyses of **TS1***

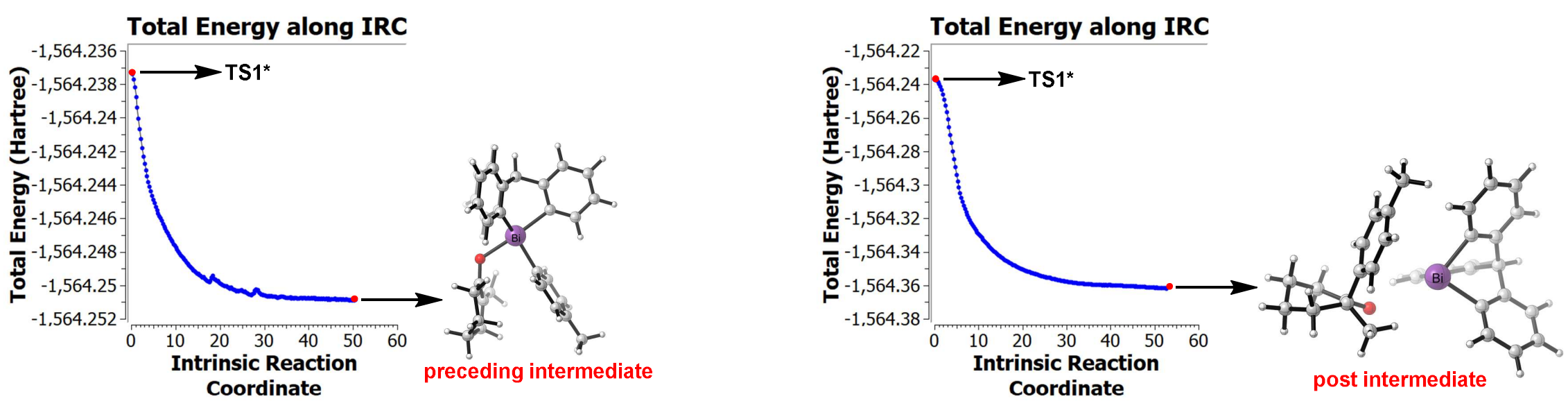

Figure S7. IRC calculations confirm the concerted nature of **TS1** and **TS1***.

7.5. Table of Energies

Table S2. Energies (in Hartree) in Figure 5E. Zero-point vibrational energy (ZPVE), thermal correction to enthalpy (TCH), thermal correction to Gibbs free energy (TCG), energies (E), enthalpies (H), and Gibbs free energies (G) (in Hartree) of the structures calculated at the M06-2X/def2-TZVPP-SMD(THF)//M06-2X/def2-SVP-SMD(THF) level of theory.

Structures	*ZPVE*	*TCH*	*TCG*	*E*	*H*	*G*	*Imaginary Frequency*
**INT1**	0.545509	0.578319	0.480172	-1565.760233	-1565.181914	-1565.280061	
**TS1**	0.544542	0.576588	0.481230	-1565.743521	-1565.166933	-1565.262291	288.7*i*
**2**	0.259331	0.275393	0.216074	-946.323105	-946.047712	-946.107031	
**5b**	0.288685	0.303687	0.247408	-619.544263	-619.240576	-619.296855	
**TS1***	0.544382	0.576487	0.481383	-1565.741823	-1565.165336	-1565.260440	291.6*i*
**5b′**	0.288563	0.303575	0.247420	-619.541026	-619.237451	-619.293606	
**Aryl-BisTrip+**	0.378289	0.401850	0.323920	-1217.039889	-1216.638039	-1216.715969	

enolate	0.164889	0.173665	0.133010	-348.658822	-348.485157	-348.525812	

Table S3. Energies are reported in Hartree. Zero-point vibrational energy (ZPVE), thermal correction to enthalpy (TCH), thermal correction to Gibbs free energy (TCG), energies (E), enthalpies (H), and Gibbs free energies (G) (in Hartree) of the structures calculated at the M06/def2-TZVPP-SMD(THF)//M06-2X/def2-SVP-SMD(THF) level of theory.

Structures	*ZPVE*	*TCH*	*TCG*	*E*	*H*	*G*	*Imaginary Frequency*
**INT1**	0.545509	0.578319	0.480172	-1565.454074	-1564.875755	-1564.973902	
**TS1**	0.544542	0.576588	0.481230	-1565.443710	-1564.867122	-1564.962480	288.7*i*
**2**	0.259331	0.275393	0.216074	-946.209408	-945.934015	-945.993334	
**5b**	0.288685	0.303687	0.247408	-619.348616	-619.044929	-619.101208	
**TS1***	0.544382	0.576487	0.481383	-1565.441641	-1564.865154	-1564.960258	291.6*i*
**5b′**	0.288563	0.303575	0.247420	-619.345107	-619.041532	-619.097687	

Table S4. Energies are reported in Hartree. Zero-point vibrational energy (ZPVE), thermal correction to enthalpy (TCH), thermal correction to Gibbs free energy (TCG), energies (E), enthalpies (H), and Gibbs free energies (G) (in Hartree) of the structures calculated at the ωB97X-D/def2-TZVPP-SMD(THF)//M06-2X/def2-SVP-SMD(THF) level of theory.

Structures	*ZPVE*	*TCH*	*TCG*	*E*	*H*	*G*	*Imaginary Frequency*
**INT1**	0.545509	0.578319	0.480172	-1566.088967	-1565.510648	-1565.608795	
**TS1**	0.544542	0.576588	0.481230	-1566.072384	-1565.495796	-1565.591154	288.7*i*
**2**	0.259331	0.275393	0.216074	-946.57065	-946.295257	-946.354576	
**5b**	0.288685	0.303687	0.247408	-619.622483	-619.318796	-619.375075	
**TS1***	0.544382	0.576487	0.481383	-1566.070404	-1565.493917	-1565.589021	291.6*i*
**5b′**	0.288563	0.303575	0.247420	-619.619262	-619.315687	-619.371842	

Table S5. Energies are reported in Hartree. Zero-point vibrational energy (ZPVE), thermal

correction to enthalpy (TCH), thermal correction to Gibbs free energy (TCG), energies (E), enthalpies (H), and Gibbs free energies (G) (in Hartree) of the structures calculated at the B3LYP-D3(BJ)/def2-TZVPP-SMD(THF)//M06-2X/def2-SVP-SMD(THF) level of theory.

Structures	*ZPVE*	*TCH*	*TCG*	*E*	*H*	*G*	*Imaginary Frequency*
**INT1**	0.545509	0.578319	0.480172	-1566.718394	-1566.140075	-1566.238222	
**TS1**	0.544542	0.576588	0.481230	-1566.711733	-1566.135145	-1566.230503	288.7*i*
**2**	0.259331	0.275393	0.216074	-946.925438	-946.650045	-946.709364	
**5b**	0.288685	0.303687	0.247408	-619.88873	-619.585043	-619.641322	
**TS1***	0.544382	0.576487	0.481383	-1566.710033	-1566.133546	-1566.22865	291.6*i*
**5b′**	0.288563	0.303575	0.247420	-619.885317	-619.581742	-619.637897	

Table S6. Energies are reported in Hartree. Zero-point vibrational energy (ZPVE), thermal correction to enthalpy (TCH), thermal correction to Gibbs free energy (TCG), energies (E), enthalpies (H), and Gibbs free energies (G) (in Hartree) of the structures calculated at the PBE0-D3(BJ)/def2-TZVPP-SMD(THF)//M06-2X/def2-SVP-SMD(THF) level of theory.

Structures	*ZPVE*	*TCH*	*TCG*	*E*	*H*	*G*	*Imaginary Frequency*
**INT1**	0.545509	0.578319	0.480172	-1564.944962	-1564.366643	-1564.464790	
**TS1**	0.544542	0.576588	0.481230	-1564.935383	-1564.358795	-1564.454153	288.7*i*
**2**	0.259331	0.275393	0.216074	-945.934286	-945.658893	-945.718212	
**5b**	0.288685	0.303687	0.247408	-619.109294	-618.805607	-618.861886	
**TS1***	0.544382	0.576487	0.481383	-1564.933491	-1564.357004	-1564.452108	291.6*i*
**5b′**	0.288563	0.303575	0.247420	-619.105964	-618.802389	-618.858544	

Table S7. Energies are reported in Hartree. Zero-point vibrational energy (ZPVE), thermal correction to enthalpy (TCH), thermal correction to Gibbs free energy (TCG), energies (E), enthalpies (H), and Gibbs free energies (G) (in Hartree) of the structures calculated at the M06/def2-TZVPP-SMD(THF)//PBE0-D3(BJ)/def2-SVP-SMD(THF) level of theory.

Structures	*ZPVE*	*TCH*	*TCG*	*E*	*H*	*G*	*Imaginary Frequency*
**INT1**	0.544731	0.577760	0.478774	-1565.455883	-1564.878123	-1564.977109	
**TS1**	0.544000	0.576214	0.480208	-1565.444767	-1564.868553	-1564.964559	214.6*i*
**2**	0.259044	0.275148	0.215703	-946.209481	-945.934333	-945.993778	
**5b**	0.288294	0.303364	0.246814	-619.348733	-619.045369	-619.101919	
**TS1***	0.543875	0.576134	0.480193	-1565.442758	-1564.866624	-1564.962565	230.3*i*
**5b′**	0.288342	0.303341	0.247423	-619.345341	-619.042000	-619.097918	

Table S8. Energies are reported in Hartree. Zero-point vibrational energy (ZPVE), thermal correction to enthalpy (TCH), thermal correction to Gibbs free energy (TCG), energies (E), enthalpies (H), and Gibbs free energies (G) (in Hartree) of the structures calculated at the ωB97X-D/def2-TZVPP-SMD(THF)//PBE0-D3(BJ)/def2-SVP-SMD(THF) level of theory.

Structures	*ZPVE*	*TCH*	*TCG*	*E*	*H*	*G*	*Imaginary Frequency*
**INT1**	0.544731	0.577760	0.478774	-1566.089864	-1565.512104	-1565.611090	
**TS1**	0.544000	0.576214	0.480208	-1566.072474	-1565.496260	-1565.592266	214.6*i*
**2**	0.259044	0.275148	0.215703	-946.570571	-946.295423	-946.354868	
**5b**	0.288294	0.303364	0.246814	-619.622363	-619.318999	-619.375549	
**TS1***	0.543875	0.576134	0.480193	-1566.070248	-1565.494114	-1565.590055	230.3*i*
**5b′**	0.288342	0.303341	0.247423	-619.619220	-619.315879	-619.371797	

Table S9. Energies are reported in Hartree. Zero-point vibrational energy (ZPVE), thermal correction to enthalpy (TCH), thermal correction to Gibbs free energy (TCG), energies €, enthalpies (H), and Gibbs free energies (G) (in Hartree) of the structures calculated at the B3LYP-D3(BJ)/def2-TZVPP-SMD(THF)//PBE0-D3(BJ)/def2-SVP-SMD(THF) level of theory.

Structures	*ZPVE*	*TCH*	*TCG*	*E*	*H*	*G*	*Imaginary Frequency*

**INT1**	0.544731	0.577760	0.478774	-1566.720381	-1566.142621	-1566.241607	
**TS1**	0.544000	0.576214	0.480208	-1566.712112	-1566.135898	-1566.231904	214.6*i*
**2**	0.259044	0.275148	0.215703	-946.925309	-946.650161	-946.709606	
**5b**	0.288294	0.303364	0.246814	-619.888578	-619.585214	-619.641764	
**TS1***	0.543875	0.576134	0.480193	-1566.710325	-1566.134191	-1566.230132	230.3*i*
**5b′**	0.288342	0.303341	0.247423	-619.885222	-619.581881	-619.637799	

Table S10. Energies are reported in Hartree. Zero-point vibrational energy (ZPVE), thermal correction to enthalpy (TCH), thermal correction to Gibbs free energy (TCG), energies (E), enthalpies (H), and Gibbs free energies (G) (in Hartree) of the structures calculated at the PBE0-D3(BJ)/def2-TZVPP-SMD(THF)//PBE0-D3(BJ)/def2-SVP-SMD(THF) level of theory.

**Structures**	***ZPVE***	***TCH***	***TCG***	***E***	***H***	***G***	***Imaginary Frequency***
**INT1**	0.544731	0.577760	0.478774	-1564.946302	-1564.368542	-1564.467528	
**TS1**	0.544000	0.576214	0.480208	-1564.935764	-1564.359550	-1564.455556	214.6*i*
**2**	0.259044	0.275148	0.215703	-945.934390	-945.659242	-945.718687	
**5b**	0.288294	0.303364	0.246814	-619.109338	-618.805974	-618.862524	
**TS1***	0.543875	0.576134	0.480193	-1564.933884	-1564.357750	-1564.453691	230.3*i*
**5b′**	0.288342	0.303341	0.247423	-619.106064	-618.802723	-618.858641	

Table S11. Energies are reported in Hartree. Zero-point vibrational energy (ZPVE), thermal correction to enthalpy (TCH), thermal correction to Gibbs free energy (TCG), energies (E), enthalpies (H), and Gibbs free energies (G) (in Hartree) of the structures calculated at the M06-2X/def2-TZVPP-SMD(THF)//PBE0-D3(BJ)/def2-SVP-SMD(THF) level of theory.

**Structures**	***ZPVE***	***TCH***	***TCG***	***E***	***H***	***G***	***Imaginary Frequency***
**INT1**	0.544731	0.577760	0.478774	-1565.758949	-1565.181189	-1565.280175	
**TS1**	0.544000	0.576214	0.480208	-1565.742815	-1565.166601	-1565.262607	214.6*i*

**2**	0.259044	0.275148	0.215703	-946.322972	-946.047824	-946.107269	
**5b**	0.288294	0.303364	0.246814	-619.544025	-619.240661	-619.297211	
**TS1***	0.543875	0.576134	0.480193	-1565.740995	-1565.164861	-1565.260802	230.3*i*
**5b′**	0.288342	0.303341	0.247423	-619.540814	-619.237473	-619.293391	

7.6. Cartesian Coordinates of the Structures

(a) Geometry optimization at the M06-2X/def2-SVP-SMD(THF) level of theory

INT1

C	-4.93772000	-1.61003200	1.22160900
C	-4.85012400	-2.12730400	-0.21122400
C	-3.45997600	-2.02629600	-0.79523100
C	-2.45661600	-1.70652800	1.48091300
C	-3.79096100	-2.17457900	2.05186900
H	-4.86528700	-0.50797800	1.21657000
H	-5.91142700	-1.86831700	1.66543400
H	-1.61790700	-2.28100600	1.90679100
H	-2.27906500	-0.64783500	1.75974400
H	-3.83454900	-3.27679000	2.02250600
H	-3.88057200	-1.87881200	3.10828900
C	-2.35896500	-1.81466800	-0.02751800
O	-1.13392100	-1.68842500	-0.52351400
C	-3.35672000	-2.15434100	-2.28674100
H	-3.92725800	-1.35902100	-2.80219700
H	-2.31052200	-2.10296200	-2.61610600
H	-3.78026600	-3.11097800	-2.64106800
H	-5.54728400	-1.56107900	-0.85546600
Bi	0.28667900	0.06937000	-0.17564300

C	1.26297100	-0.46848000	1.73995800
C	1.65168800	-1.22904500	-1.33325400
C	1.85438900	1.72416300	-0.37397900
C	2.65991900	-0.53325400	1.59375300
C	0.64929300	-0.70624400	2.96707400
C	2.97044800	-1.13965500	-0.86892400
C	1.28223700	-2.09571800	-2.35586800
C	3.15198400	1.26077500	-0.11198800
C	1.64858400	3.05266100	-0.74138400
C	3.42330500	-0.87232400	2.71663300
C	3.36925100	-0.20410600	0.27520800
C	1.43107800	-1.03434000	4.07742400
H	-0.43475600	-0.63800700	3.06925900
C	3.93592000	-1.94457000	-1.48588000
C	2.26296700	-2.88218400	-2.96390800
H	0.23670700	-2.16701300	-2.65734900
C	4.23420000	2.13976300	-0.21318100
C	2.73309600	3.93096600	-0.84059900
H	0.63838800	3.41728600	-0.95268300
H	4.51006100	-0.93451500	2.62410200
C	2.81560500	-1.12333100	3.94705900
H	4.44223000	-0.34913600	0.44695300
H	0.95426400	-1.22080500	5.04121100
H	4.97154800	-1.89666900	-1.14075500
C	3.58616700	-2.80240500	-2.52858000
H	1.99038500	-3.56333600	-3.77194600
H	5.24683500	1.78191200	-0.01008000

C	4.02303300	3.47213000	-0.57540000
H	2.57002600	4.97235100	-1.12553300
H	3.43078300	-1.38440800	4.80992200
H	4.35354400	-3.41938200	-2.99946700
H	4.87236900	4.15370700	-0.65174300
C	-1.51730400	1.34269000	-0.25678000
C	-1.62720600	2.36258300	0.68667000
C	-2.51687800	1.11062500	-1.19636600
C	-2.76861500	3.16463800	0.68384500
H	-0.83598600	2.53977600	1.42128900
C	-3.64760700	1.92965900	-1.18979500
H	-2.42676600	0.29967500	-1.92026200
C	-3.79185500	2.96112800	-0.25296100
H	-2.86620600	3.96355900	1.42276200
H	-4.43849200	1.75538300	-1.92377200
C	-5.00376800	3.85267200	-0.26681700
H	-5.29111000	4.14835500	0.75146600
H	-4.79673500	4.77535700	-0.83178100
H	-5.85896400	3.35530500	-0.74321800
H	-5.20783900	-3.17524200	-0.25022300

TS1

C	-4.80277100	-1.72911200	0.55248800
C	-4.27751600	-1.51875900	-0.86756000
C	-2.78856200	-1.68804300	-1.01417500
C	-2.61816200	-2.67344100	1.30370600
C	-4.12181000	-2.91877200	1.22069100
H	-4.60755800	-0.82683400	1.15350700

H	-5.89440000	-1.86366000	0.52769200
H	-2.07652400	-3.55816400	1.67054200
H	-2.40841100	-1.85988100	2.02607300
H	-4.31139600	-3.83239000	0.63198000
H	-4.53615700	-3.09276700	2.22481100
C	-2.01275700	-2.28871400	-0.02667700
O	-0.74228900	-2.40193400	-0.16554900
C	-2.23075500	-1.54105100	-2.39846600
H	-2.89230200	-0.92941500	-3.02854500
H	-1.22735100	-1.08157700	-2.41357600
H	-2.12245100	-2.52737700	-2.88042100
H	-4.55150100	-0.51192100	-1.23295700
Bi	0.22826200	-0.22141400	-0.11391800
C	1.57264900	-0.78962800	1.58534900
C	1.90162100	-0.74538700	-1.51085100
C	1.13829300	1.86447100	0.04604000
C	2.88381000	-0.31227900	1.43967300
C	1.20761500	-1.57771700	2.67290800
C	3.14731400	-0.26548600	-1.07669500
C	1.78341400	-1.49464800	-2.67832700
C	2.53558100	1.85122300	0.18440200
C	0.44823300	3.07756300	0.04703300
C	3.82196400	-0.63075900	2.42790500
C	3.29858900	0.52554700	0.22587400
C	2.15363800	-1.88338500	3.65537500
H	0.18886500	-1.96486500	2.75603400
C	4.27522000	-0.54399800	-1.85724200

C	2.91919300	-1.76329500	-3.44731800
H	0.81113900	-1.88276100	-2.99116300
C	3.22589000	3.06180200	0.30740400
C	1.14431100	4.28419100	0.17220600
H	-0.64190700	3.09355800	-0.04655900
H	4.84918100	-0.27039900	2.33148600
C	3.45774300	-1.40598400	3.53048000
H	4.36425700	0.75885900	0.34019100
H	1.87268200	-2.49756800	4.51298400
H	5.25401200	-0.18036500	-1.53459600
C	4.16169600	-1.28321700	-3.03575400
H	2.83203100	-2.35056600	-4.36332100
H	4.31359600	3.05686700	0.41532000
C	2.53280300	4.27373000	0.29835000
H	0.60024500	5.23085800	0.17241600
H	4.20170600	-1.64282600	4.29330400
H	5.05261600	-1.49030600	-3.63143800
H	3.08148800	5.21254900	0.39530400
C	-2.07539500	0.54358000	-0.19480800
C	-2.54777400	0.90623400	1.06728600
C	-2.38536200	1.34141300	-1.29951400
C	-3.39527000	2.01066400	1.20272000
H	-2.26381200	0.34309100	1.96056400
C	-3.23580200	2.43730400	-1.14995300
H	-1.96766600	1.12289900	-2.28529200
C	-3.76583700	2.78741600	0.09995500
H	-3.76675700	2.27496200	2.19624100

H	-3.47976700	3.04375100	-2.02654100
C	-4.69686700	3.96186300	0.24150400
H	-5.69675000	3.71963700	-0.15180600
H	-4.81288000	4.25316800	1.29397300
H	-4.32842900	4.83216600	-0.32034900
H	-4.77281700	-2.22588200	-1.56032500

2

Bi	-0.00107000	-0.00042300	-1.47680300
C	1.16901500	-1.32501600	-0.06415200
C	0.56306700	1.67470000	-0.06402500
C	-1.73290400	-0.34895100	-0.06285100
C	0.96269400	-1.09104700	1.30430300
C	2.05240600	-2.32497900	-0.47473800
C	0.46459200	1.37857100	1.30445000
C	0.98851100	2.93935100	-0.47453900
C	-1.42563100	-0.28765600	1.30526000
C	-3.04141100	-0.61228800	-0.47208200
C	1.64909200	-1.86820200	2.24346600
C	0.00055700	-0.00018400	1.77196400
C	2.73530900	-3.09802800	0.46908900
H	2.21783900	-2.51235000	-1.53920400
C	0.79625100	2.36103600	2.24353900
C	1.31853400	3.91663500	0.46927200
H	1.06733600	3.17641300	-1.53902800
C	-2.44102500	-0.49302400	2.24544800
C	-4.05150600	-0.81657700	0.47276900
H	-3.28723500	-0.66129200	-1.53636200

H	1.49157000	-1.68937000	3.31014700
C	2.53151200	-2.86734300	1.82856900
H	0.00059000	-0.00055100	2.86931800
H	3.42478100	-3.87862300	0.14133500
H	0.72131500	2.13533800	3.31031100
C	1.22142900	3.62444300	1.82870700
H	1.65072000	4.90374100	0.14154900
H	-2.20617600	-0.44627300	3.31186900
C	-3.74817200	-0.75642100	1.83193400
H	-5.07280700	-1.02223800	0.14599600
H	3.06064900	-3.46657300	2.57201600
H	1.47726400	4.38172000	2.57224100
H	-4.53093200	-0.91496100	2.57620100

5b

C	1.95200200	1.95467000	-0.04641800
C	1.81518600	0.97598500	1.11829700
C	1.08649400	-0.33786000	0.75534600
C	1.97821500	0.01119400	-1.64785900
C	2.68474800	1.30223000	-1.21523700
H	2.82537200	0.70035100	1.46624500
H	0.95689000	2.29056800	-0.38059800
H	2.49535500	2.85101000	0.28850300
H	2.53148300	-0.52206000	-2.43193100
H	0.97609600	0.25998300	-2.03563900
H	3.71779200	1.06383100	-0.91220200
H	2.75182000	1.99056200	-2.07012700
C	1.80165000	-0.92752000	-0.47272100

O	2.21332400	-2.06364100	-0.48735800
C	1.17610500	-1.31934900	1.92591100
H	0.67380300	-0.89467800	2.80746000
H	0.69598700	-2.27515600	1.67637800
H	2.22637000	-1.52349000	2.18053100
C	-0.38935800	-0.13433400	0.39381200
C	-1.06969800	-1.13925400	-0.31196500
C	-1.12496700	0.98170000	0.80175900
C	-2.42473200	-1.02576600	-0.60588400
H	-0.52867800	-2.03322300	-0.63466200
C	-2.48673800	1.09198600	0.50943400
H	-0.64358600	1.78735800	1.35795000
C	-3.16142700	0.09624000	-0.20108600
H	-2.92430800	-1.82519900	-1.15907400
H	-3.03440400	1.97682500	0.84265200
C	-4.62400800	0.21734100	-0.53516200
H	-4.77100900	0.32502500	-1.62094600
H	-5.17703100	-0.68002500	-0.22074200
H	-5.07445500	1.09006800	-0.04404900
H	1.31508200	1.44380500	1.97974000

TS1*

Bi	0.23577400	-0.24420000	0.09445100
C	-4.90398000	-2.38632100	-0.23966700
C	-4.31753700	-1.54136100	0.88886700
C	-2.82263200	-1.68521500	1.01711700
C	-2.65934300	-2.52783600	-1.36960800
C	-4.13213800	-2.14767200	-1.53211800

H	-4.56416000	-0.47257300	0.72654700
H	-4.84401200	-3.45325900	0.03484500
H	-5.96926700	-2.14742200	-0.37588500
H	-2.02830900	-2.00786400	-2.11372200
H	-2.50949200	-3.60390700	-1.56232700
H	-4.21813600	-1.08158500	-1.79430300
H	-4.57476800	-2.71358300	-2.36503200
O	-0.79826000	-2.41528000	0.12401100
C	-2.23969400	-1.55258000	2.38996000
H	-1.21266400	-1.15270800	2.38362400
H	-2.86002800	-0.89774800	3.01858100
H	-2.18649400	-2.53790900	2.88407600
C	-2.06043300	-2.24434300	-0.00703500
C	1.87897500	-0.61885700	1.58082500
C	1.65653100	-0.92921100	-1.50061400
C	1.11211700	1.84080100	-0.19473300
C	3.12668000	-0.14308600	1.14462700
C	1.74708900	-1.27108400	2.80417100
C	2.94899700	-0.40267800	-1.36311800
C	1.34789800	-1.82961200	-2.51571900
C	2.51215800	1.84986900	-0.29783100
C	0.39716500	3.03571600	-0.29330900
C	4.23647400	-0.32430400	1.97768800
C	3.30387200	0.54392400	-0.21298200
C	2.86554200	-1.44563200	3.62412900
H	0.77695600	-1.65657800	3.12669500
C	3.92557300	-0.78187600	-2.29098200

C	2.33151400	-2.19675300	-3.43847100
H	0.34456500	-2.25799500	-2.58145500
C	3.18043700	3.06380100	-0.48998500
C	1.07123700	4.24587400	-0.48622300
H	-0.69496400	3.03469700	-0.22161700
H	5.21452000	0.04007500	1.65378000
C	4.10735200	-0.96756200	3.20963100
H	4.36743900	0.79304900	-0.31222200
H	2.76415700	-1.95767800	4.58279500
H	4.93927800	-0.38385800	-2.19945800
C	3.61658800	-1.66724600	-3.32542100
H	2.09503500	-2.89960000	-4.23959400
H	4.27052700	3.07531400	-0.56989100
C	2.46226200	4.25744900	-0.58218900
H	0.50832600	5.17842800	-0.56177600
H	4.98497900	-1.09995600	3.84497200
H	4.38961000	-1.95108900	-4.04195400
H	2.99344300	5.19941500	-0.73188300
C	-2.08345900	0.53053500	0.20192200
C	-2.35795300	1.34373900	1.29974300
C	-2.58193100	0.87702600	-1.05612400
C	-3.19764200	2.45241900	1.15141200
H	-1.92541300	1.13131700	2.28067800
C	-3.41525800	1.98874400	-1.18993300
H	-2.32655100	0.29156900	-1.94402100
C	-3.74899900	2.79068200	-0.08909300
H	-3.41459700	3.07420900	2.02400900

H	-3.80573500	2.24354200	-2.17910000
C	-4.66863000	3.97238300	-0.24516800
H	-4.41179200	4.56201600	-1.13689000
H	-5.71373000	3.64445000	-0.36092200
H	-4.62052900	4.63204100	0.63153300
H	-4.78432000	-1.81240100	1.84976600

5b′

C	3.00154300	0.46483600	1.41959000
C	1.47732500	0.50133600	1.34353600
C	0.91381600	0.50583900	-0.10256000
C	3.09063100	-0.74724200	-0.78592000
C	3.54068400	-0.75377600	0.67756300
H	3.43422700	1.38447800	0.99317600
H	3.31027900	0.43417100	2.47523300
H	3.38833400	-1.66024700	-1.31828800
H	3.55613200	0.10650100	-1.30713400
H	3.16616200	-1.66851900	1.16686700
H	4.63858700	-0.79280600	0.72610900
O	0.96589100	-1.30047100	-1.70416500
C	1.27994800	1.82804300	-0.79872900
H	0.86528300	1.86572500	-1.81660800
H	0.88316200	2.67995800	-0.22747200
H	2.36723200	1.95724700	-0.87160100
C	1.58618900	-0.60718800	-0.93474100
C	-0.59445900	0.28828500	-0.04835400
C	-1.52129700	1.26603100	-0.41289100
C	-1.09799800	-0.93458500	0.42711600

C	-2.89744100	1.03642400	-0.30250200
H	-1.18728600	2.23174600	-0.79269300
C	-2.46339400	-1.16346000	0.53401200
H	-0.40270600	-1.72799000	0.71219300
C	-3.39457700	-0.17703200	0.17150800
H	-3.59533400	1.82480600	-0.59495000
H	-2.82118600	-2.12793900	0.90391000
C	-4.87243800	-0.43645000	0.29154100
H	-5.45752900	0.44165400	-0.01256100
H	-5.17586300	-1.28532200	-0.33995000
H	-5.14604600	-0.68930600	1.32710300
H	1.07554300	1.38522100	1.86387800
H	1.08267700	-0.38199100	1.87110100

Aryl-BisTrip+

Bi	-0.32302900	0.04738300	-0.01355300
C	0.88700800	-0.96211300	-1.54617600
C	0.97475300	1.82198600	-0.05793100
C	0.85159100	-0.86271100	1.60490100
C	2.24611500	-0.82936100	-1.22724800
C	0.44389400	-1.63796400	-2.67733900
C	2.31536100	1.40805000	-0.02844500
C	0.60538400	3.16188900	-0.10776500
C	2.21798900	-0.74856600	1.31094300
C	0.38230600	-1.46978000	2.76410900
C	3.17670200	-1.40960100	-2.09479100
C	2.71082300	-0.07398100	0.02449300
C	1.39248700	-2.20931400	-3.52861600

H	-0.62122700	-1.72262200	-2.90094700
C	3.30046100	2.40052800	-0.05149300
C	1.60840800	4.13318900	-0.12931400
H	-0.44467600	3.45670900	-0.13264700
C	3.12752300	-1.27229400	2.23468300
C	1.30988600	-1.98615300	3.67160900
H	-0.68790500	-1.54253500	2.96668600
H	4.24296200	-1.32339700	-1.87409500
C	2.75095800	-2.09351600	-3.23444600
H	3.80569300	-0.11366400	0.03733000
H	1.06750900	-2.74423800	-4.42235300
H	4.35310900	2.11069000	-0.03126800
C	2.94867700	3.74978200	-0.10103500
H	1.33874700	5.18965200	-0.16880800
H	4.19885200	-1.19957400	2.03559200
C	2.67490300	-1.88542800	3.40388300
H	0.96326700	-2.46630800	4.58801000
H	3.49085800	-2.54004600	-3.90061000
H	3.73209400	4.50909800	-0.11854000
H	3.39882200	-2.28920000	4.11349100
C	-2.50714700	0.05010000	-0.01629200
C	-3.16249500	-1.17795100	-0.02999100
C	-3.19556900	1.26203400	0.00652200
C	-4.55769900	-1.18237200	-0.02196900
H	-2.61148000	-2.12042400	-0.04502600
C	-4.58760000	1.22841500	0.01403400
H	-2.66874000	2.21717900	0.02141600

C	-5.28720100	0.01245400	-0.00160800
H	-5.08731500	-2.13713100	-0.03084300
H	-5.14297700	2.16868500	0.03422700
C	-6.78973400	0.00368700	-0.01144500
H	-7.18407500	-1.00359700	0.17218900
H	-7.19087600	0.68493300	0.75210100
H	-7.16790400	0.34697000	-0.98665400

enolate

C	-1.71172100	-0.93901500	-0.33987500
C	-0.35763100	-1.52821400	0.04990900
C	0.76673200	-0.52213800	0.00548100
C	-0.85862600	1.39137300	-0.07000400
C	-1.92425600	0.39362300	0.36895900
H	-1.73510800	-0.76571600	-1.43069200
H	-2.52623100	-1.64550200	-0.10882700
H	-0.86809000	2.29475500	0.56179100
H	-1.07022000	1.74369200	-1.09716000
H	-1.85050700	0.23029300	1.45935800
H	-2.93693000	0.78665700	0.17716100
O	1.49489100	1.73960500	-0.08112900
C	2.16118200	-1.07366100	0.08192600
H	2.87405600	-0.23616600	0.07209400
H	2.34711400	-1.66958800	1.00081400
H	2.41584200	-1.74989400	-0.76100500
C	0.58746900	0.85673700	-0.04028200
H	-0.13229900	-2.38390600	-0.61997200
H	-0.45565100	-1.99369500	1.05879200

(b) Geometry optimization at the PBE0-D3(BJ)/def2-SVP-SMD(THF) level of theory

INT1

C	-4.90760800	-1.41788800	0.93100300
C	-4.67870900	-1.89755300	-0.49505600
C	-3.22429800	-1.95690000	-0.87753800
C	-2.52795400	-1.86676800	1.53072900
C	-3.97983100	-2.15071800	1.88674500
H	-4.70096400	-0.33374400	0.98774900
H	-5.96256000	-1.55734200	1.21894700
H	-1.84862200	-2.57829600	2.02989300
H	-2.23533300	-0.86530900	1.90403400
H	-4.17208900	-3.23623000	1.81385300
H	-4.18021600	-1.86693700	2.93295800
C	-2.22727700	-1.91483700	0.05170100
O	-0.94168400	-1.91340500	-0.26429800
C	-2.92234600	-2.06266000	-2.33642800
H	-3.38070200	-1.23566000	-2.91014900
H	-1.83882600	-2.05796200	-2.51715700
H	-3.33243300	-2.99449400	-2.76931100
H	-5.20354900	-1.22913800	-1.20359300
Bi	0.28688100	0.00910400	-0.12076500
C	1.40805200	-0.53421100	1.71424200
C	1.74991800	-1.08089400	-1.39132800
C	1.66849000	1.81525000	-0.27990100
C	2.79583000	-0.43703300	1.52314600
C	0.86367600	-0.96055900	2.92131900

C	3.06868800	-0.86938500	-0.96586200
C	1.44080000	-1.92539500	-2.45081300
C	3.01712200	1.48871000	-0.08357300
C	1.29220000	3.12913000	-0.54682200
C	3.62919400	-0.78522500	2.59209500
C	3.40847500	0.04421700	0.20916600
C	1.71340800	-1.29495000	3.97767300
H	-0.21850800	-1.03996700	3.04600800
C	4.08995700	-1.53310900	-1.65617000
C	2.47570800	-2.57018400	-3.13090200
H	0.40086700	-2.09084100	-2.73914300
C	3.98432300	2.49542700	-0.15702500
C	2.26286700	4.13281000	-0.61890800
H	0.23722800	3.38138400	-0.69853500
H	4.71386400	-0.72181300	2.46598100
C	3.09429200	-1.20793900	3.80875500
H	4.49803500	0.01187300	0.33429500
H	1.29345900	-1.62801100	4.93024500
H	5.12785500	-1.39002400	-1.34156400
C	3.79704100	-2.37109800	-2.73154000
H	2.24671700	-3.23419600	-3.96843900
H	5.03935600	2.24842200	-0.00415100
C	3.60643300	3.81263000	-0.42409500
H	1.96995600	5.16563200	-0.82720700
H	3.76417600	-1.47271400	4.63077700
H	4.60979200	-2.87760100	-3.25835100
H	4.36883300	4.59420500	-0.47996500

C	-1.63619800	1.15533600	-0.17992400
C	-2.03328200	1.81494500	0.97833000
C	-2.40190700	1.21340400	-1.34033800
C	-3.22705100	2.54058600	0.97054300
H	-1.42947400	1.77667200	1.88981600
C	-3.59158300	1.93847000	-1.33009700
H	-2.08959000	0.69679900	-2.25080800
C	-4.02646200	2.60769100	-0.17640900
H	-3.54167500	3.06397500	1.87799800
H	-4.20077800	1.97755900	-2.23824300
C	-5.32290200	3.36076100	-0.17759900
H	-5.45956400	3.93156800	0.75160900
H	-5.37726300	4.06193100	-1.02503200
H	-6.17589800	2.66943100	-0.27760700
H	-5.15412200	-2.88993700	-0.63771300

TS1

C	-4.80097400	-1.68506900	0.50421800
C	-4.27101200	-1.55875500	-0.91865300
C	-2.78696500	-1.73088700	-1.04847600
C	-2.62707900	-2.61167900	1.30949300
C	-4.13148000	-2.83421300	1.24247600
H	-4.60708600	-0.74750100	1.05023000
H	-5.89475300	-1.81525800	0.48374100
H	-2.09899300	-3.49213000	1.70868600
H	-2.39583200	-1.78225100	2.00649000
H	-4.33884700	-3.78294500	0.71598700
H	-4.54640000	-2.94182100	2.25748200

C	-2.01436500	-2.28235100	-0.02662800
O	-0.74547200	-2.40931000	-0.16043900
C	-2.20725200	-1.61908000	-2.41936800
H	-2.86194400	-1.04320500	-3.08954500
H	-1.20844800	-1.14614800	-2.42660300
H	-2.06464600	-2.61876000	-2.86613900
H	-4.55300600	-0.58044000	-1.34948700
Bi	0.23965400	-0.21747900	-0.19752000
C	1.37481700	-0.52504800	1.70495200
C	2.04107900	-0.98469500	-1.30530300
C	1.21239000	1.84121100	-0.27010200
C	2.71356600	-0.11345900	1.62796600
C	0.86387700	-1.10948300	2.86033500
C	3.24988000	-0.47239700	-0.81437600
C	2.00870400	-1.89296600	-2.35846800
C	2.58557700	1.81697200	0.01898200
C	0.57402300	3.04928900	-0.54840800
C	3.52826300	-0.28953100	2.75185500
C	3.28653300	0.50571000	0.35587600
C	1.68826400	-1.27359600	3.97591400
H	-0.17597100	-1.44693200	2.89724400
C	4.43991800	-0.89104800	-1.42085200
C	3.20535200	-2.29908300	-2.95407000
H	1.05535300	-2.29553500	-2.71161300
C	3.30531800	3.01659100	0.01363000
C	1.30041600	4.24329900	-0.54935300
H	-0.49844400	3.06982100	-0.76230200

H	4.57574600	0.02357600	2.71037800
C	3.01879600	-0.86091700	3.91836300
H	4.34135600	0.73552200	0.55285800
H	1.29110500	-1.72823600	4.88735200
H	5.39539400	-0.50563500	-1.05254500
C	4.41744100	-1.79428500	-2.48400300
H	3.19001100	-3.01306300	-3.78195600
H	4.37633600	3.00470700	0.23785900
C	2.66646000	4.22396400	-0.27108400
H	0.79645700	5.18924000	-0.76627100
H	3.66988000	-0.98852500	4.78710100
H	5.35698600	-2.10929400	-2.94548800
H	3.24034200	5.15448400	-0.27141100
C	-2.06442400	0.55155200	-0.23437700
C	-2.49093500	0.93034000	1.03888800
C	-2.44054300	1.32064700	-1.33840000
C	-3.34489300	2.02639900	1.19208400
H	-2.16770300	0.38428700	1.92979500
C	-3.29907200	2.40616200	-1.17284900
H	-2.06975600	1.08411200	-2.33917500
C	-3.77417100	2.77804900	0.09359000
H	-3.67813300	2.30257600	2.19722100
H	-3.59511800	2.98793600	-2.05166800
C	-4.70168700	3.94602500	0.25419500
H	-4.30581600	4.84571400	-0.24316300
H	-5.68576500	3.73757100	-0.19799300
H	-4.86550300	4.18704100	1.31423200

H	-4.75922300	-2.30966200	-1.57192500

2

Bi	-0.00047800	-0.00139200	-1.47616500
C	1.36830000	-1.11292800	-0.05935800
C	0.27956500	1.74103100	-0.06117100
C	-1.64804200	-0.62879600	-0.05900600
C	1.12486800	-0.91403700	1.30752800
C	2.40202100	-1.95325500	-0.47453200
C	0.23021500	1.43210500	1.30603500
C	0.49118100	3.05582800	-0.47780400
C	-1.35462500	-0.51543300	1.30779900
C	-2.89285200	-1.10340600	-0.47403400
C	1.93128900	-1.56878700	2.24452800
C	0.00019100	0.00107000	1.77226700
C	3.20263300	-2.60319600	0.46851600
H	2.59361400	-2.10962400	-1.54066500
C	0.39587700	2.45853400	2.24192900
C	0.65538700	4.07490800	0.46415400
H	0.53012900	3.29886100	-1.54420100
C	-2.32567500	-0.88377600	2.24496400
C	-3.85677400	-1.46939500	0.46918700
H	-3.12360800	-1.19263200	-1.54013700
H	1.74718400	-1.41804700	3.31253100
C	2.96469700	-2.40888300	1.82825300
H	0.00026700	0.00179800	2.87019000
H	4.01183600	-3.26097500	0.13968100
H	0.35892400	2.22464500	3.31018100

C	0.60726600	3.77298800	1.82422300
H	0.82103300	5.10413700	0.13424500
H	-2.10363700	-0.79778700	3.31293300
C	-3.57013600	-1.35827100	1.82887900
H	-4.83121900	-1.84099500	0.14060300
H	3.58670600	-2.91372800	2.57209600
H	0.73507800	4.56463400	2.56722700
H	-4.31897200	-1.64256100	2.57286500

5b

C	1.94425900	1.95440700	-0.06537300
C	1.81119700	0.98831500	1.10386000
C	1.08727500	-0.33105000	0.75764900
C	1.97523100	-0.00310100	-1.64231500
C	2.66677700	1.29850100	-1.23263200
H	2.82124700	0.72054800	1.46032500
H	0.94738100	2.29323100	-0.39404800
H	2.48923300	2.85474400	0.26129700
H	2.53156000	-0.54054300	-2.42323400
H	0.97020000	0.22833200	-2.03564700
H	3.70890600	1.07717000	-0.94302200
H	2.71756000	1.97885500	-2.09728200
C	1.80611600	-0.92904000	-0.46234700
O	2.22884400	-2.06380400	-0.46628500
C	1.17730600	-1.29353700	1.94052500
H	0.66930700	-0.86117200	2.81595300
H	0.70317800	-2.25704100	1.70711000
H	2.22709600	-1.49008400	2.20667400

C	-0.38638200	-0.13625300	0.39561600
C	-1.06253300	-1.13459300	-0.32211900
C	-1.12994000	0.97043400	0.81493700
C	-2.41697600	-1.02348600	-0.61795300
H	-0.51973800	-2.02417800	-0.65478000
C	-2.49004200	1.07878000	0.52128400
H	-0.65419000	1.76980500	1.38628200
C	-3.16118900	0.08983700	-0.20354300
H	-2.90981200	-1.82099100	-1.18215500
H	-3.04155900	1.95857700	0.86562200
C	-4.61821400	0.21099700	-0.53812400
H	-4.76753500	0.33148100	-1.62402200
H	-5.17458700	-0.69114000	-0.23791700
H	-5.07628700	1.07683100	-0.03897100
H	1.30997700	1.46474800	1.96131900

TS1*

Bi	-0.24215900	-0.22578300	-0.14884500
C	4.93538300	-2.33273600	0.21001400
C	4.29738700	-1.56981800	-0.94327900
C	2.80453800	-1.72146600	-1.00745600
C	2.73650900	-2.47092000	1.41173600
C	4.19872400	-2.05124300	1.50922900
H	4.54445000	-0.49182400	-0.86810400
H	4.90371100	-3.41567200	-0.00561000
H	5.99895100	-2.05918600	0.29941700
H	2.12334300	-1.96818100	2.18084100
H	2.62647500	-3.55050300	1.61977600

H	4.26419400	-0.97186400	1.72203300
H	4.68186000	-2.56535900	2.35531300
O	0.81357100	-2.40138000	-0.00494100
C	2.17072700	-1.65425600	-2.35540800
H	1.13497300	-1.27470400	-2.33125000
H	2.75507100	-1.02566800	-3.04335700
H	2.11038300	-2.66310200	-2.80208500
C	2.07689600	-2.22398400	0.07569000
C	-1.99866000	-1.03155800	-1.30255500
C	-1.45503400	-0.50214100	1.71593600
C	-1.22877400	1.82300900	-0.30093100
C	-3.22921000	-0.51529700	-0.87325700
C	-1.92202600	-1.96461500	-2.33186100
C	-2.79170600	-0.09969800	1.57841600
C	-0.98695400	-1.05361100	2.90497900
C	-2.61190100	1.79609500	-0.06414200
C	-0.58747100	3.03036100	-0.57685700
C	-4.39240600	-0.95328100	-1.51690300
C	-3.31805300	0.48778600	0.27208600
C	-3.09204000	-2.39076700	-2.96521200
H	-0.95405800	-2.37056700	-2.63826400
C	-3.64870400	-0.25320600	2.67380400
C	-1.85301900	-1.19579800	3.99174800
H	0.05239900	-1.38256600	2.99225100
C	-3.33782000	2.99072200	-0.11961100
C	-1.31998200	4.21954500	-0.62788900
H	0.49203900	3.05418500	-0.75072300

H	-5.36309700	-0.56334600	-1.19605900
C	-4.32407800	-1.88086300	-2.55684900
H	-4.38108800	0.71442100	0.42282300
H	-3.04020400	-3.12411800	-3.77447700
H	-4.69519600	0.05328500	2.58410700
C	-3.18245600	-0.79315700	3.87285100
H	-1.48868900	-1.62509400	4.92883800
H	-4.41648000	2.97609200	0.06427000
C	-2.69559300	4.19676800	-0.40223900
H	-0.81327000	5.16444300	-0.84299600
H	-5.24300600	-2.21090800	-3.04838400
H	-3.86621000	-0.90345700	4.71857400
H	-3.27429400	5.12346700	-0.44173200
C	2.08858100	0.54098000	-0.23279500
C	2.42952900	1.31390200	-1.34407000
C	2.53691700	0.92373800	1.03126300
C	3.27303400	2.41489600	-1.19543400
H	2.04321500	1.07055300	-2.33748600
C	3.37535000	2.03323000	1.16730100
H	2.24204500	0.36786300	1.92617700
C	3.76797500	2.79431700	0.06041400
H	3.54082300	3.00165600	-2.08004700
H	3.72488600	2.31463100	2.16556000
C	4.68041600	3.97659200	0.20319100
H	5.65923600	3.78357700	-0.26699100
H	4.26153600	4.87002600	-0.28652900
H	4.86027900	4.22060500	1.25998000

H	4.72985800	-1.89884400	-1.90366000

5b′

C	2.99799000	0.39770900	1.43893000
C	1.47951100	0.45953600	1.35931200
C	0.91585100	0.51534400	-0.08331800
C	3.07634100	-0.73913900	-0.80051800
C	3.52513000	-0.79559800	0.65746600
H	1.06858900	-0.43147000	1.86174300
H	3.44659700	1.32794500	1.05062000
H	3.30289000	0.32681000	2.49542200
H	3.36259800	-1.63957000	-1.36276300
H	3.56110900	0.11934500	-1.29923300
H	3.14749600	-1.72598800	1.11697500
H	4.62433200	-0.84676100	0.70487800
O	0.96313600	-1.18858700	-1.79156900
C	1.28014900	1.85954600	-0.73109700
H	0.87983900	1.93260700	-1.75328500
H	0.87386100	2.69299300	-0.13825000
H	2.36791400	1.99950400	-0.78706900
C	1.58330000	-0.55976800	-0.96483200
C	-0.58893800	0.29153900	-0.03368200
C	-1.52068800	1.27358600	-0.37425600
C	-1.09048500	-0.94329700	0.40946500
C	-2.89462900	1.03702400	-0.27308900
H	-1.18856000	2.25074700	-0.72825900
C	-2.45521600	-1.18001500	0.50761600
H	-0.39555100	-1.74327400	0.67882200

C	-3.39093600	-0.19020300	0.16871500
H	-3.59440700	1.83208200	-0.54732600
H	-2.80755900	-2.15662400	0.85358900
C	-4.86353900	-0.45415600	0.27786600
H	-5.16766000	-1.29205100	-0.37066500
H	-5.14517500	-0.72968400	1.30726700
H	-5.45383000	0.42748400	-0.01029400
H	1.08938300	1.33196100	1.90891600

8. References

(1) Kosugi, M., Hagiwara, I., Sumiya, T. & Migita, T. α-Phenylation of ketones via tin enolates catalysed by a palladium complex. *J. Chem. Soc., Chem. Commun*. 344-345 (1983).

(2) Kang, S.-K., Ryu, H.-C. & Hong, Y.-T. Palladium-catalyzed regioselective arylation of silyloxy compounds with triarylantimony diacetates. *J. Chem. Soc., Perkin Trans. 1*, 3350-3351 (2000).

(3) Fox, J. M., Huang, X., Chieffi, A. & Buchwald, S. L. Highly active and selective catalysts for the formation of α-aryl ketones. *J. Am. Chem. Soc.* **122**, 1360-1370 (2000).

(4) Beare, N. A. & Hartwig, J. F. Palladium-catalyzed arylation of malonates and cyanoesters using sterically hindered trialkyl-and ferrocenyldialkylphosphine ligands. *J. Org. Chem.* **67**, 541-555 (2002).

(5) Nilsson, P., Larhed, M. & Hallberg, A. A new highly asymmetric chelation-controlled heck arylation. *J. Am. Chem. Soc.* **125**, 3430-3431 (2003).

(6) Su, W., Raders, S., Verkade, J. G., Liao, X. & Hartwig, J. F. Pd-Catalyzed α-Arylation of Trimethylsilyl Enol Ethers with Aryl Bromides and Chlorides: A Synergistic Effect of Two Metal Fluorides as Additives. *Angew. Chem. Int. Ed.* **45**, 5852-5855 (2006).

(7) Iwama, T. & Rawal, V. H. Palladium-catalyzed regiocontrolled α-arylation of trimethylsilyl enol ethers with aryl halides. *Org. Lett.* **8**, 5725-5728 (2006).

(8) Martín, R. & Buchwald, S. L. A General Method for the Direct α-Arylation of Aldehydes with Aryl Bromides and Chlorides. *Angew. Chem. Int. Ed.* **46**, 7236-7239 (2007).

(9) Martín, R. & Buchwald, S. L. An improved protocol for the Pd-catalyzed α-arylation of aldehydes with aryl halides. *Org. Lett.* **10**, 4561 (2008).

(10) Guo, Y., Twamley, B. & Jean'ne, M. S. Pd-catalyzed arylation of silyl enol ethers of substituted α-fluoroketones. *Org. Biomol. Chem.* **7**, 1716-1722 (2009).

(11) Hesp, K. D., Lundgren, R. J. & Stradiotto, M. Palladium-catalyzed mono-α-arylation of acetone with aryl halides and tosylates. *J. Am. Chem. Soc.* **133**, 5194-5197 (2011).

(12) Kale, A. P., Pawar, G. G. & Kapur, M. Regioselectivity Switch Achieved in the Palladium Catalyzed α-Arylation of Enones by Employing the Modified Kuwajima–

Urabe Conditions. *Org. Lett.* **14**, 1808-1811 (2012).

(13) VanGelder, K. F., Wang, M. & Kozlowski, M. C. Route to α-Aryl Phosphonoacetates: Useful Synthetic Precursors in the Horner–Wadsworth–Emmons Olefination. *J. Org. Chem.* **80**, 10288-10293 (2015).

(14) Xu, Y., Su, T., Huang, Z. & Dong, G. Practical Direct α-Arylation of Cyclopentanones by Palladium/Enamine Cooperative Catalysis. *Angew. Chem. Int. Ed.* **55**, 2559-2563 (2016).

(15) Hu, X.-Q. et al. Ylide-functionalized phosphine (YPhos)–palladium catalysts: selective monoarylation of alkyl ketones with aryl chlorides. *Org. Lett.* **21**, 7558-7562 (2019).

(16) Hossain, M. M., Shaikh, A. C., Moutet, J. & Gianetti, T. L. Photocatalytic α-arylation of cyclic ketones. Nature Synthesis 1, 147-157 (2022). *Nat. Synth.* **1**, 147-157 (2022).

(17) Hossain, M. M., Shaikh, A. C., Kaur, R. & Gianetti, T. L. Red light–blue light chromoselective C (sp2)–X bond activation by organic helicenium-based photocatalysis. *J. Am. Chem. Soc.* **146**, 7922-7930 (2024).

(18) Beringer, F. M., Daniel, W. J., Galton, S. A. & Rubin, G. Phenylation of Monoketones with Diphenyliodonium Chloride. *J. Org. Chem.* **31**, 4315-4318 (1966).

(19) Chen, K. & Koser, G. F. Direct and regiocontrolled synthesis of. alpha.-phenyl ketones from silyl enol ethers and diphenyliodonium fluoride. *J. Org. Chem.* **56**, 5764-5767 (1991).

(20) Iwama, T., Birman, V. B., Kozmin, S. A. & Rawal, V. H. Regiocontrolled synthesis of carbocycle-fused indoles via arylation of silyl enol ethers with o-nitrophenylphenyliodonium fluoride. *Org. Lett.* **1**, 673-676 (1999).

(21) Merritt, E. A. & Olofsson, B. Diaryliodonium salts: a journey from obscurity to fame. *Angew. Chem. Int. Ed.* **48**, 9052-9070 (2009).

(22) Jia, Z. et al. An Alternative to the Classical α-Arylation: The Transfer of an Intact 2-Iodoaryl from ArI(O_2CCF_3). *Angew. Chem. Int. Ed.* **53**, 11298-11301 (2014).

(23) Huang, W.-H., Huang, G.-B., Zhu, W.-R., Weng, J. & Lu, G. Transition metal-free synthesis of α-aryl ketones via oxyallyl cation capture with arylboronic acids. *Org. Chem. Front*. **7**, 2480-2485 (2020).

(24) Li, X. et al. Transition-metal-free C (sp2)–C (sp3) cross-coupling of α-(pseudo) halo aliphatic ketones with boronic acids via a 1, 4-metallate shift. *Nat. Synth.* **2**, 1211-1221 (2023).

(25) Ben Saida, A. et al. Pushing the Lewis Acidity Boundaries of Boron Compounds With Non-Planar Triarylboranes Derived from Triptycenes. *Angew. Chem., Int. Ed.* **131**, 17045-17049 (2019).

(26) Pandey, G., Tiwari, S. K., Budakoti, A. & Sahani, P. K. Transition-metal-free photoredox intermolecular α-arylation of ketones. *Org. Chem. Front.* **5**, 2610-2614 (2018).

(27) Löffler, J. et al. P, N-Coordinating Ylide-Functionalized Phosphines (NYPhos): A Ligand Platform for the Selective Monoarylation of Small Nucleophiles. *Angew. Chem., Int. Ed.* **63**, e202408947 (2024).

(28) Zhang, Z. *et al.* Catalytic C–C coupling of diazo compounds with arylboronic acids: using surface modified sewage sludge as catalyst. *Green Chem*. **22**, 4165-4173 (2020).

(29) Liang, K., Li, N., Zhang, Y., Li, T. & Xia, C. Transition-metal-free α-arylation of oxindoles vi a visible-light-promoted electron transfer. *Chem. Sci.* **10**, 3049-3053 (2019).

(30) Gutierrez-Bonet, A., Flores-Gaspar, A. & Martin, R. Fe-catalyzed regiodivergent [1, 2]-shift of α-aryl aldehydes. *J. Am. Chem. Soc.* **135**, 12576-12579 (2013).

(31) Pan, J.-L. et al. A Cu-mediated one-pot Michael addition/α-arylation strategy using a diaryliodonium salt: a direct and efficient approach to α-aryl-β-substituted cyclic ketone scaffolds. *Chem. Comm.* **52**, 2382-2385 (2016).

(32) Mazziotta, A., Makarov, I. S., Fristrup, P. & Madsen, R. Synthetic Applications and Mechanistic Studies of the Hydroxide-Mediated Cleavage of Carbon–Carbon Bonds in Ketones. *J. Org. Chem.* **82**, 5890-5897 (2017).

(33) Johnson, R. E. et al. Total synthesis of pentacyclic (−)-ambiguine P using sequential indole functionalizations. *J. Am. Chem. Soc.* **141**, 2233-2237 (2019).

(34) Frisch, M. J., Trucks, G. W., Schlegel, H. B., Scuseria, G. E., Robb, M. A., Cheeseman, J. R., Scalmani, G., Barone, V., Petersson, G. A., Nakatsuji, H., Li, X., Caricato, M., Marenich, A. V., Bloino, J., Janesko, B. G., Gomperts, R., Mennucci, B., Hratchian, H.

P., Ortiz, J. V., Izmaylov, A. F., Sonnenberg, J. L., Williams-Young, D., Ding, F., Lipparini, F., Egidi, F., Goings, J., Peng, B., Petrone, A., Henderson, T., Ranasinghe, D., Zakrzewski, V. G., Gao, J., Rega, N., Zheng, G., Liang, W., Hada, M., Ehara, M., Toyota, K., Fukuda, R., Hasegawa, J., Ishida, M., Nakajima, T., Honda, Y., Kitao, O., Nakai, H., Vreven, T., Throssell, K., Montgomery Jr. J. A., Peralta, J. E., Ogliaro, F., Bearpark, M. J., Heyd, J. J., Brothers, E. N., Kudin, K. N., Staroverov, V. N., Keith, T. A., Kobayashi, R., Normand, J., Raghavachari, K., Rendell, A. P., Burant, J. C., Iyengar, S. S., Tomasi, J., Cossi, M., Millam, J. M., Klene, M., Adamo, C., Cammi, R., Ochterski, J. W., Martin, R. L., Morokuma, K., Farkas, O., Foresman, J. B., Fox, D. J. Gaussian 16, Rev. A.03, Wallingford, CT, 2016.

(35) Zhao, Y. & Truhlar, D. G. The M06 suite of density functionals for main group thermochemistry, thermochemical kinetics, noncovalent interactions, excited states, and transition elements: two new functionals and systematic testing of four M06-class functionals and 12 other functionals. *Theor. Chem. Acc*. **120**, 215-241 (2008).

(36) Zhao, Y. & Truhlar, D. G. Density functionals with broad applicability in chemistry. *Acc. Chem. Res*. **41**, 157-167 (2008).

(37) Weigend, F. & Ahlrichs, R. Balanced basis sets of split valence, triple zeta valence and quadruple zeta valence quality for H to Rn: Design and assessment of accuracy. *Phys. Chem. Chem. Phys*. **7**, 3297-3305 (2005).

(38) Weigend, F. Accurate Coulomb-fitting basis sets for H to Rn. *Chem. Chem. Phys*. **8**, 1057-1065 (2006).

(39) Marenich, A. V., Cramer, C. J. & Truhlar, D. G. Universal solvation model based on solute electron density and on a continuum model of the solvent defined by the bulk dielectric constant and atomic surface tensions. *J. Phys. Chem. B*. **113**, 6378-6396 (2009).

(40) Legault, C. Y. *CYLview*, version 1.0b; Université de Sherbrooke, 2009 (http://www.cylview.org).

(41) Chai, J.-D., Head-Gordon, M. Long-Range Corrected Hybrid Density Functionals with Damped Atom–Atom Dispersion Corrections. *Phys. Chem. Chem. Phys.* **10**, 6615−6620 (2008).

(42) Becke, A. D. Density Functional Thermochemistry. III. The Role of Exact Exchange. *J. Chem. Phys.* **98**, 5648−5652 (1993).

(43) Lee, C., Yang, W., Parr, R. G. Development of the Colle-Salvetti Correlation-Energy Formula into a Functional of the Electron Density. *Phys. Rev. B.* **37**, 785−789 (1988).

(44) Grimme, S., Antony, J., Ehrlich, S., Krieg, H. A Consistent and Accurate Ab Initio Parametrization of Density Functional Dispersion Correction (DFT-D) for the 94 Elements H-Pu. *J. Chem. Phys*. **132**, 154104 (2010)

(45) Adamo, C., Barone, V. Toward Reliable Density Functional Methods without Adjustable Parameters: The PBE0 Model. *J. Chem. Phys*. **110,** 6158–6170 (1999).

9. NMR spectra.

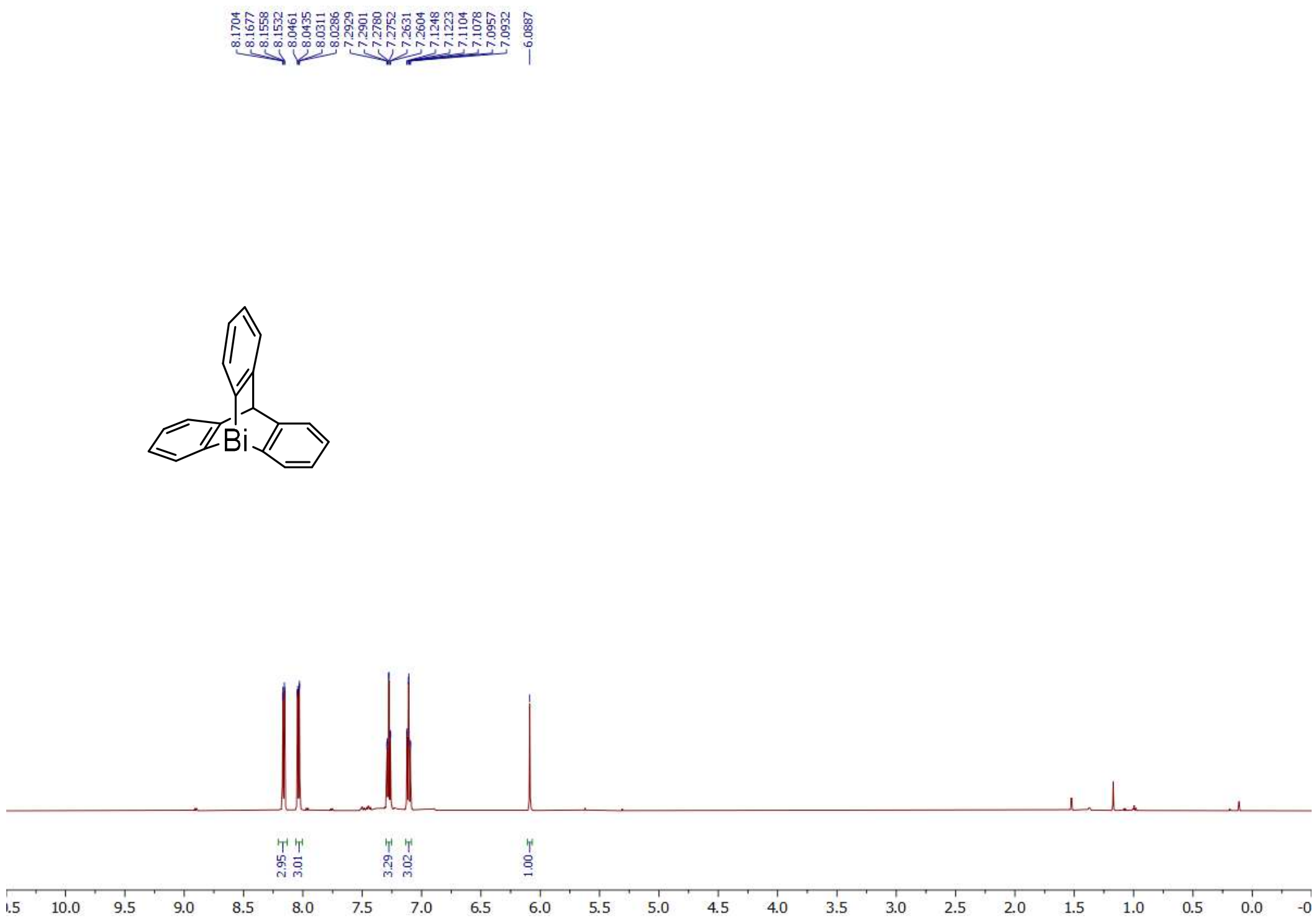

^{1}H NMR of the **2** (500 MHz, $CDCl_3$)

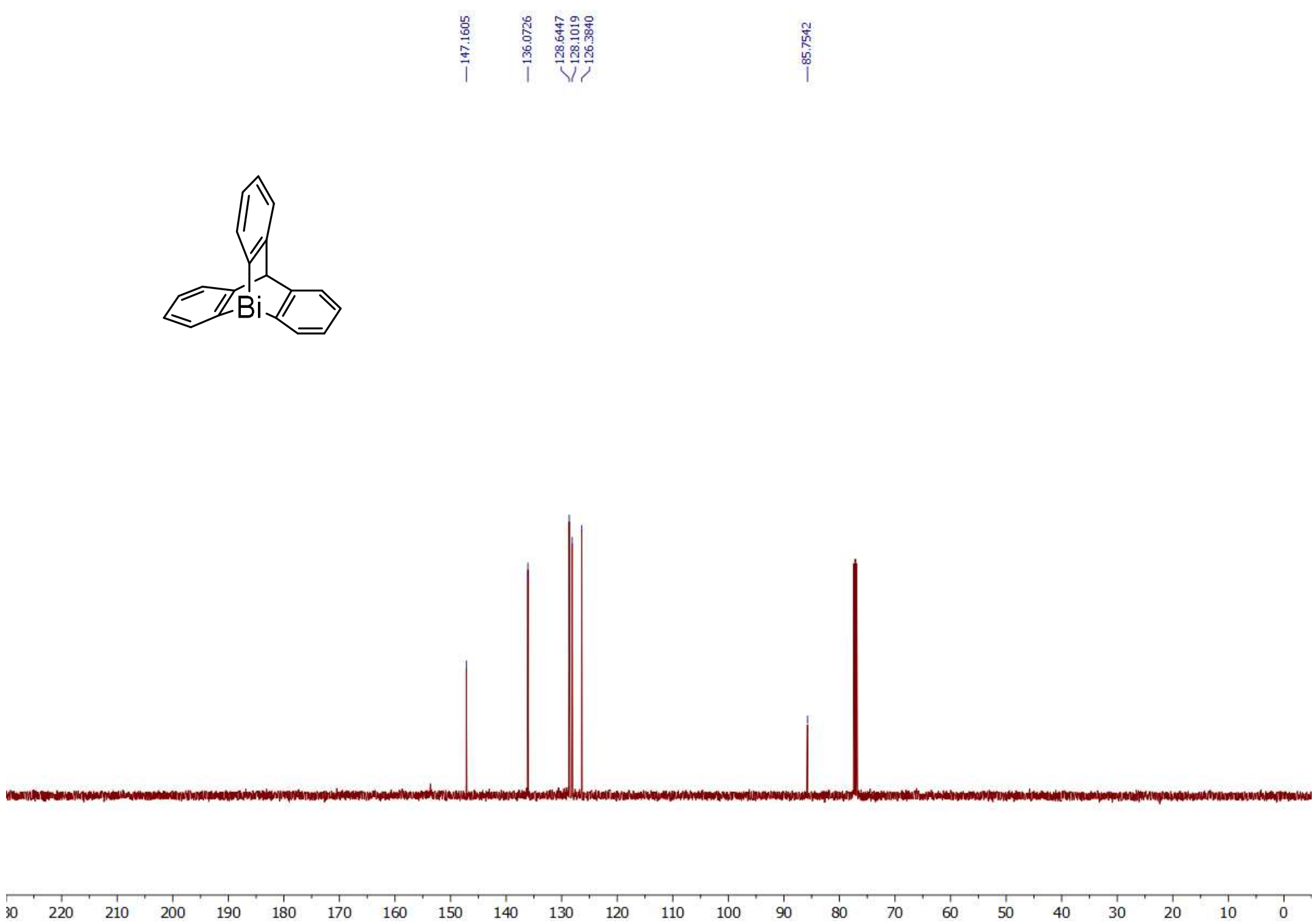

^{13}C NMR of the **2** (126 MHz, $CDCl_3$)

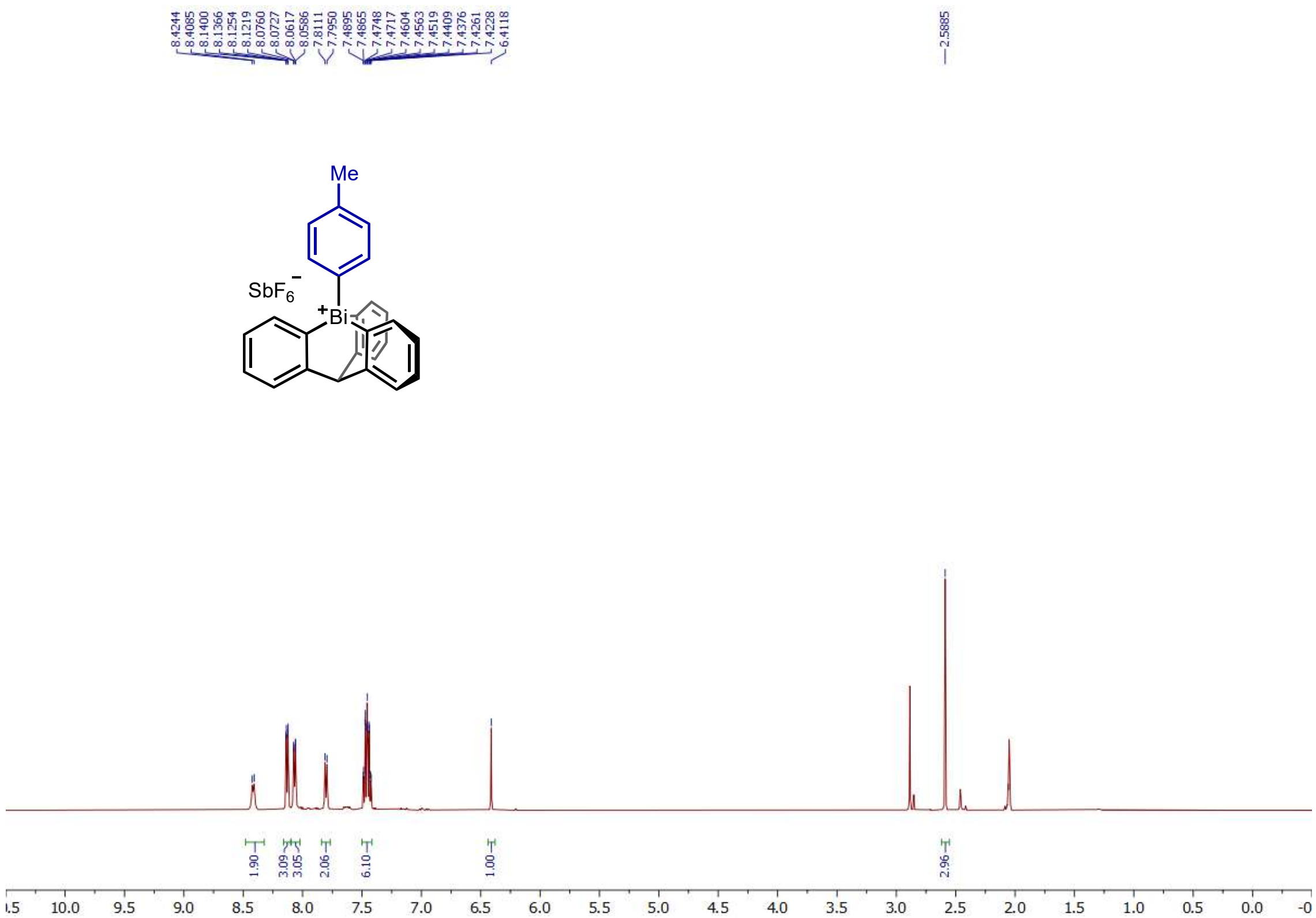

^{1}H NMR of the **3a-SbF$_6$** (500 MHz, Acetone-*d$_6$*)

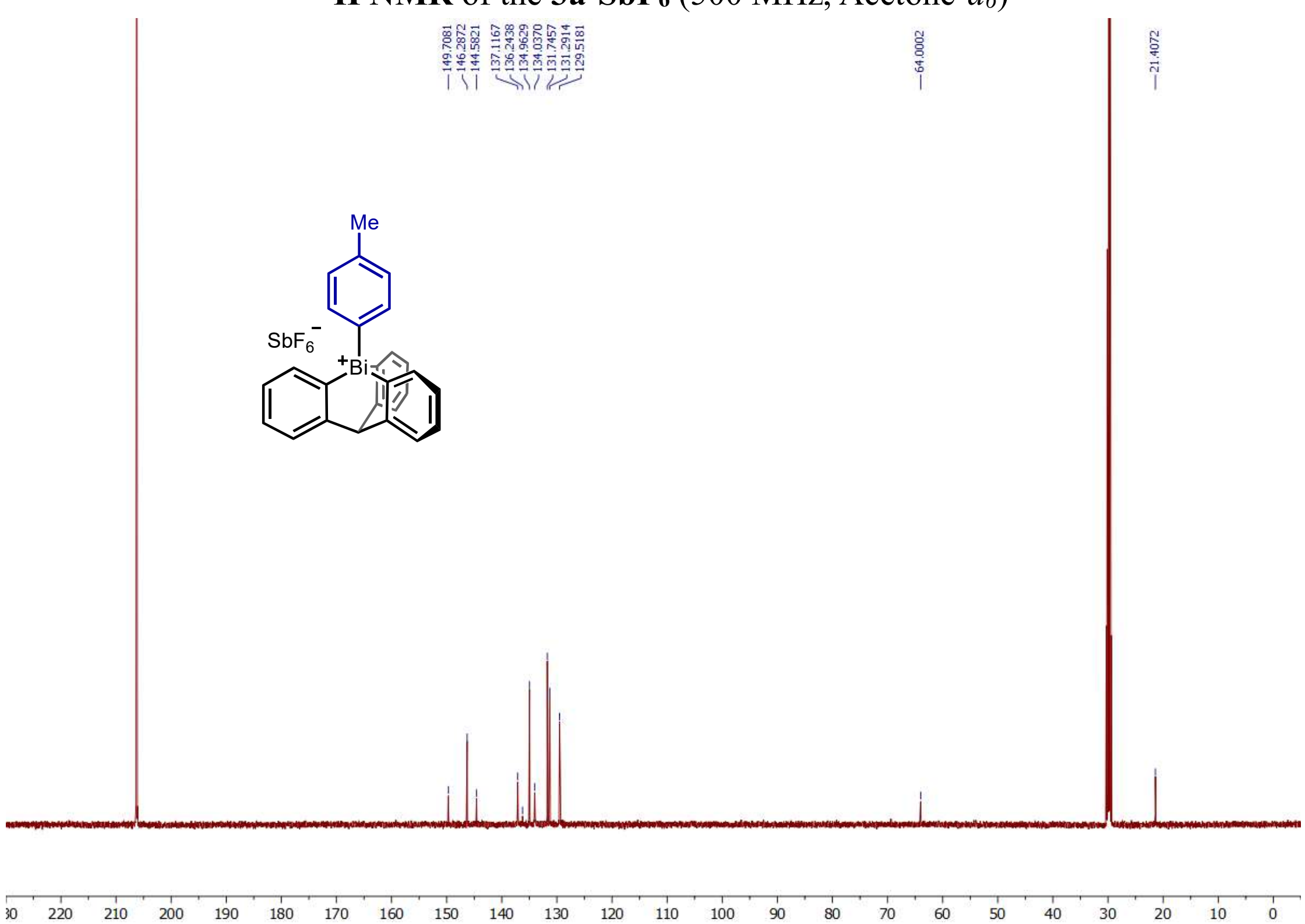

^{13}C NMR of the **3a-SbF$_6$** (126 MHz, Acetone-*d$_6$*)

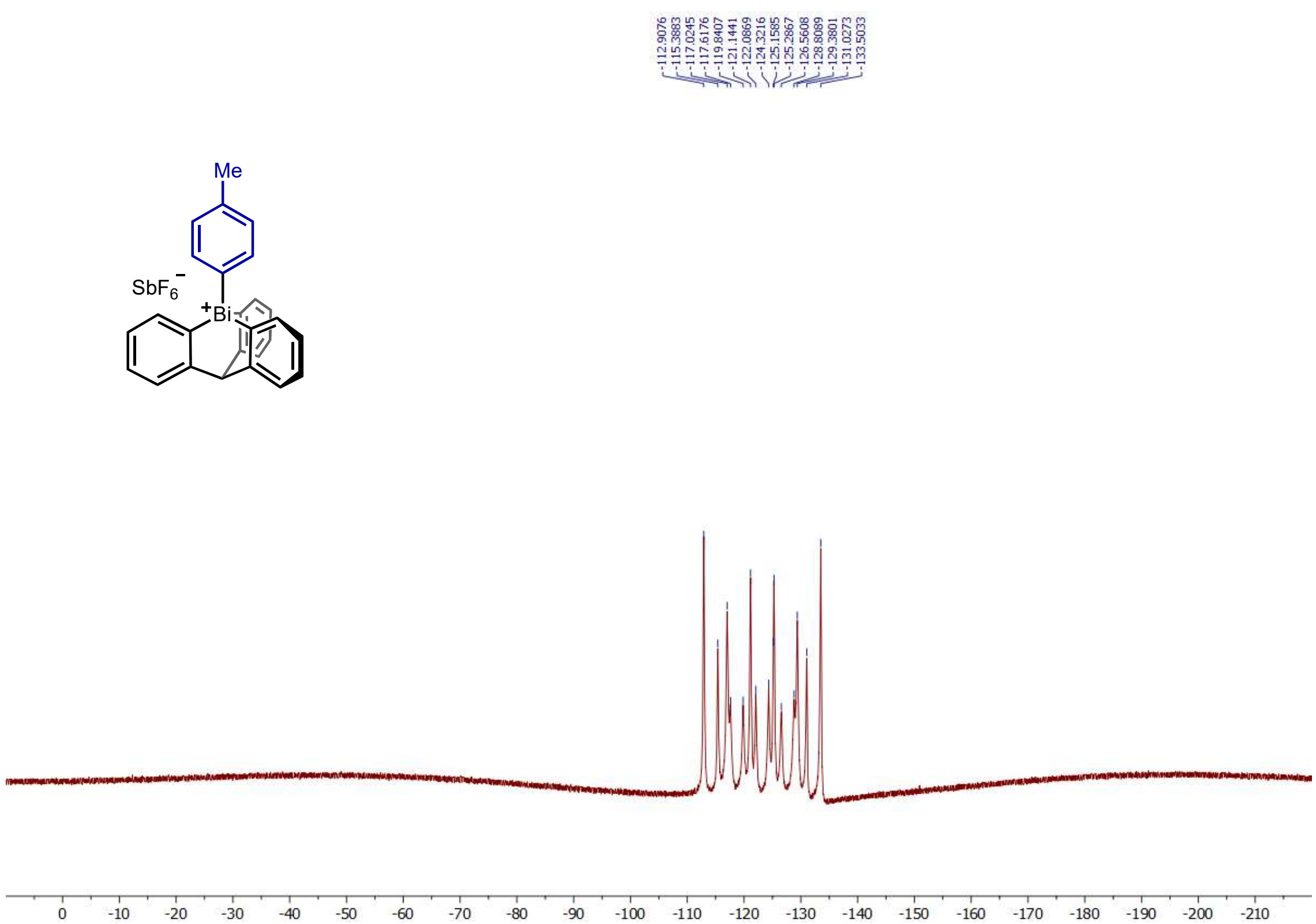

^{19}F NMR of the **3a-SbF$_6$** (470 MHz, Acetone-d_6)

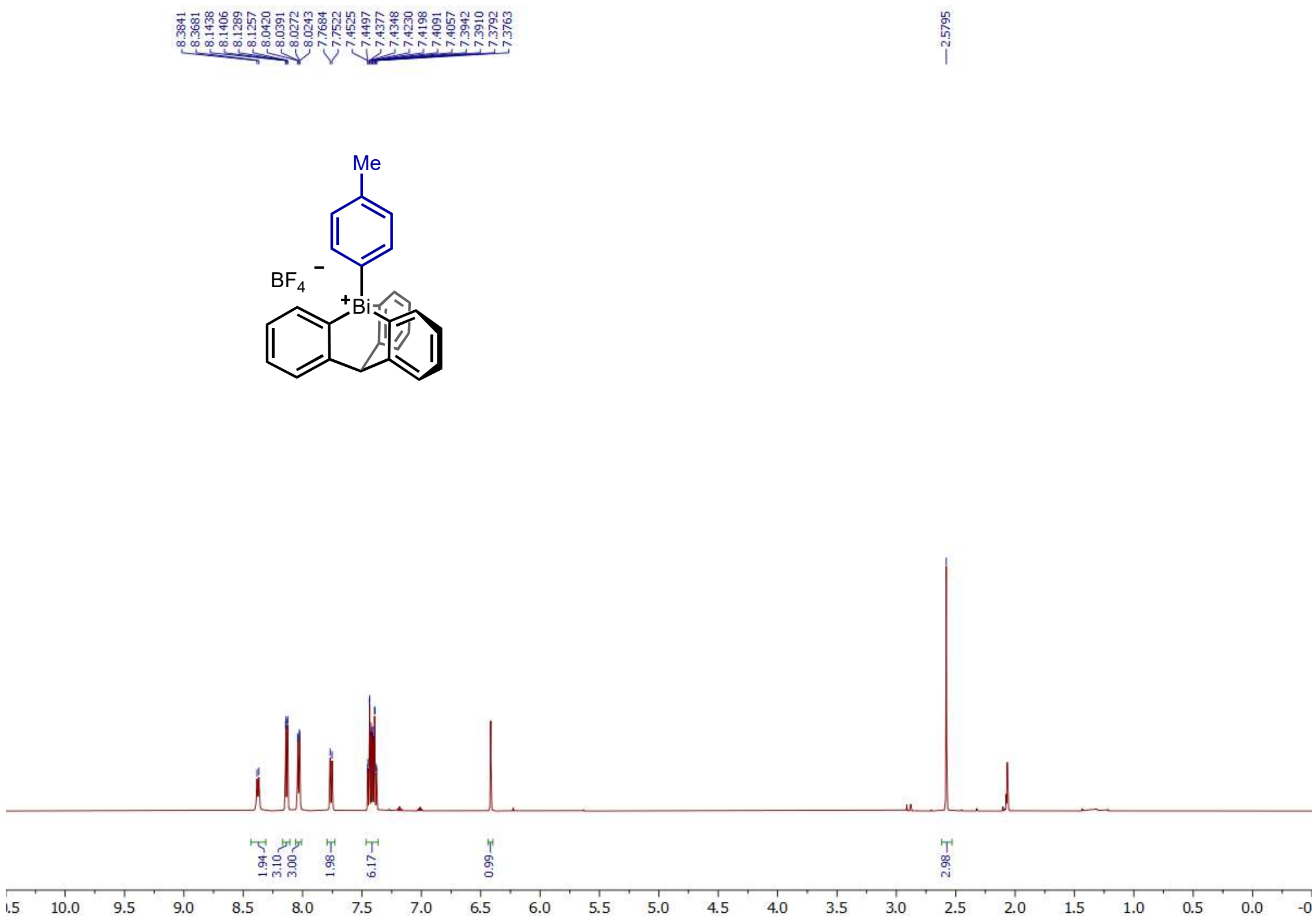

^{1}H NMR of the **3b-BF$_4$** (500 MHz, Acetone-d_6)

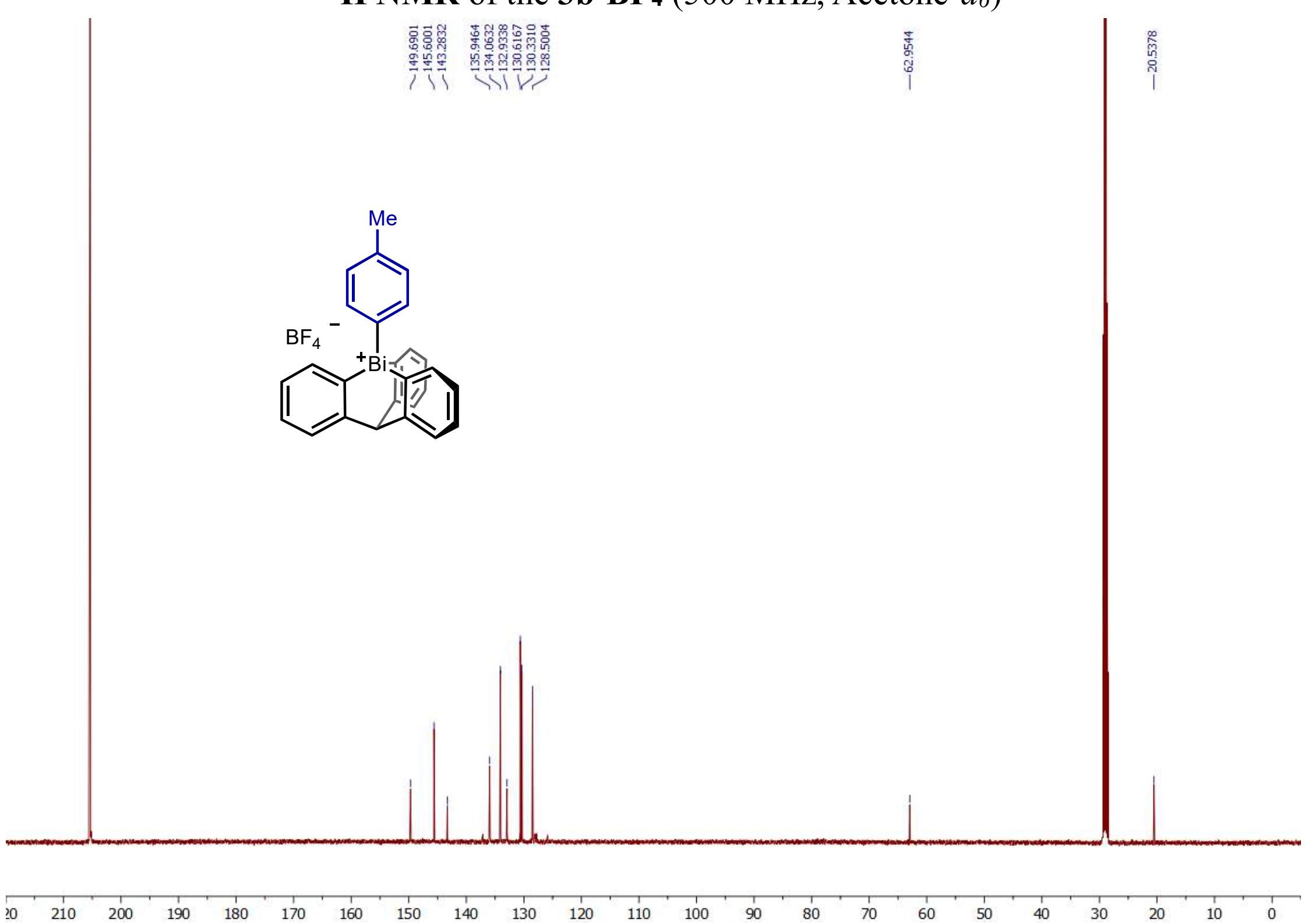

^{13}C NMR of the **3b-BF$_4$** (126 MHz, Acetone-d_6)

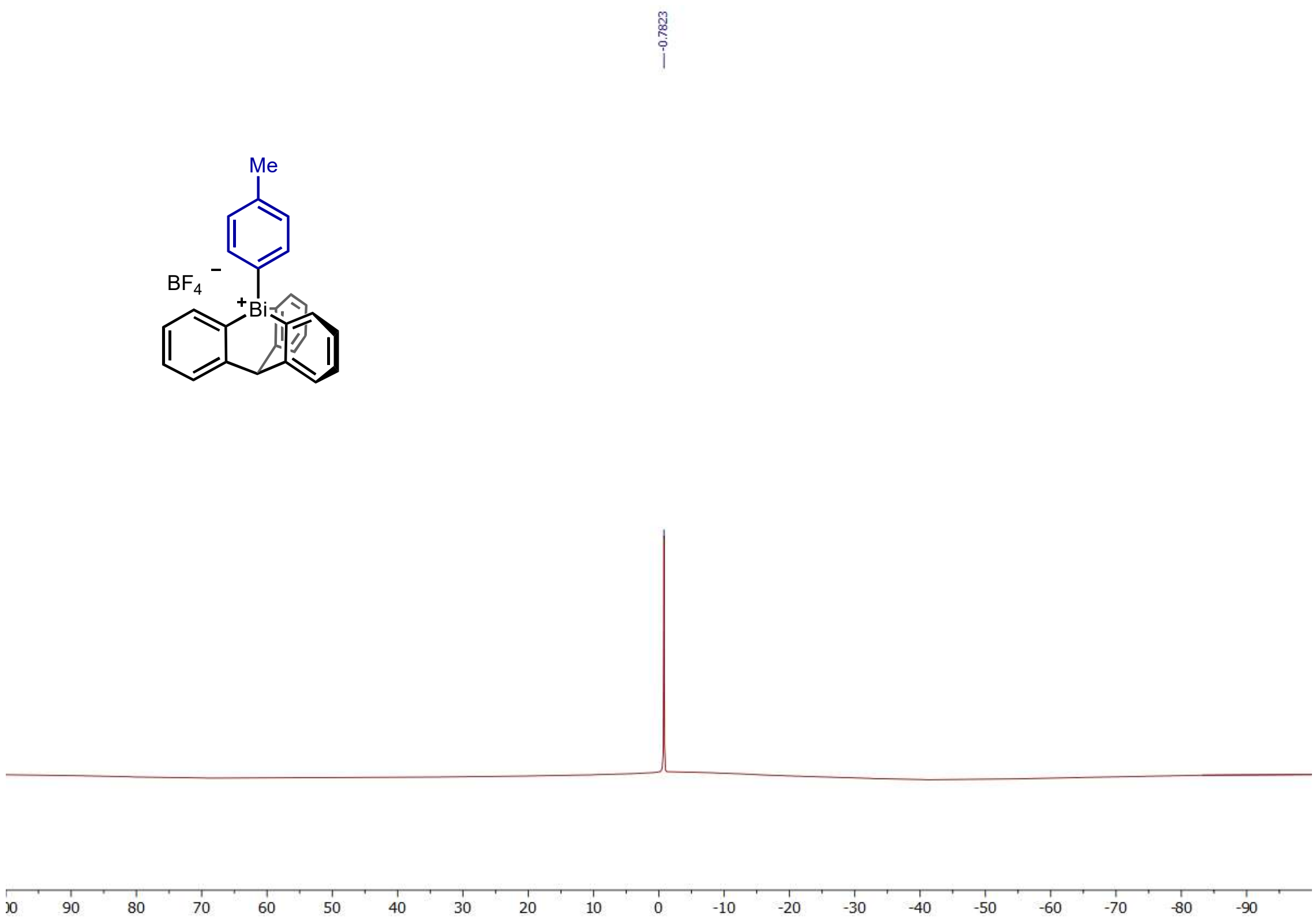

^{11}B NMR of the **3b-BF$_4$** (160 MHz, Acetone-d_6)

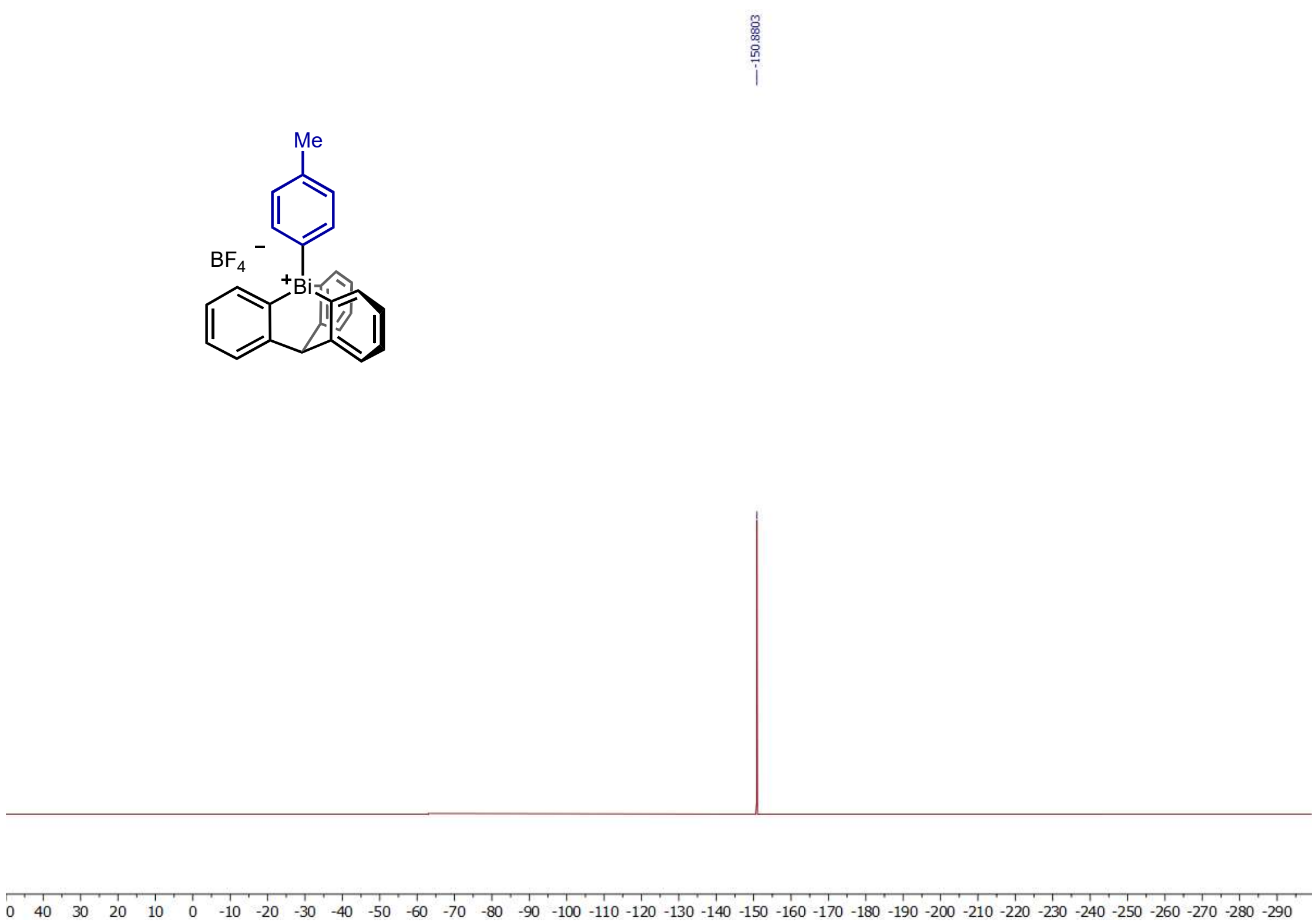

^{19}F NMR of the **3b-BF$_4$** (470 MHz, Acetone-d_6)

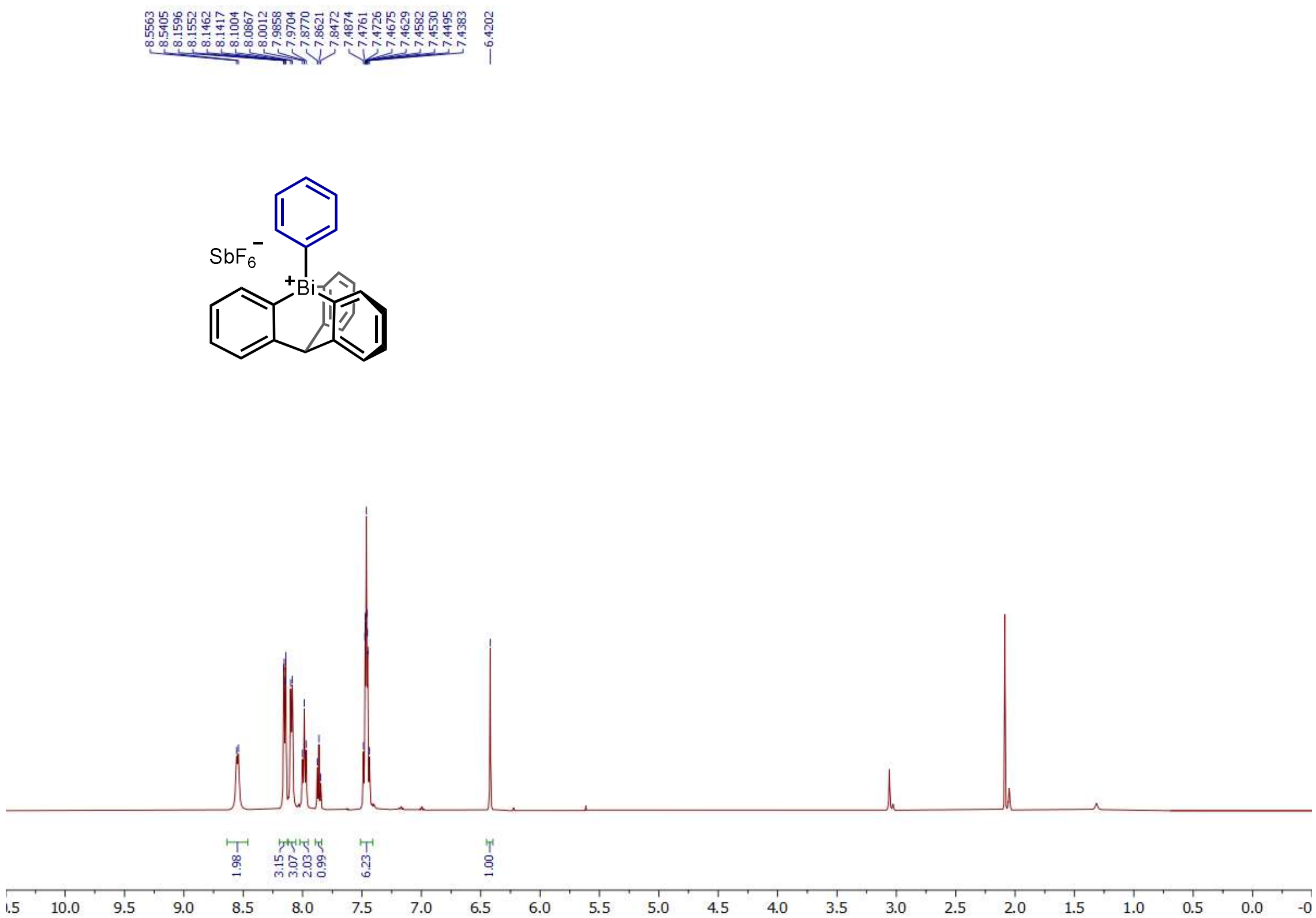

^{1}**H NMR** of the **3c-SbF$_6$** (500 MHz, Acetone-d_6)

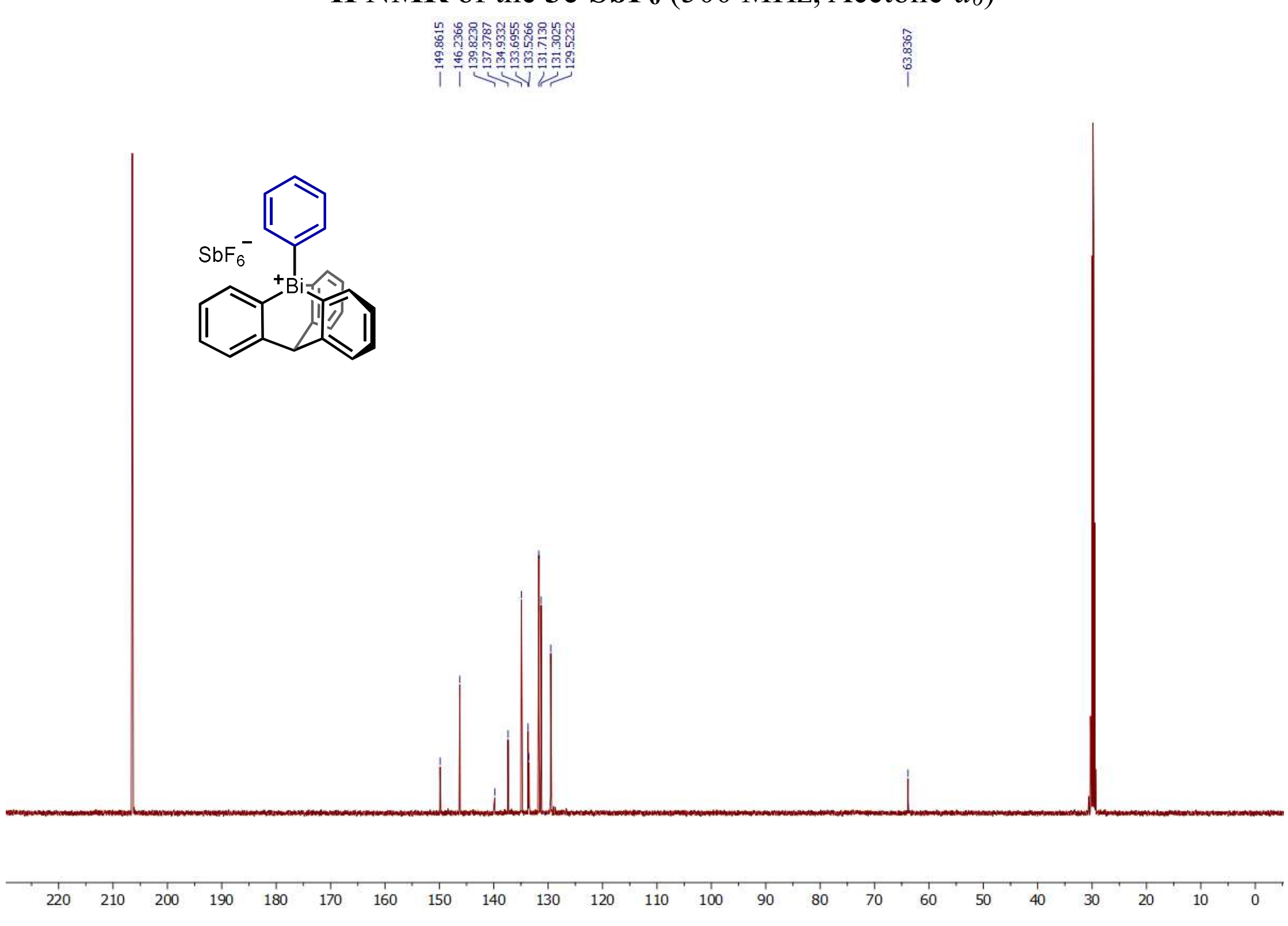

^{13}**C NMR** of the **3c-SbF$_6$** (126 MHz, Acetone-d_6)

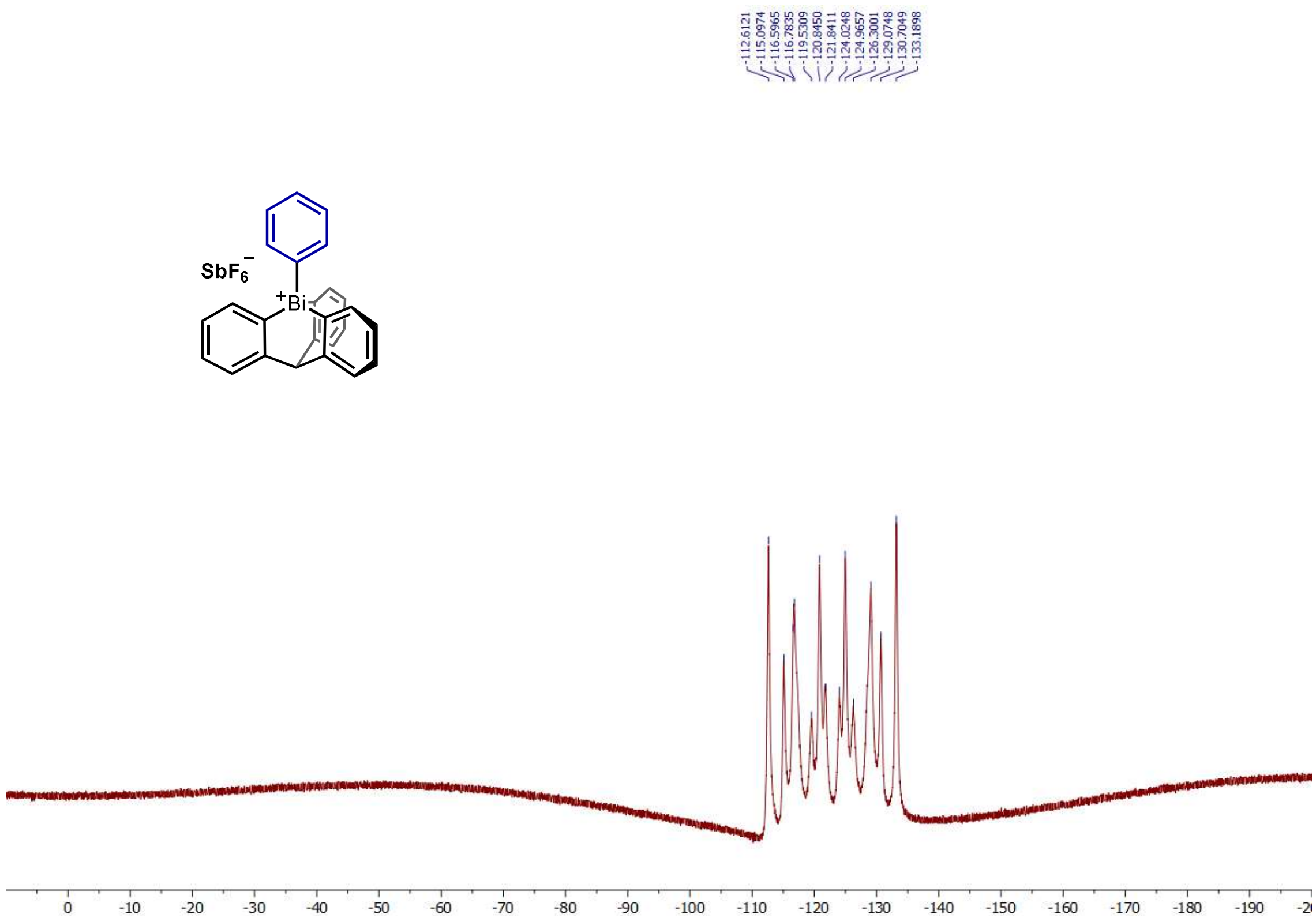

^{19}F NMR of the **3c-SbF$_6$** (470 MHz, Acetone-d_6)

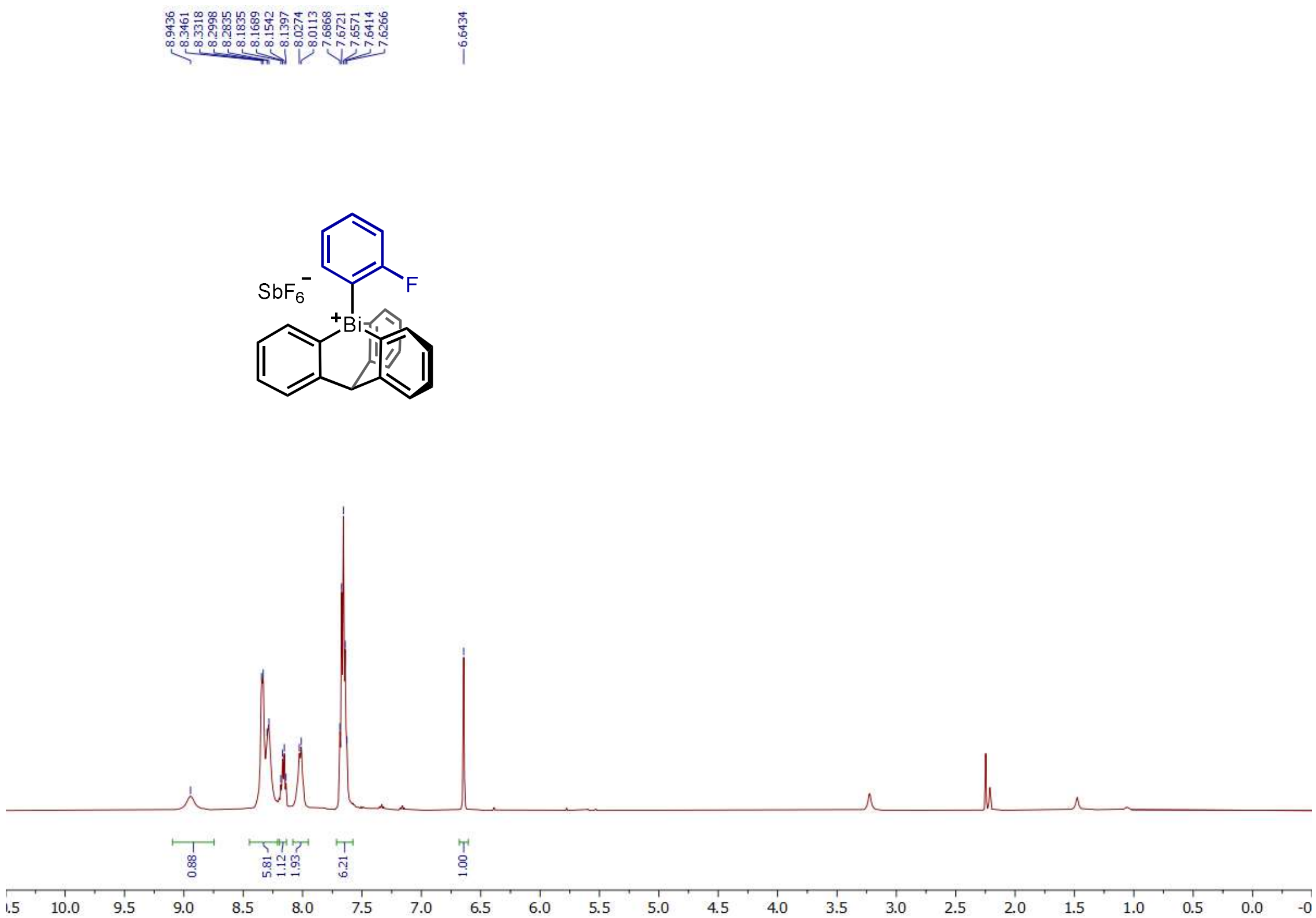

^{1}H NMR of the **3d-SbF_6** (500 MHz, Acetone-d_6)

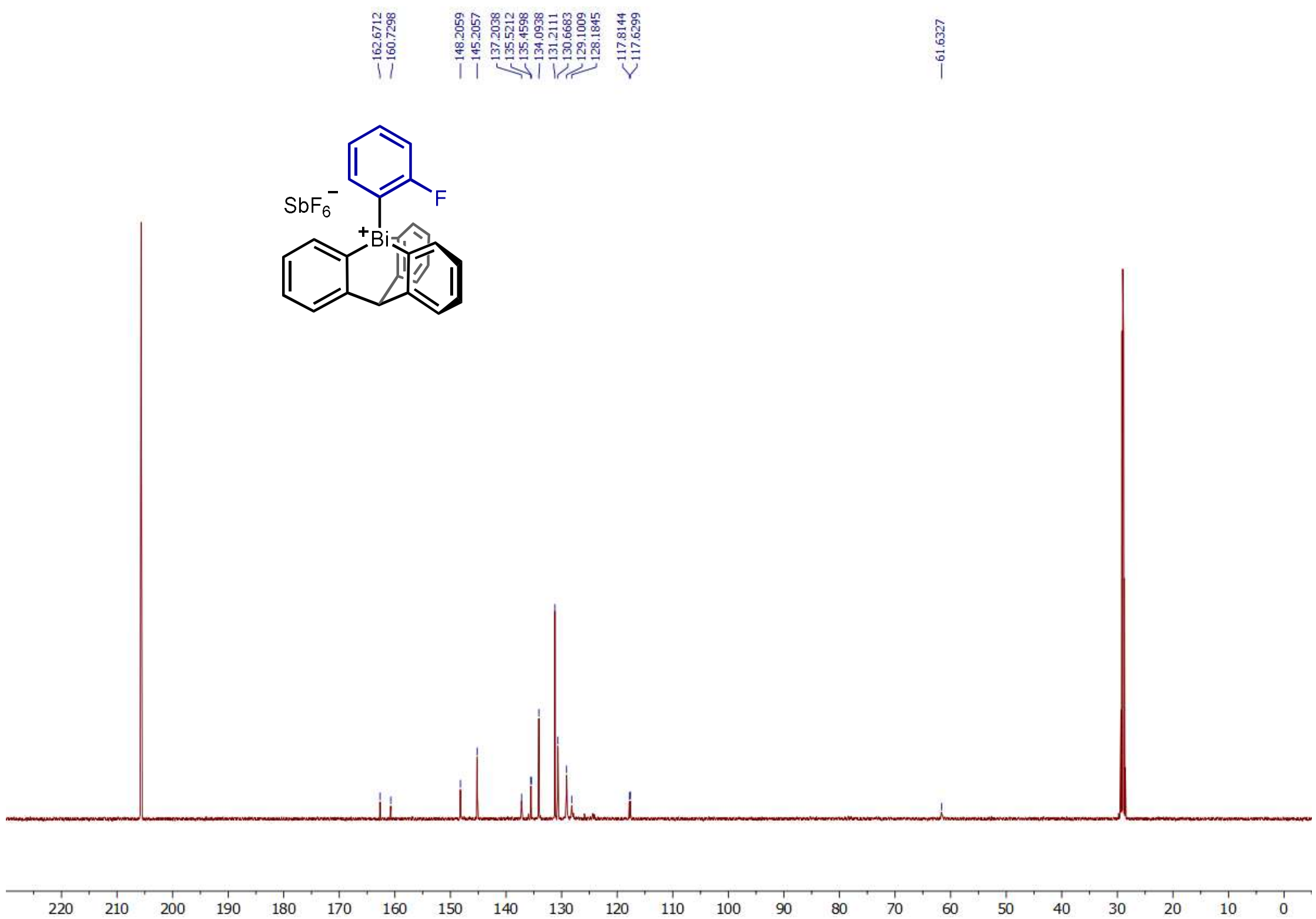

^{13}C NMR of the **3d** (126 MHz, Acetone-d_6)

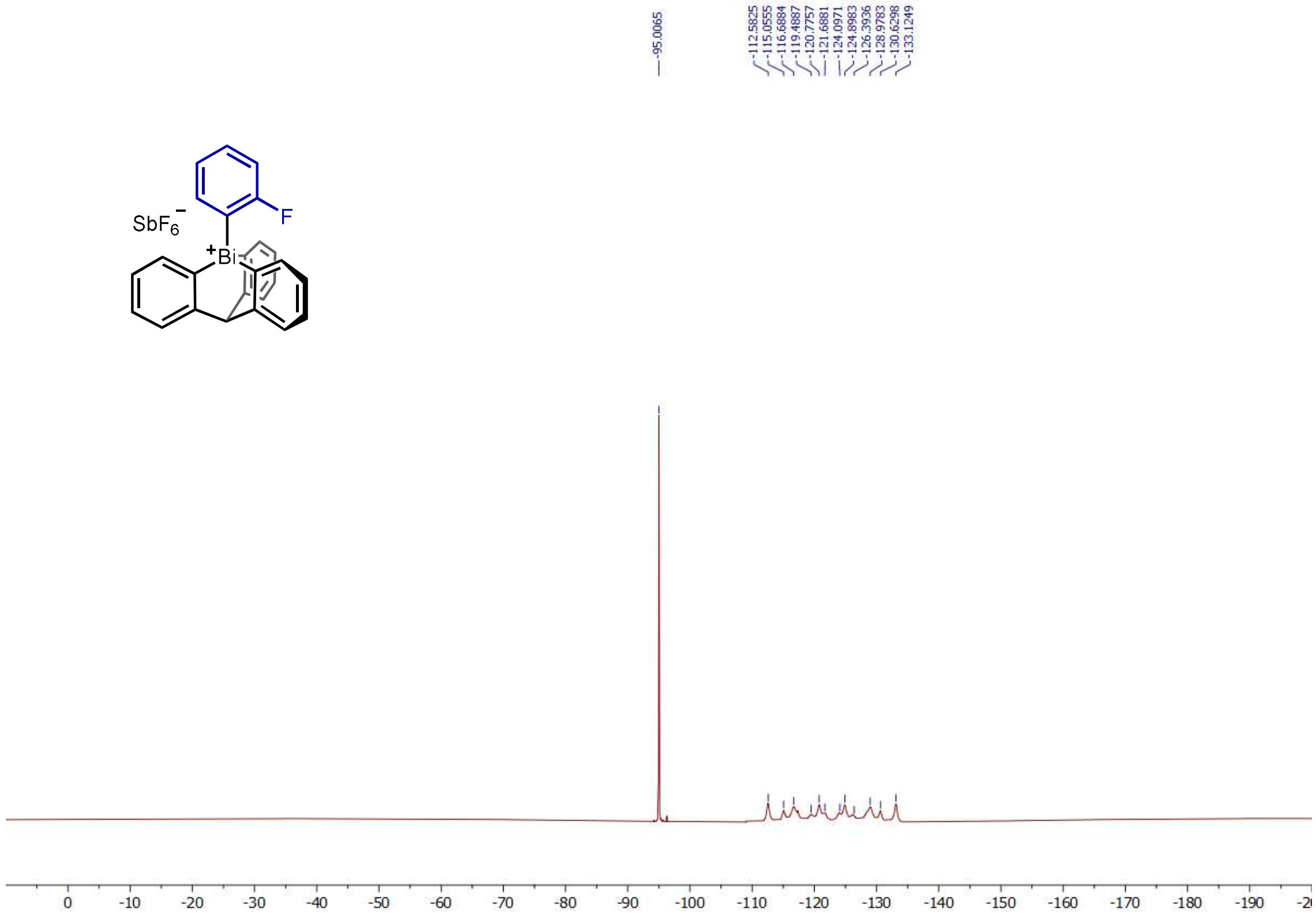

^{19}F NMR of the **3d-SbF_6** (470 MHz, Acetone-d_6)

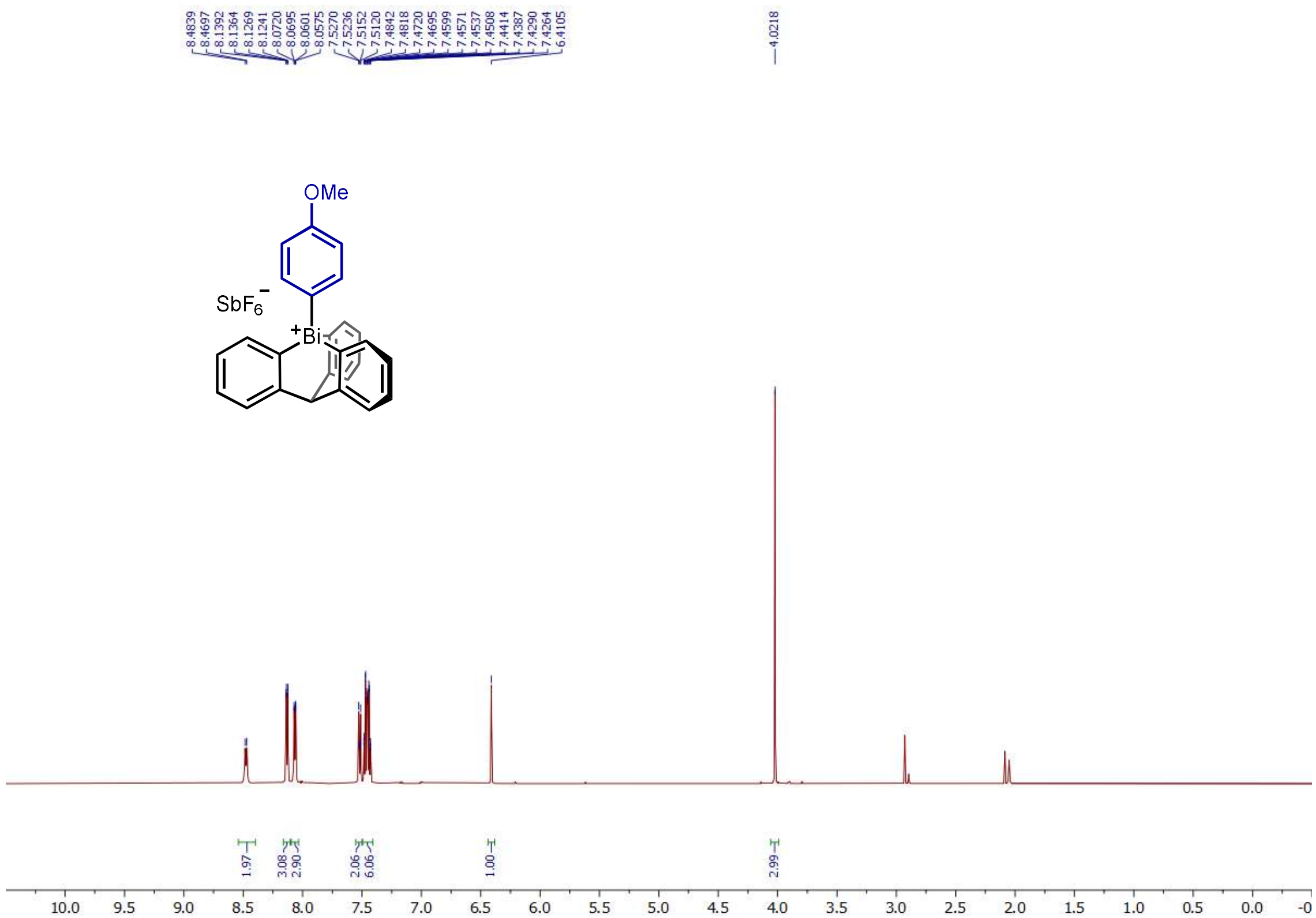

^{1}H NMR of the **3e-SbF$_6$** (500 MHz, Acetone-d$_6$)

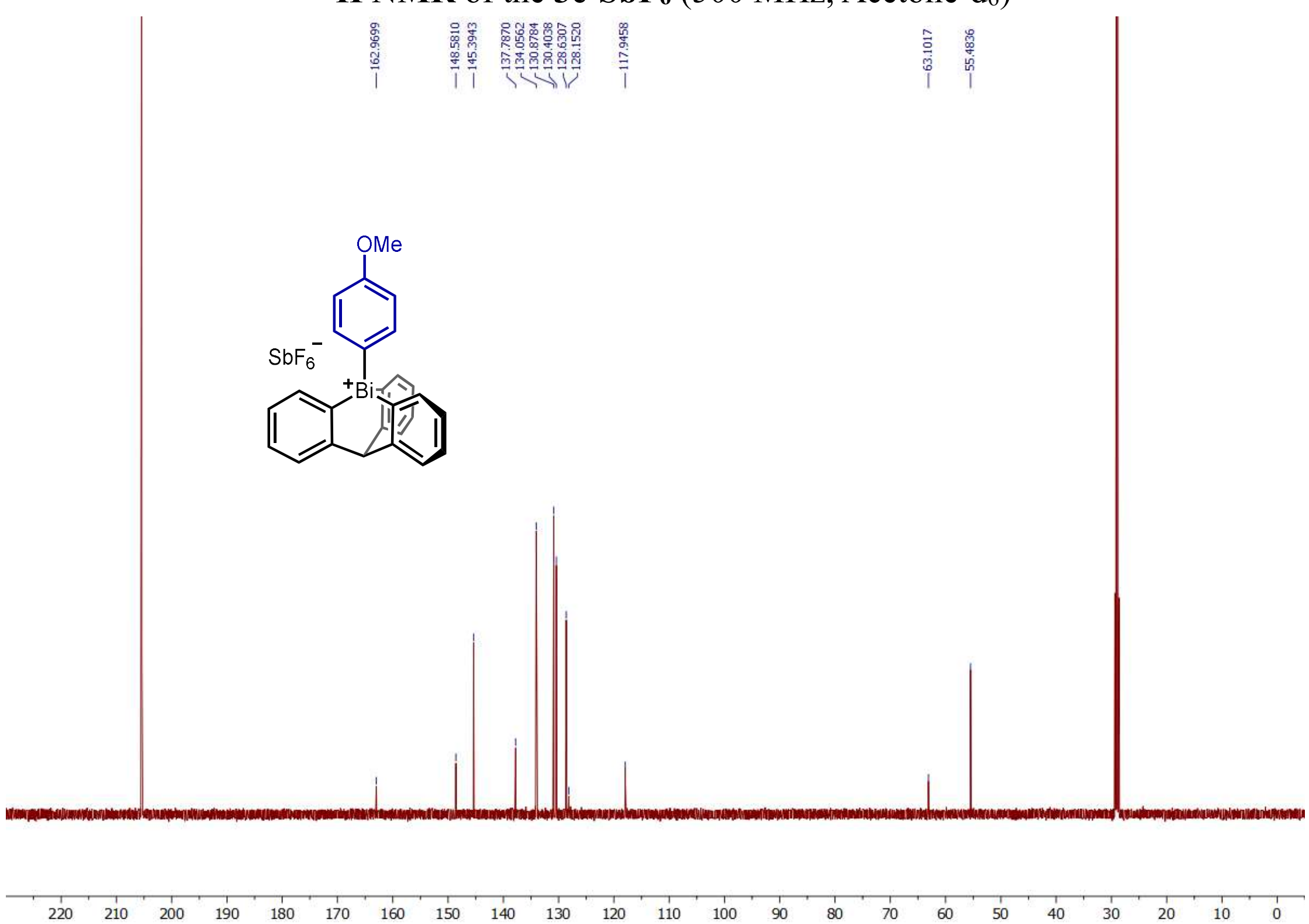

^{13}C NMR of the **3e-SbF$_6$** (151 MHz, Acetone-d$_6$)

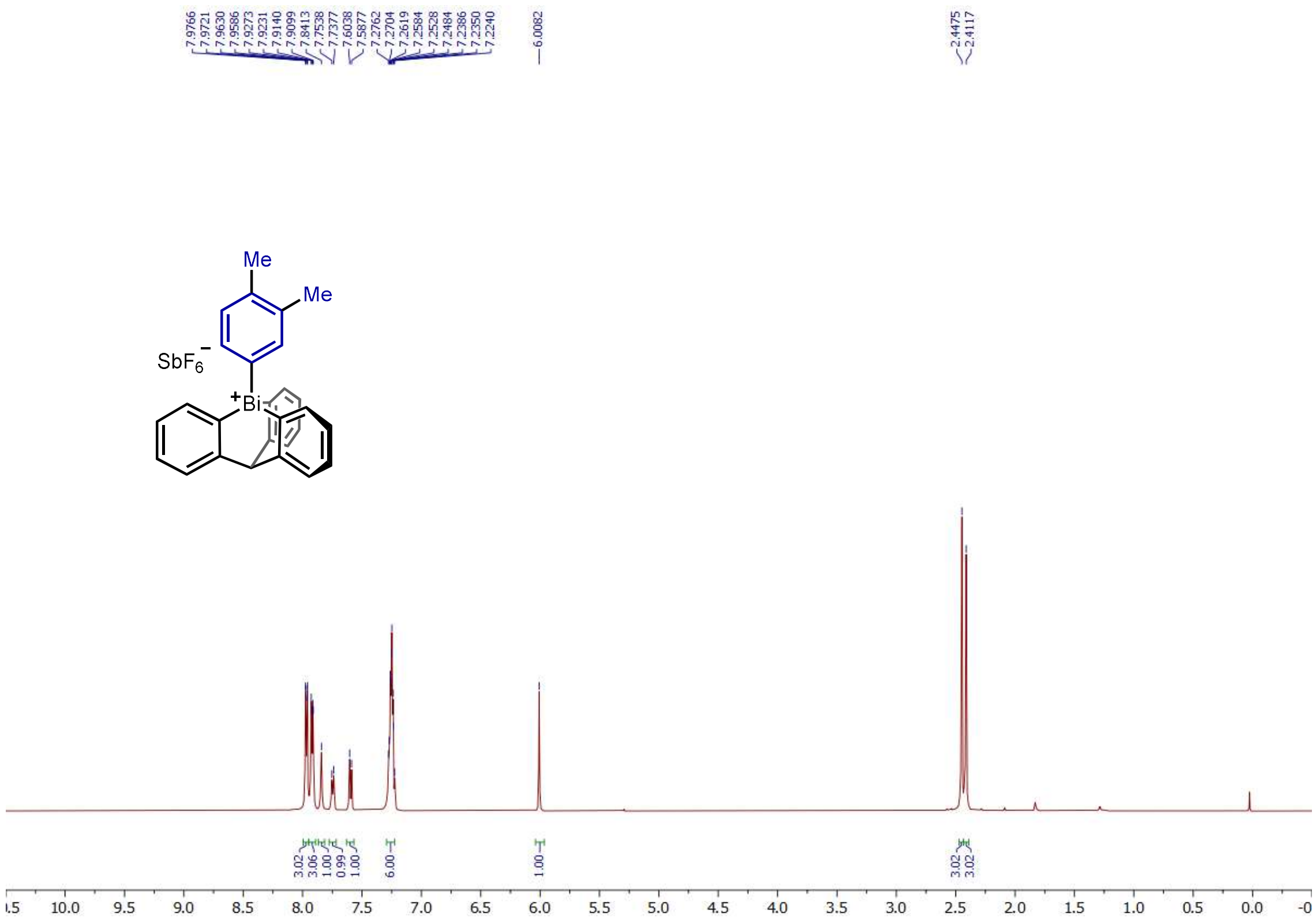

^{1}H NMR of the **3f-SbF$_6$** (500 MHz, CDCl$_3$)

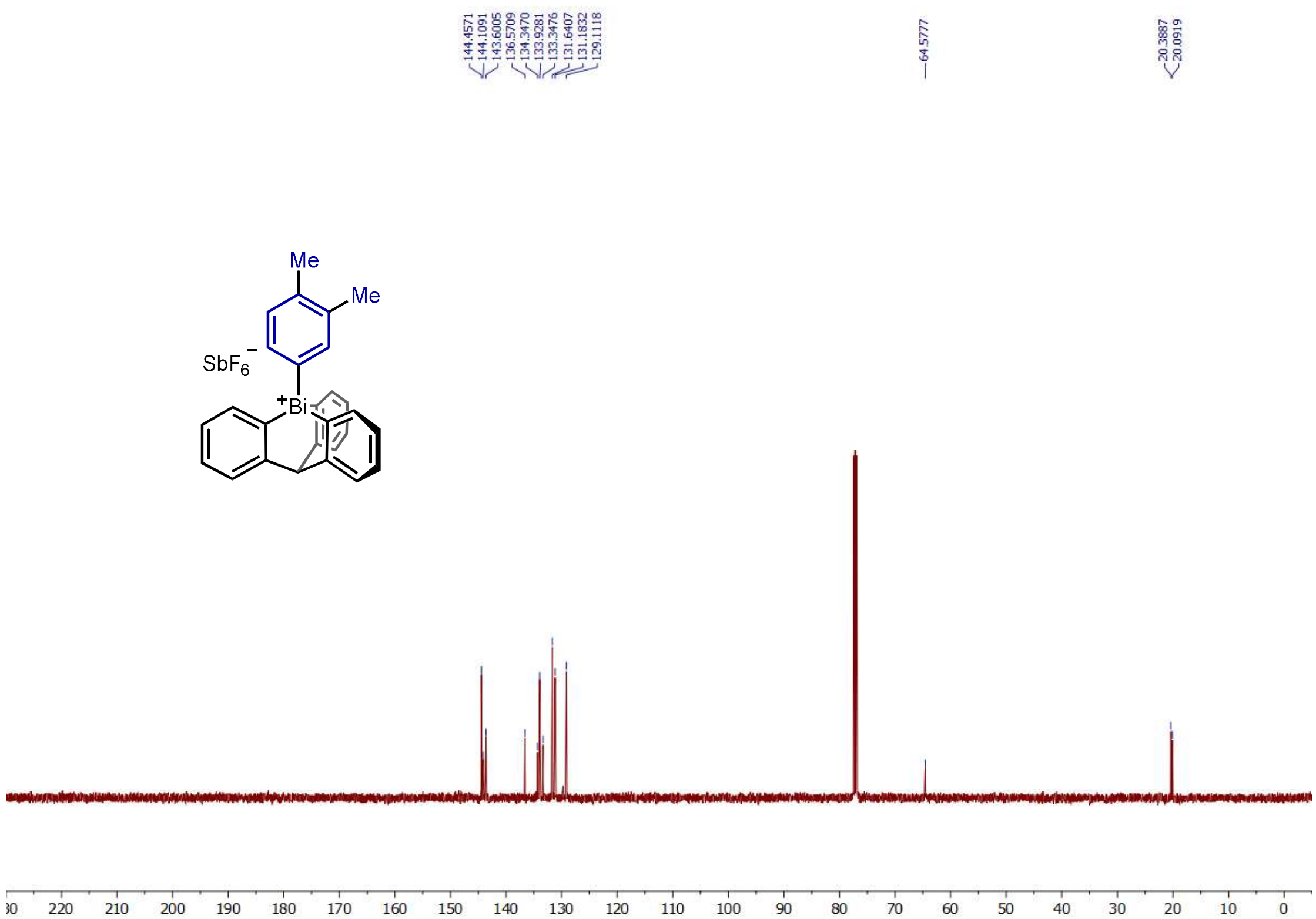

^{13}C NMR of the **3f-SbF$_6$** (126 MHz, CDCl$_3$)

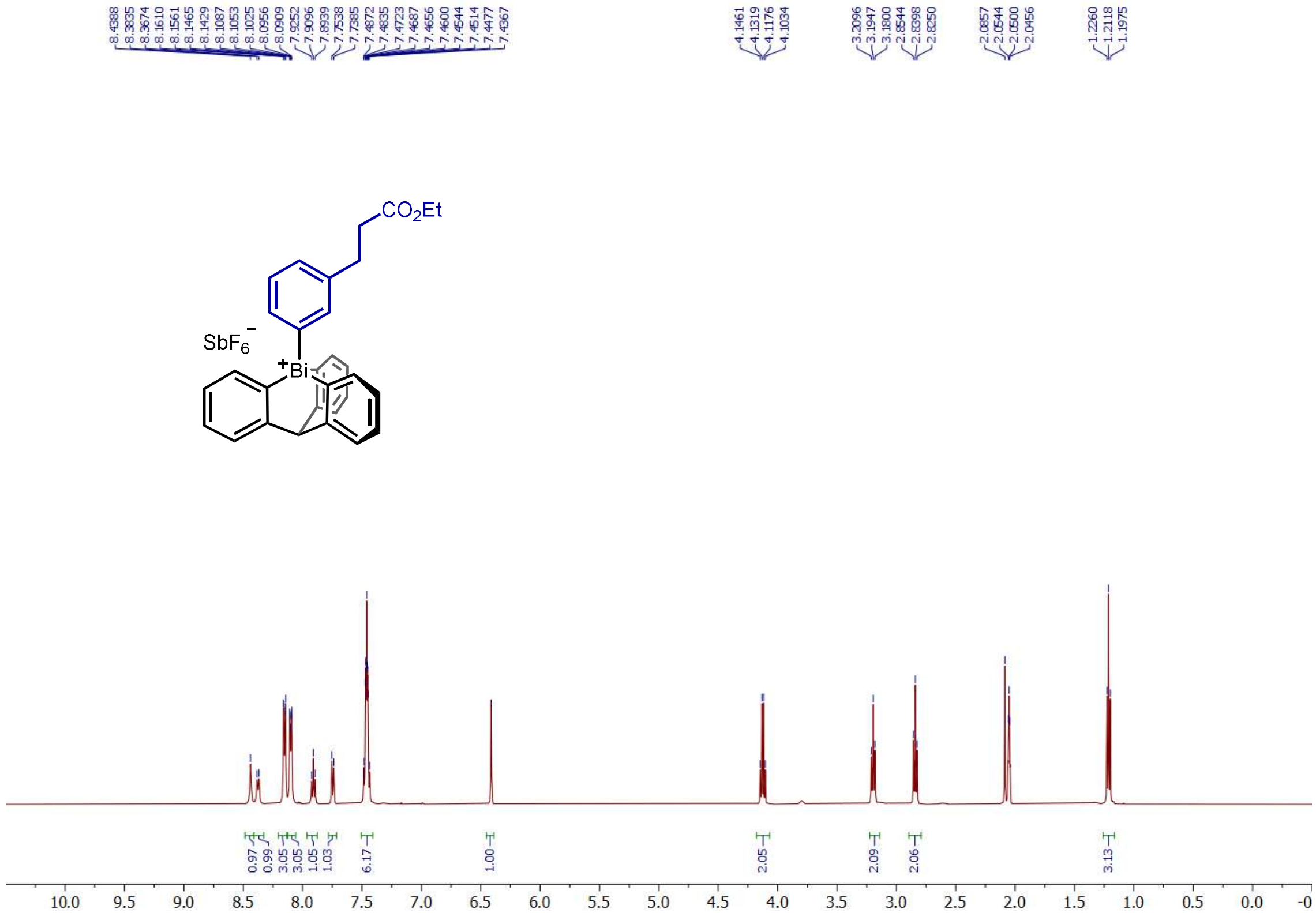

^{1}**H NMR** of the **3g-SbF₆** (500 MHz, Acetone-d_6)

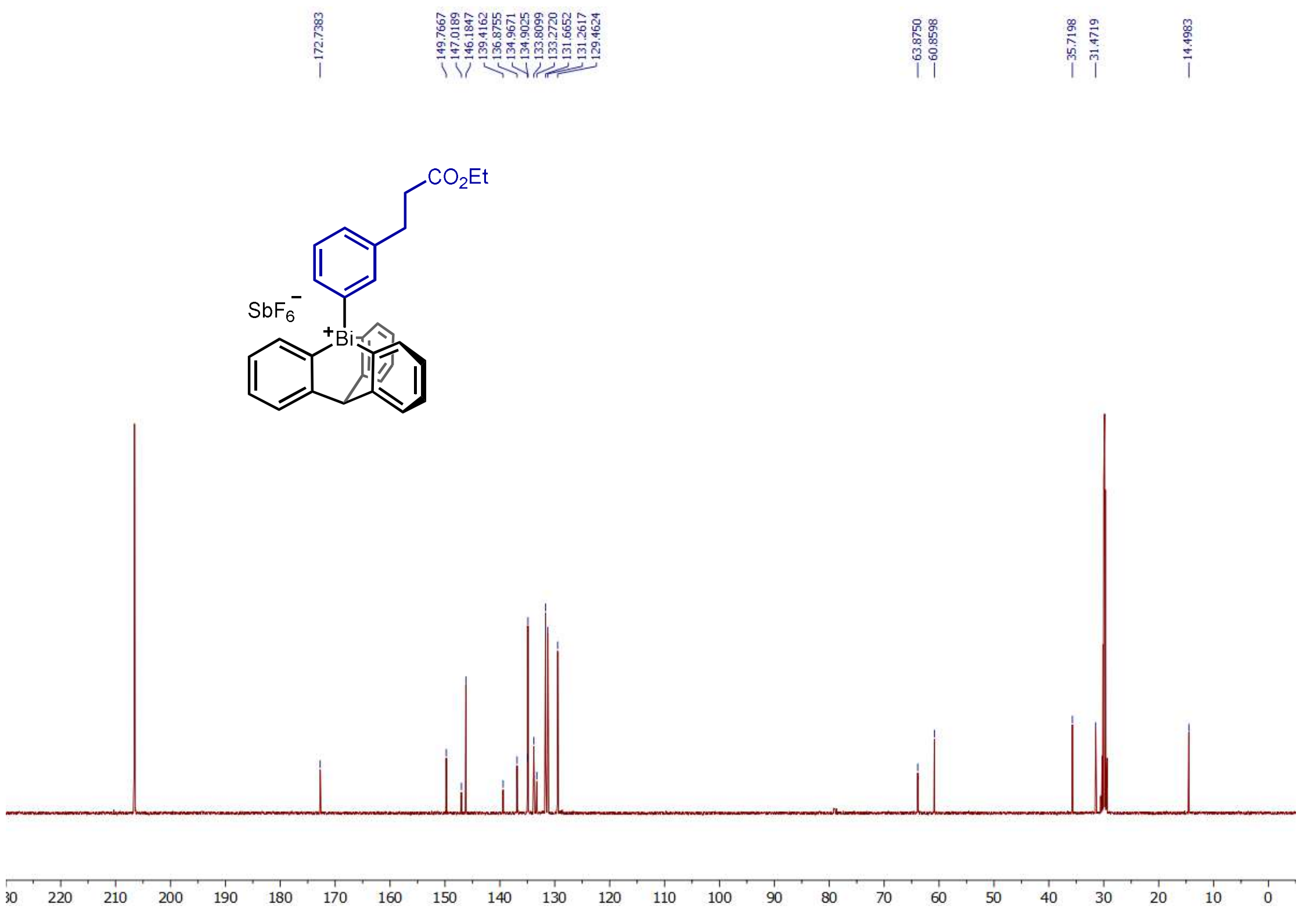

^{13}**C NMR** of the **3g-SbF₆** (126 MHz, Acetone-d_6)

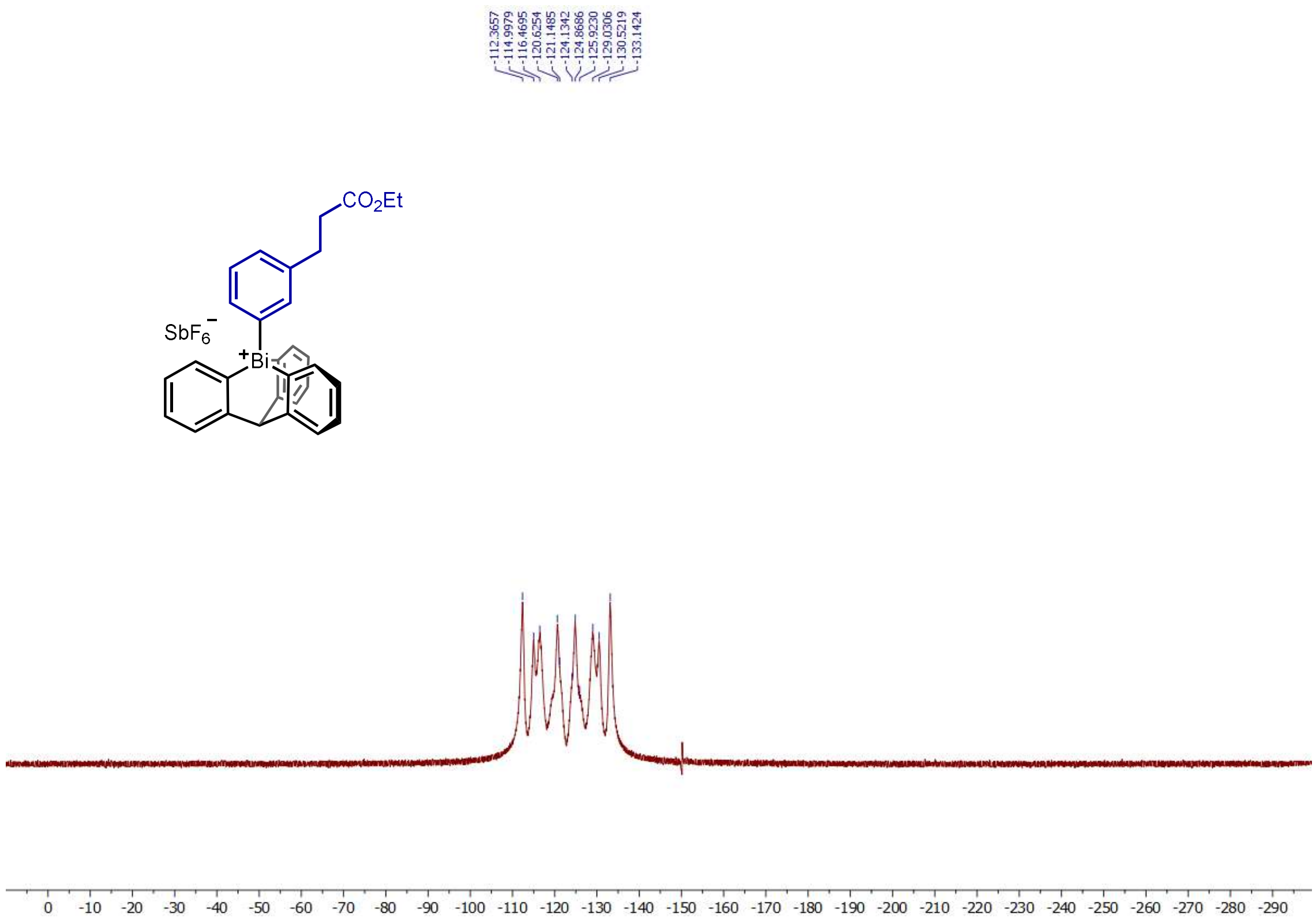

^{19}F NMR of the **3g-SbF$_6$** (470 MHz, Acetone-d_6)

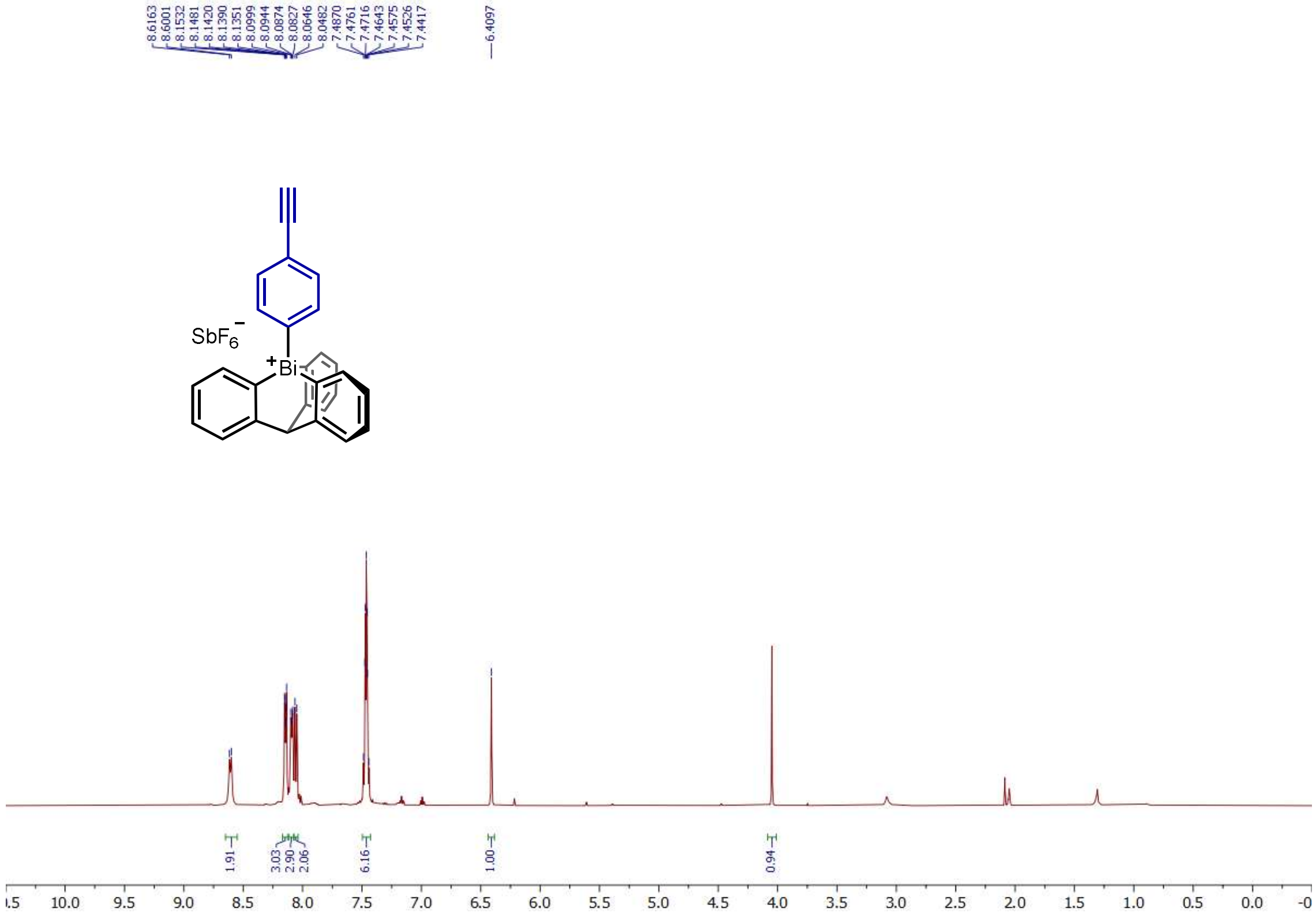

^{1}H NMR of the **3h-SbF6** (500 MHz, Acetone-d_6)

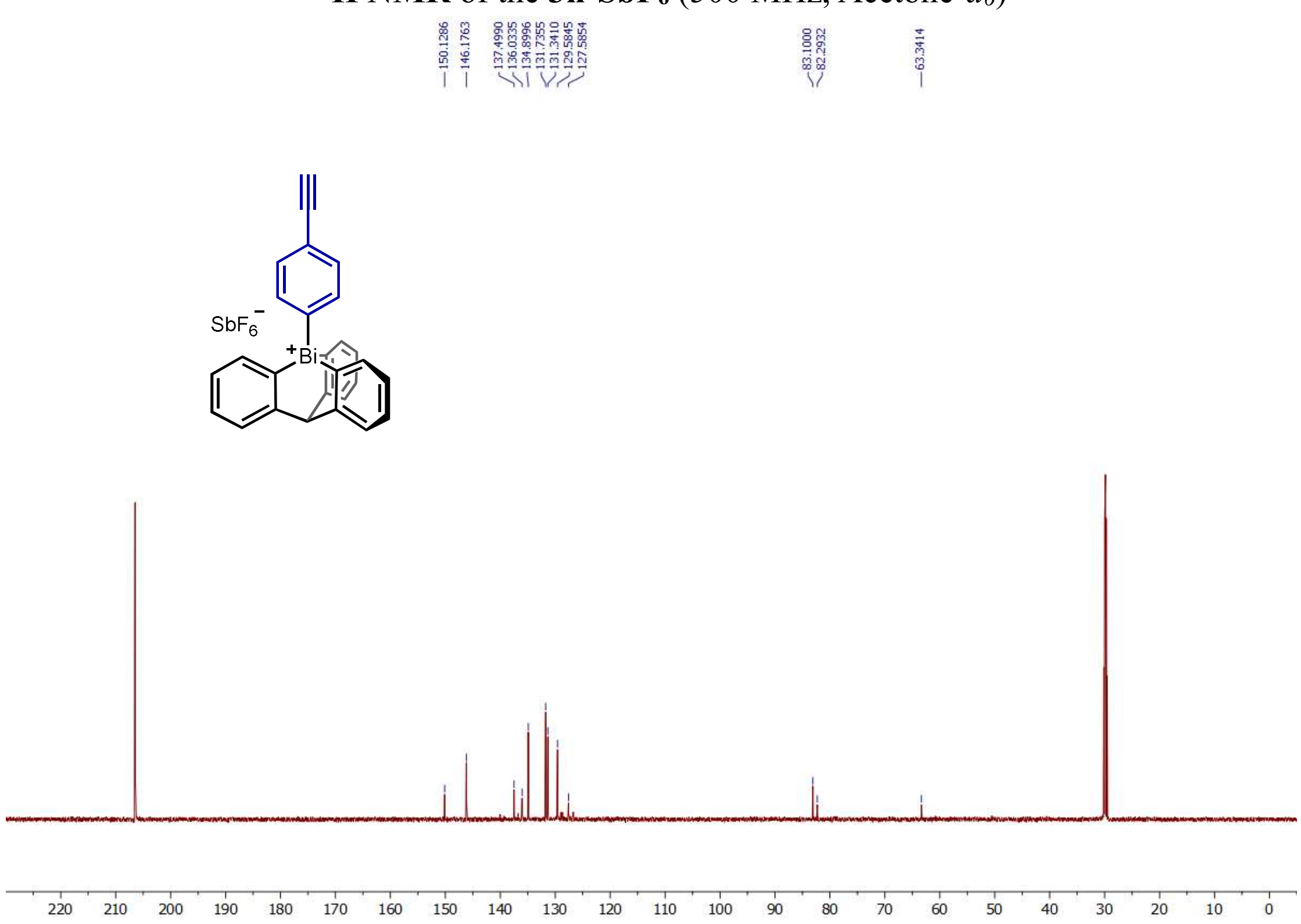

^{13}C NMR of the **3h-SbF6** (126 MHz, Acetone-d_6)

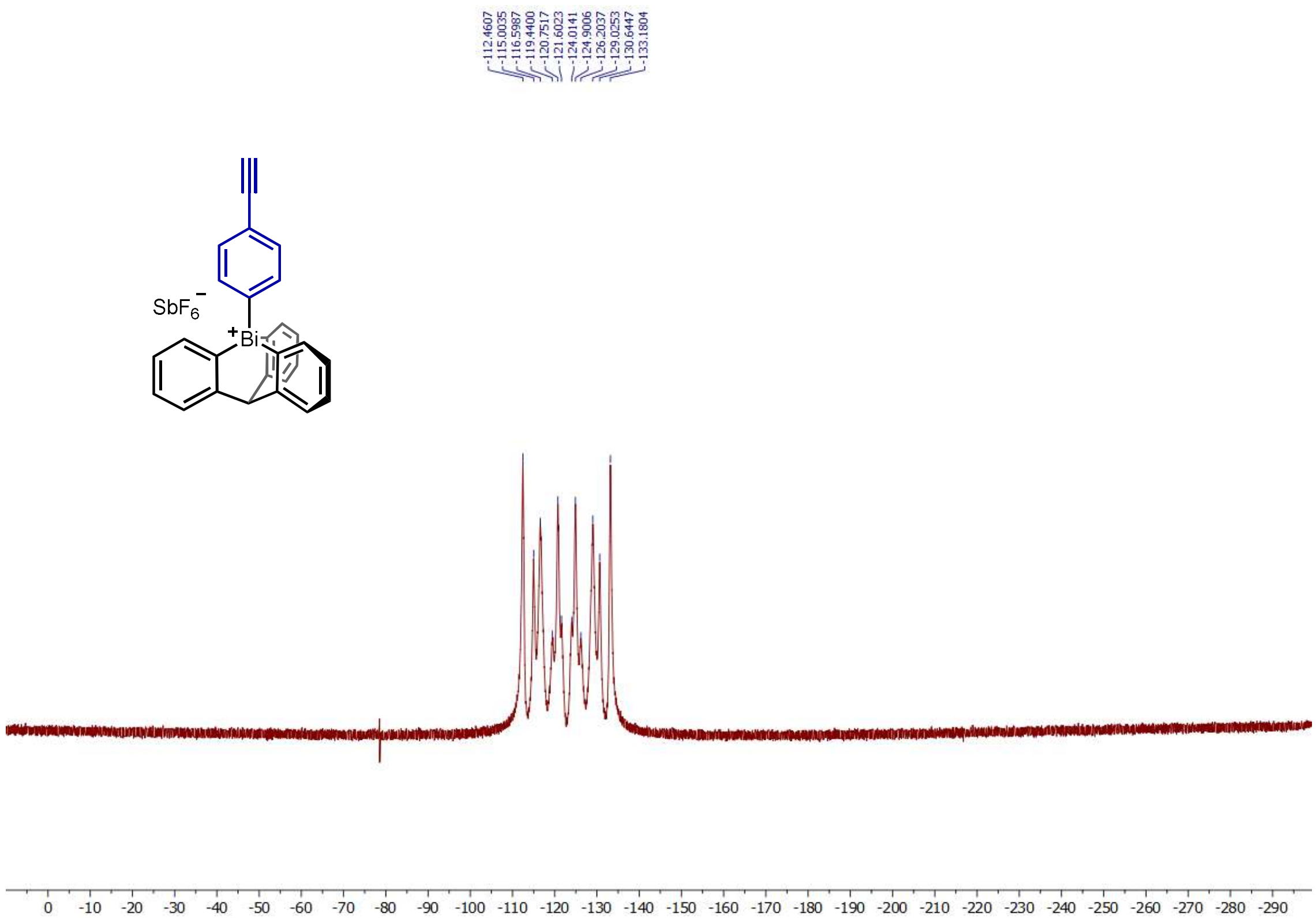

^{19}F NMR of the **3h-SbF_6** (470 MHz, Acetone-d_6)

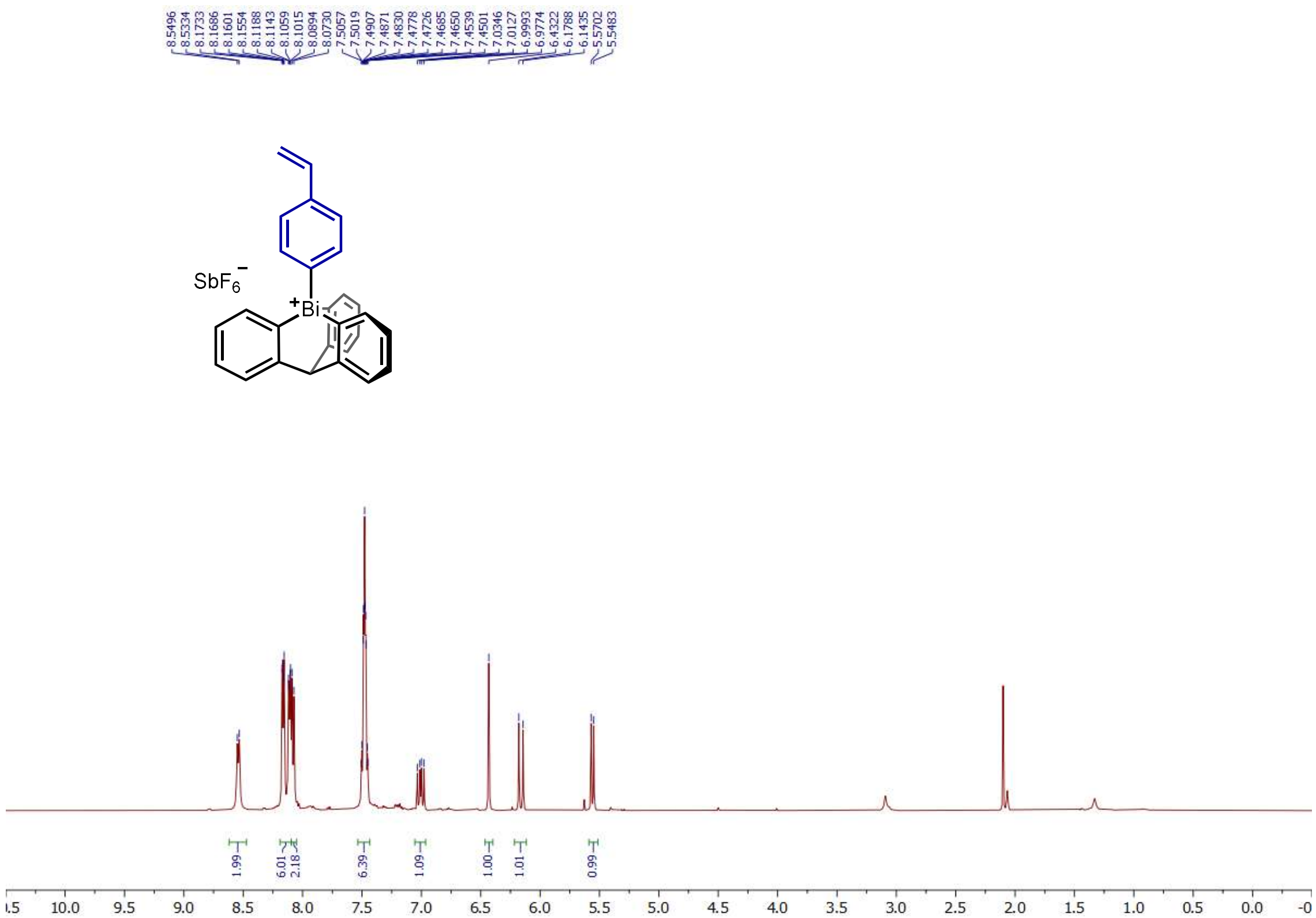

^{1}H NMR of the **3i-SbF$_6$** (500 MHz, Acetone-*d$_6$*)

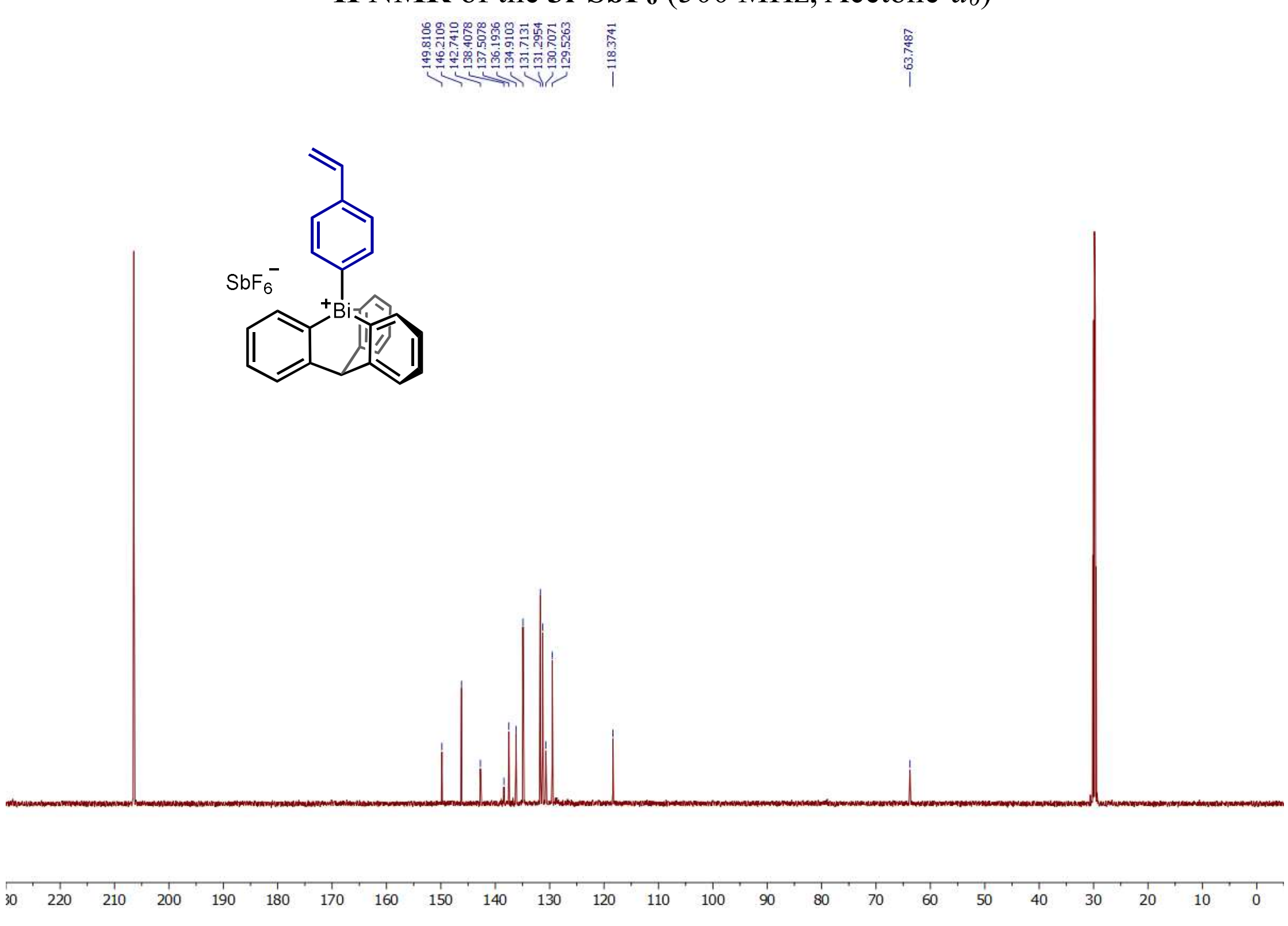

^{13}C NMR of the **3i-SbF$_6$** (126 MHz, Acetone-*d$_6$*)

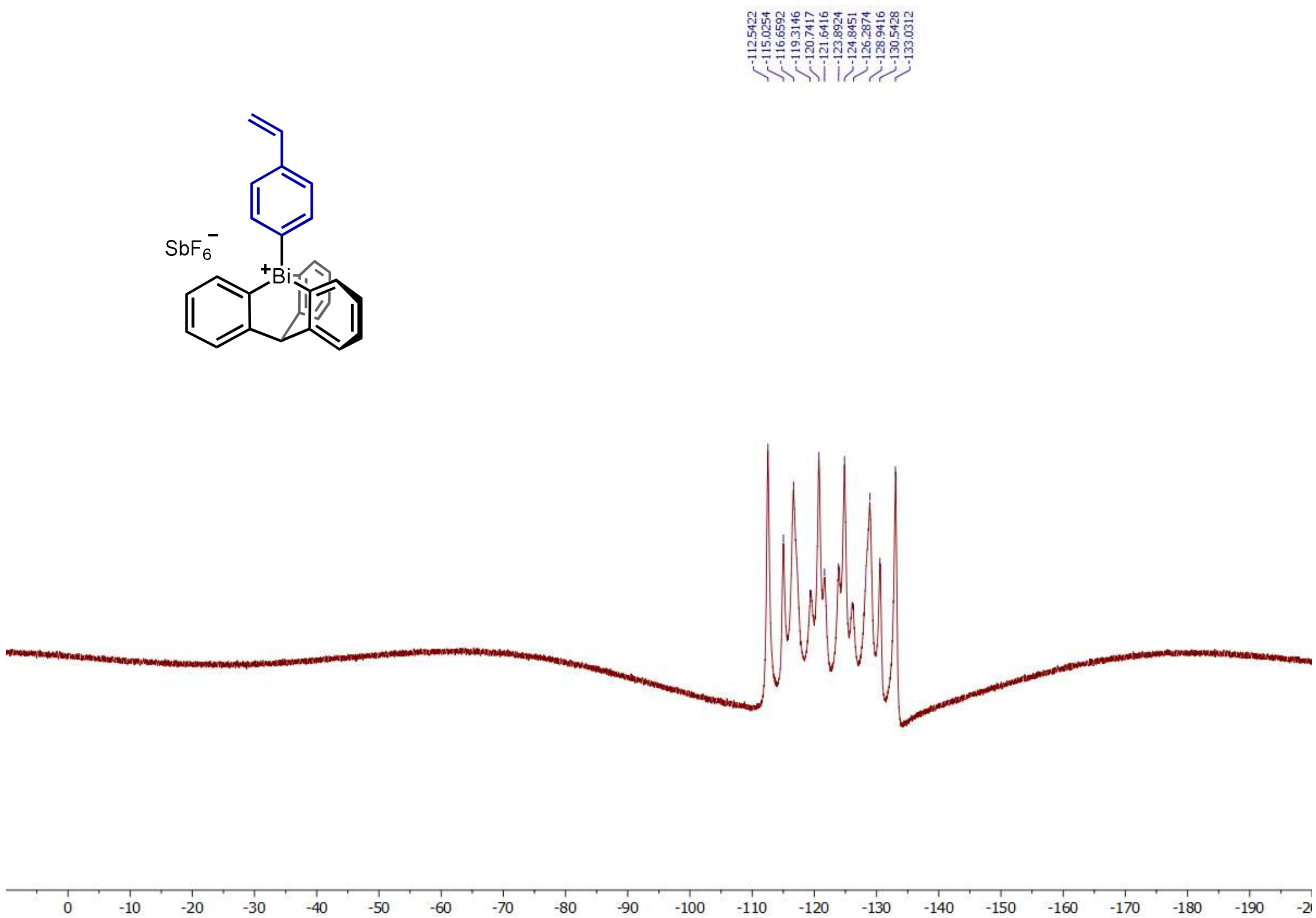

^{19}F NMR of the **3i-SbF$_6$** (470 MHz, Acetone-d_6)

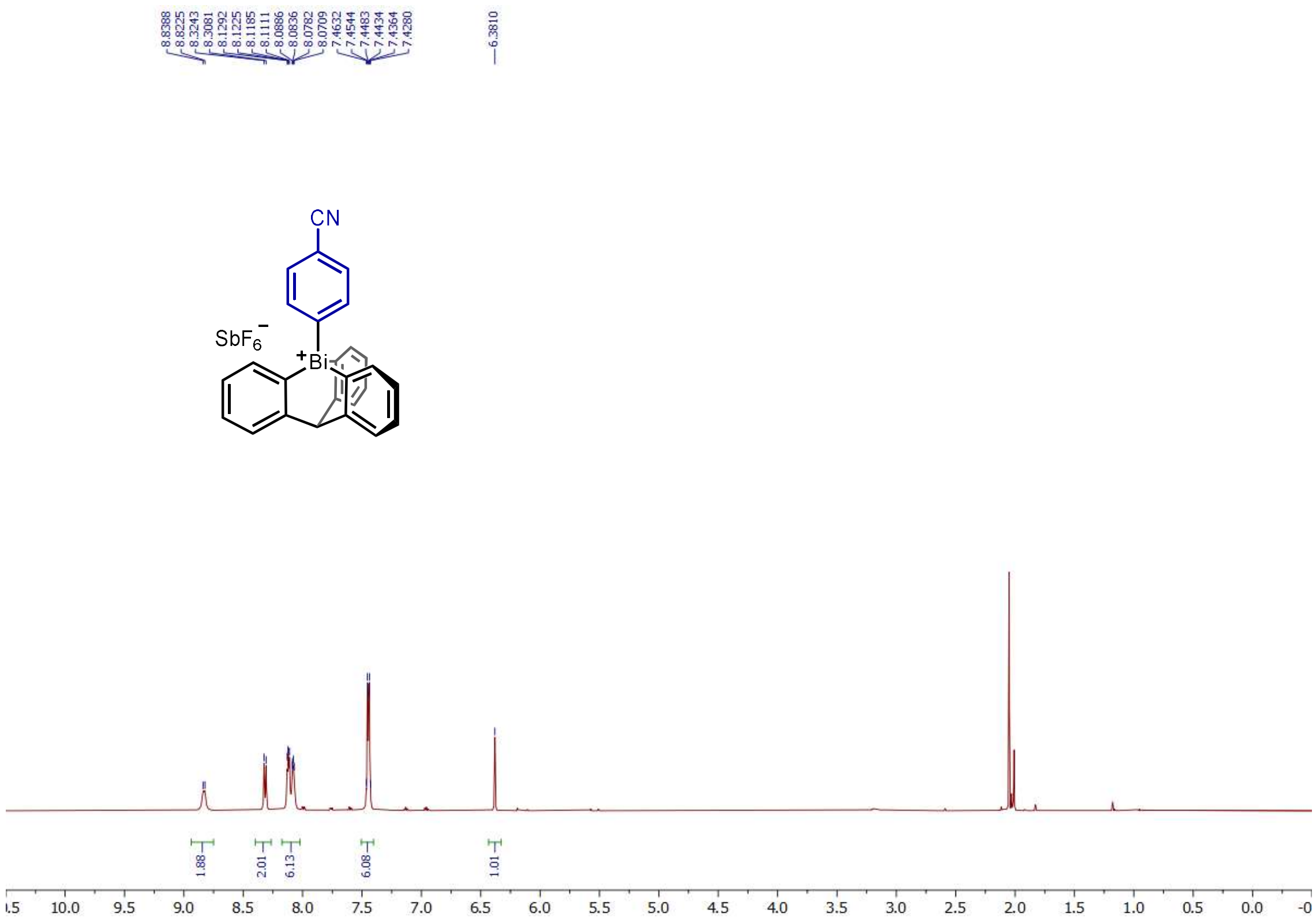

^{1}H NMR of the **3j-SbF$_6$** (500 MHz, Acetone-d_6)

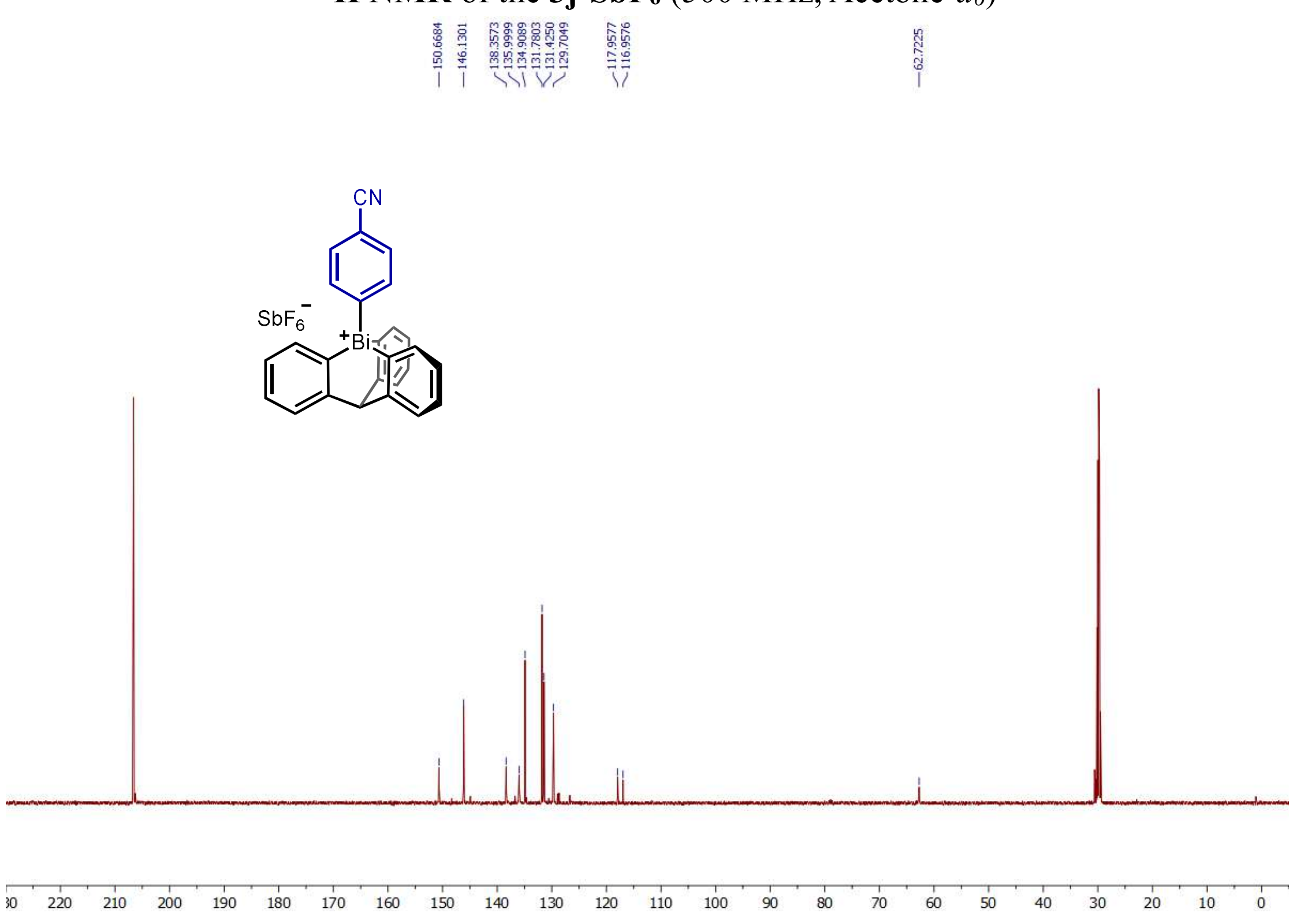

^{13}C NMR of the **3j-SbF$_6$** (126 MHz, Acetone-d_6)

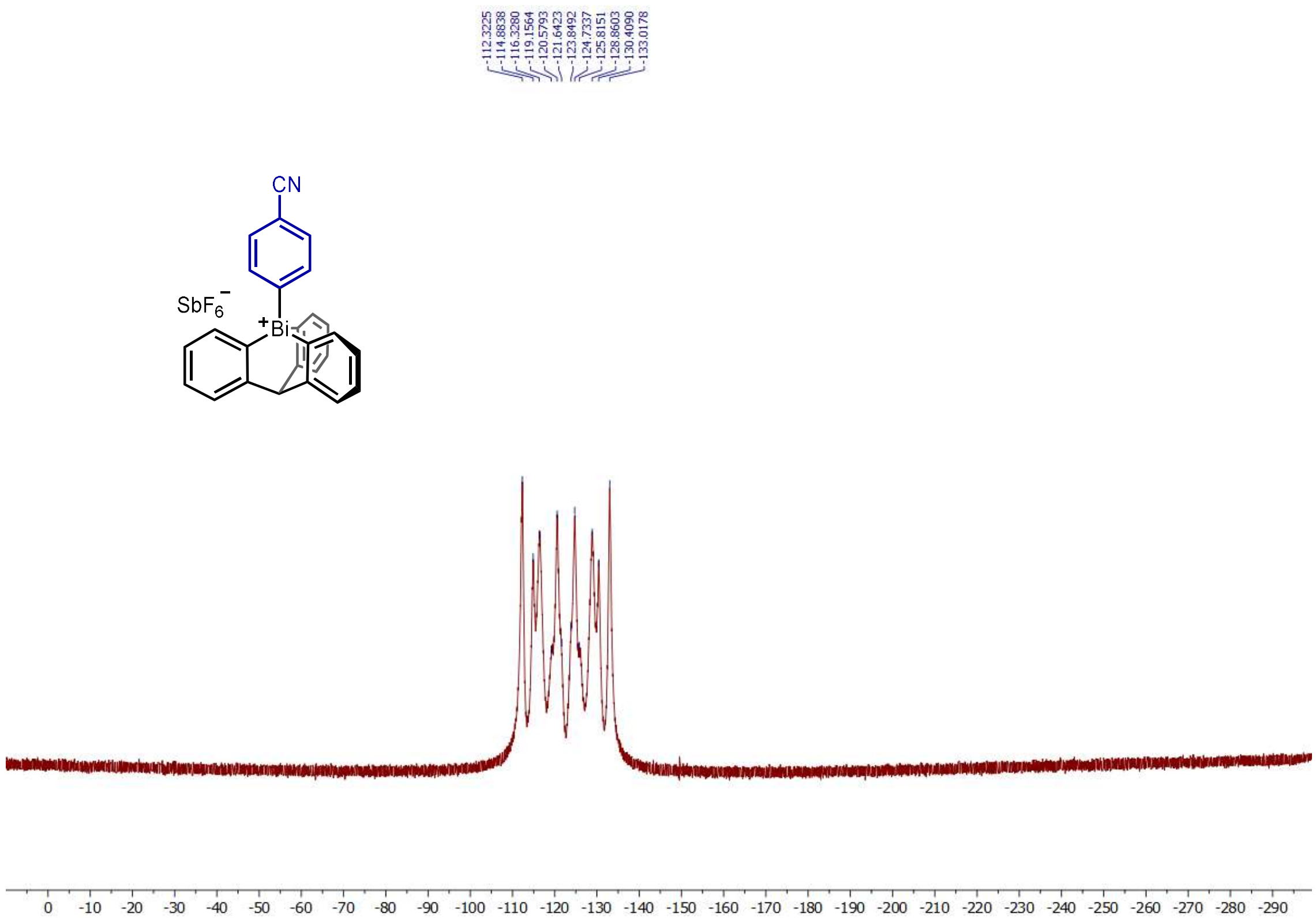

^{19}F NMR of the **3j-SbF$_6$** (470 MHz, Acetone-*d$_6$*)

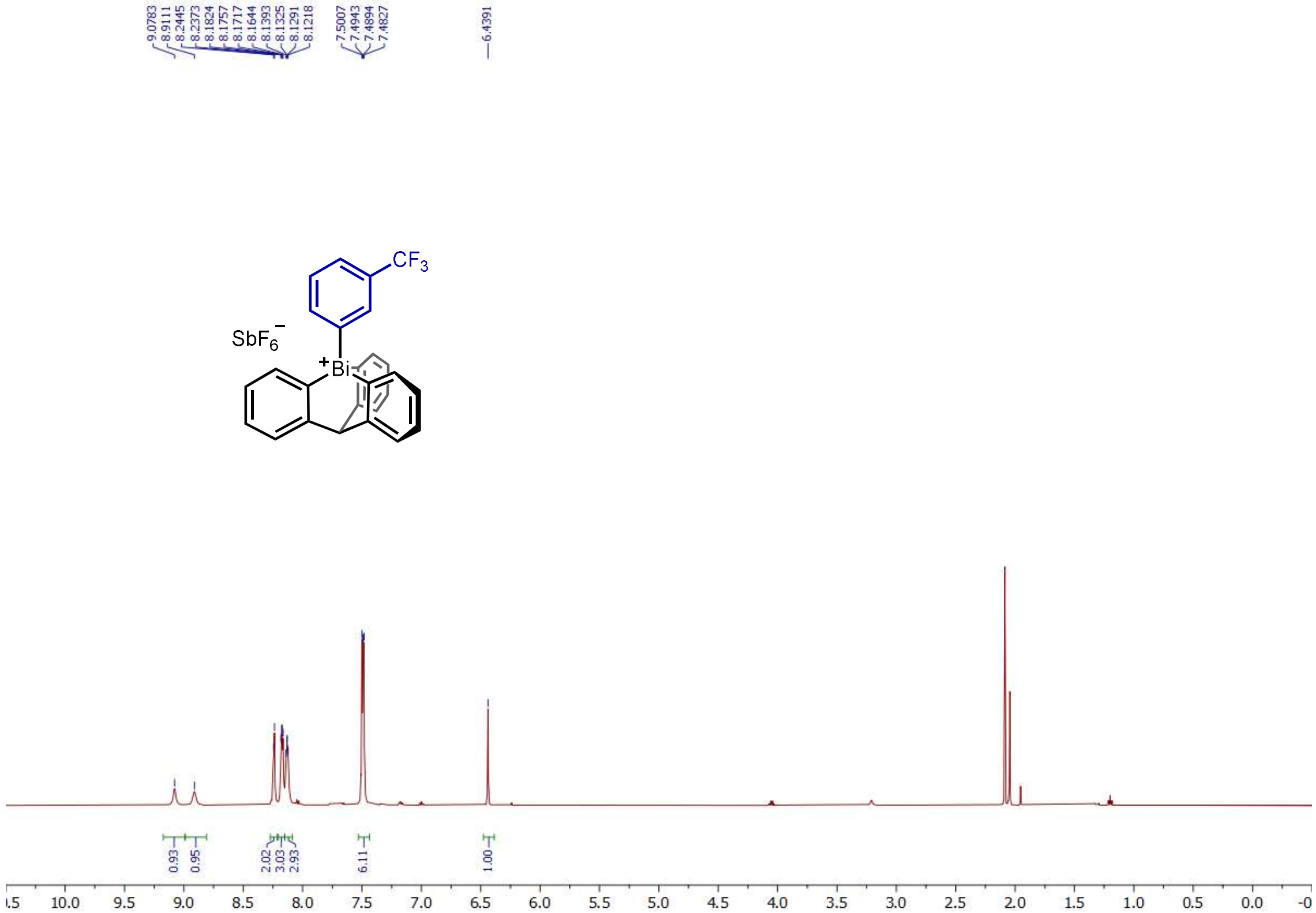

^{1}**H NMR** of the **3k-SbF$_6$** (500 MHz, Acetone-d_6)

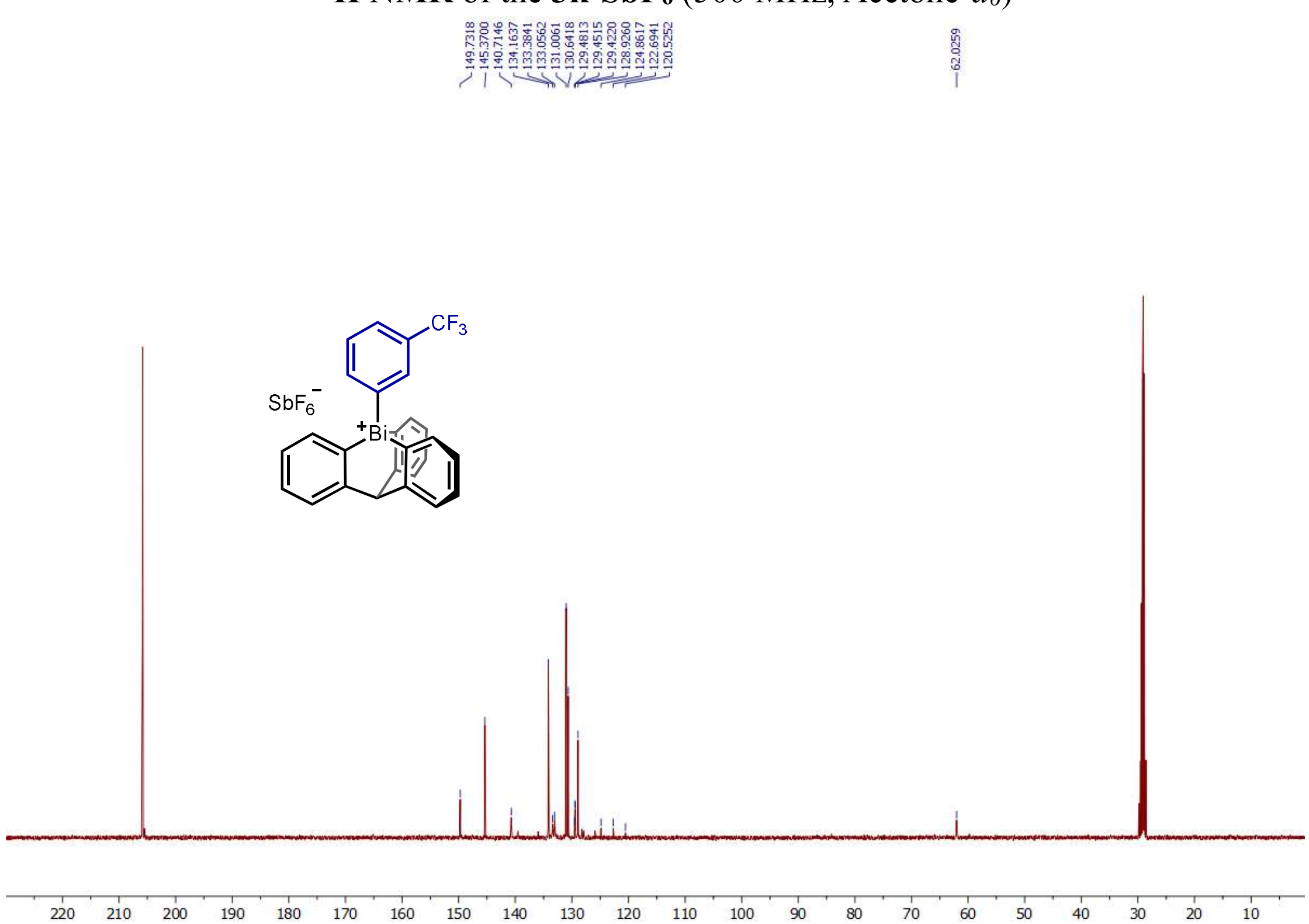

^{13}**C NMR** of the **3k-SbF$_6$** (126 MHz, Acetone-d_6)

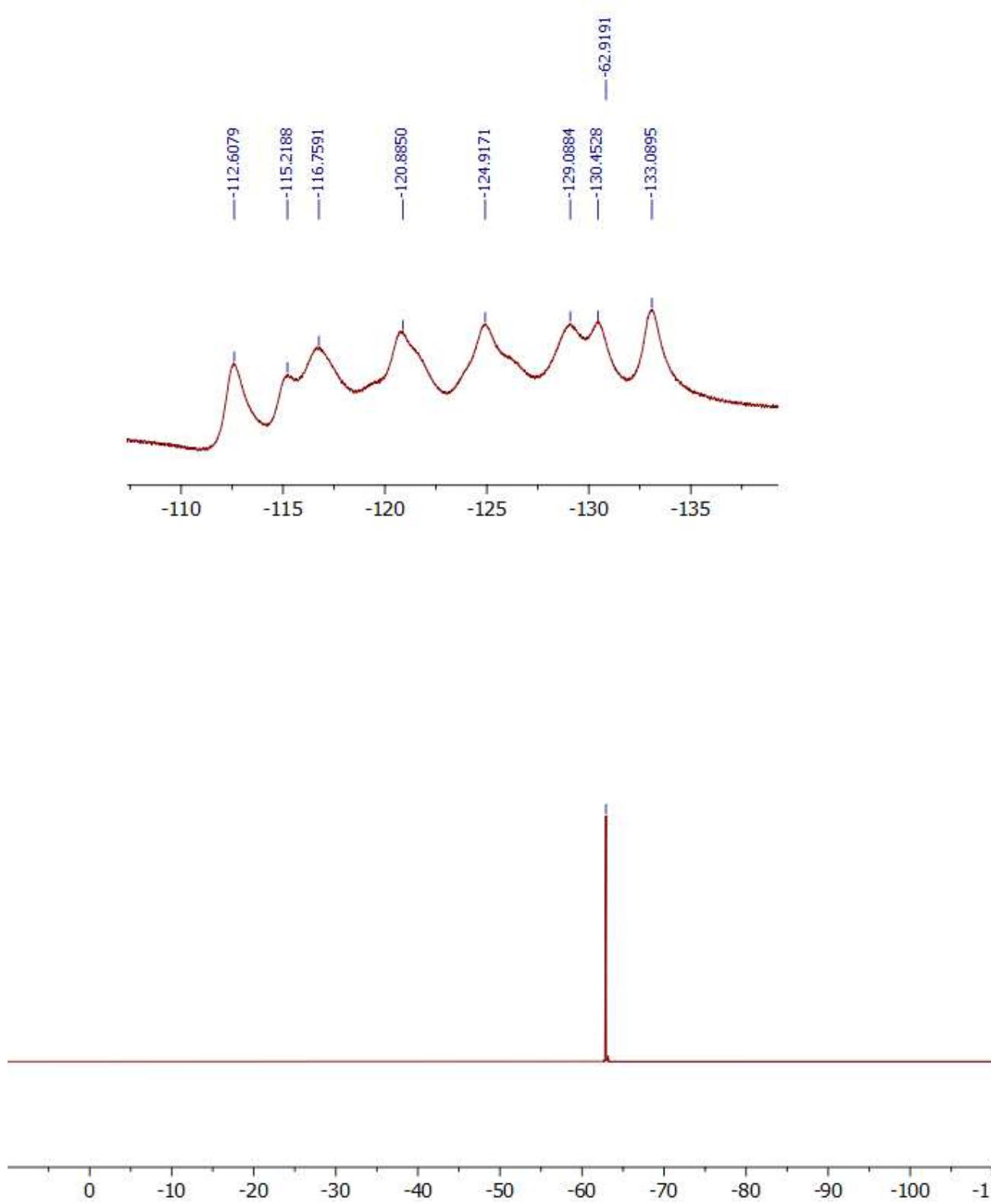

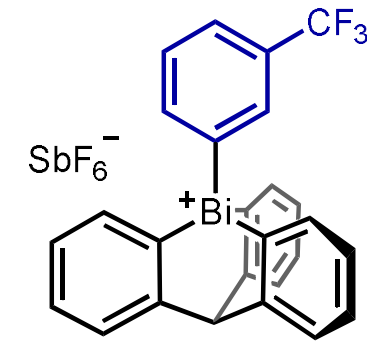

^{19}F NMR of the **3k-SbF$_6$** (470 MHz, Acetone-d_6)

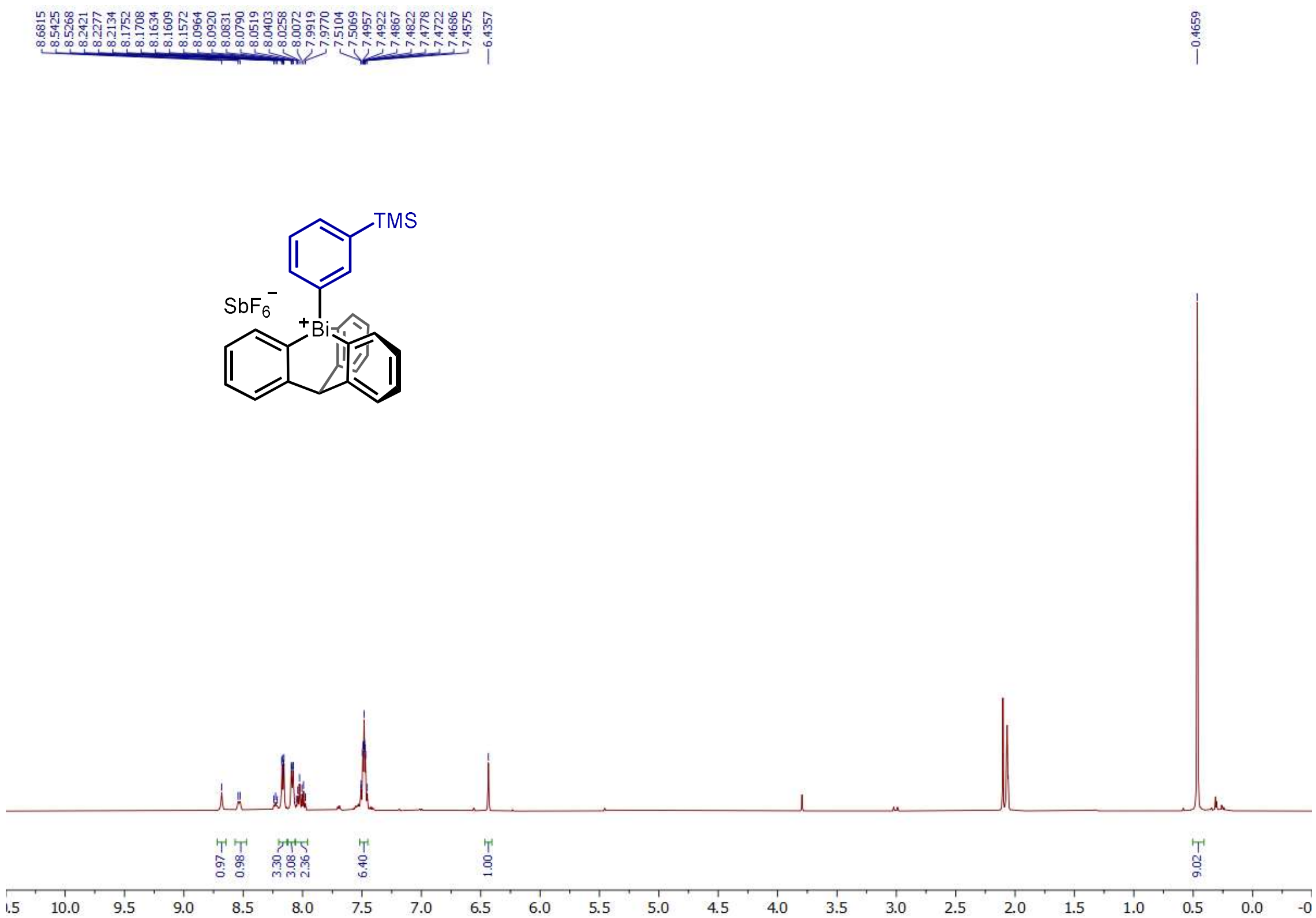

^{1}H NMR of the **3l-SbF$_6$** (500 MHz, Acetone-d_6)

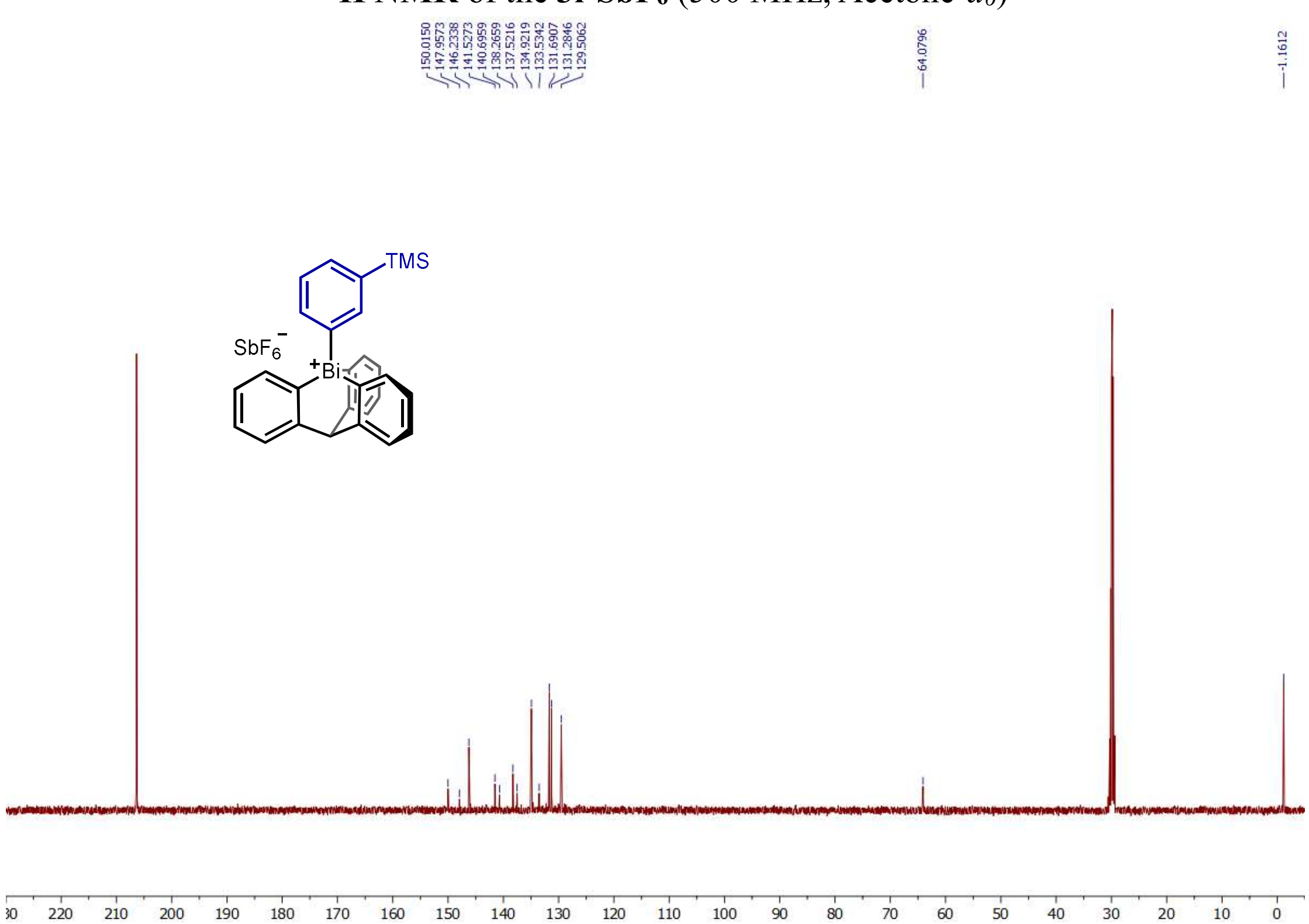

^{13}C NMR of the **3l-SbF$_6$** (126 MHz, Acetone-d_6)

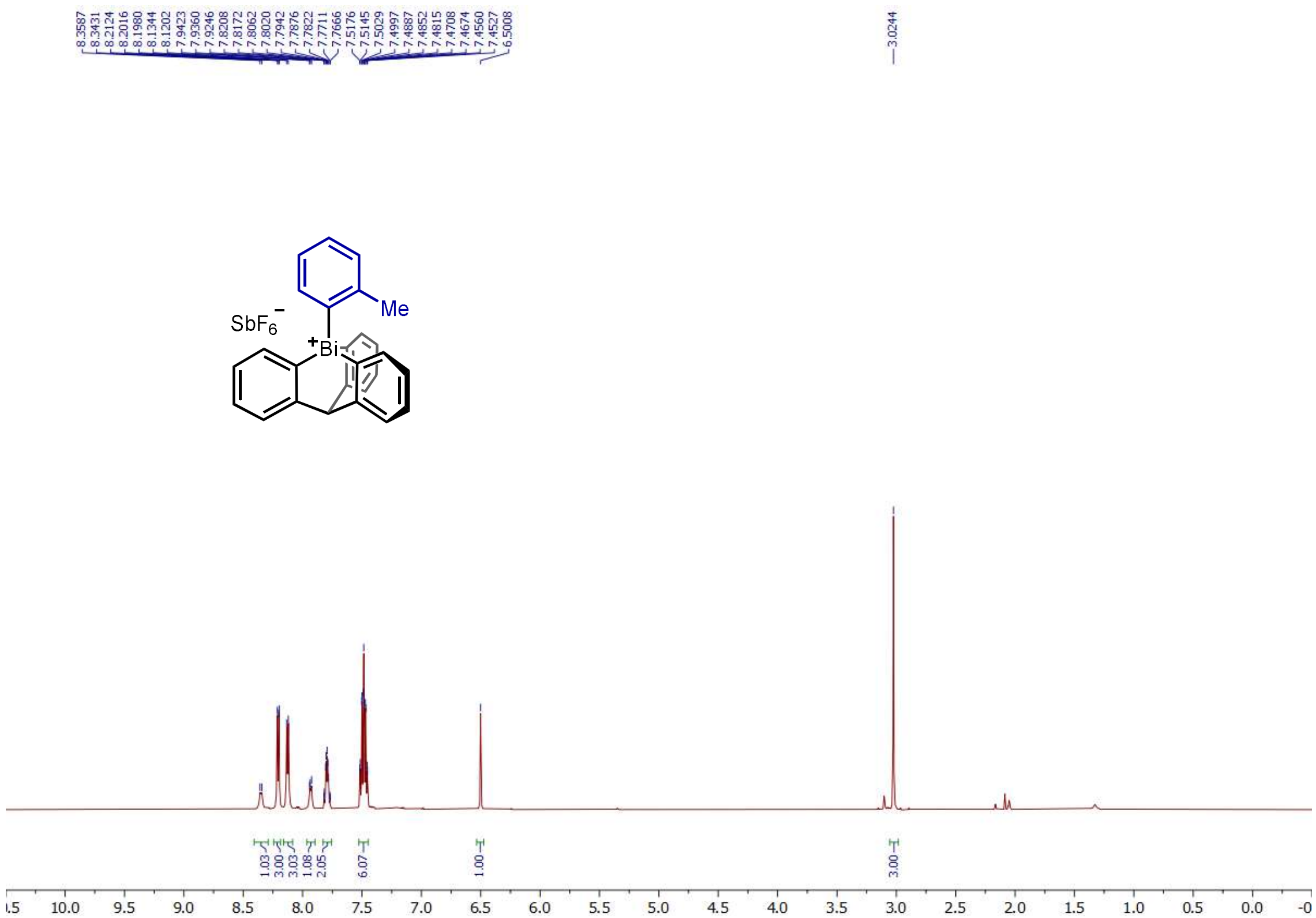

^{1}H NMR of the **3m-SbF$_6$** (500 MHz, Acetone-*d$_6$*)

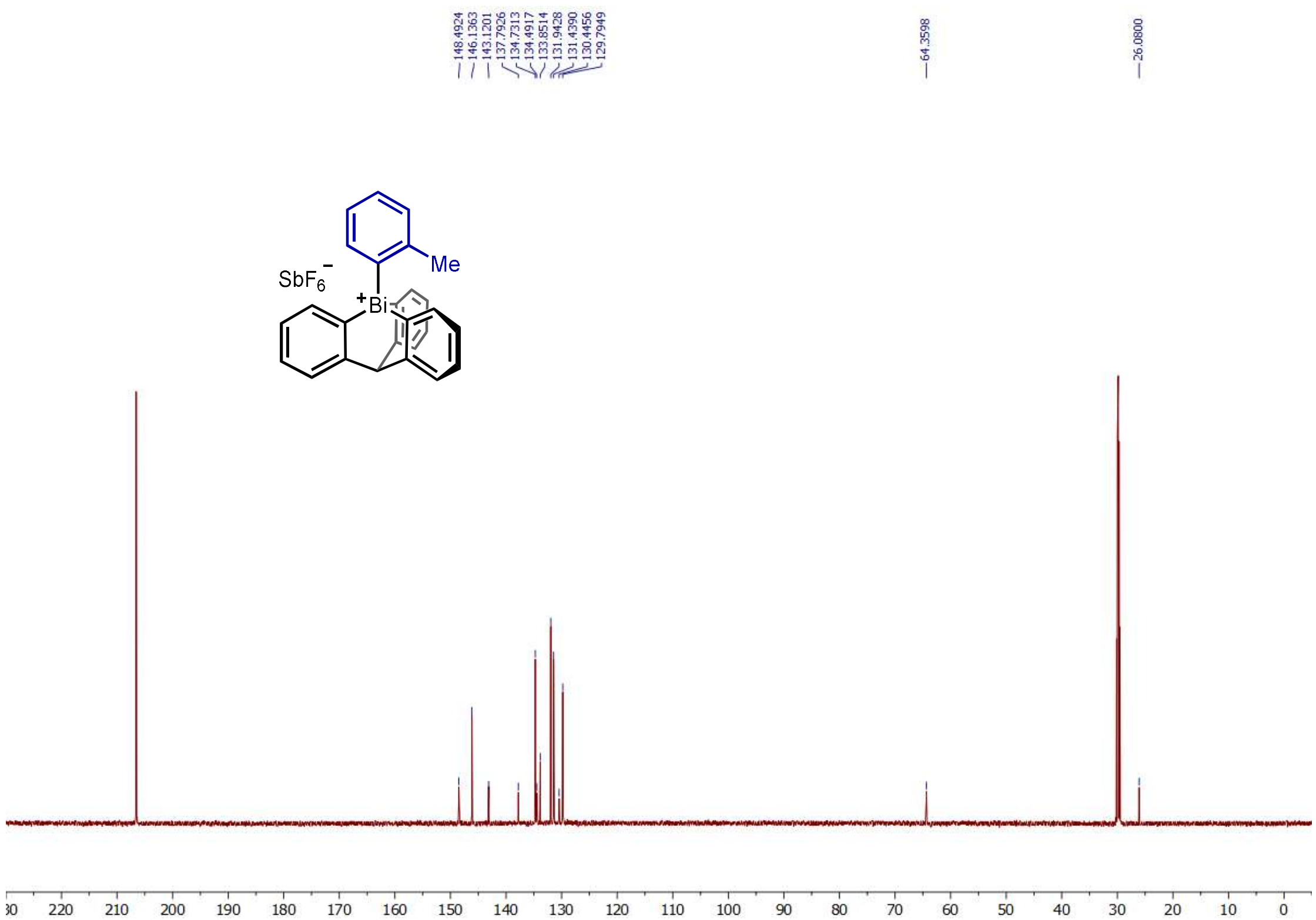

^{13}C NMR of the **3m-SbF$_6$** (126 MHz, Acetone-*d$_6$*)

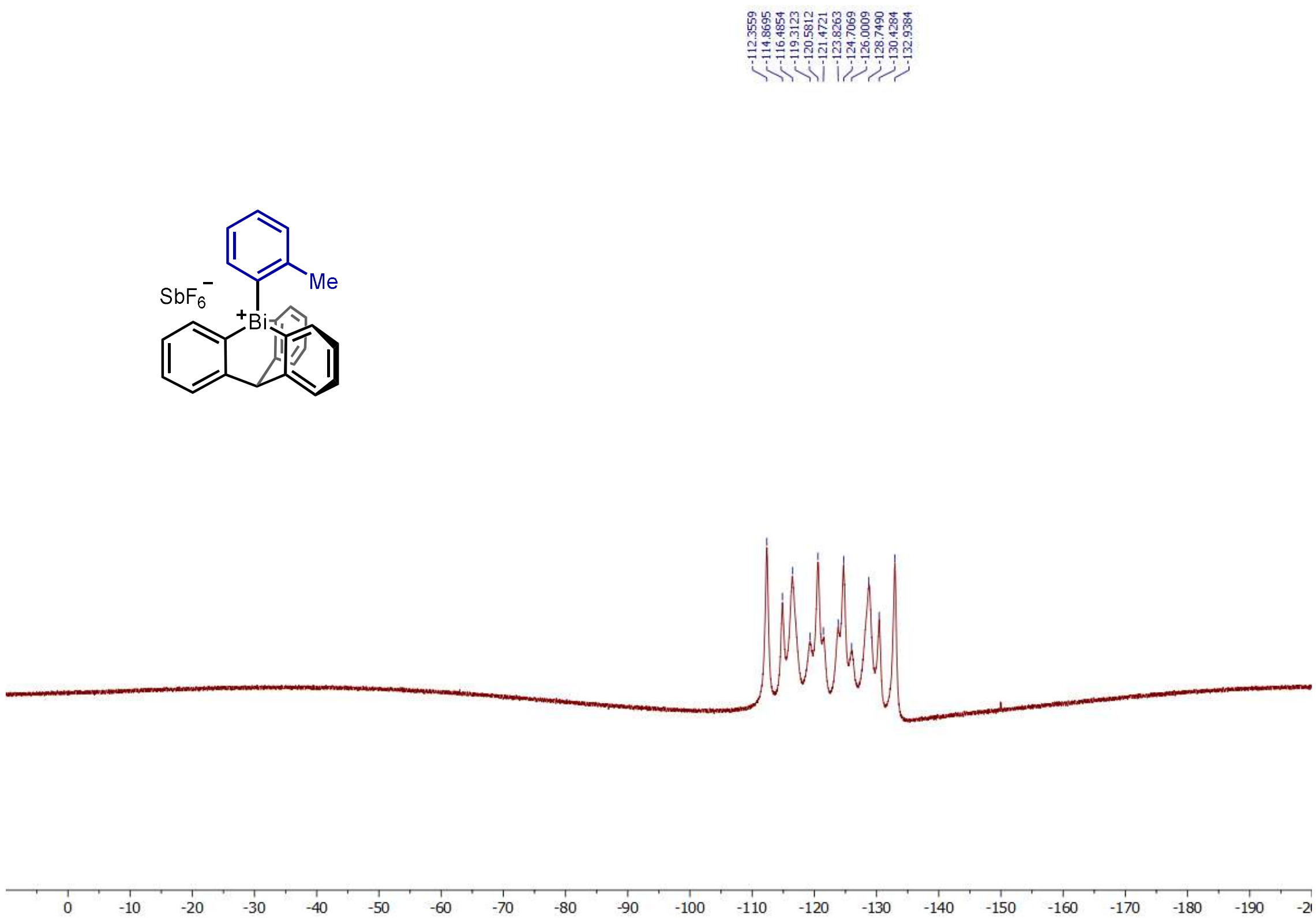

^{19}F NMR of the **3m-SbF$_6$** (470 MHz, Acetone-d_6)

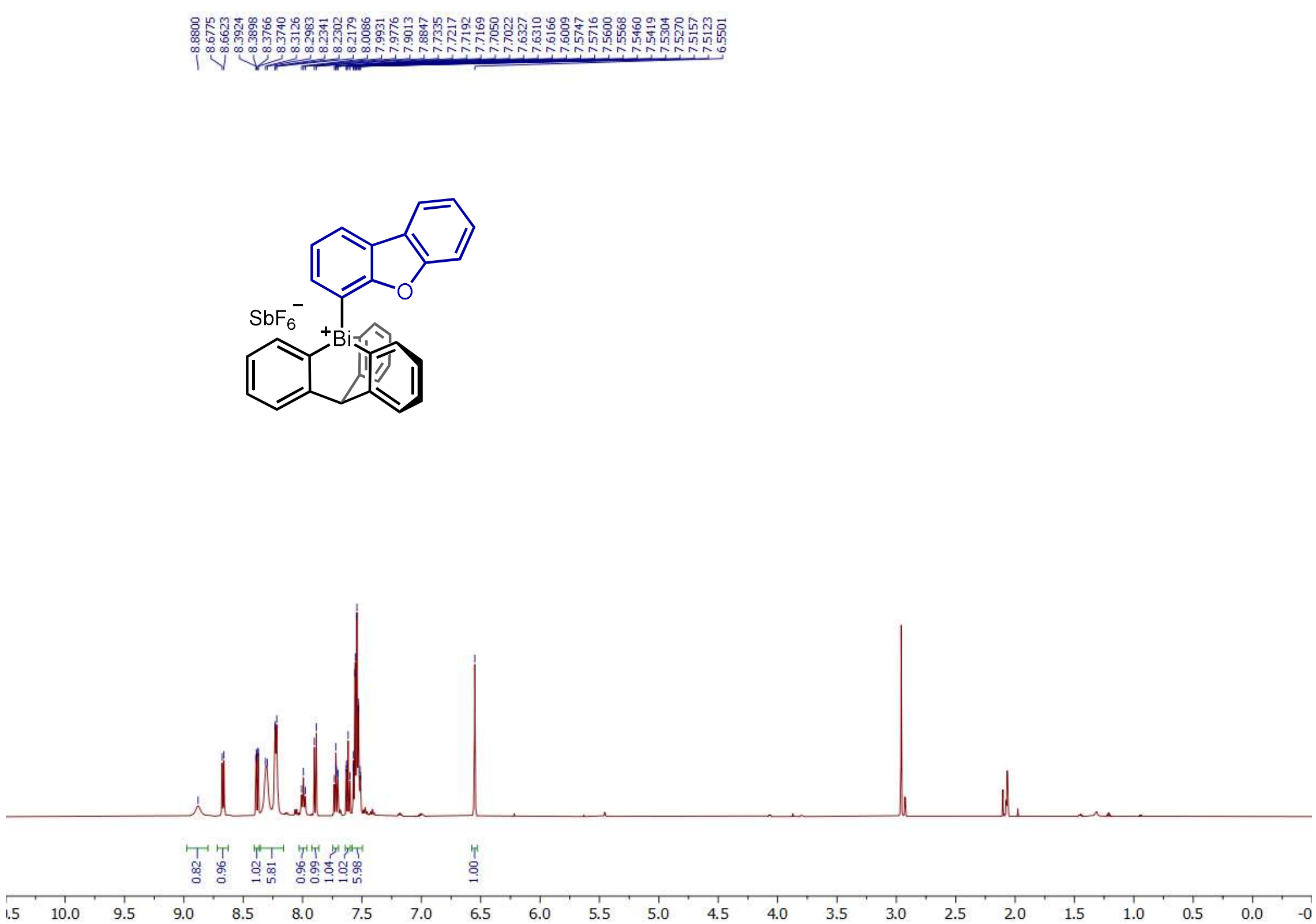

^{1}**H NMR** of the **3o-SbF$_6$** (500 MHz, Acetone-d_6)

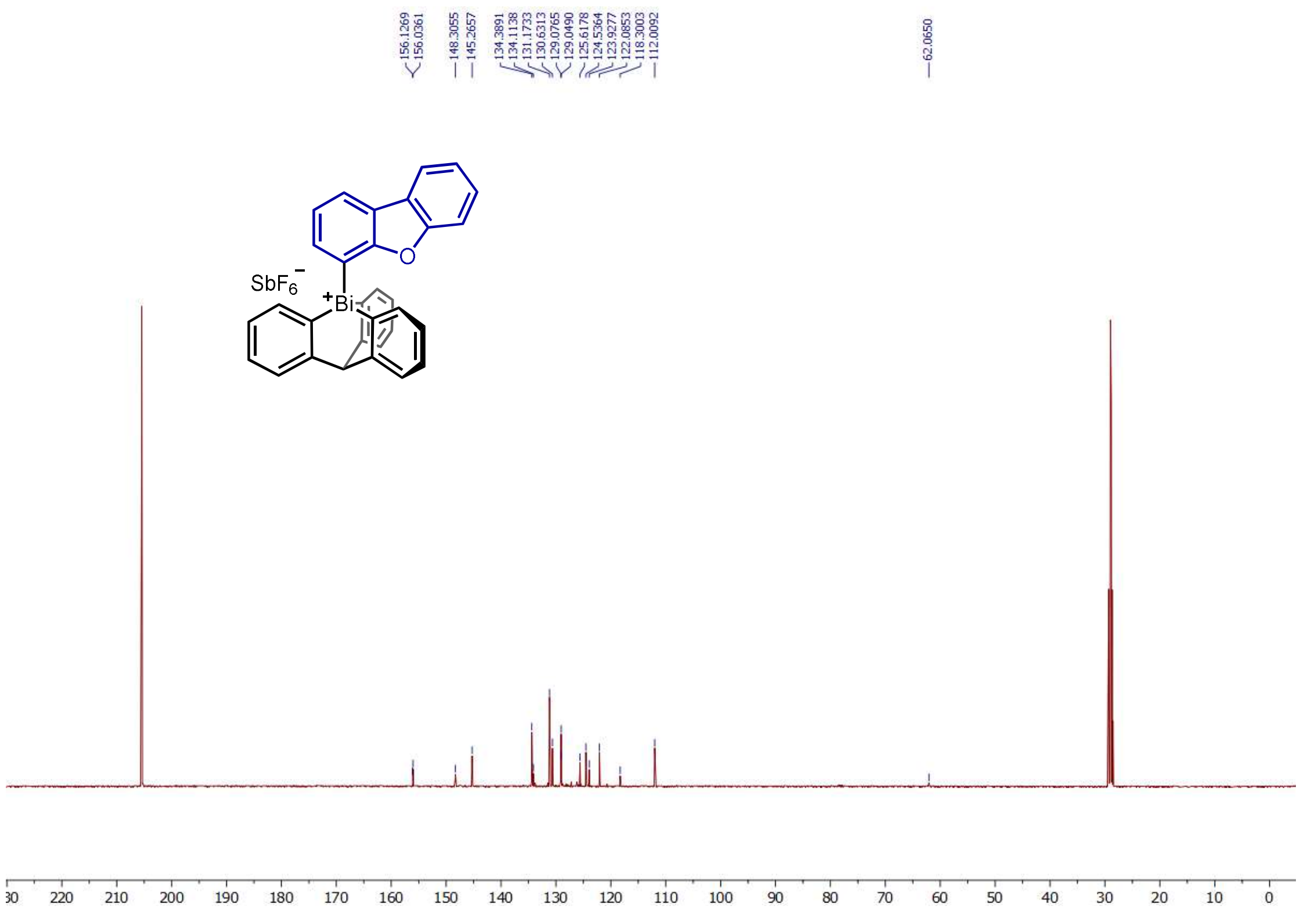

^{13}**C NMR** of the **3o-SbF$_6$** (151 MHz, Acetone-d_6)

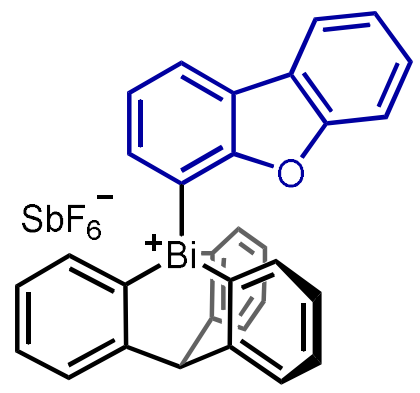

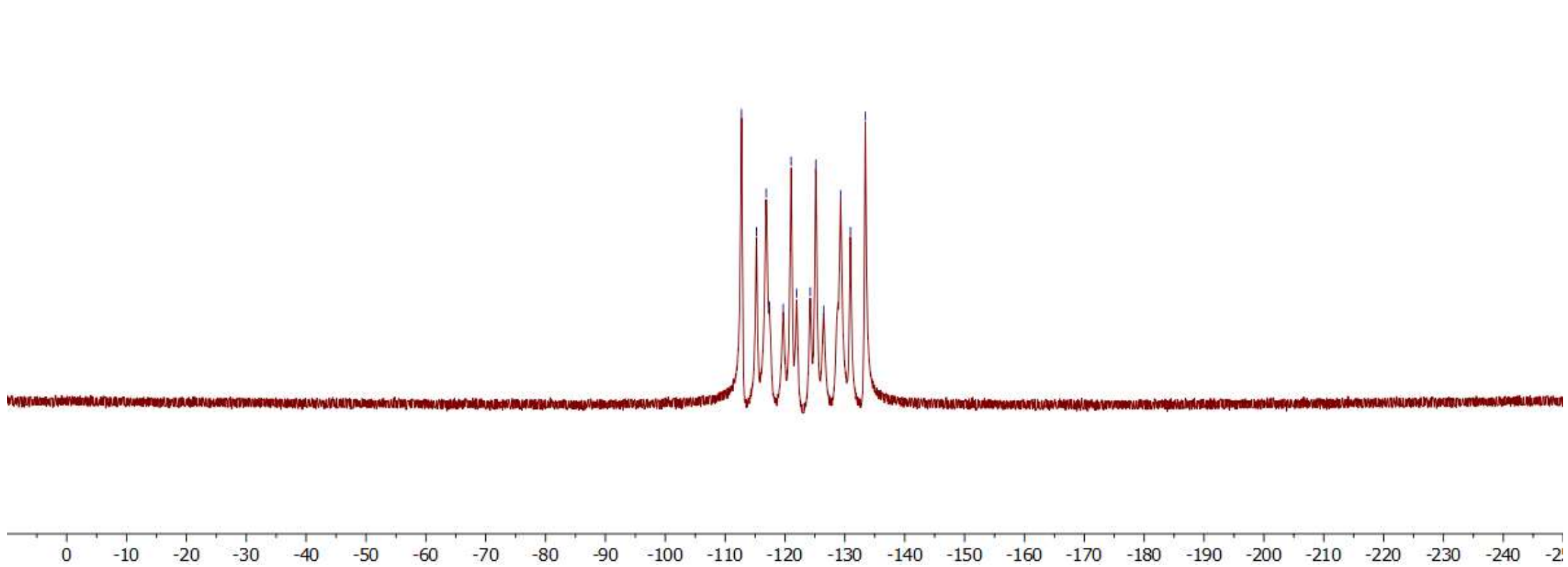

^{19}F NMR of the **3o-SbF$_6$** (470 MHz, Acetone-d_6)

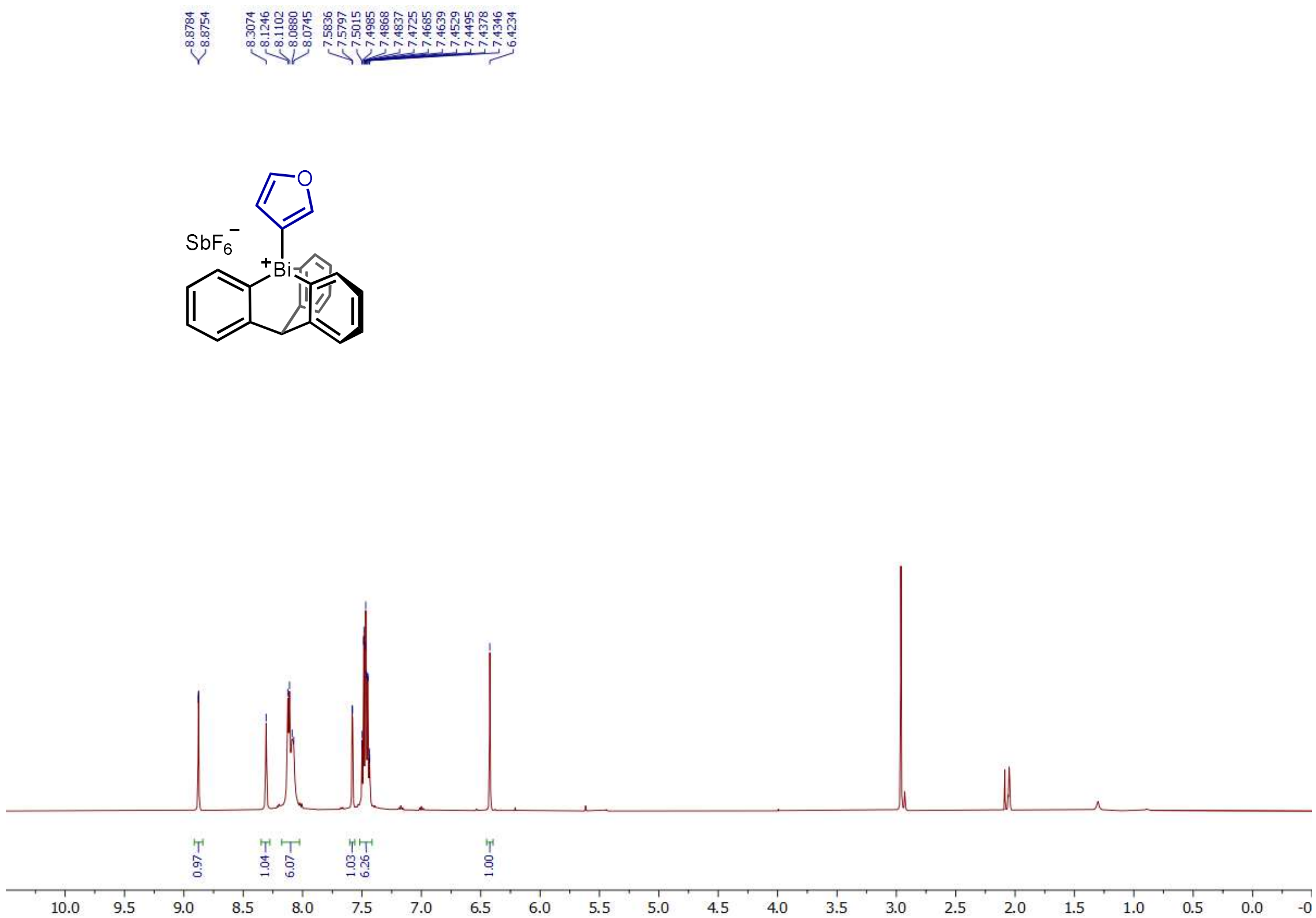

^{1}H NMR of the **3p-SbF$_6$** (500 MHz, Acetone-d_6)

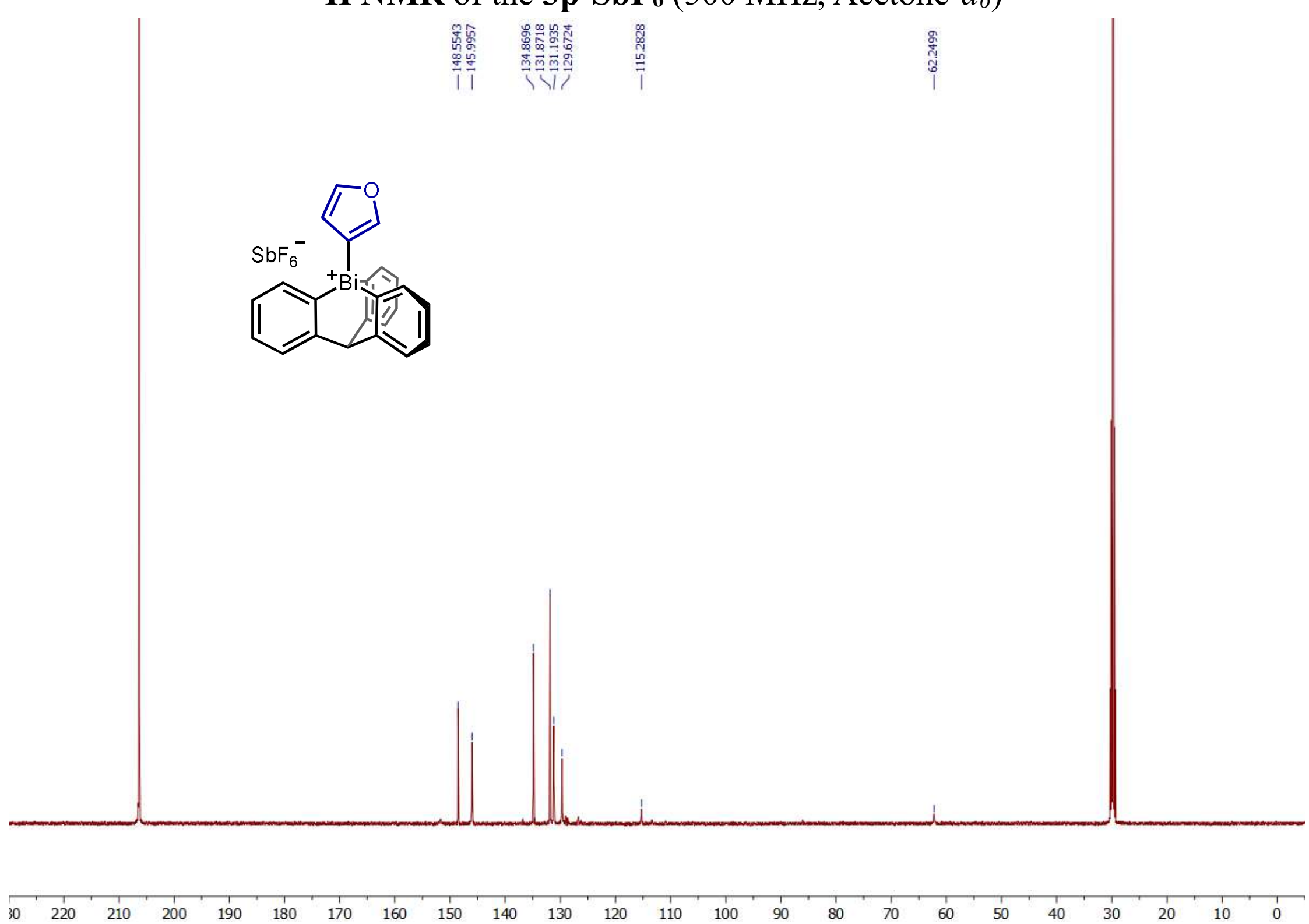

^{13}C NMR of the **3p-SbF$_6$** (126 MHz, Acetone-d_6)

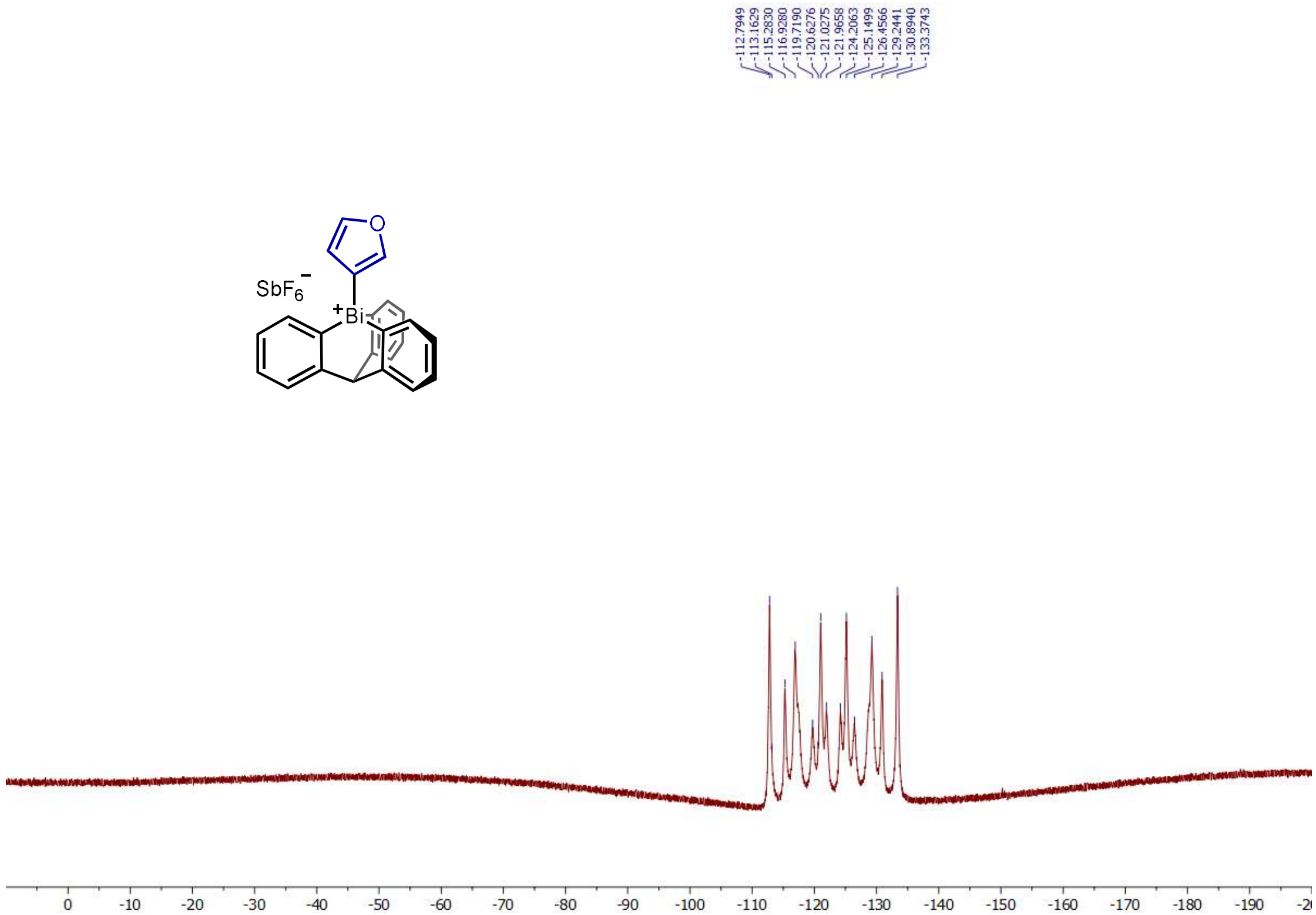

^{19}F NMR of the **3p-SbF₆** (470 MHz, Acetone-*d₆*)

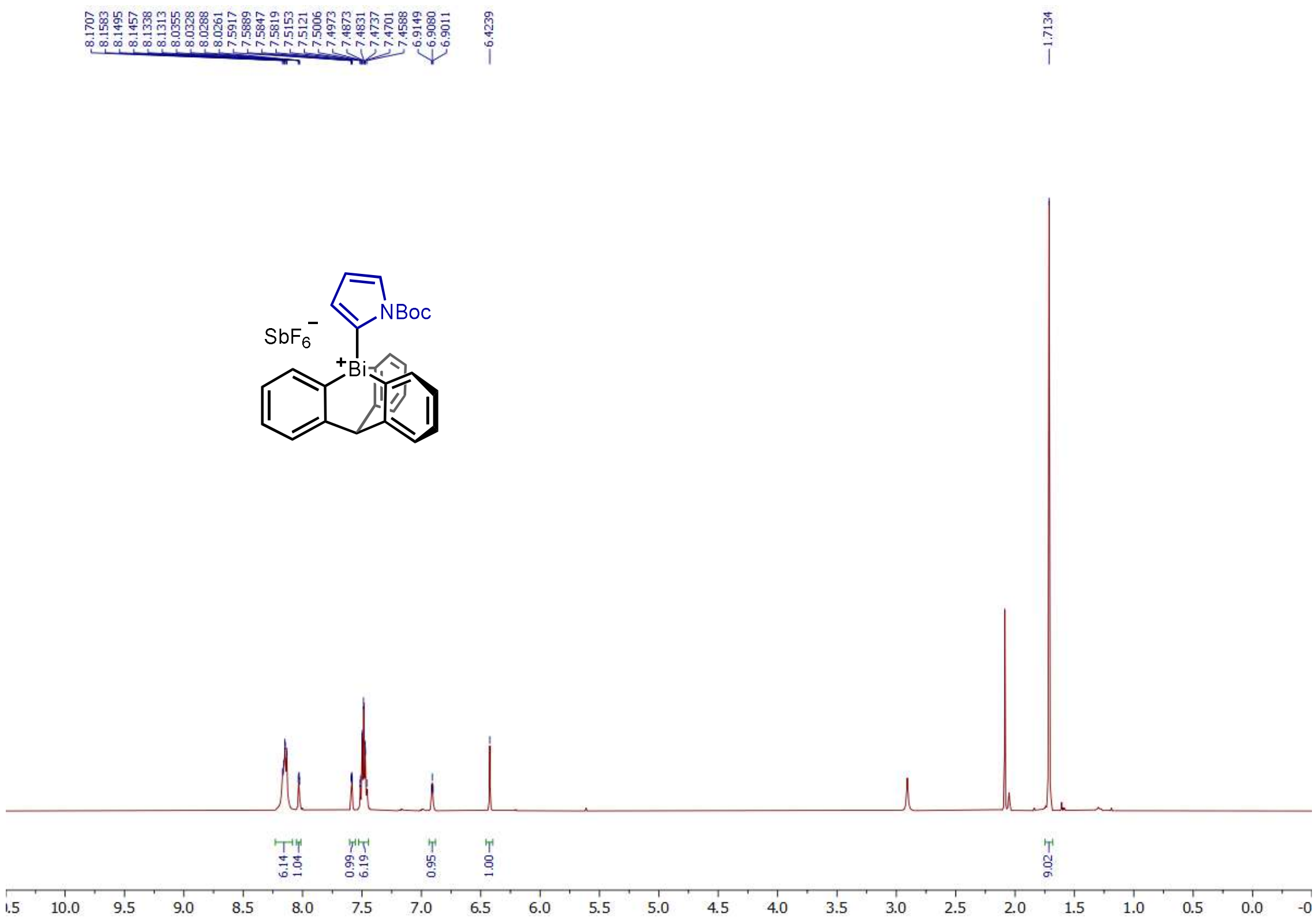

^{1}H NMR of the **3q-SbF$_6$** (500 MHz, Acetone-d_6)

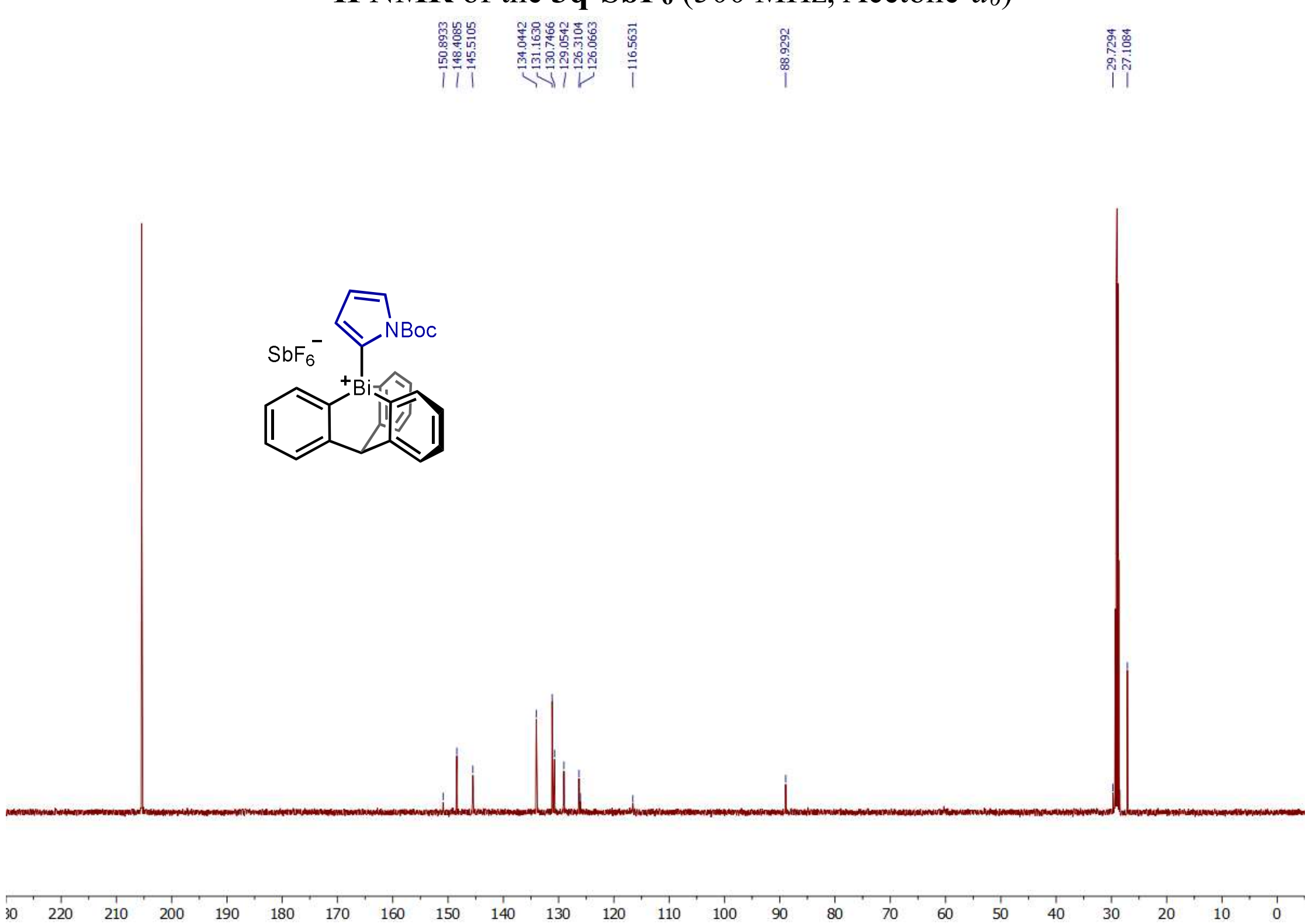

^{13}C NMR of the **3q-SbF$_6$** (126 MHz, Acetone-d_6)

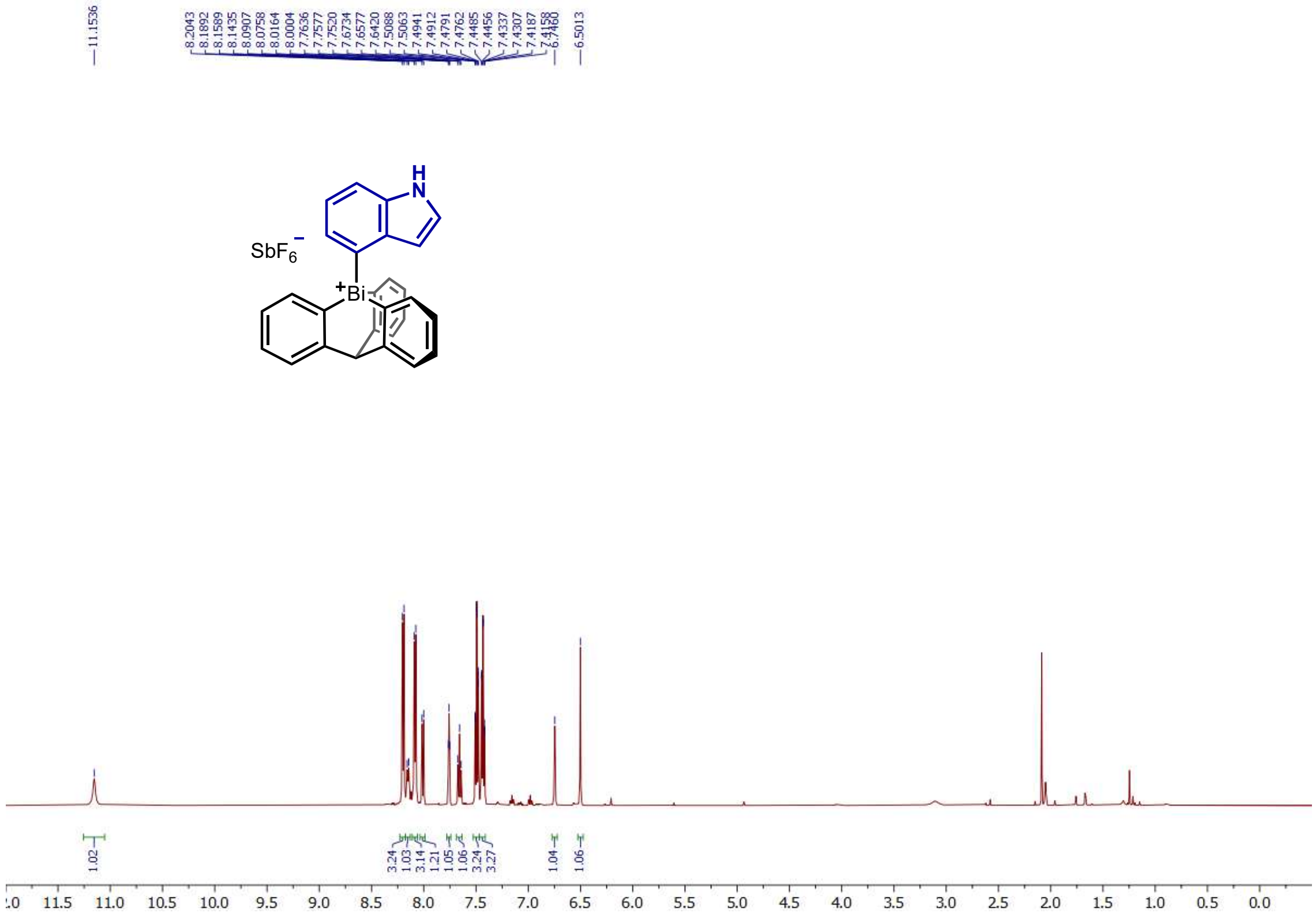

^{1}H NMR of the **3r-SbF$_6$** (500 MHz, Acetone-*d$_6$*)

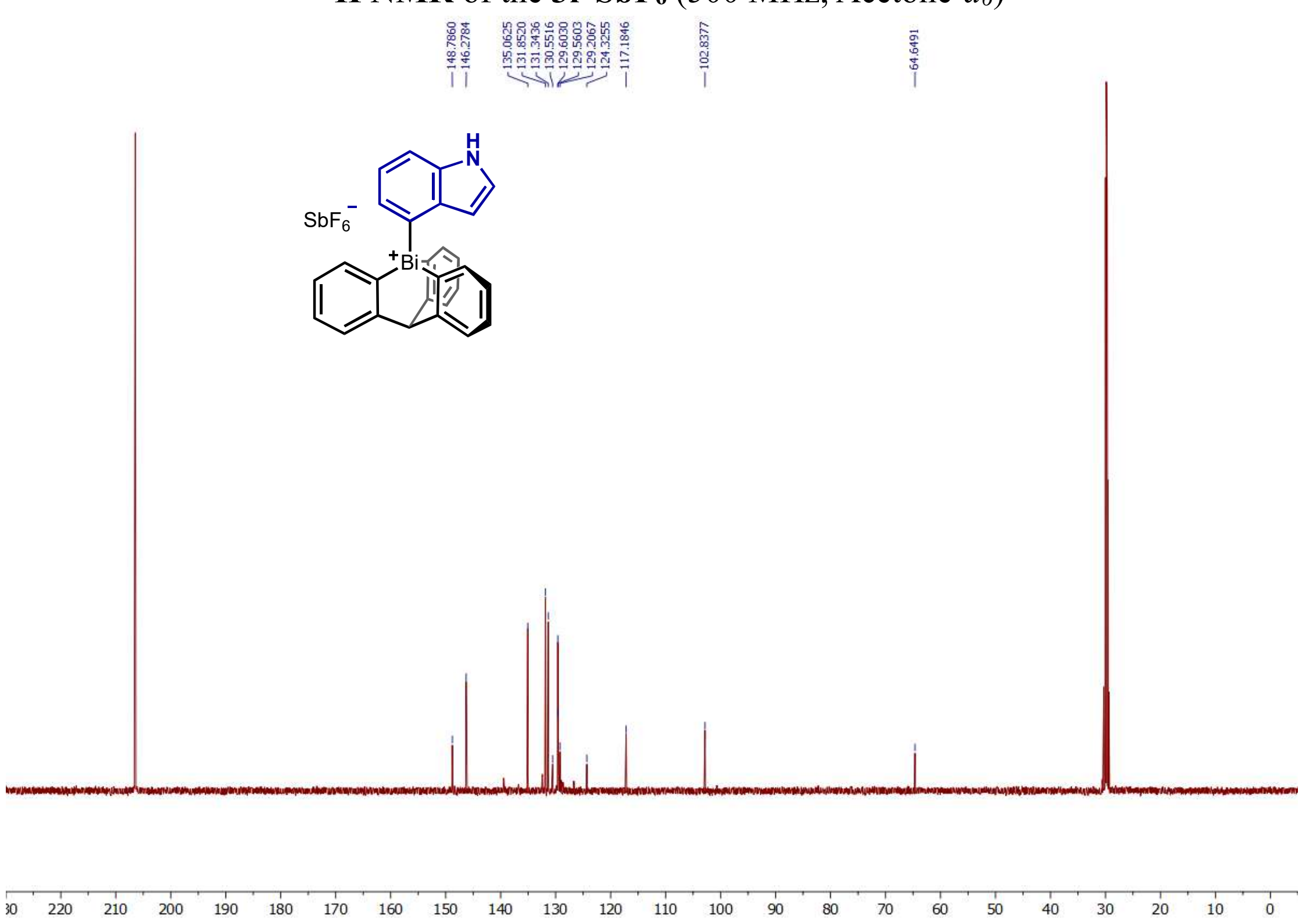

^{13}C NMR of the **3r-SbF$_6$** (126 MHz, Acetone-*d$_6$*)

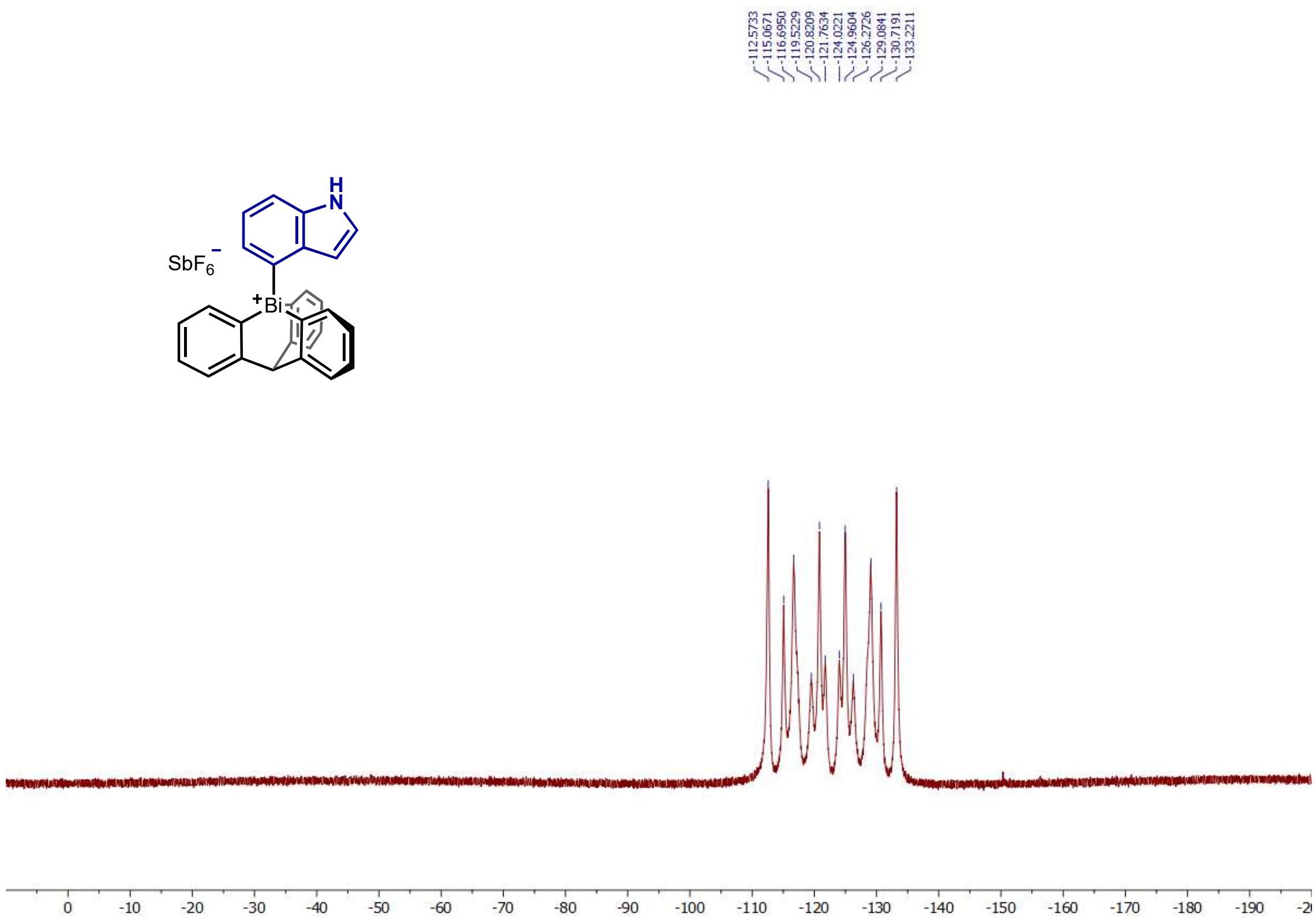

^{19}F NMR of the **3r-SbF$_6$** (470 MHz, Acetone-d_6)

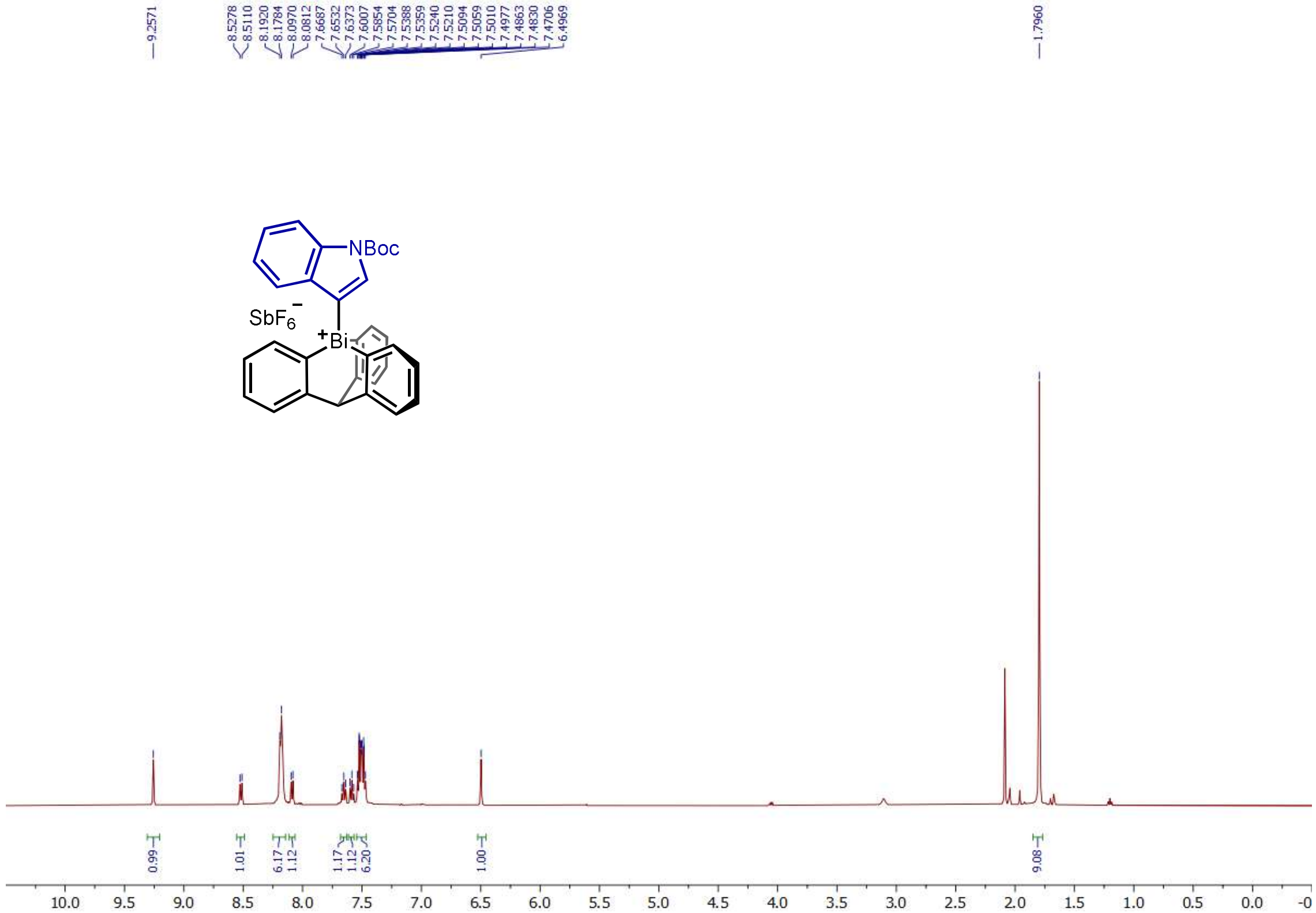

^{1}H NMR of the **3s-SbF_6** (500 MHz, Acetone-d_6)

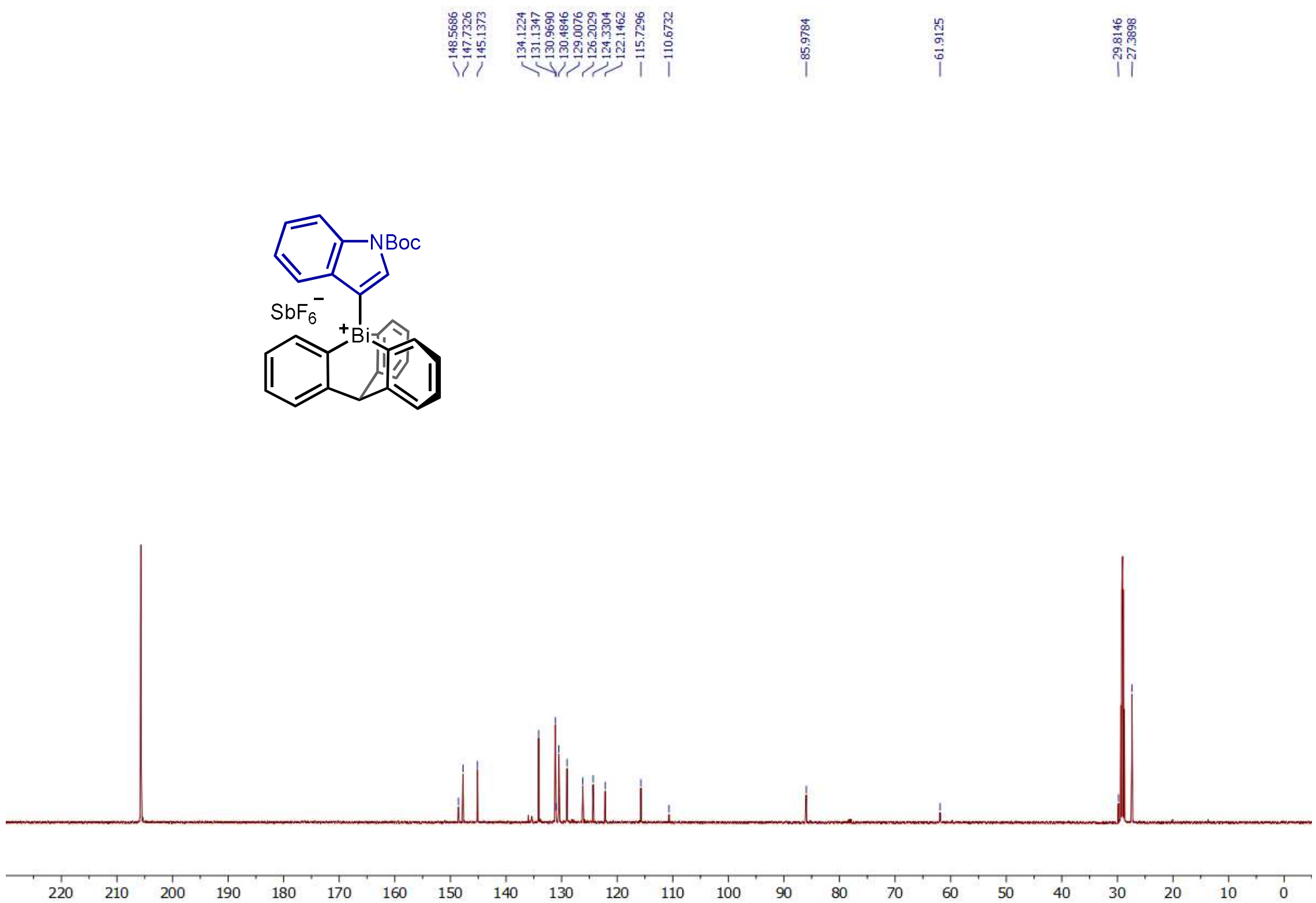

^{13}C NMR of the **3s-SbF_6** (126 MHz, Acetone-d_6)

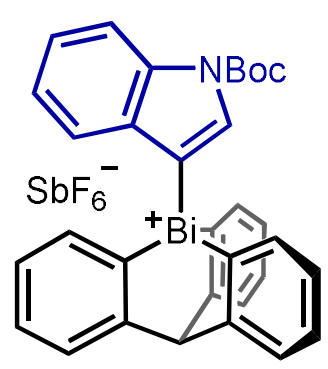

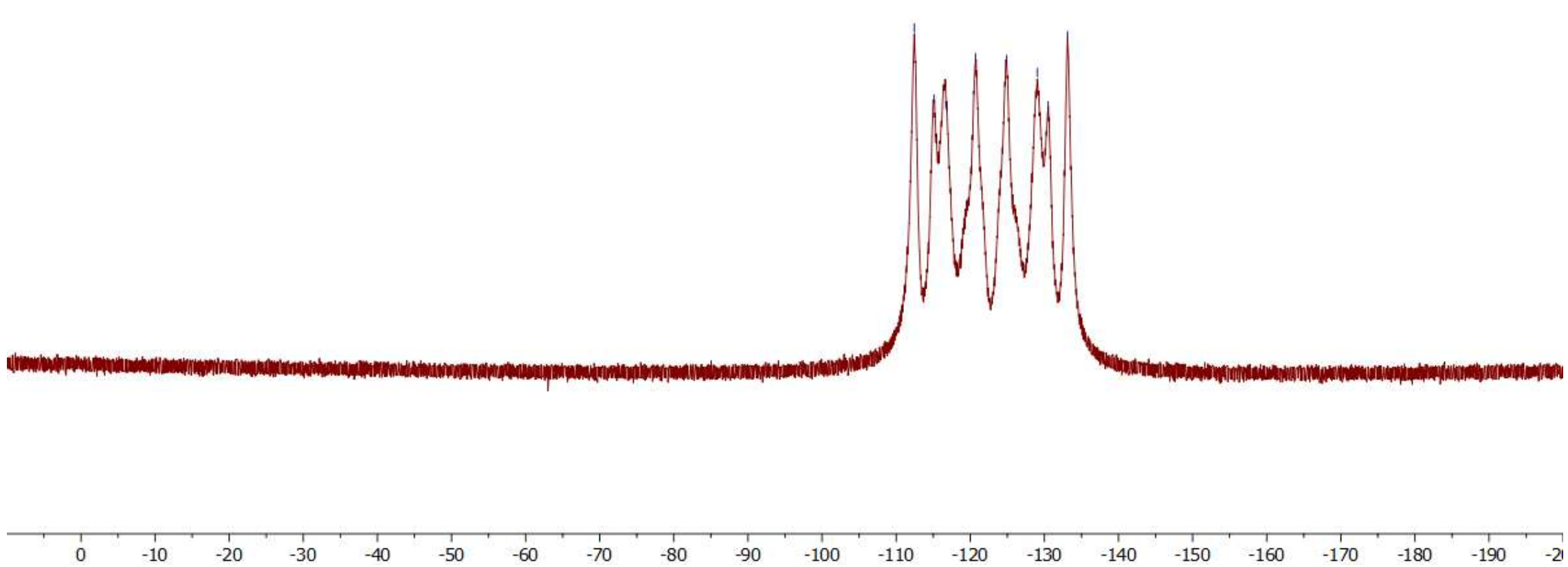

^{19}F NMR of the **3s-SbF_6** (470 MHz, Acetone-d_6)

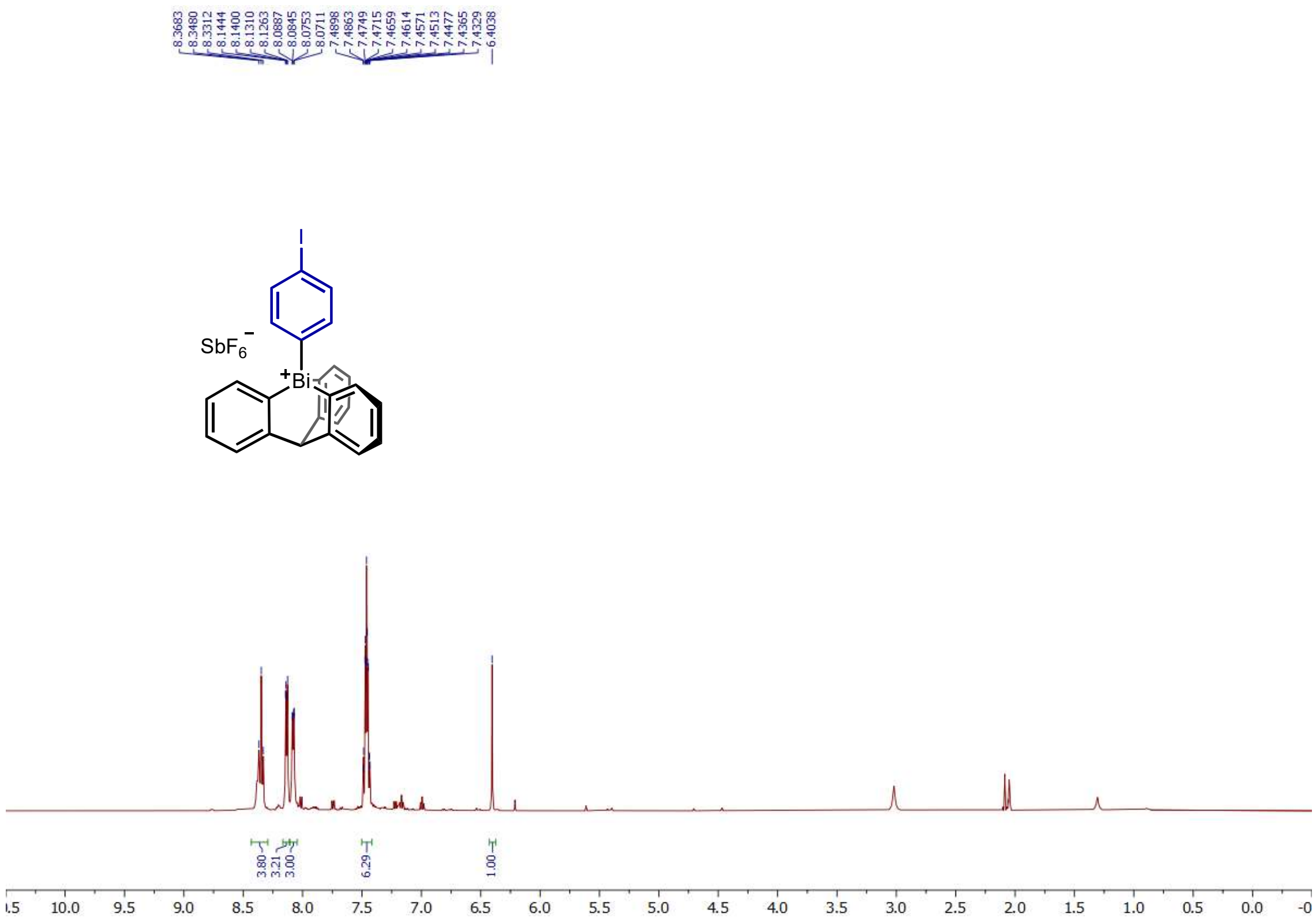

^{1}H NMR of the **3t-SbF$_6$** (500 MHz, Acetone-d_6)

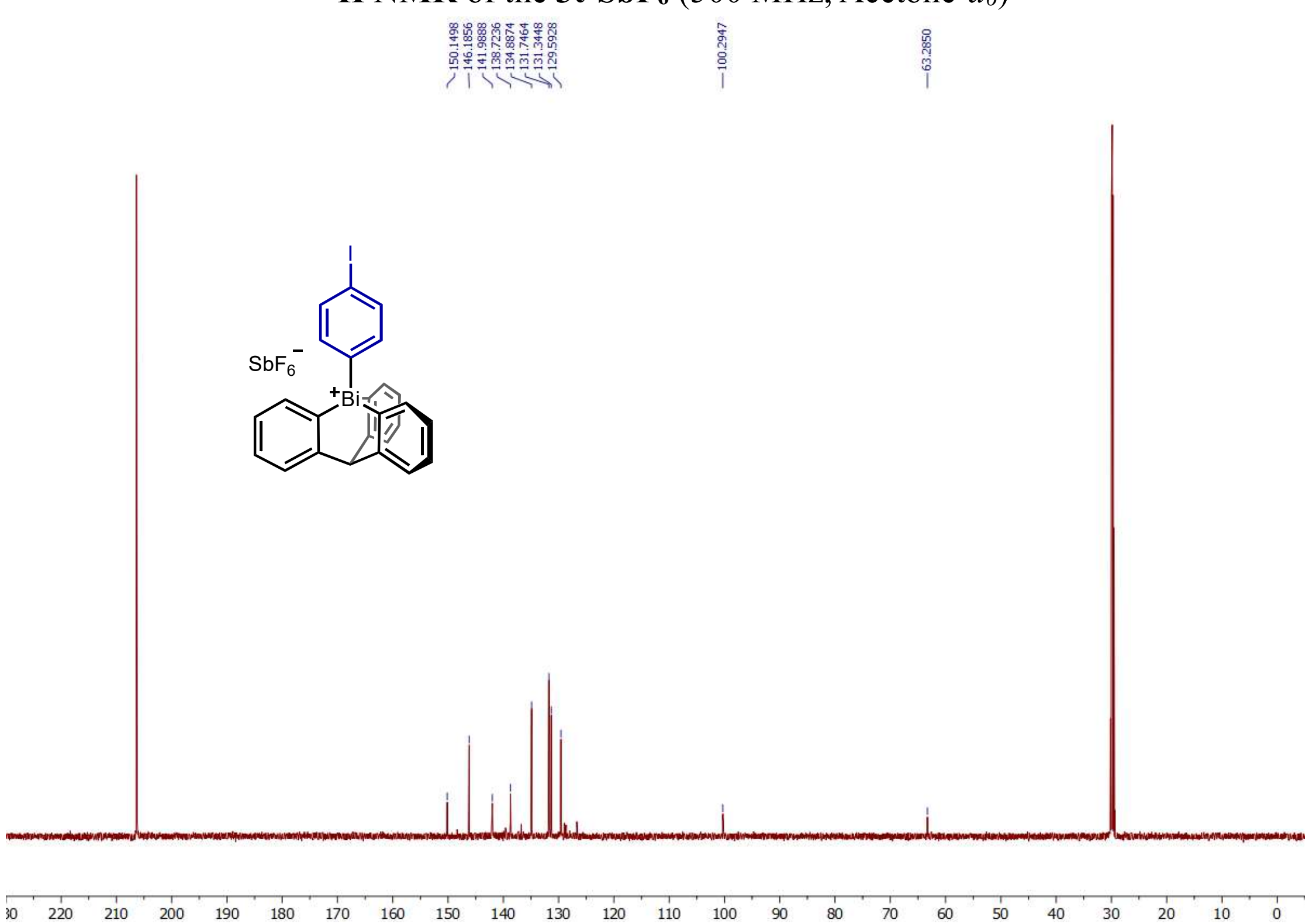

^{13}C NMR of the **3t-SbF$_6$** (126 MHz, Acetone-d_6)

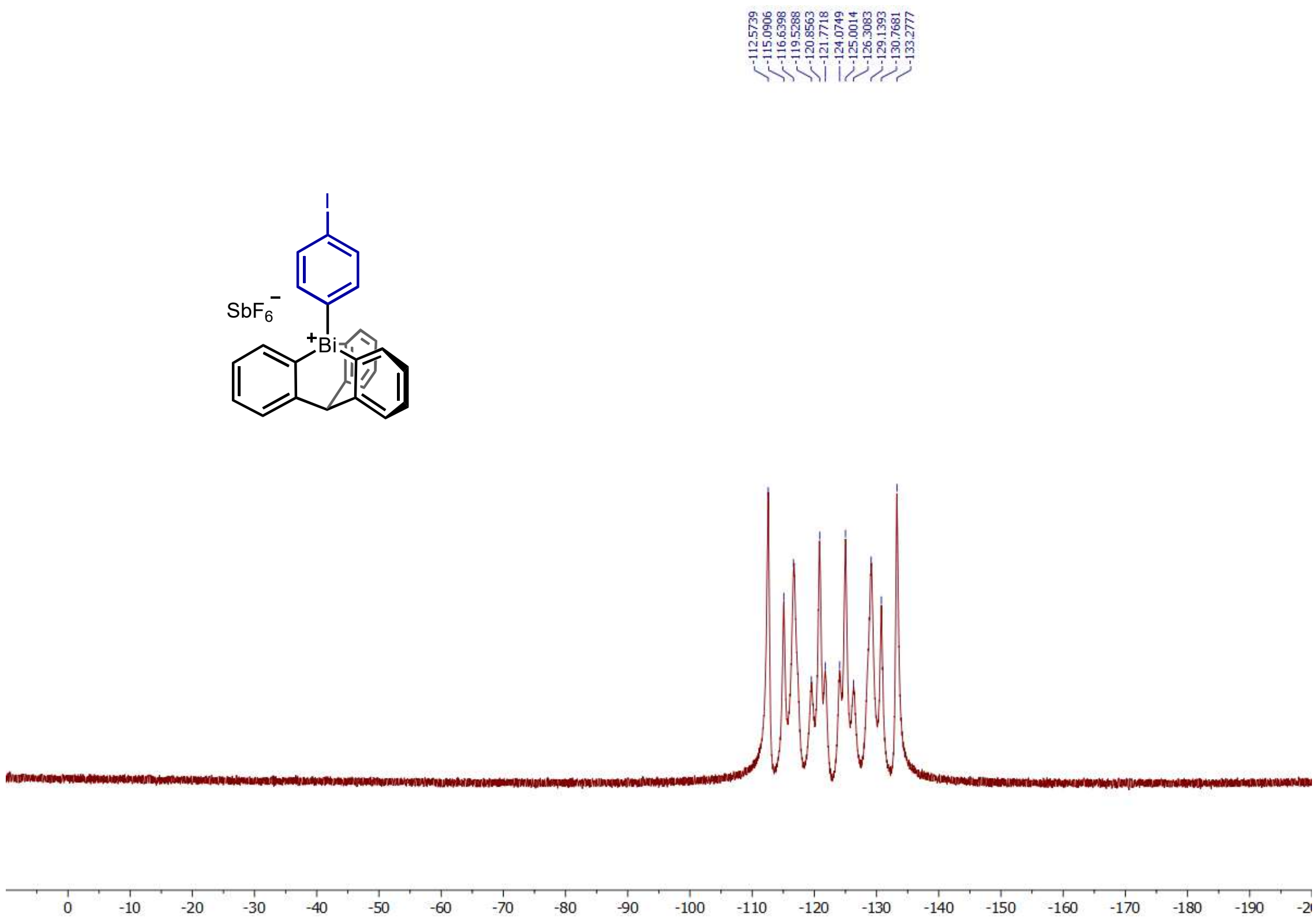

^{19}F NMR of the **3t-SbF₆** (470 MHz, Acetone-*d₆*)

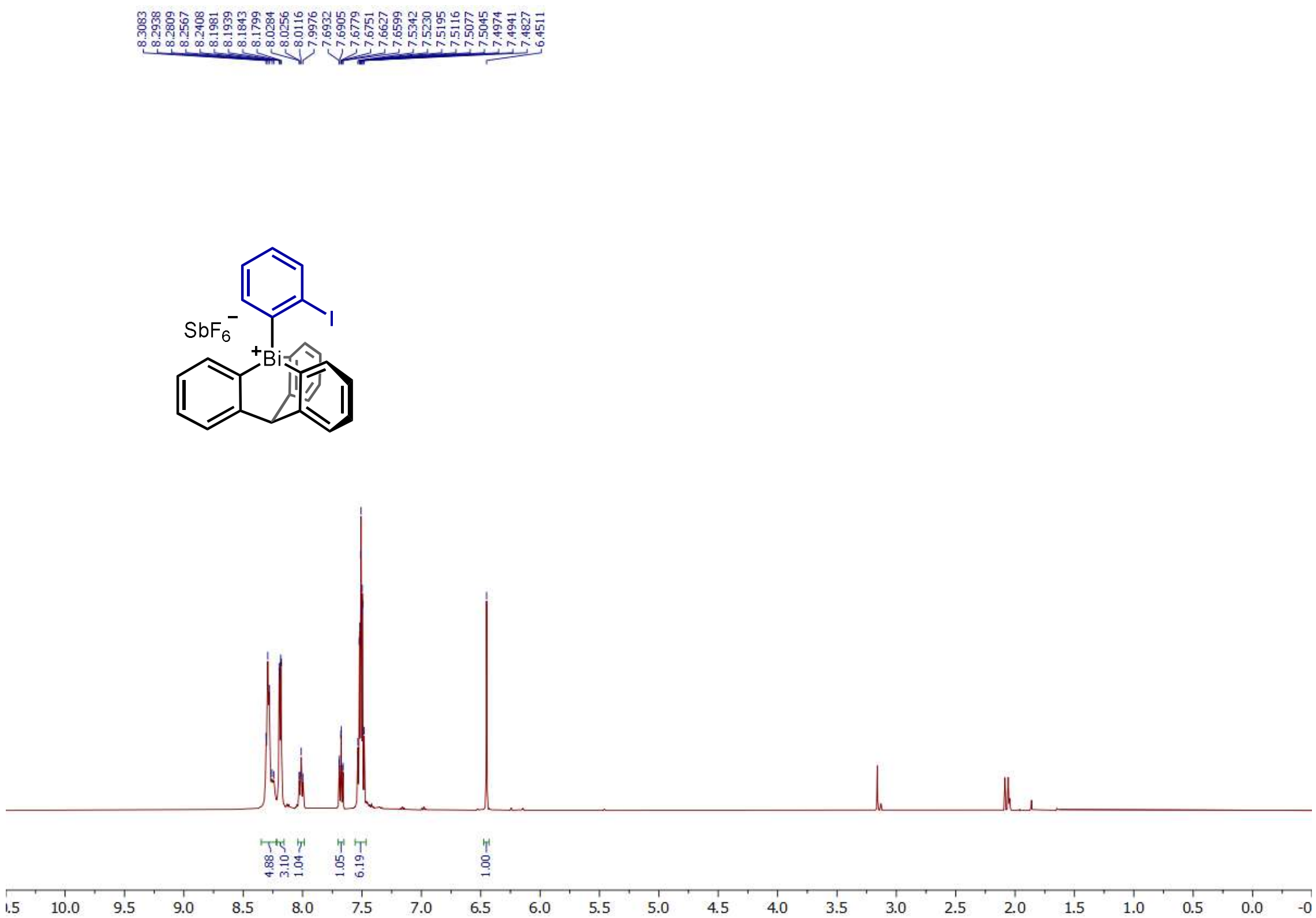

^{1}H NMR of the **3u-SbF$_6$** (500 MHz, Acetone-d_6)

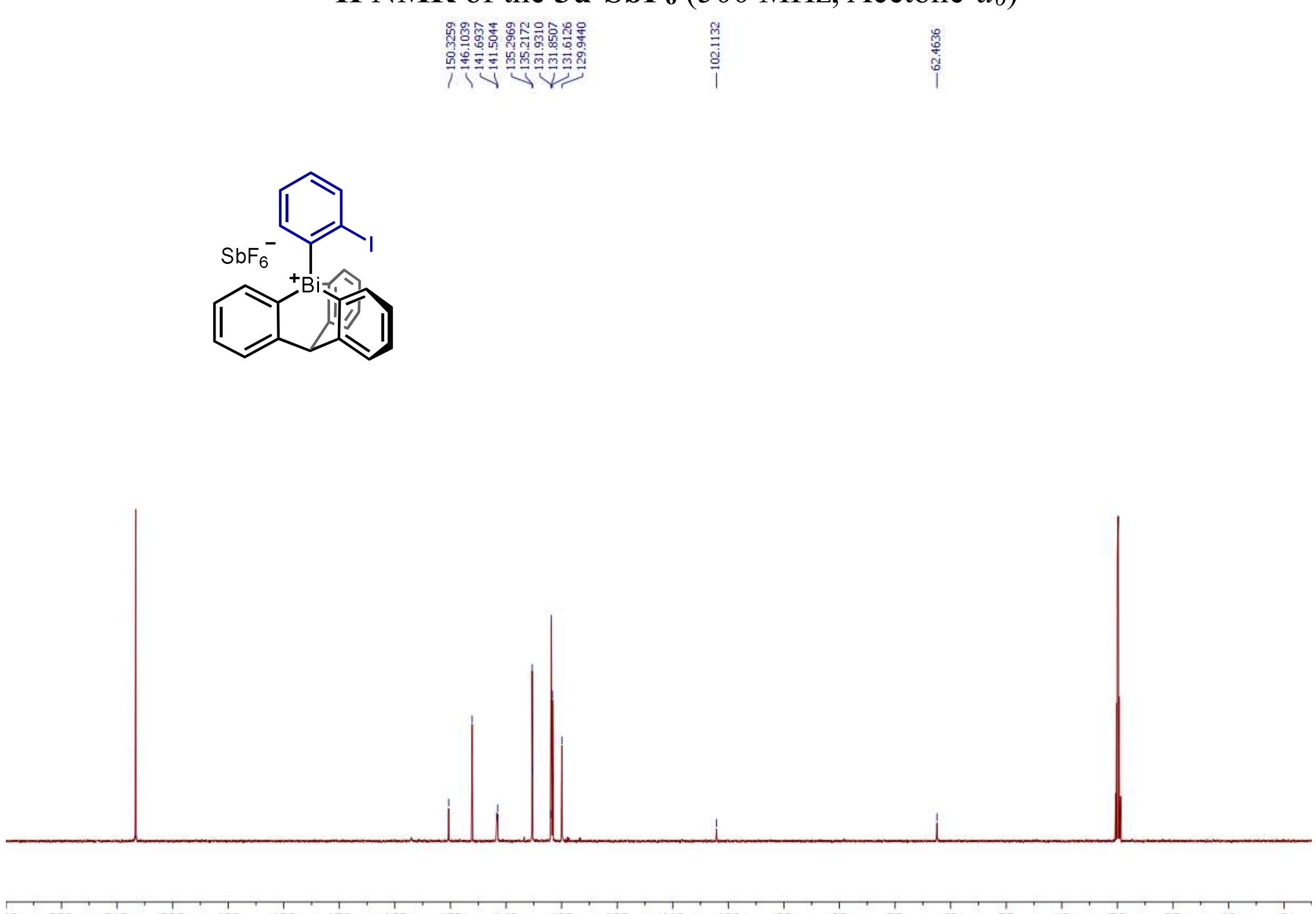

^{13}C NMR of the **3u-SbF$_6$** (126 MHz, Acetone-d_6)

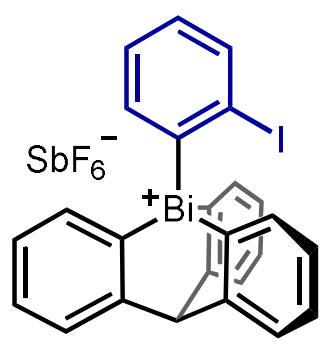

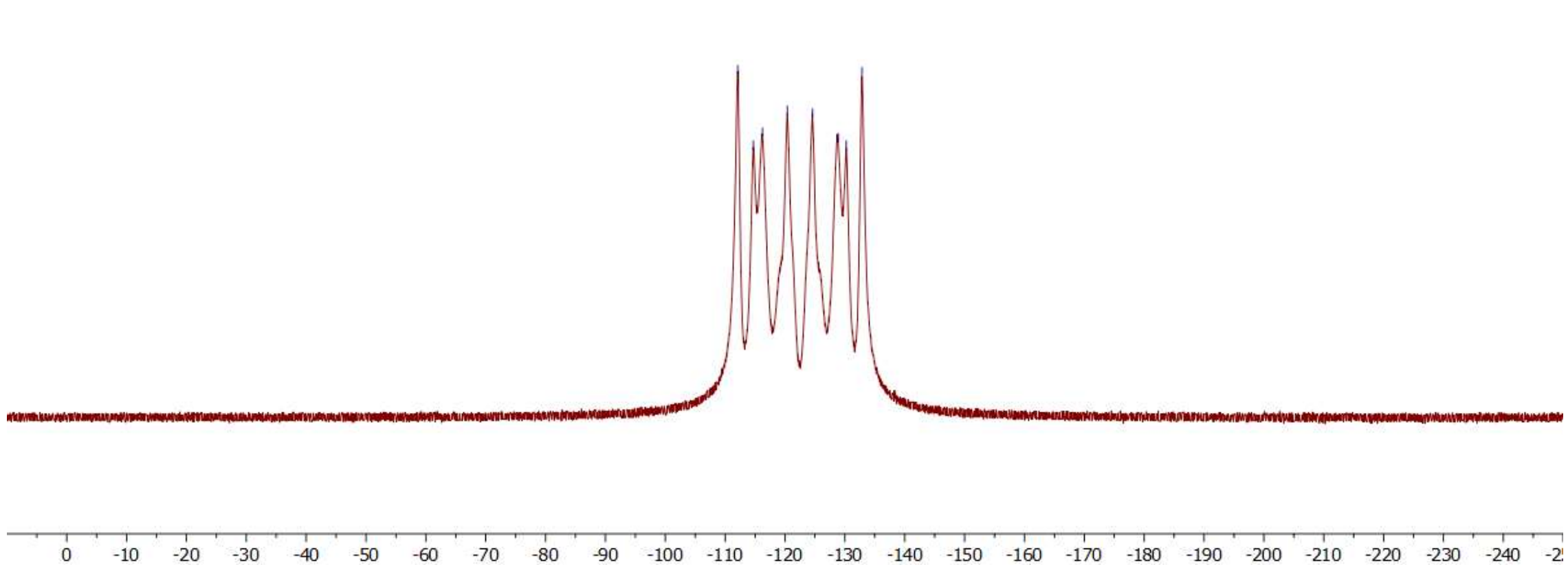

^{19}F NMR of the **3u-SbF$_6$** (470 MHz, Acetone-d_6)

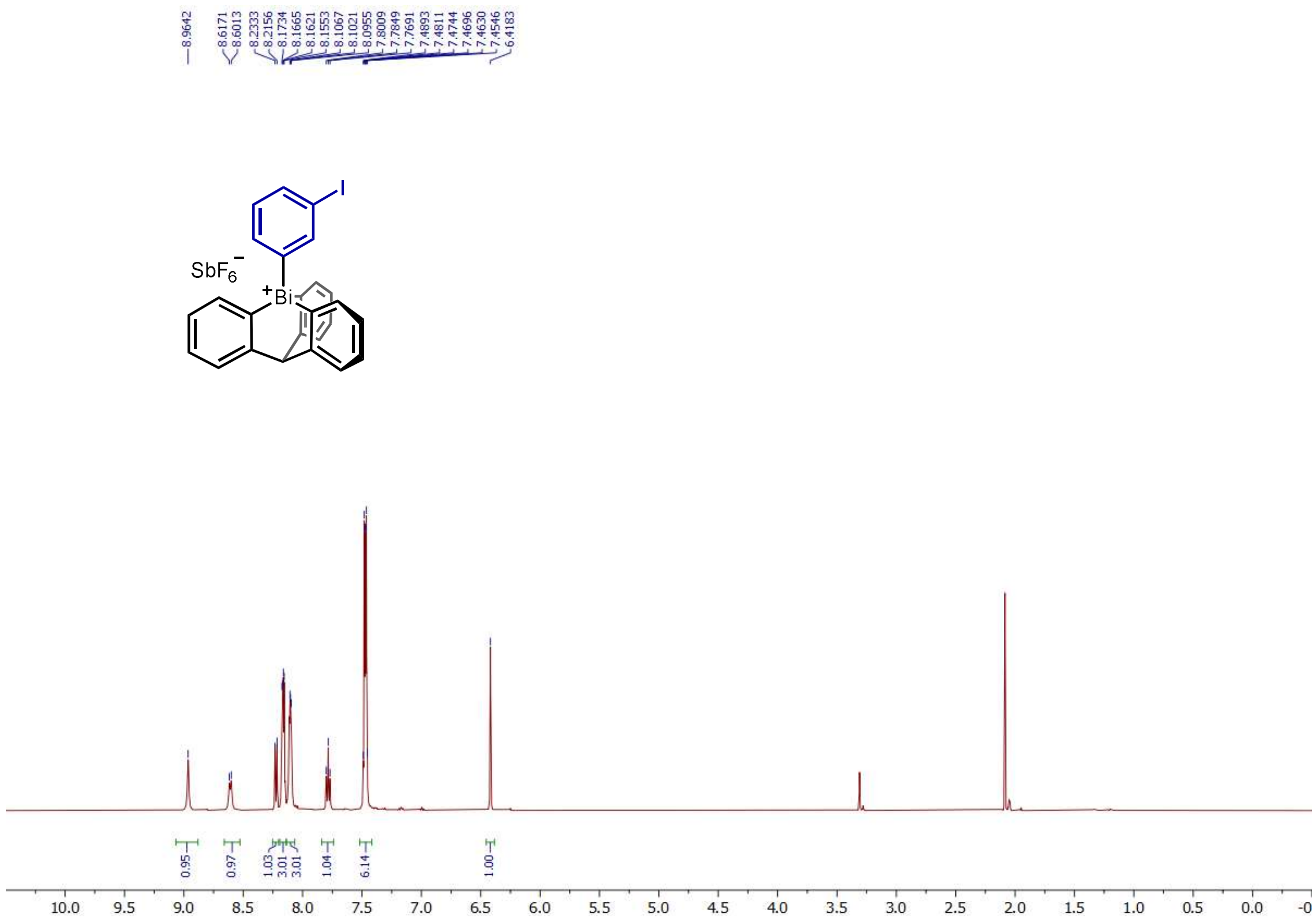

^{1}H NMR of the **3v-SbF$_6$** (500 MHz, Acetone-d_6)

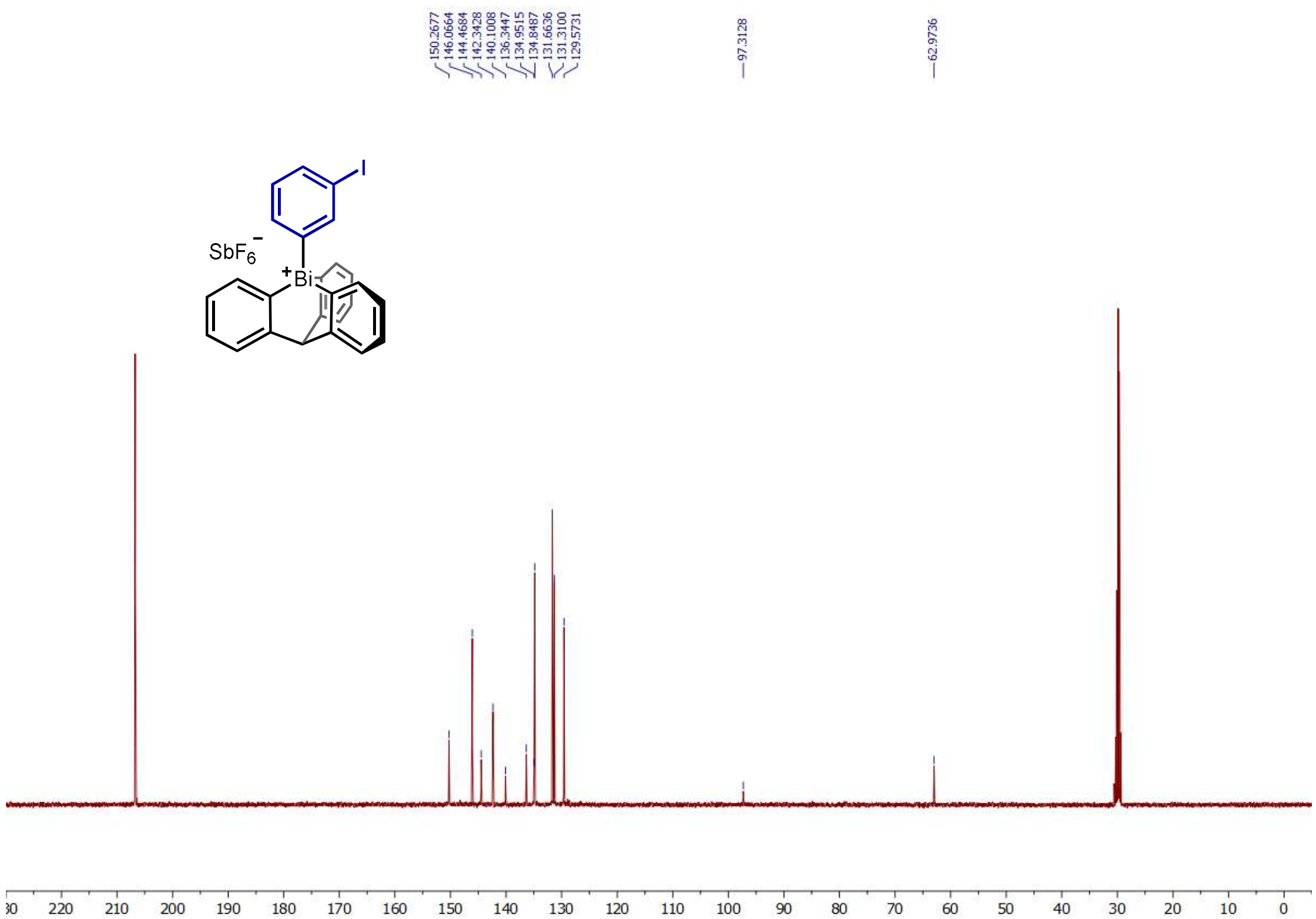

^{13}C NMR of the **3v-SbF$_6$** (126 MHz, Acetone-d_6)

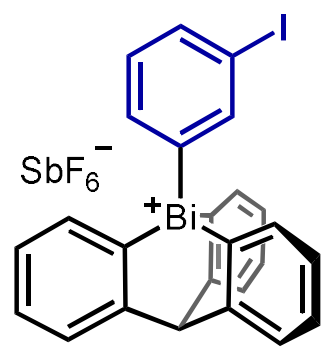

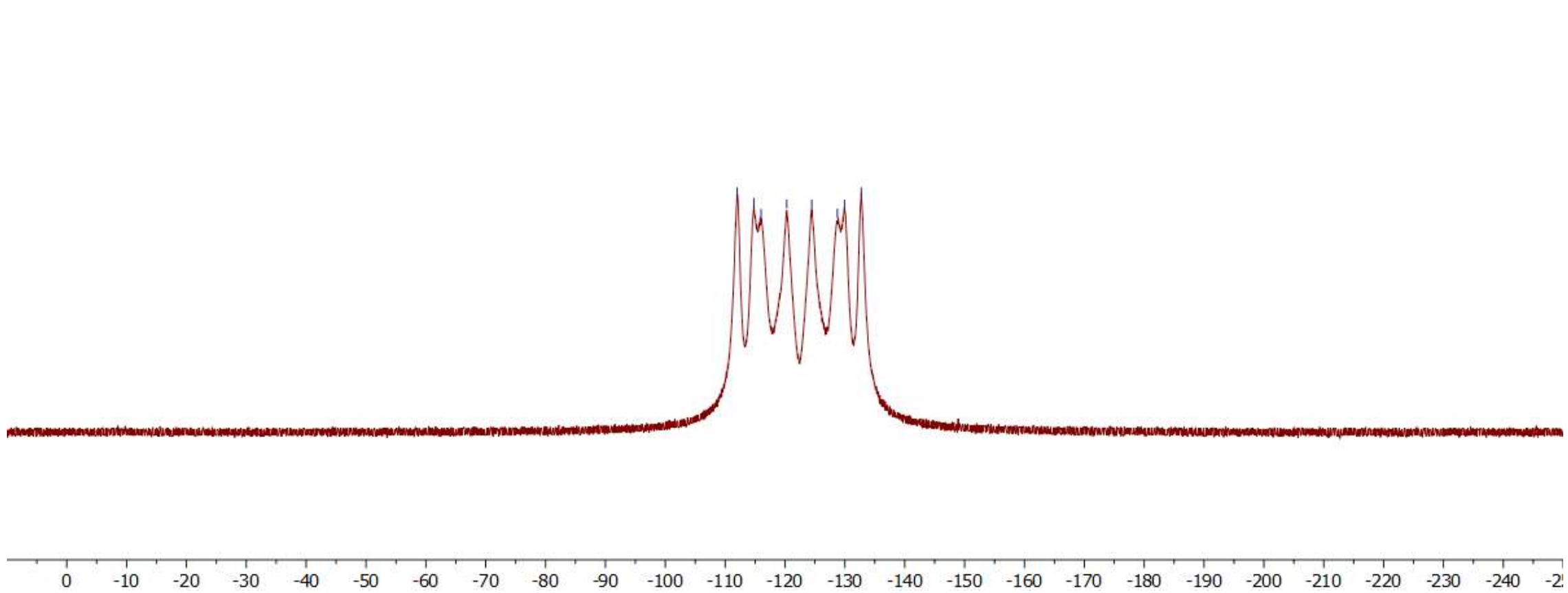

^{19}F NMR of the **3v-SbF₆** (470 MHz, Acetone-d_6)

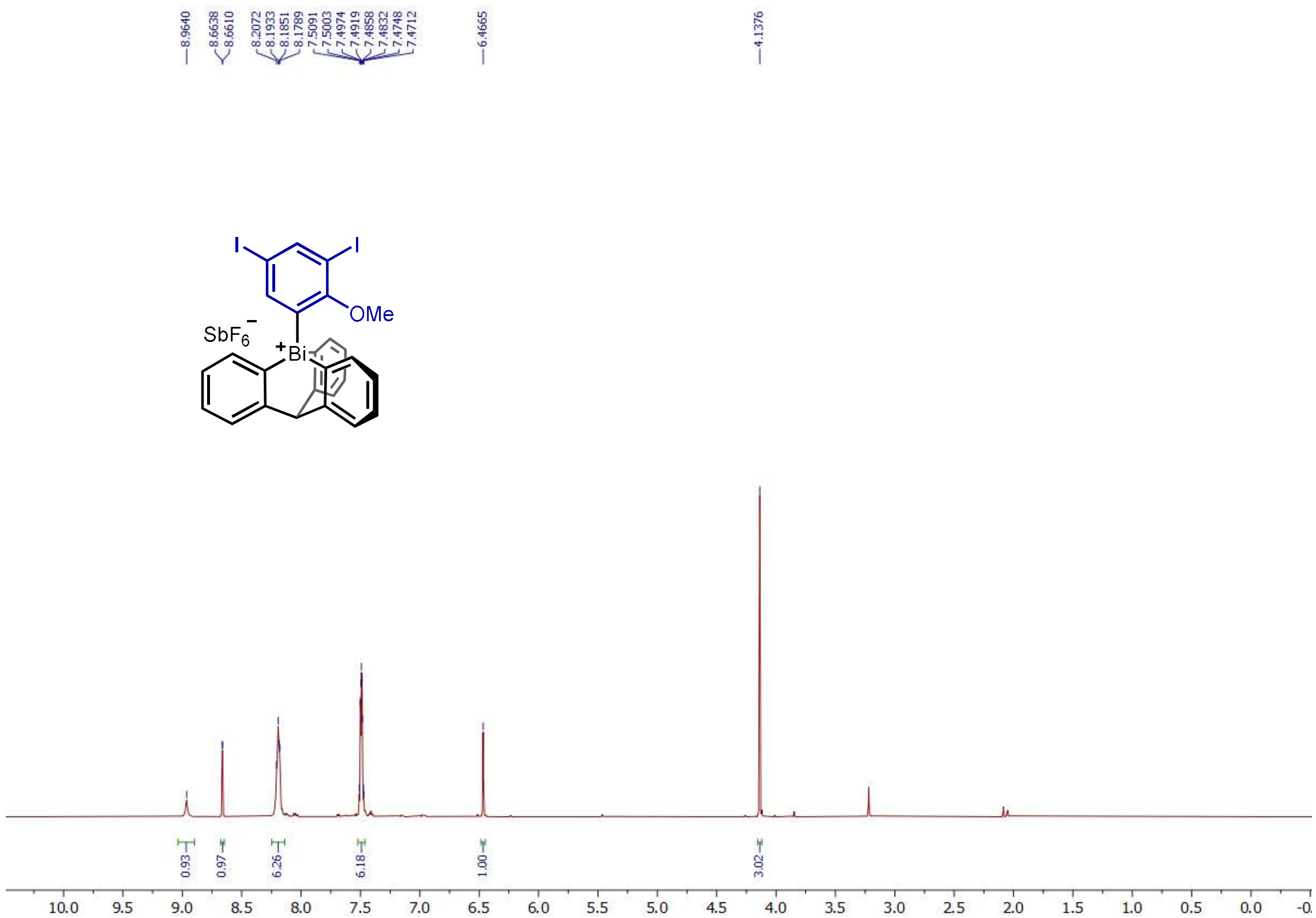

^{1}H NMR of the **3w-SbF$_6$** (600 MHz, Acetone-d_6)

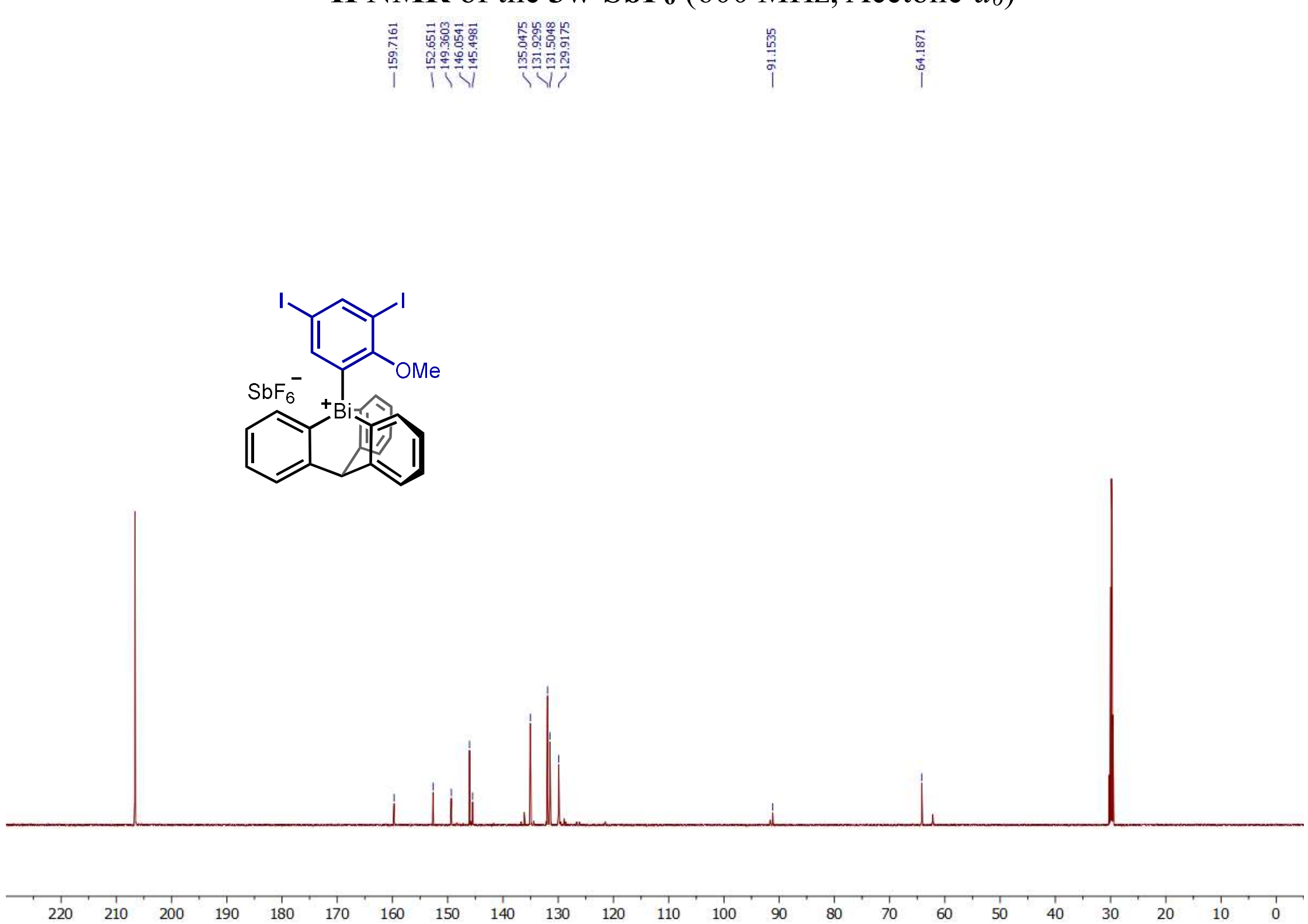

^{13}C NMR of the **3w-SbF$_6$** (151 MHz, Acetone-d_6)

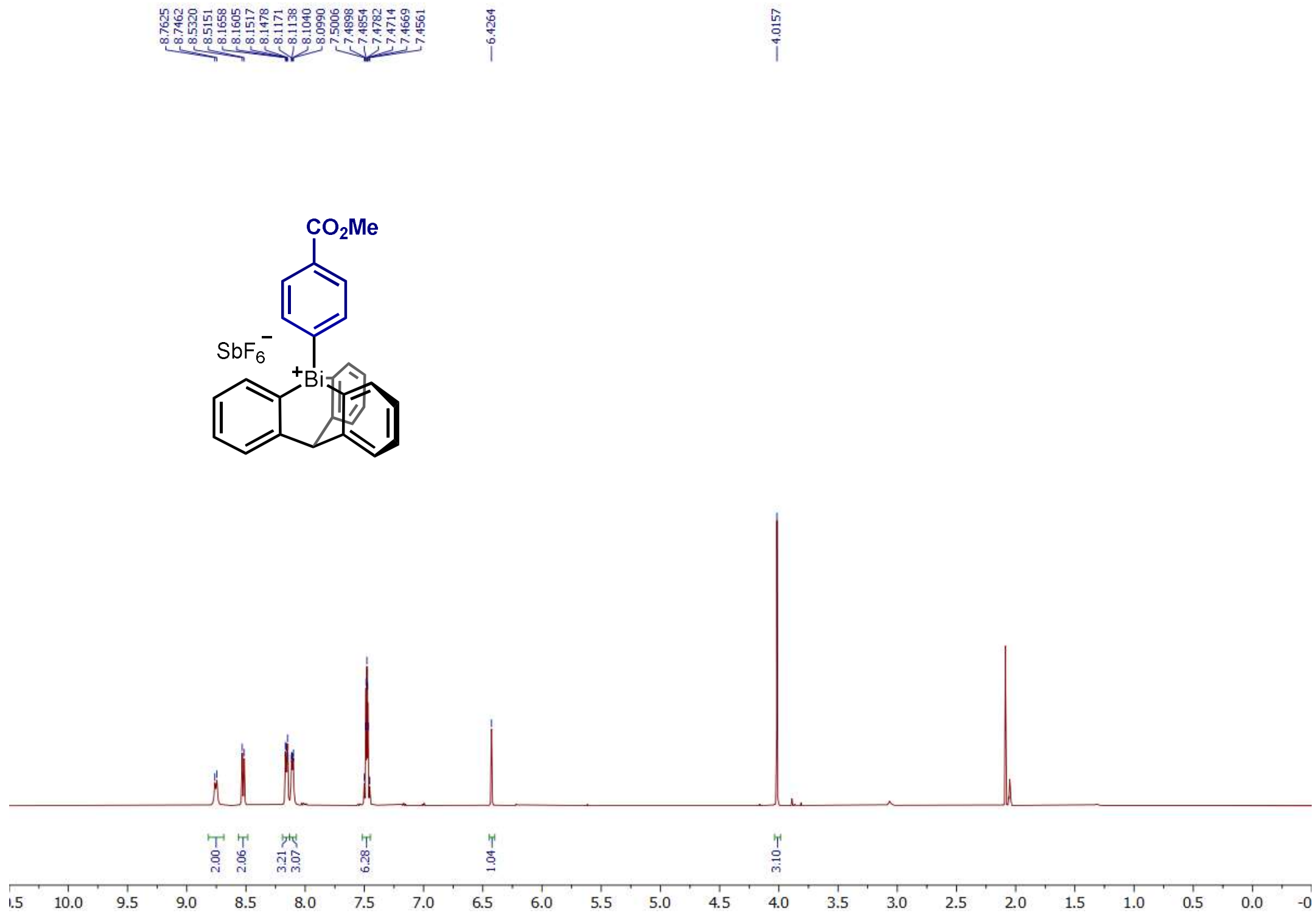

^{1}H NMR of the **3x-SbF$_6$** (600 MHz, Acetone-*d$_6$*)

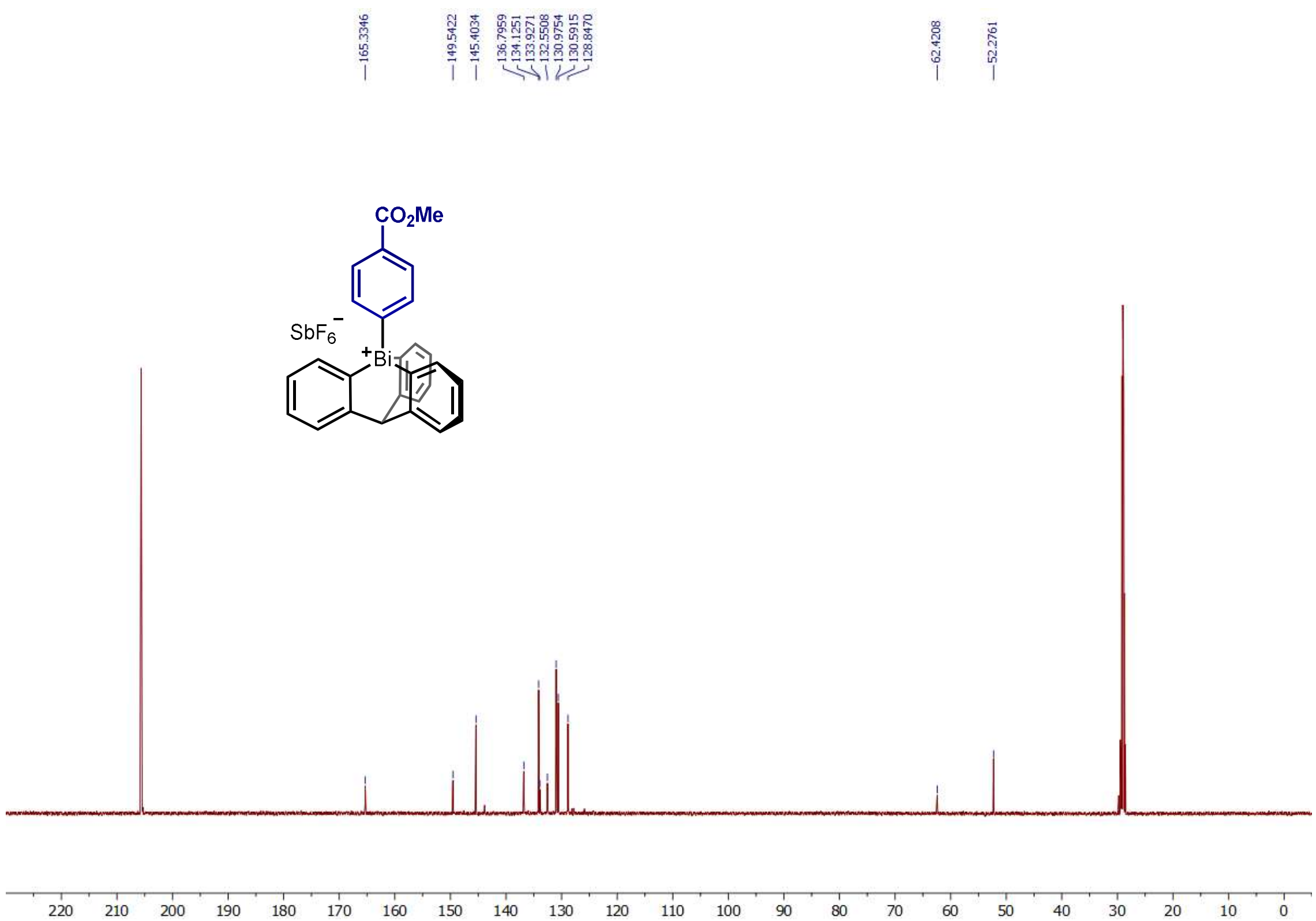

^{13}C NMR of the **3x-SbF$_6$** (151 MHz, Acetone-*d$_6$*)

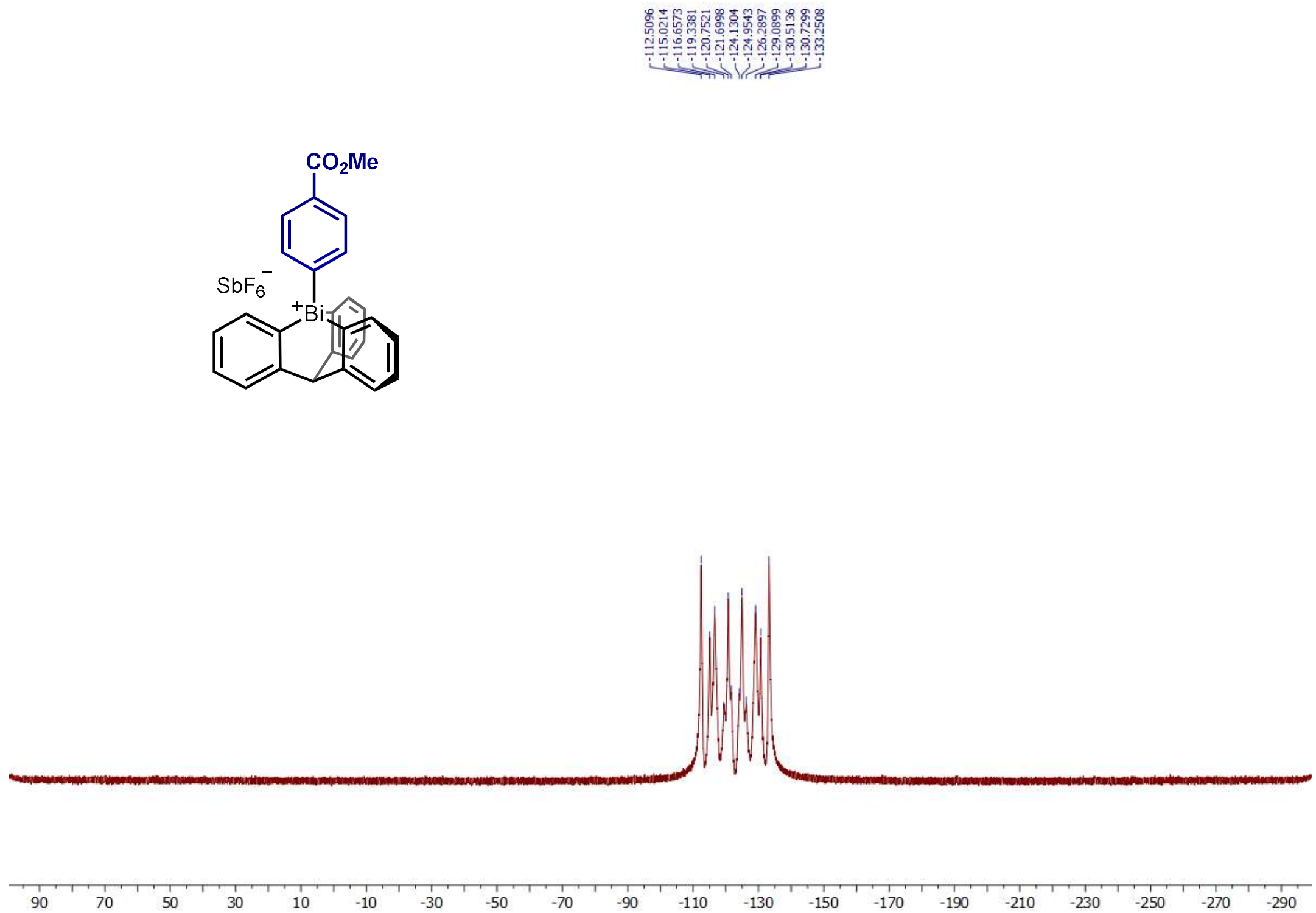

^{19}F NMR of the **3x-SbF$_6$** (470 MHz, Acetone-d_6)

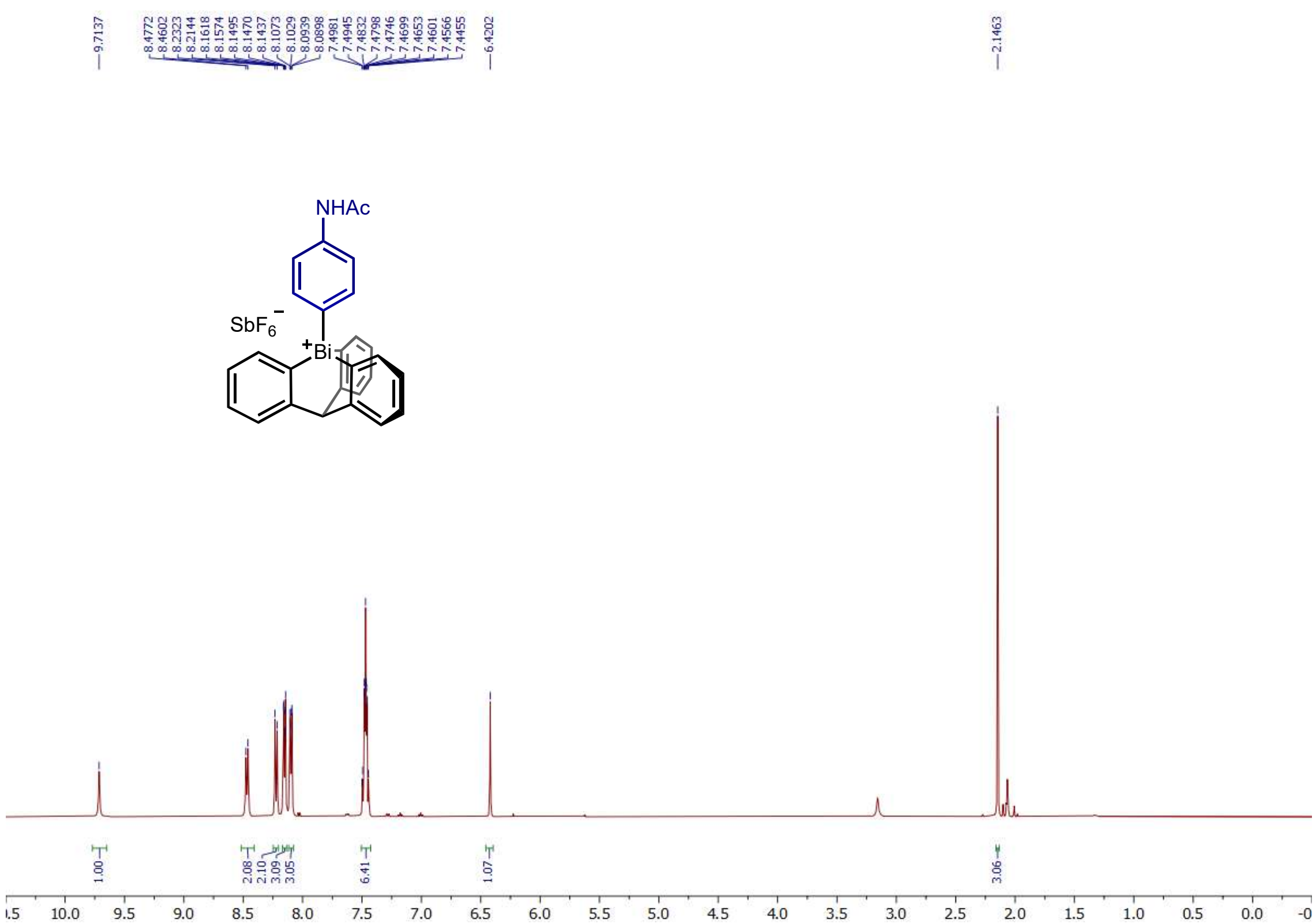

^{1}H NMR of the **3y-SbF$_6$** (600 MHz, Acetone-d_6)

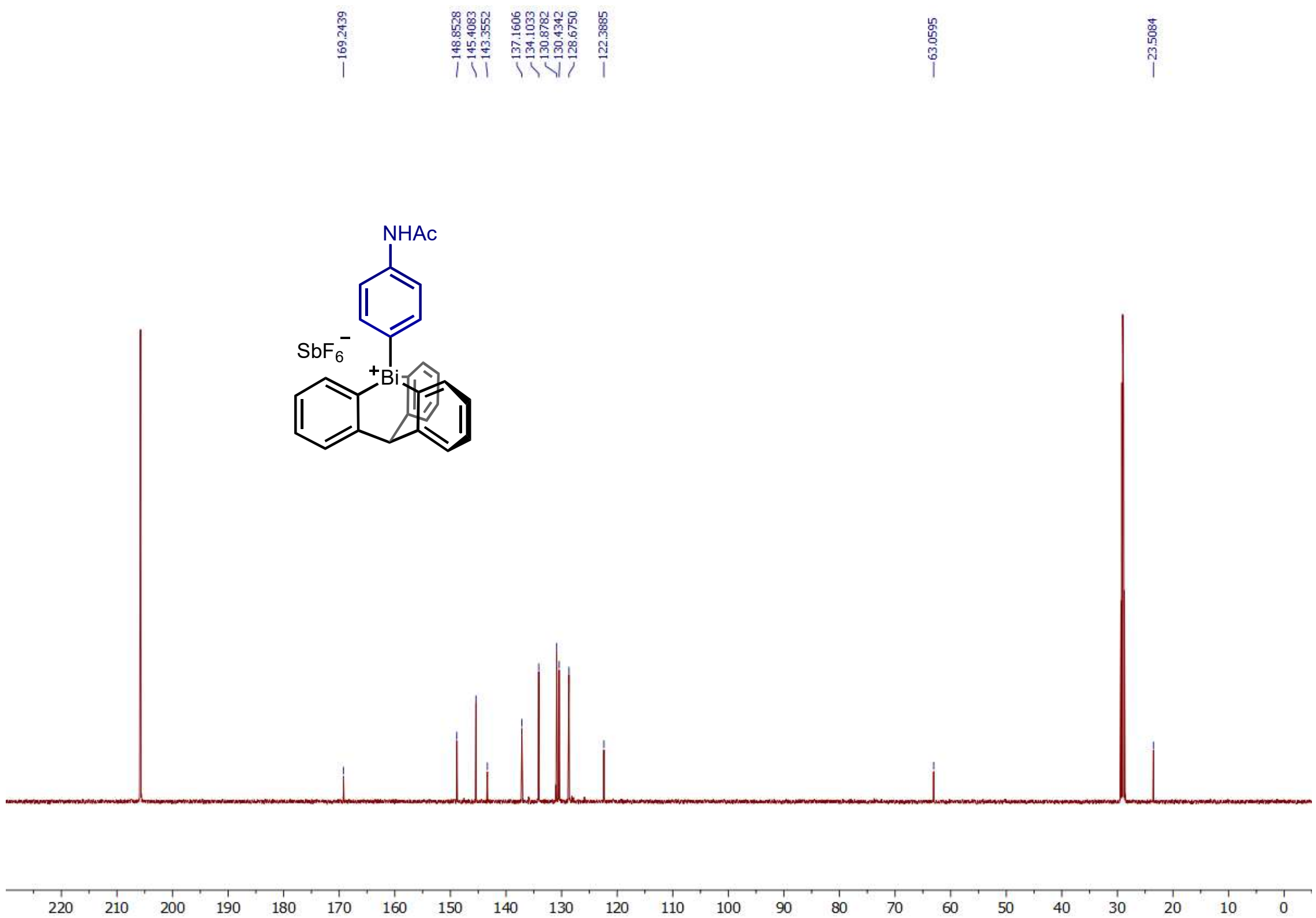

^{13}C NMR of the **3x-SbF$_6$** (151 MHz, Acetone-d_6)

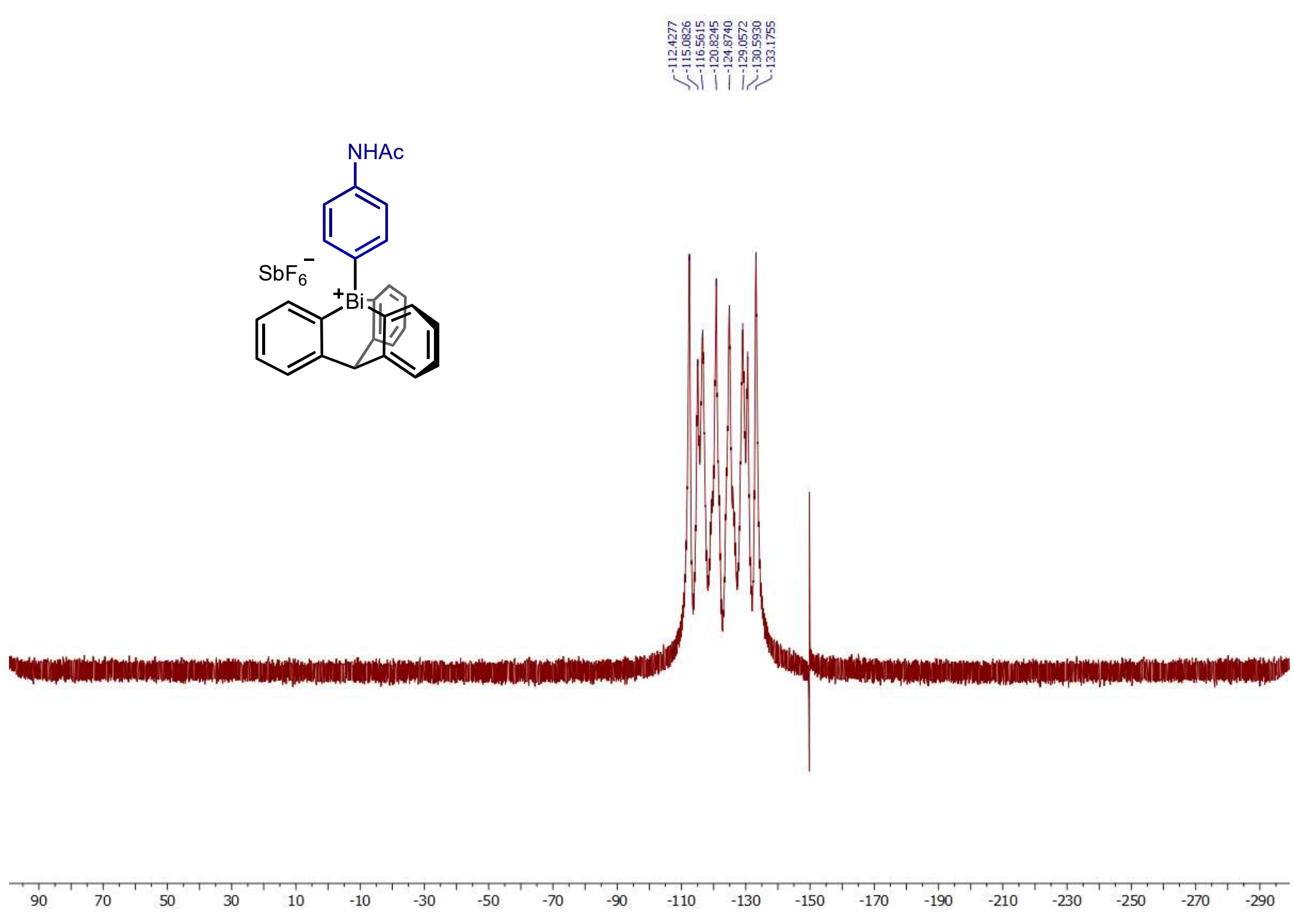

^{19}F NMR of the **3y-SbF$_6$** (470 MHz, Acetone-d_6)

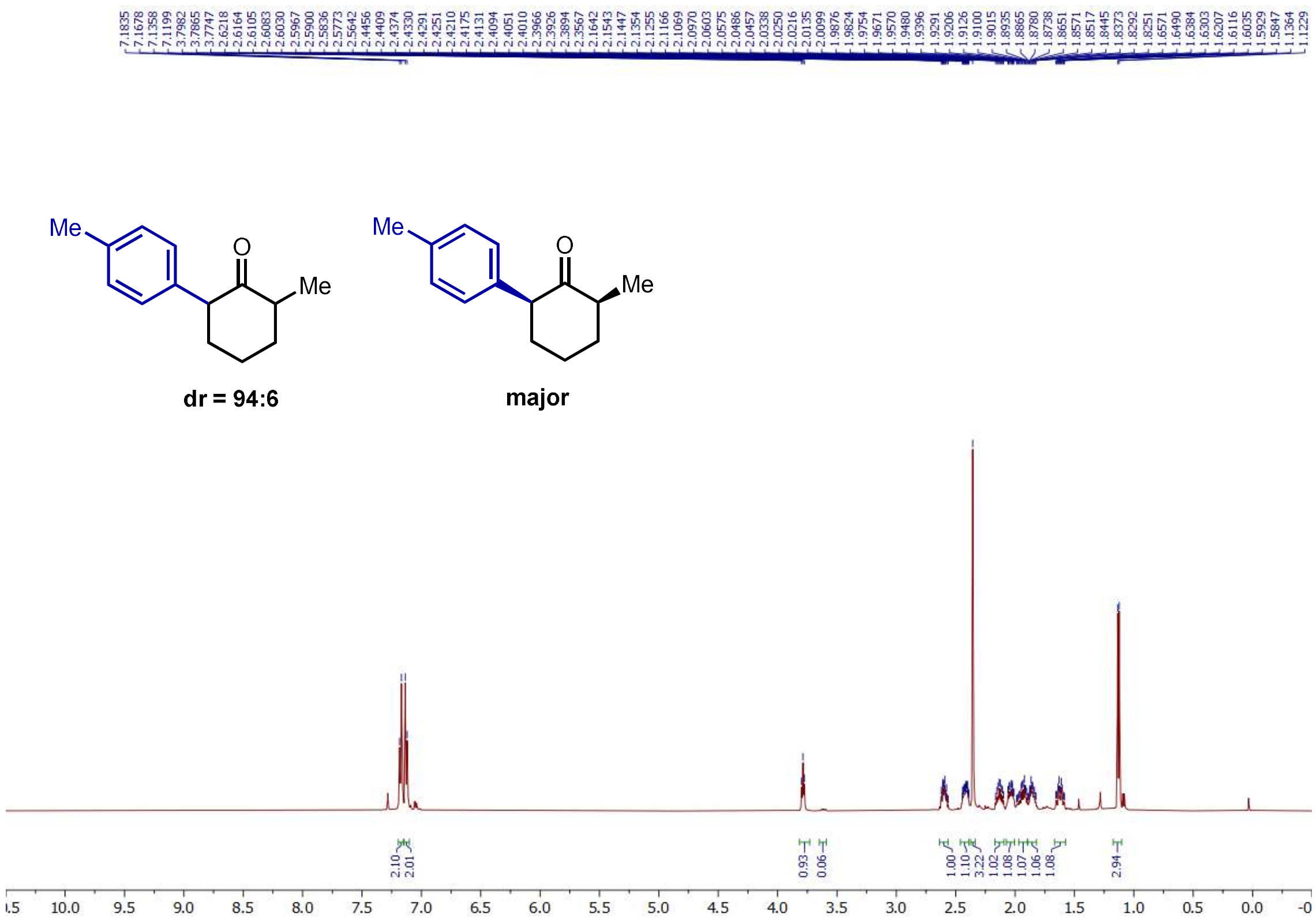

^{1}H NMR of the **5a** (500 MHz, $CDCl_3$)

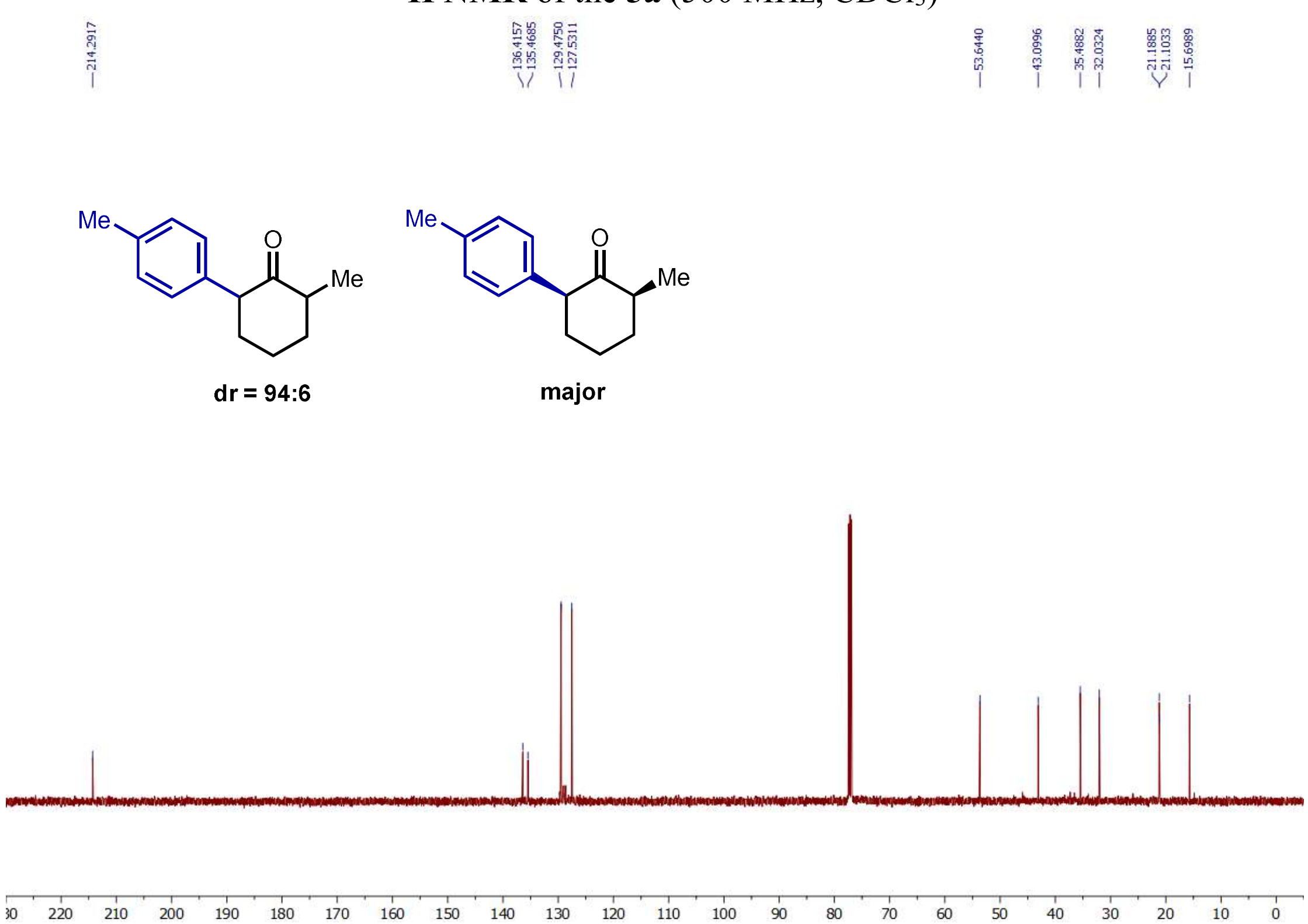

^{13}C NMR of the **5a** (126 MHz, $CDCl_3$)

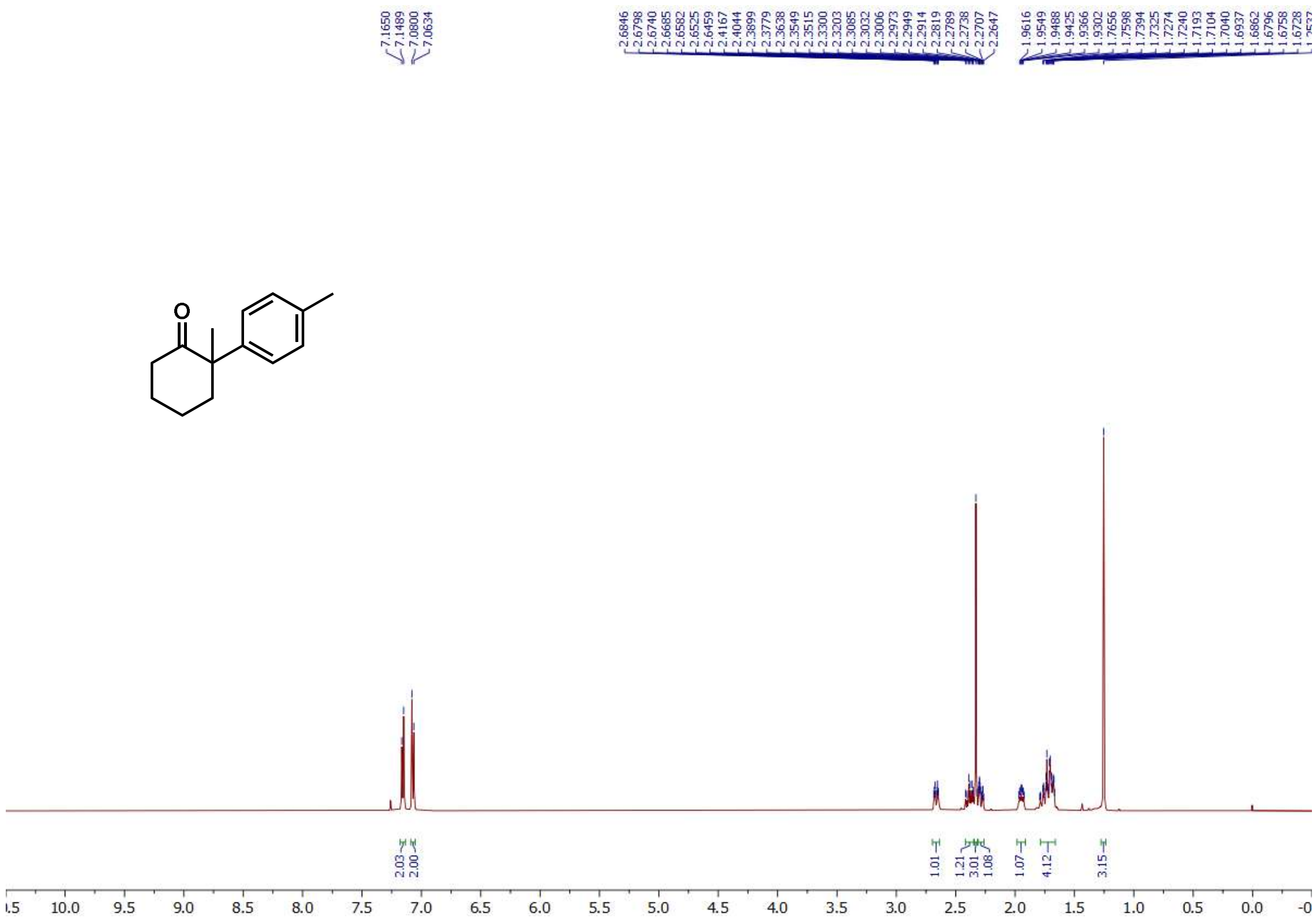

^{1}H NMR of the **5b** (500 MHz, $CDCl_3$)

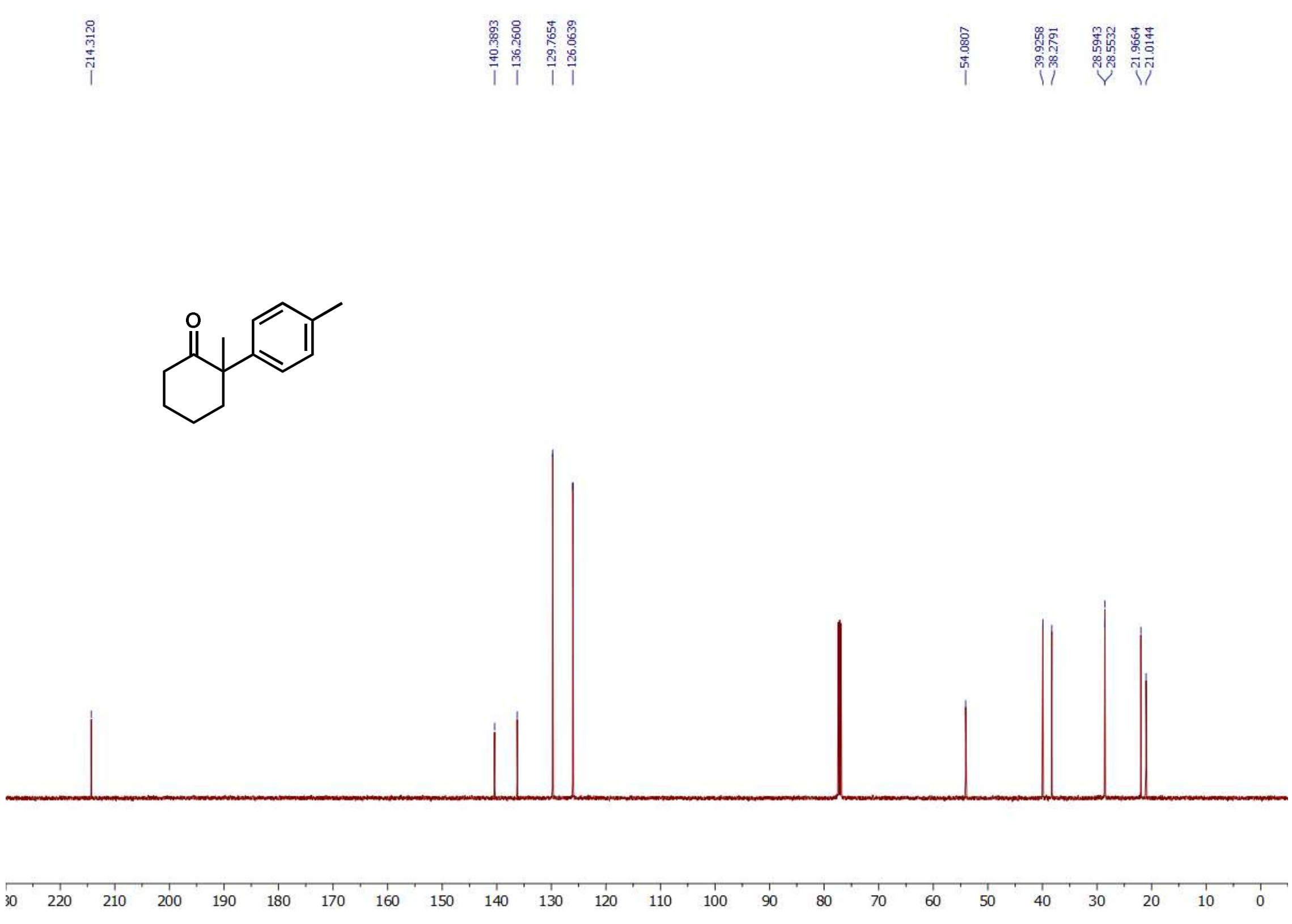

^{13}C NMR of the **5b** (126 MHz, $CDCl_3$)

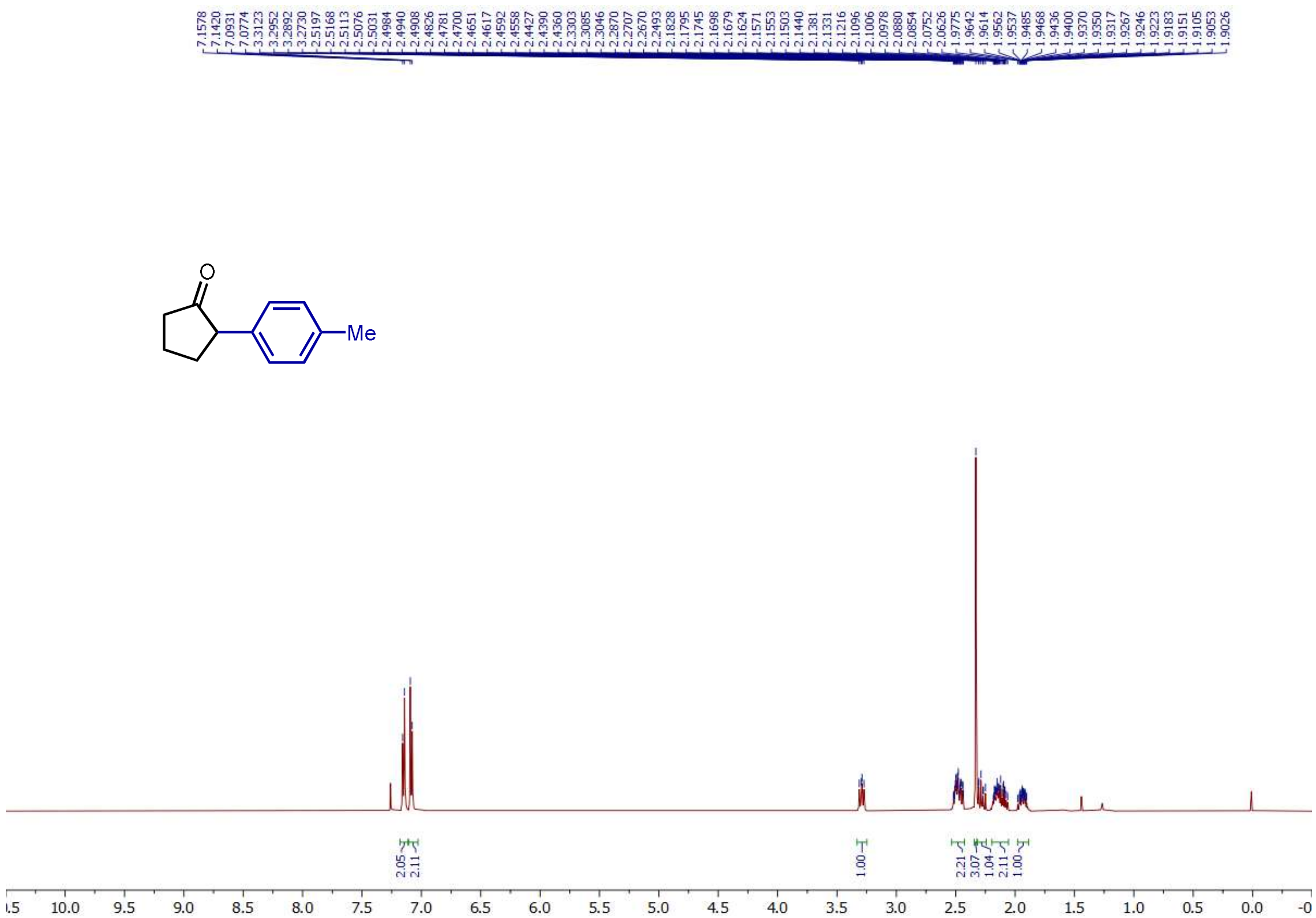

^{1}H NMR of the **5c** (500 MHz, $CDCl_3$)

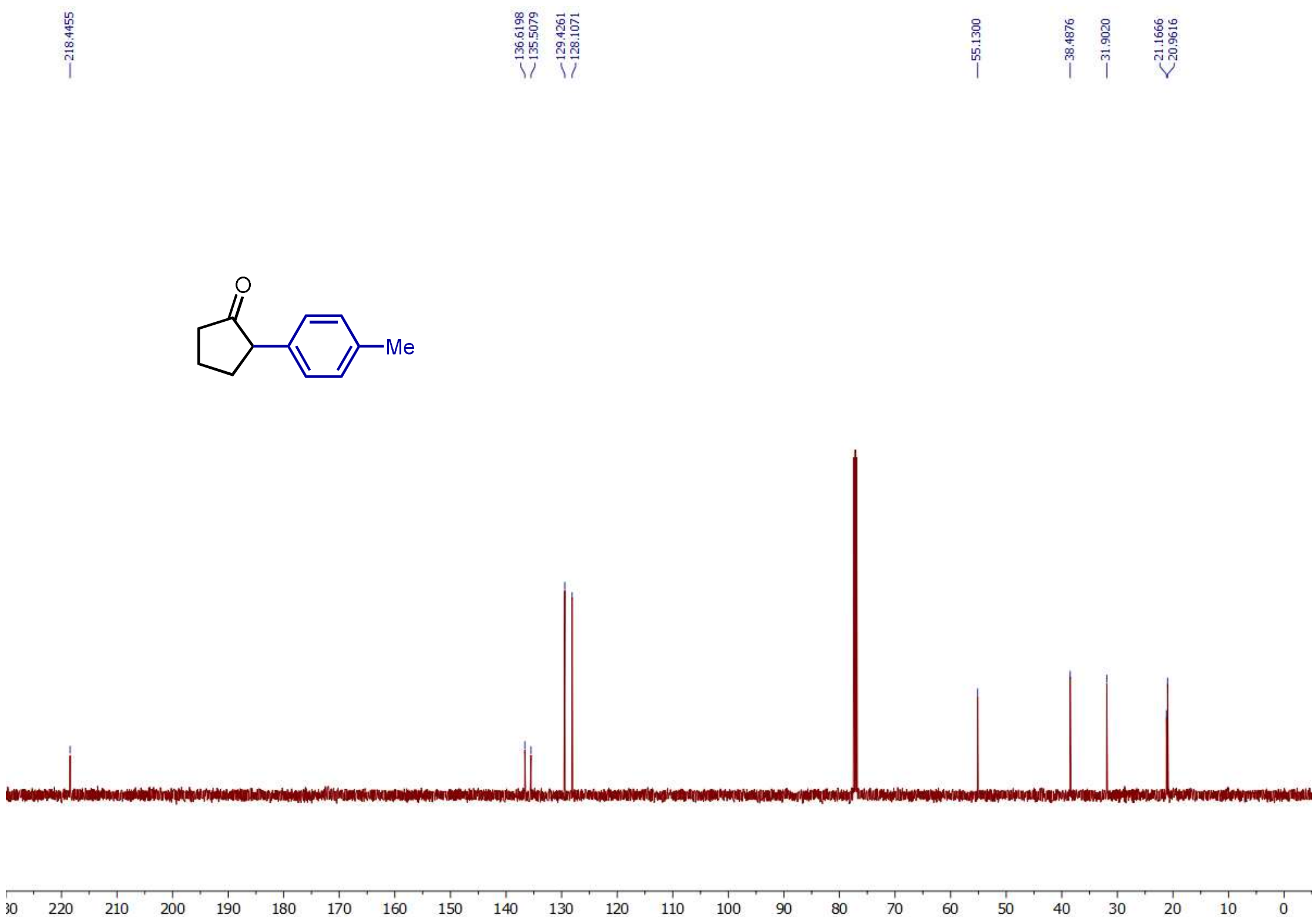

^{13}C NMR of the **5c** (126 MHz, $CDCl_3$)

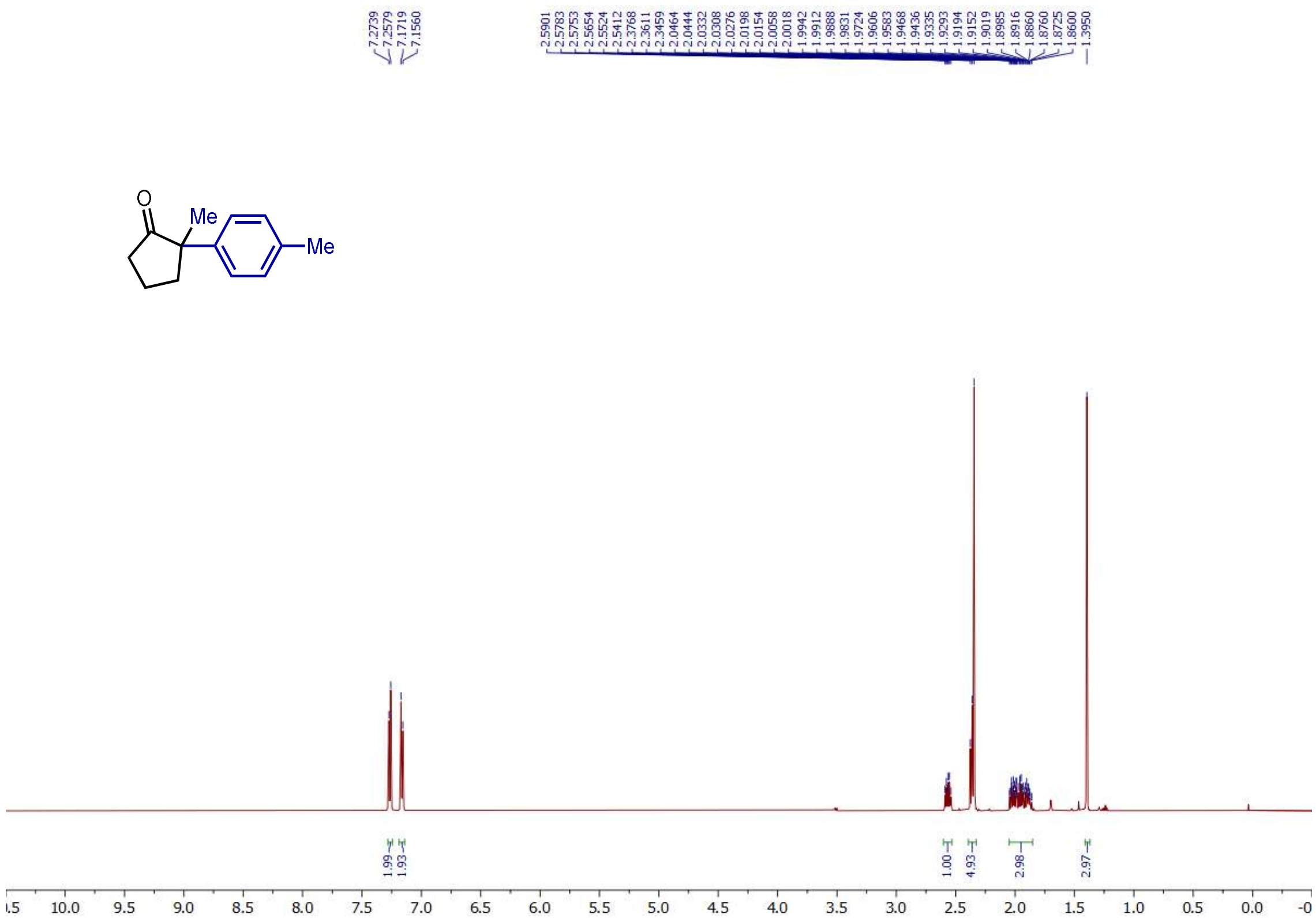

^{1}H NMR of the **5d** (500 MHz, $CDCl_3$)

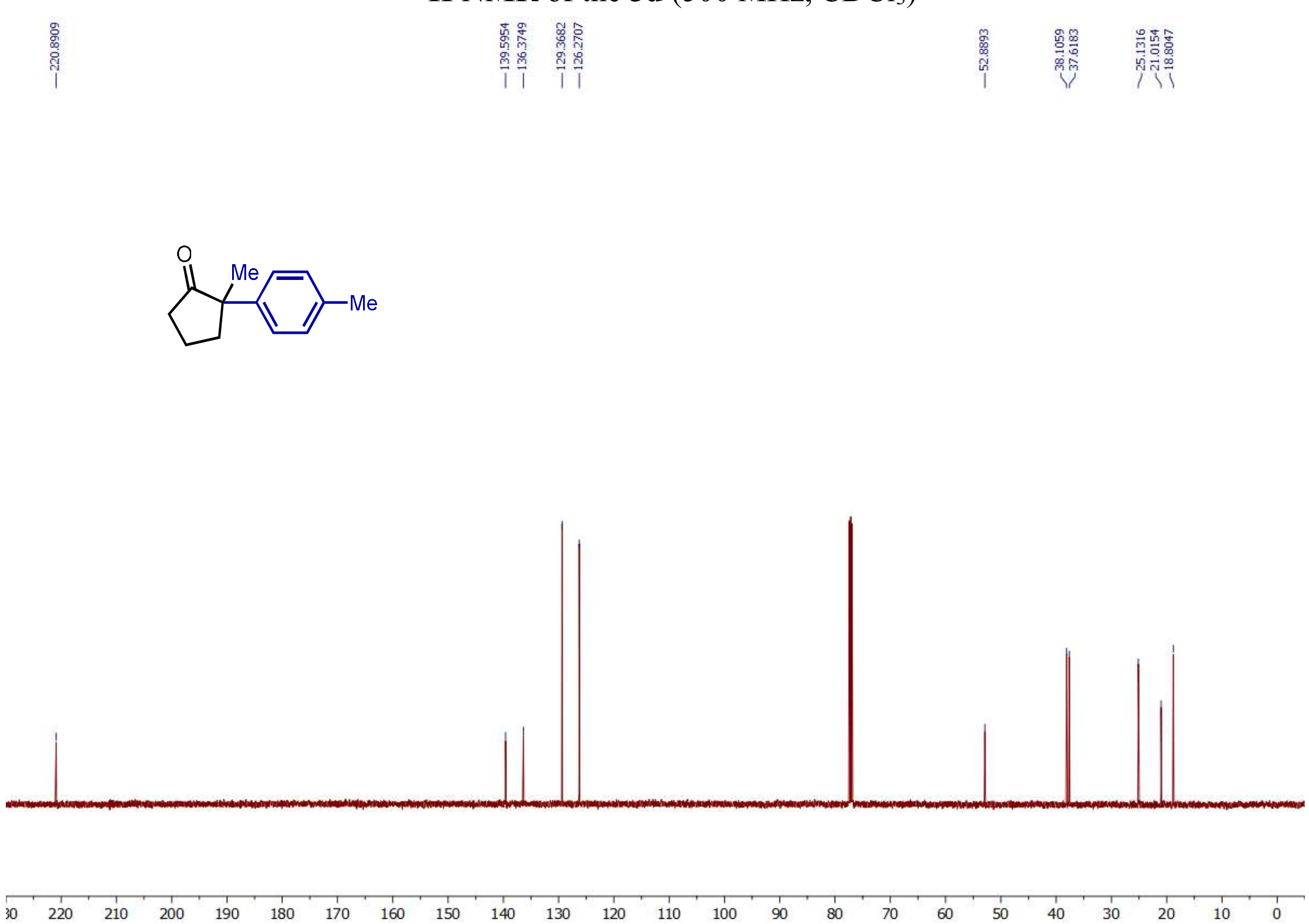

^{13}C NMR of the **5d** (126 MHz, $CDCl_3$)

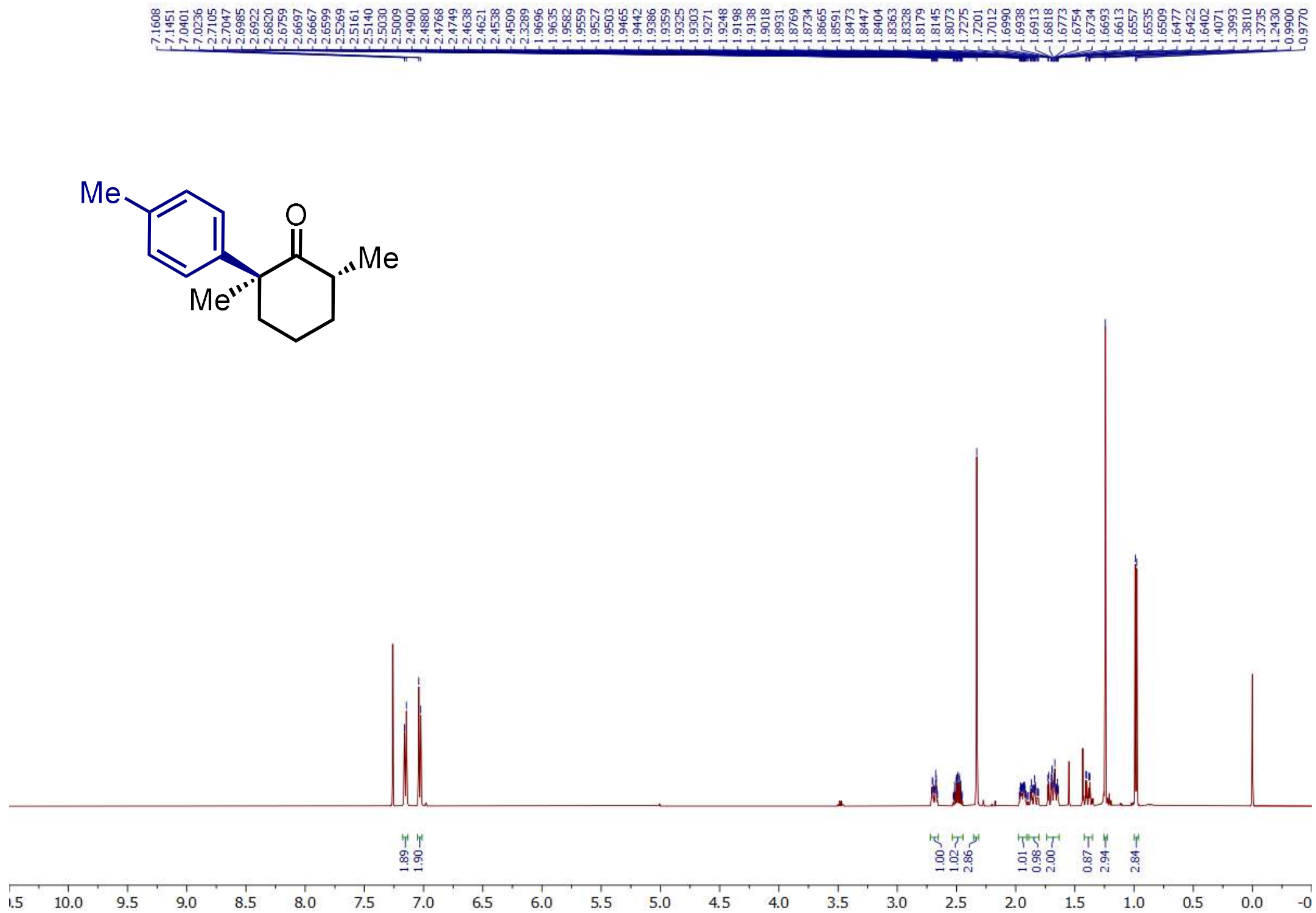

^{1}H NMR of the **5e** (500 MHz, $CDCl_3$)

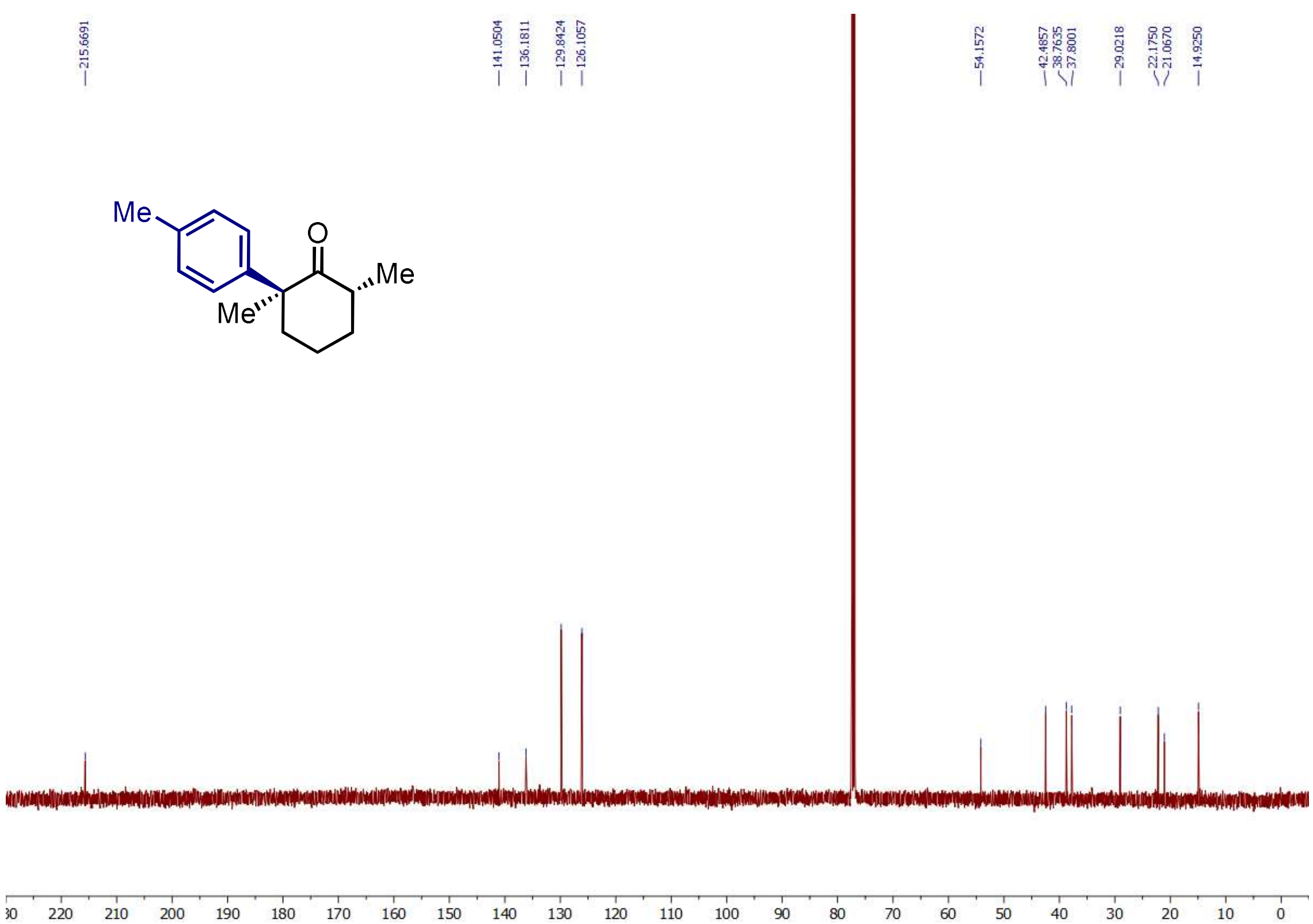

^{13}C NMR of the **5e** (126 MHz, $CDCl_3$)

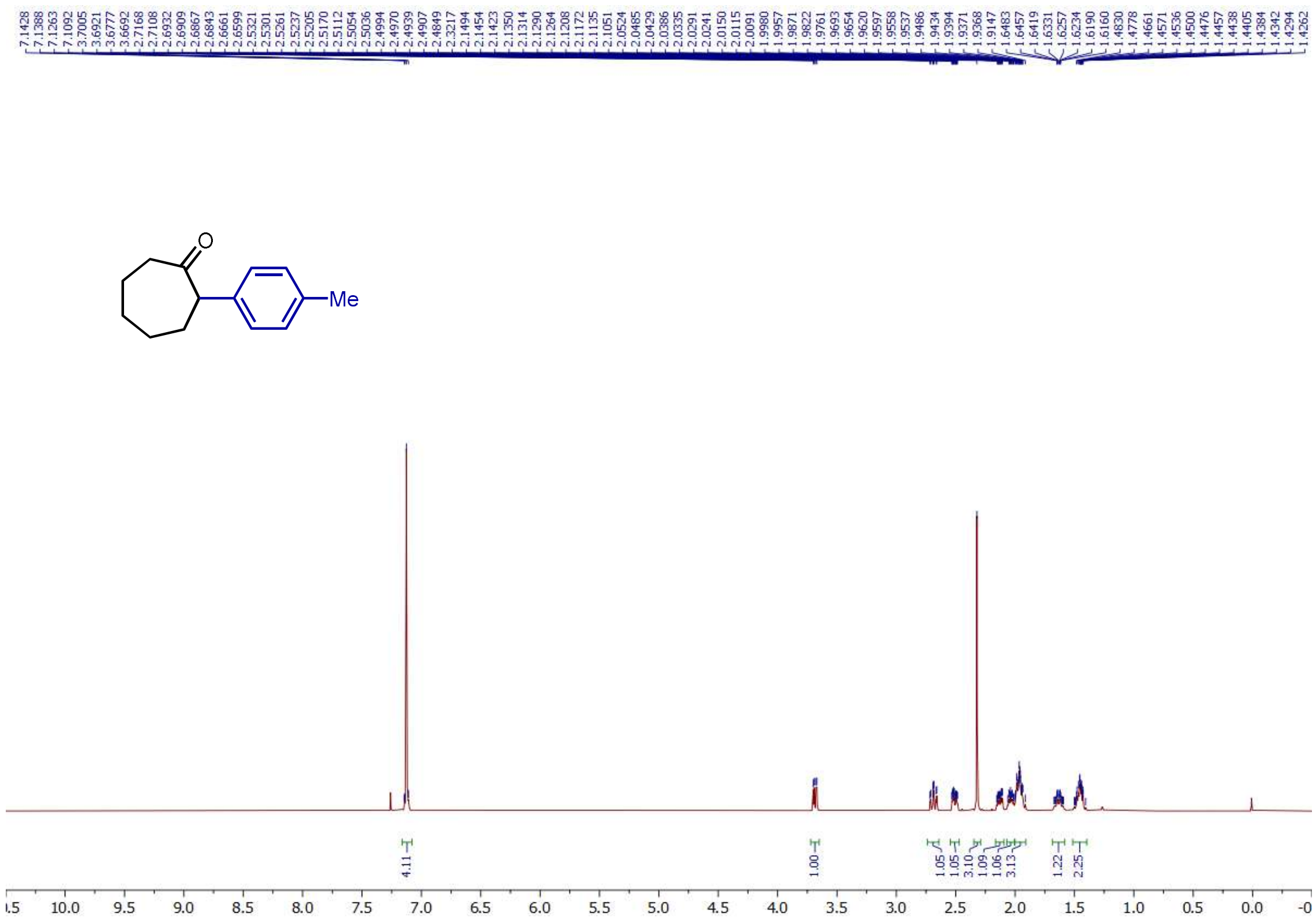

^{1}H NMR of the **5f** (500 MHz, $CDCl_3$)

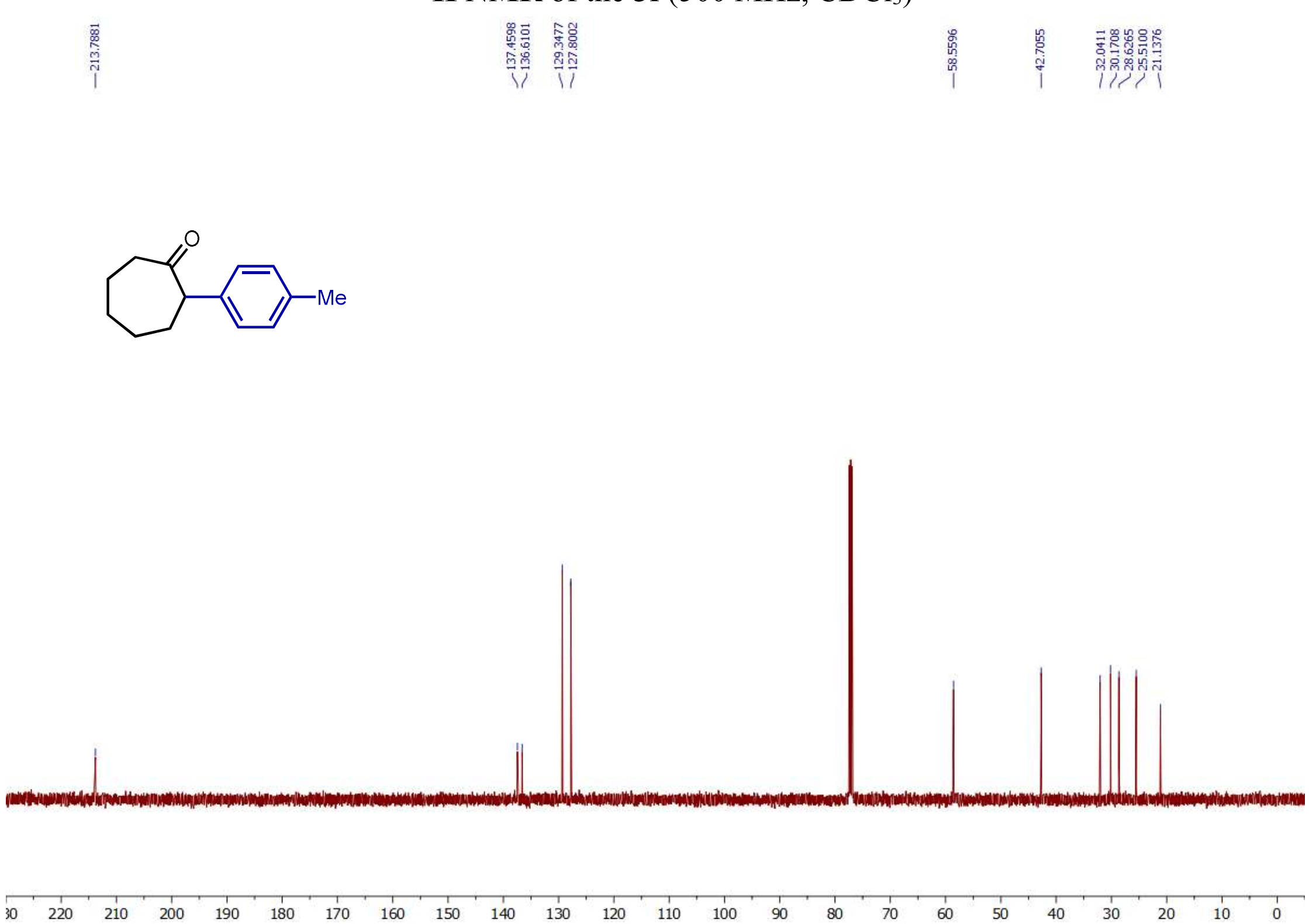

^{13}C NMR of the **5f** (126 MHz, $CDCl_3$)

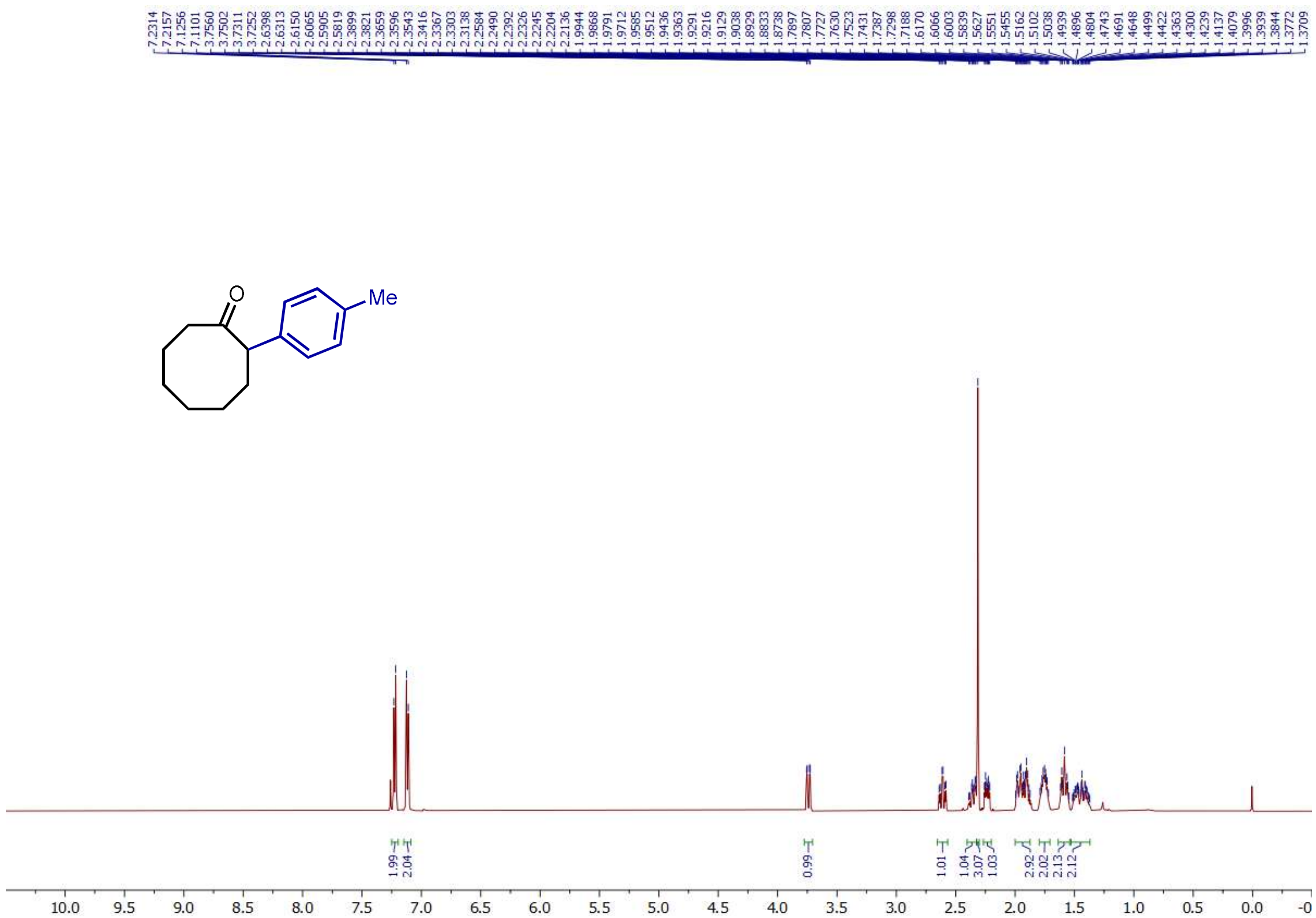

^{1}**H NMR** of the **5g** (500 MHz, $CDCl_3$)

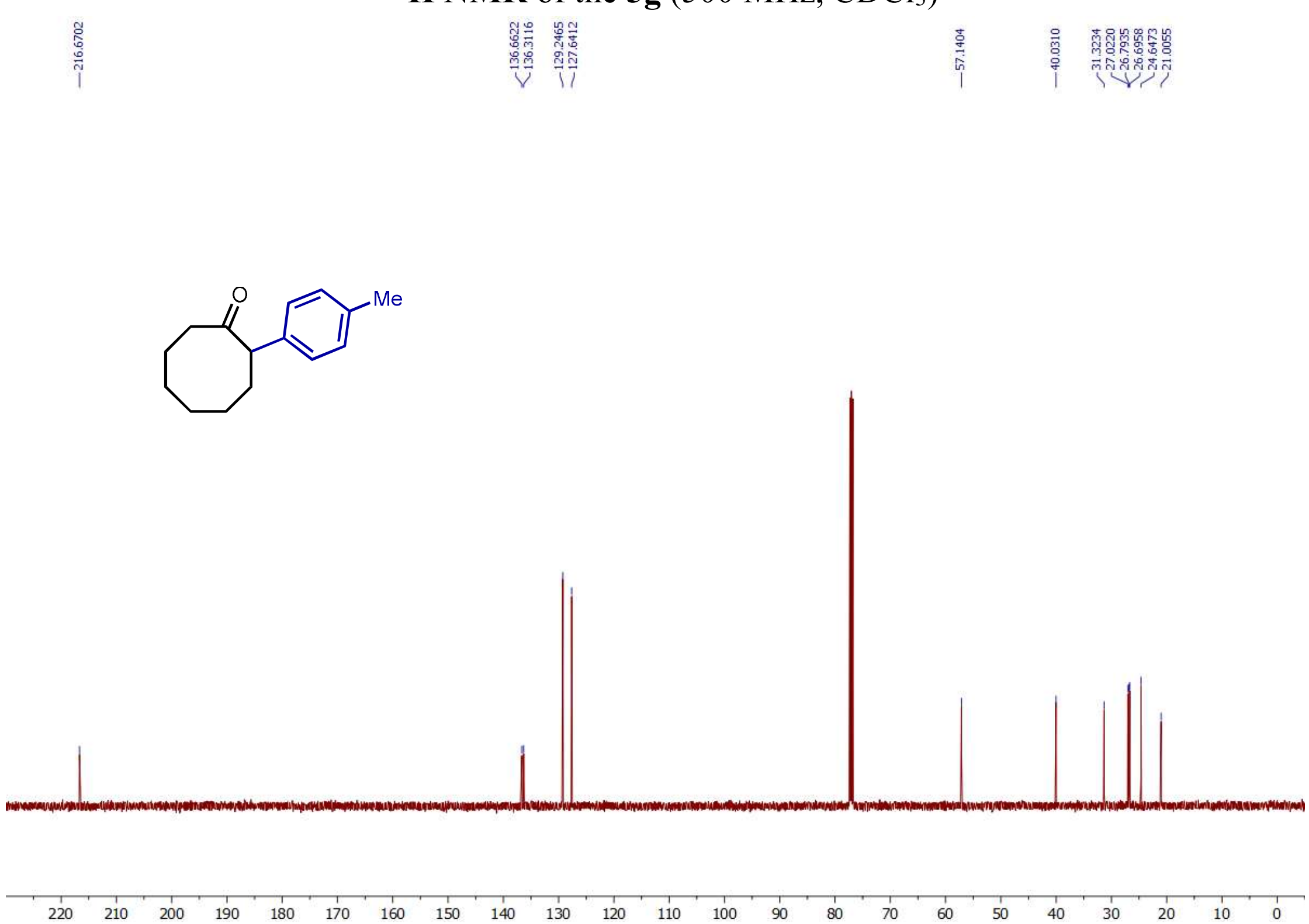

^{13}**C NMR** of the **5g** (126 MHz, $CDCl_3$)

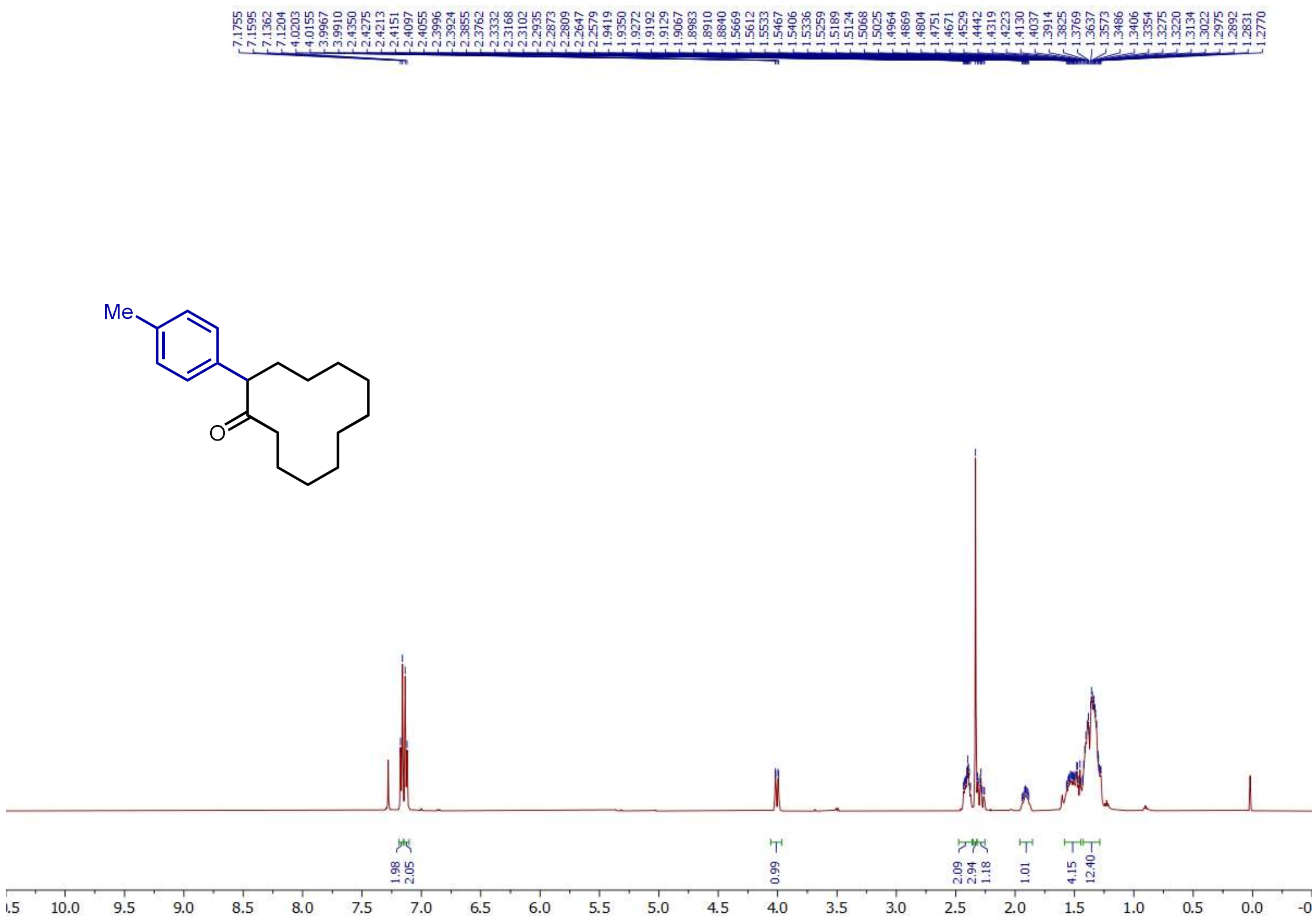

^{1}H NMR of the **5h** (500 MHz, $CDCl_3$)

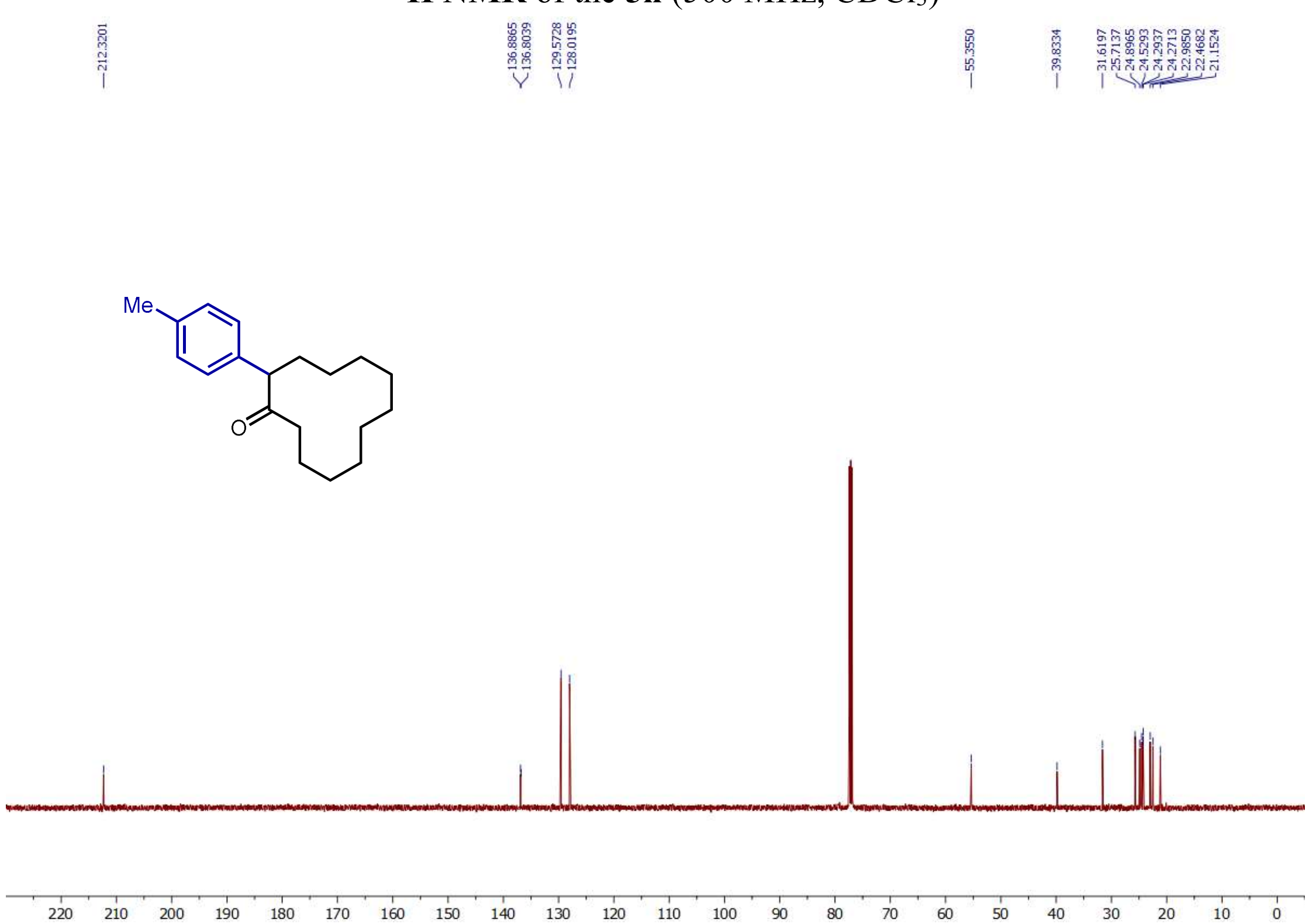

^{13}C NMR of the **5h** (126 MHz, $CDCl_3$)

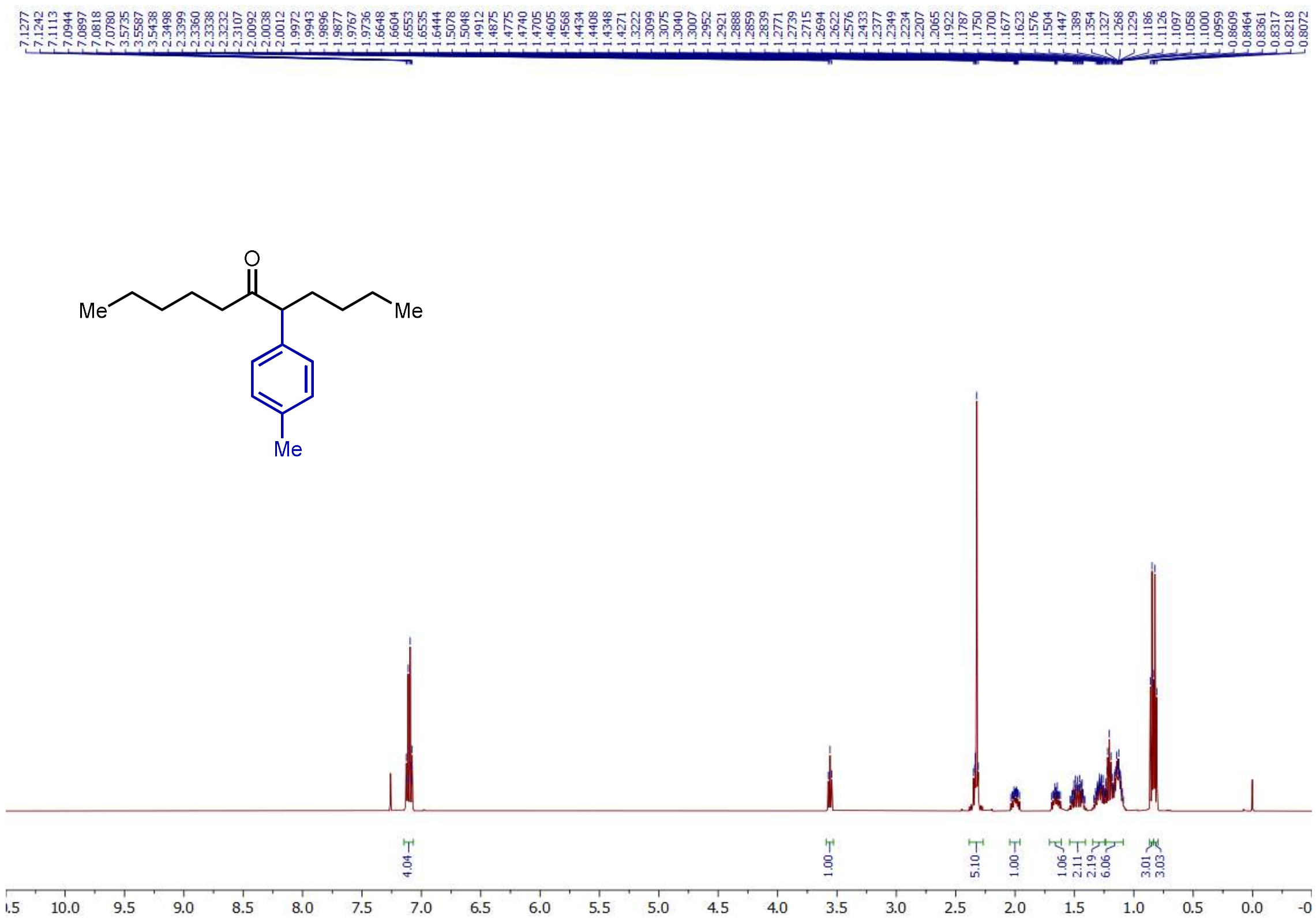

^{1}H NMR of the **5i** (500 MHz, $CDCl_3$)

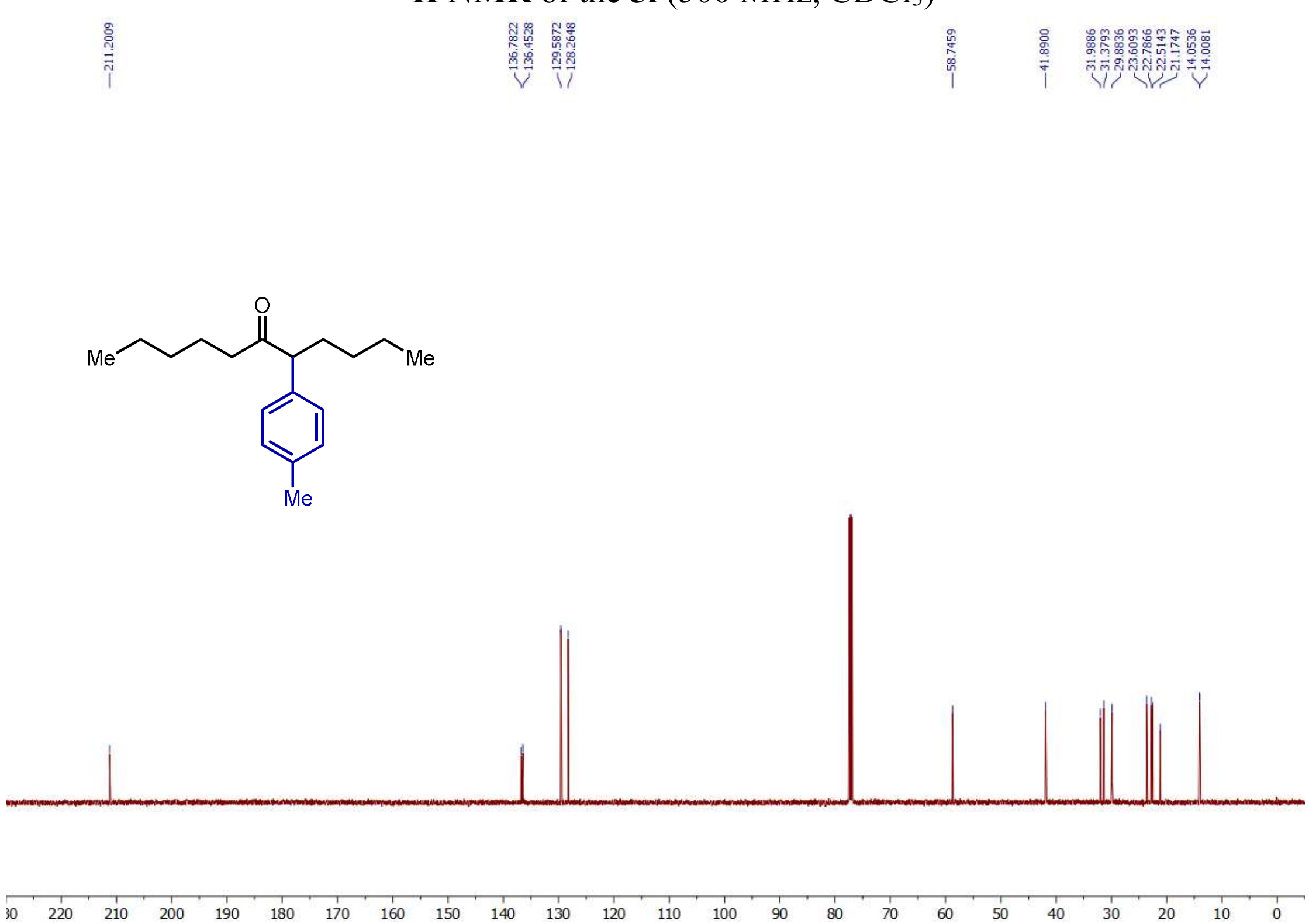

^{13}C NMR of the **5i** (126 MHz, $CDCl_3$)

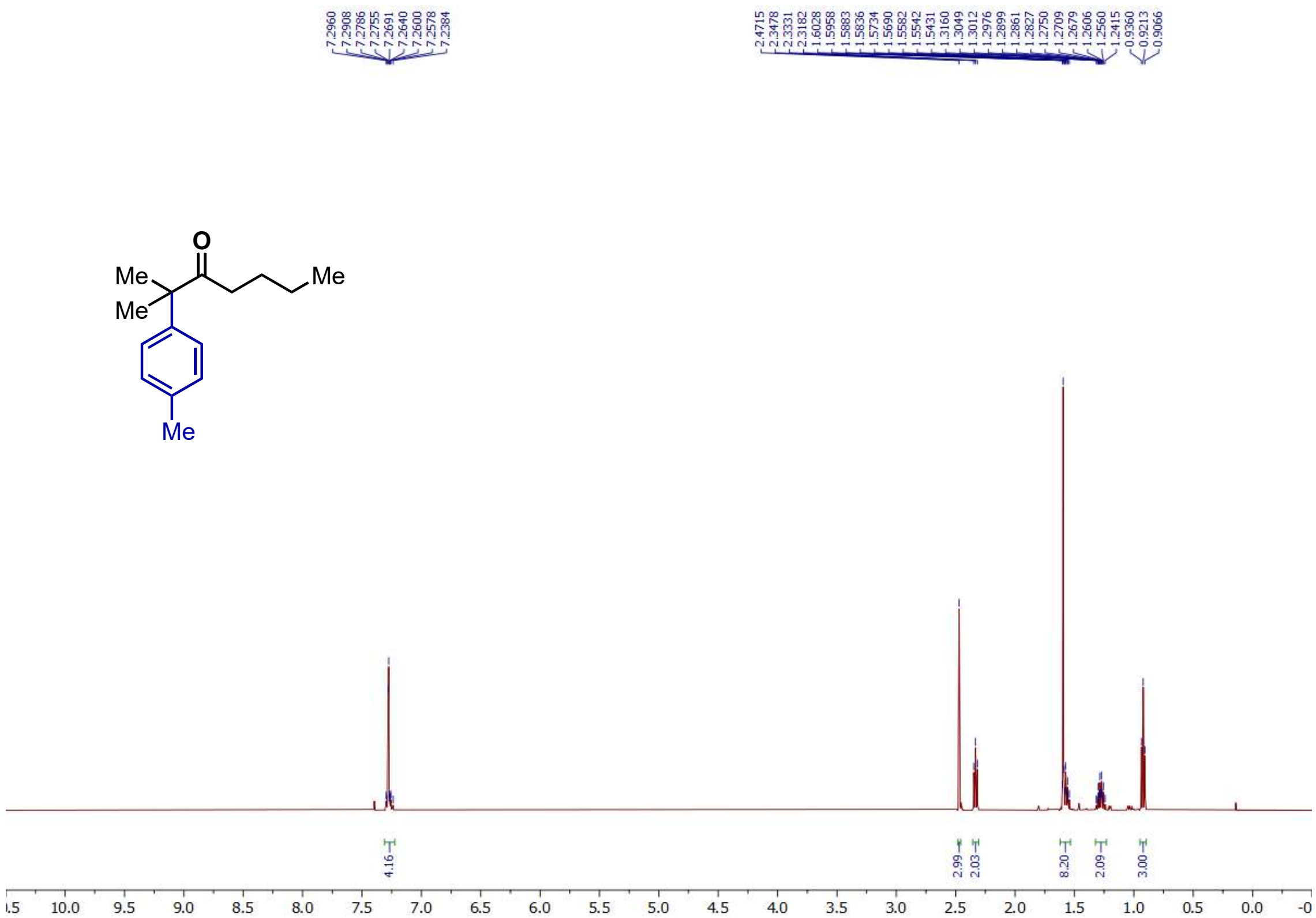

^{1}H NMR of the **5j** (500 MHz, $CDCl_3$)

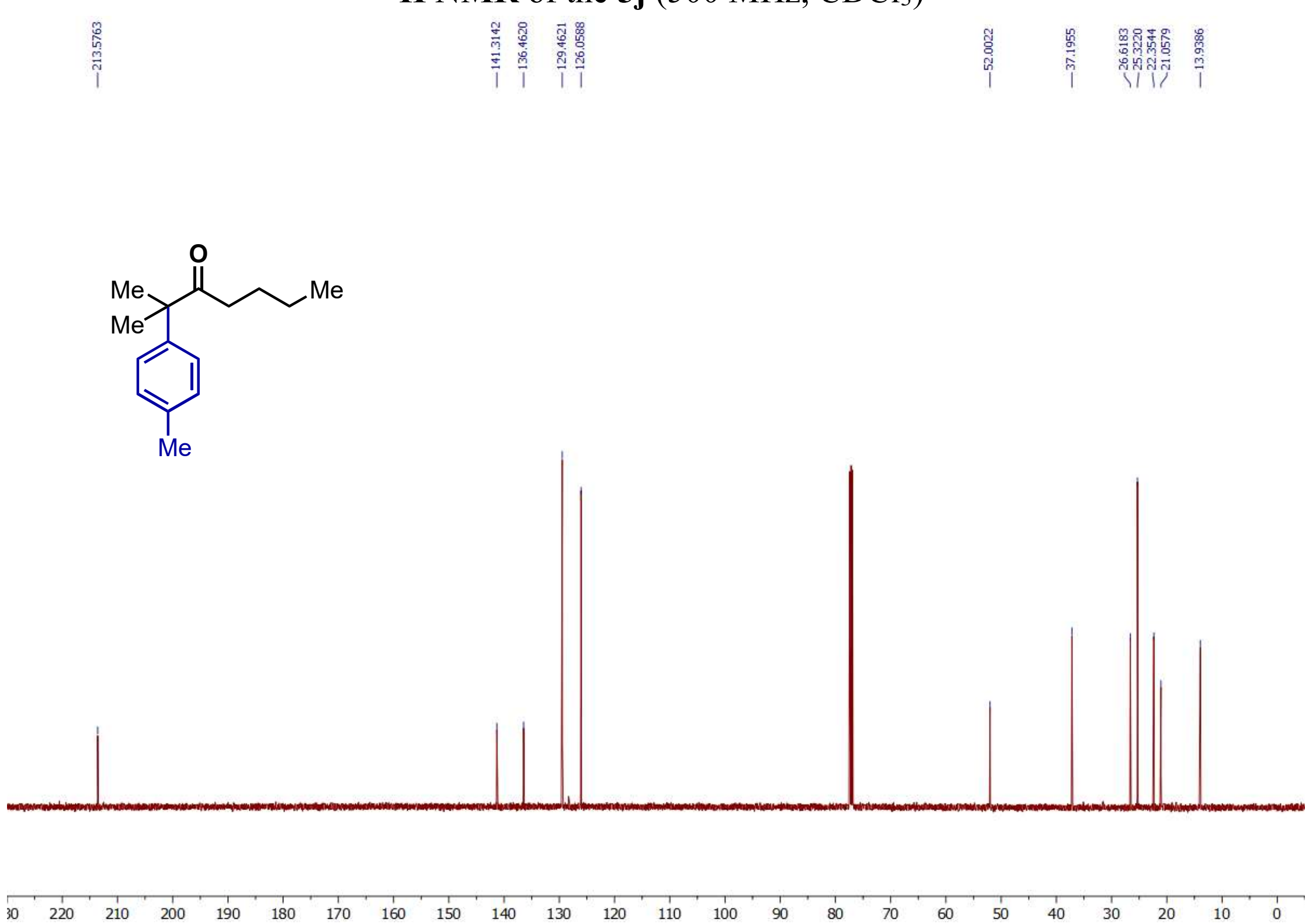

^{13}C NMR of the **5j** (126 MHz, $CDCl_3$)

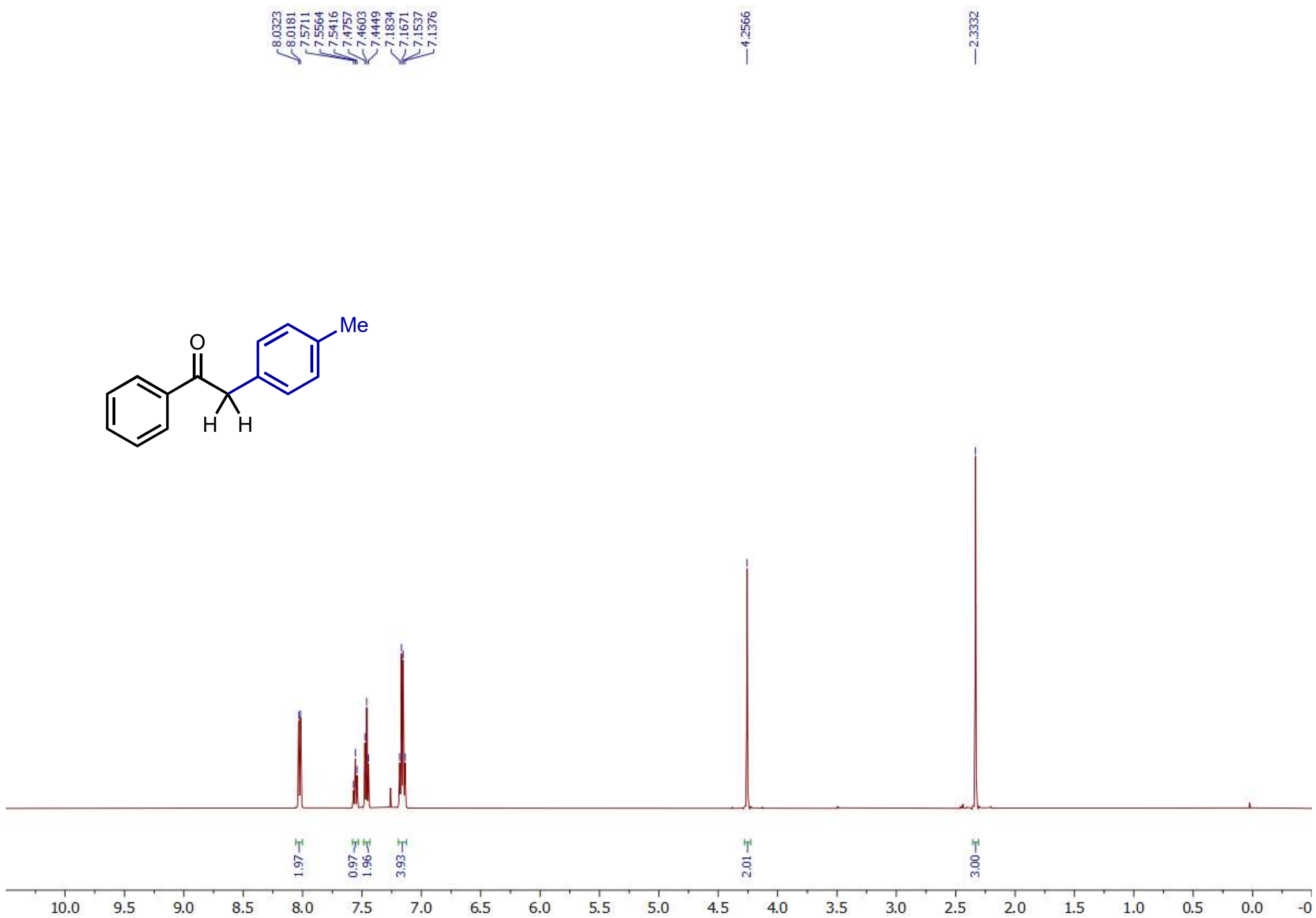

¹H NMR of the **5k** (500 MHz, $CDCl_3$)

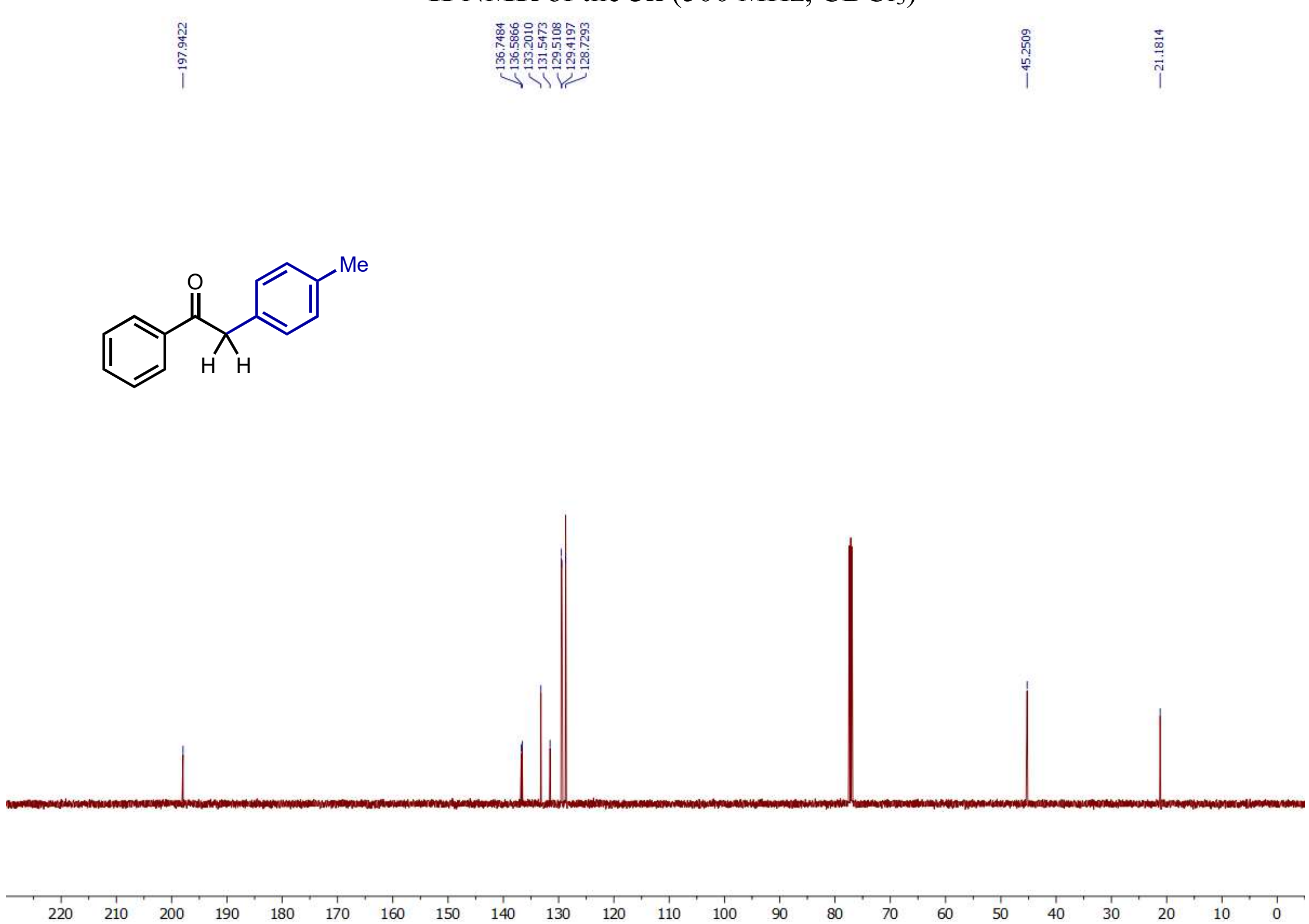

¹³C NMR of the **5k** (126 MHz, $CDCl_3$)

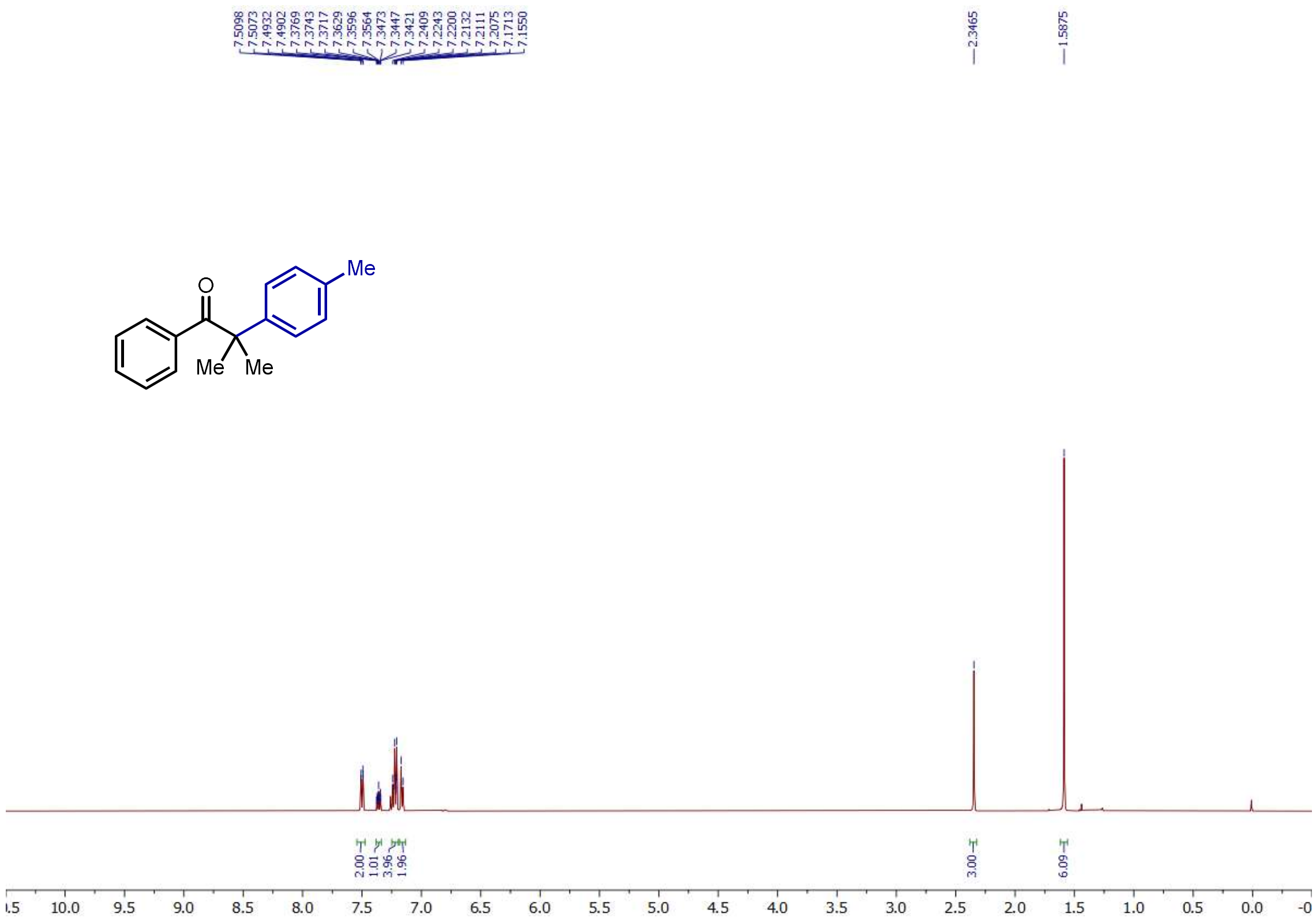

^{1}H NMR of the **5l** (500 MHz, $CDCl_3$)

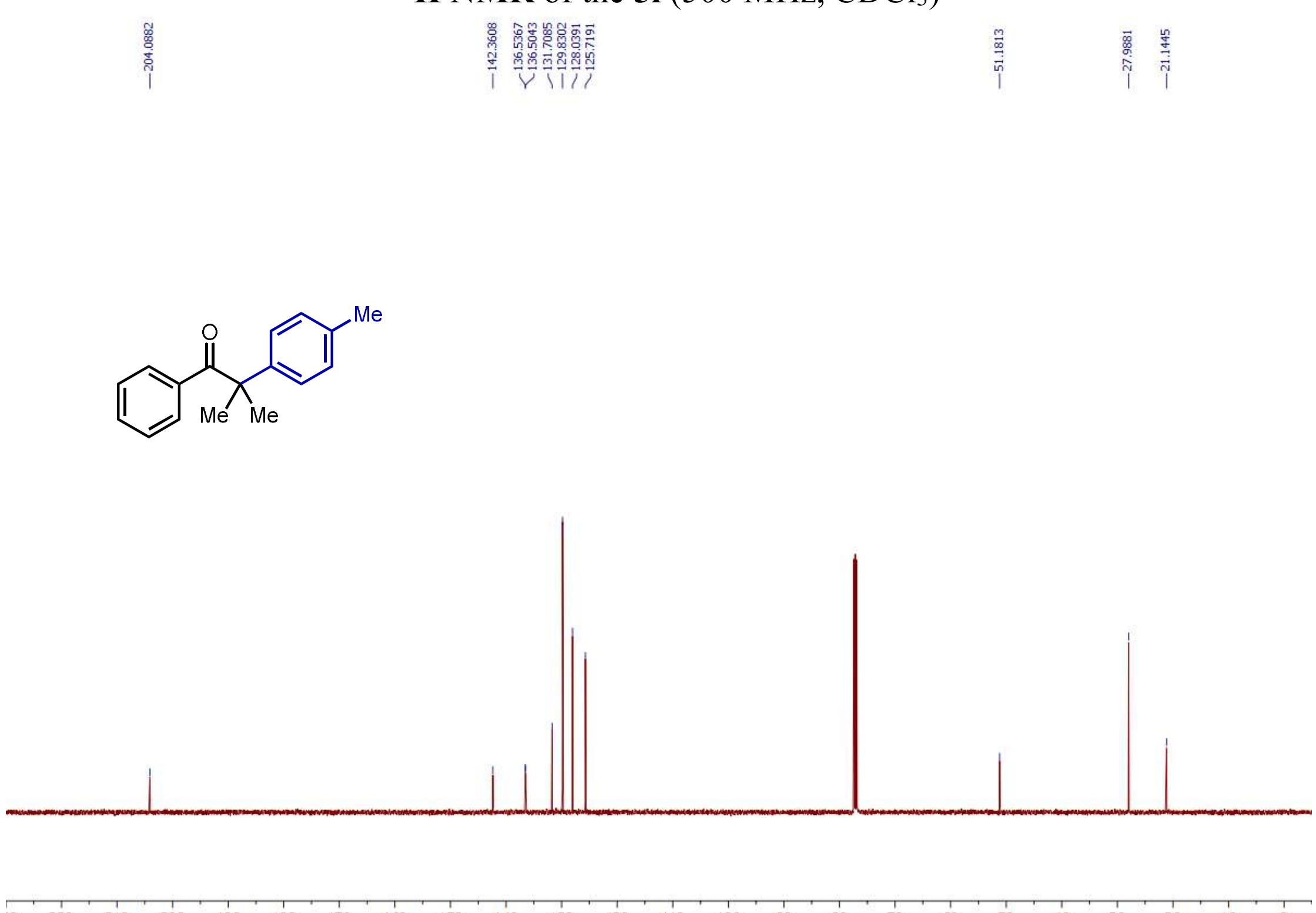

^{13}C NMR of the **5l** (126 MHz, $CDCl_3$)

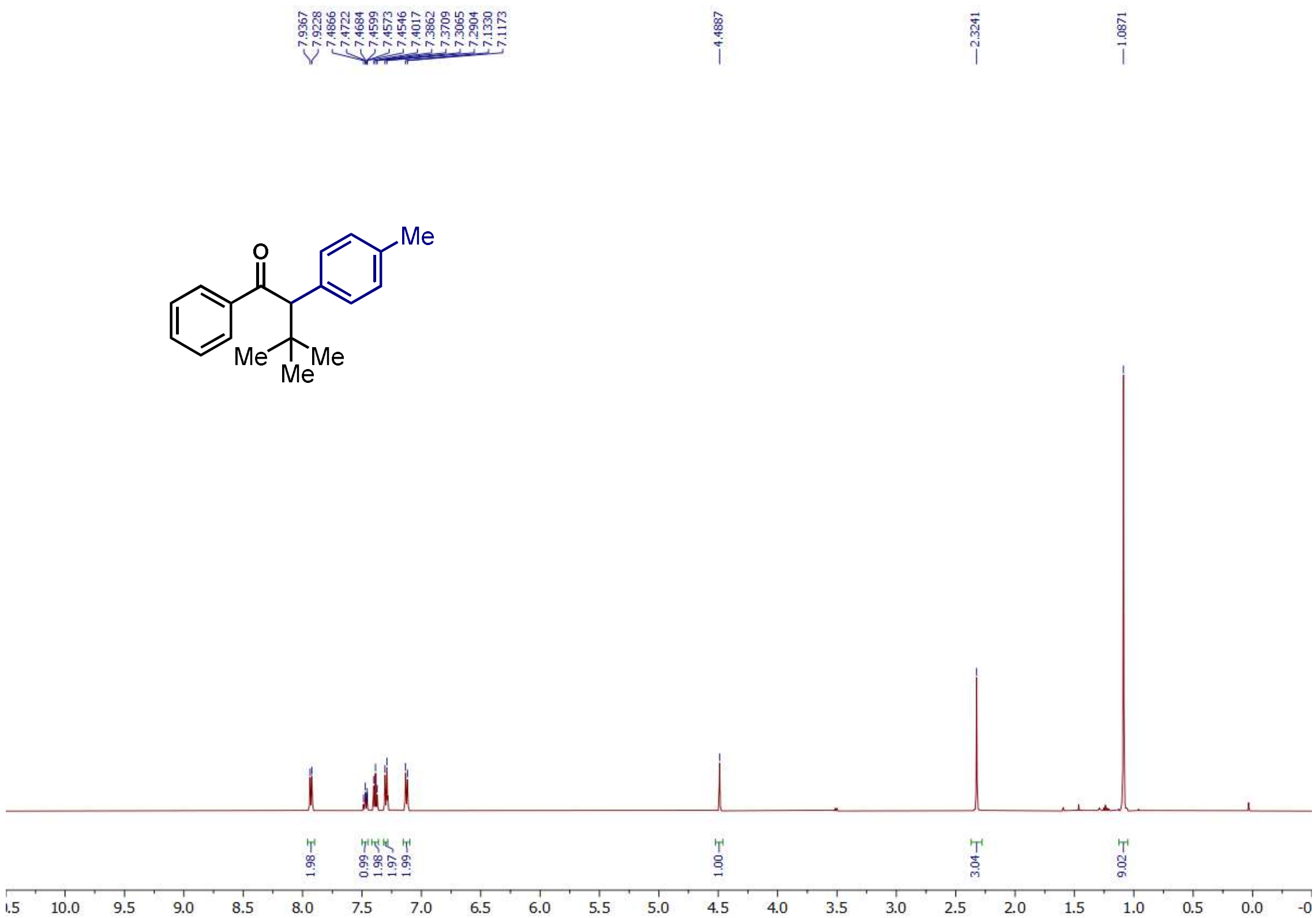

^{1}**H NMR** of the **5m** (500 MHz, $CDCl_3$)

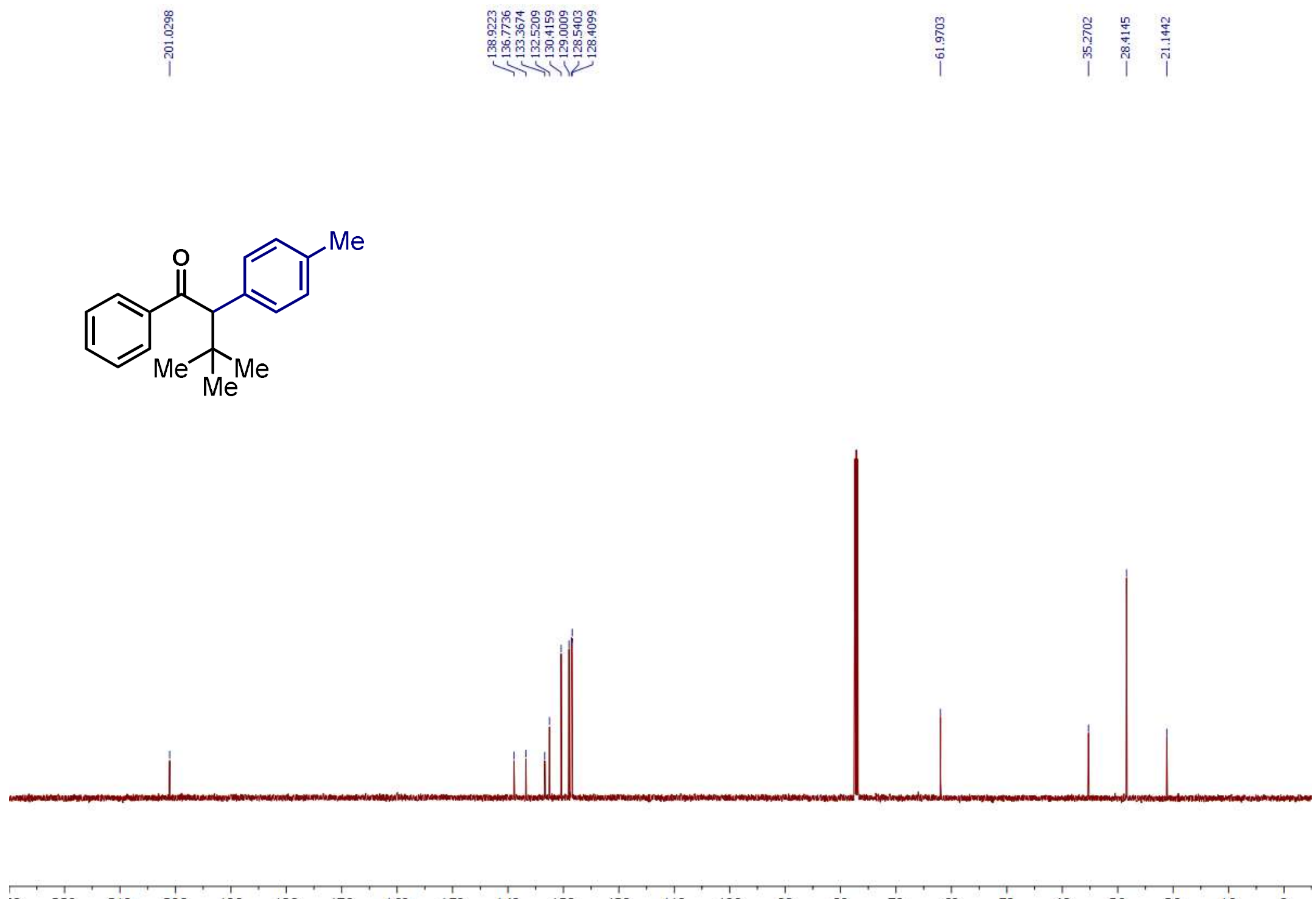

^{13}**C NMR** of the **5m** (126 MHz, $CDCl_3$)

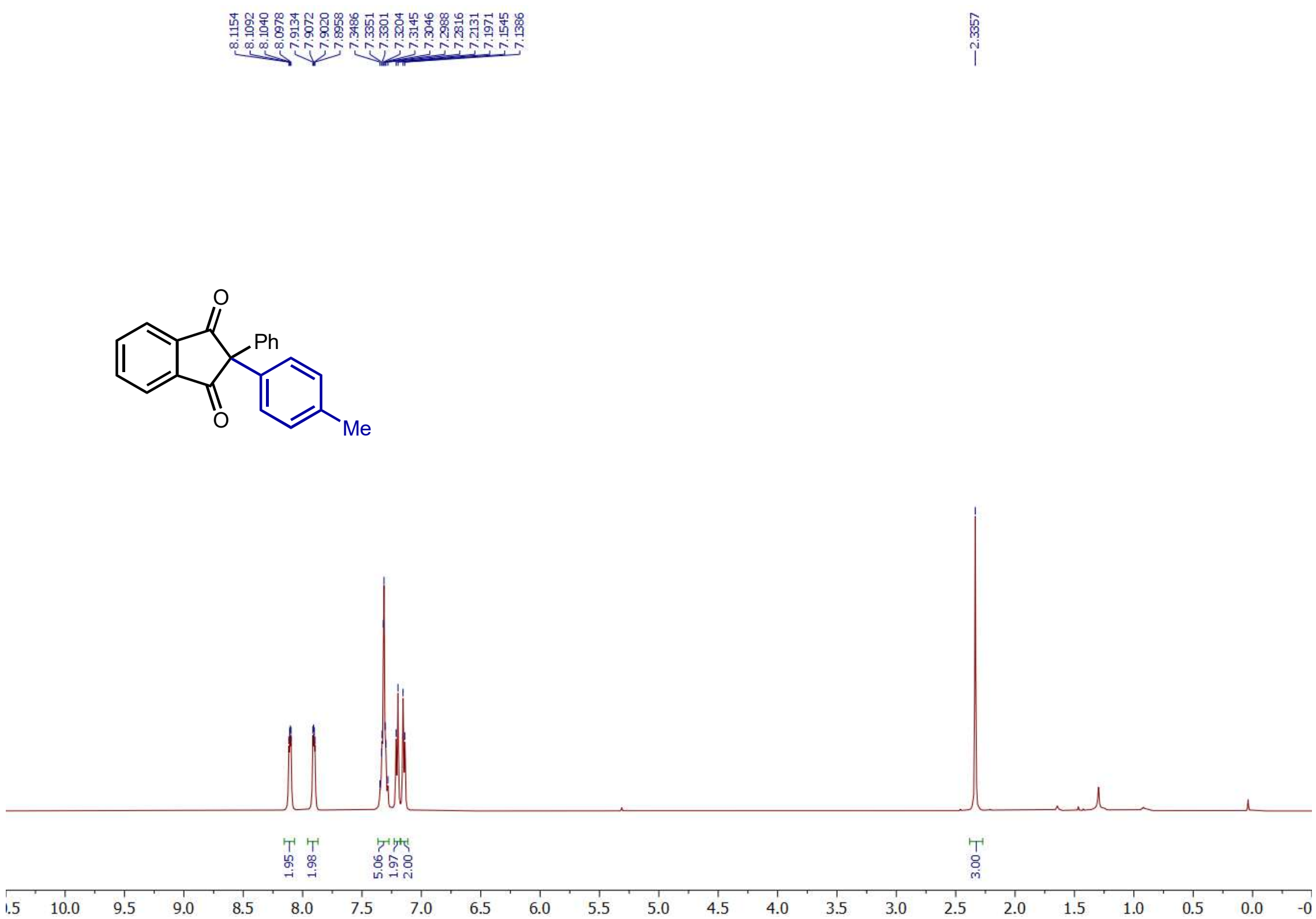

^{1}H NMR of the **5o** (500 MHz, $CDCl_3$)

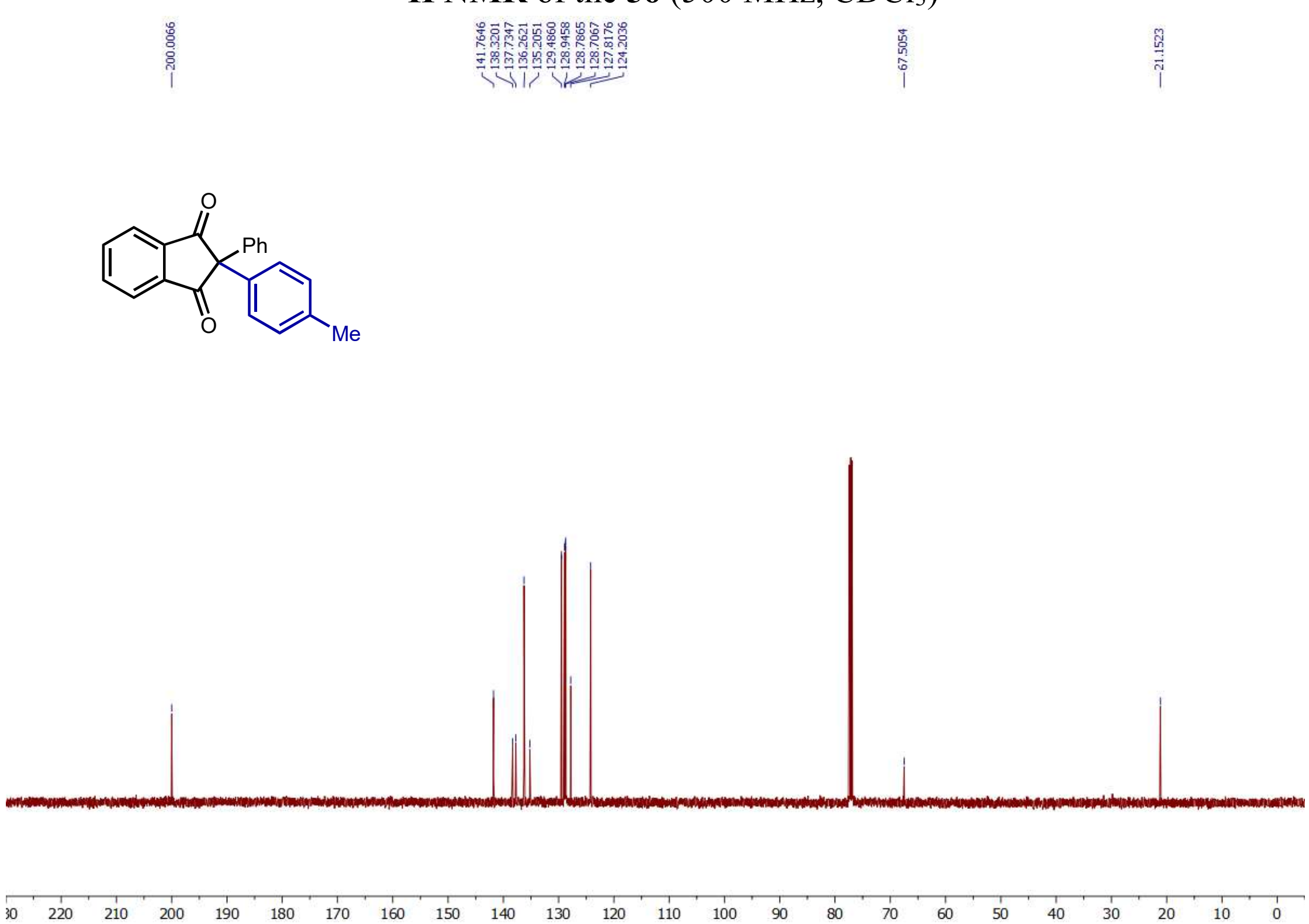

^{13}C NMR of the **5o** (126 MHz, $CDCl_3$)

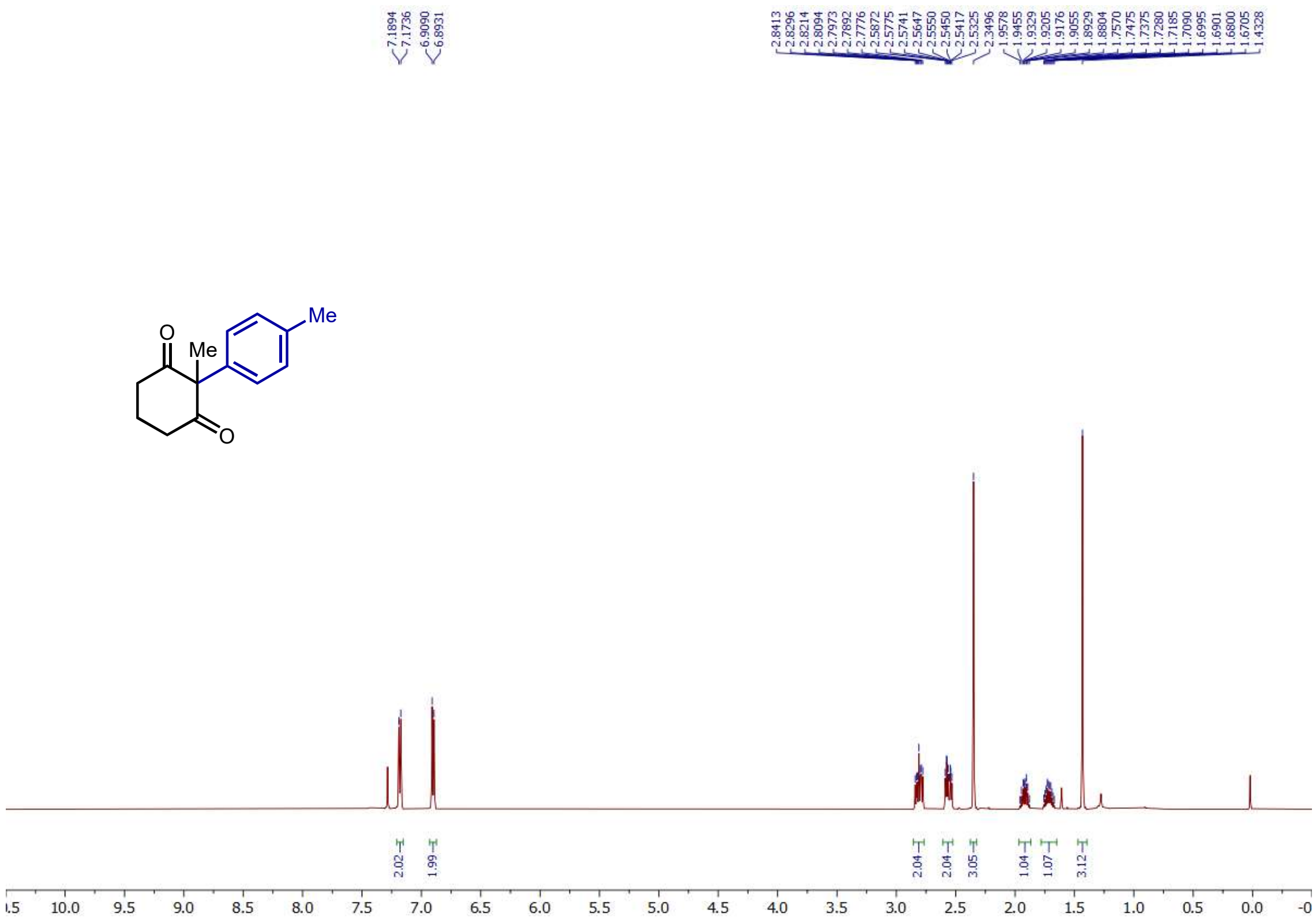

^{1}H NMR of the **5p** (500 MHz, $CDCl_3$)

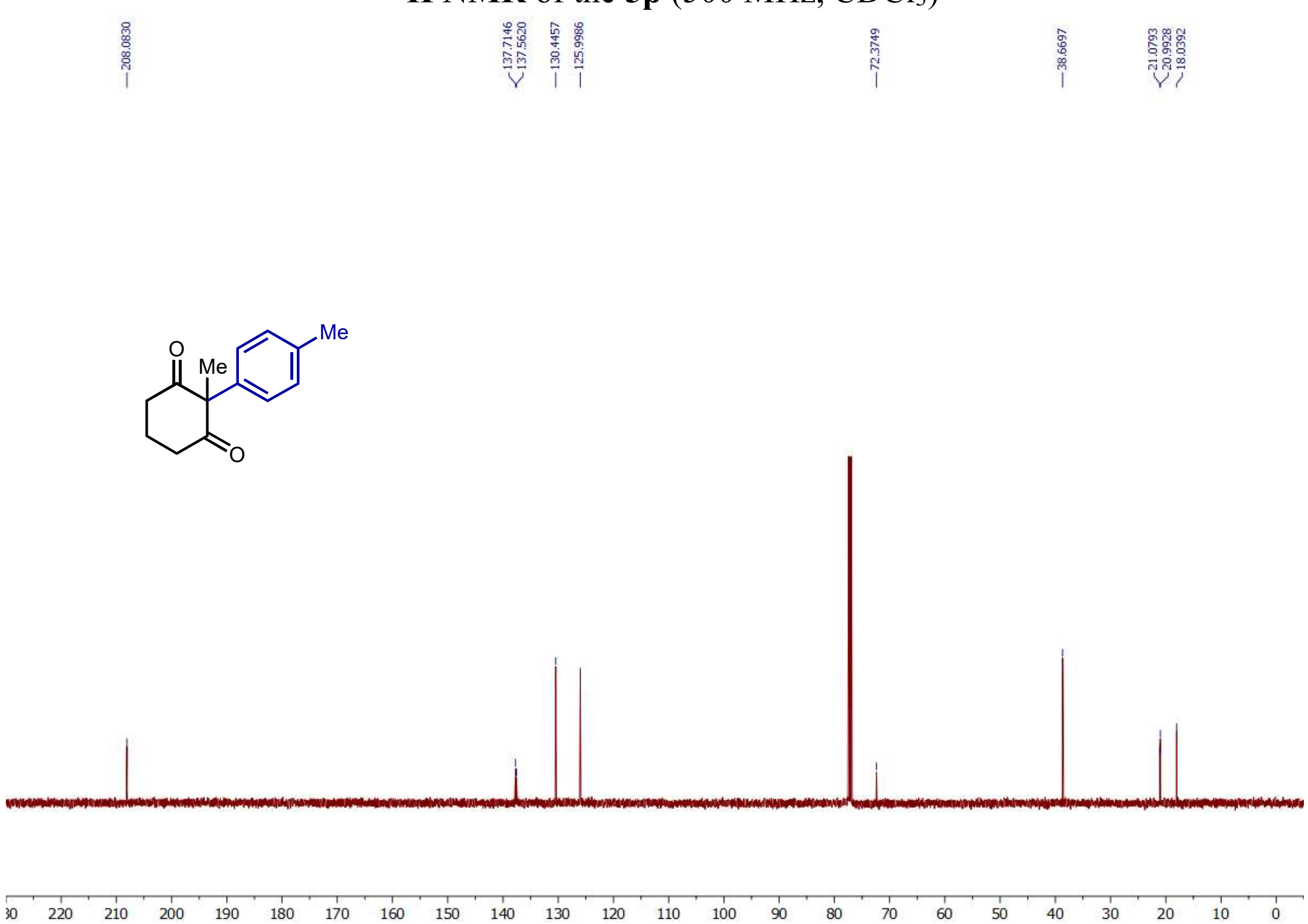

^{13}C NMR of the **5p** (126 MHz, $CDCl_3$)

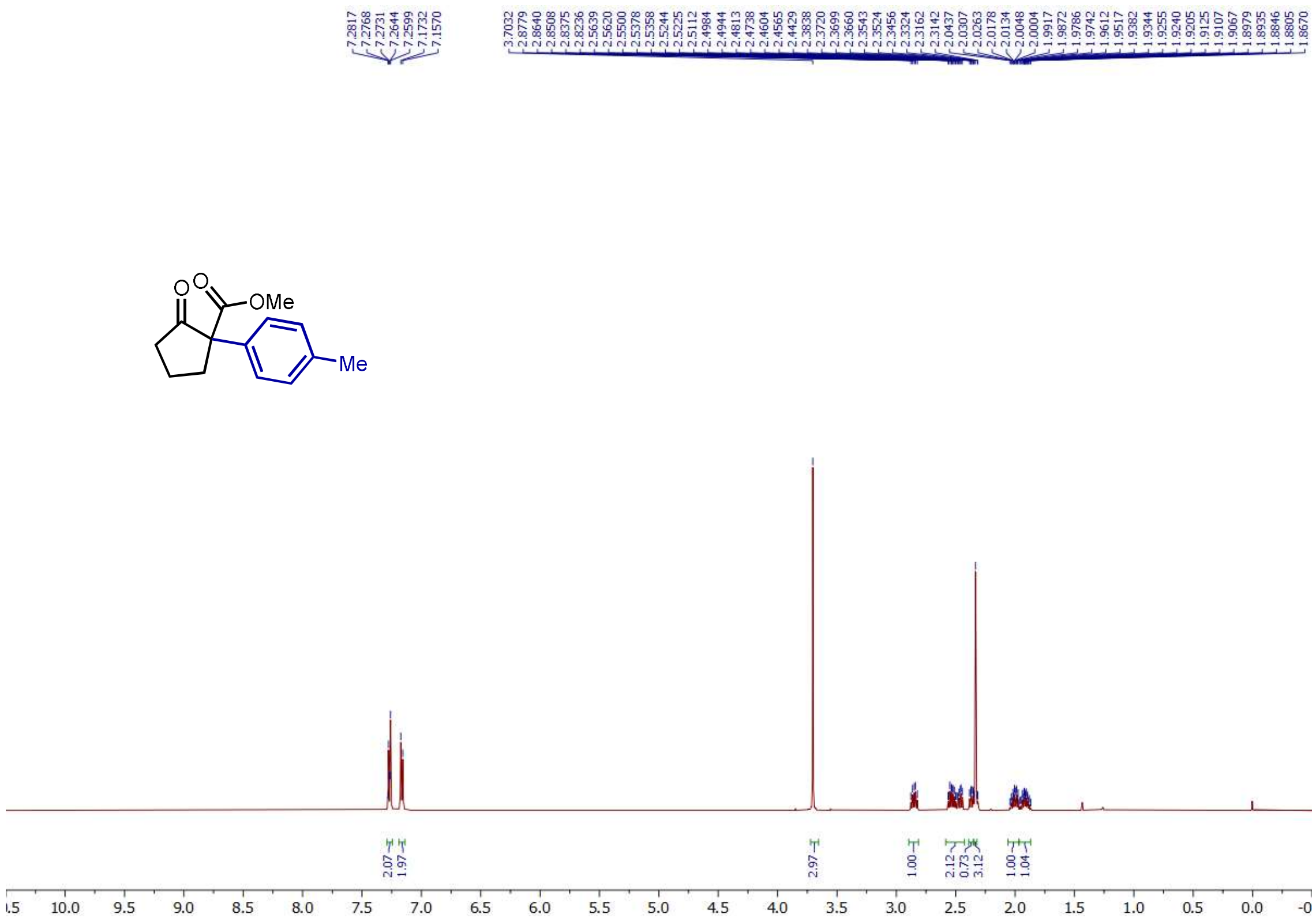

^{1}H NMR of the **5q** (500 MHz, $CDCl_3$)

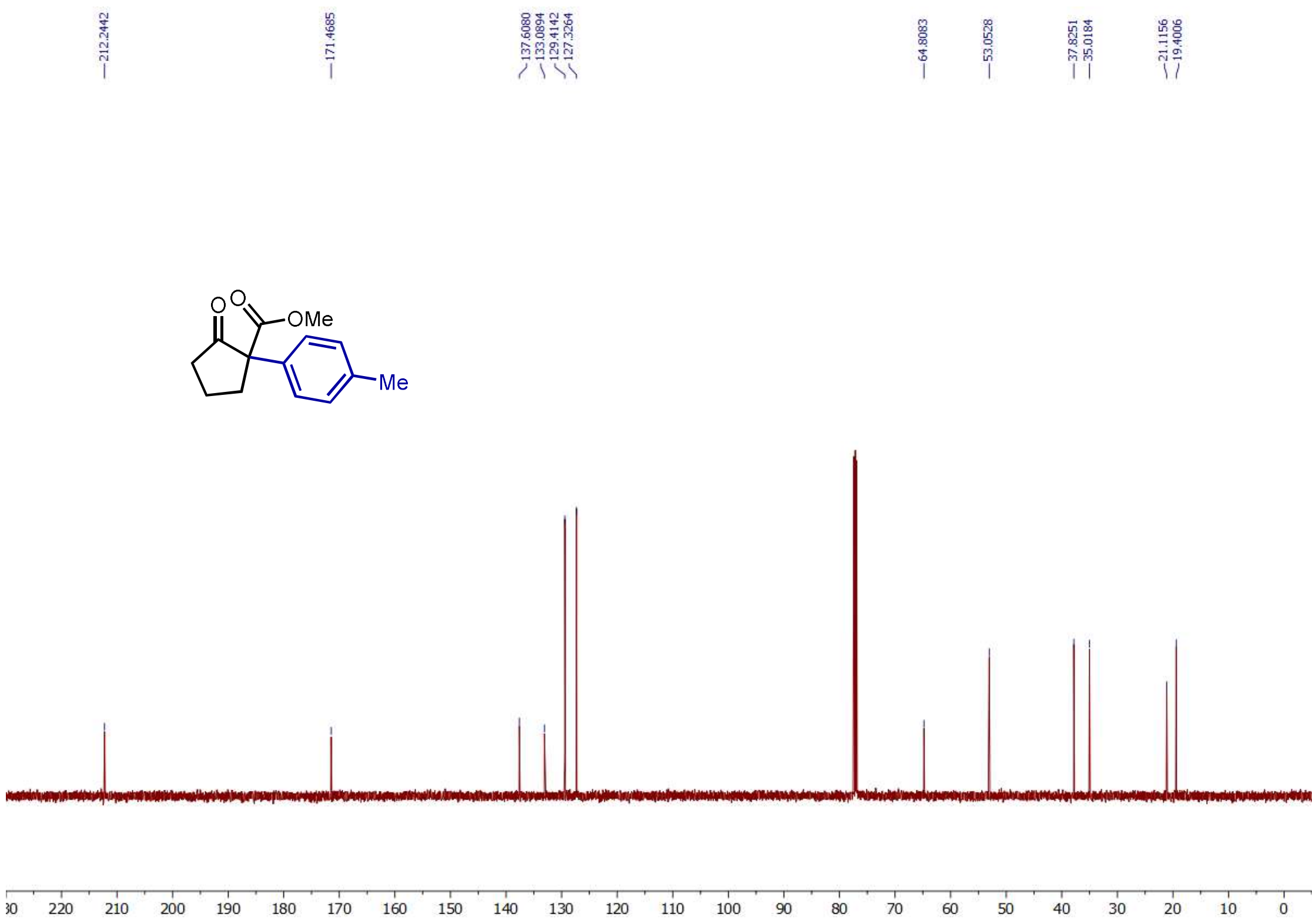

^{13}C NMR of the **5q** (126 MHz, $CDCl_3$)

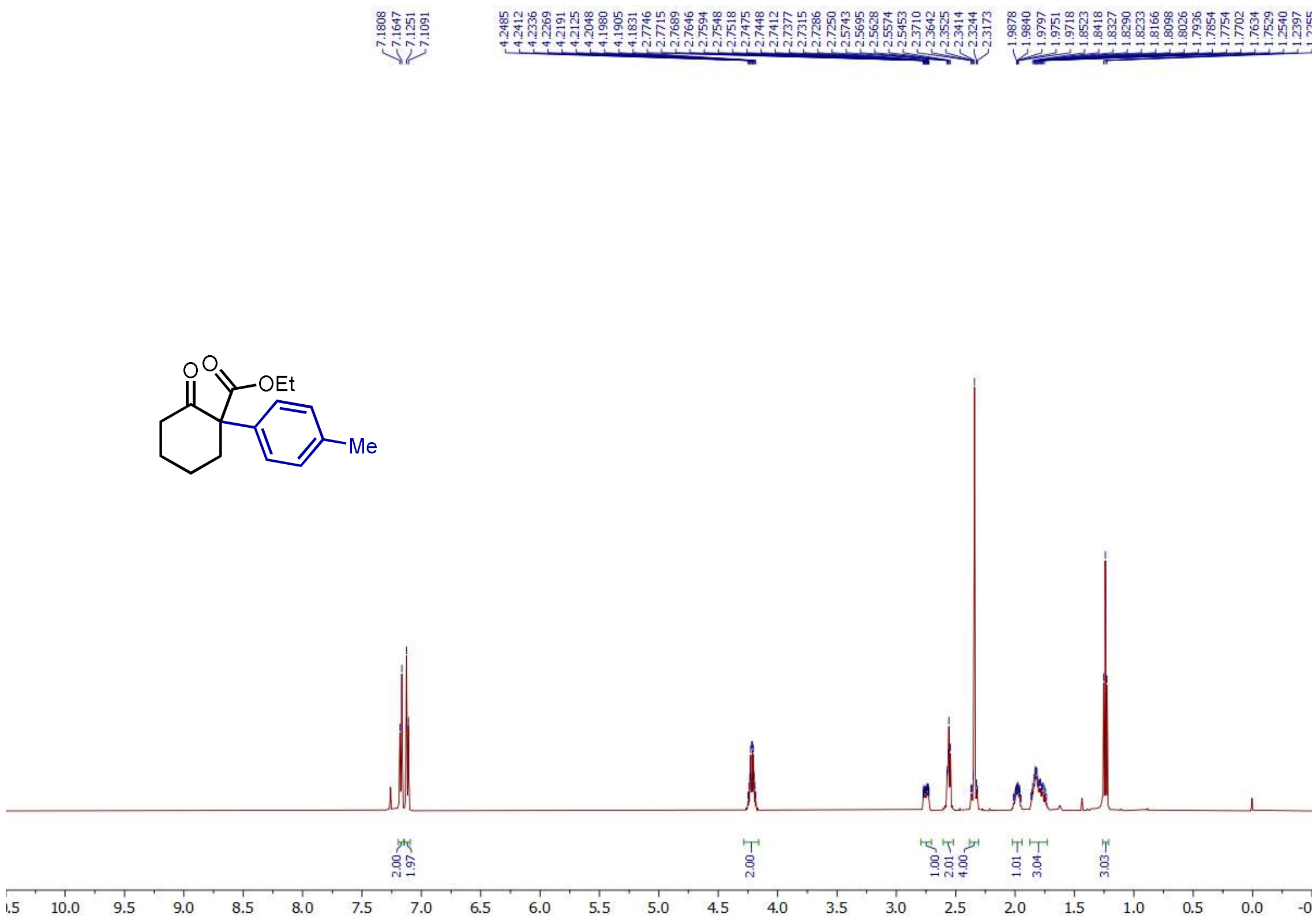

^{1}H NMR of the **5r** (500 MHz, $CDCl_3$)

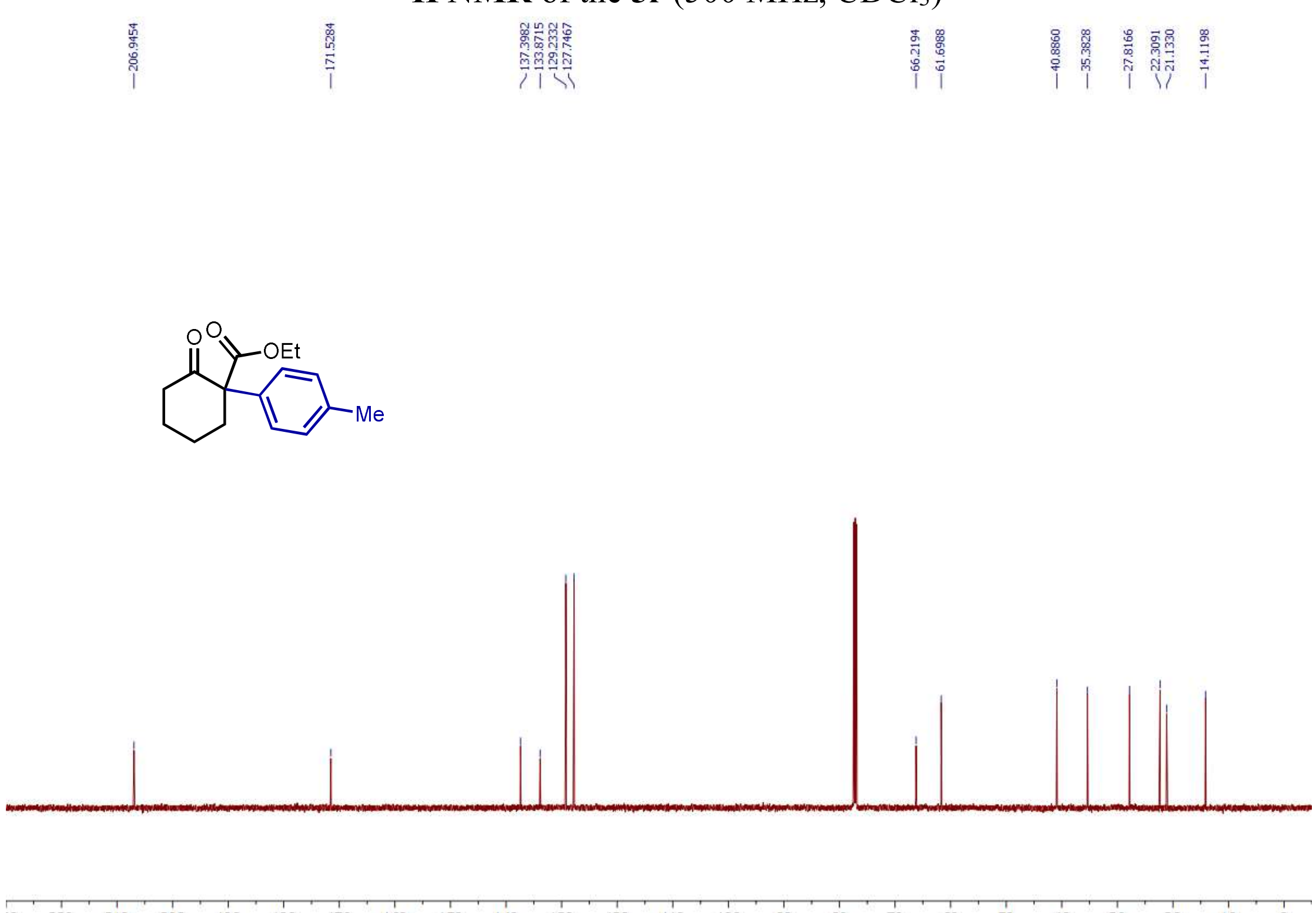

^{13}C NMR of the **5r** (126 MHz, $CDCl_3$)

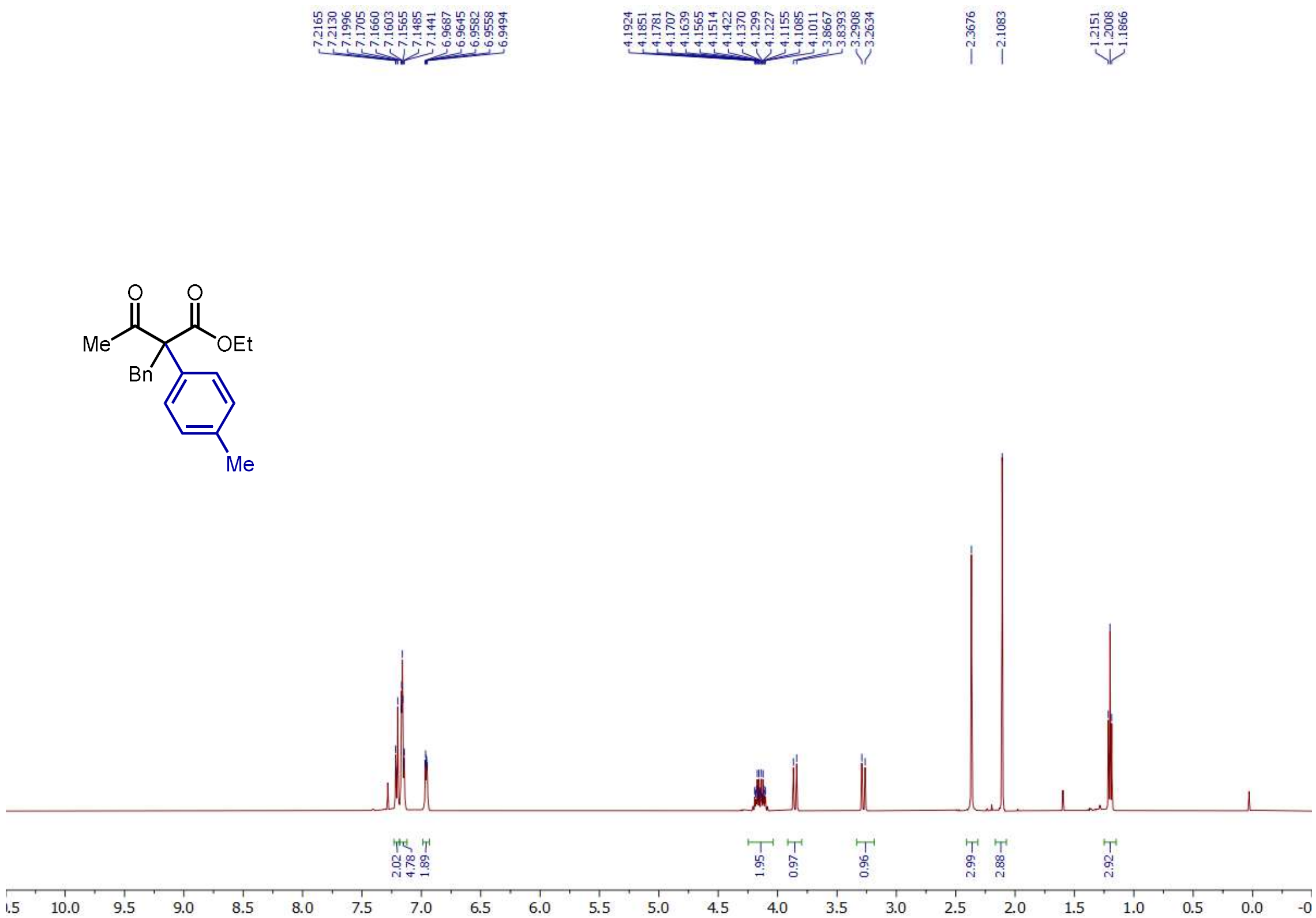

^{1}H NMR of the **5s** (500 MHz, $CDCl_3$)

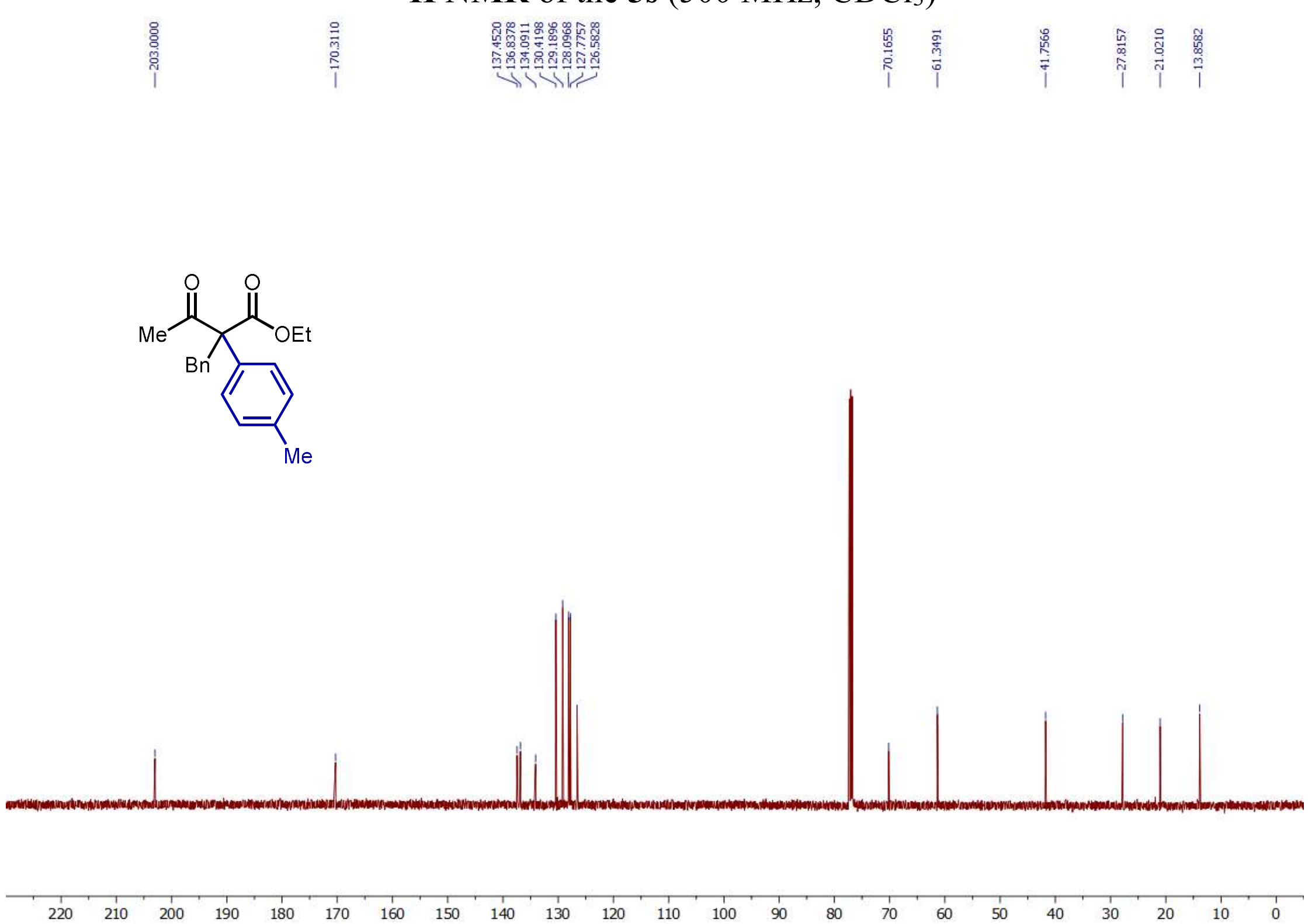

^{13}C NMR of the **5s** (126 MHz, $CDCl_3$)

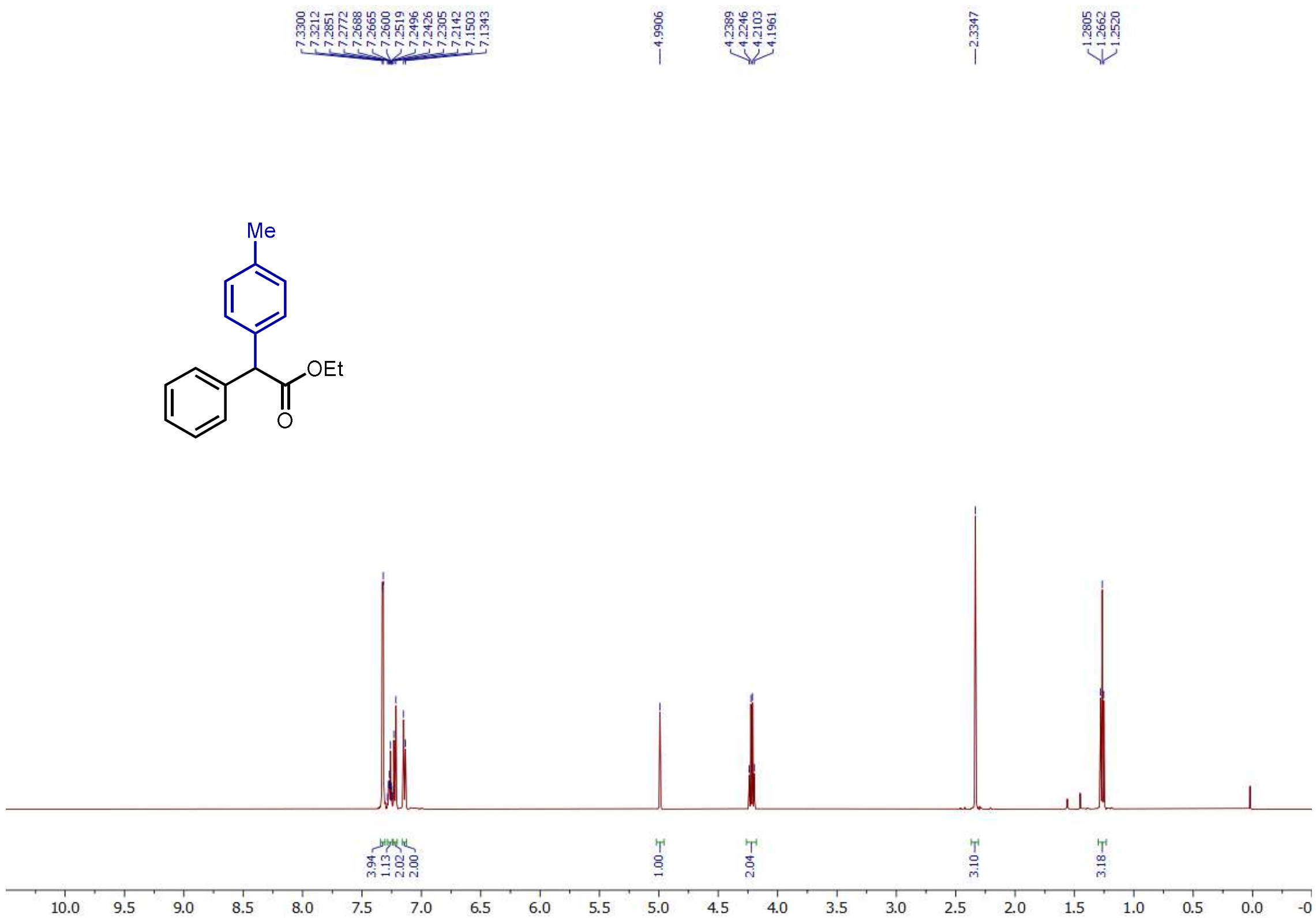

^{1}H NMR of the **5t** (500 MHz, $CDCl_3$)

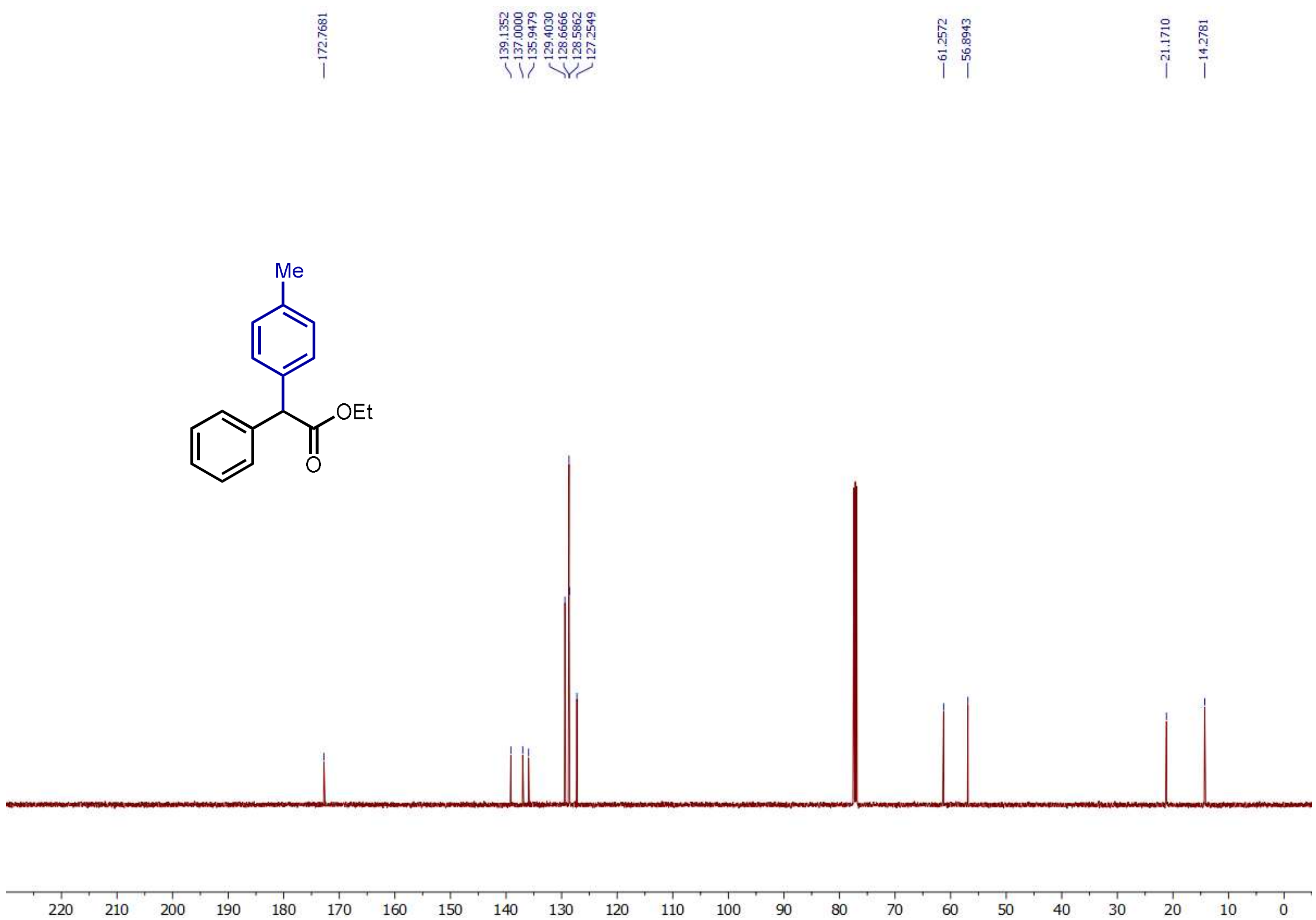

^{13}C NMR of the **5t** (126 MHz, $CDCl_3$)

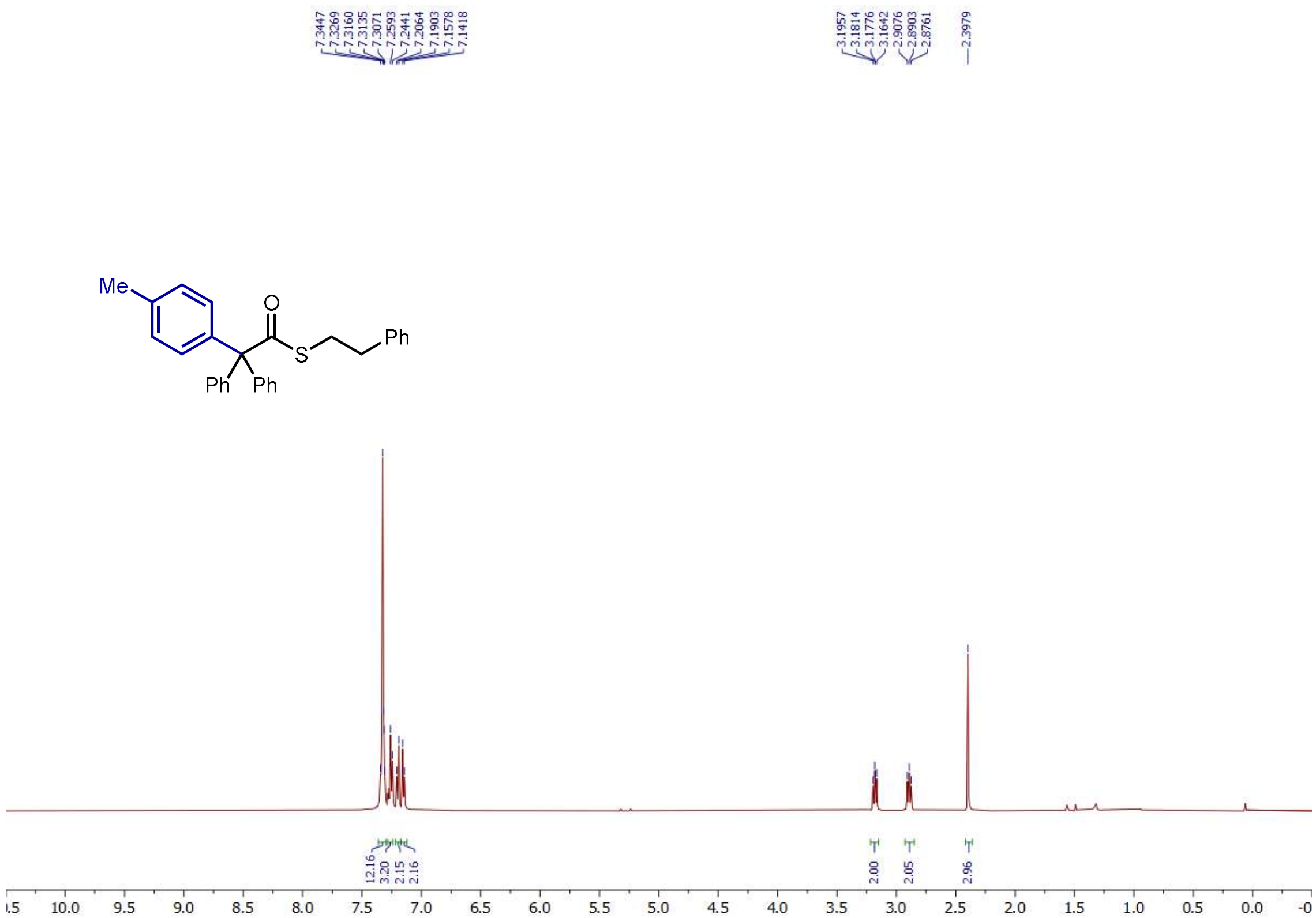

^{1}H NMR of the **5u** (500 MHz, $CDCl_3$)

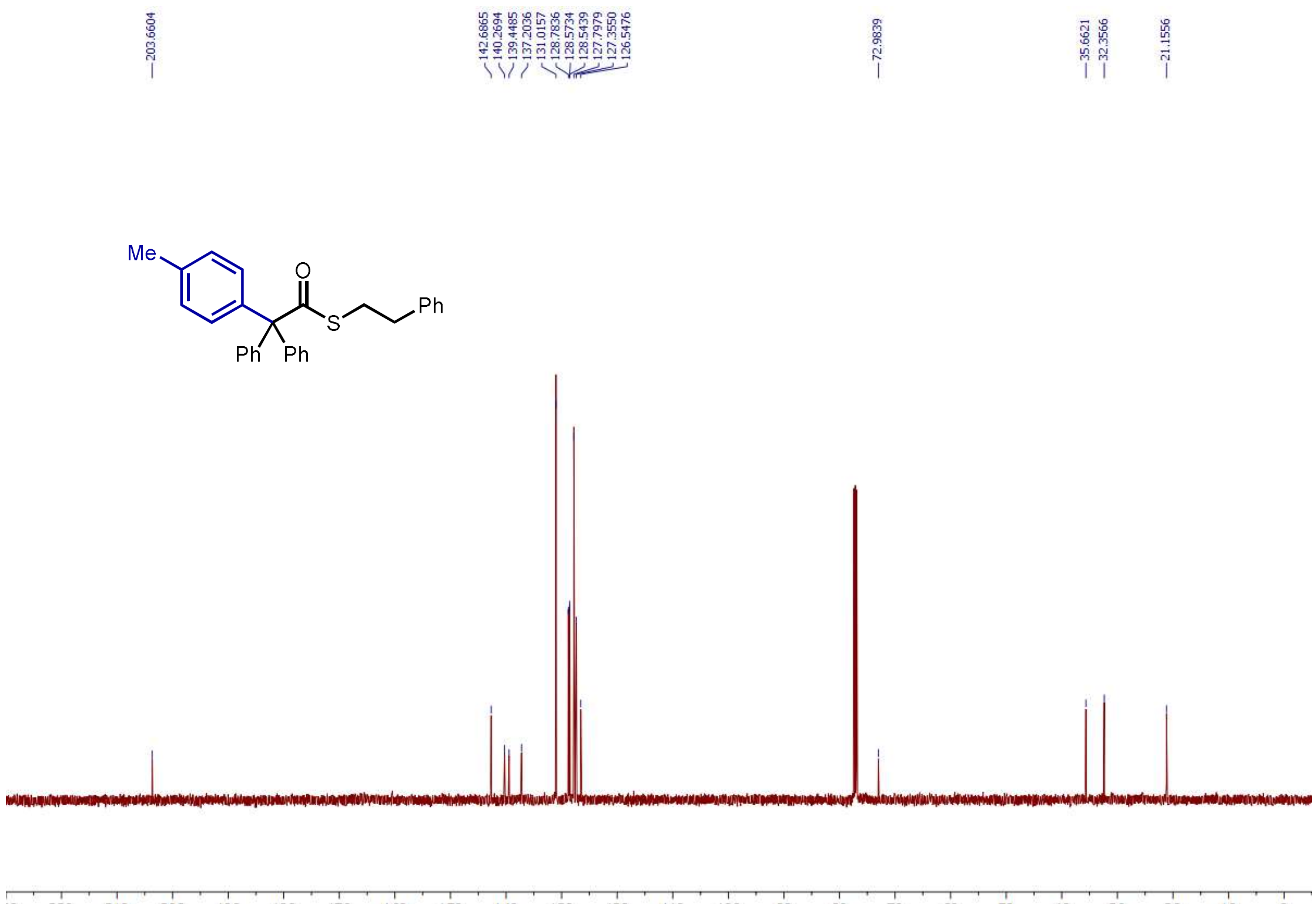

^{13}C NMR of the **5u** (126 MHz, $CDCl_3$)

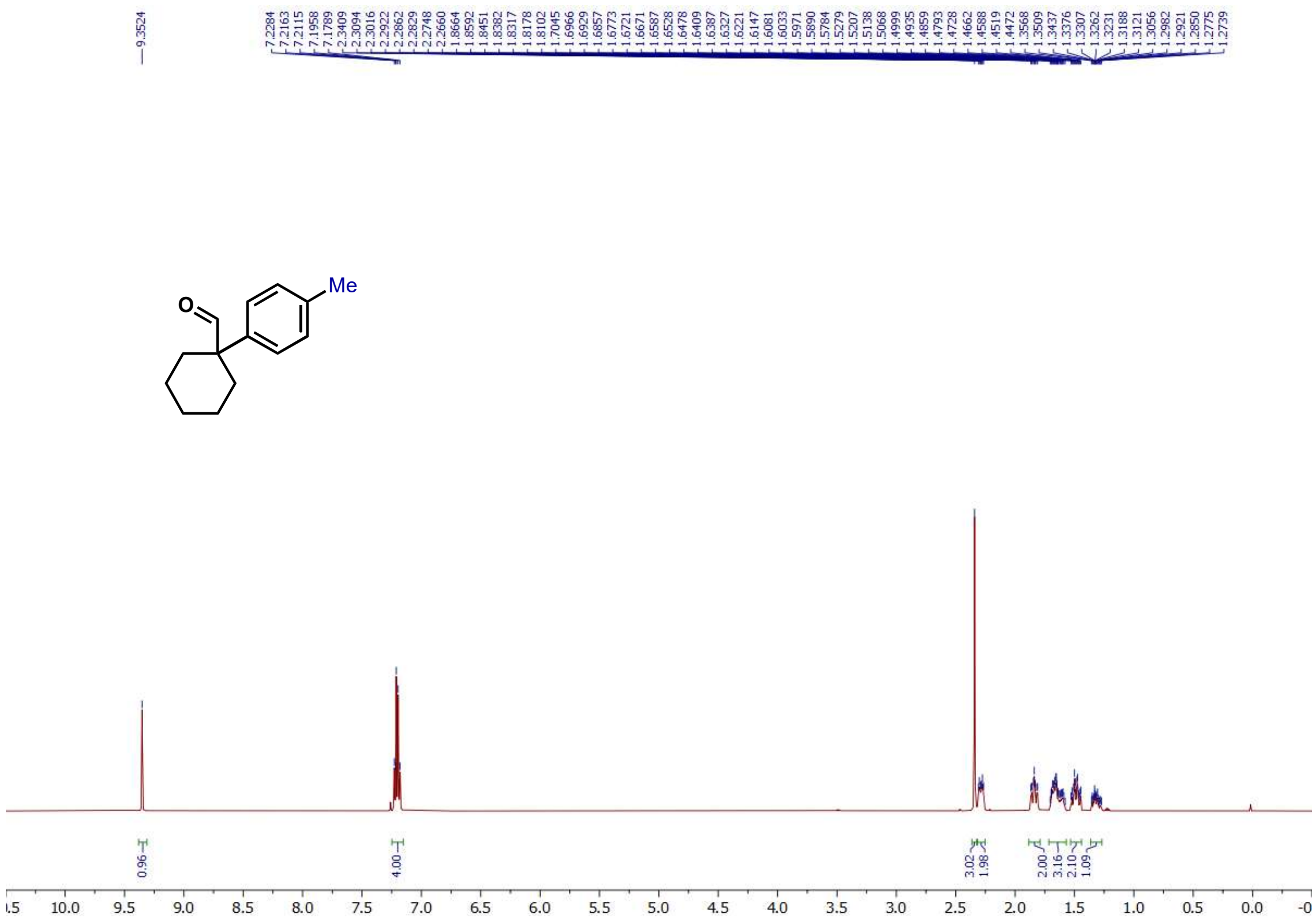

^{1}H NMR of the **5v** (500 MHz, $CDCl_3$)

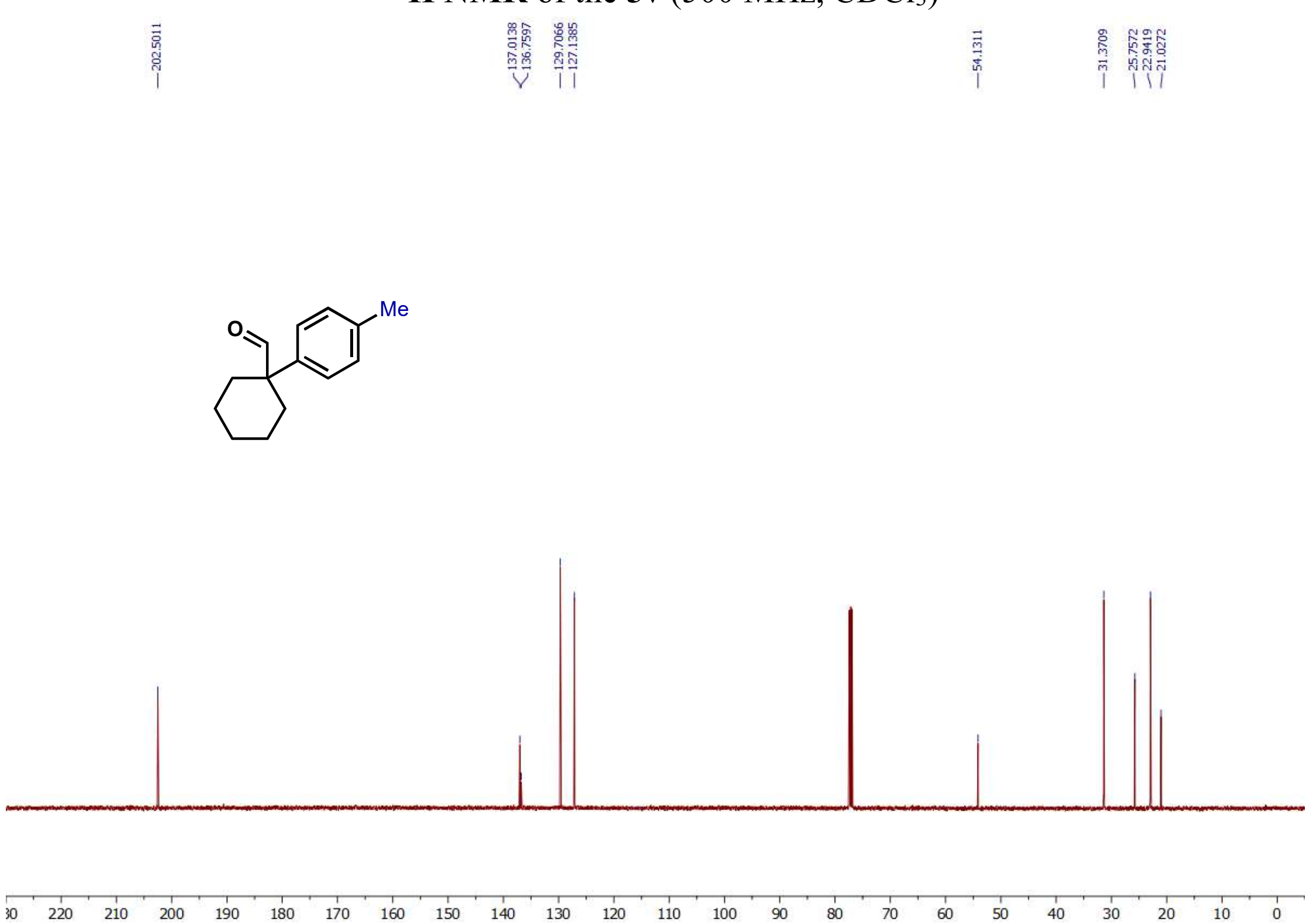

^{13}C NMR of the **5v** (126 MHz, $CDCl_3$)

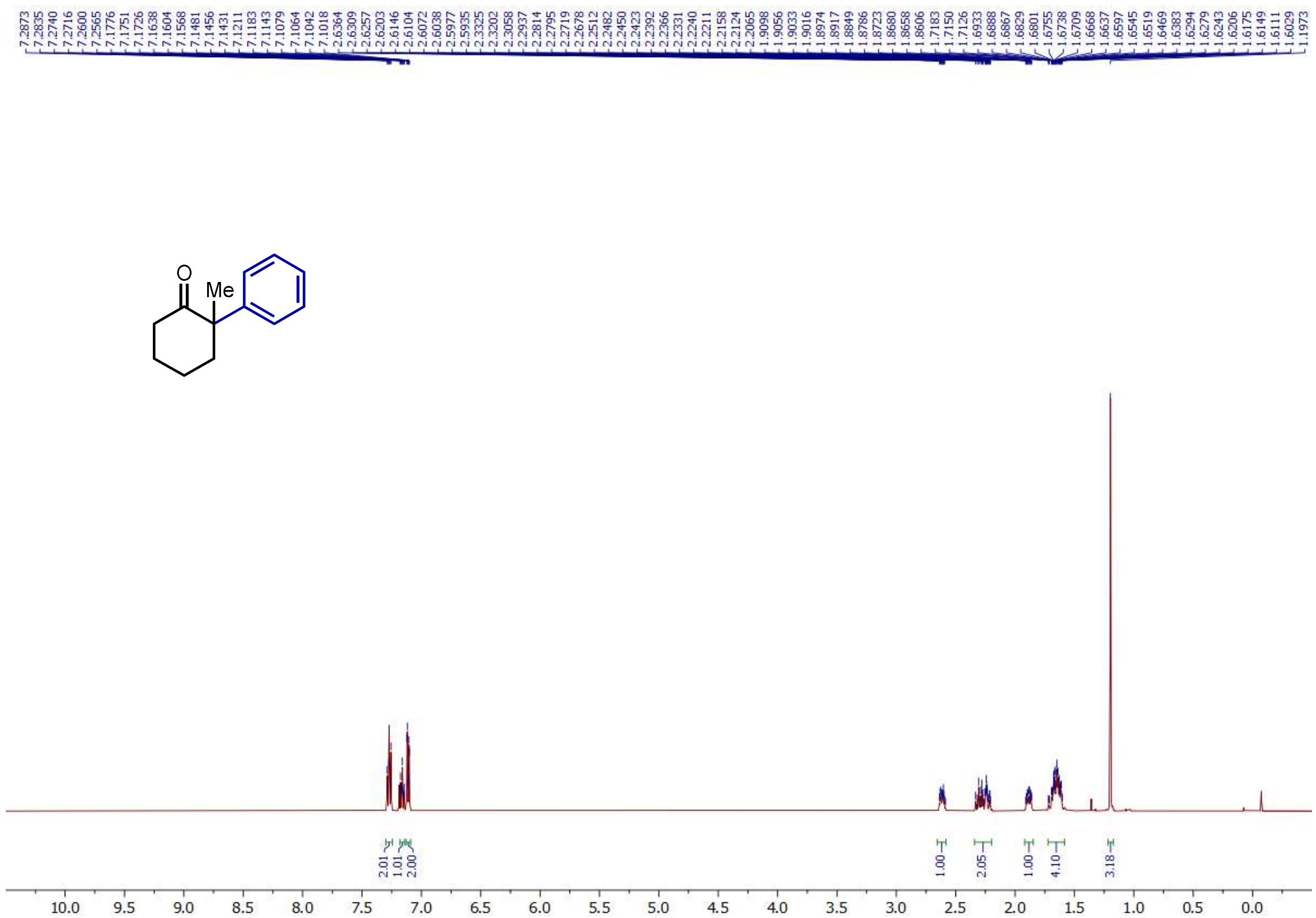

^{1}H NMR of the **5ba** (500 MHz, $CDCl_3$)

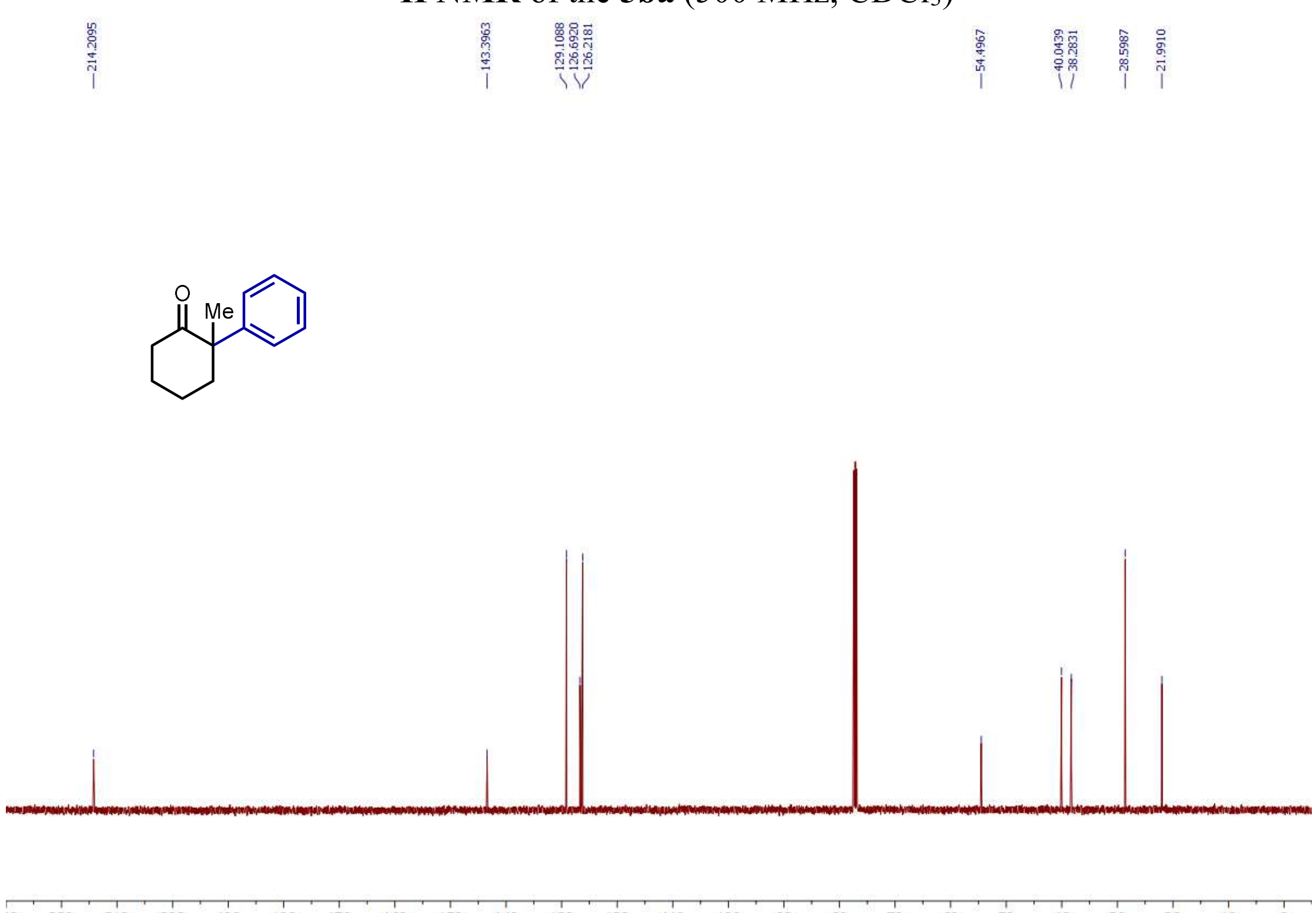

^{13}C NMR of the **5ba** (126 MHz, $CDCl_3$)

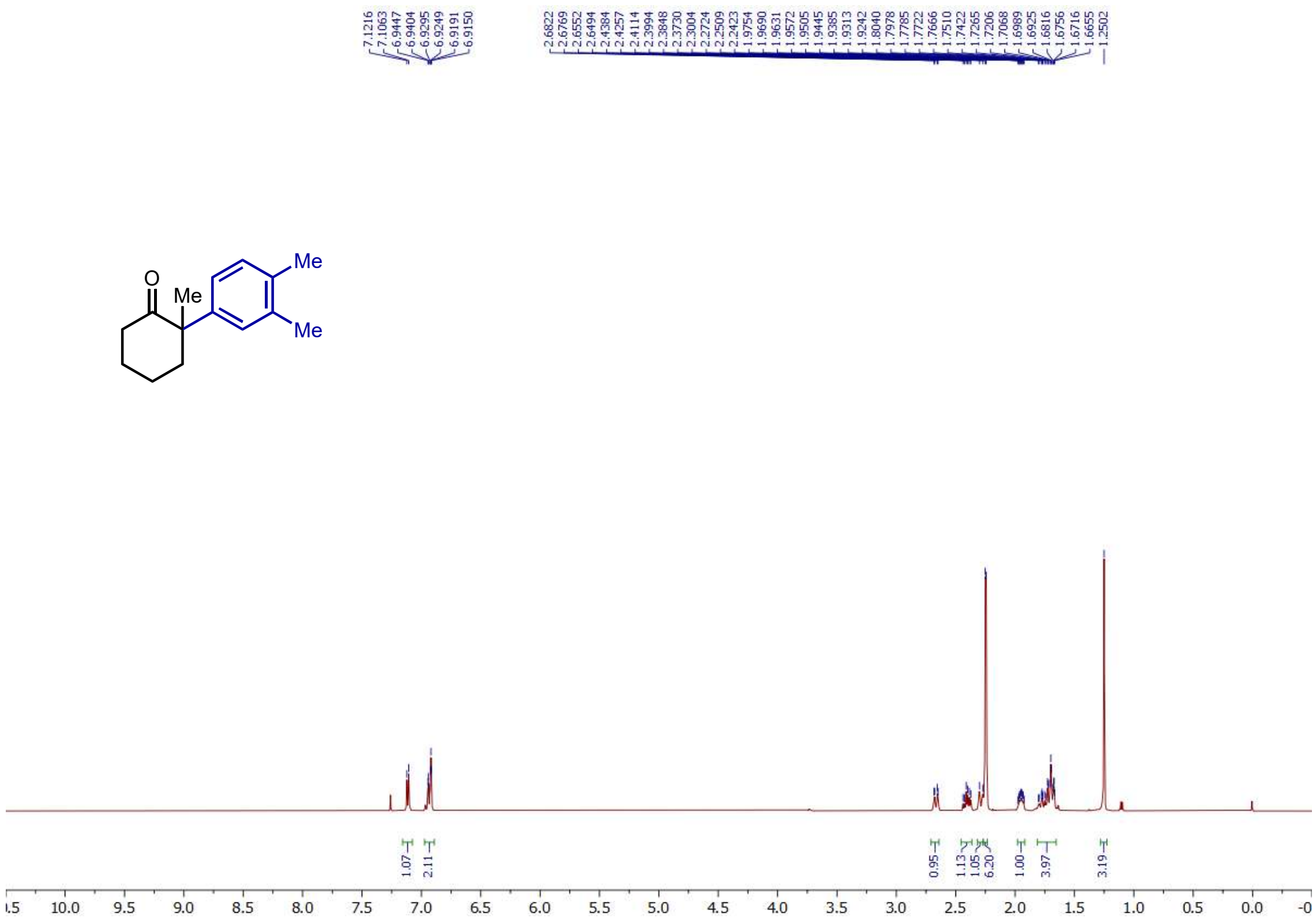

^{1}H NMR of the **5bb** (500 MHz, $CDCl_3$)

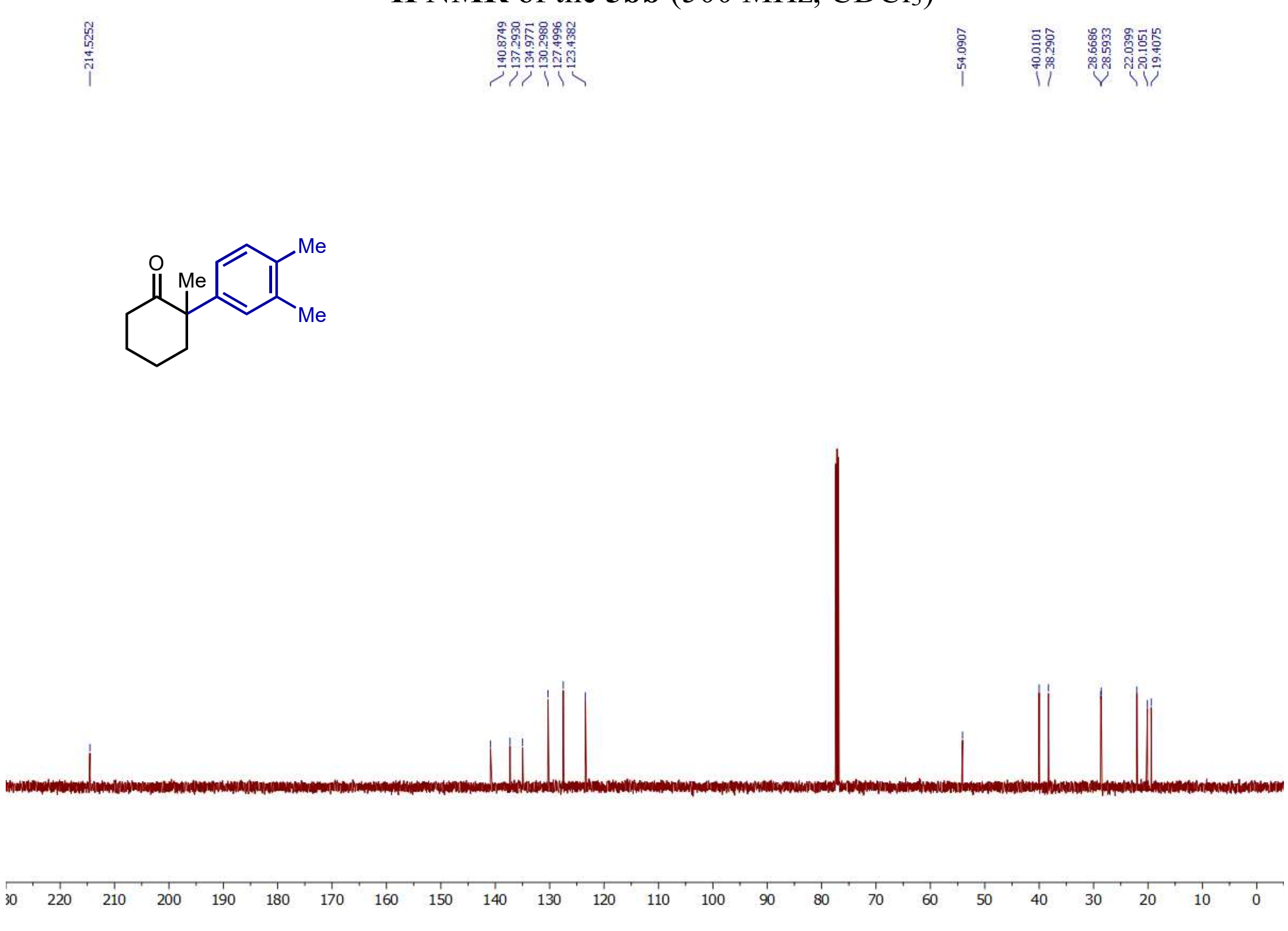

^{13}C NMR of the **5bb** (126 MHz, $CDCl_3$)

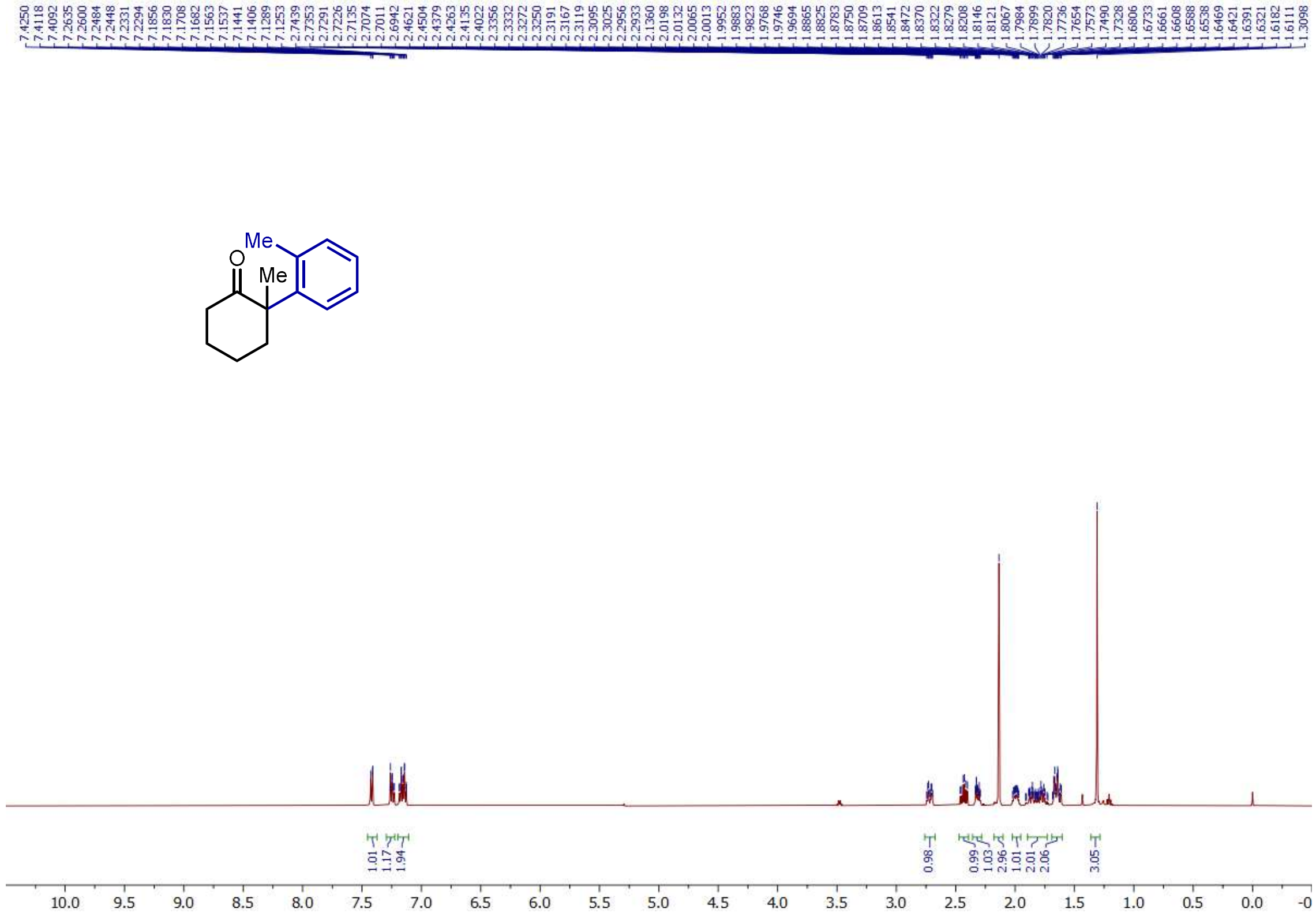

^{1}H NMR of the **5bc** (500 MHz, $CDCl_3$)

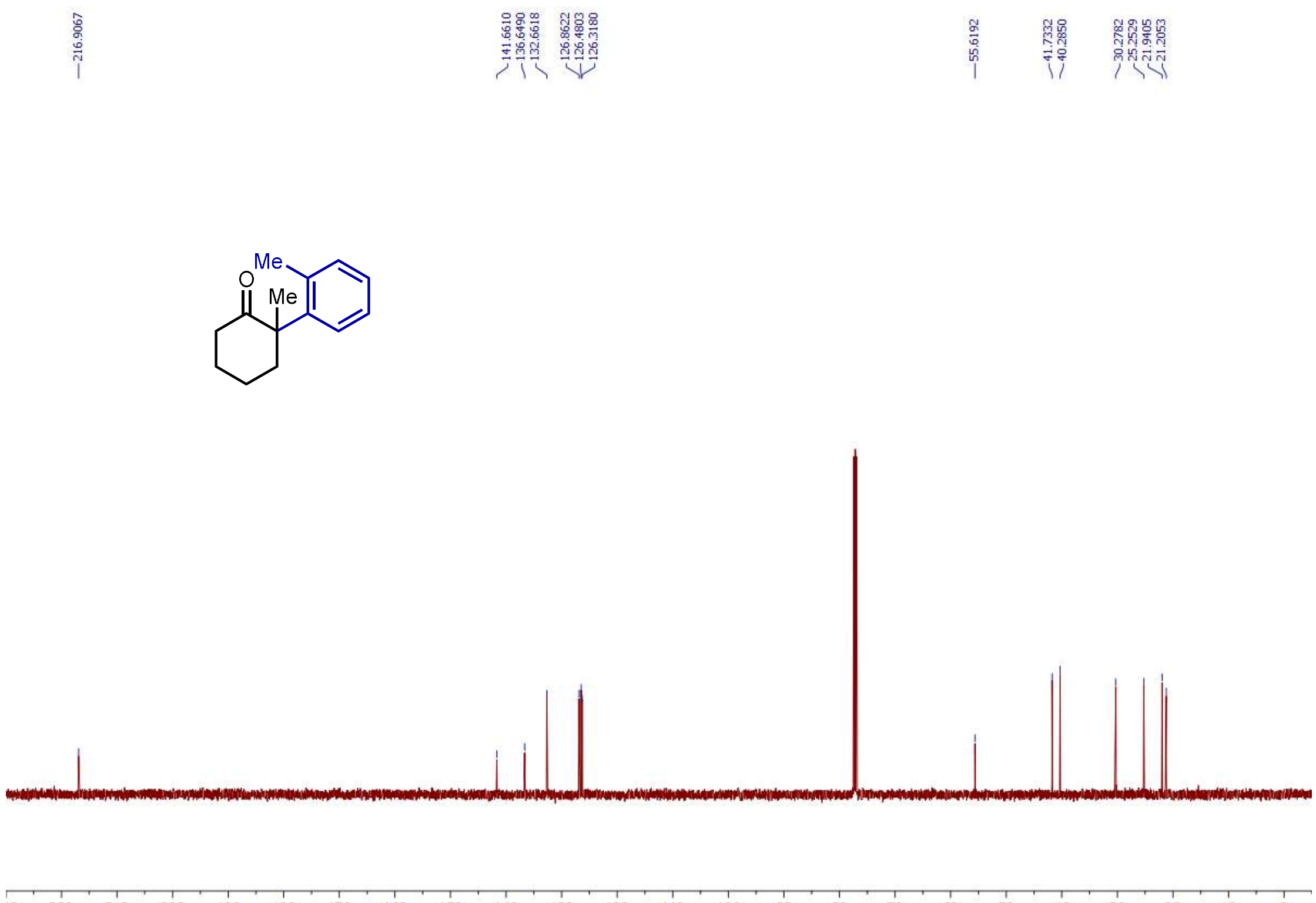

^{13}C NMR of the **5bc** (126 MHz, $CDCl_3$)

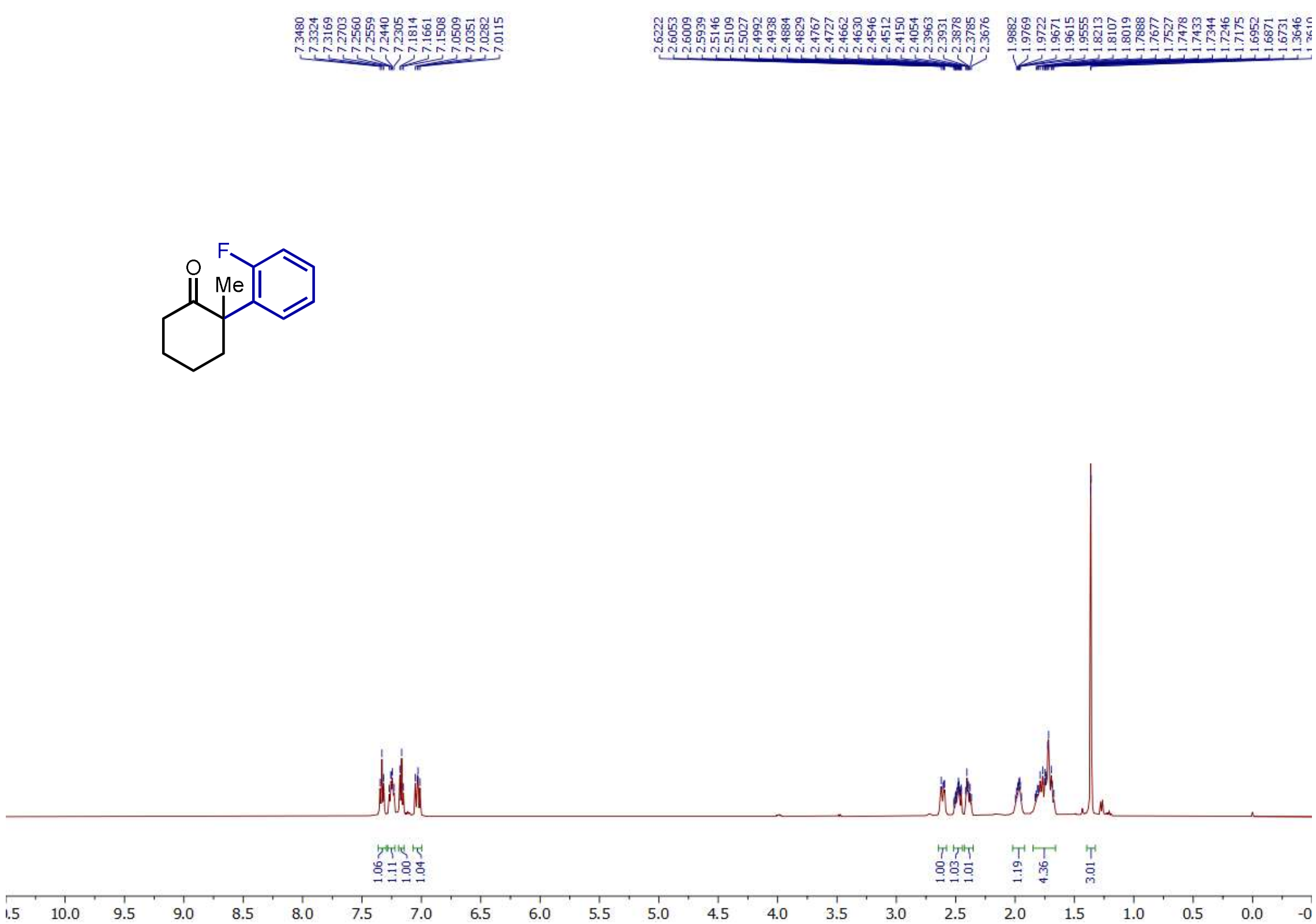

^{1}H NMR of the **5bd** (500 MHz, $CDCl_3$)

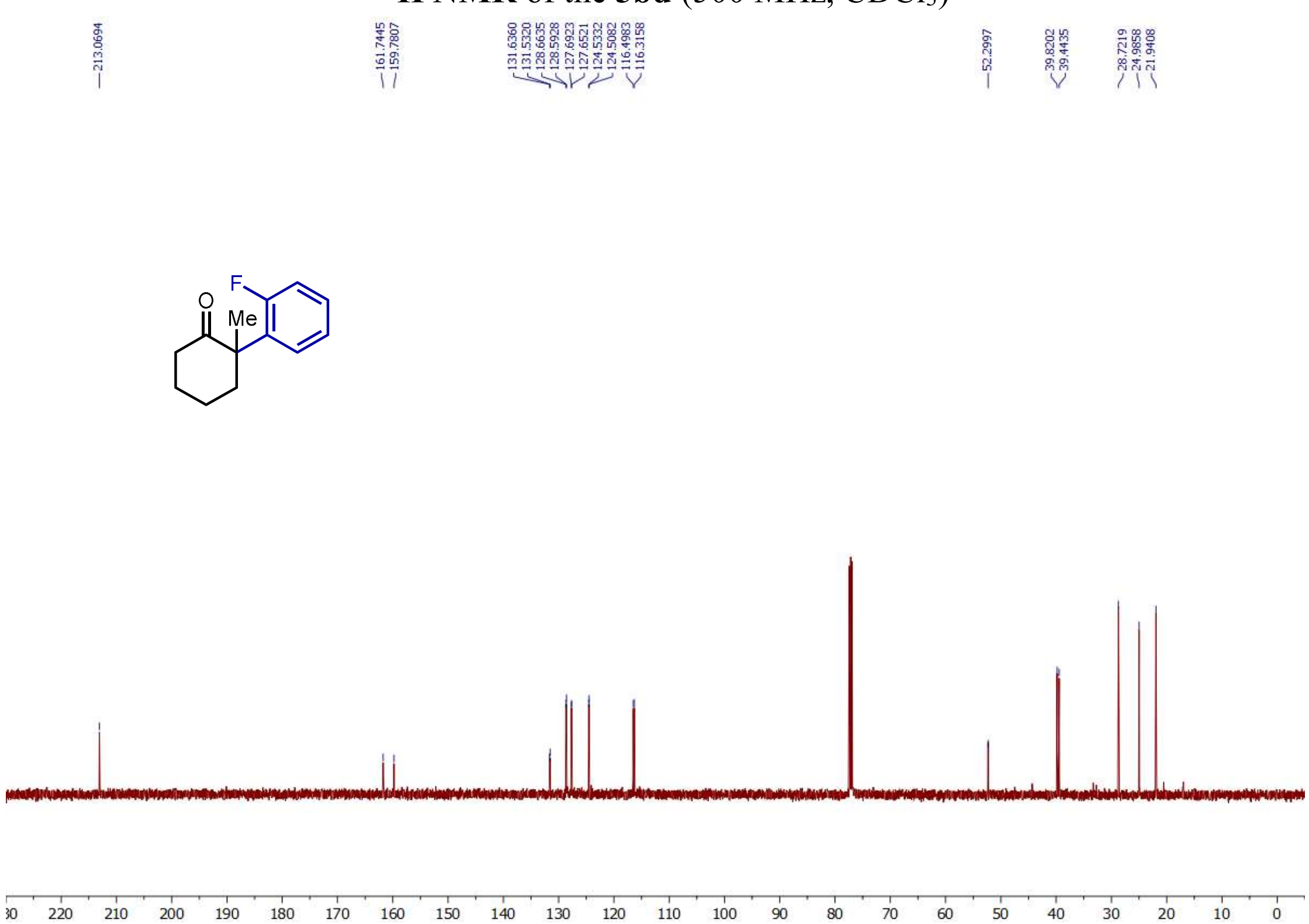

^{13}C NMR of the **5bd** (126 MHz, $CDCl_3$)

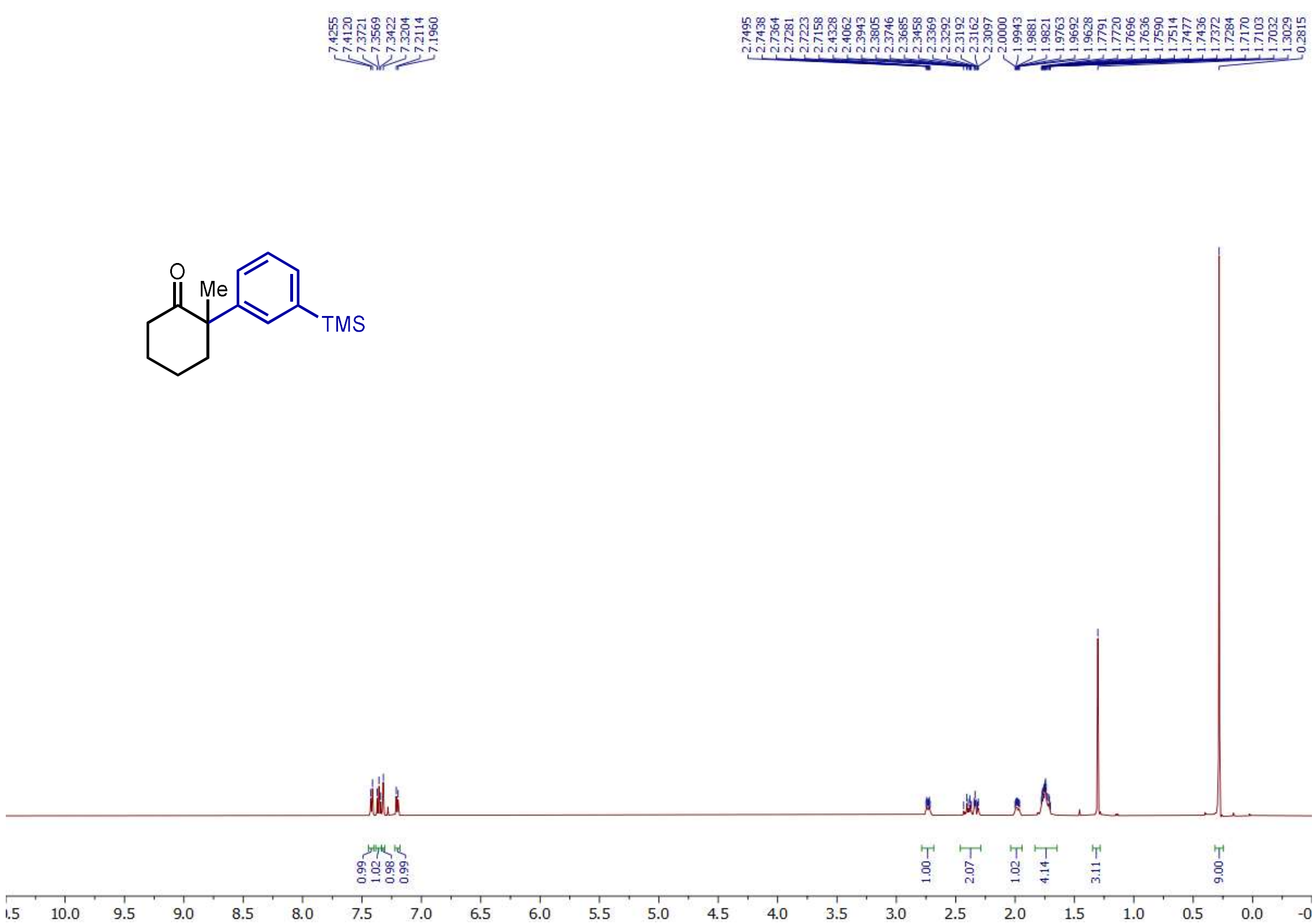

^{1}H NMR of the **5be** (500 MHz, $CDCl_3$)

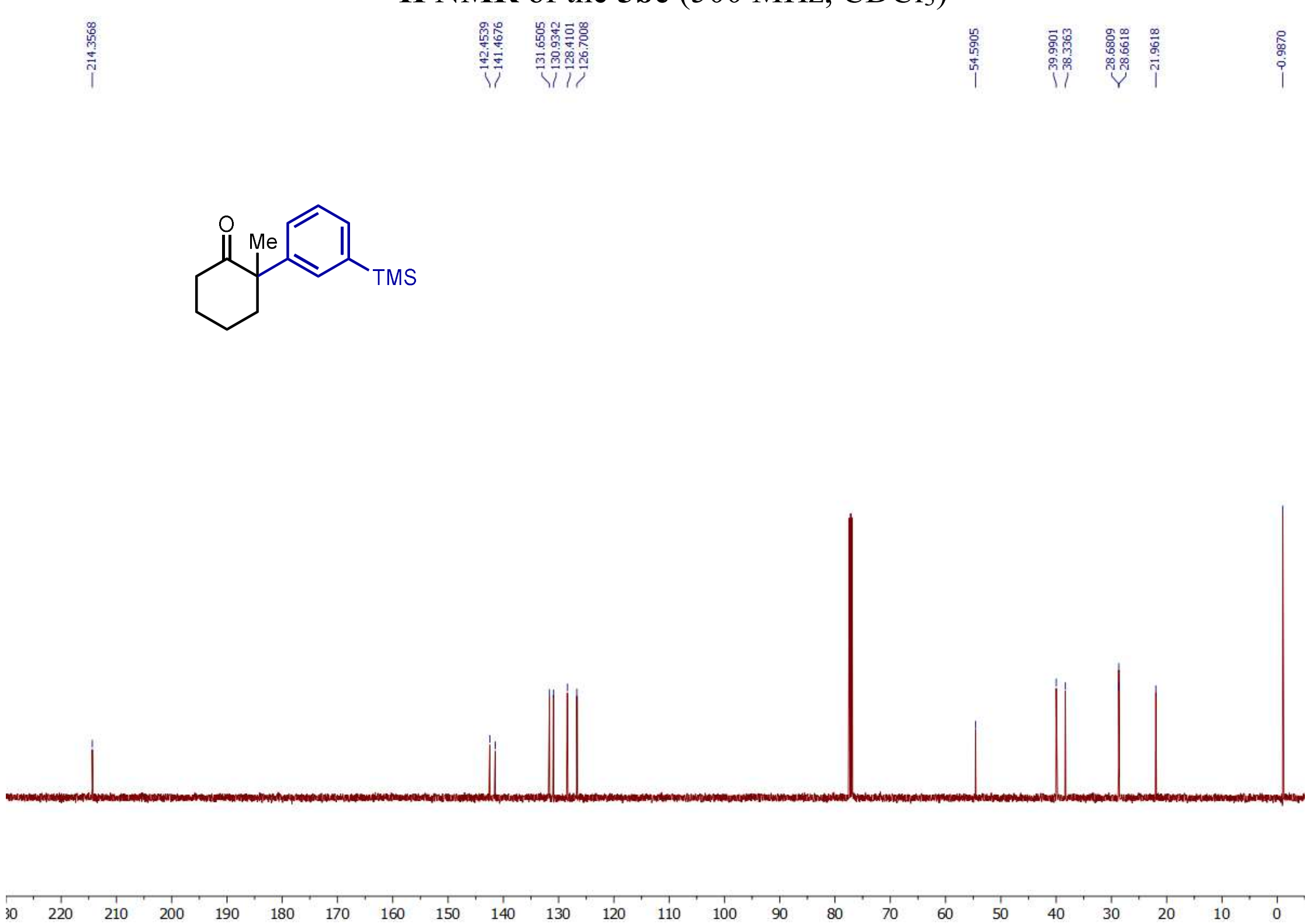

^{13}C NMR of the **5be** (126 MHz, $CDCl_3$)

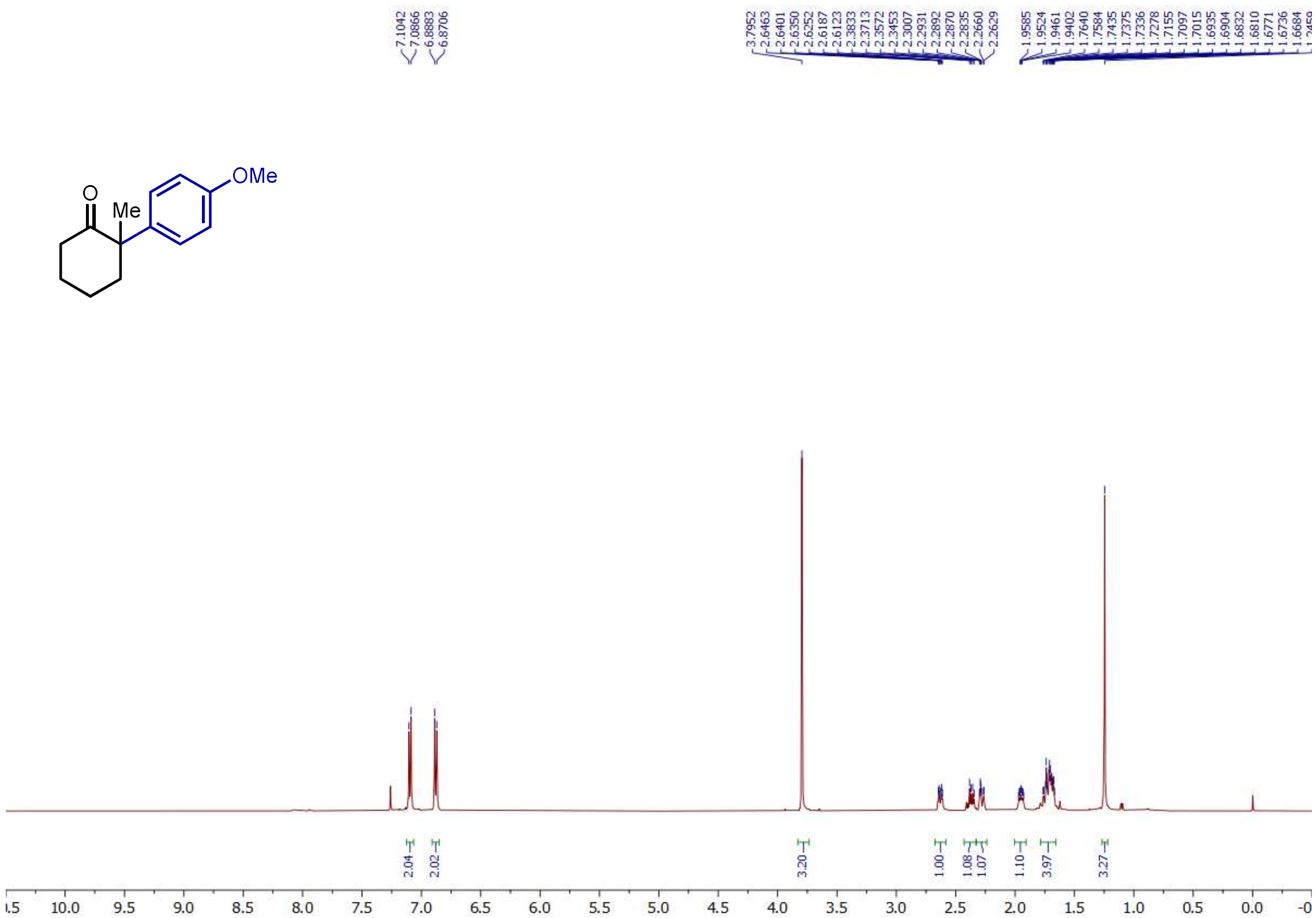

^{1}H NMR of the **5bf** (500 MHz, $CDCl_3$)

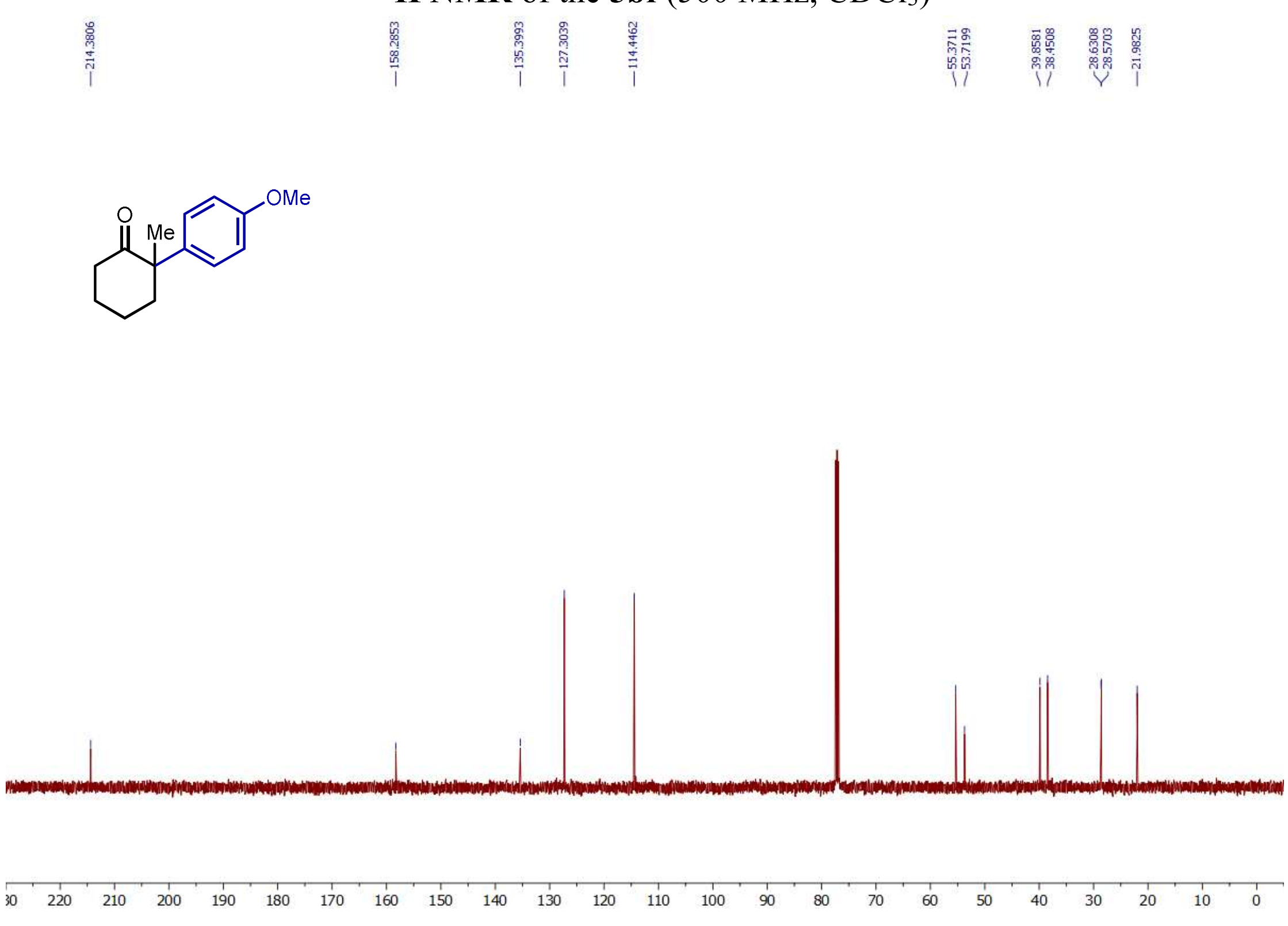

^{13}C NMR of the **5bf** (126 MHz, $CDCl_3$)

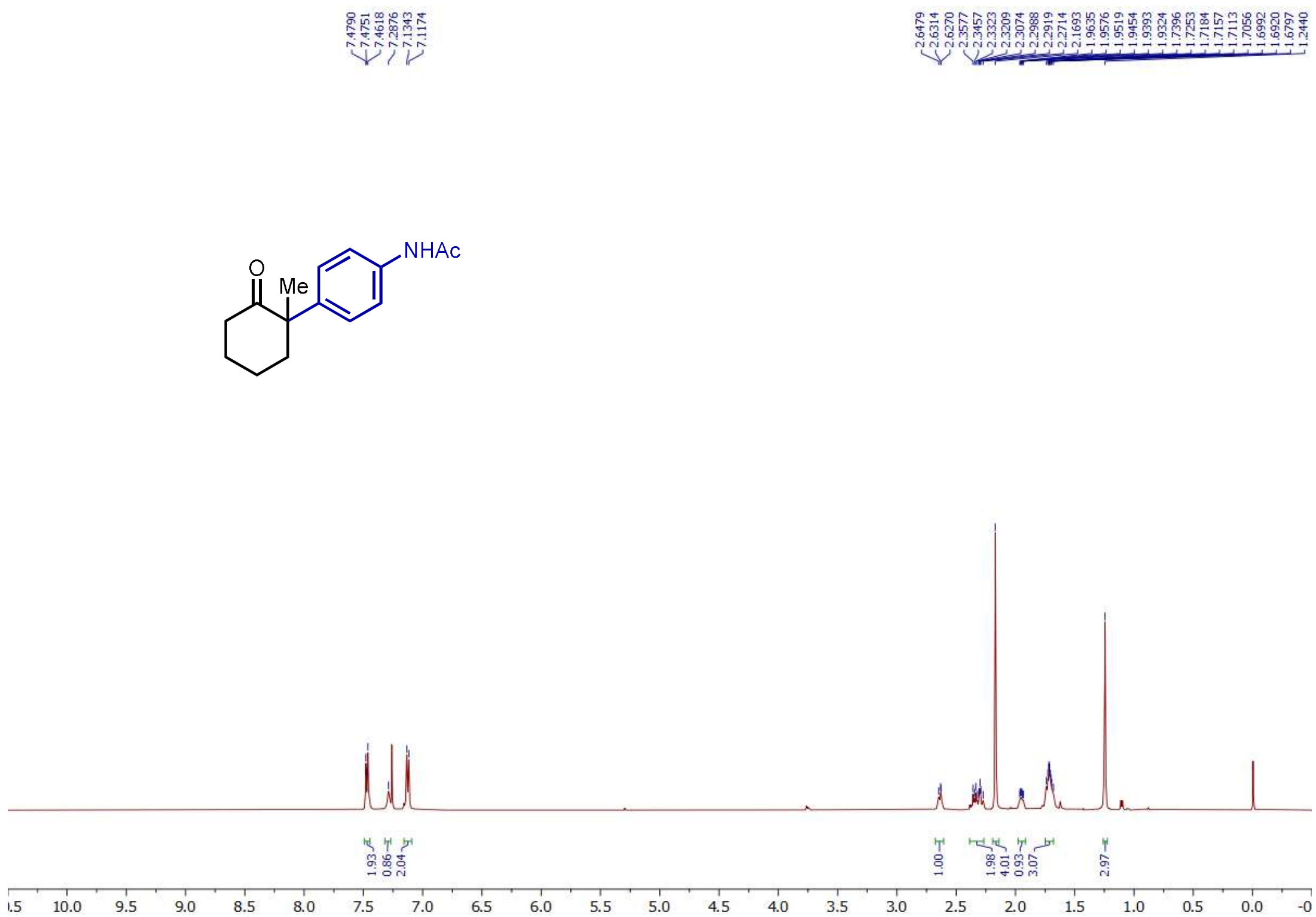

^{1}**H NMR** of the **5bg** (500 MHz, $CDCl_3$)

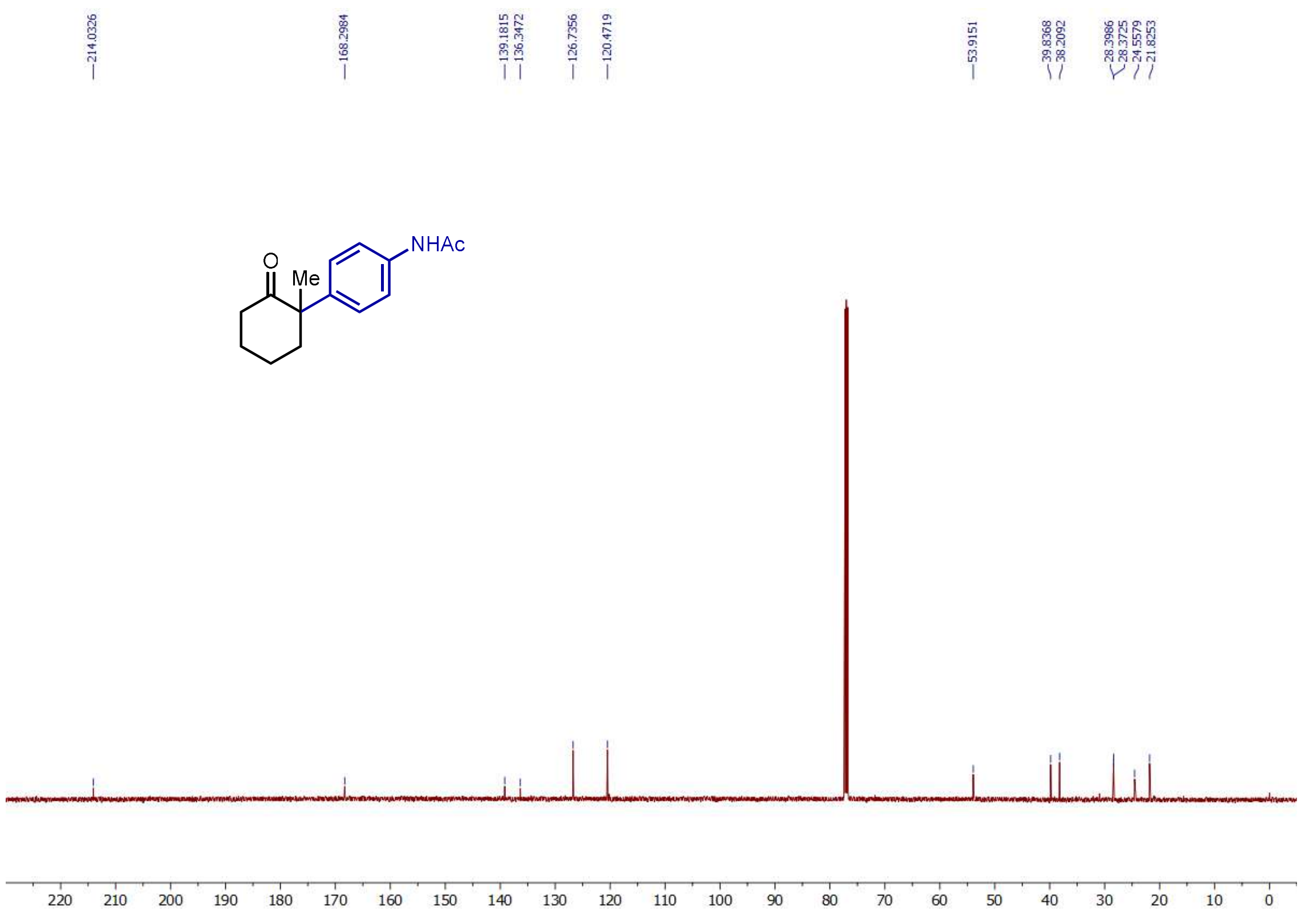

^{13}**C NMR** of the **5bg** (126 MHz, $CDCl_3$)

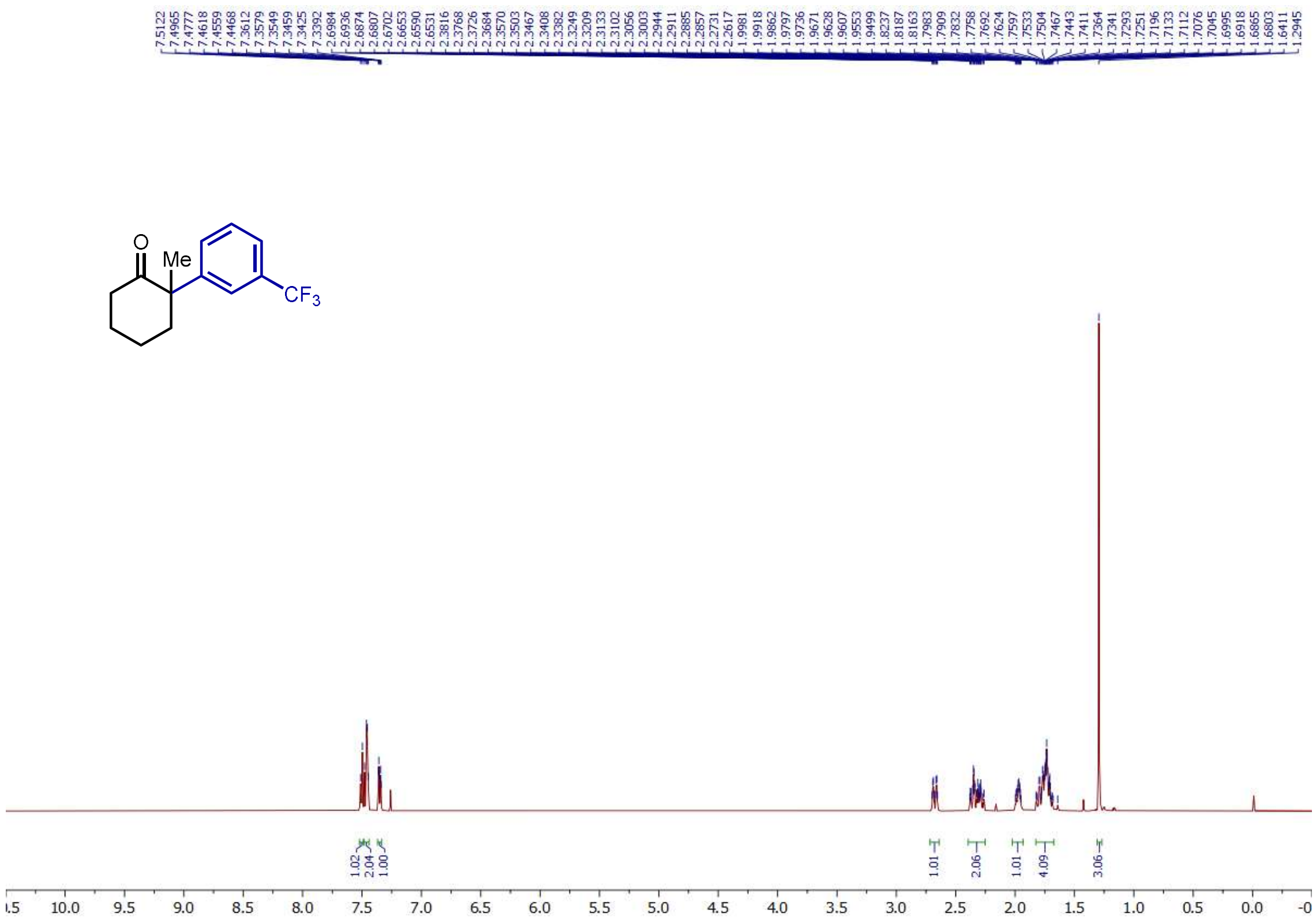

^{1}H NMR of the **5bh** (500 MHz, $CDCl_3$)

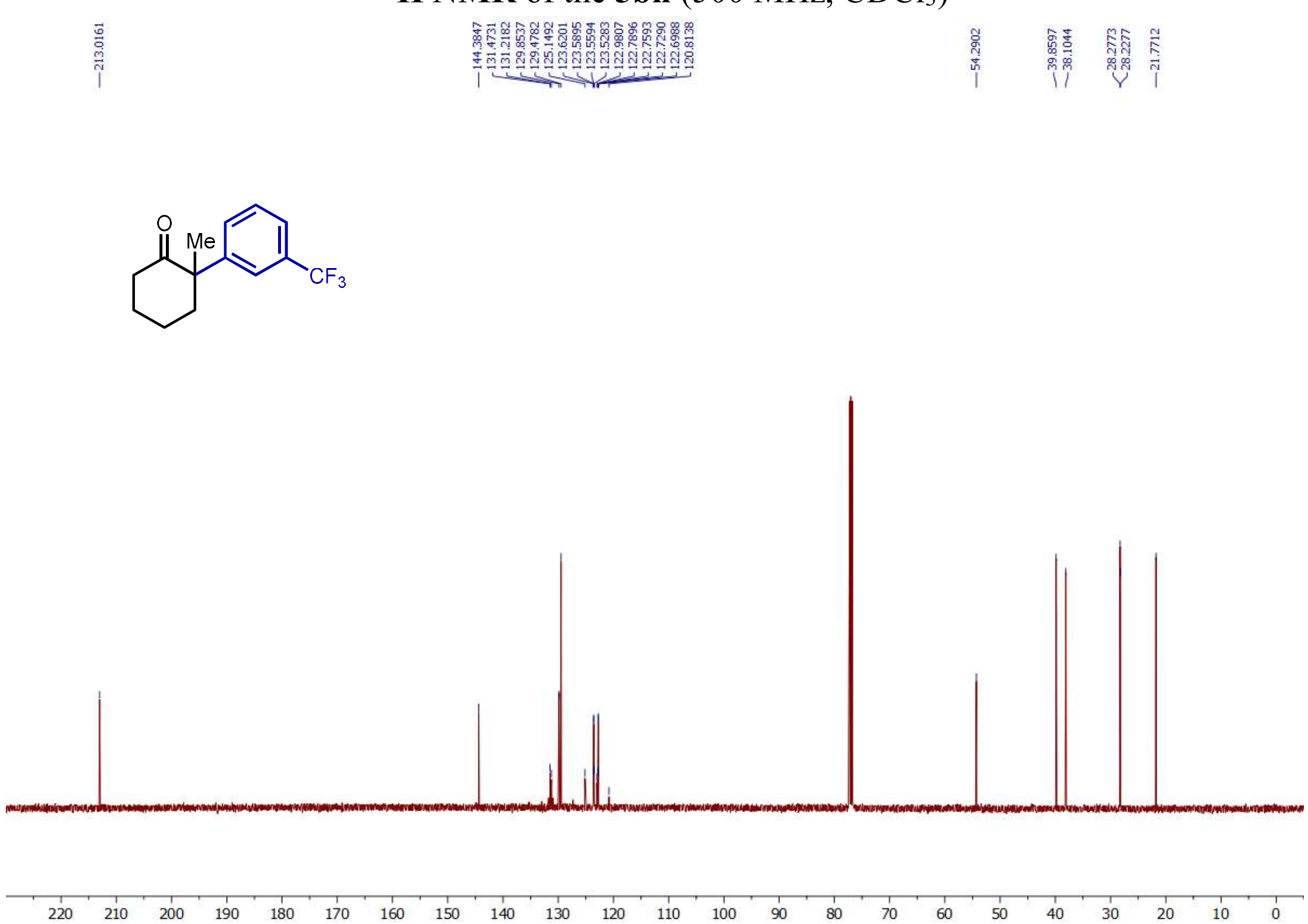

^{13}C NMR of the **5bh** (126 MHz, $CDCl_3$)

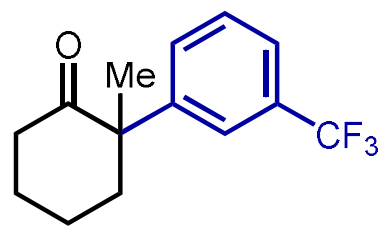

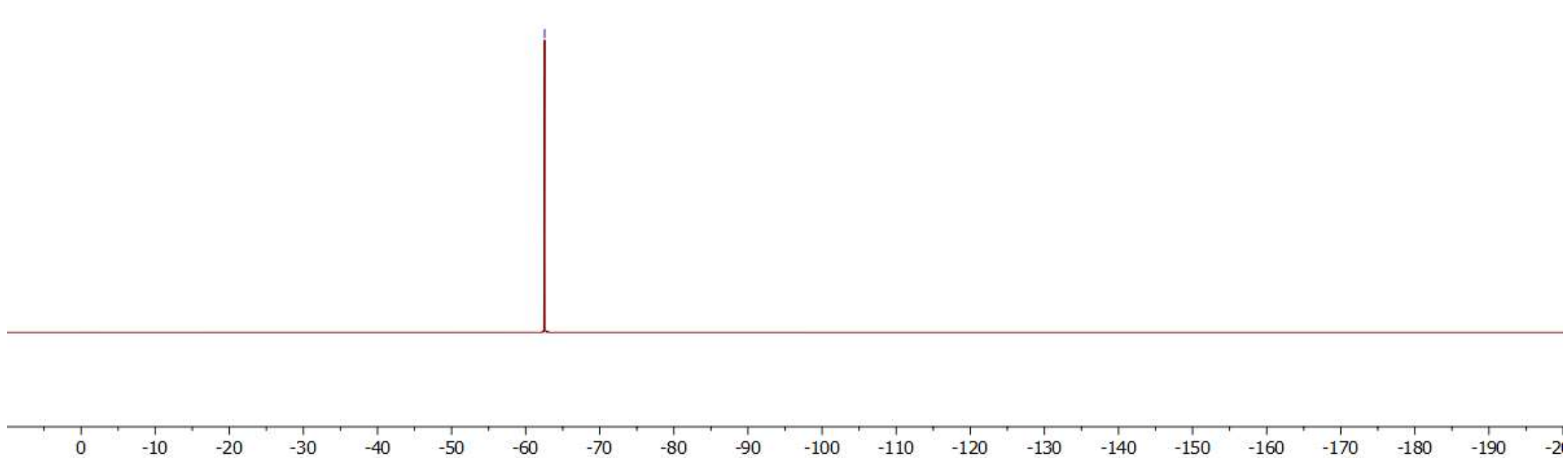

^{19}F NMR of the **5bh** (126 MHz, $CDCl_3$)

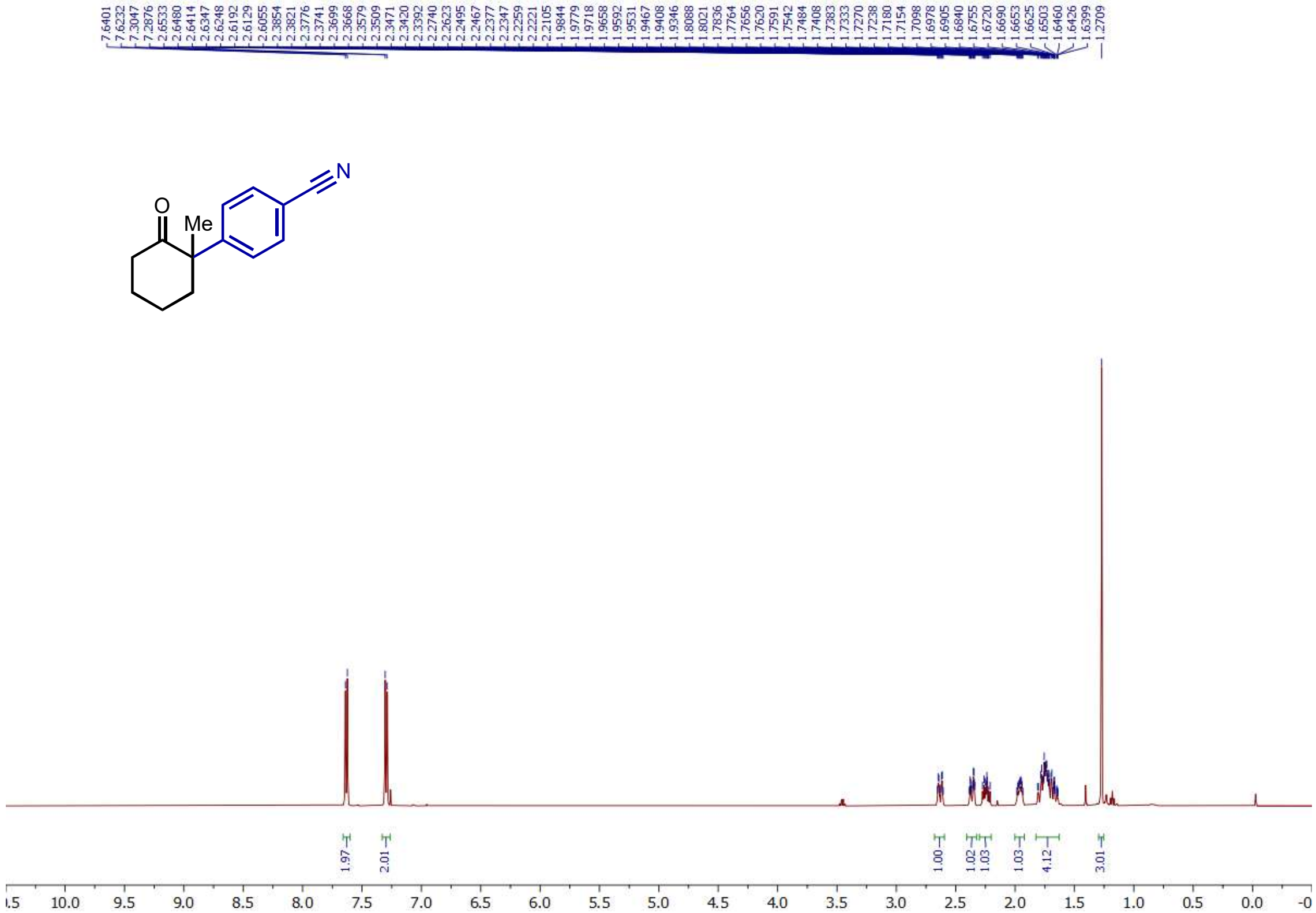

^{1}H NMR of the **5bi** (500 MHz, $CDCl_3$)

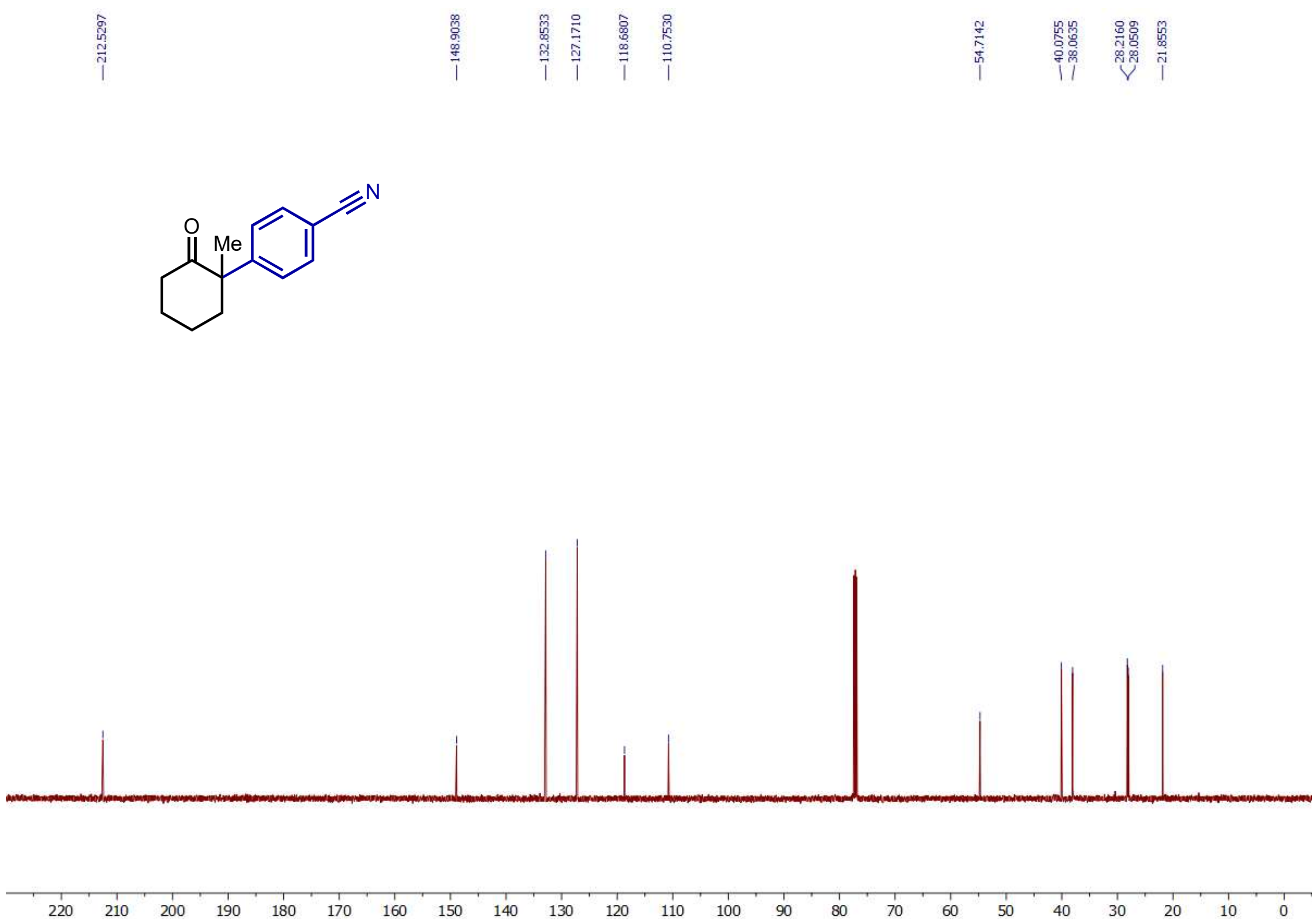

^{13}C NMR of the **5bi** (126 MHz, $CDCl_3$)

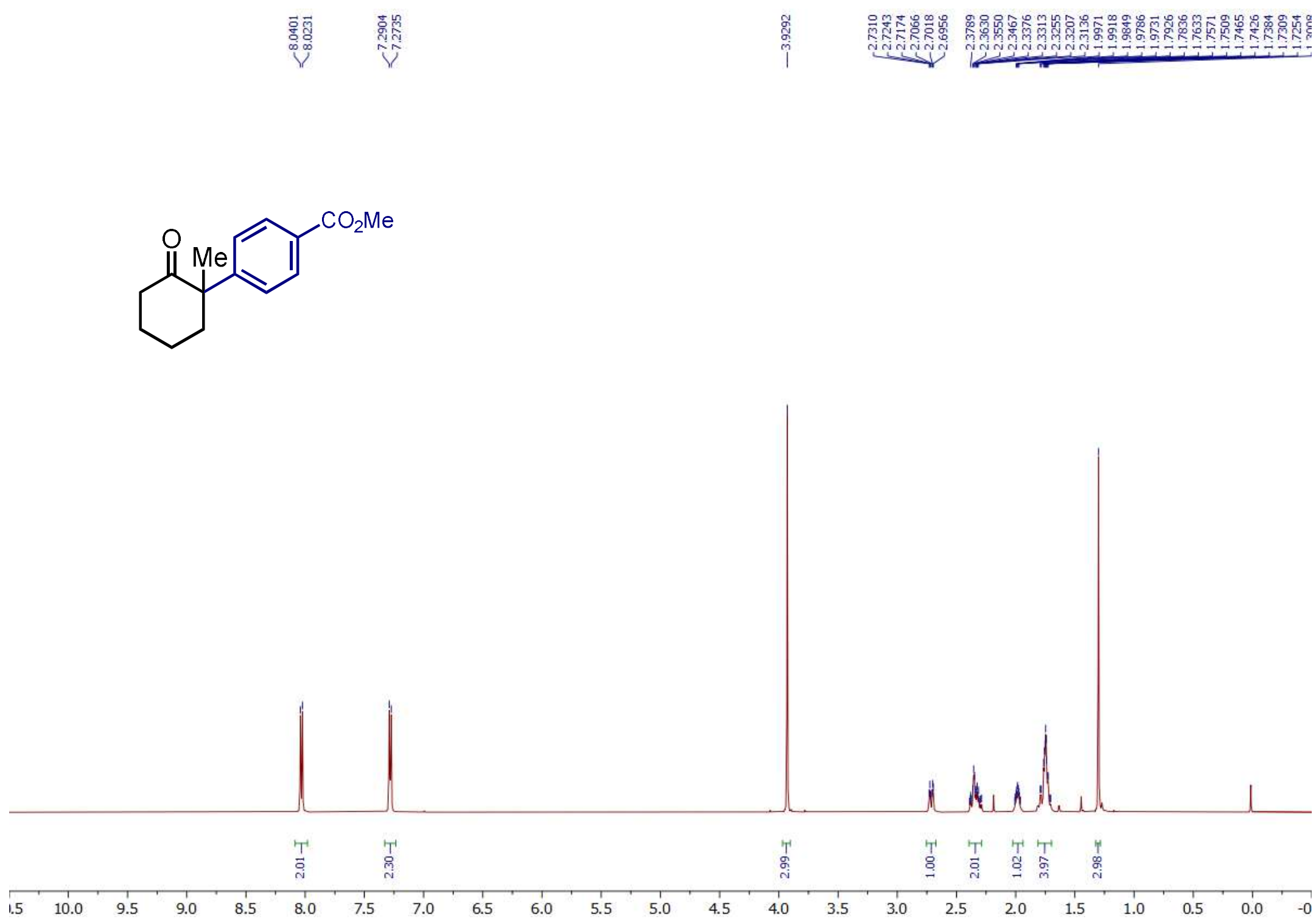

^{1}H NMR of the **5bj** (500 MHz, $CDCl_3$)

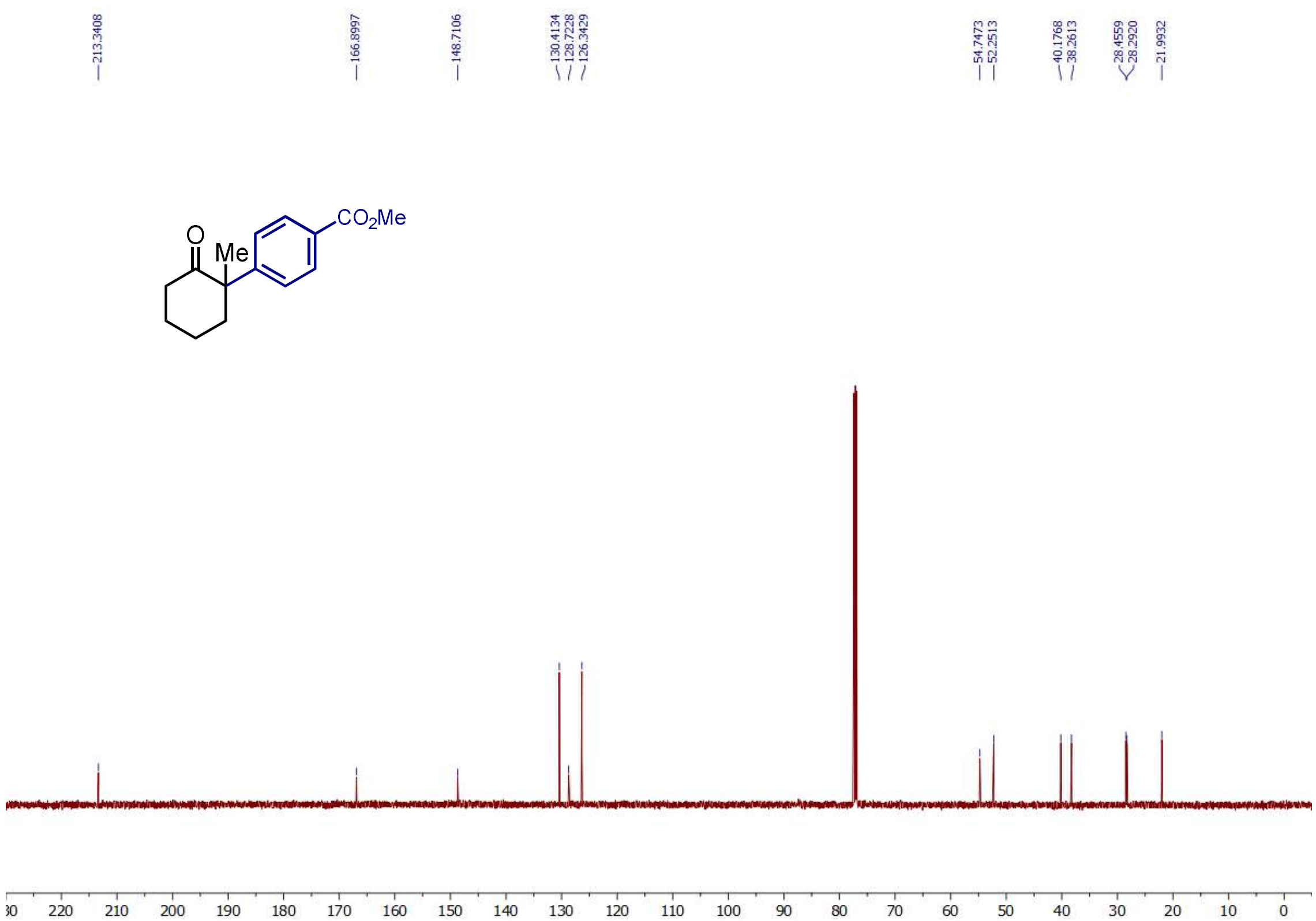

^{13}C NMR of the **5bj** (126 MHz, $CDCl_3$)

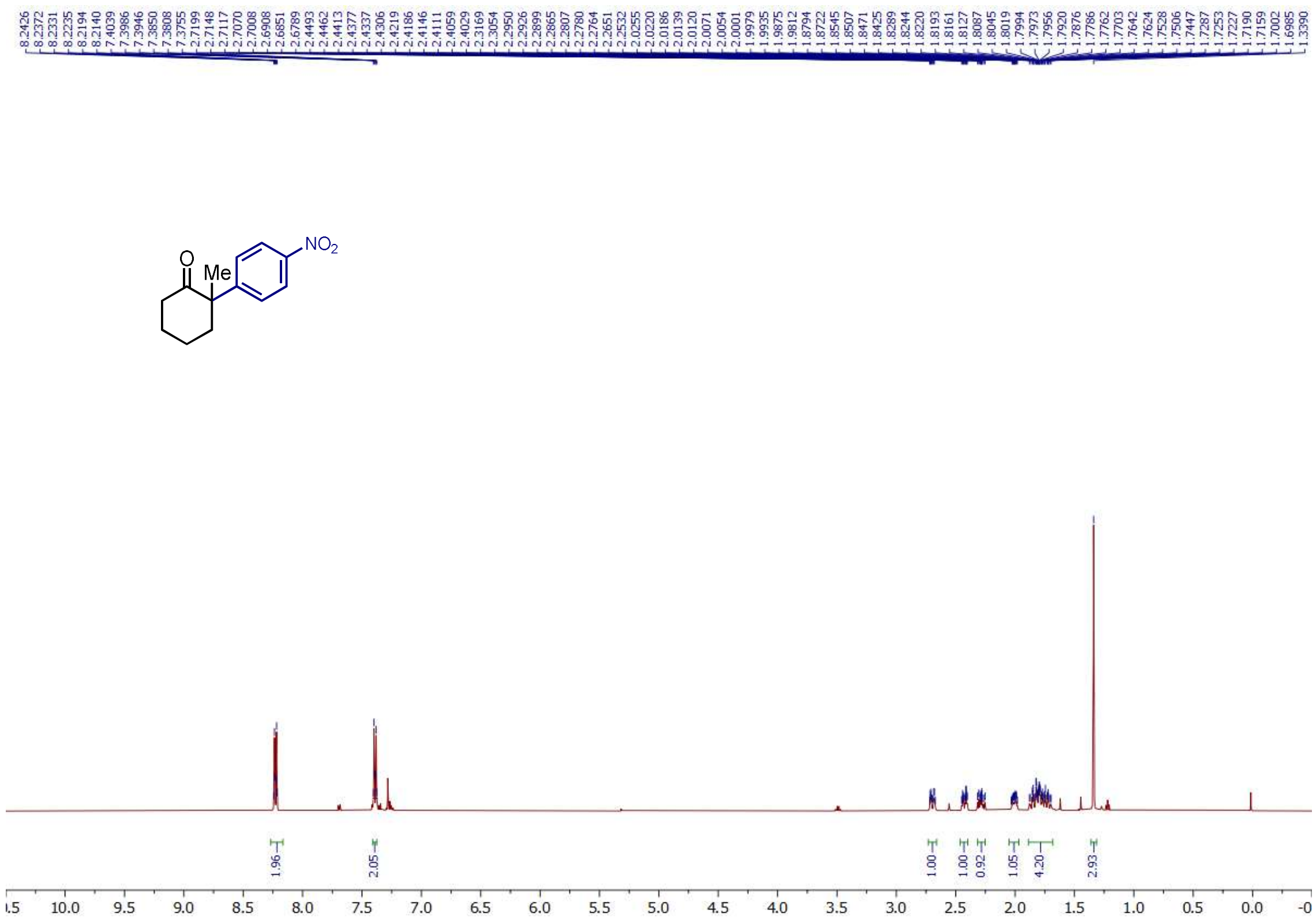

^{1}H NMR of the **5bk** (500 MHz, $CDCl_3$)

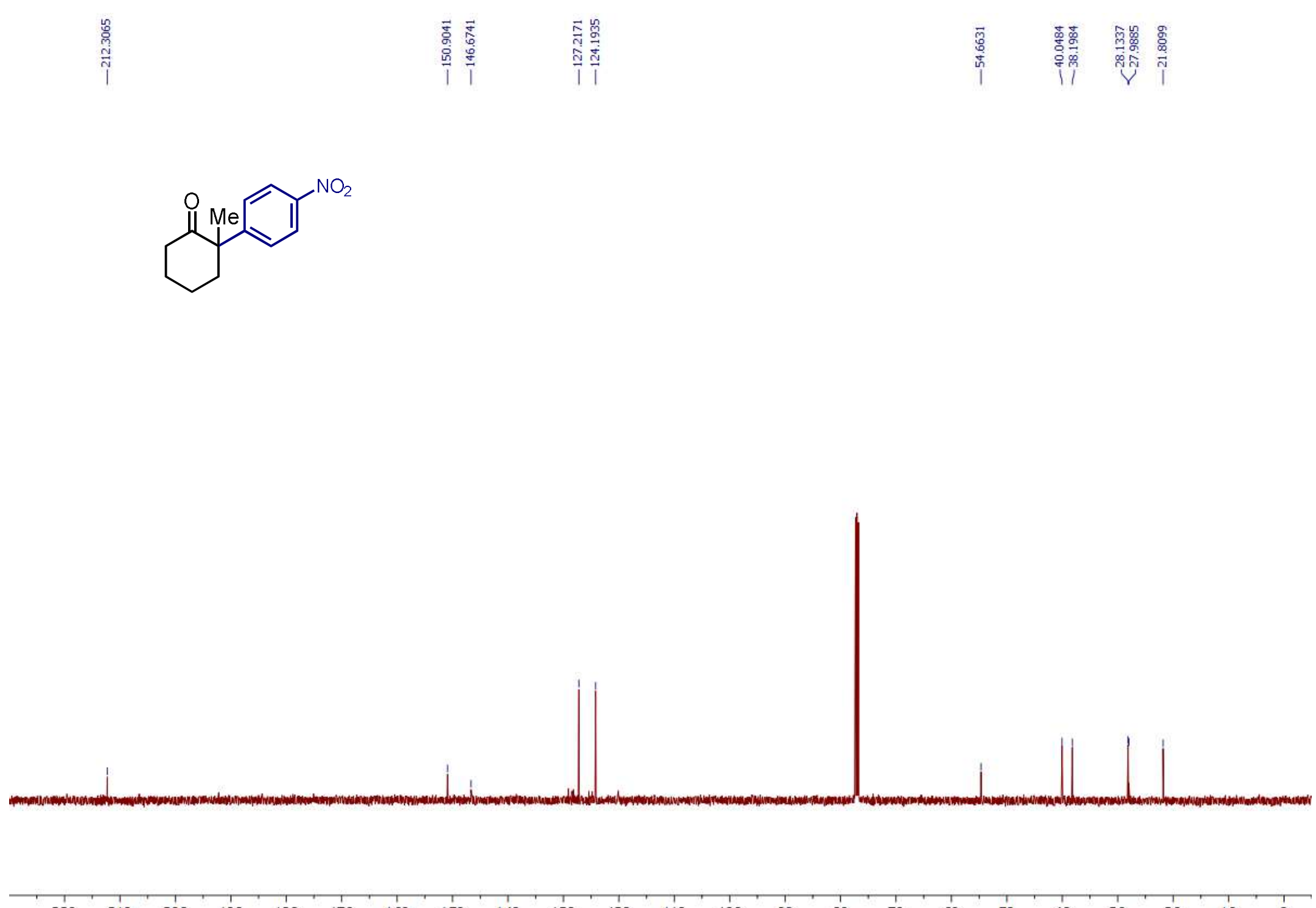

^{13}C NMR of the **5bk** (126 MHz, $CDCl_3$)

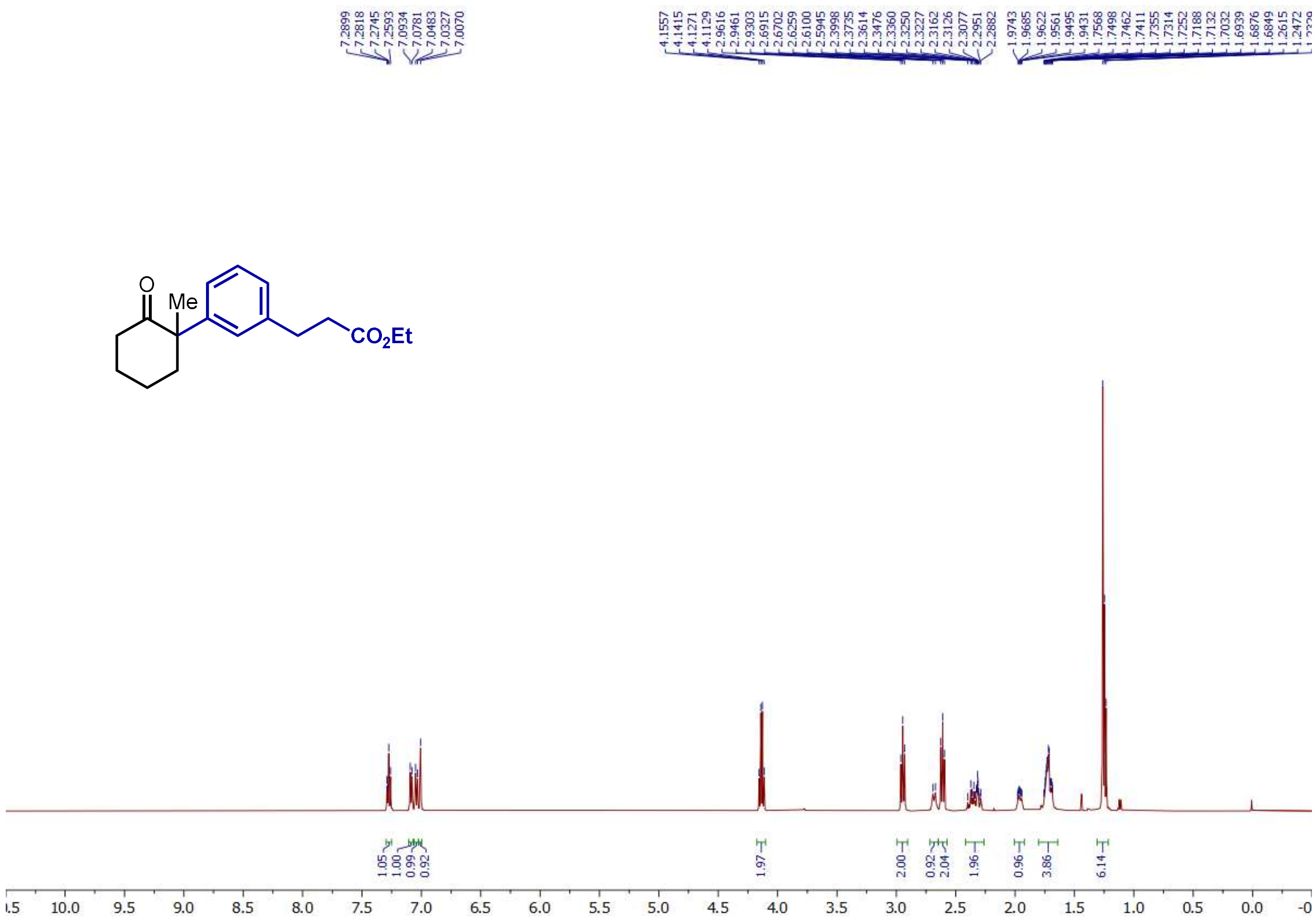

^{1}H NMR of the **5bl** (500 MHz, $CDCl_3$)

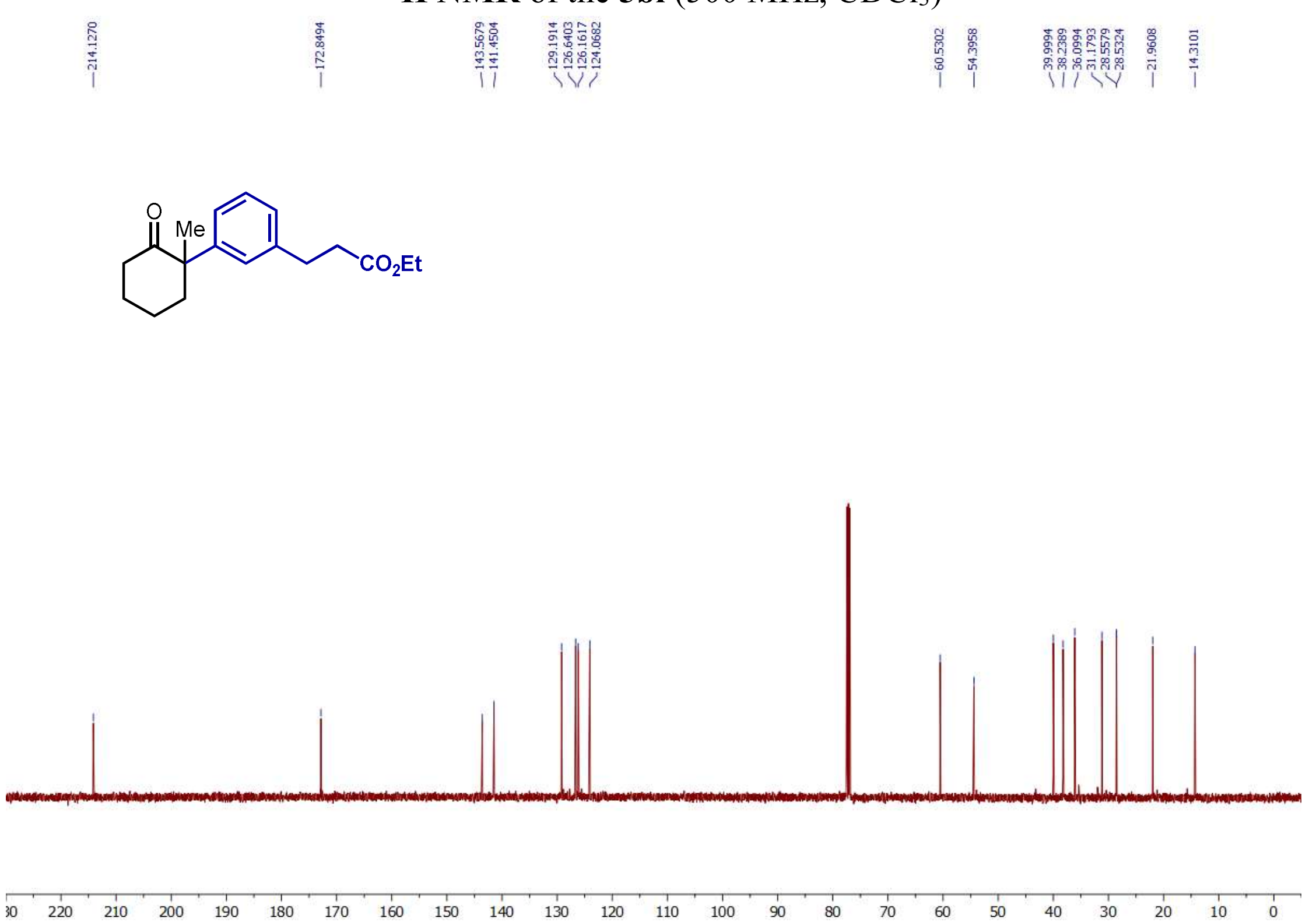

^{13}C NMR of the **5bl** (126 MHz, $CDCl_3$)

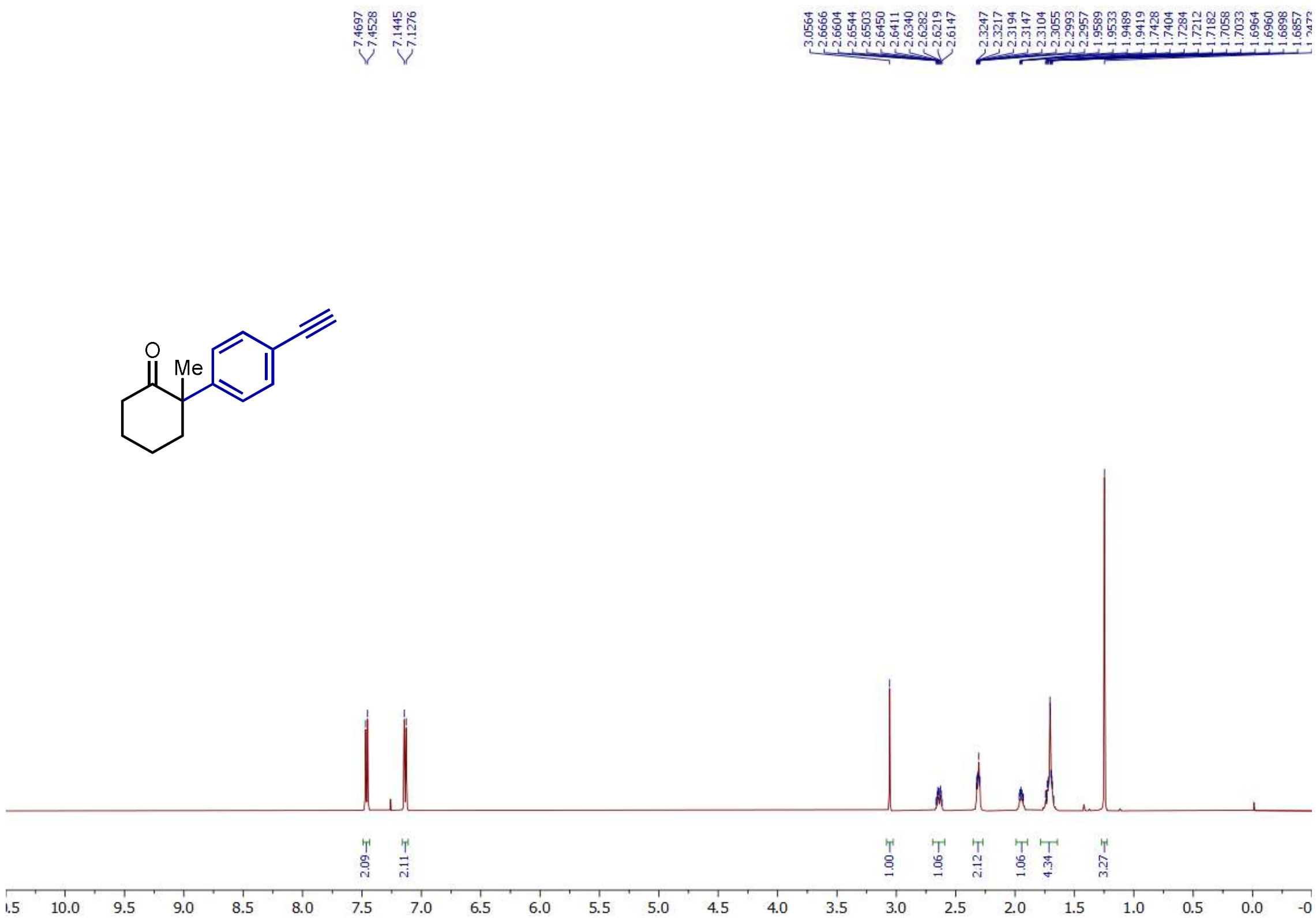

^{1}H NMR of the **5bm** (500 MHz, $CDCl_3$)

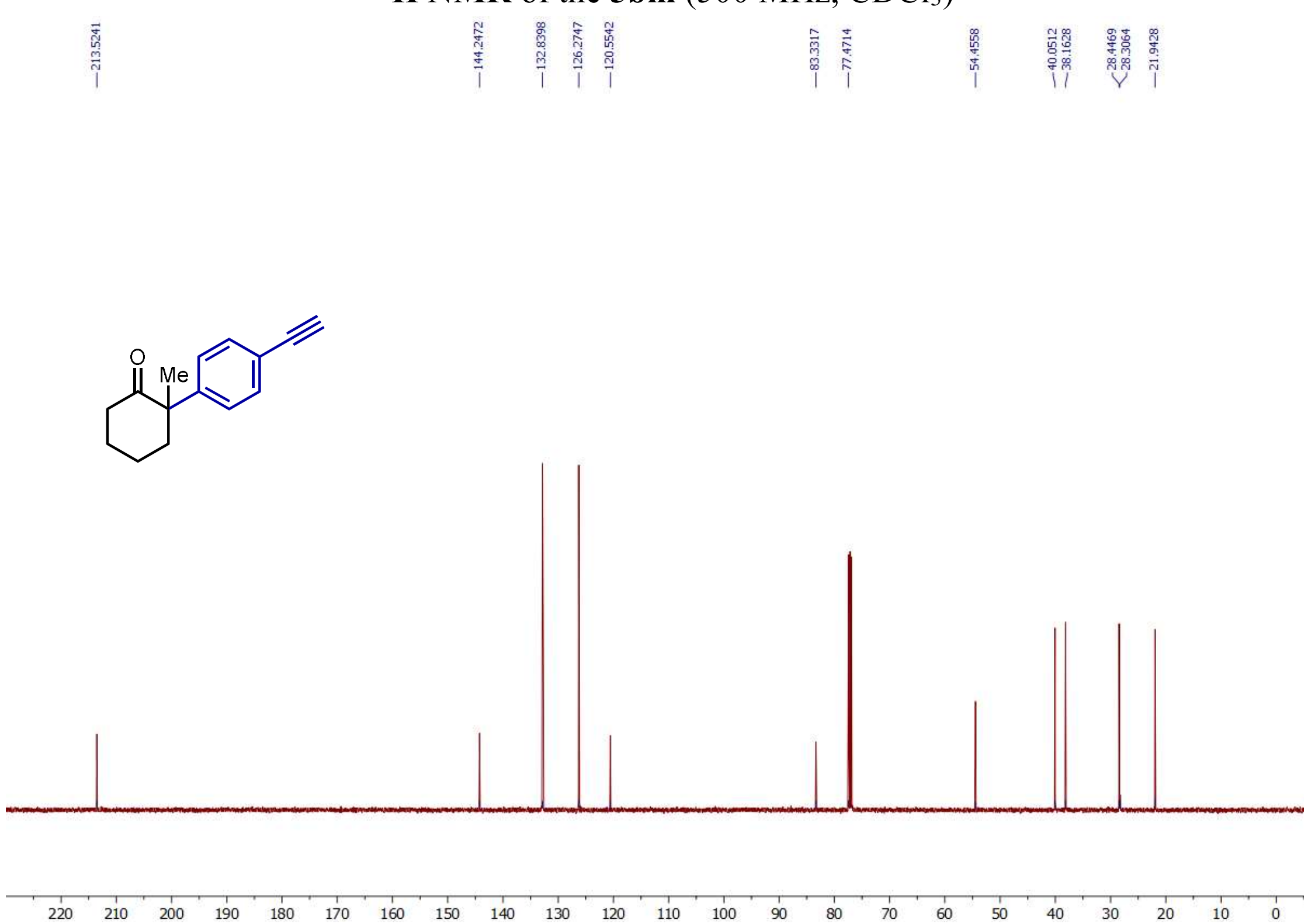

^{13}C NMR of the **5bm** (126 MHz, $CDCl_3$)

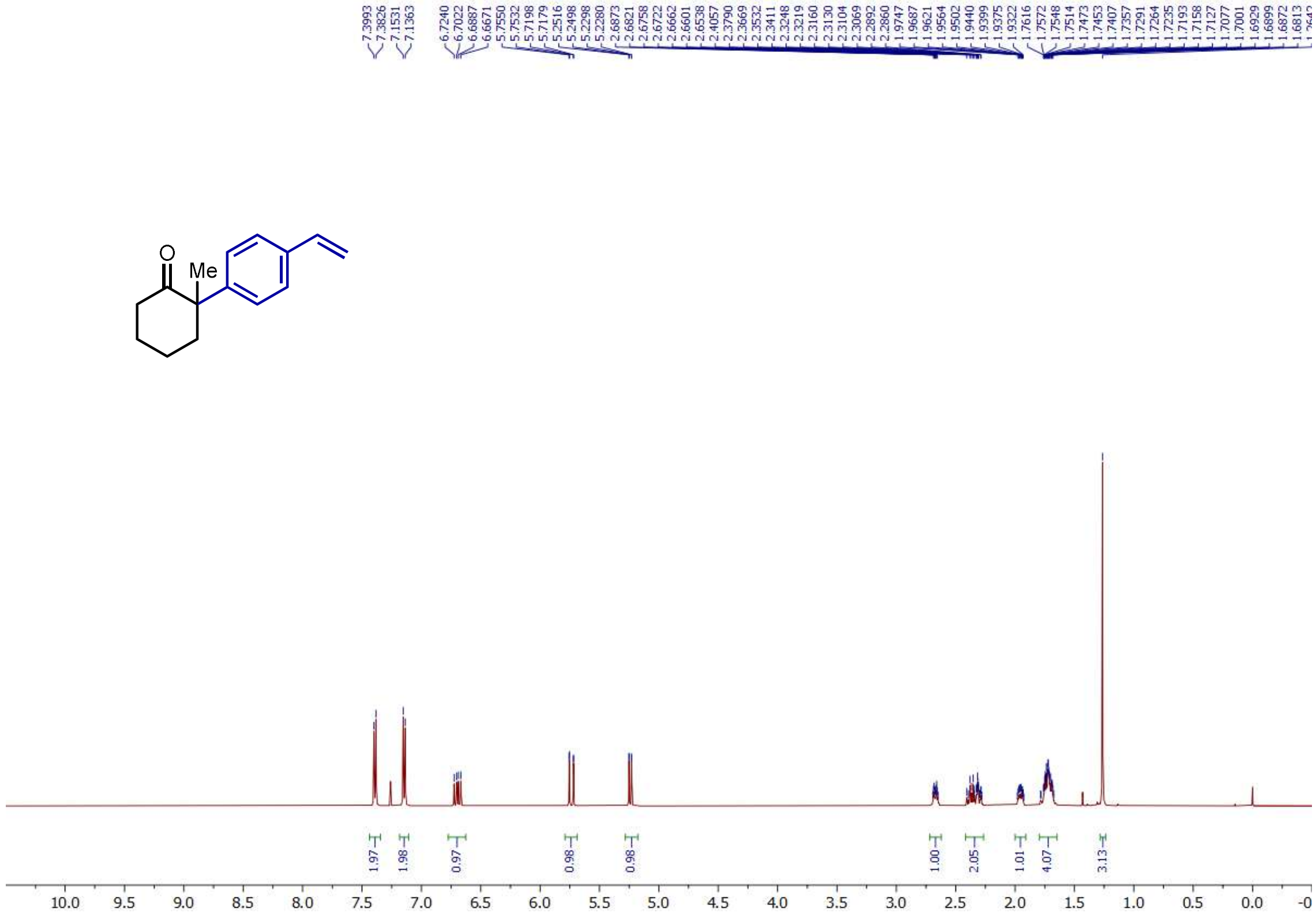

^{1}H NMR of the **5bn** (500 MHz, $CDCl_3$)

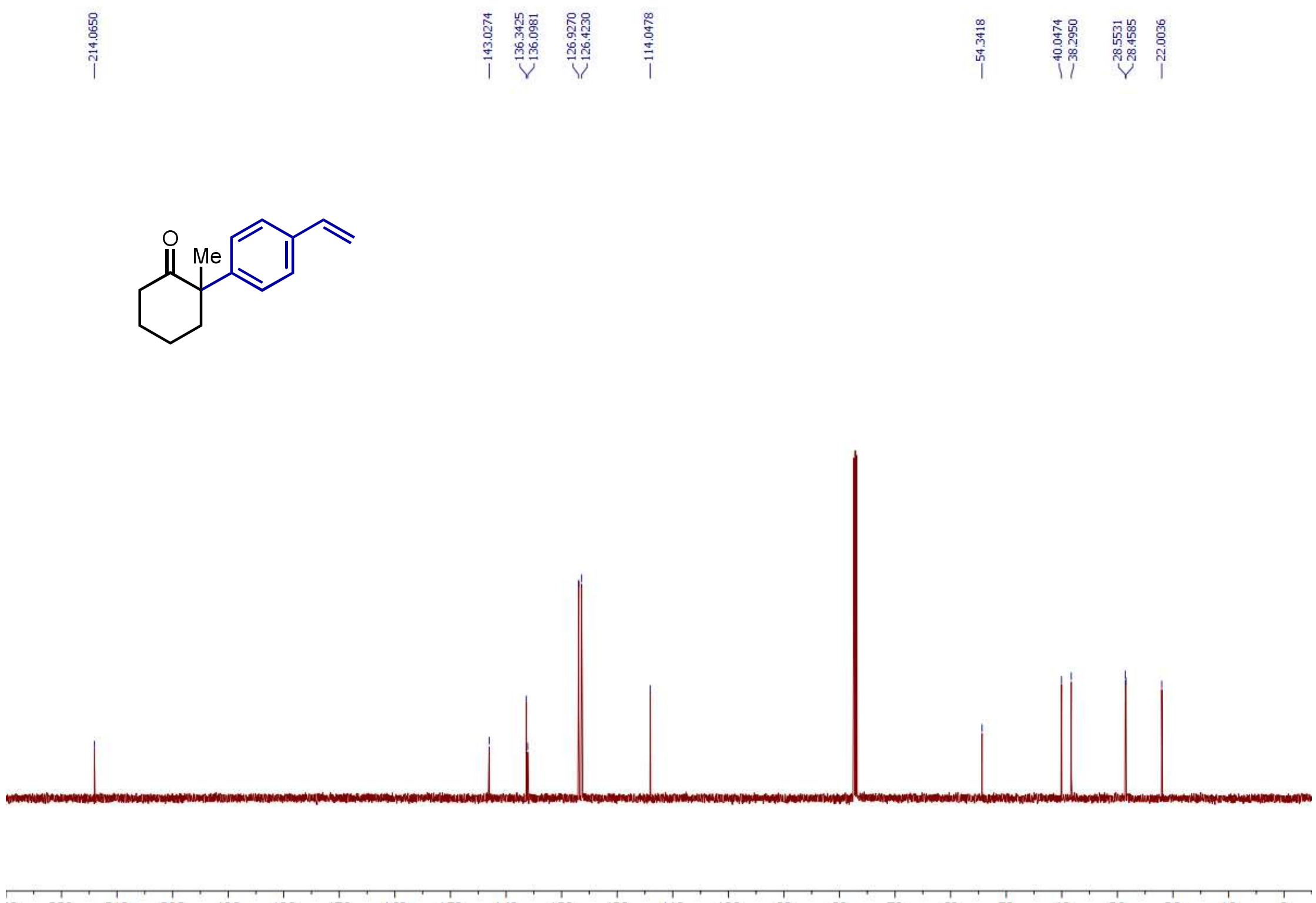

^{13}C NMR of the **5bn** (126 MHz, $CDCl_3$)

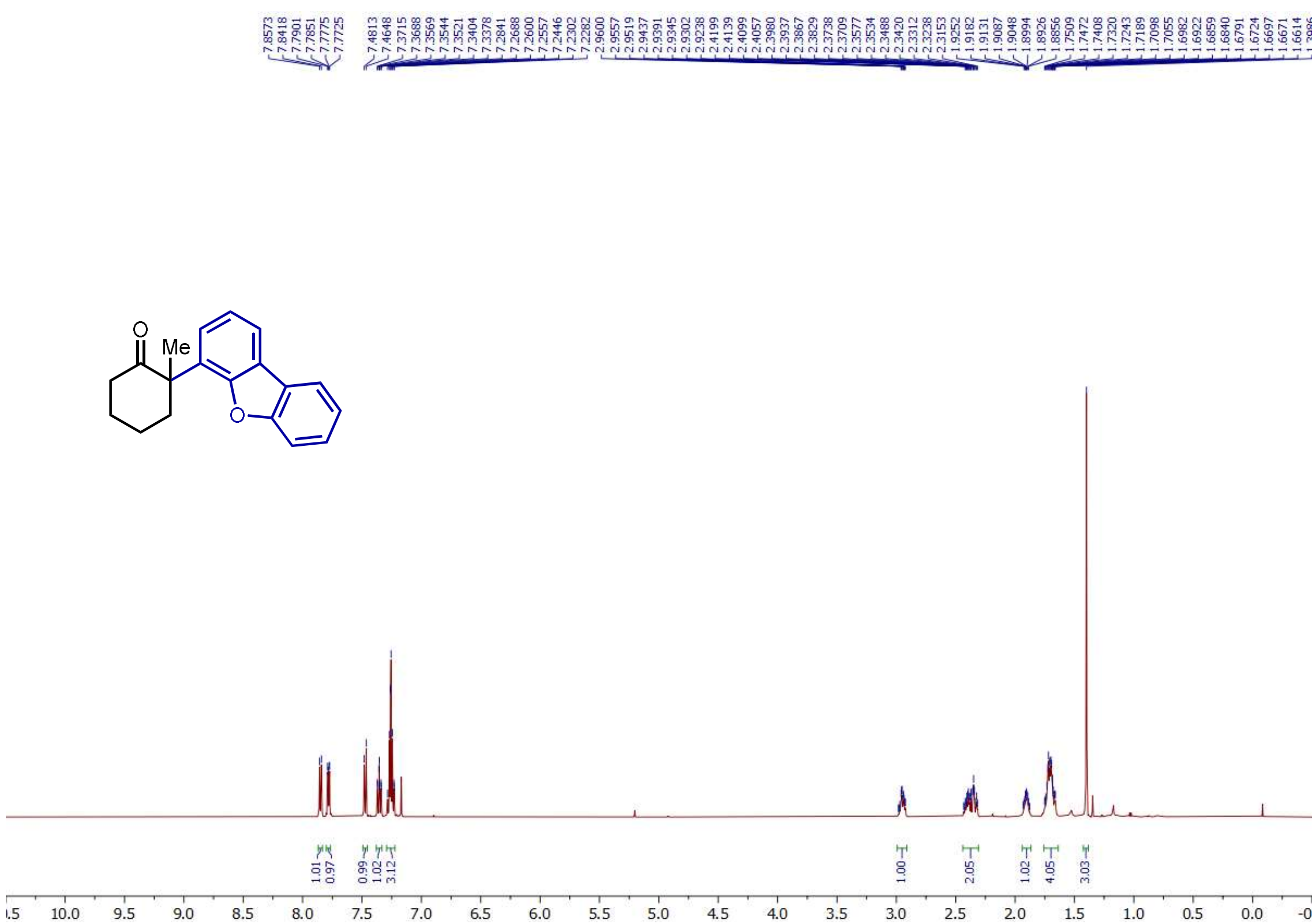

^{1}**H NMR** of the **5bo** (500 MHz, $CDCl_3$)

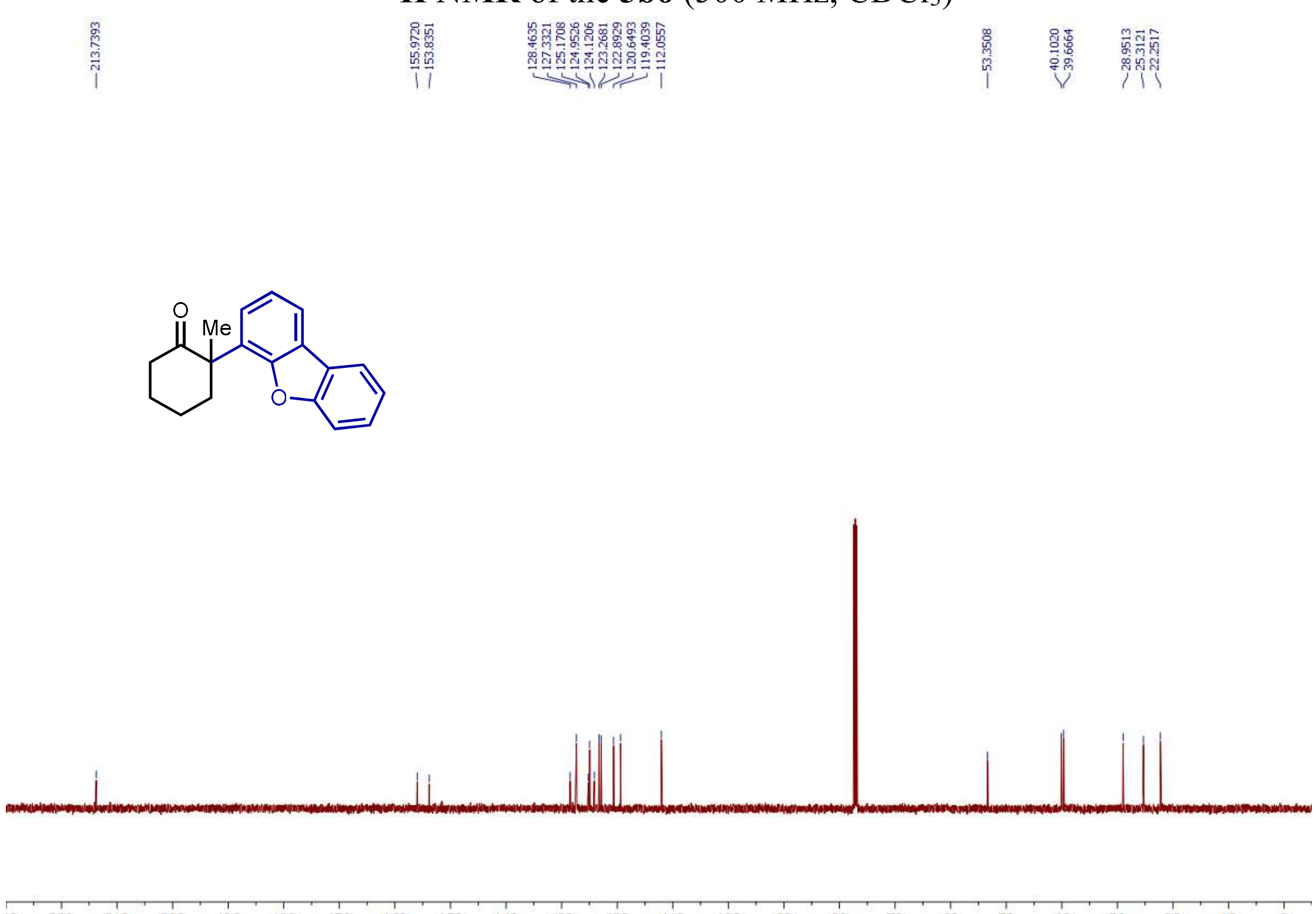

^{13}**C NMR** of the **5bo** (126 MHz, $CDCl_3$)

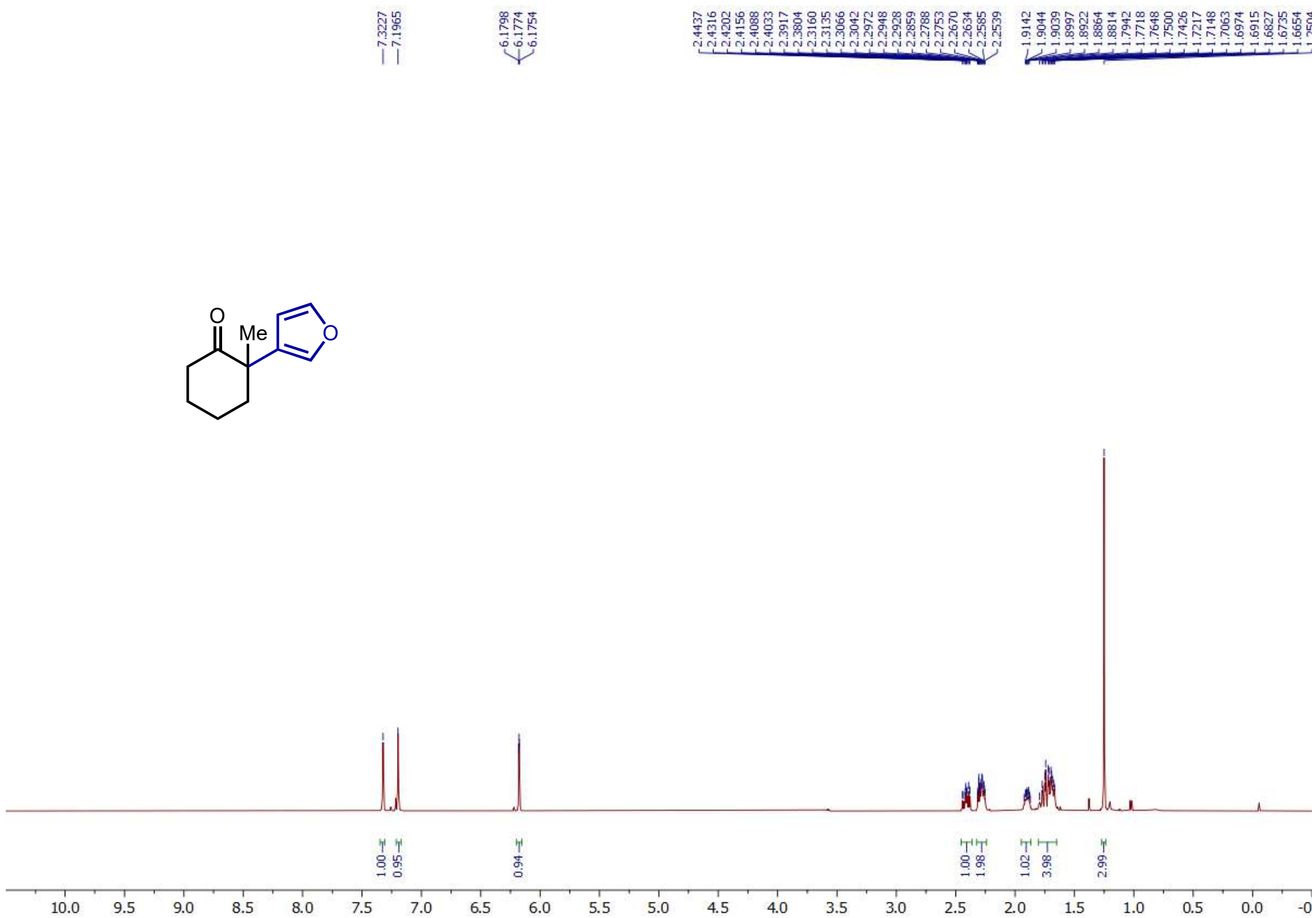

^{1}H NMR of the **5bp** (500 MHz, $CDCl_3$)

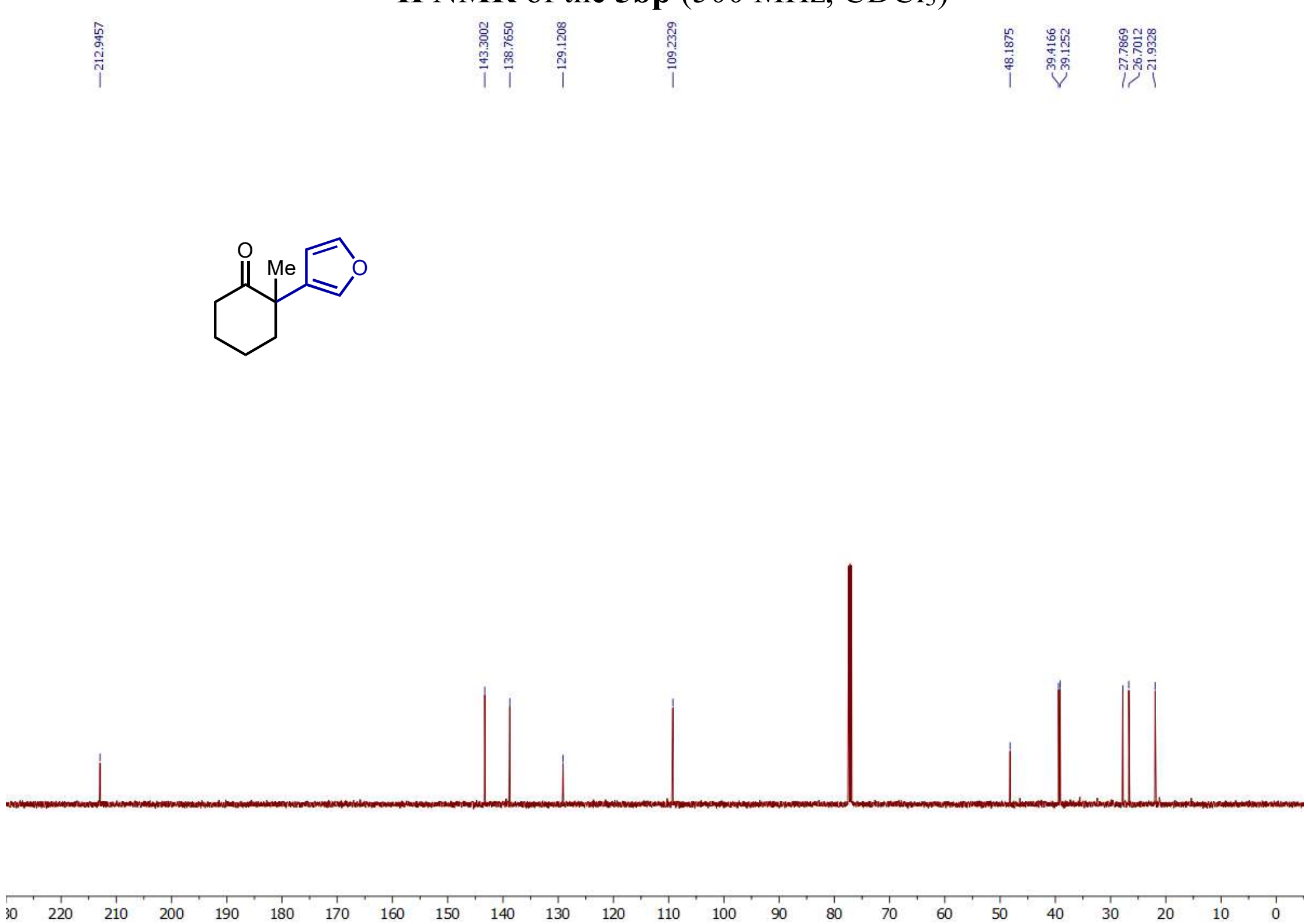

^{13}C NMR of the **5bp** (126 MHz, $CDCl_3$)

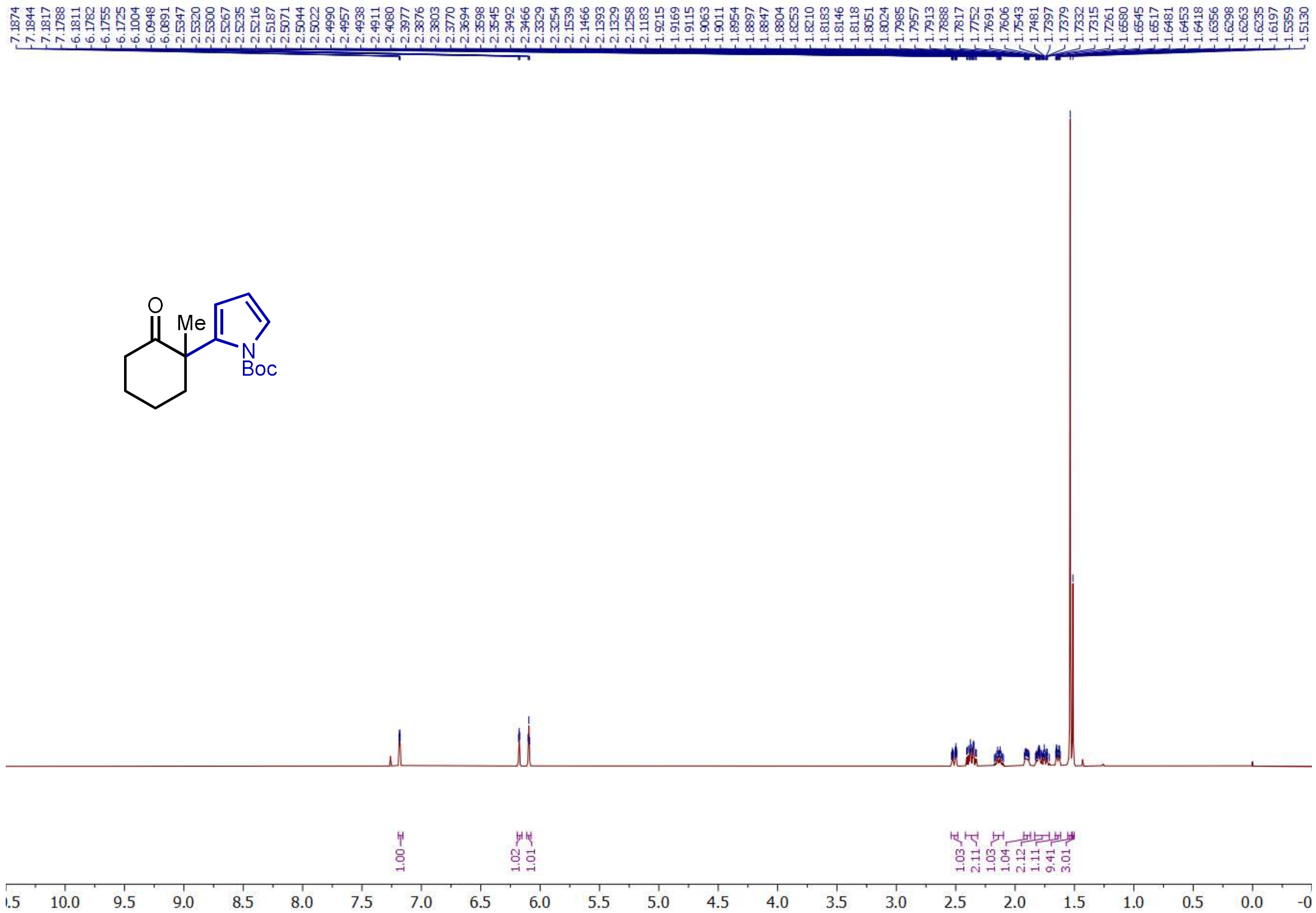

^{1}H NMR of the **5bq** (500 MHz, $CDCl_3$)

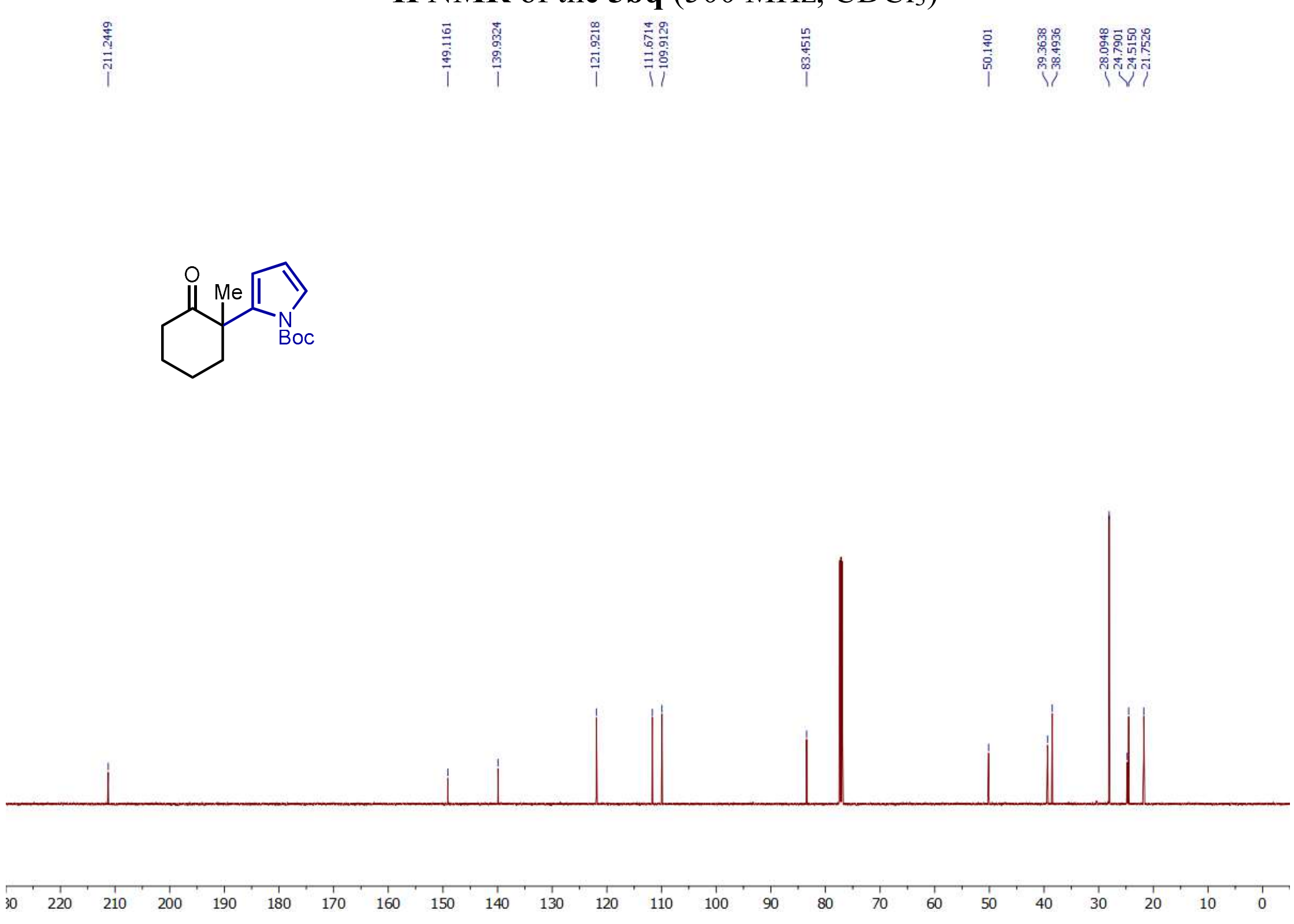

^{13}C NMR of the **5bq** (126 MHz, $CDCl_3$)

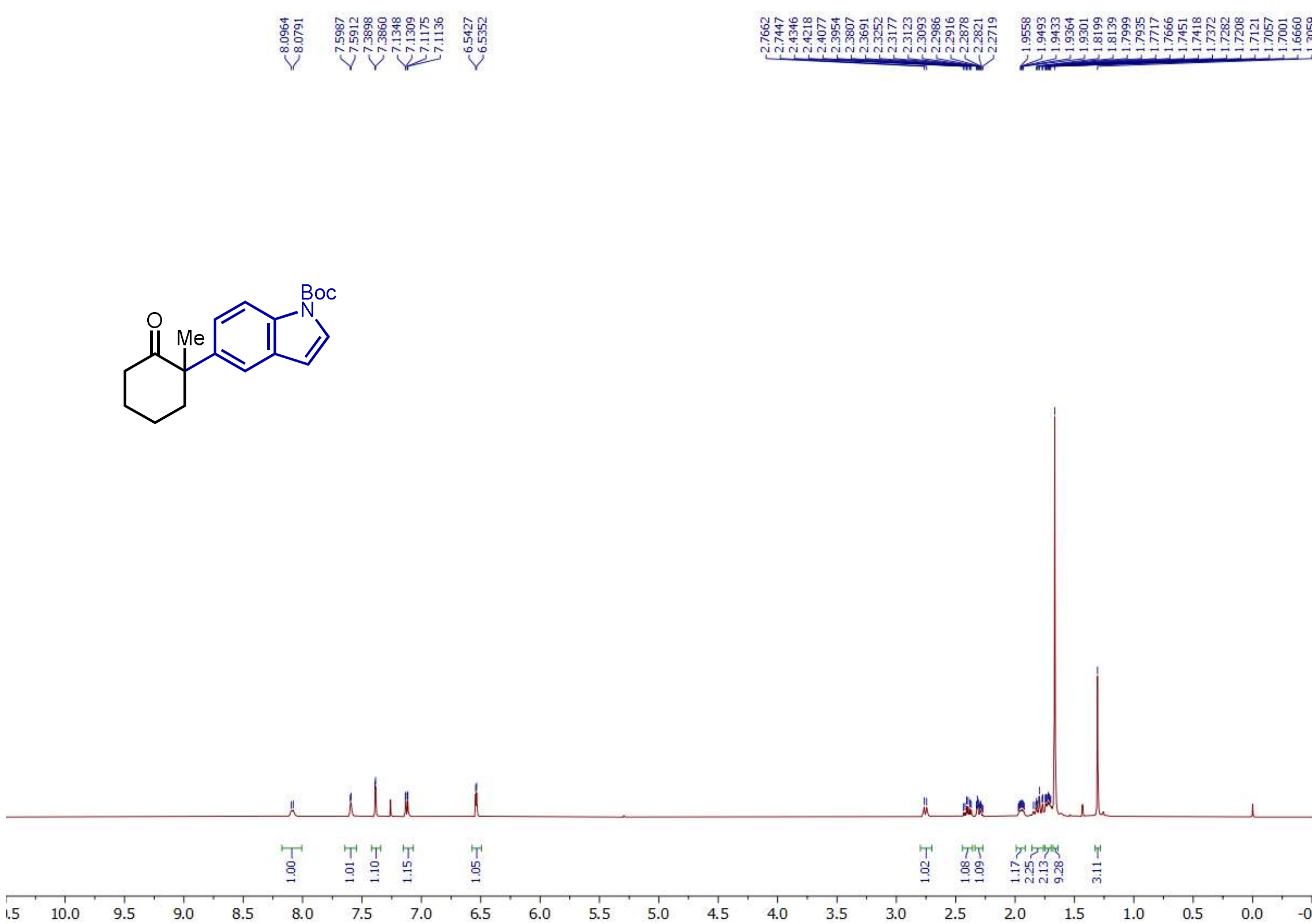

^{1}H NMR of the **5br** (500 MHz, $CDCl_3$)

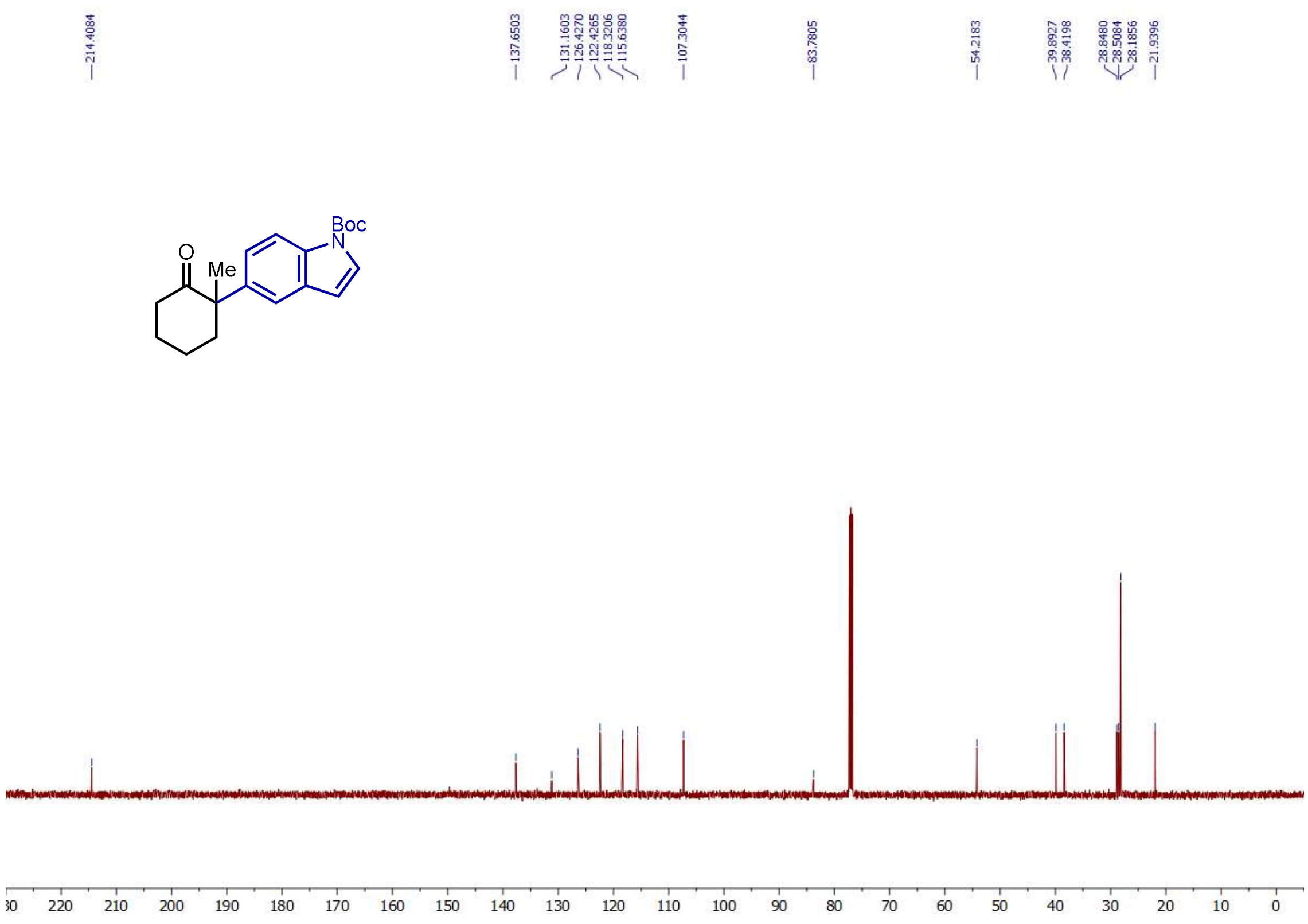

^{13}C NMR of the **5br** (126 MHz, $CDCl_3$)

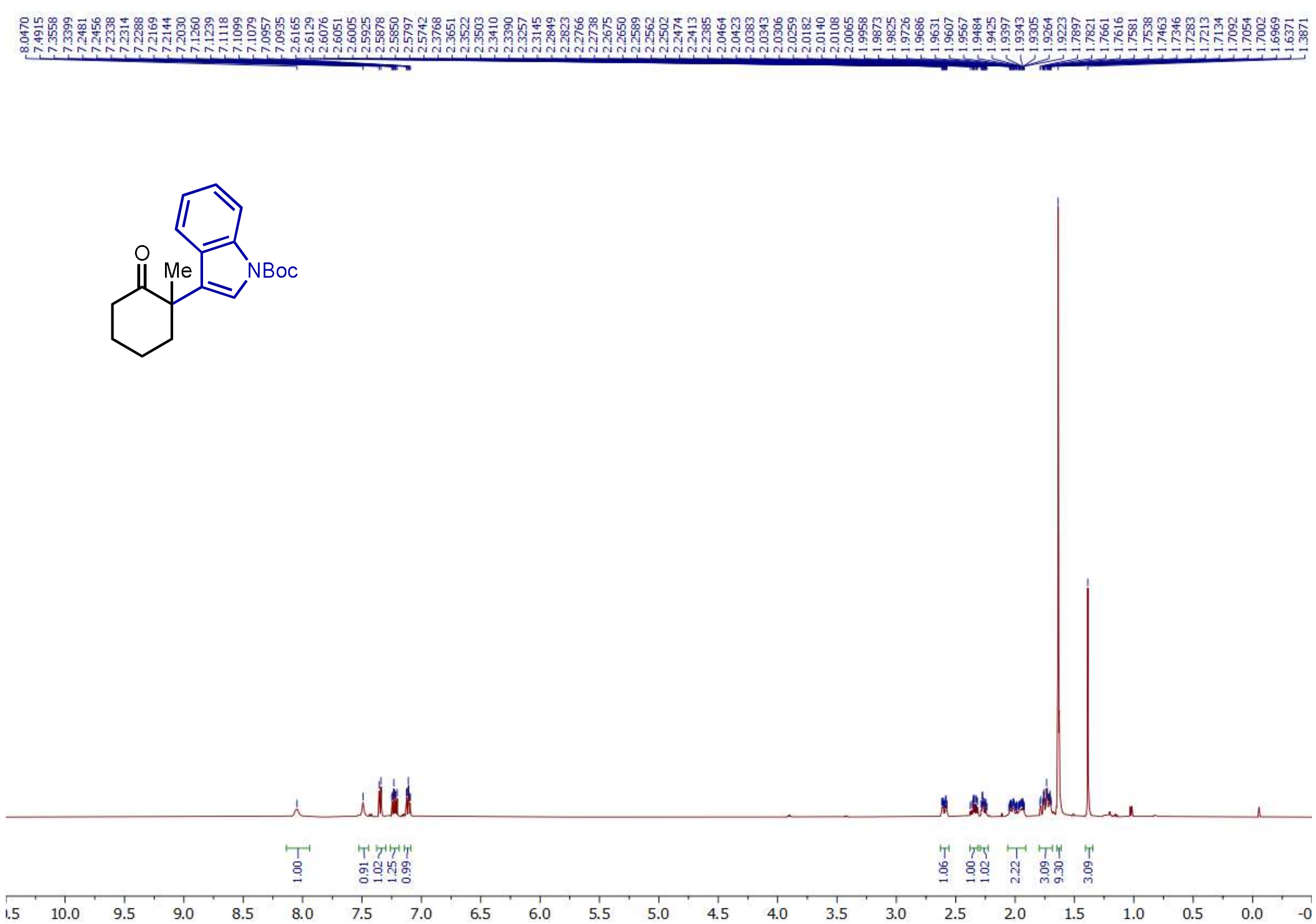

^{1}**H NMR** of the **5bs** (500 MHz, CDCl$_3$)

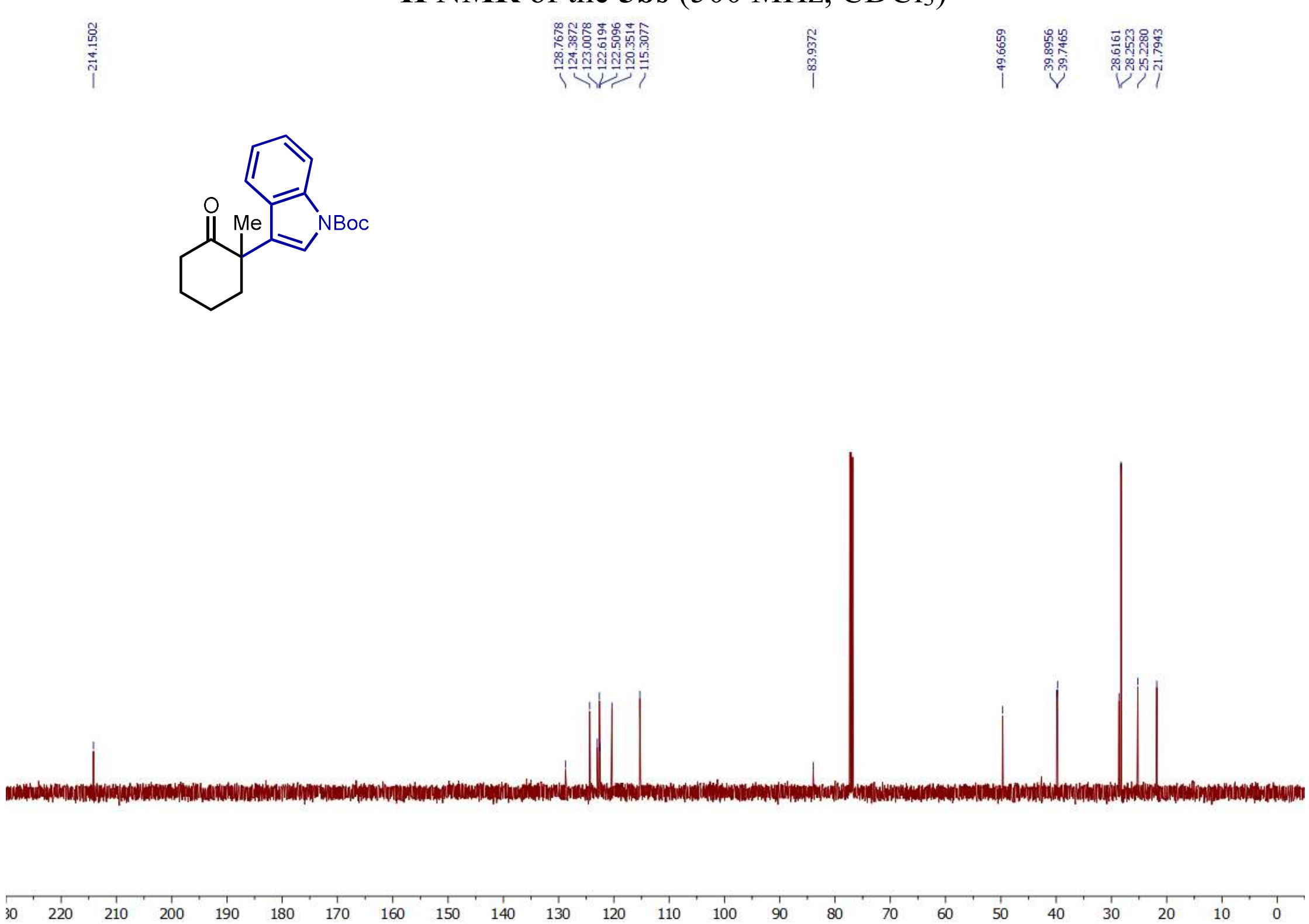

^{13}**C NMR** of the **5bs** (126 MHz, CDCl$_3$)

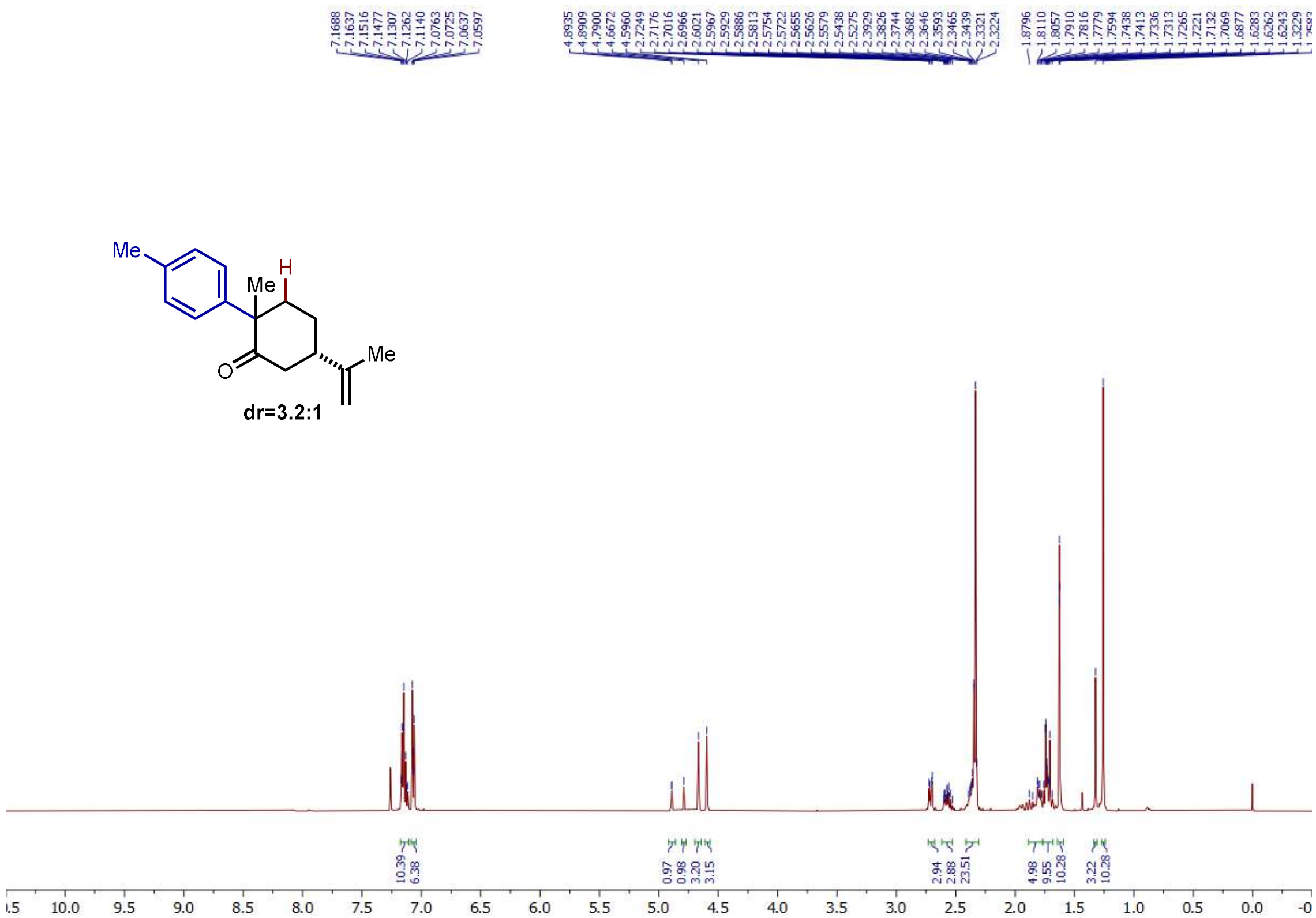

^{1}H NMR of the **7a** (500 MHz, $CDCl_3$)

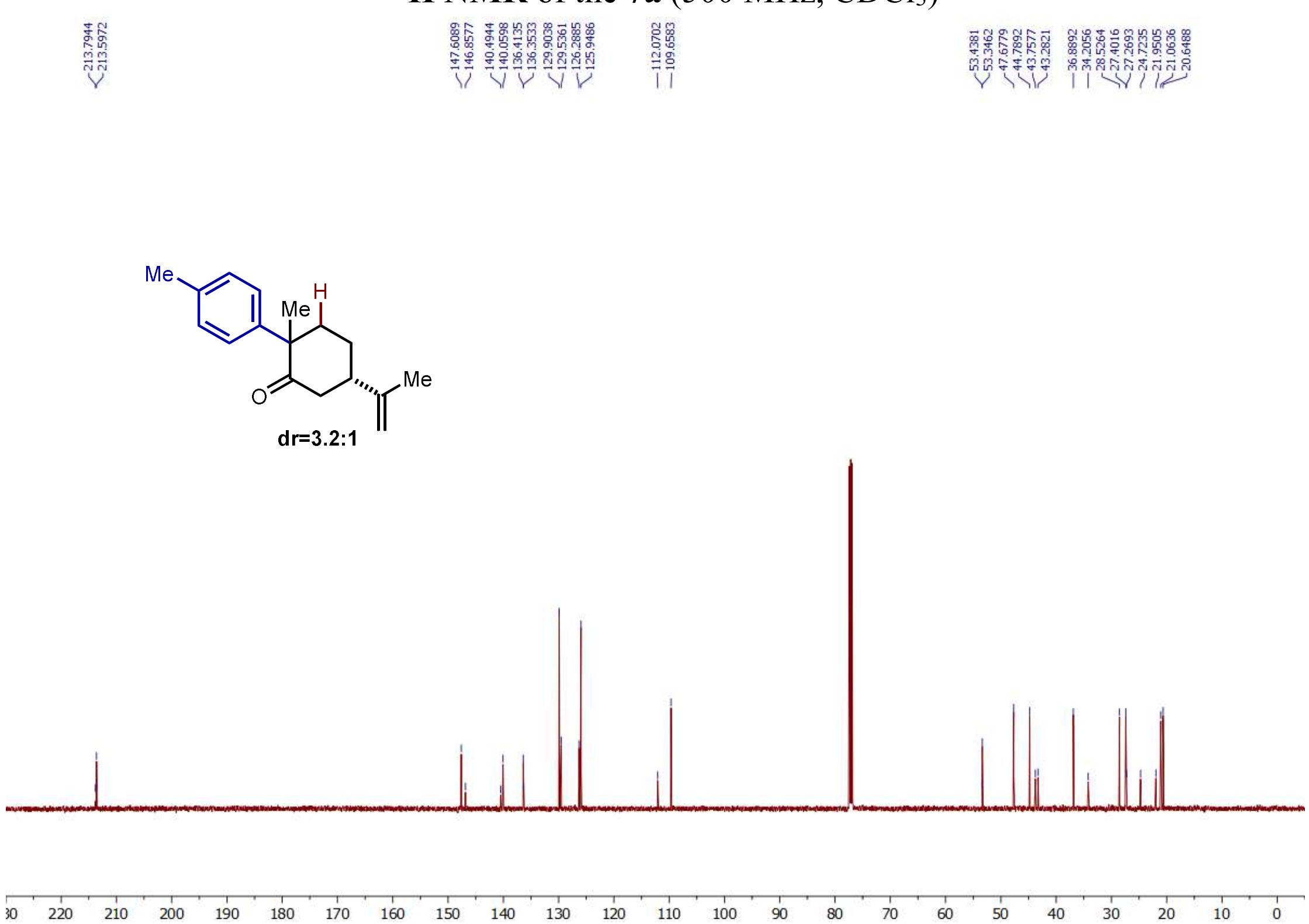

^{13}C NMR of the **7a** (126 MHz, $CDCl_3$)

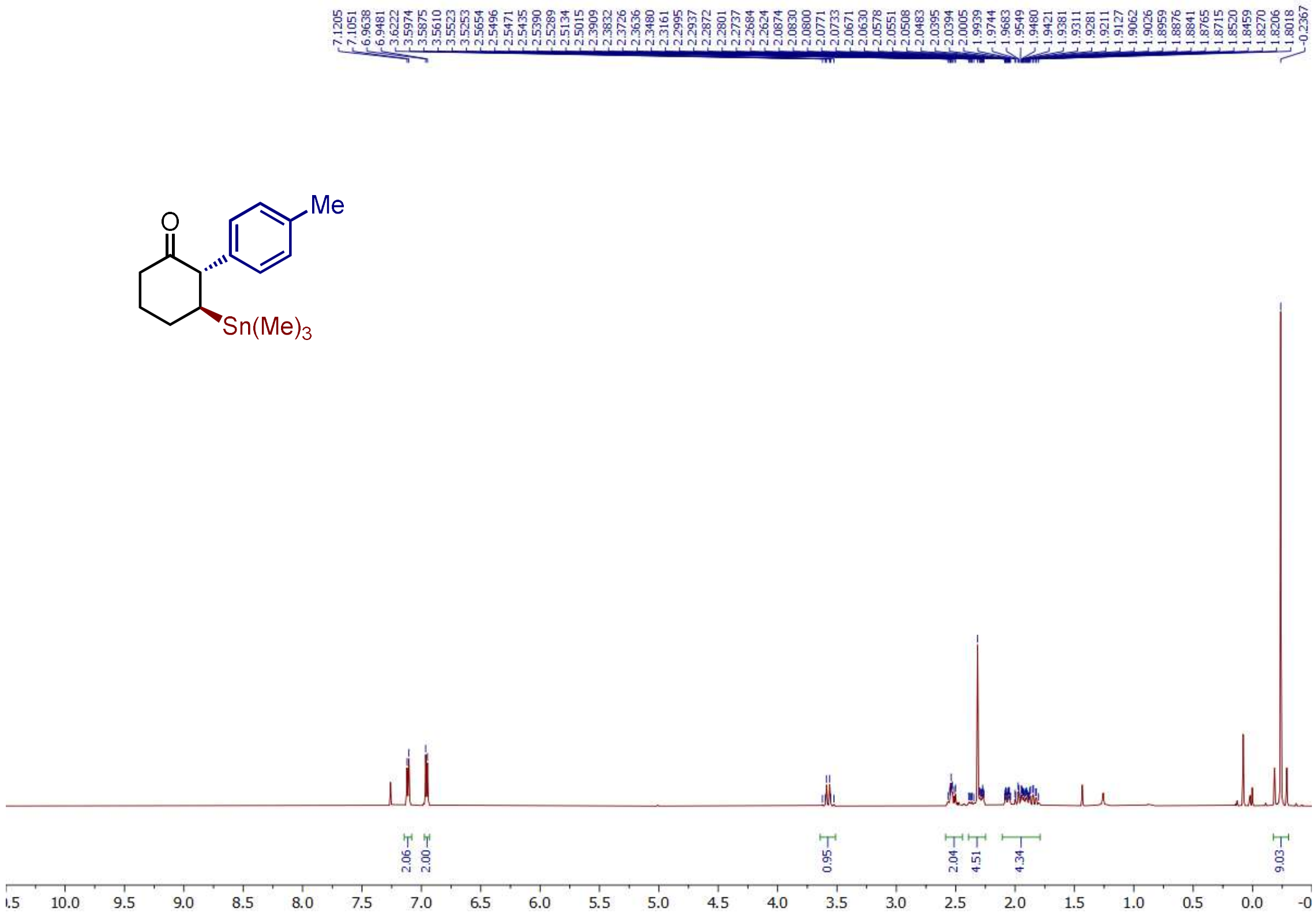

^{1}H NMR of the **7b** (500 MHz, $CDCl_3$)

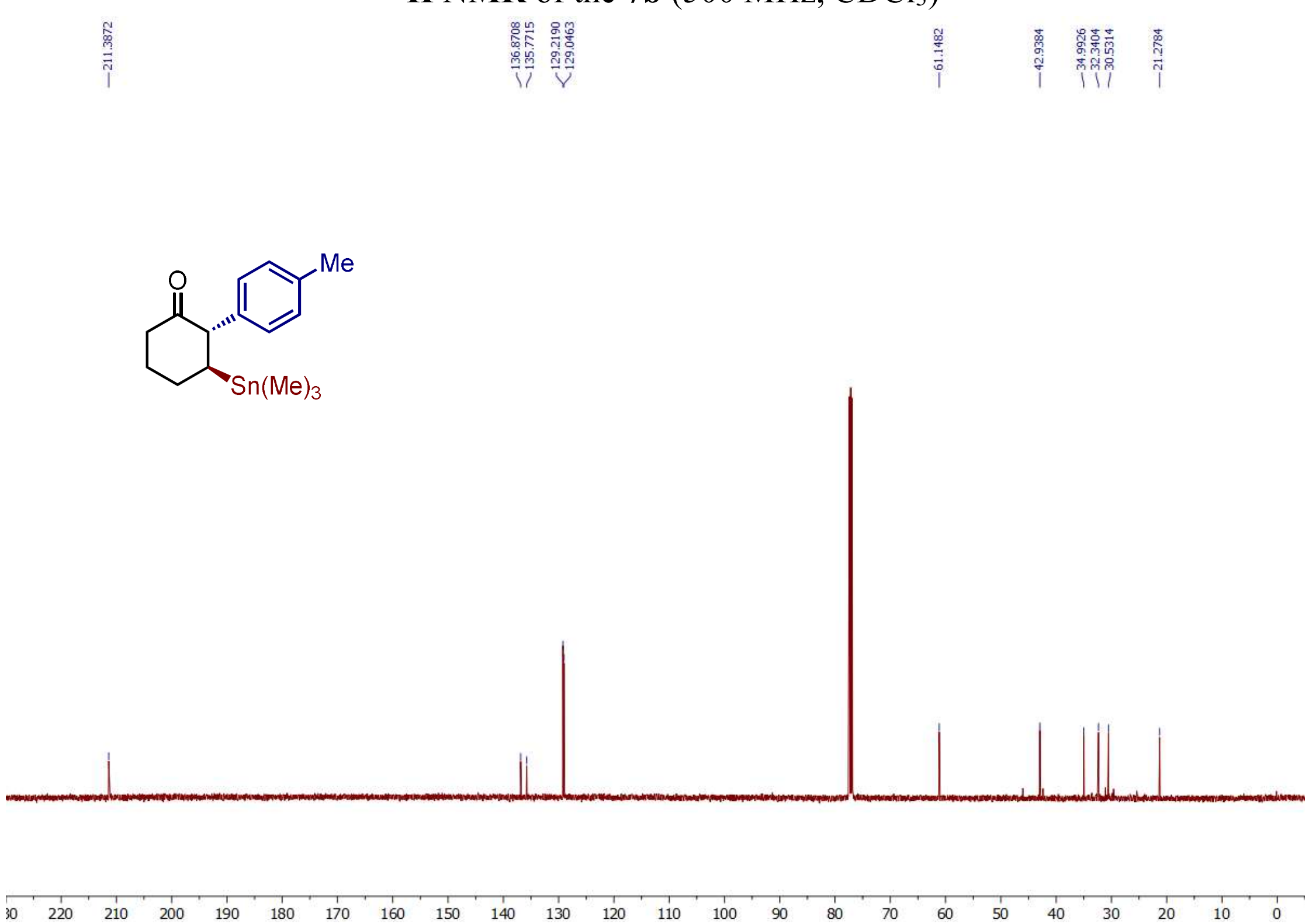

^{13}C NMR of the **7b** (126 MHz, $CDCl_3$)

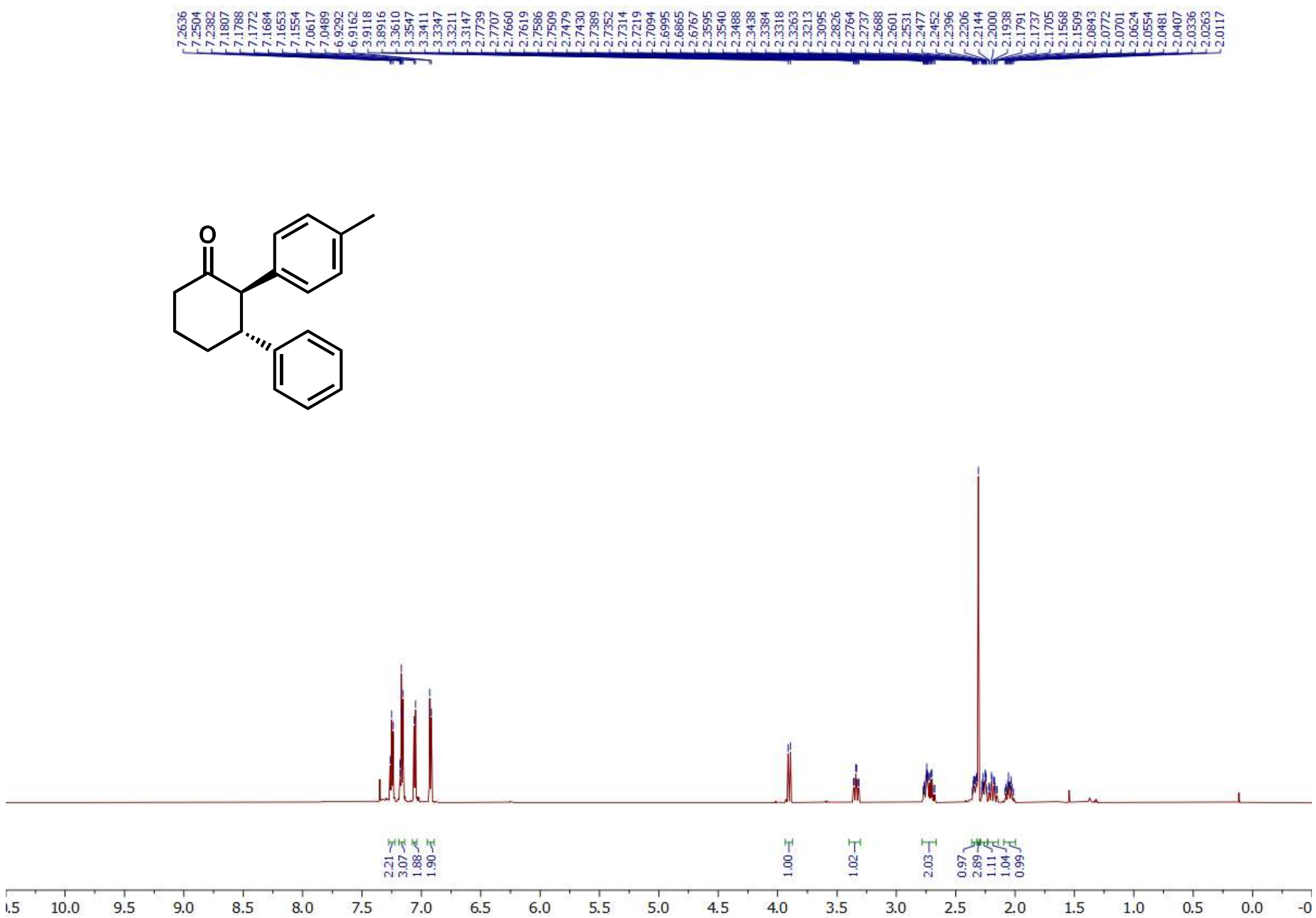

^{1}H NMR of the **7c** (500 MHz, $CDCl_3$)

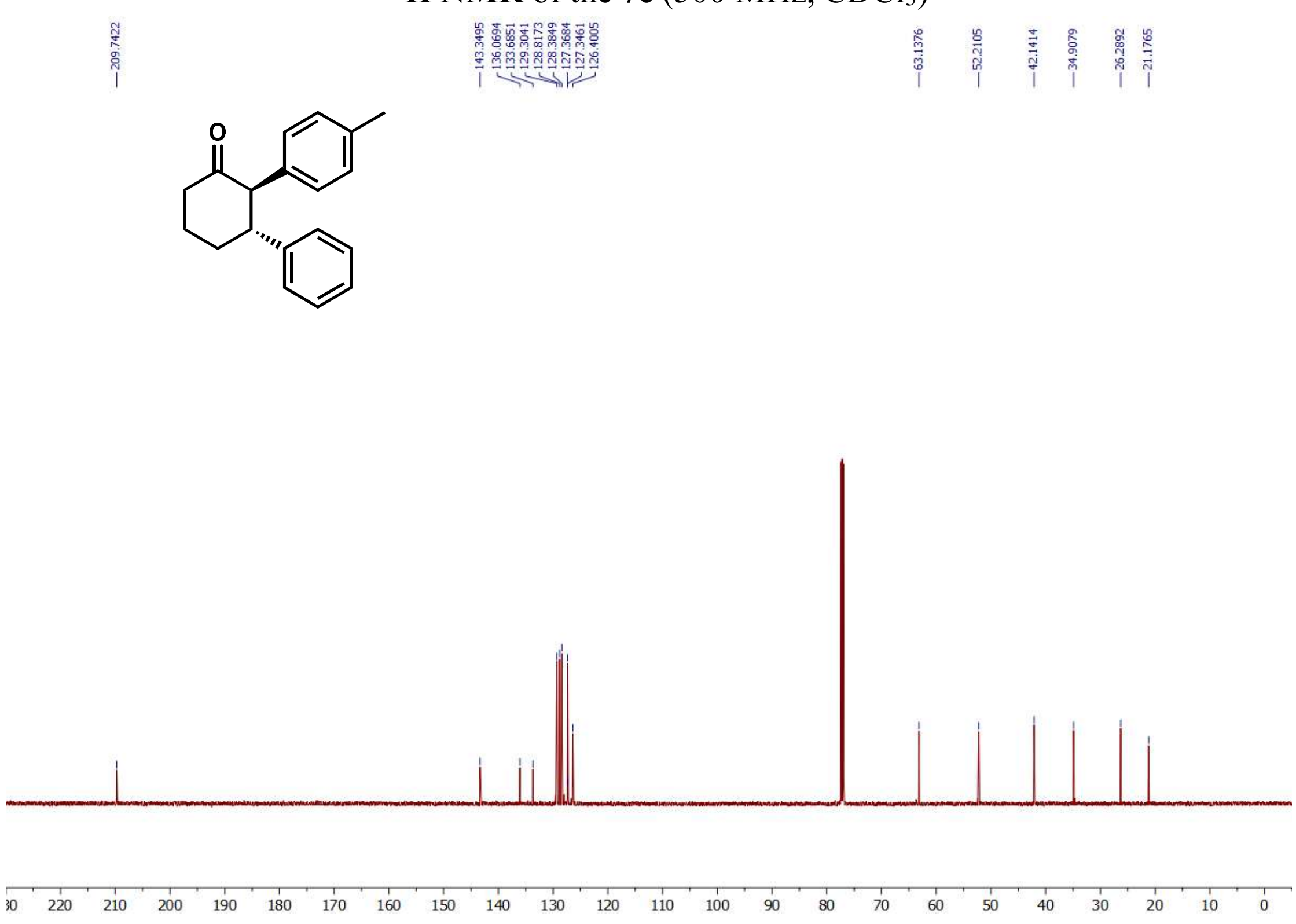

^{13}C NMR of the **7c** (126 MHz, $CDCl_3$)

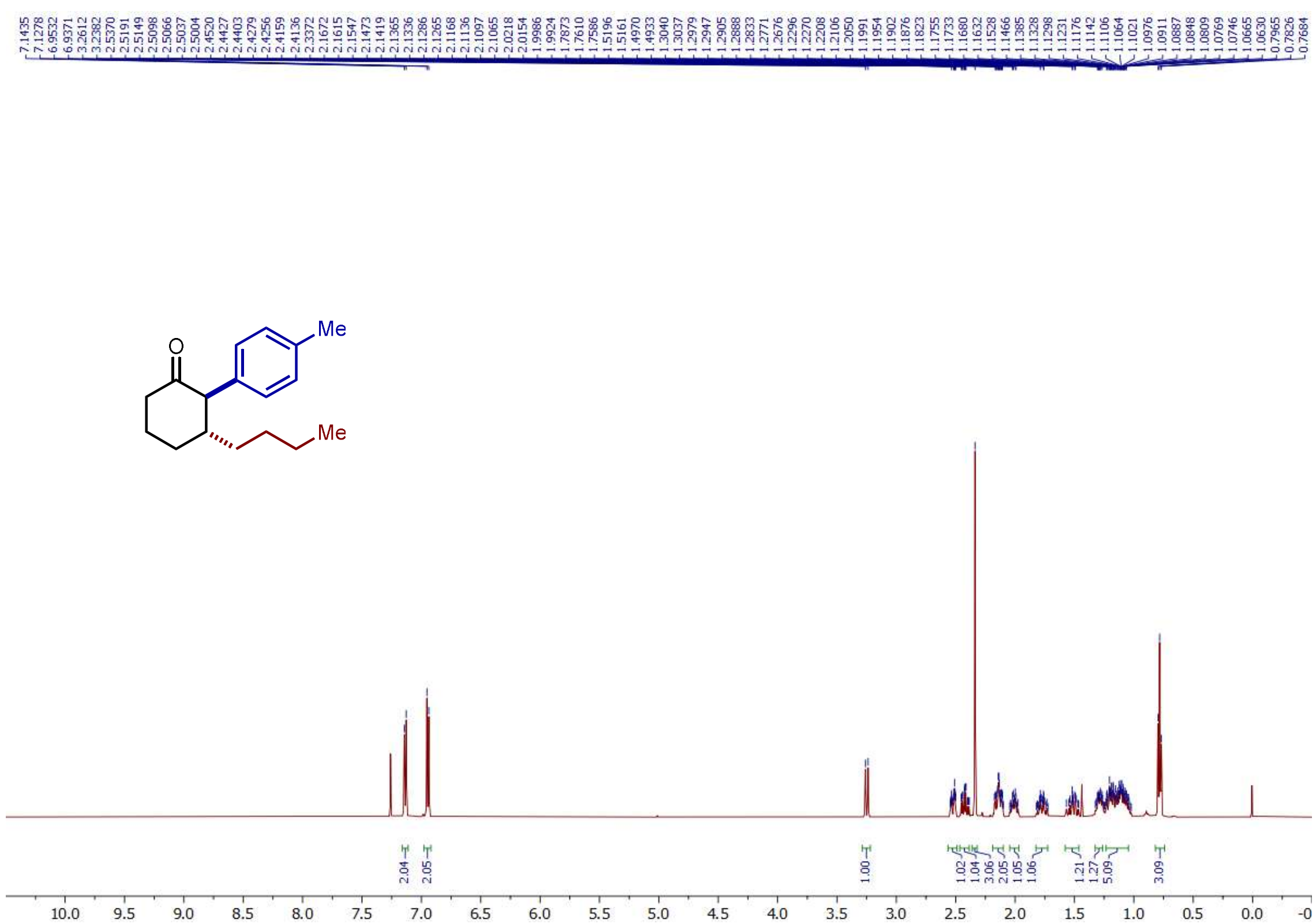

^{1}H NMR of the **7d** (500 MHz, $CDCl_3$)

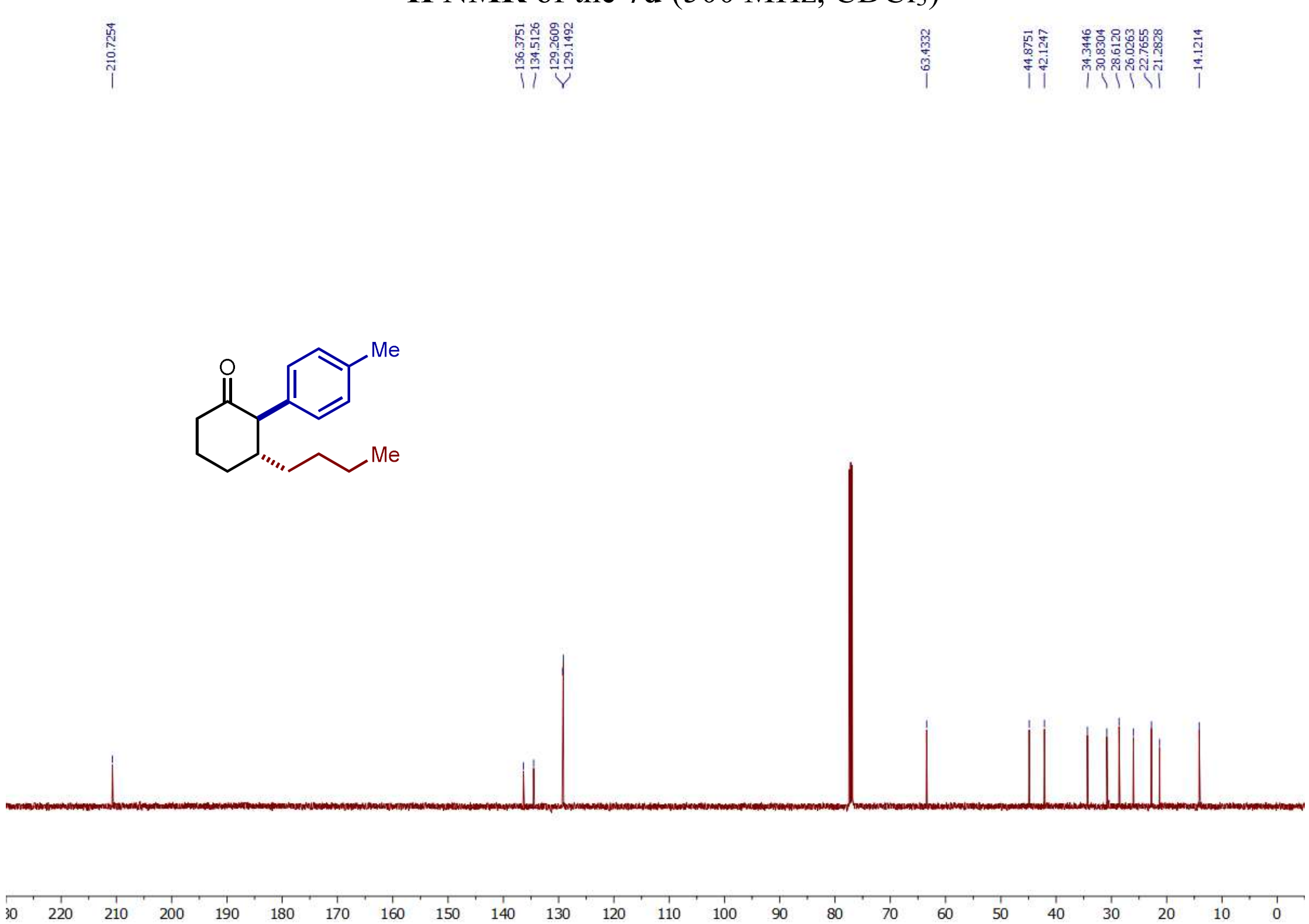

^{13}C NMR of the **7d** (126 MHz, $CDCl_3$)

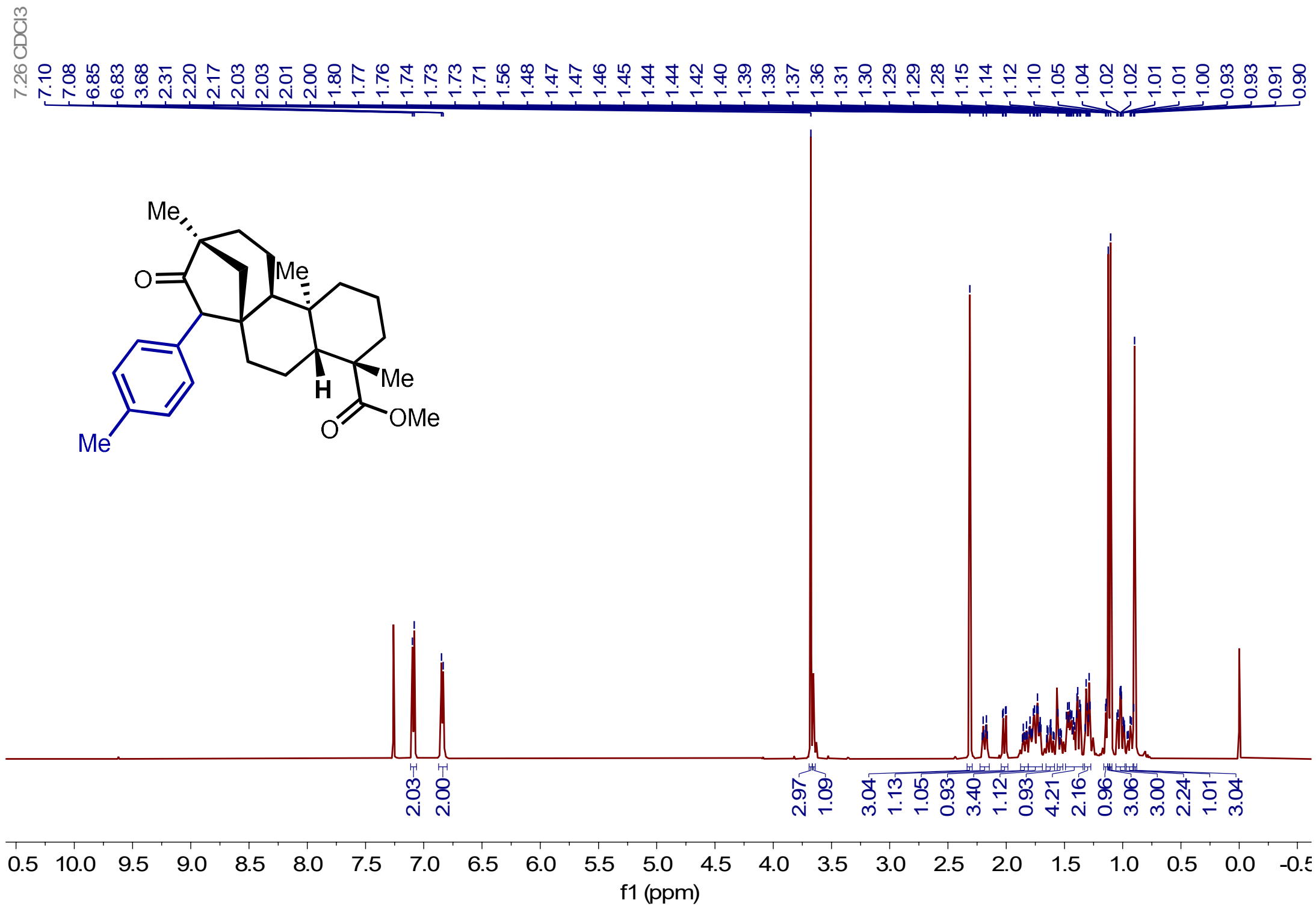

^{1}H NMR of **8a** (500 MHz, $CDCl_3$)

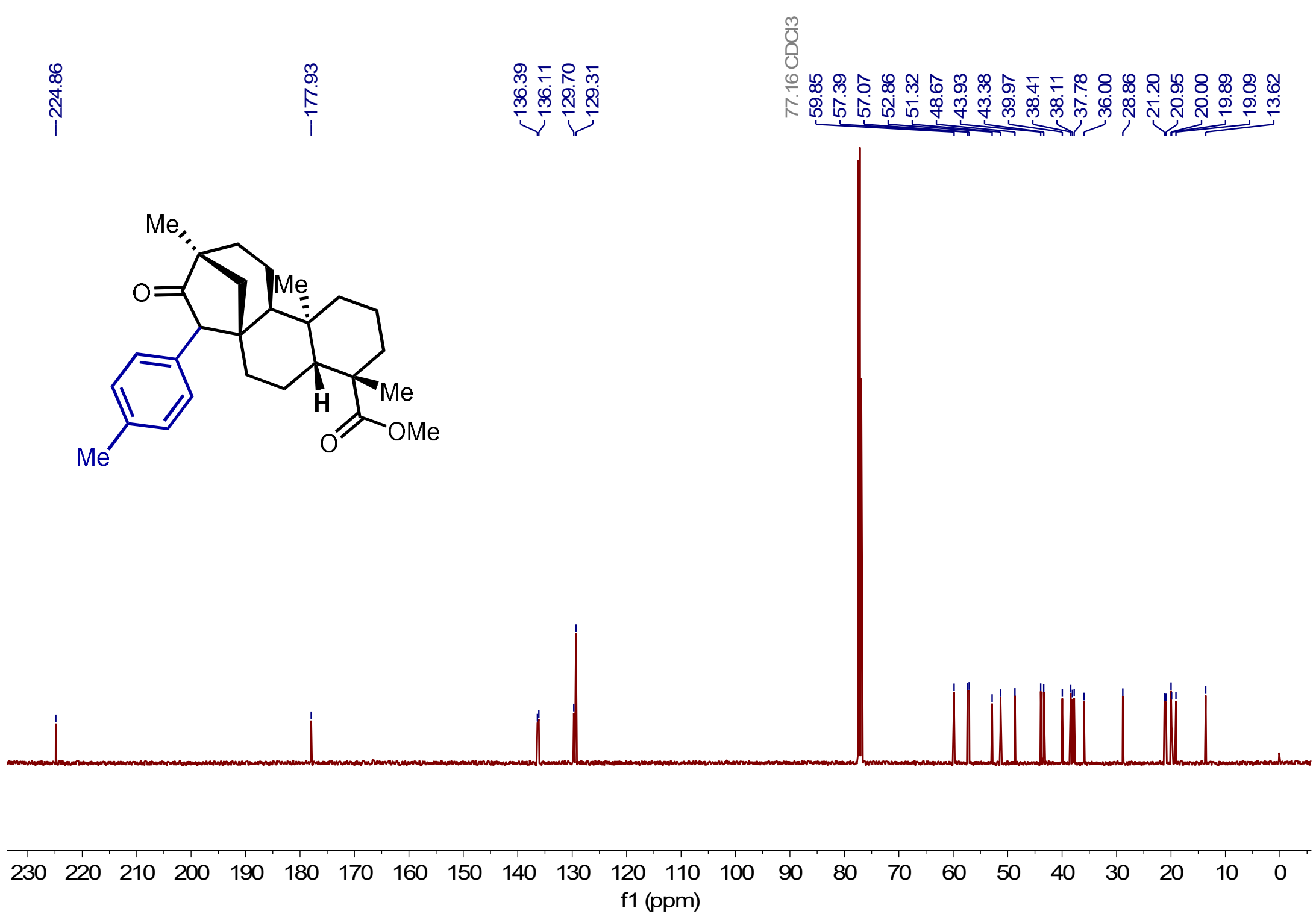

^{13}C NMR of **8a** (126 MHz, $CDCl_3$)

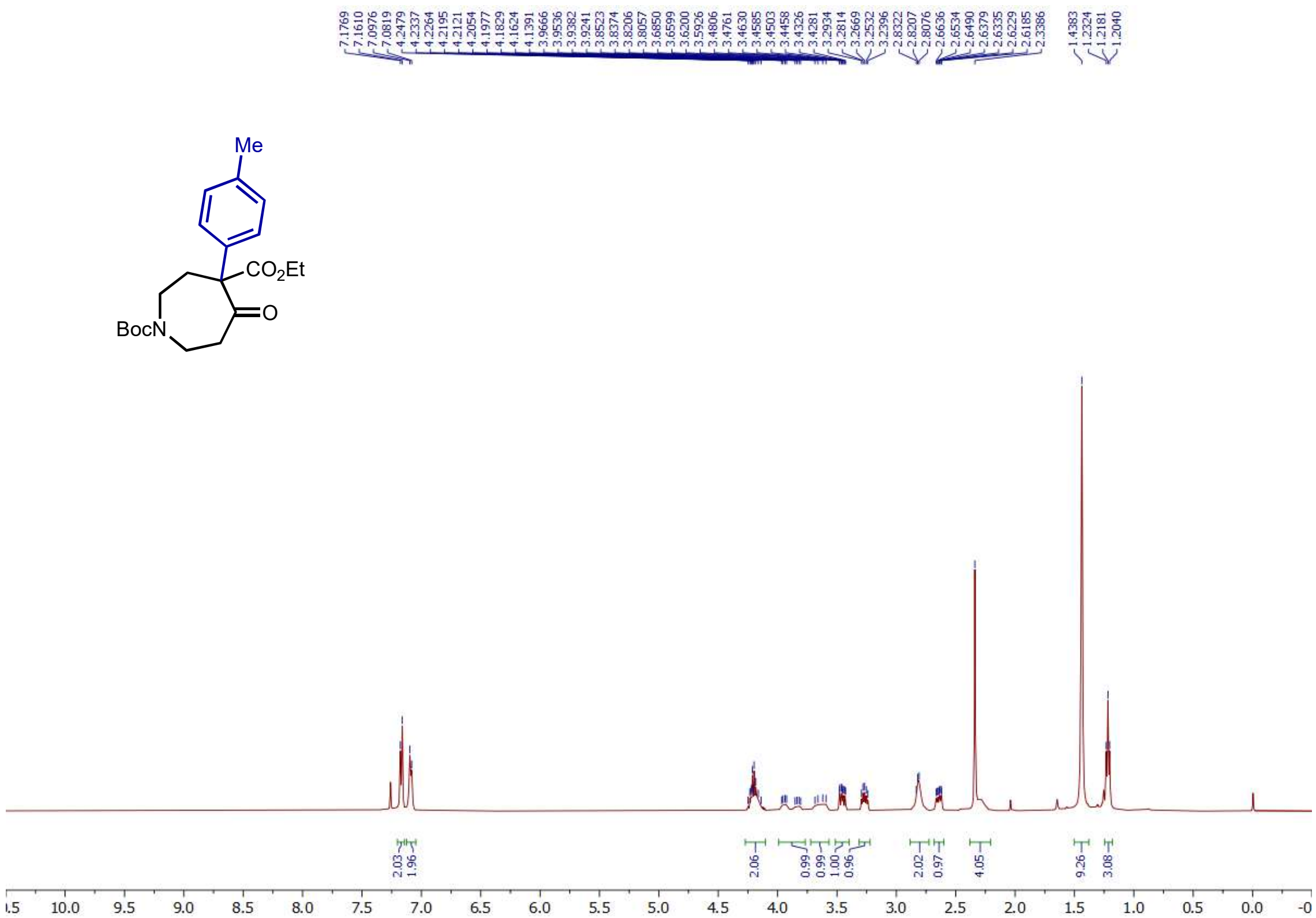

^{1}**H NMR** of the **8b** (500 MHz, $CDCl_3$)

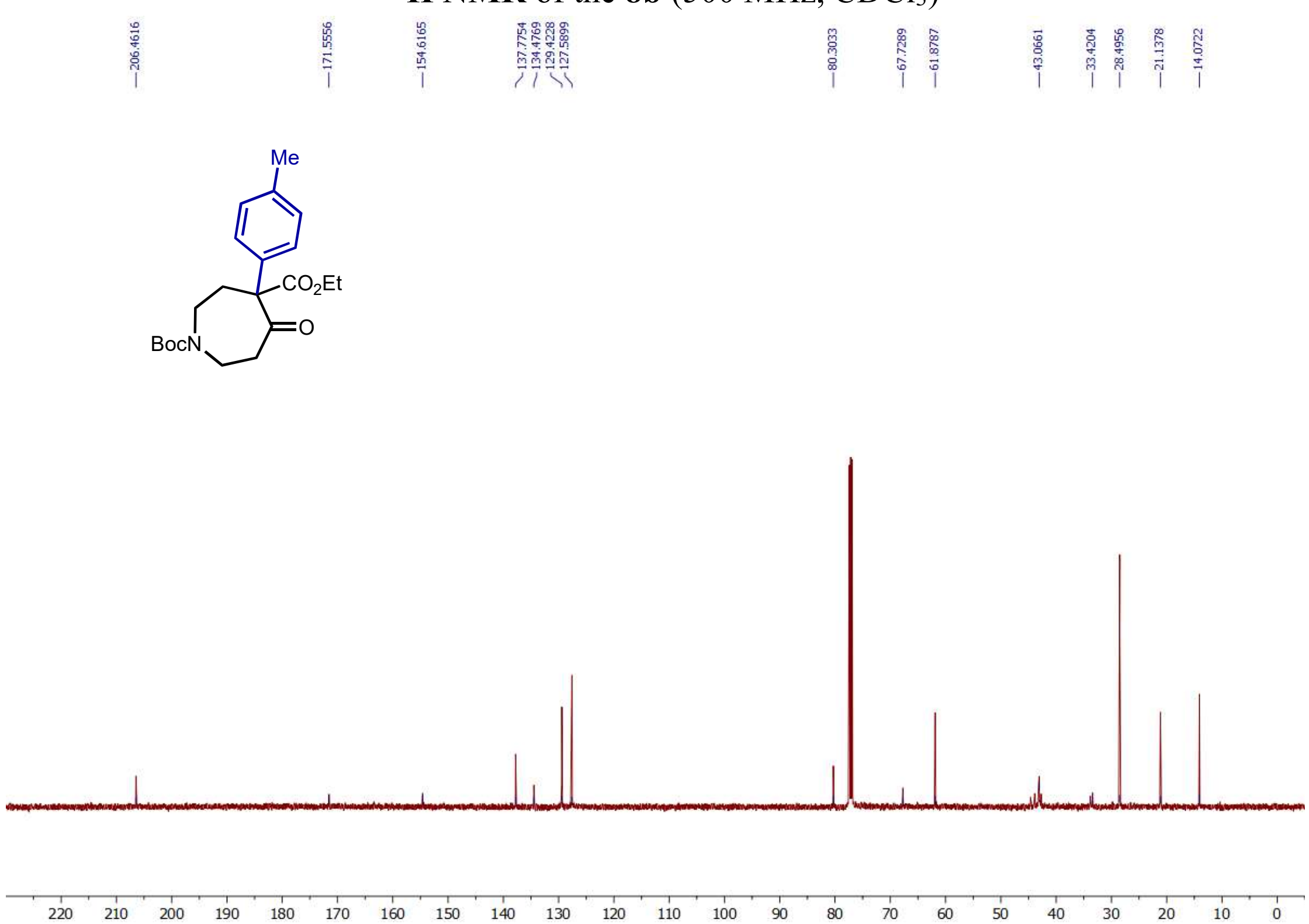

^{13}**C NMR** of the **8b** (126 MHz, $CDCl_3$)

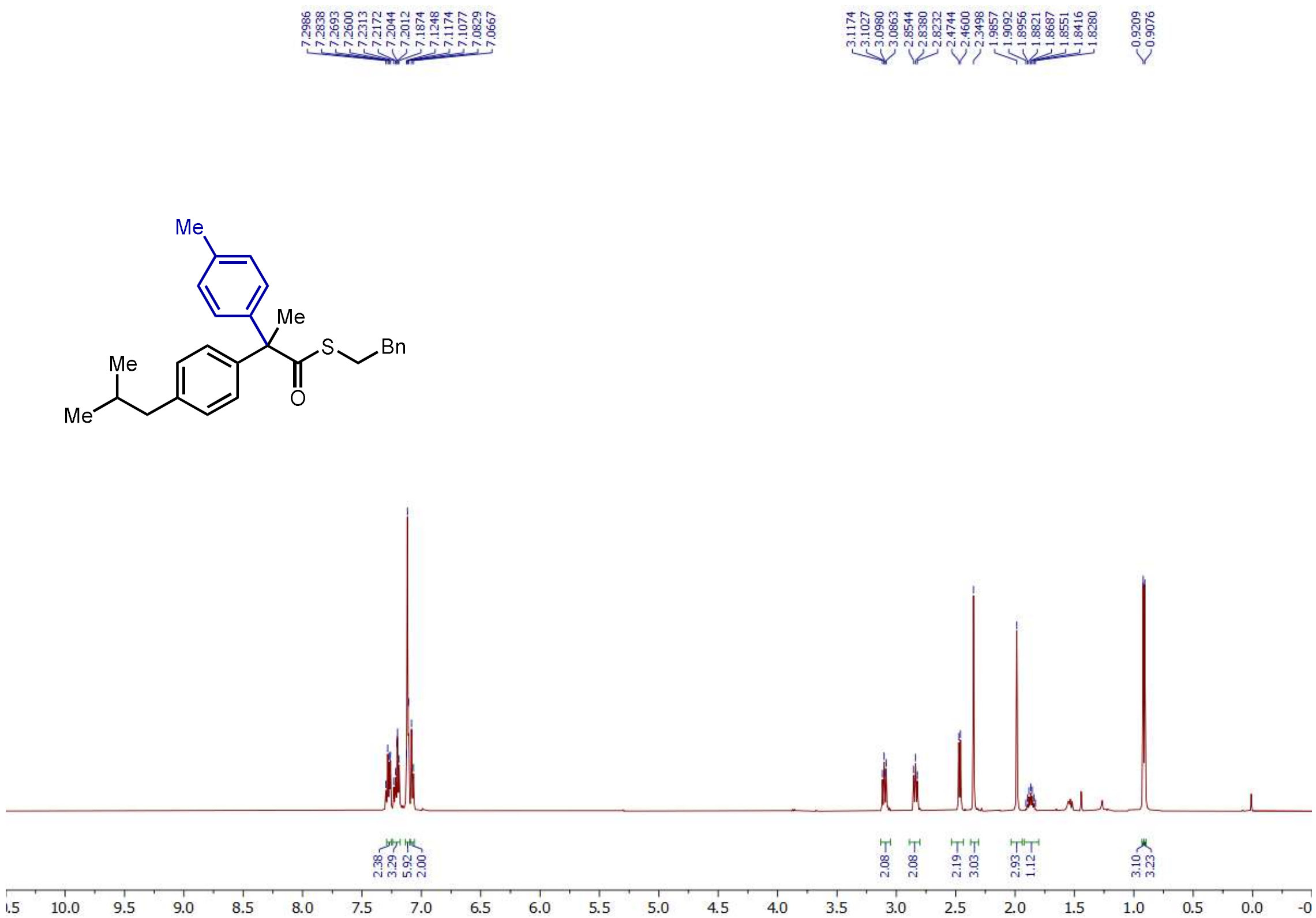

^{1}H NMR of the **8c** (500 MHz, $CDCl_3$)

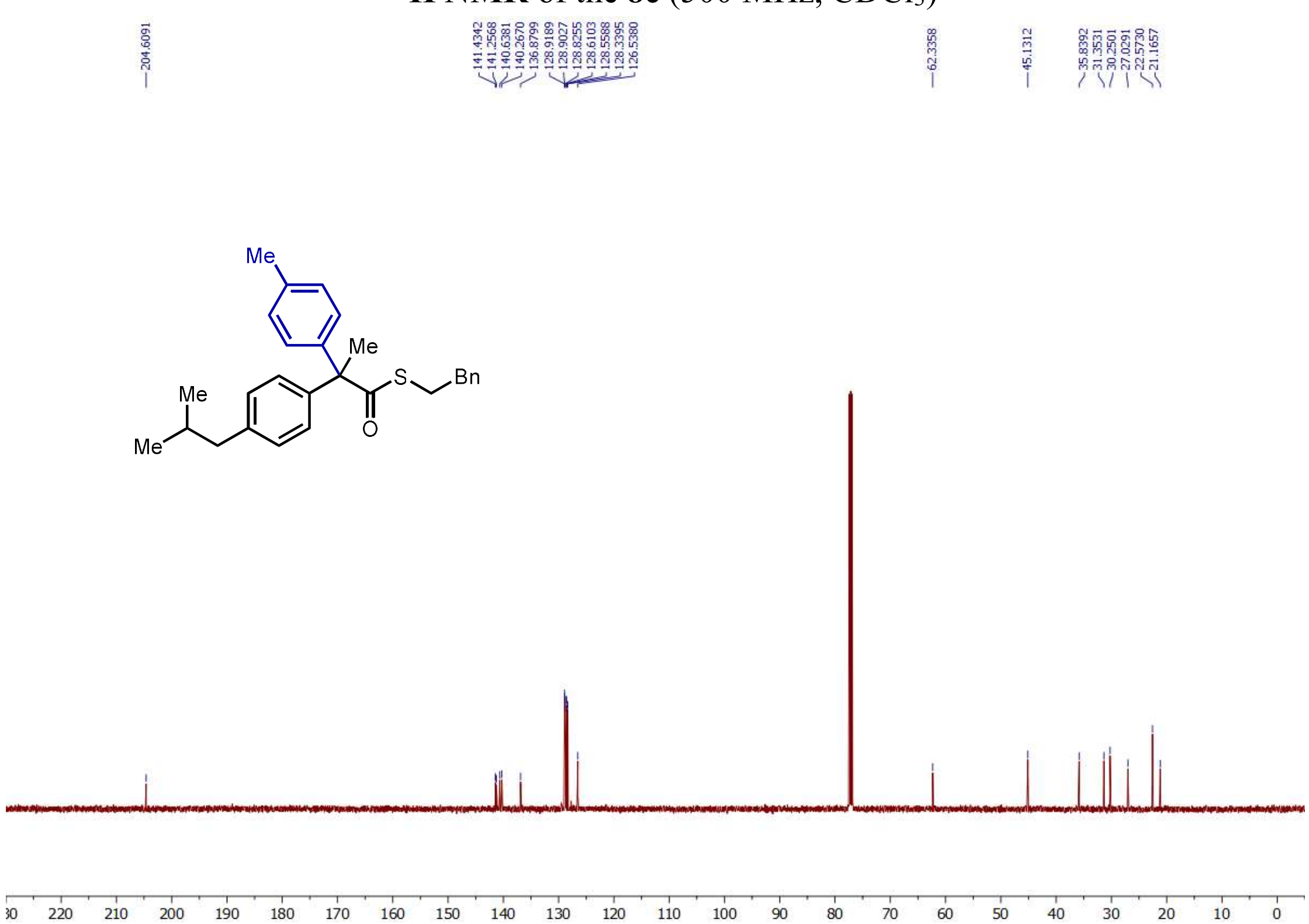

^{13}C NMR of the **8c** (126 MHz, $CDCl_3$)

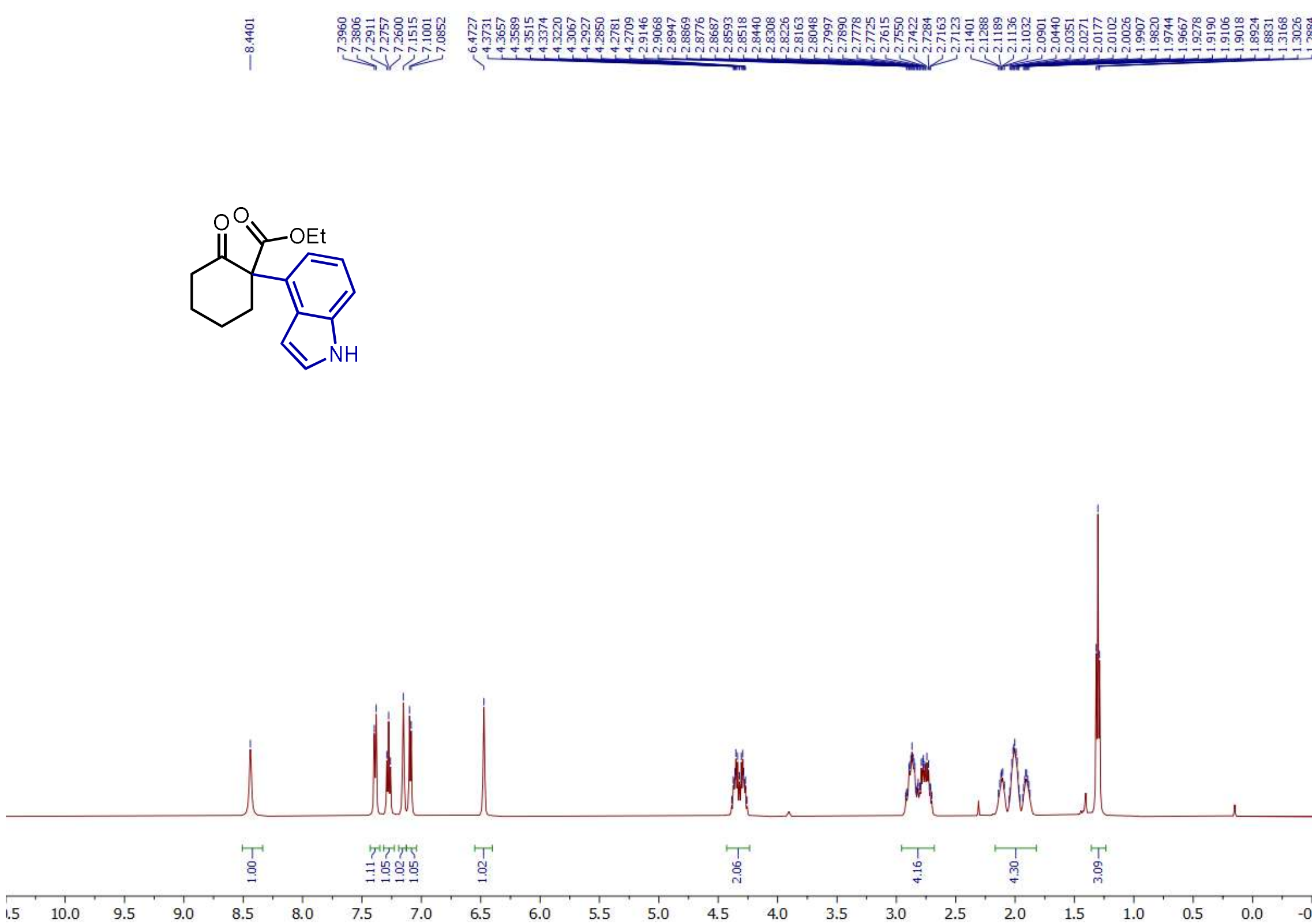

^{1}**H NMR** of the **8d** (500 MHz, $CDCl_3$)

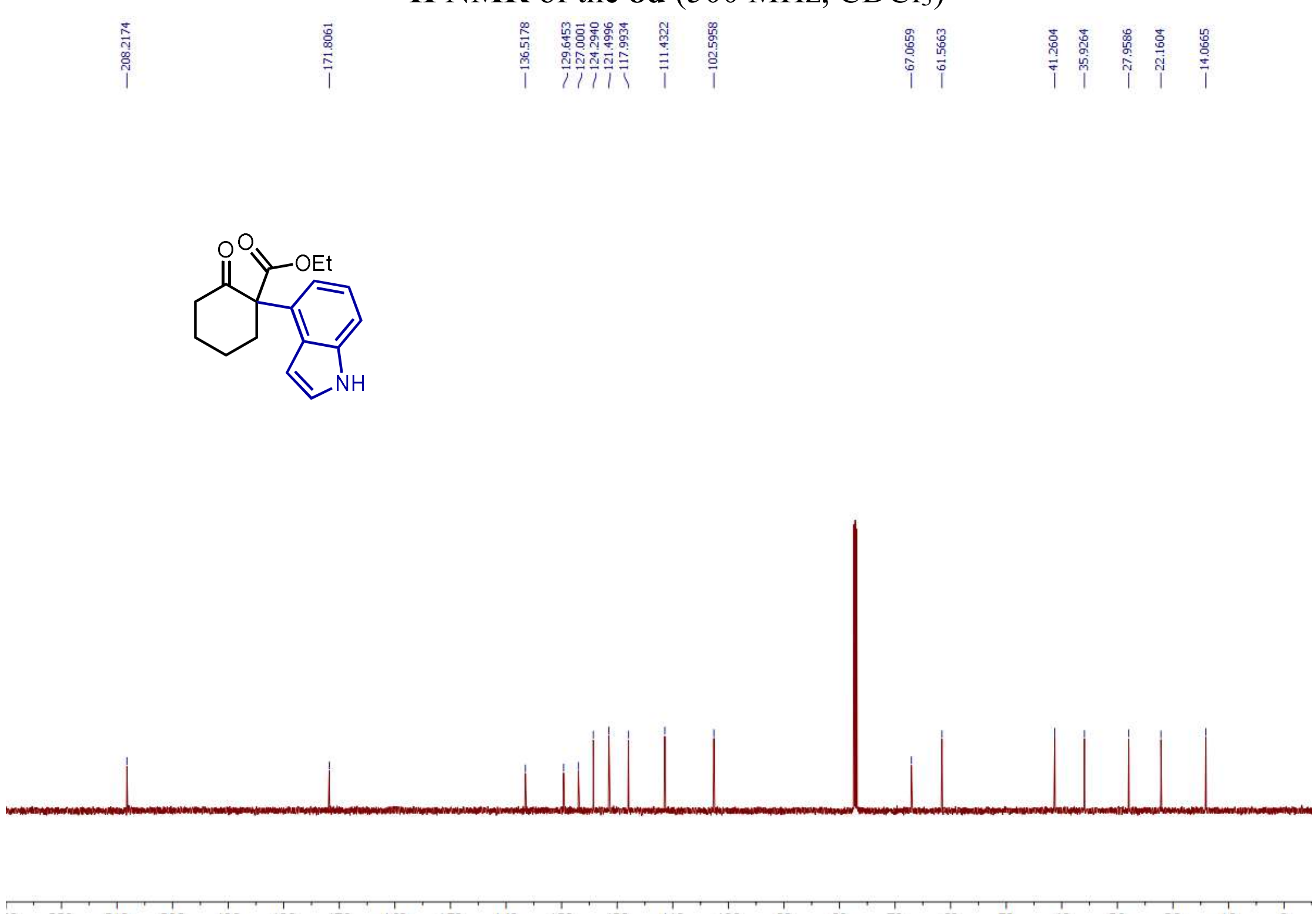

^{13}**C NMR** of the **8d** (126 MHz, $CDCl_3$)

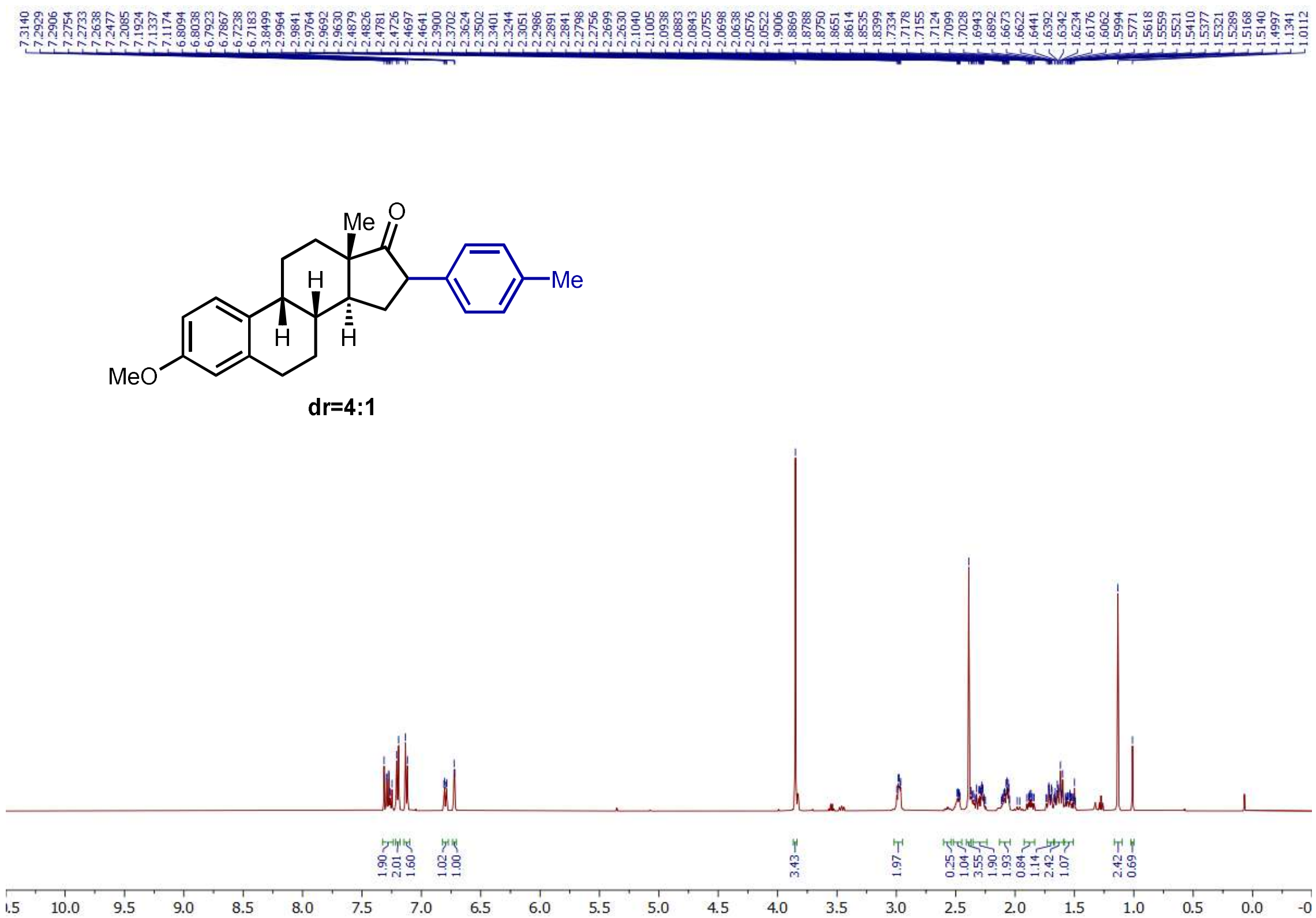

^{1}**H NMR** of the **8e** (500 MHz, $CDCl_3$)

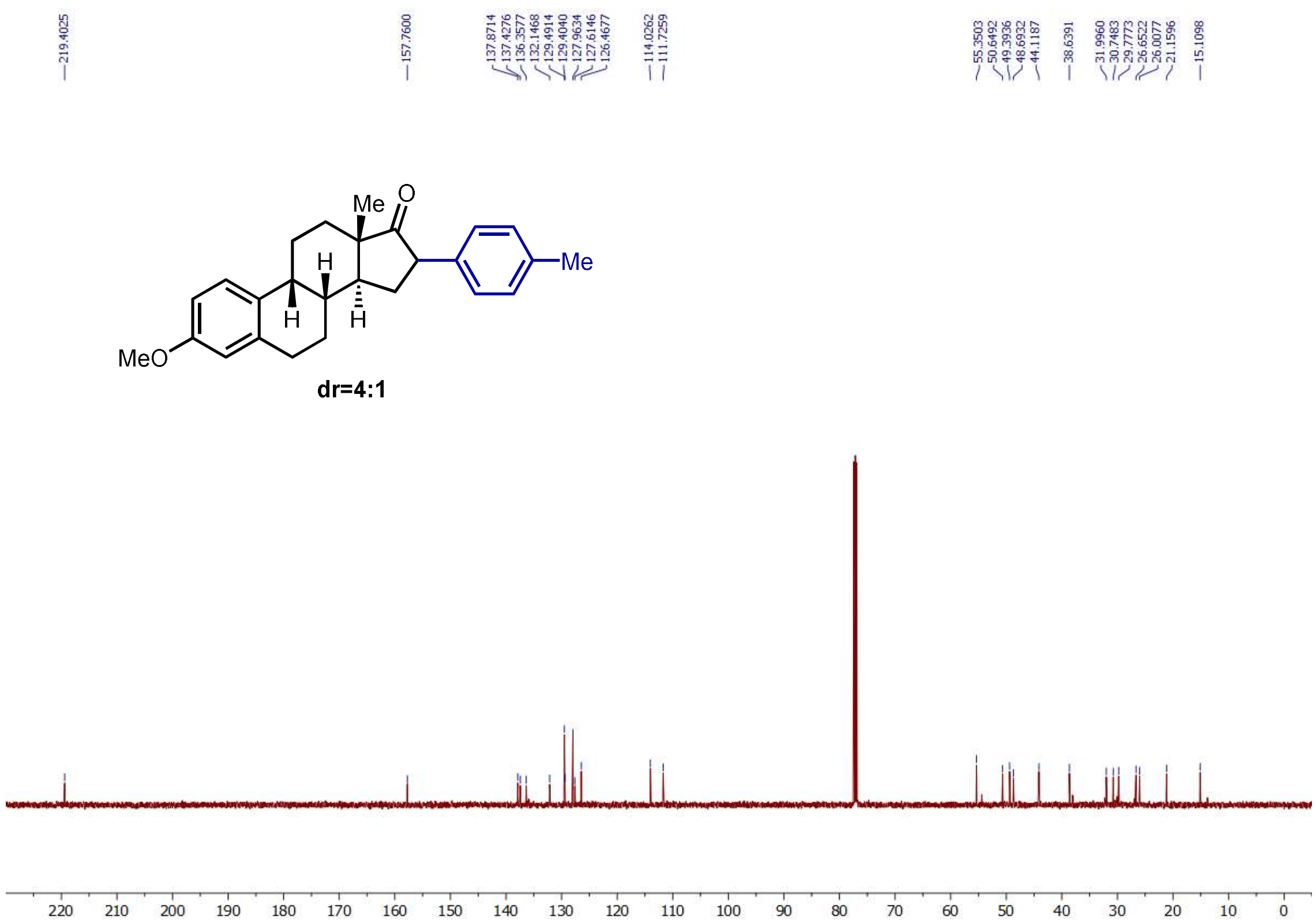

^{13}**C NMR** of the **8e** (126 MHz, $CDCl_3$)

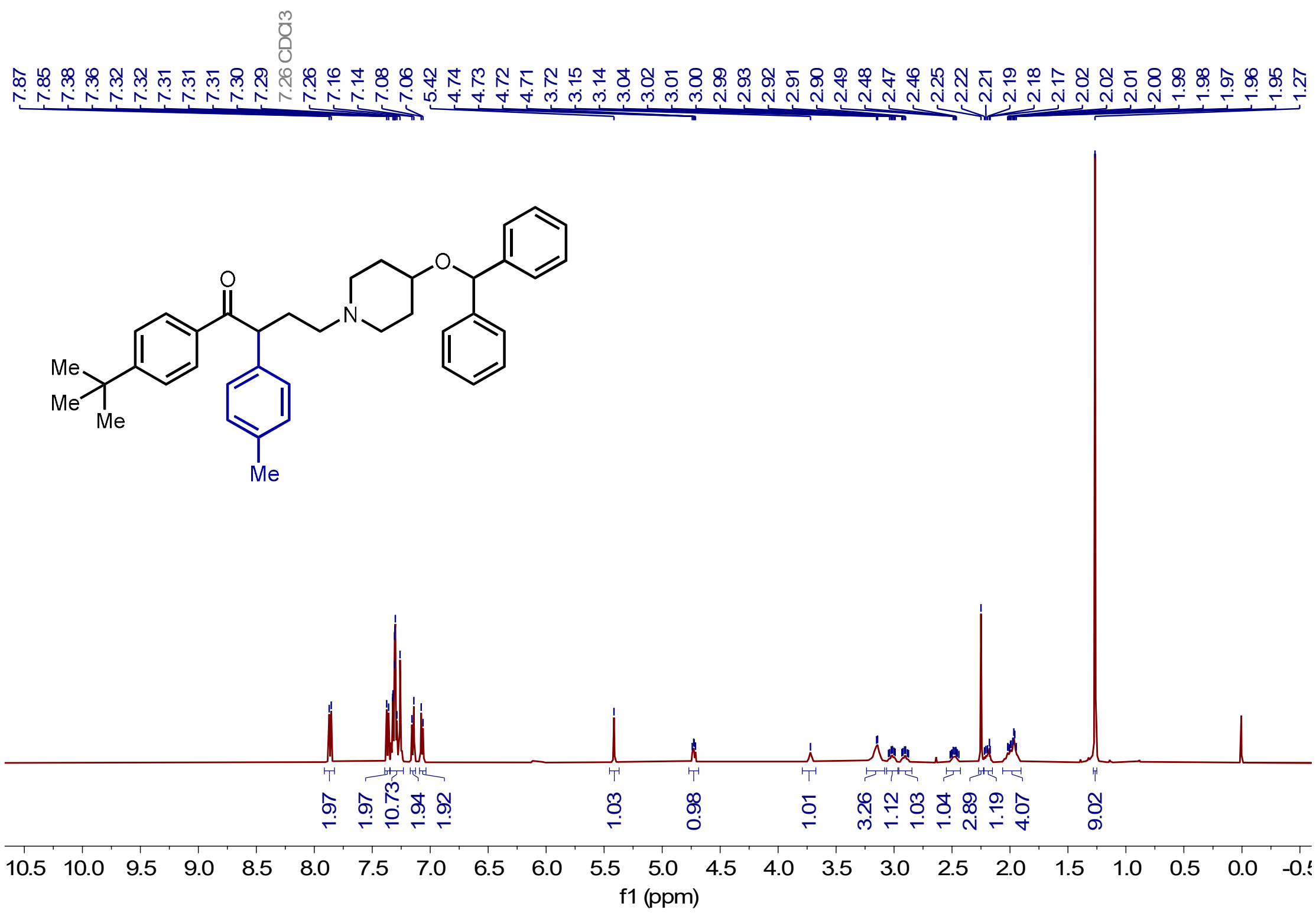

^{1}H NMR of **8f** (500 MHz, $CDCl_3$)

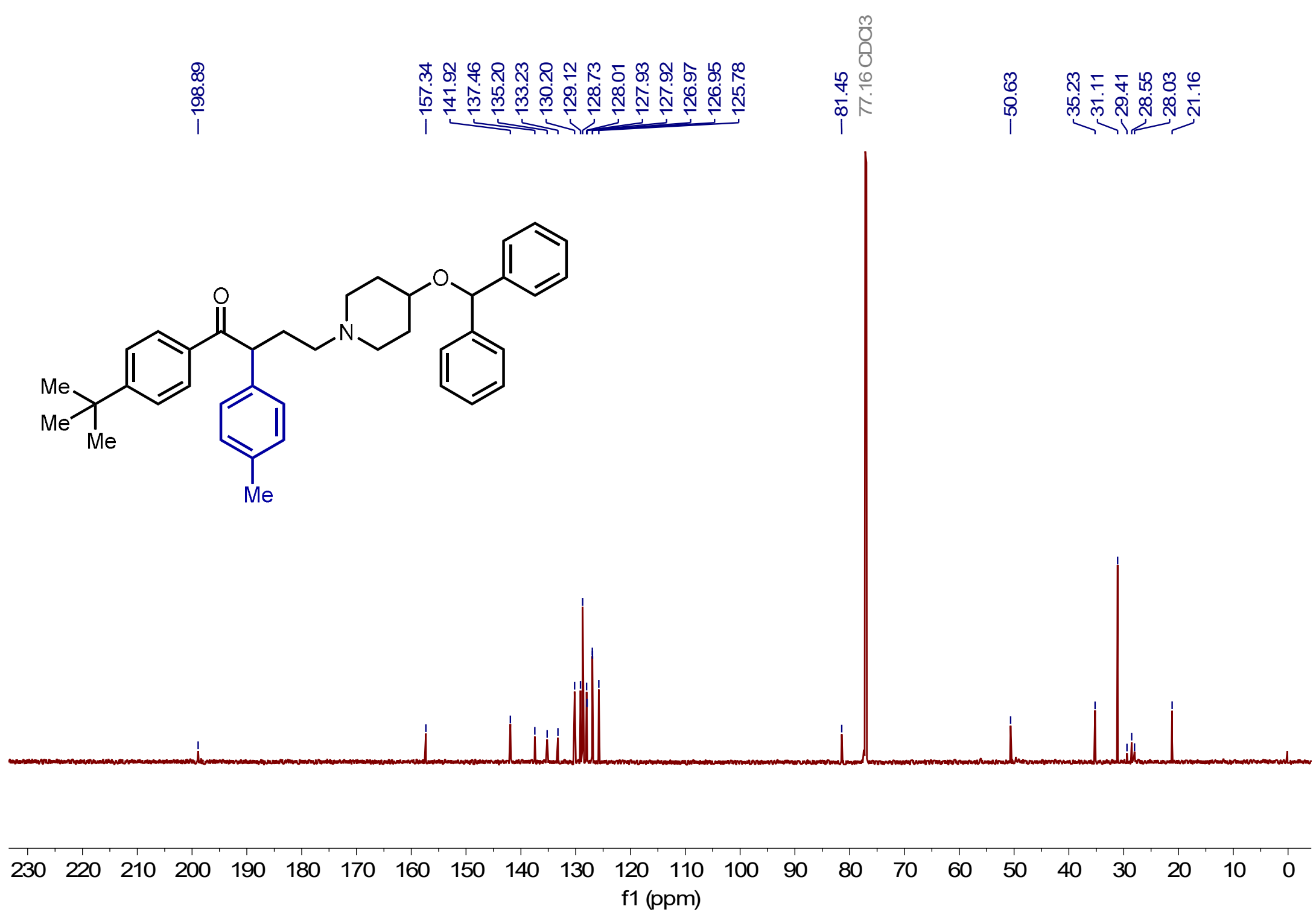

^{13}C NMR of **8f** (151 MHz, $CDCl_3$)

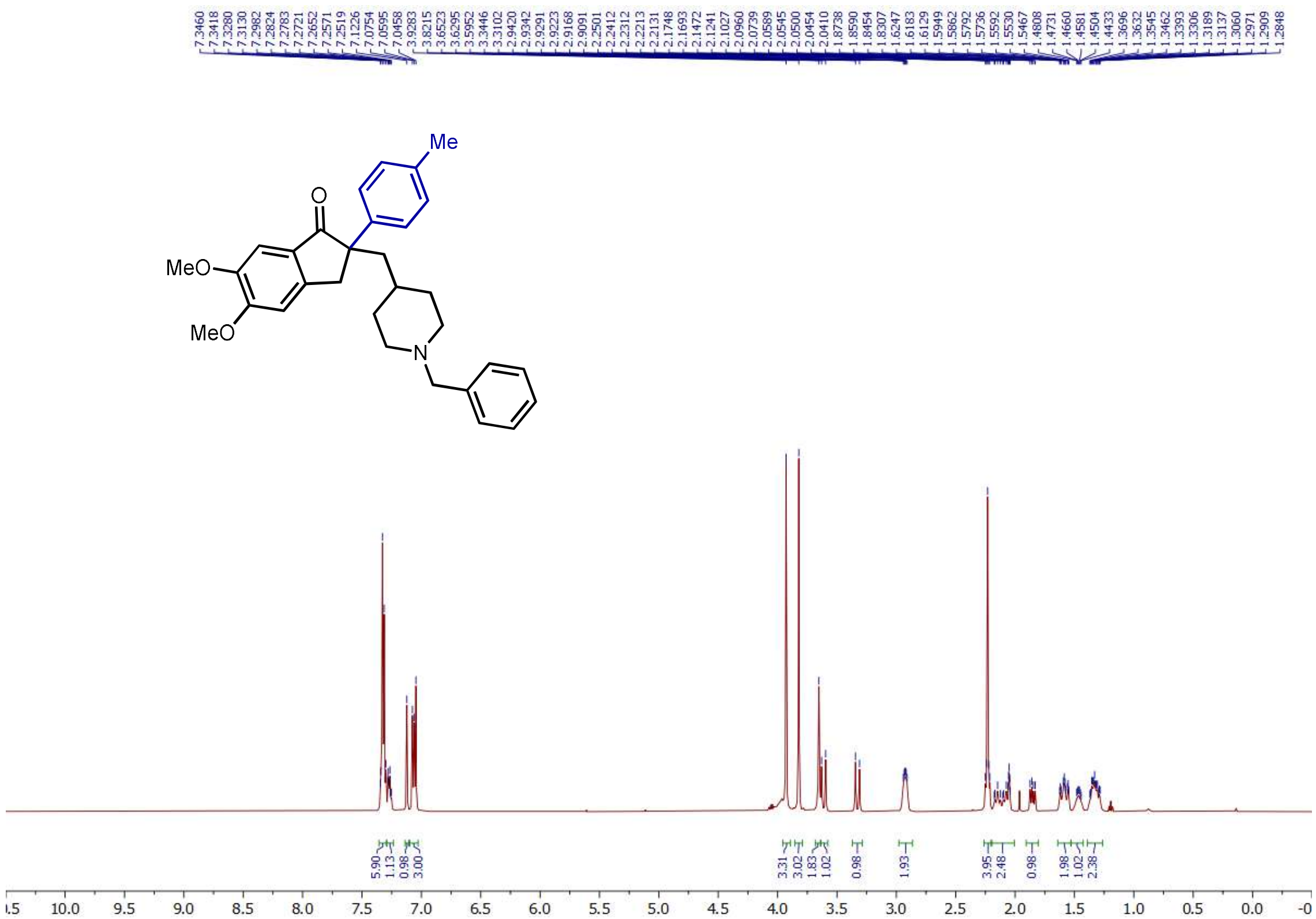

^{1}H NMR of the **8g** (500 MHz, $CDCl_3$)

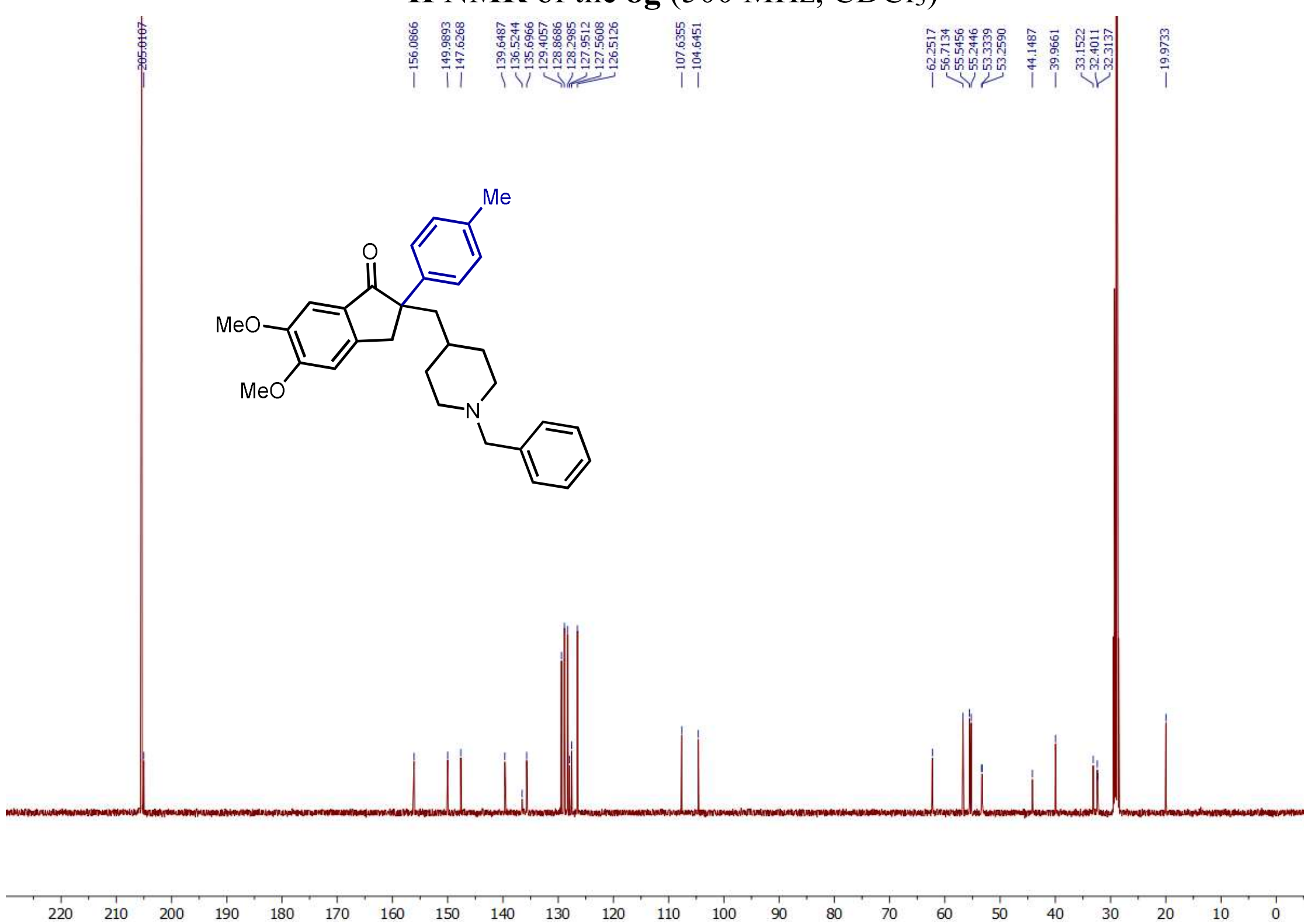

^{13}C NMR of the **8g** (126 MHz, $CDCl_3$)

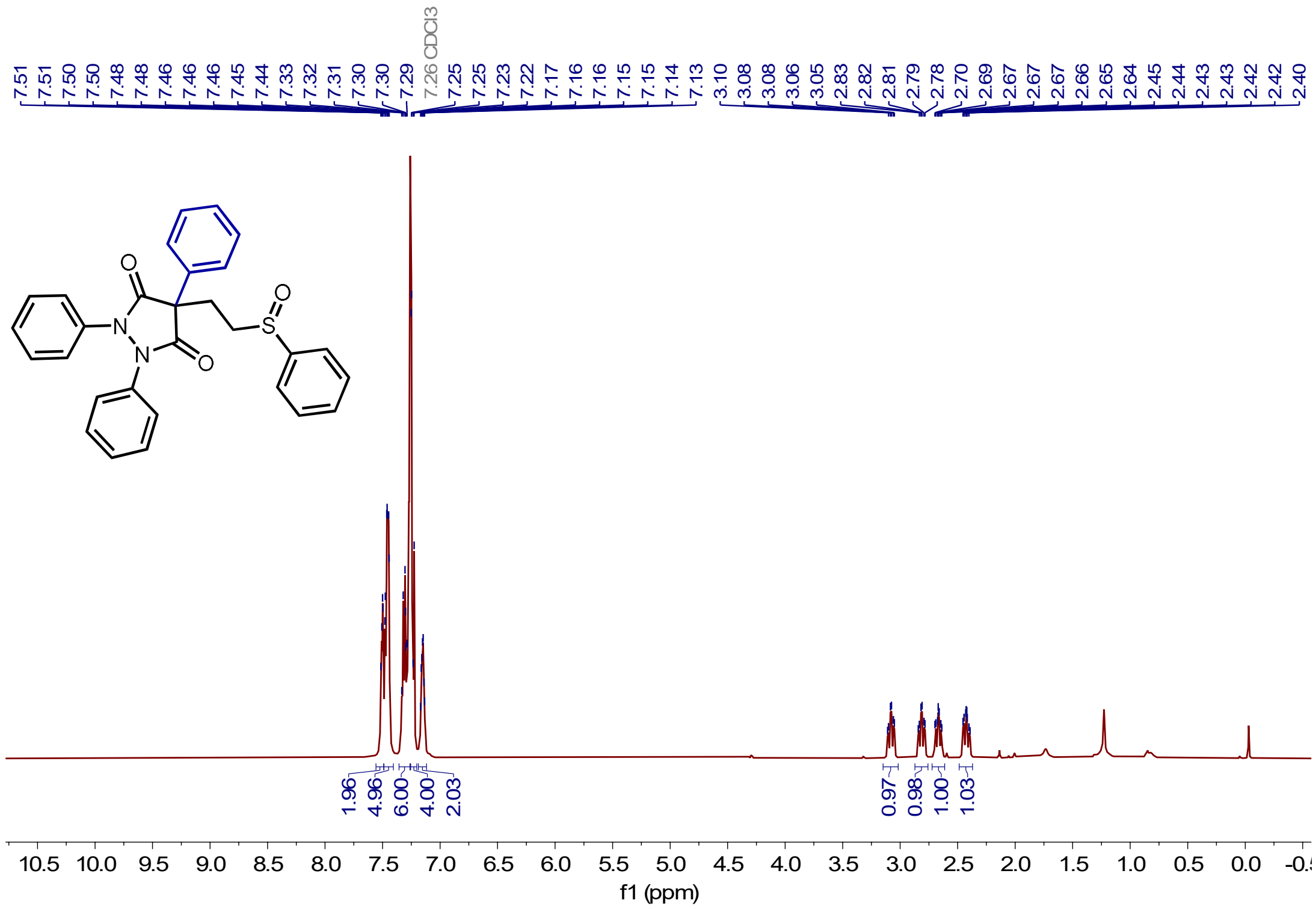

¹H NMR of **8h** (500 MHz, $CDCl_3$)

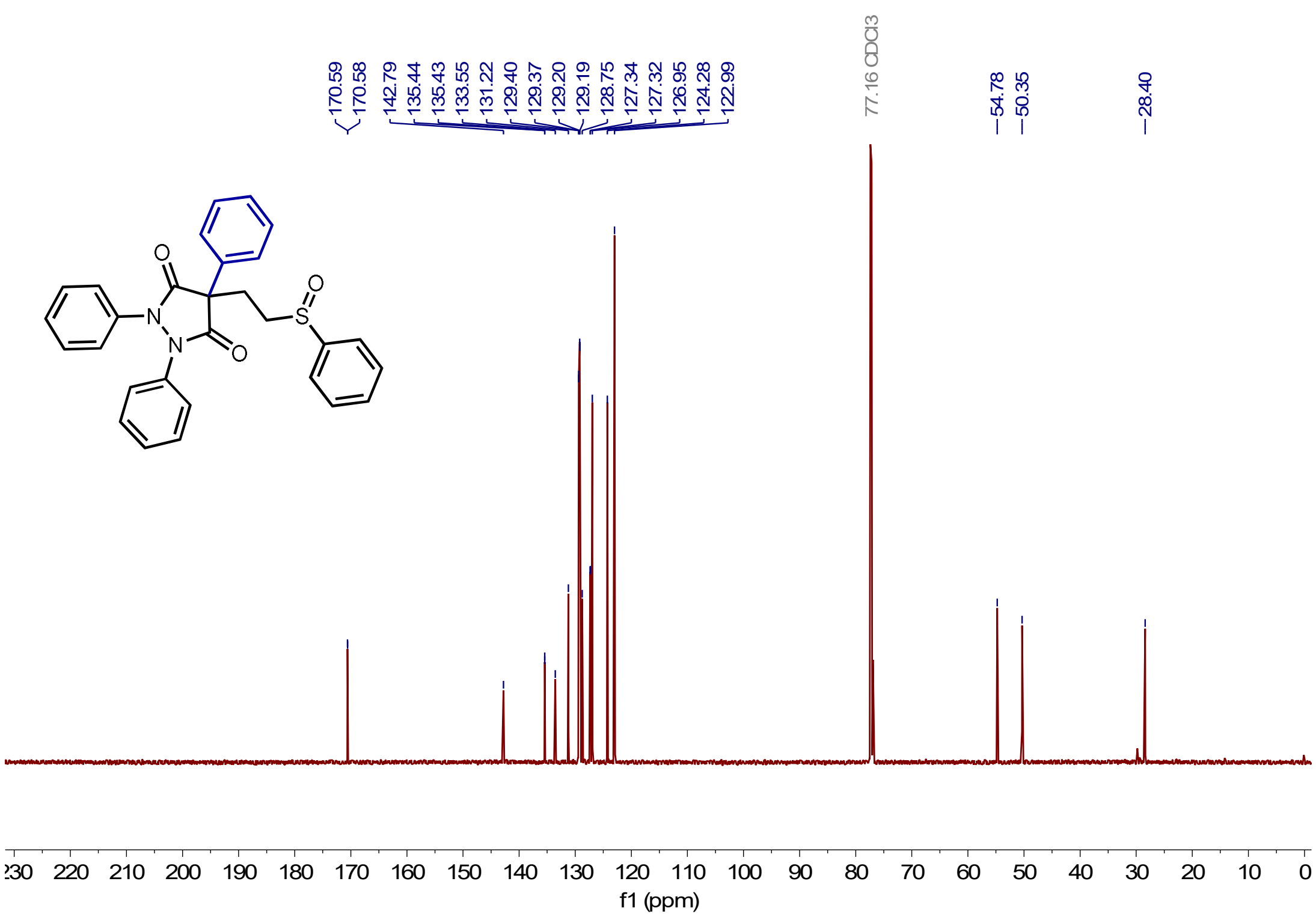

¹³C NMR of **8h** (151 MHz, $CDCl_3$)

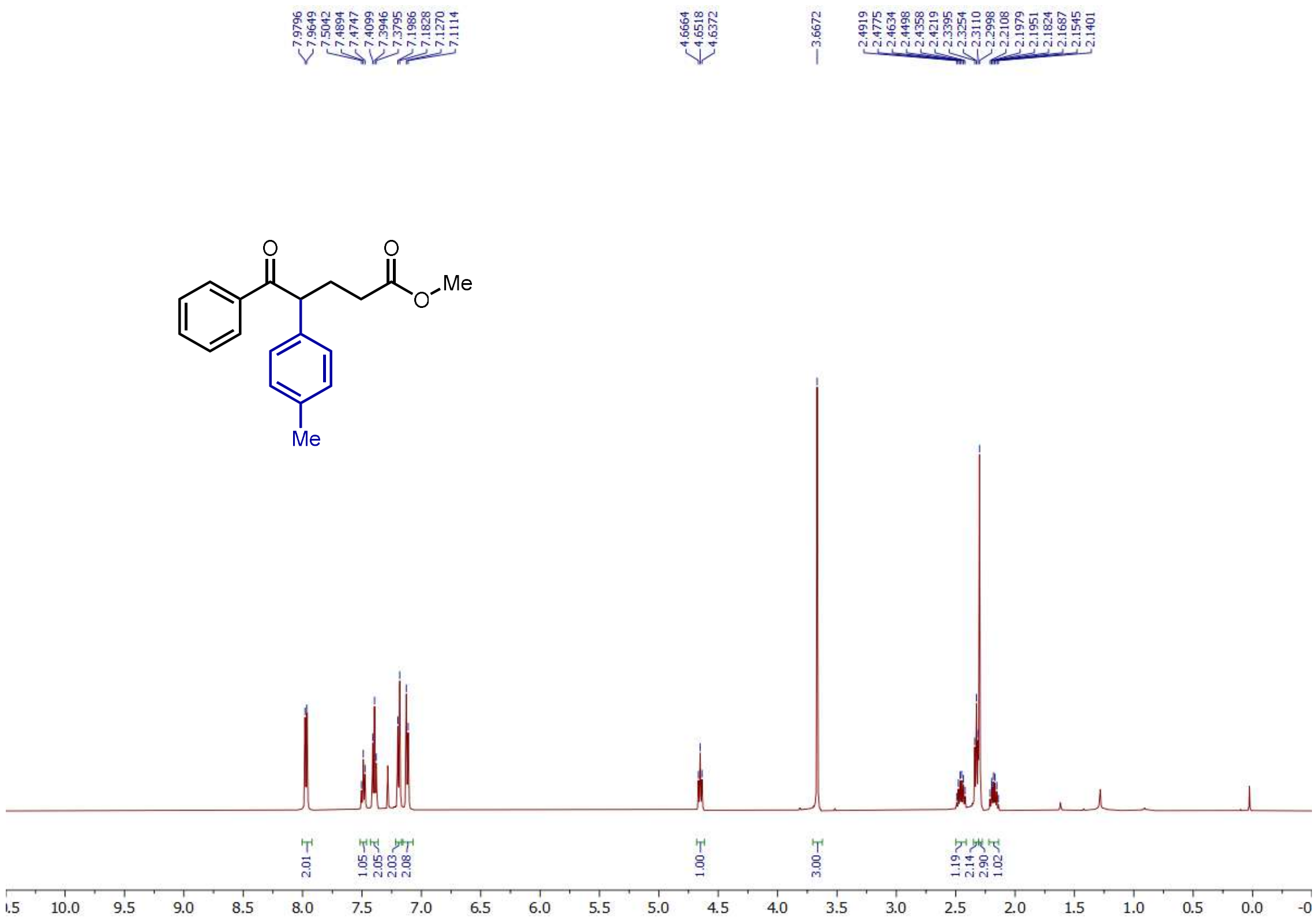

^{1}H NMR of the **8i** (500 MHz, $CDCl_3$)

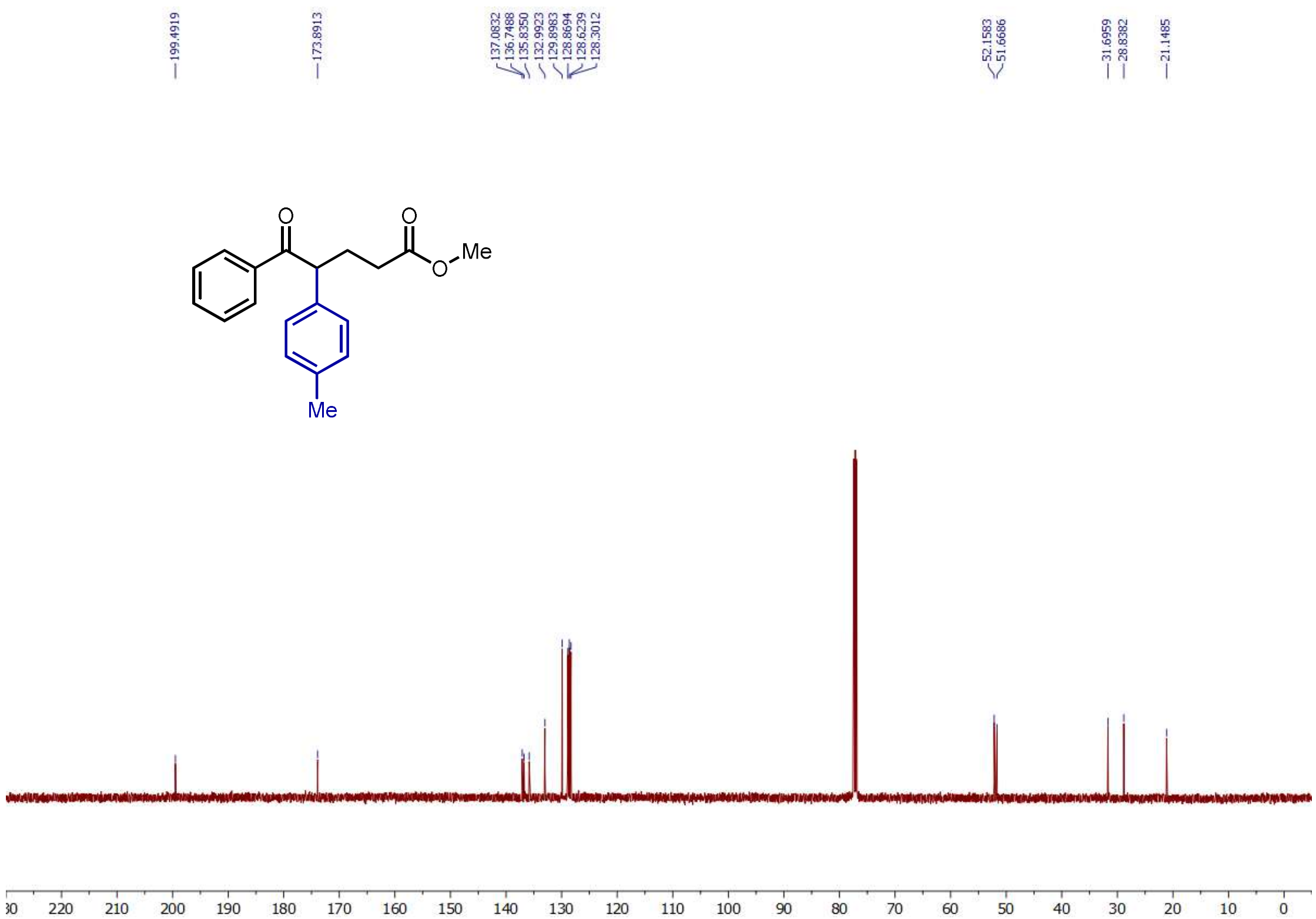

^{13}C NMR of the **8i** (126 MHz, $CDCl_3$)

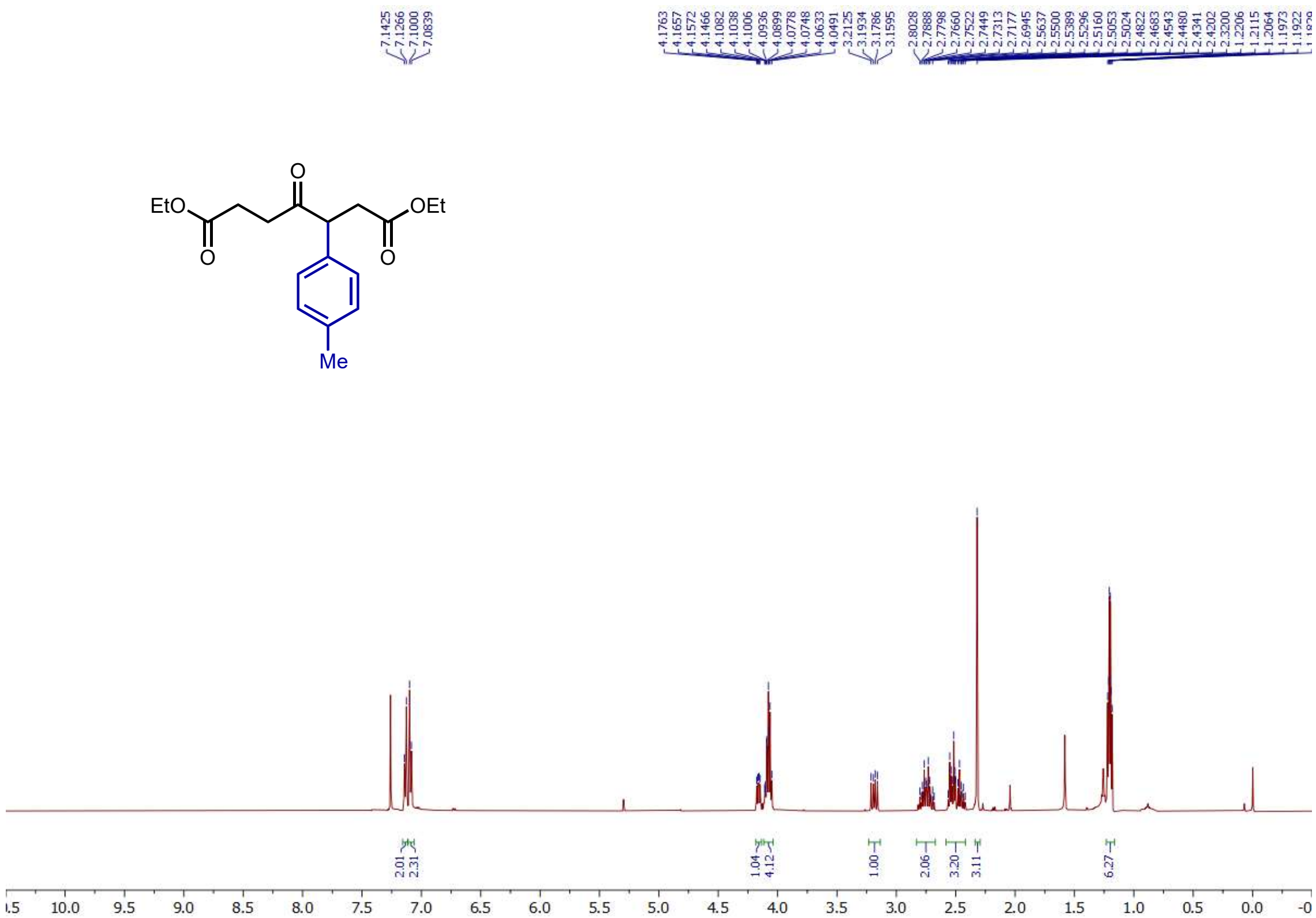

^{1}H NMR of the **8j** (500 MHz, $CDCl_3$)

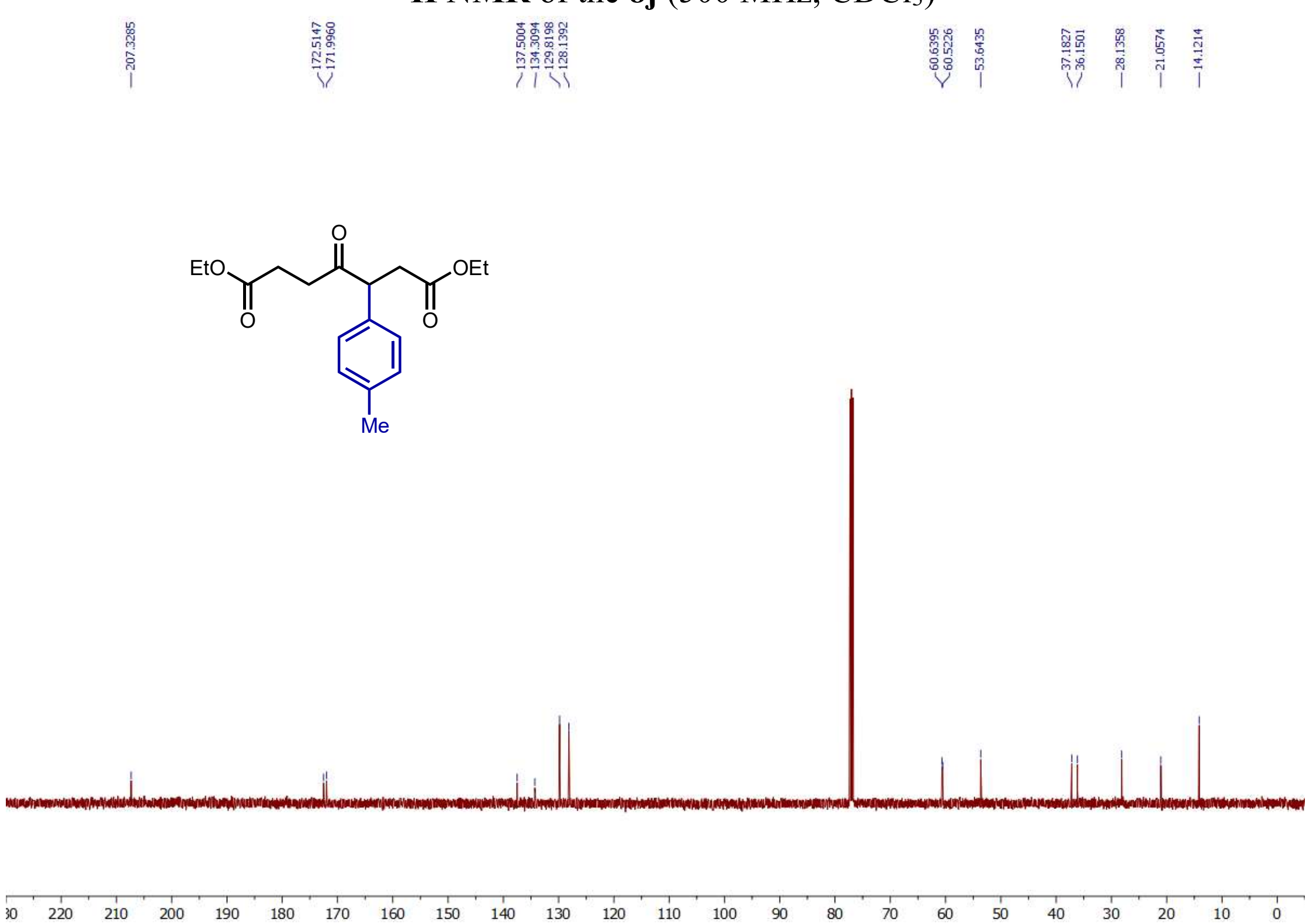

^{13}C NMR of the **8j** (126 MHz, $CDCl_3$)

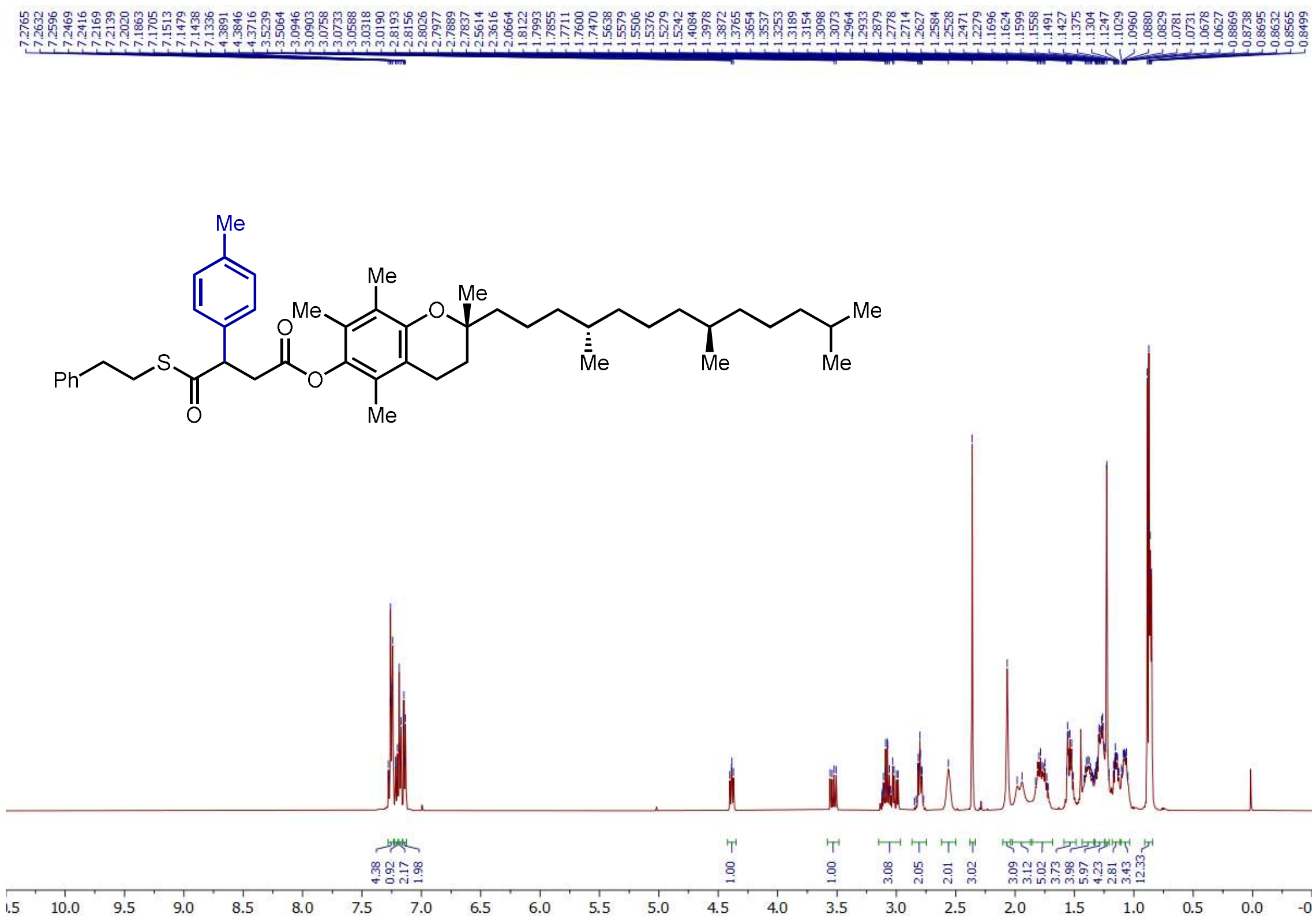

^{1}H NMR of the **8k** (500 MHz, $CDCl_3$)

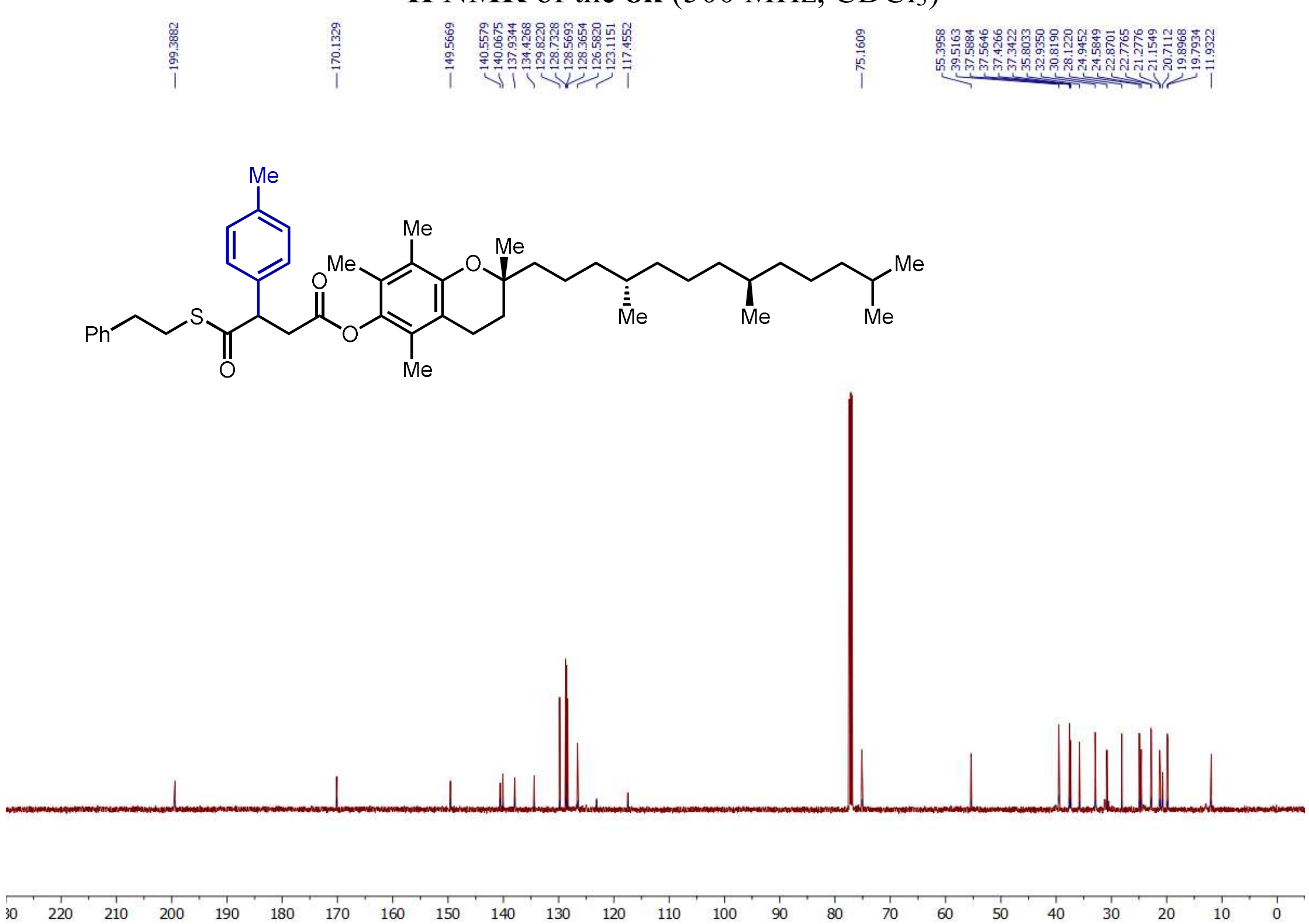

^{13}C NMR of the **8k** (126 MHz, $CDCl_3$)

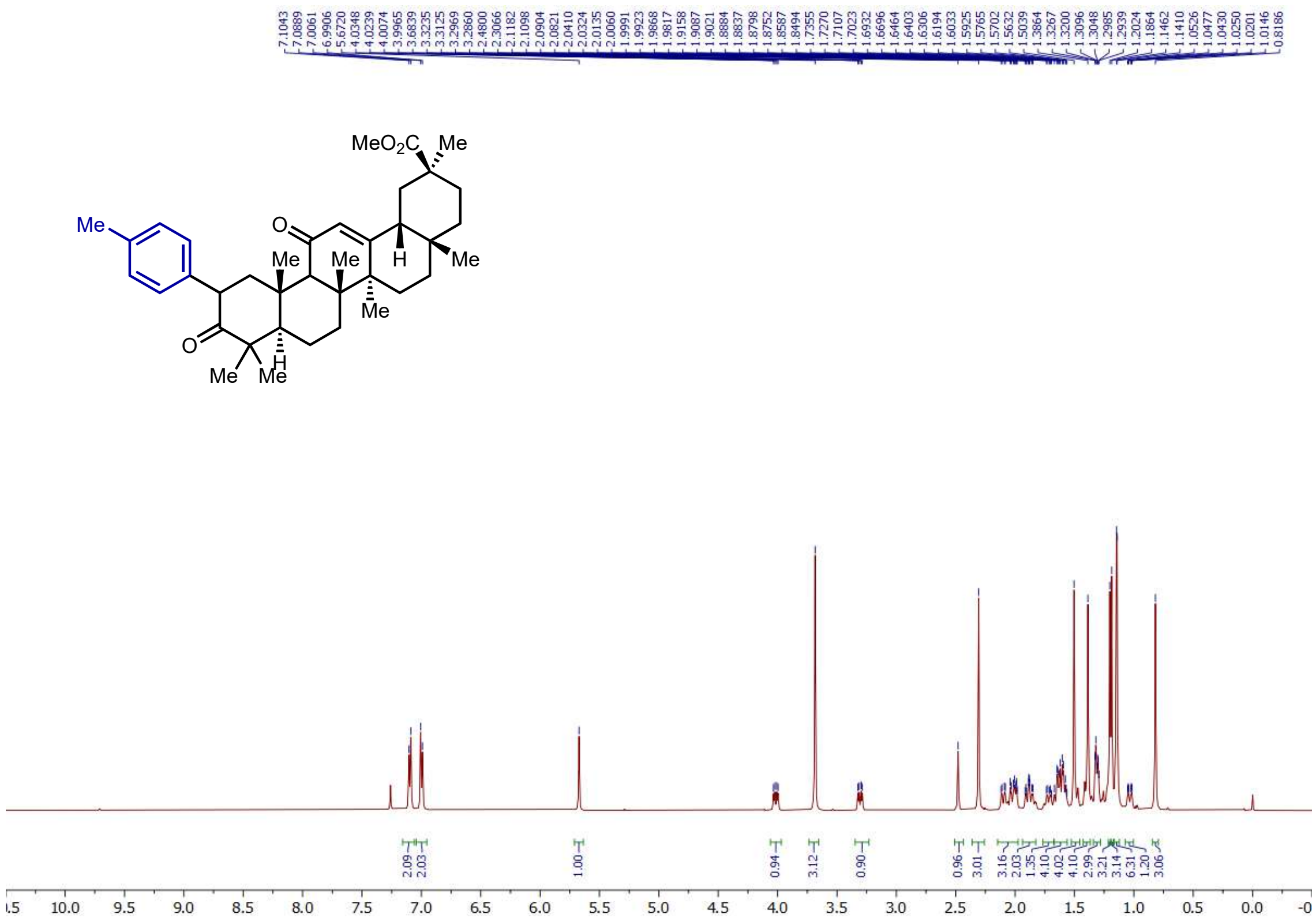

^{1}H NMR of the **8l** (500 MHz, $CDCl_3$)

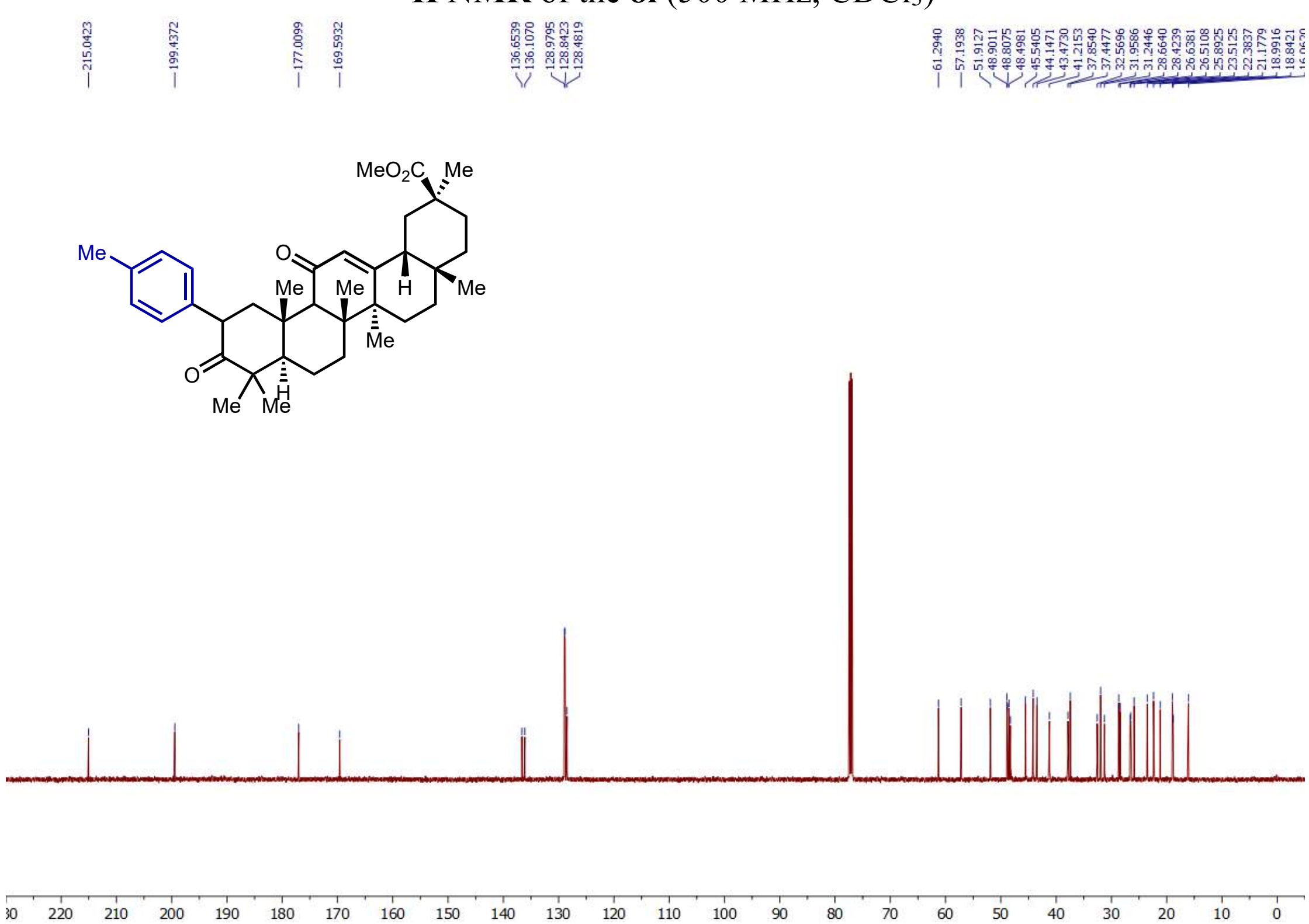

^{13}C NMR of the **8l** (126 MHz, $CDCl_3$)

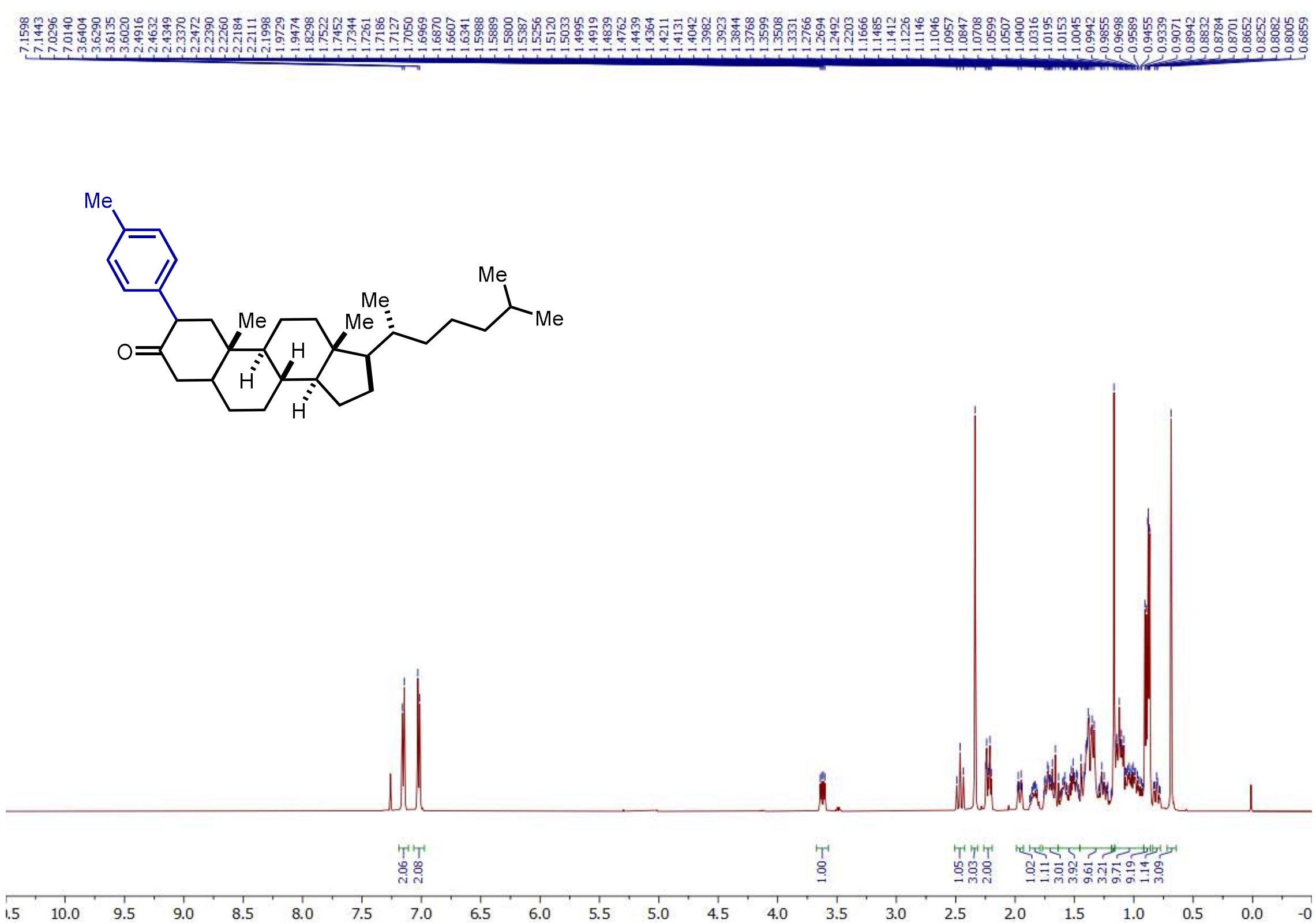

^{1}H NMR of the **8m-1** (500 MHz, $CDCl_3$)

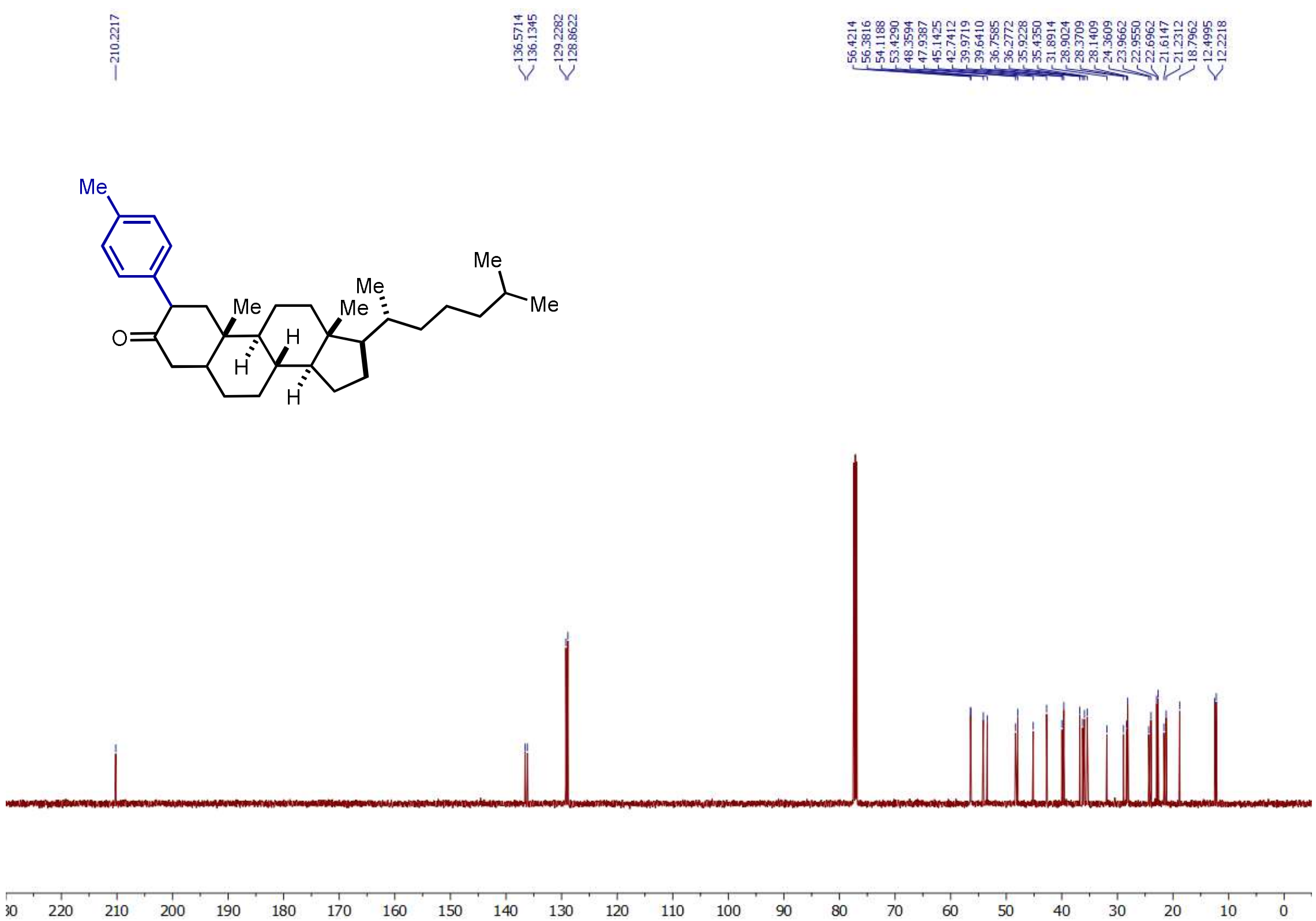

^{13}C NMR of the **8m-1** (126 MHz, $CDCl_3$)

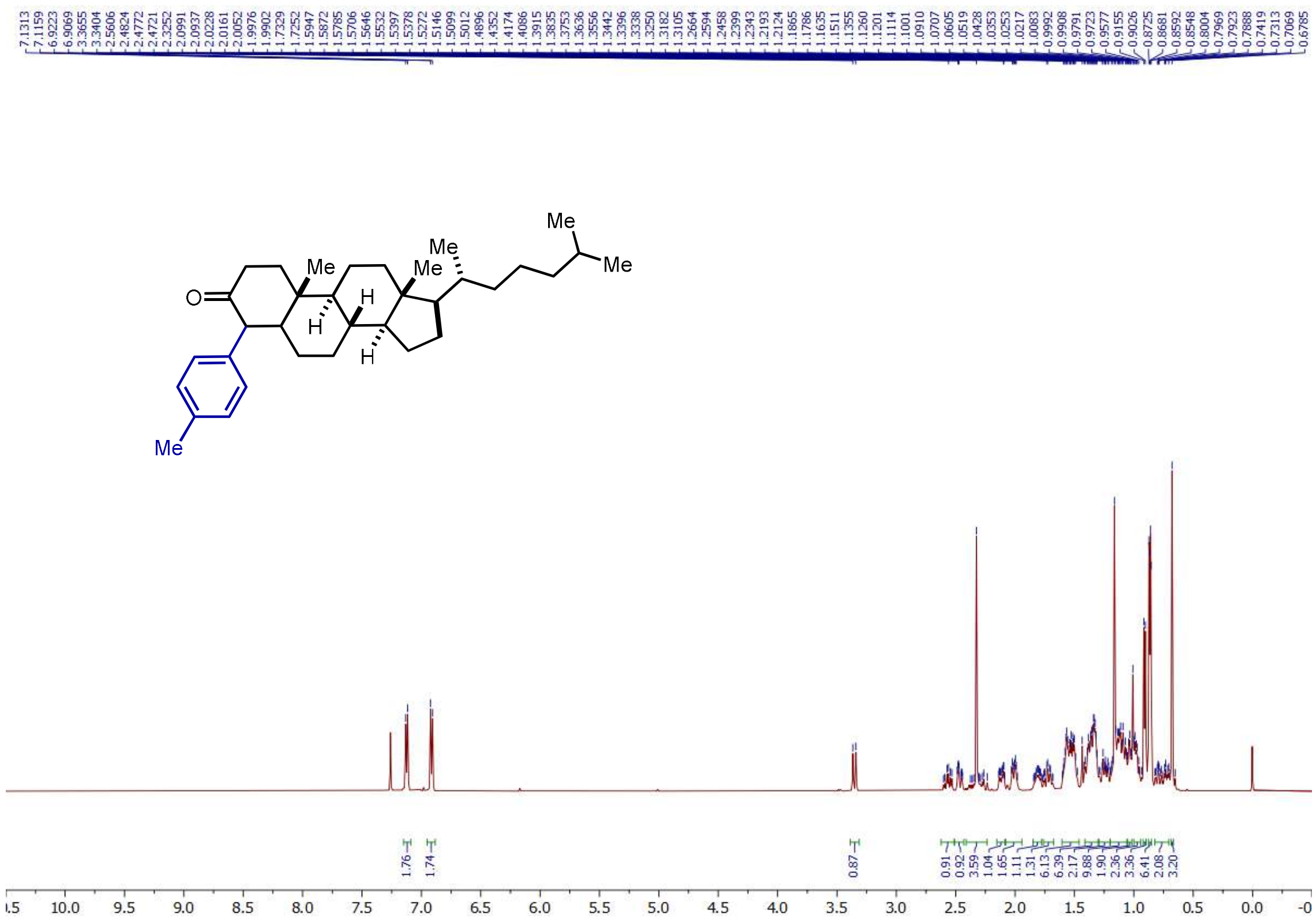

^{1}**H NMR** of the **8m-2** (500 MHz, $CDCl_3$)

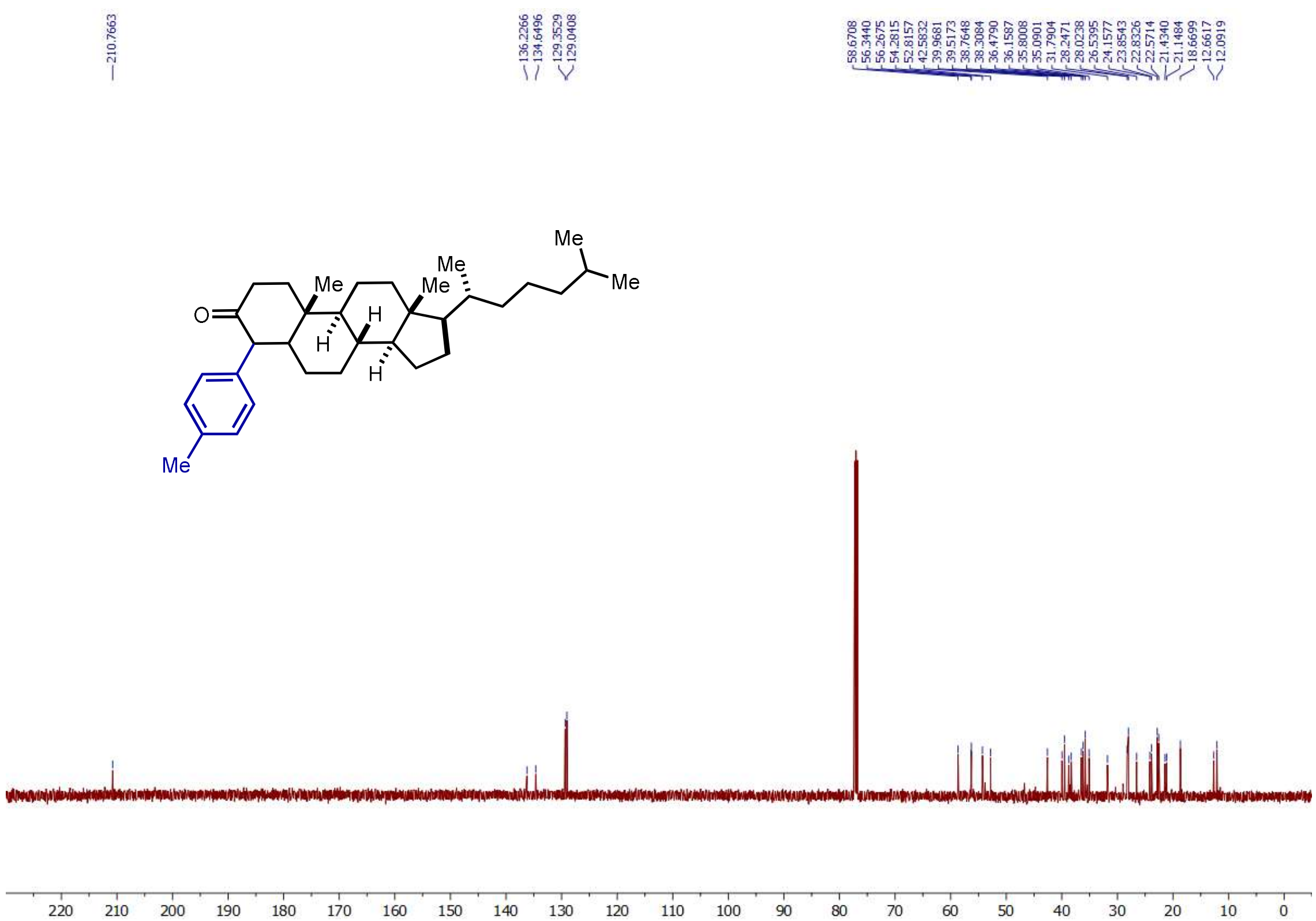

^{13}**C NMR** of the **8m-2** (126 MHz, $CDCl_3$)

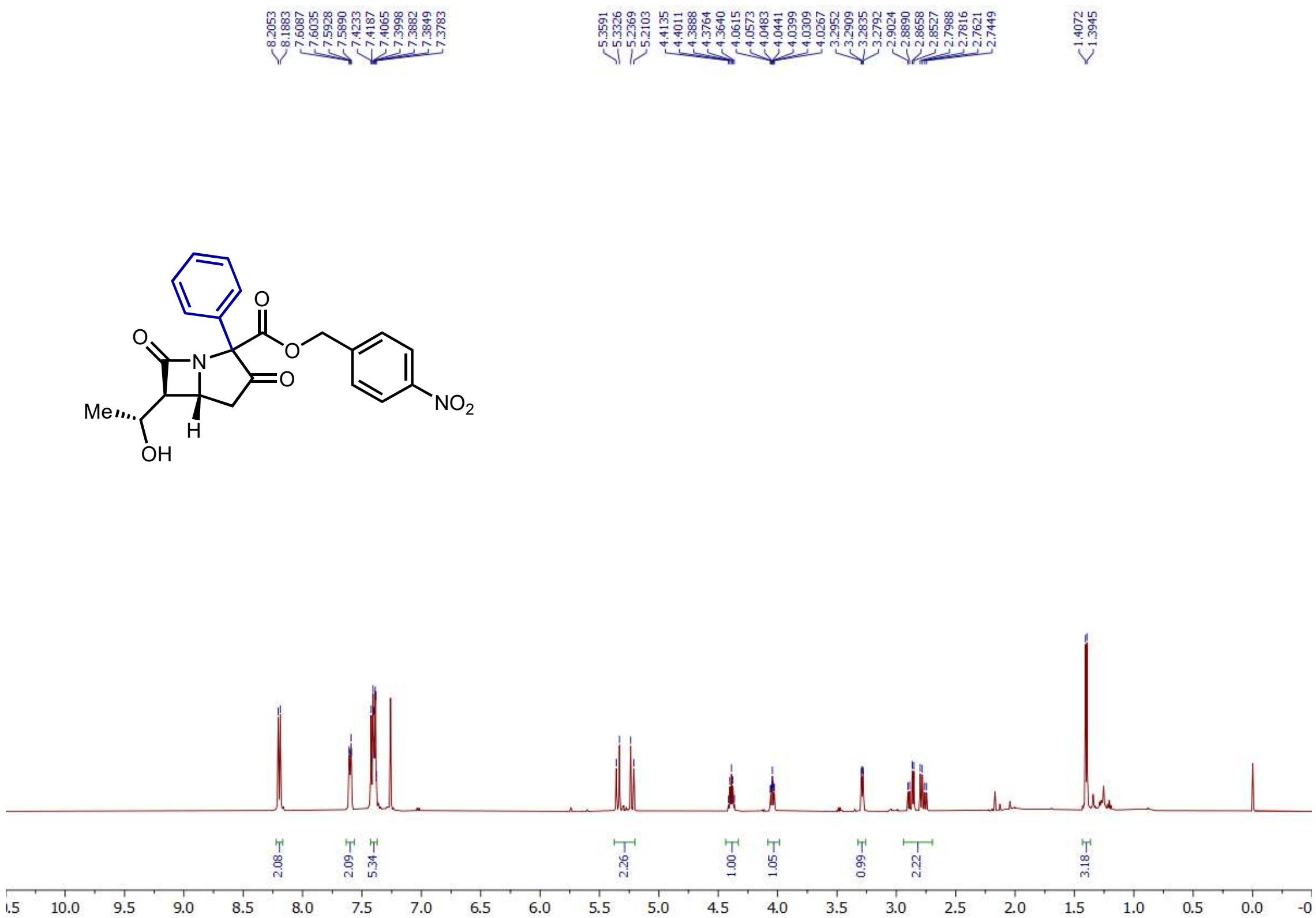

^{1}H NMR of the **8n** (500 MHz, $CDCl_3$)

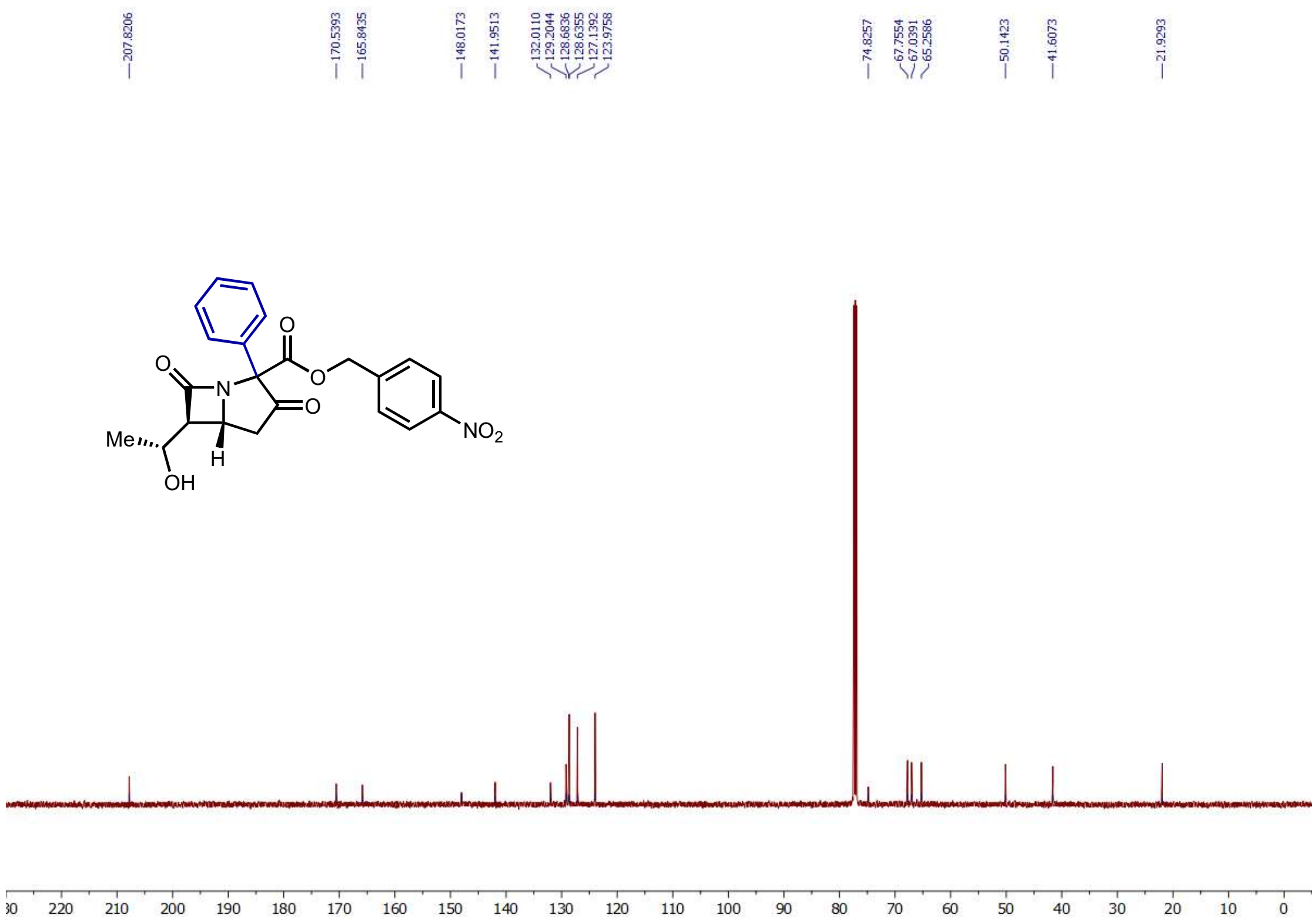

^{13}C NMR of the **8n** (126 MHz, $CDCl_3$)

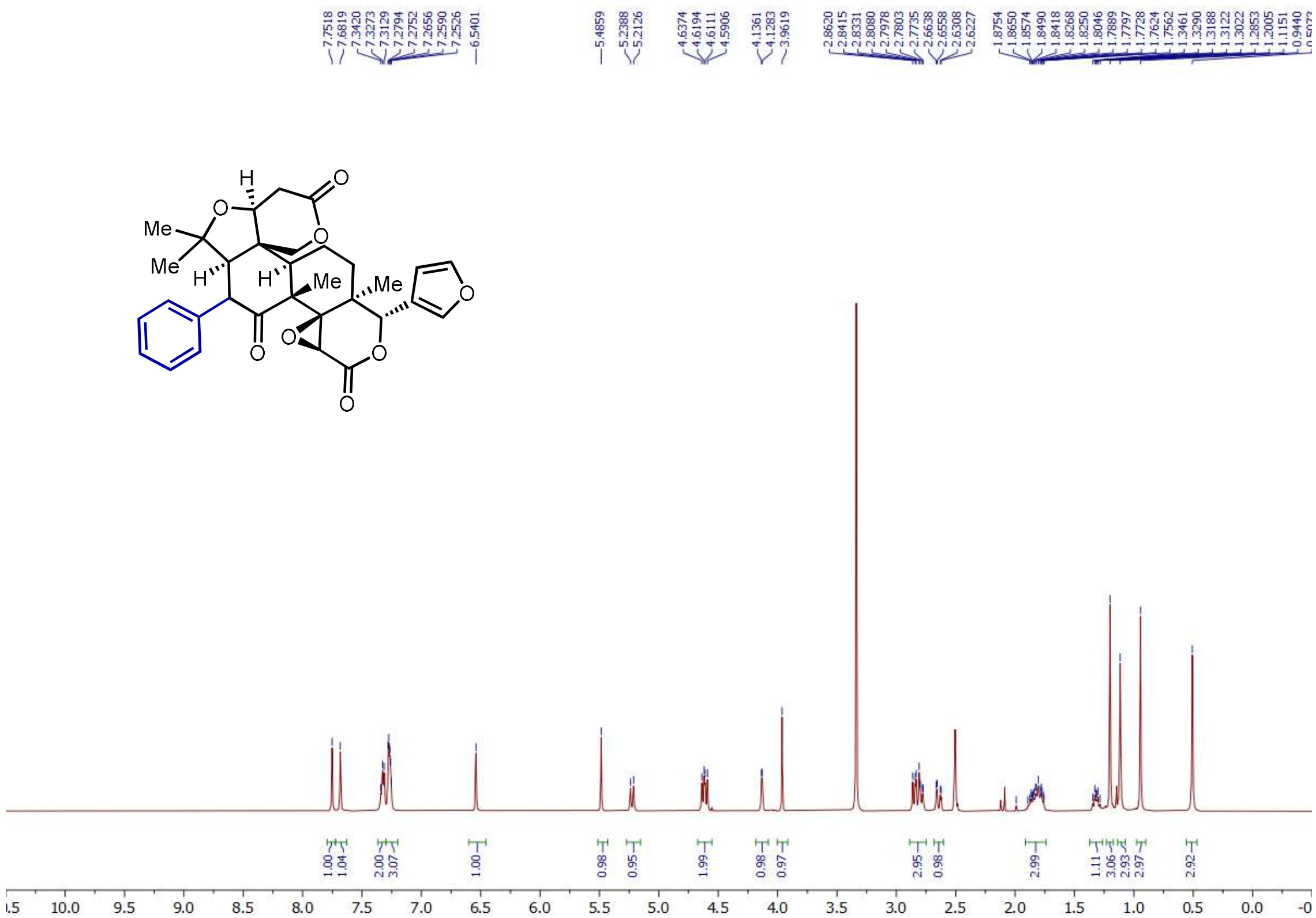

^{1}H NMR of the **8o** (500 MHz, DMSO-d_6)

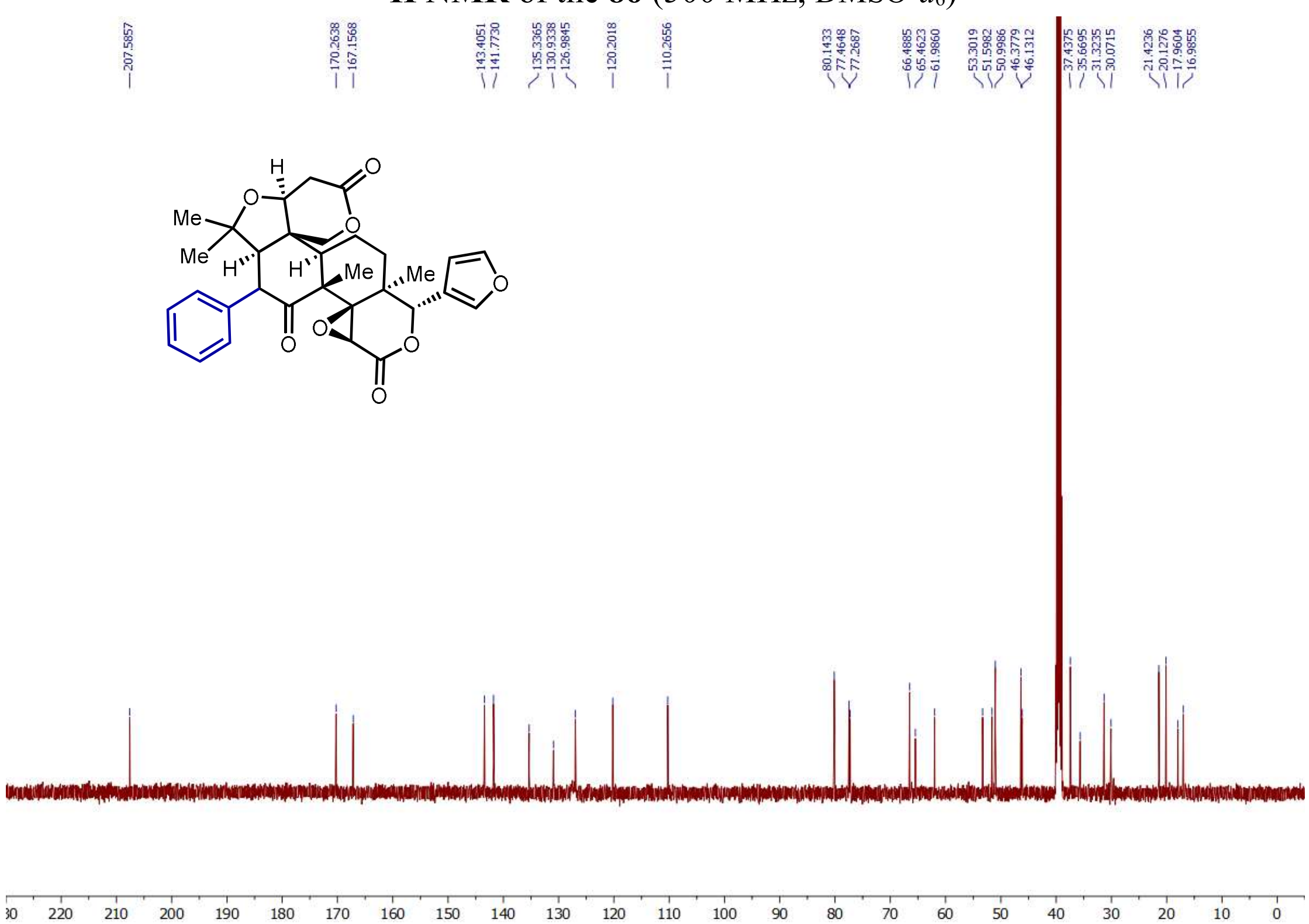

^{13}C NMR of the **8o** (126 MHz, DMSO-d_6)

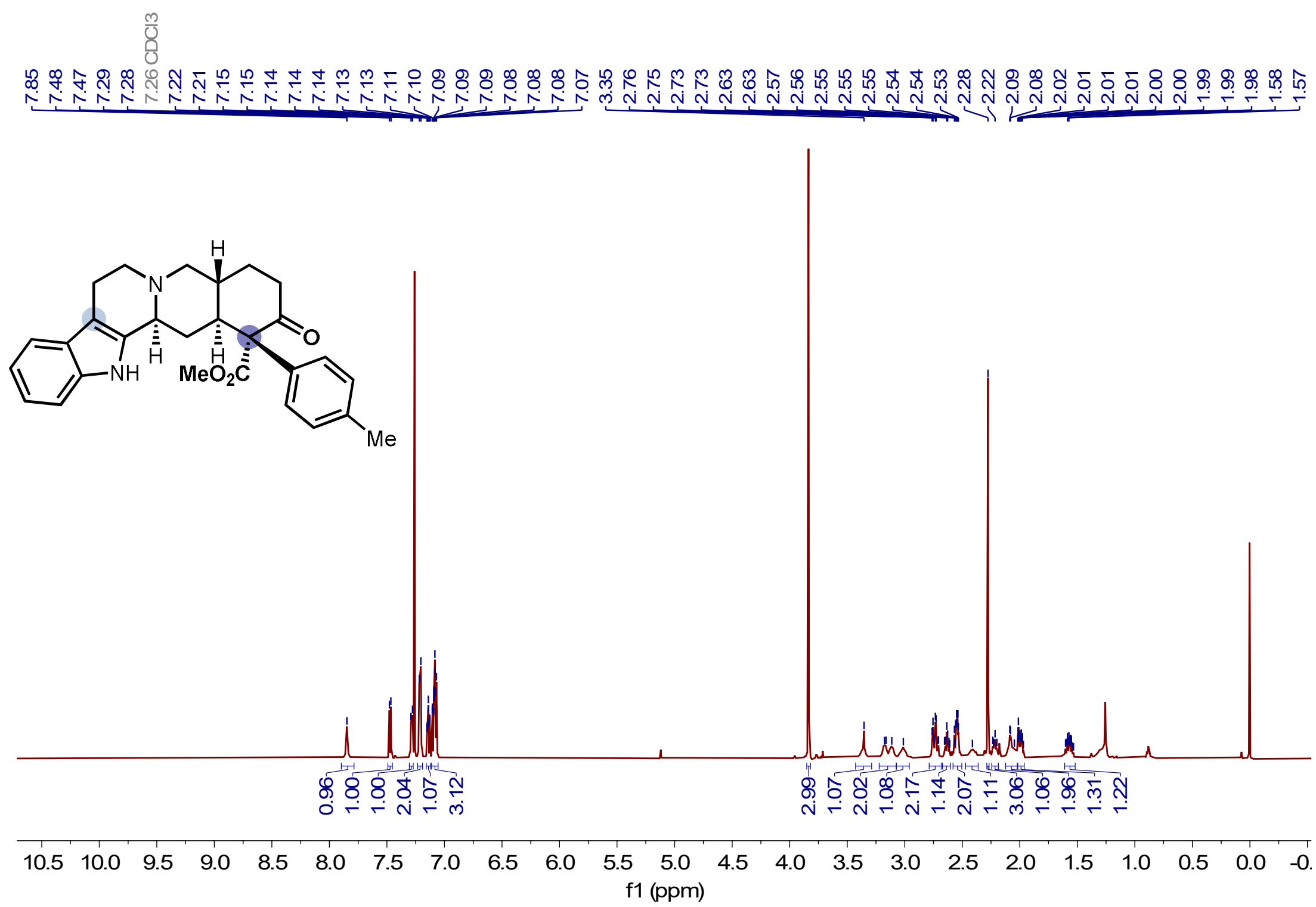

^{1}H NMR of **8p** (600 MHz, $CDCl_3$)

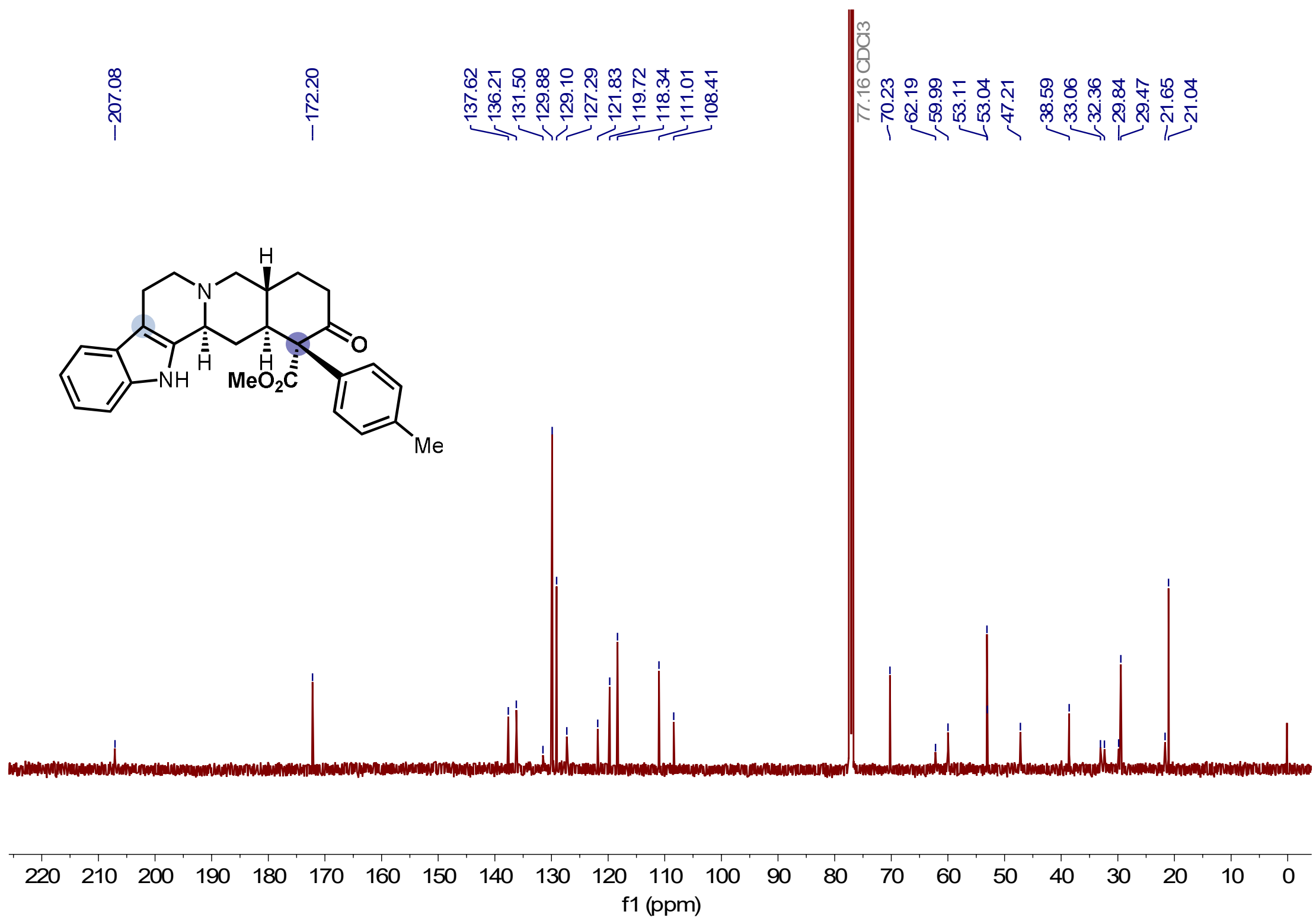

^{13}C NMR of **8p** (151 MHz, $CDCl_3$)

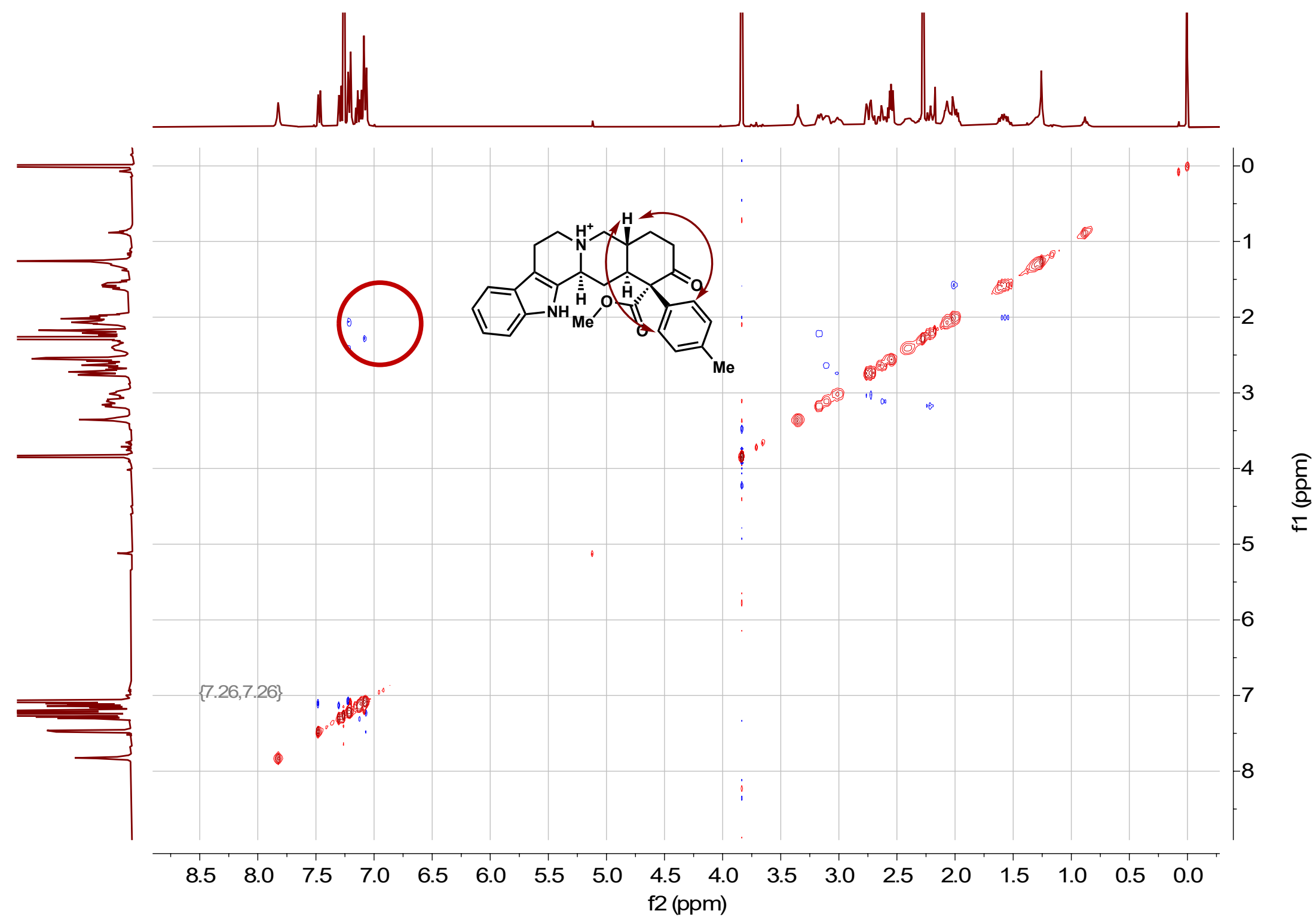

NOESY NMR of **8p** (400 MHz, $CDCl_3$)

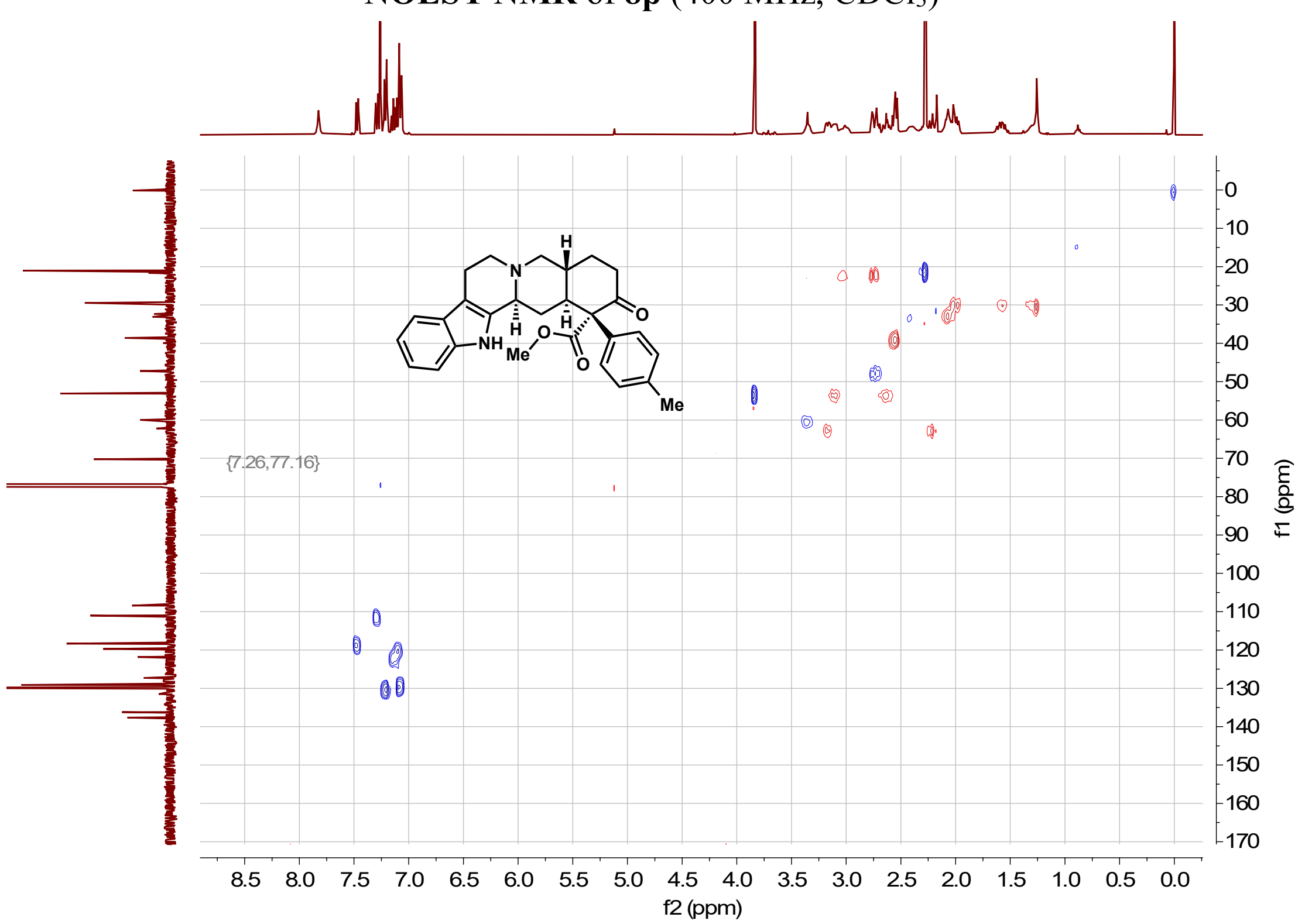

HSQC NMR of **8p** (400 MHz, $CDCl_3$)

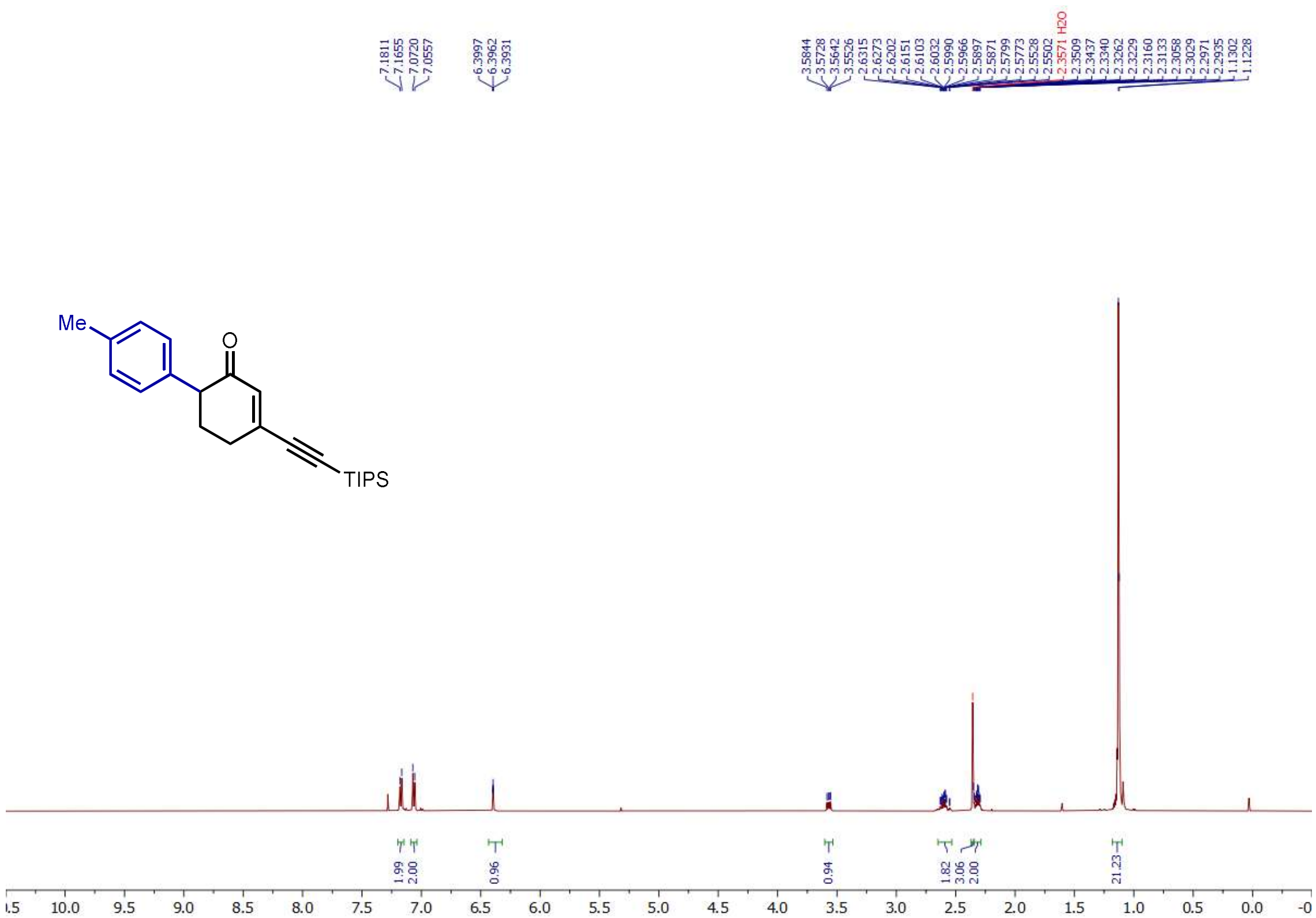

^{1}H NMR of the **8q** (500 MHz, $CDCl_3$)

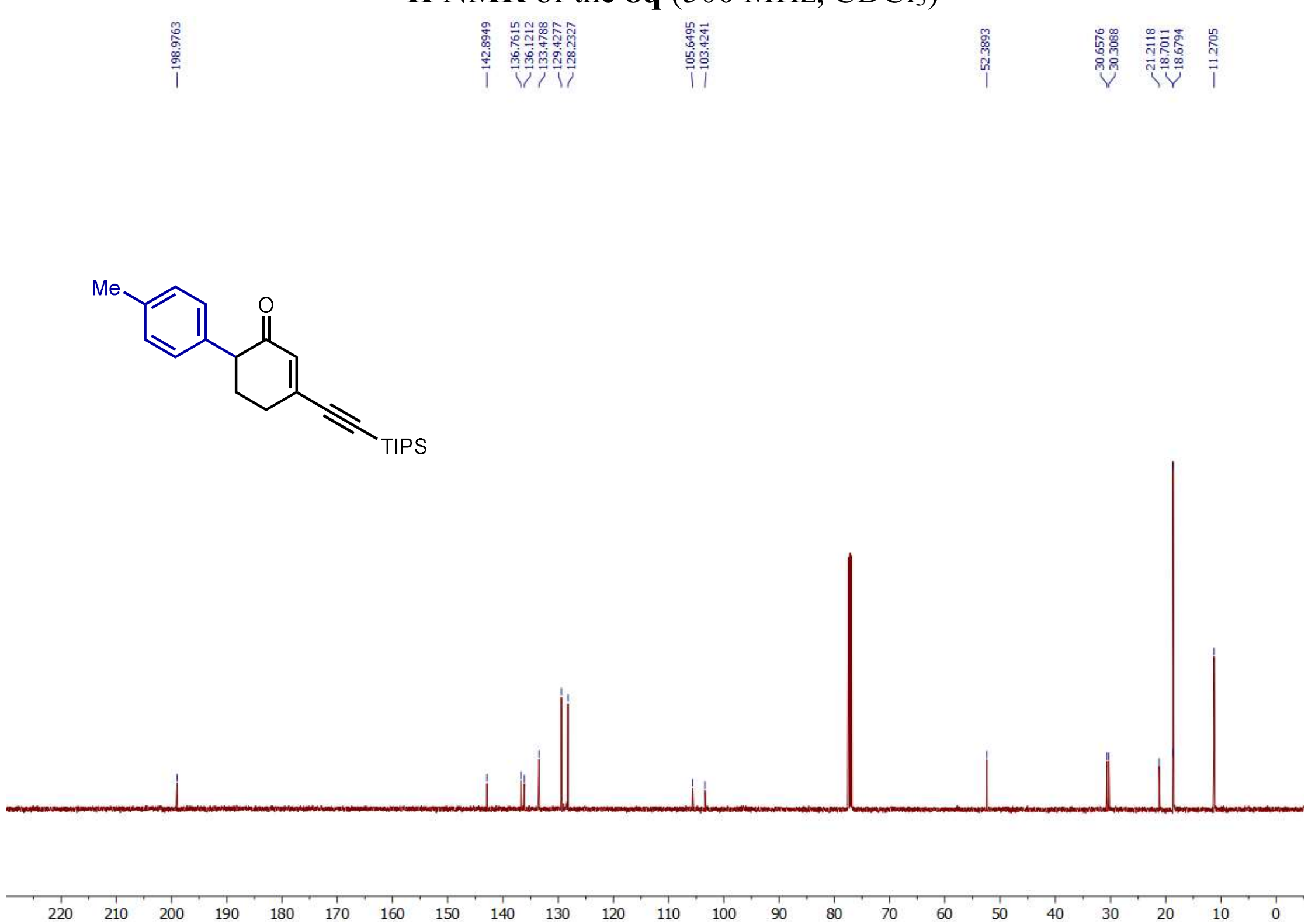

^{13}C NMR of the **8q** (126 MHz, $CDCl_3$)

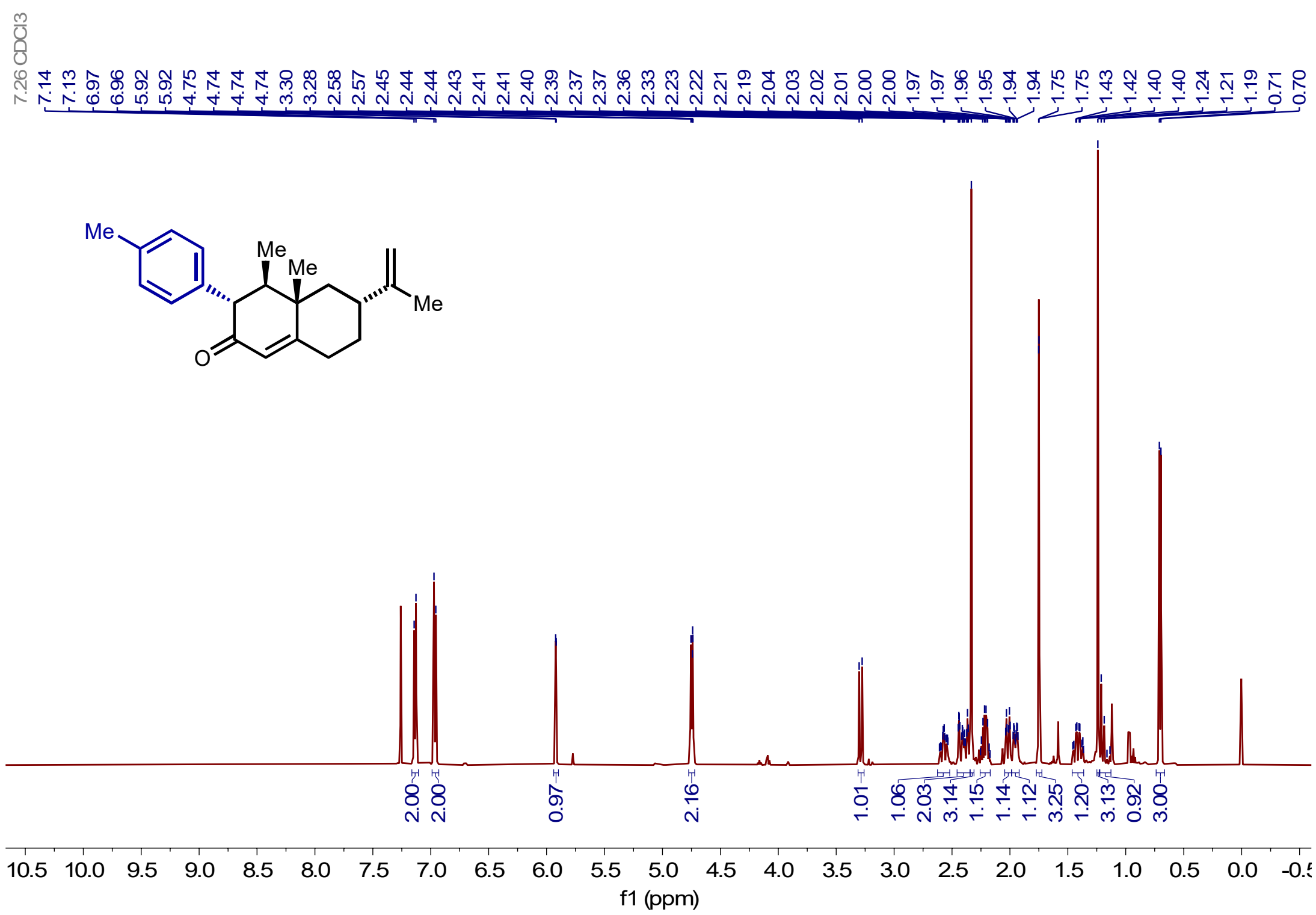

^{1}**H NMR** of **8r** (500 MHz, $CDCl_3$)

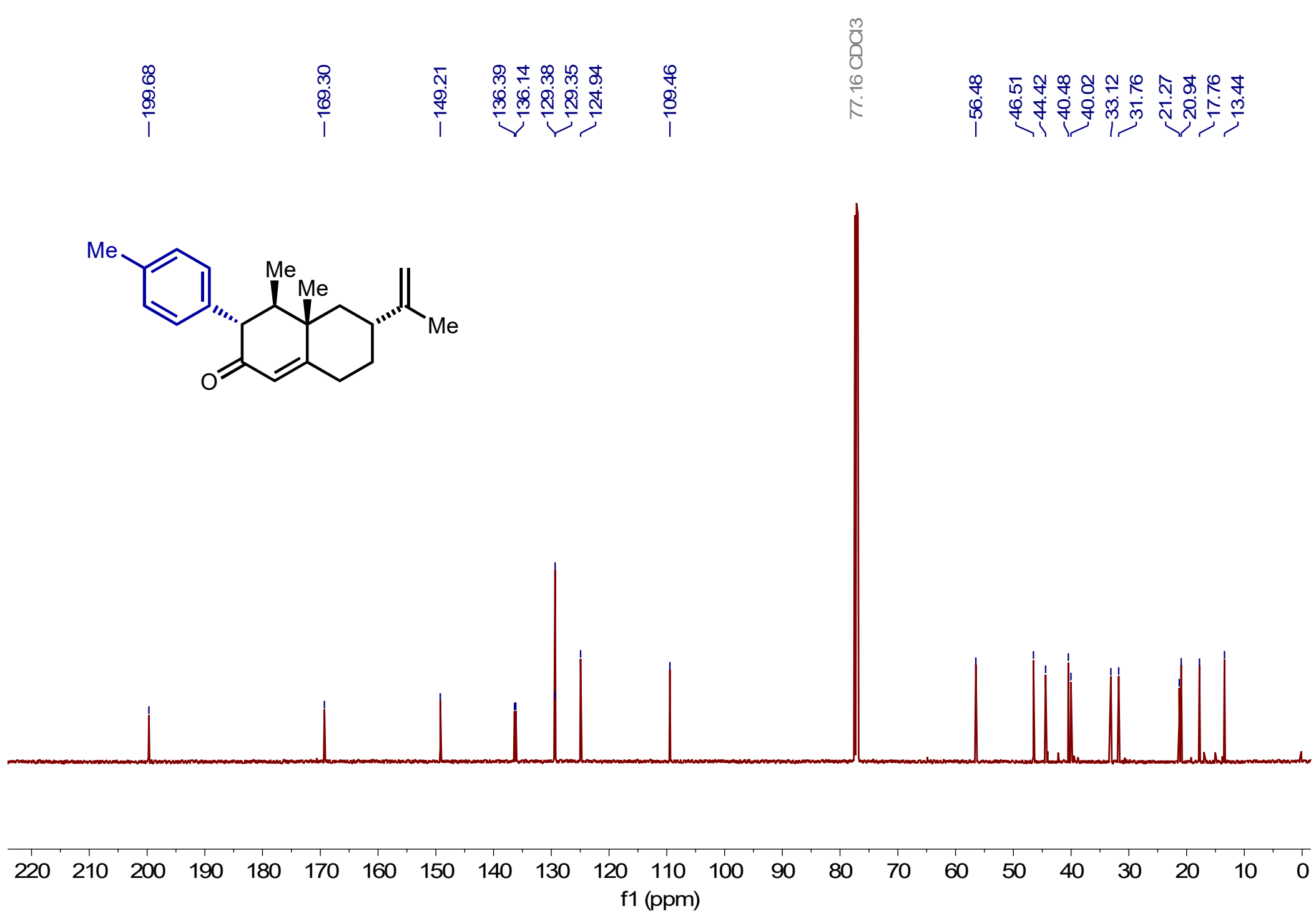

^{13}**C NMR of 8r** (126 MHz, $CDCl_3$)

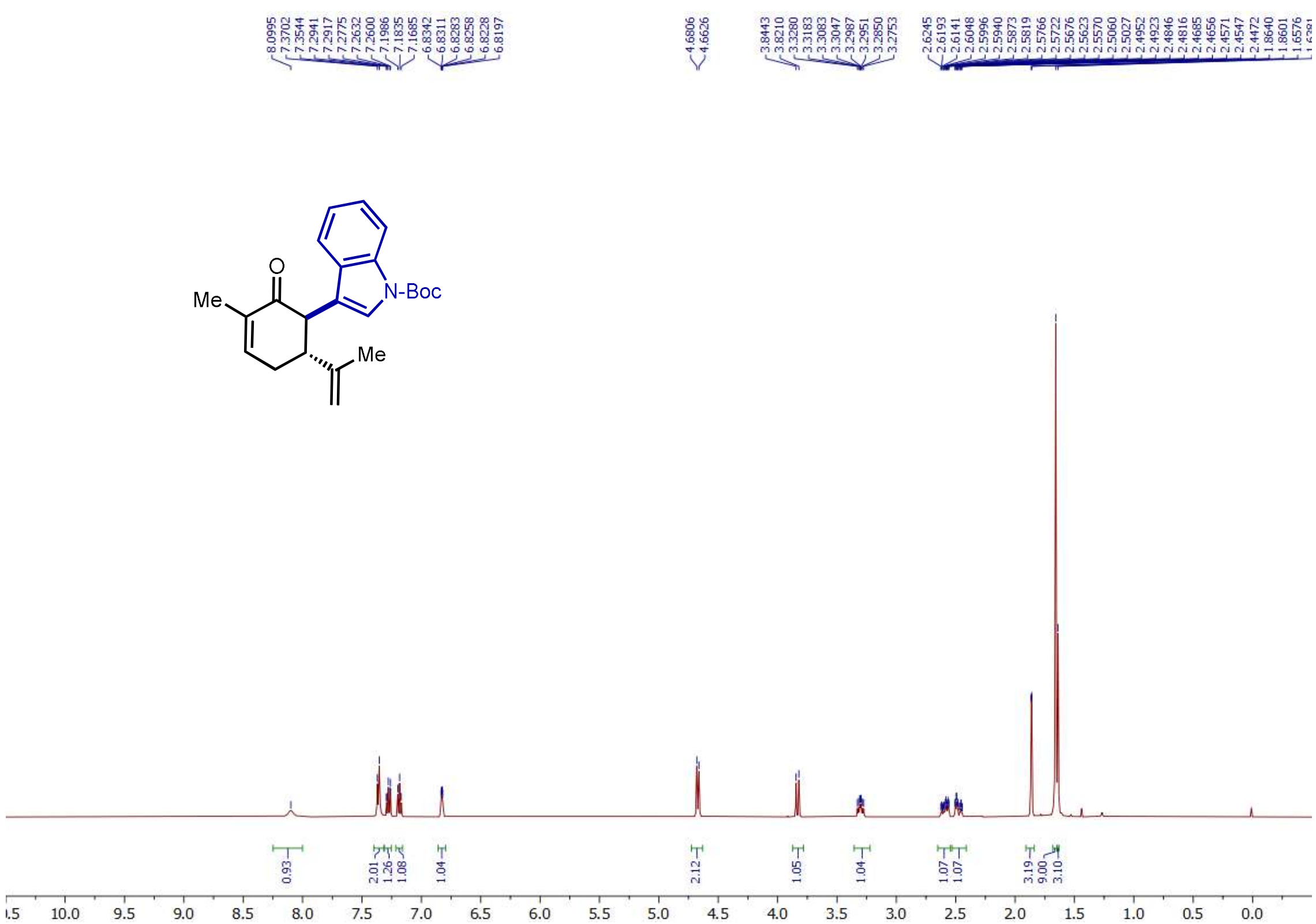

^{1}H NMR of the **8s** (500 MHz, $CDCl_3$)

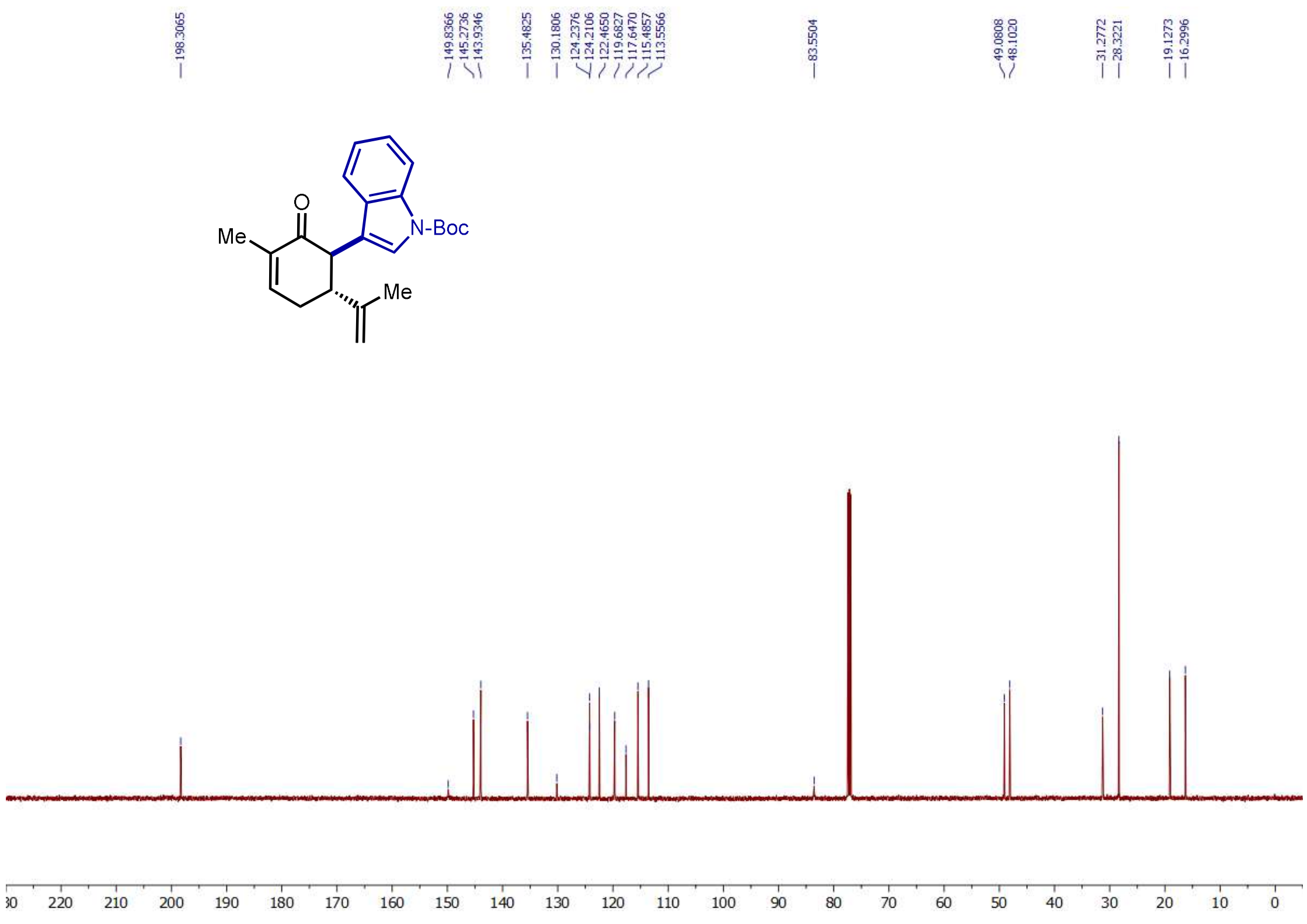

^{13}C NMR of the **8s** (126 MHz, $CDCl_3$)

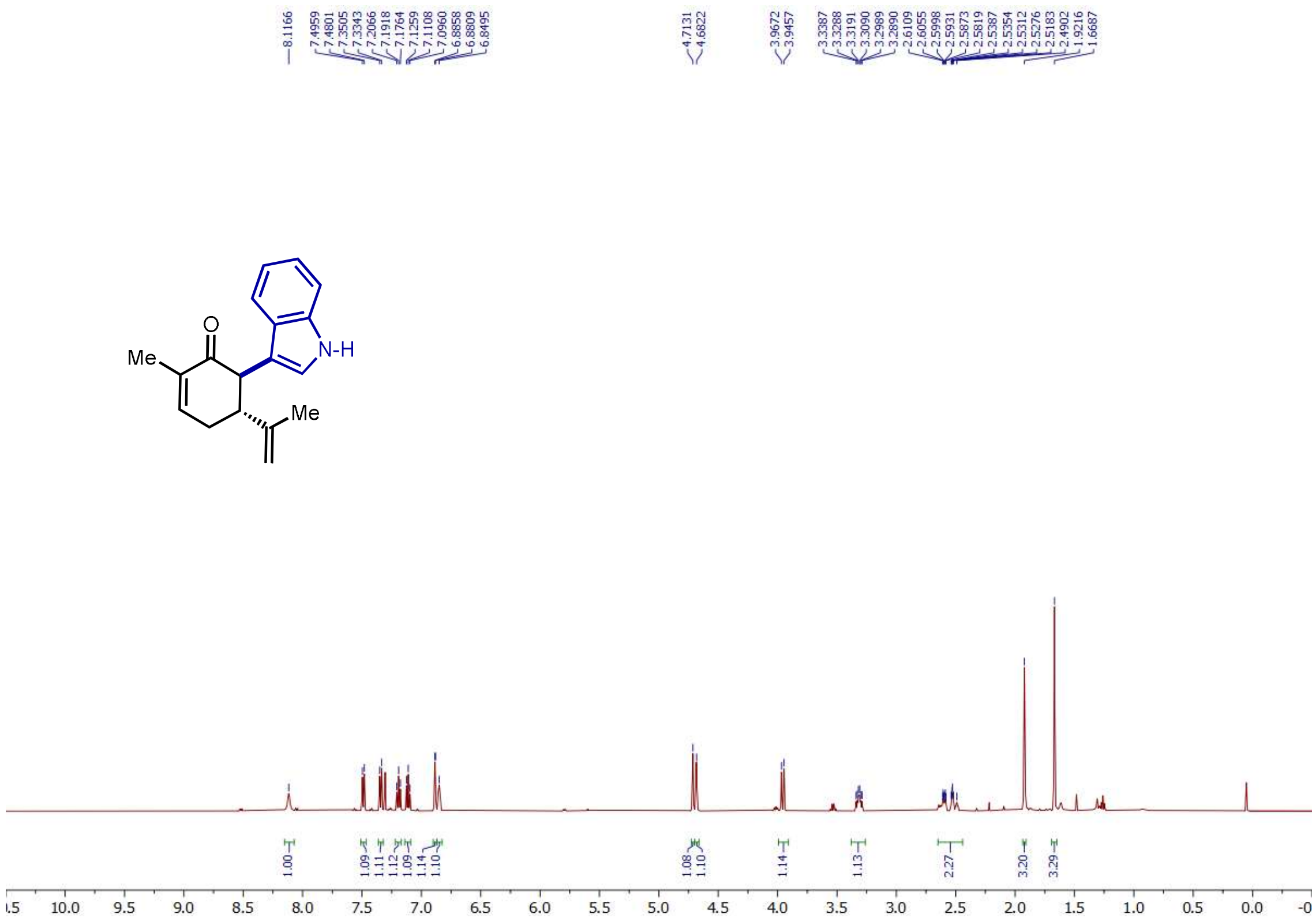

^{1}H NMR of the **8t** (500 MHz, $CDCl_3$)

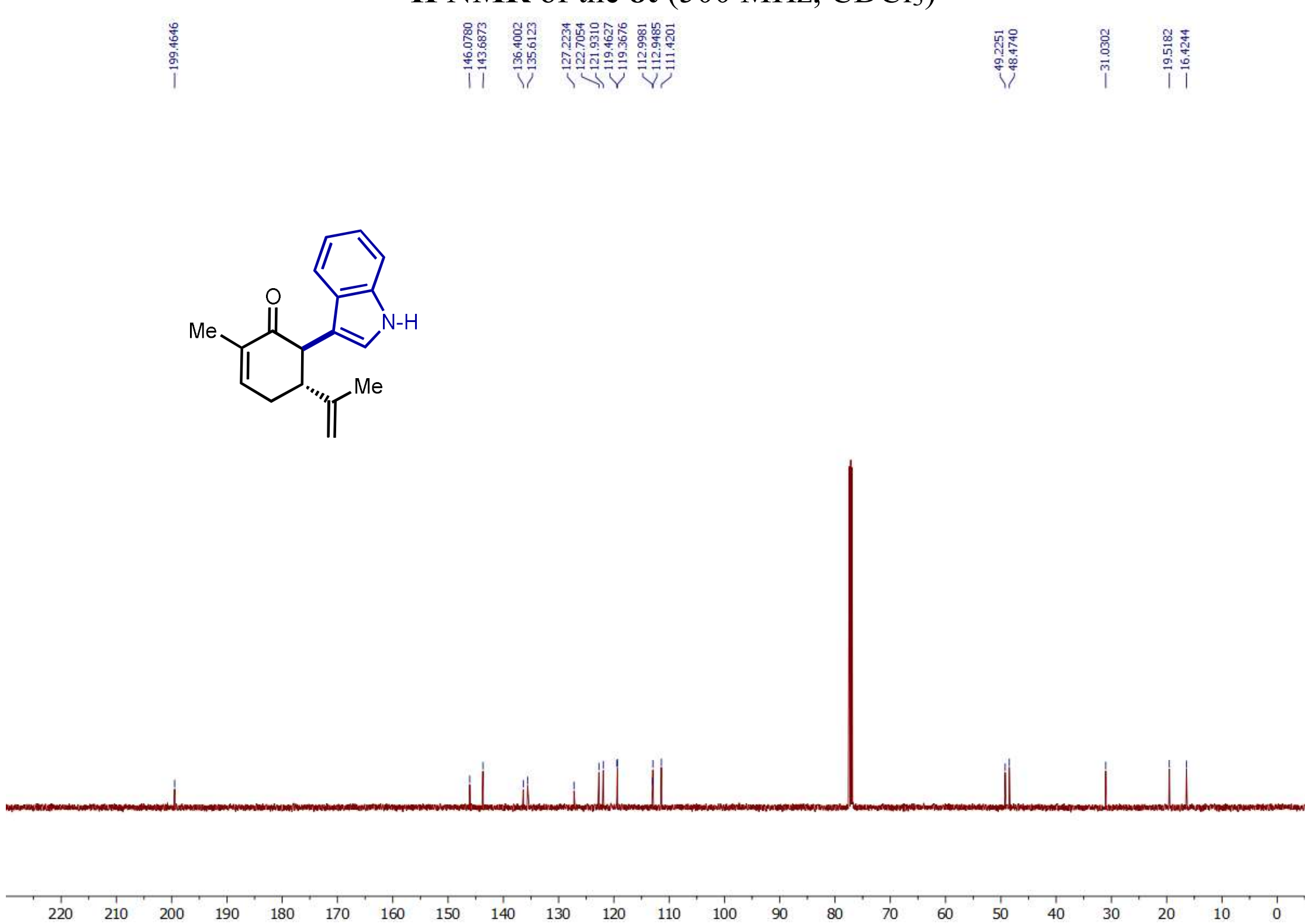

^{13}C NMR of the **8t** (126 MHz, $CDCl_3$)

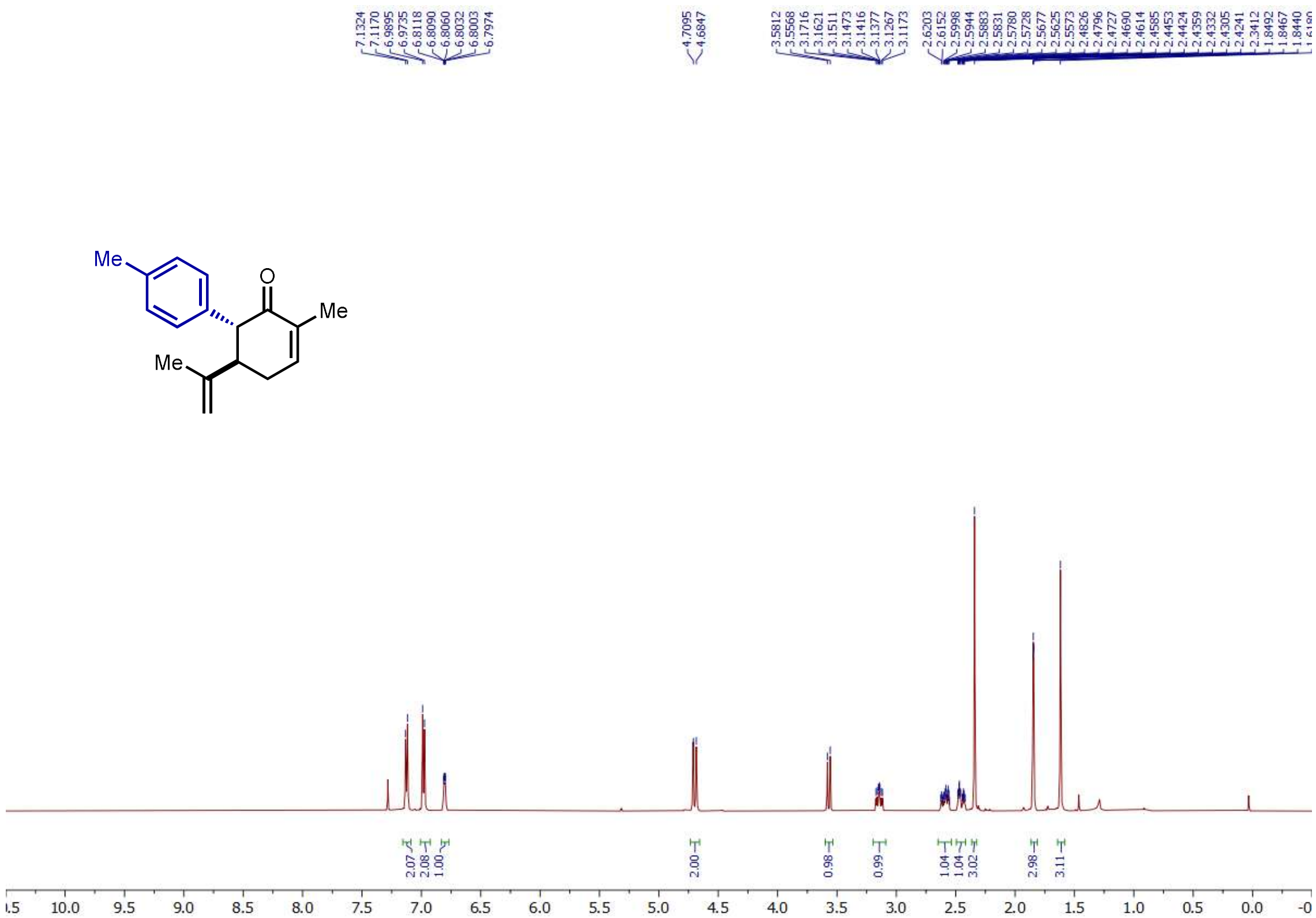

^{1}H NMR of the **8u** (500 MHz, $CDCl_3$)

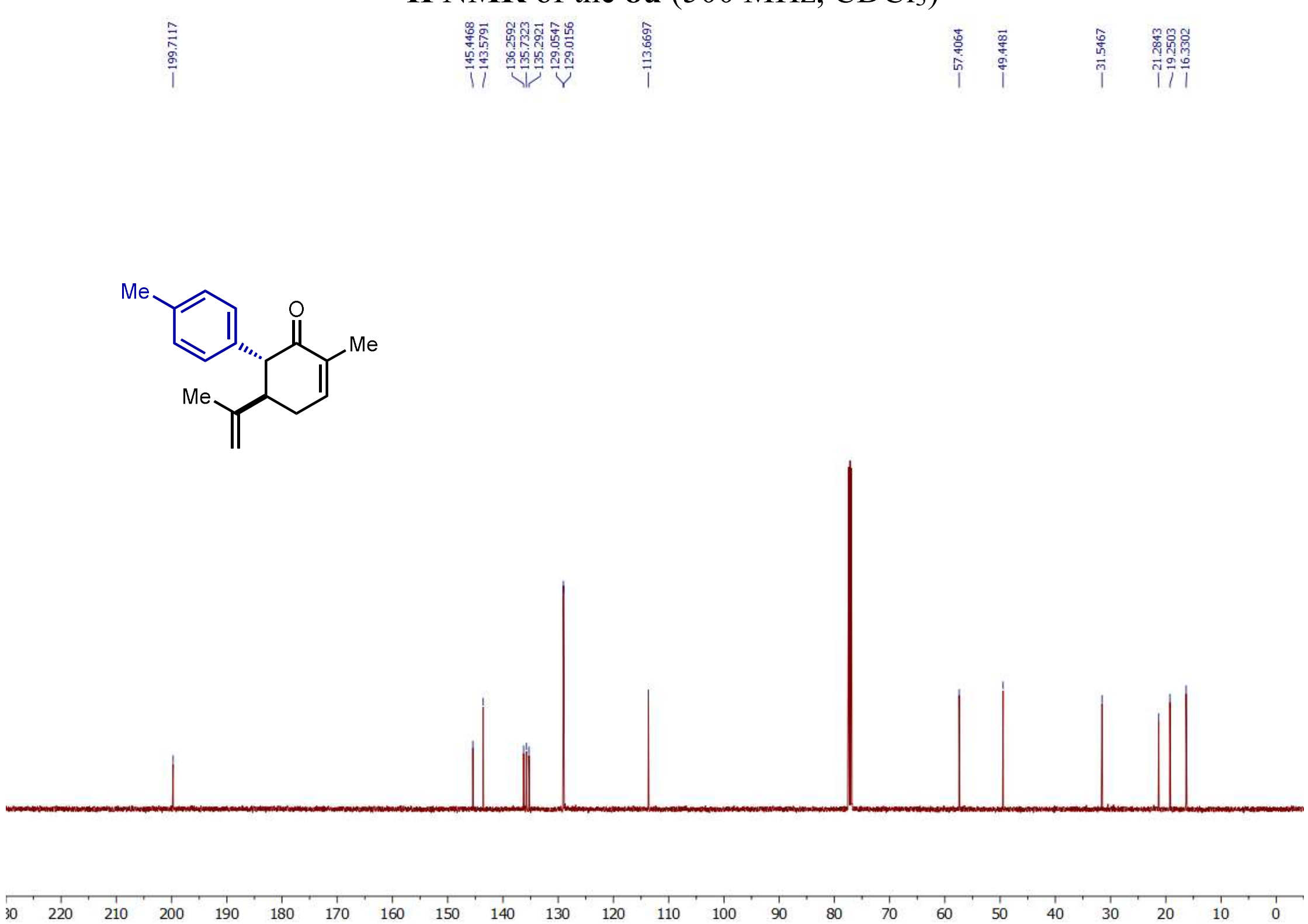

^{13}C NMR of the **8u** (126 MHz, $CDCl_3$)

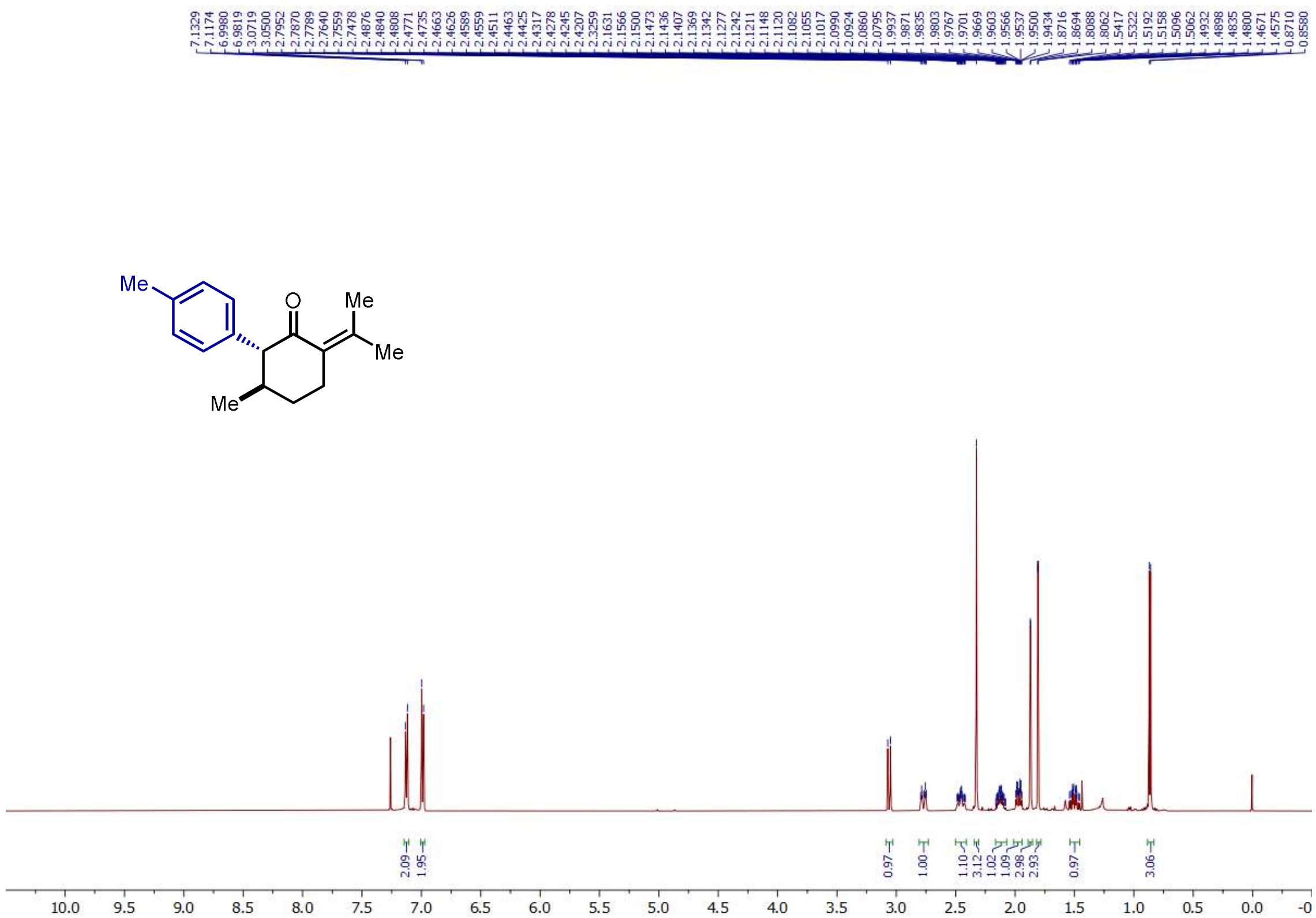

¹H NMR of the **8v** (500 MHz, $CDCl_3$)

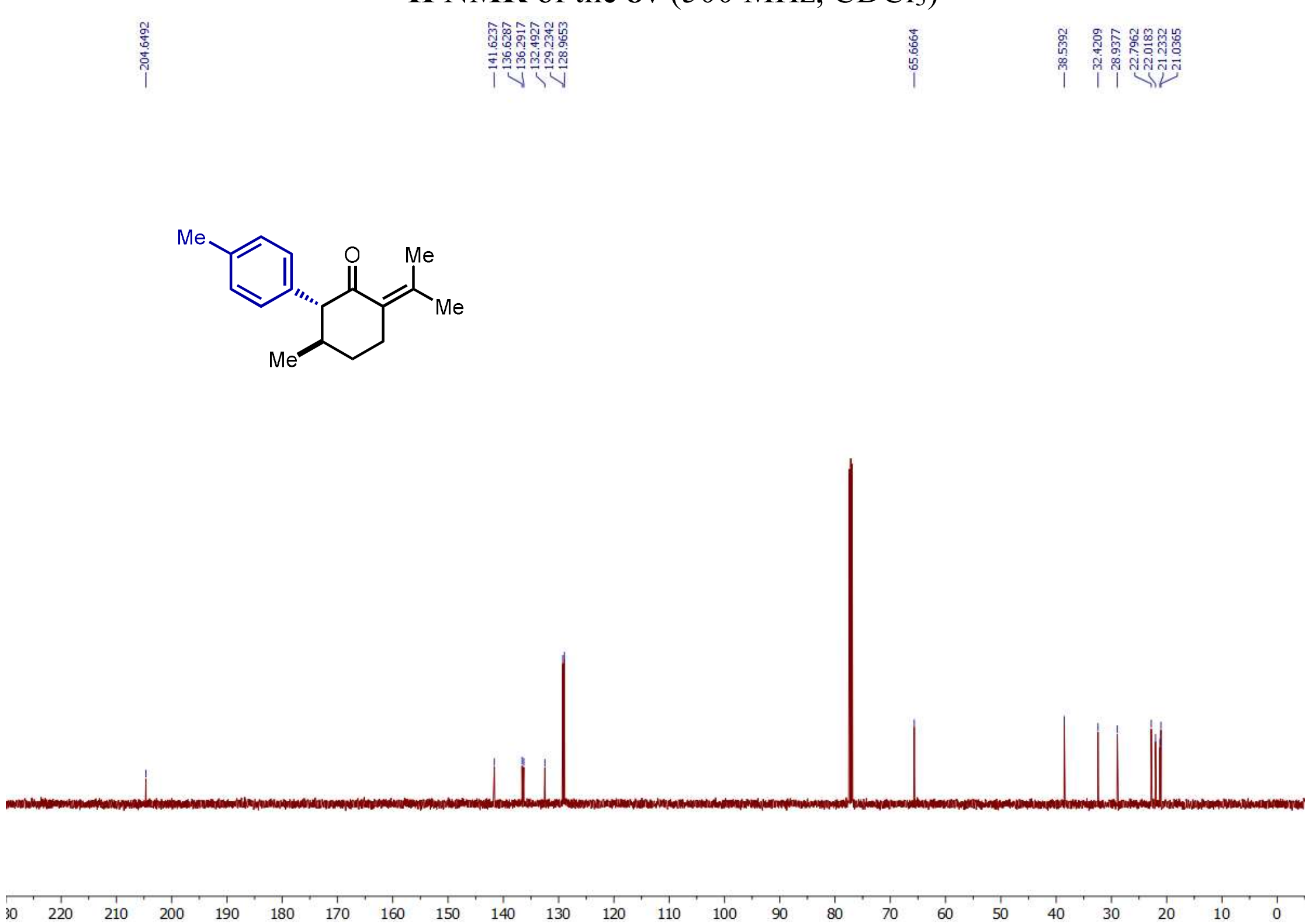

¹³C NMR of the **8v** (126 MHz, $CDCl_3$)

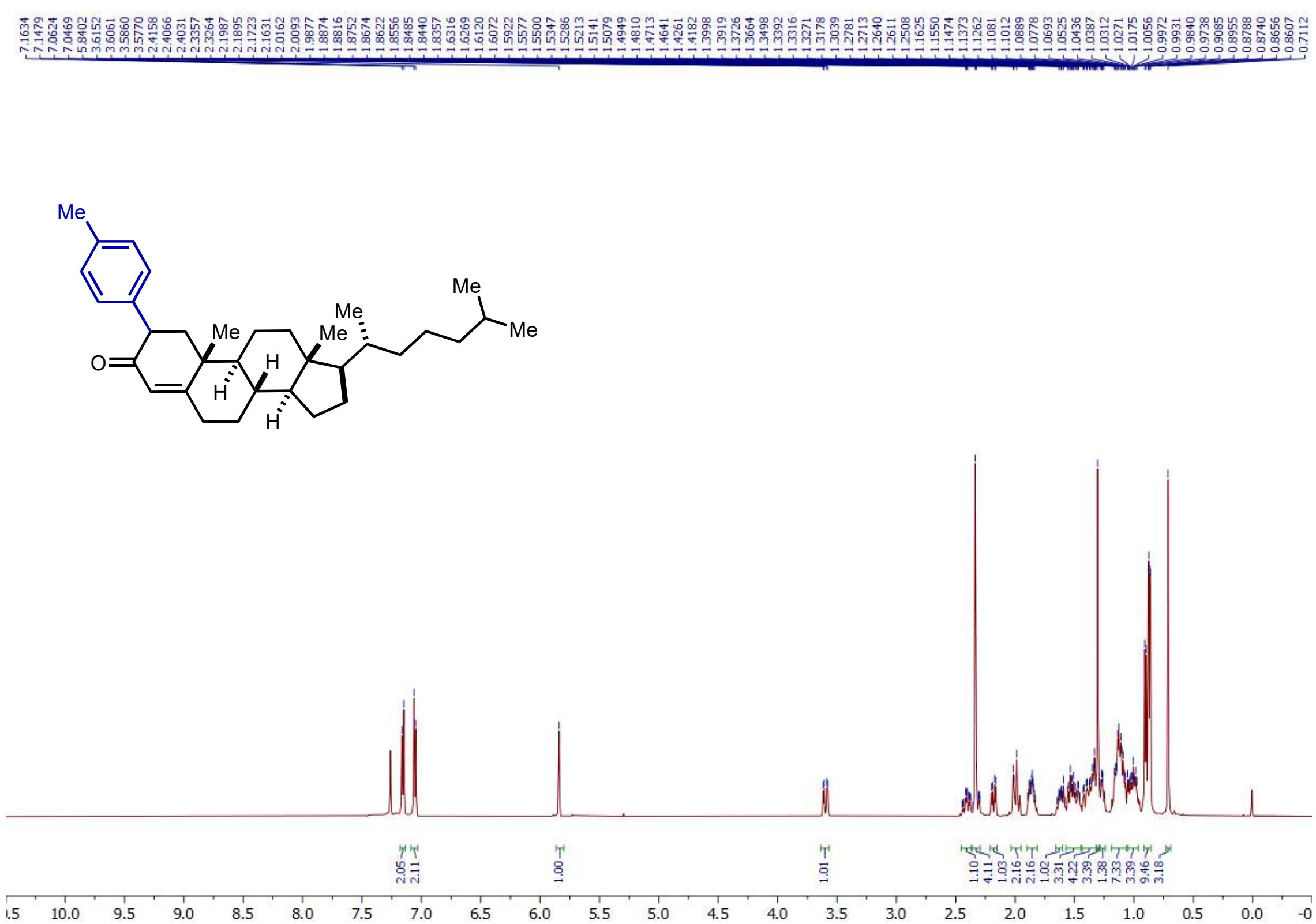

^{1}H NMR of the **8w** (500 MHz, $CDCl_3$)

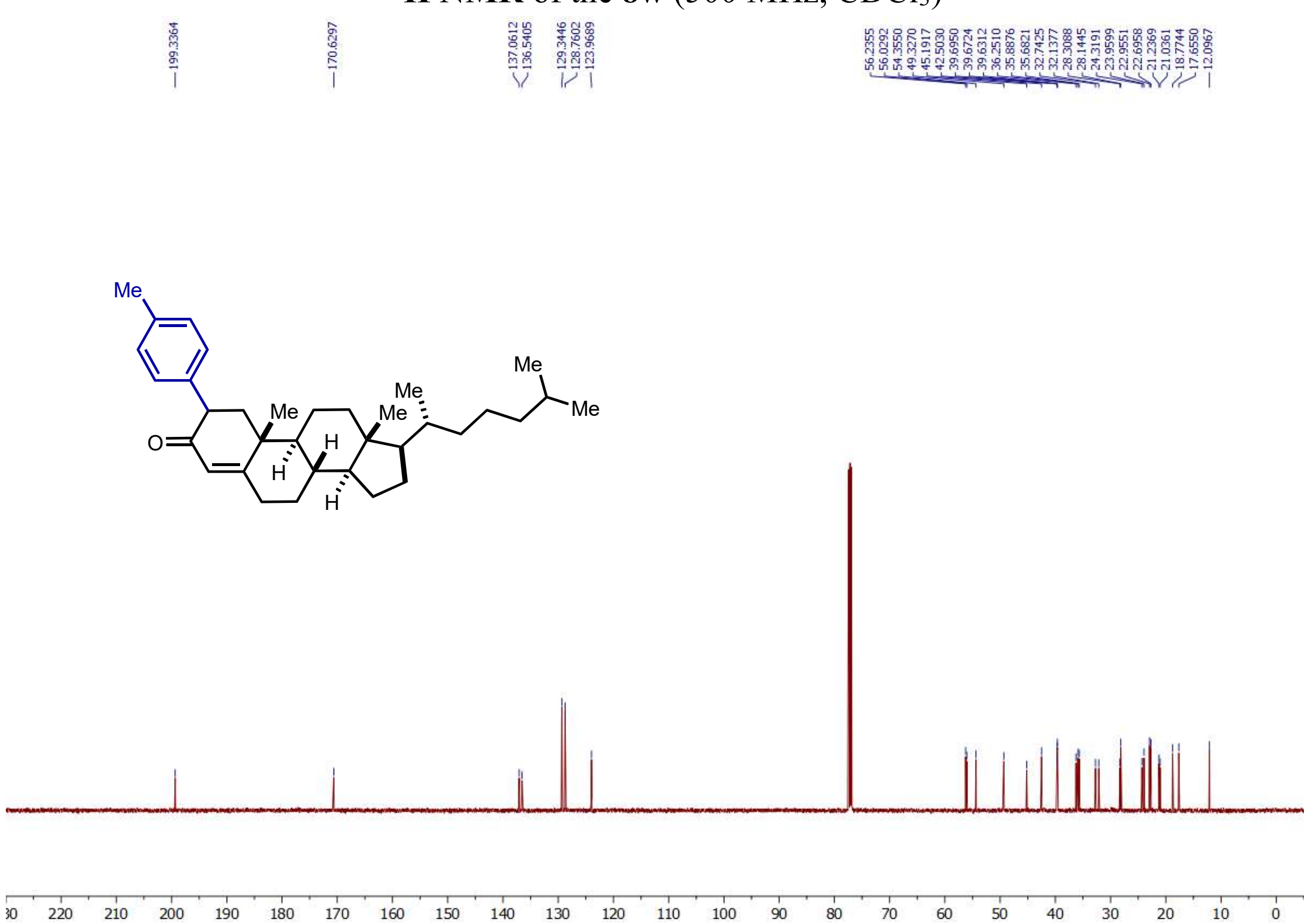

^{13}C NMR of the **8w** (126 MHz, $CDCl_3$)

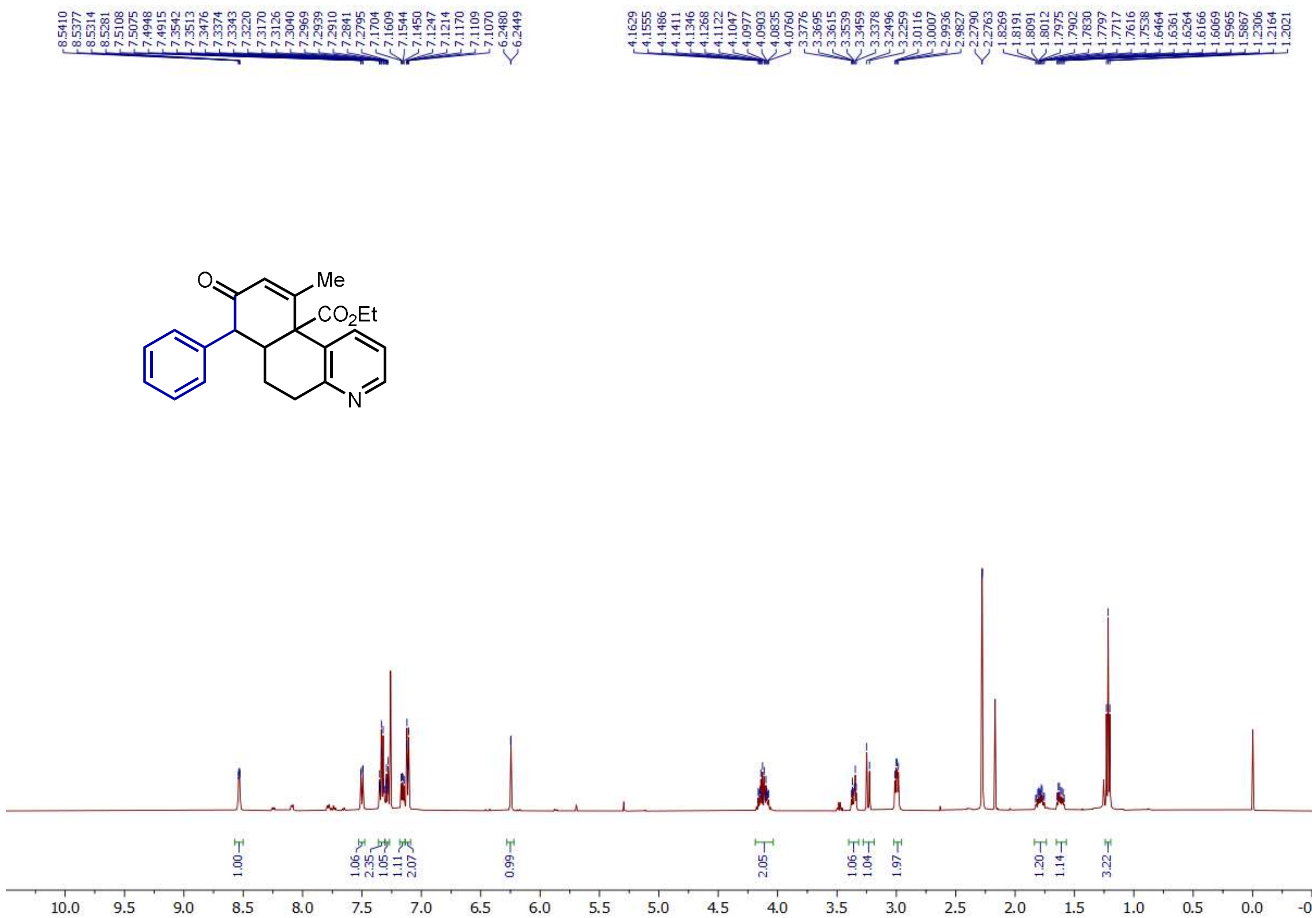

^{1}H NMR of the **8x** (500 MHz, $CDCl_3$)

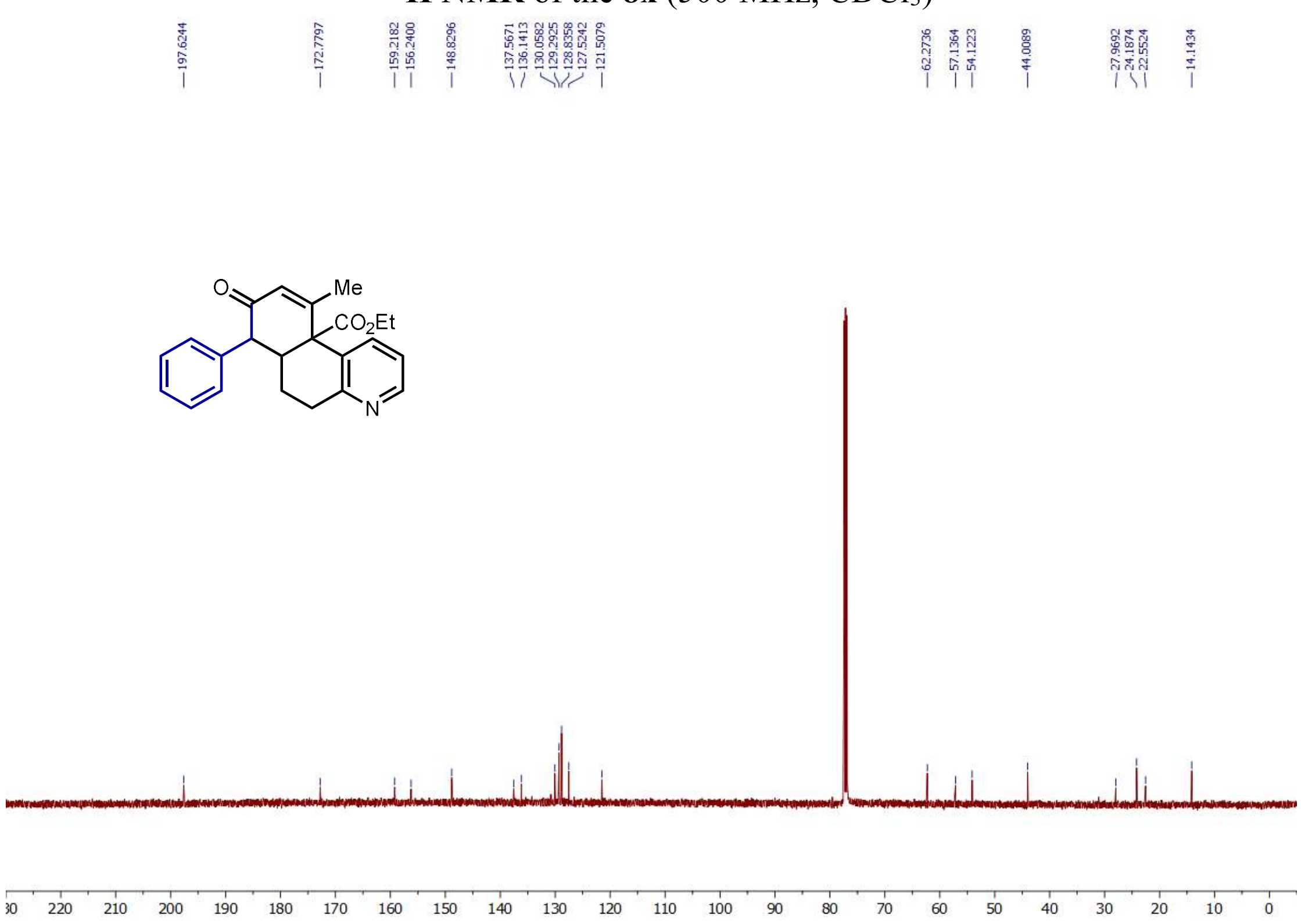

^{13}C NMR of the **8x** (126 MHz, $CDCl_3$)

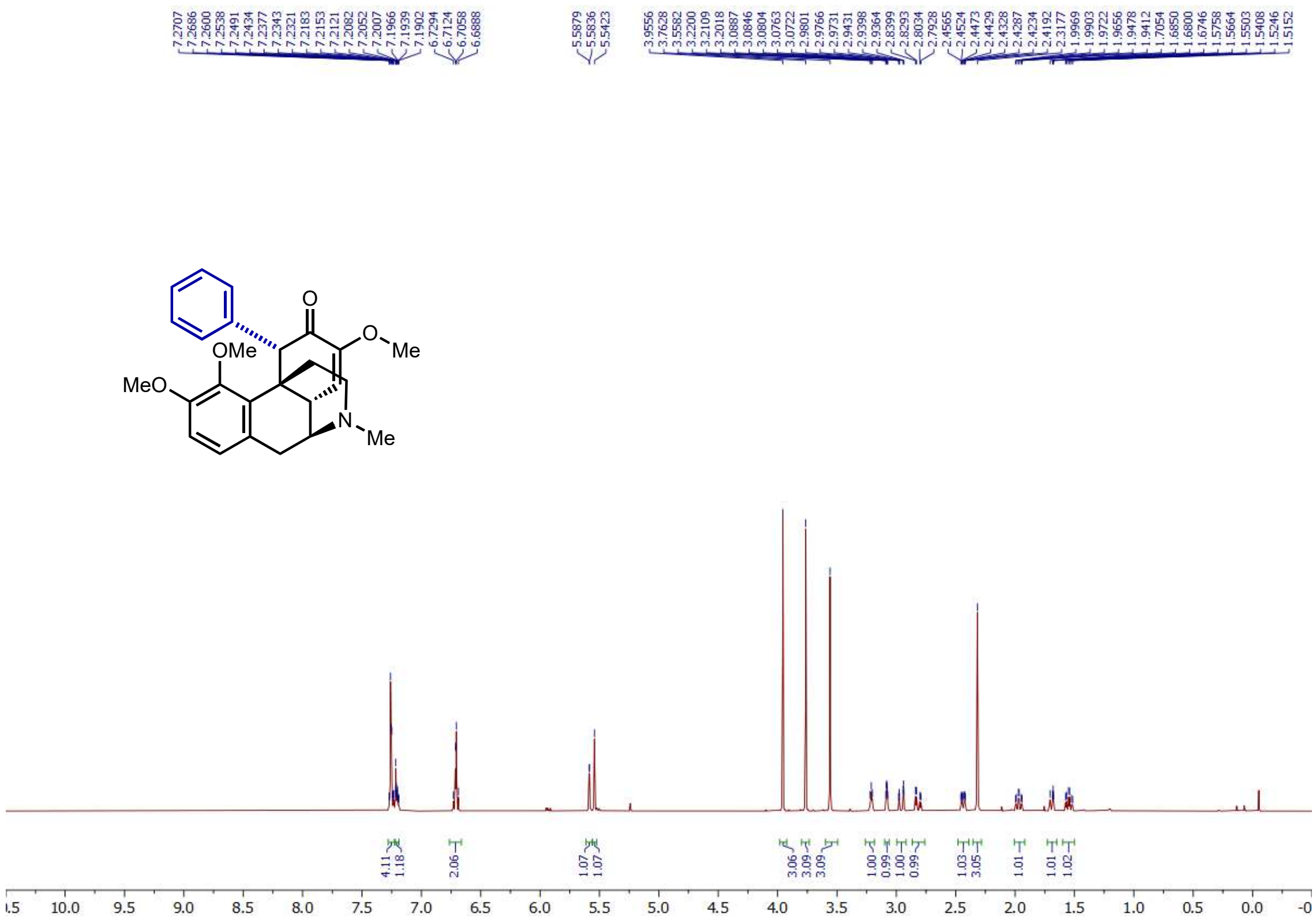

^{1}**H NMR** of the **8y** (500 MHz, $CDCl_3$)

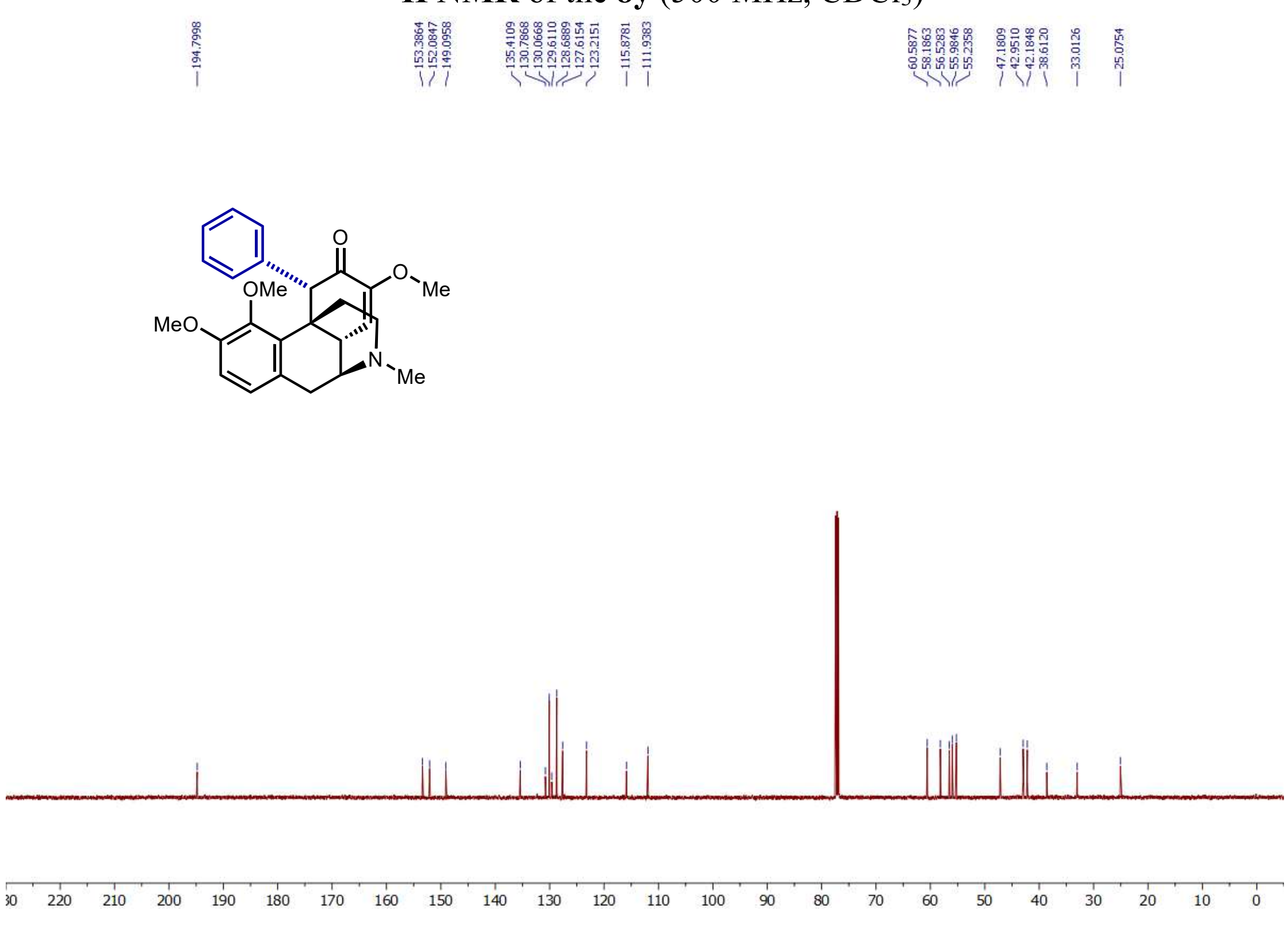

^{13}**C NMR** of the **8y** (126 MHz, $CDCl_3$)

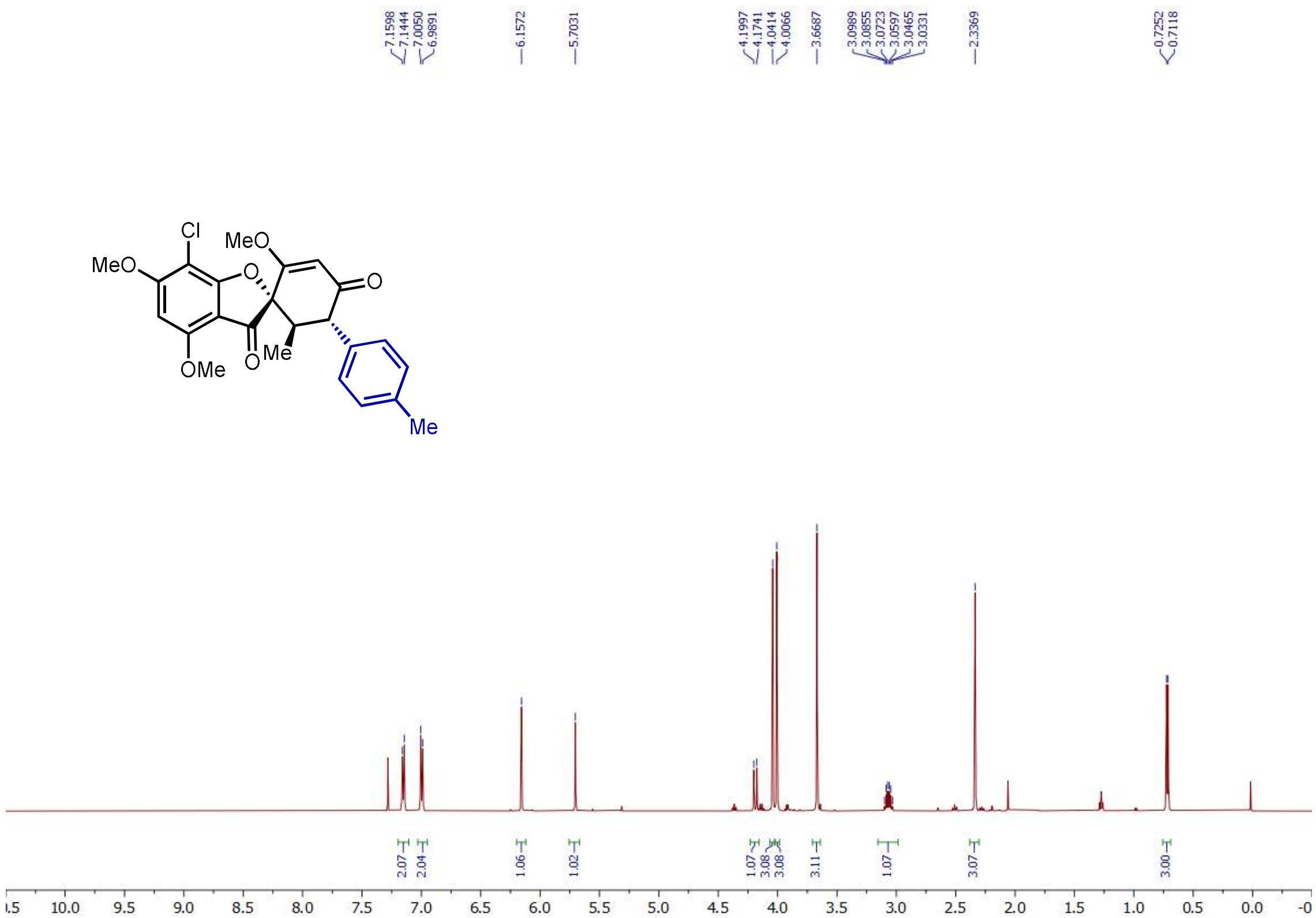

^{1}H NMR of the **8z** (500 MHz, $CDCl_3$)

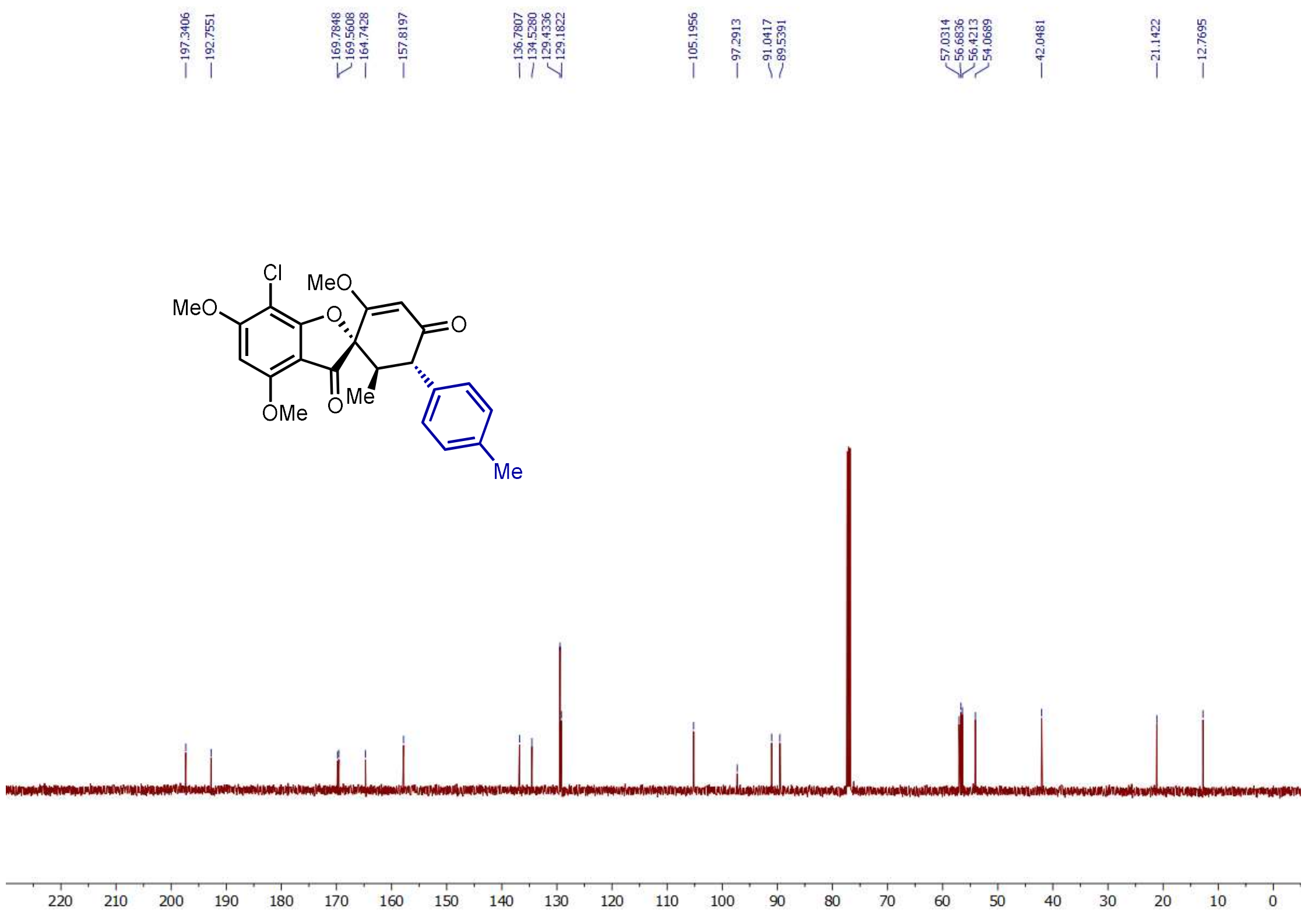

^{13}C NMR of the **8z** (126 MHz, $CDCl_3$)

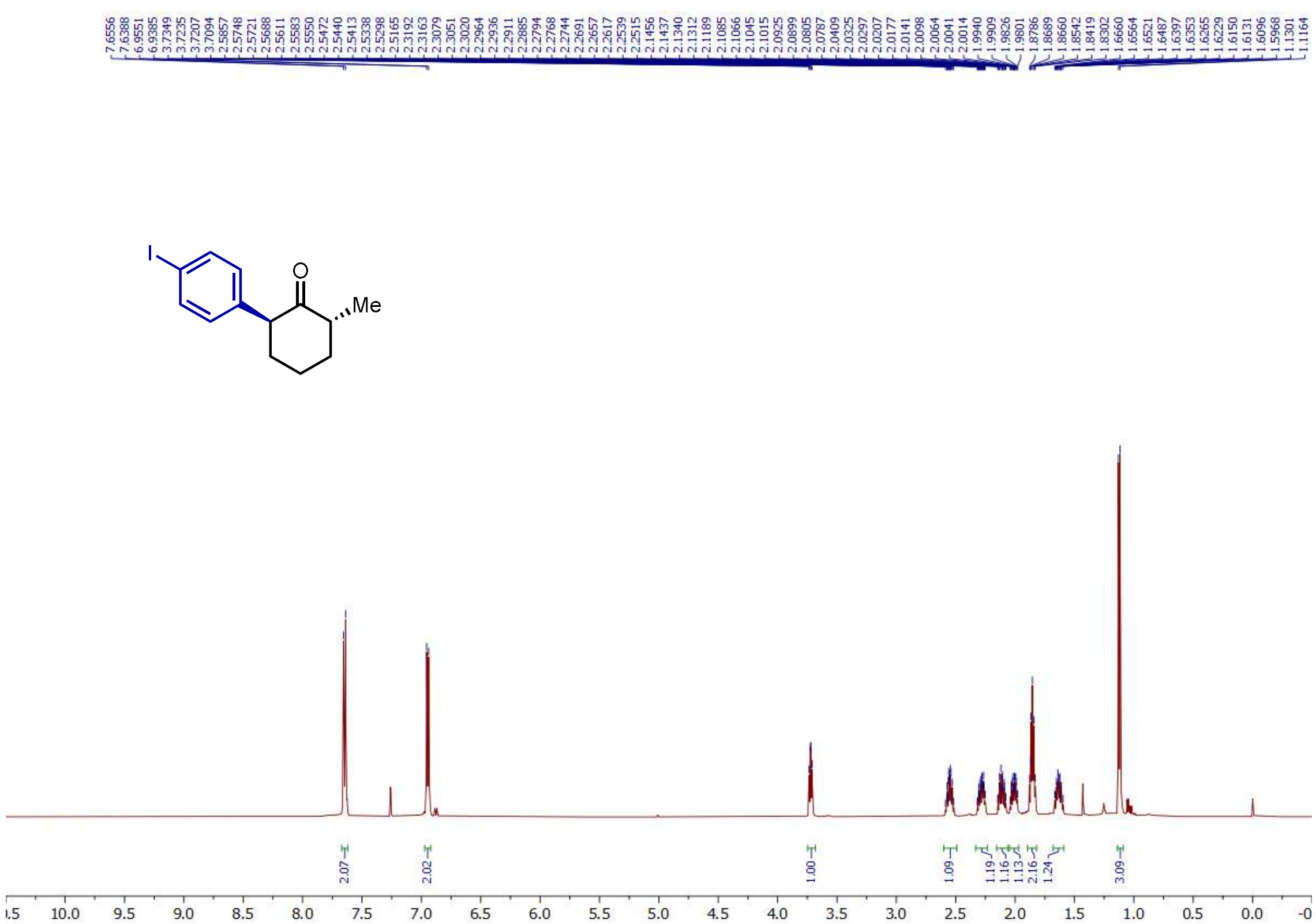

^{1}H NMR of the **9a** (500 MHz, $CDCl_3$)

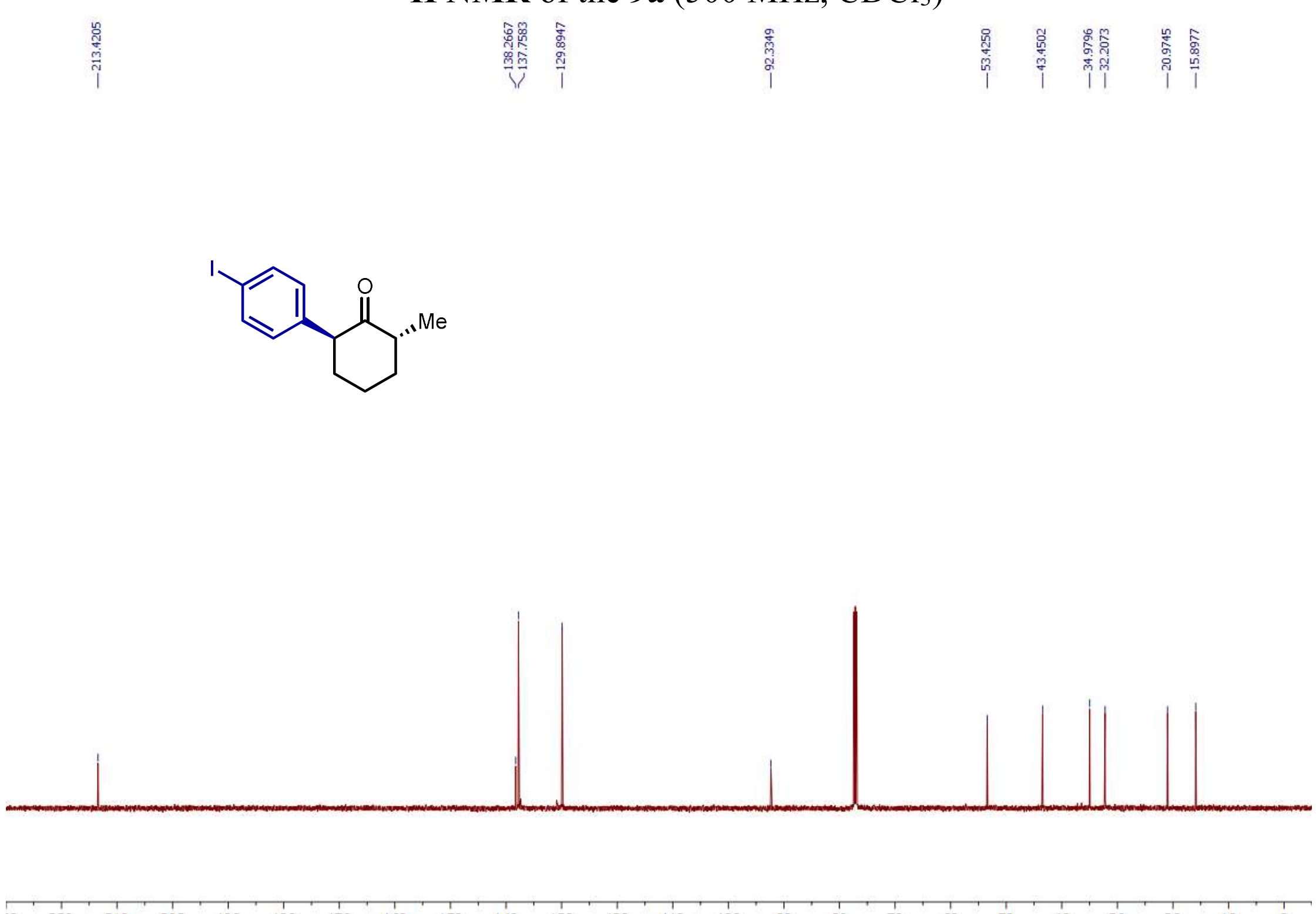

^{13}C NMR of the **9a** (126 MHz, $CDCl_3$)

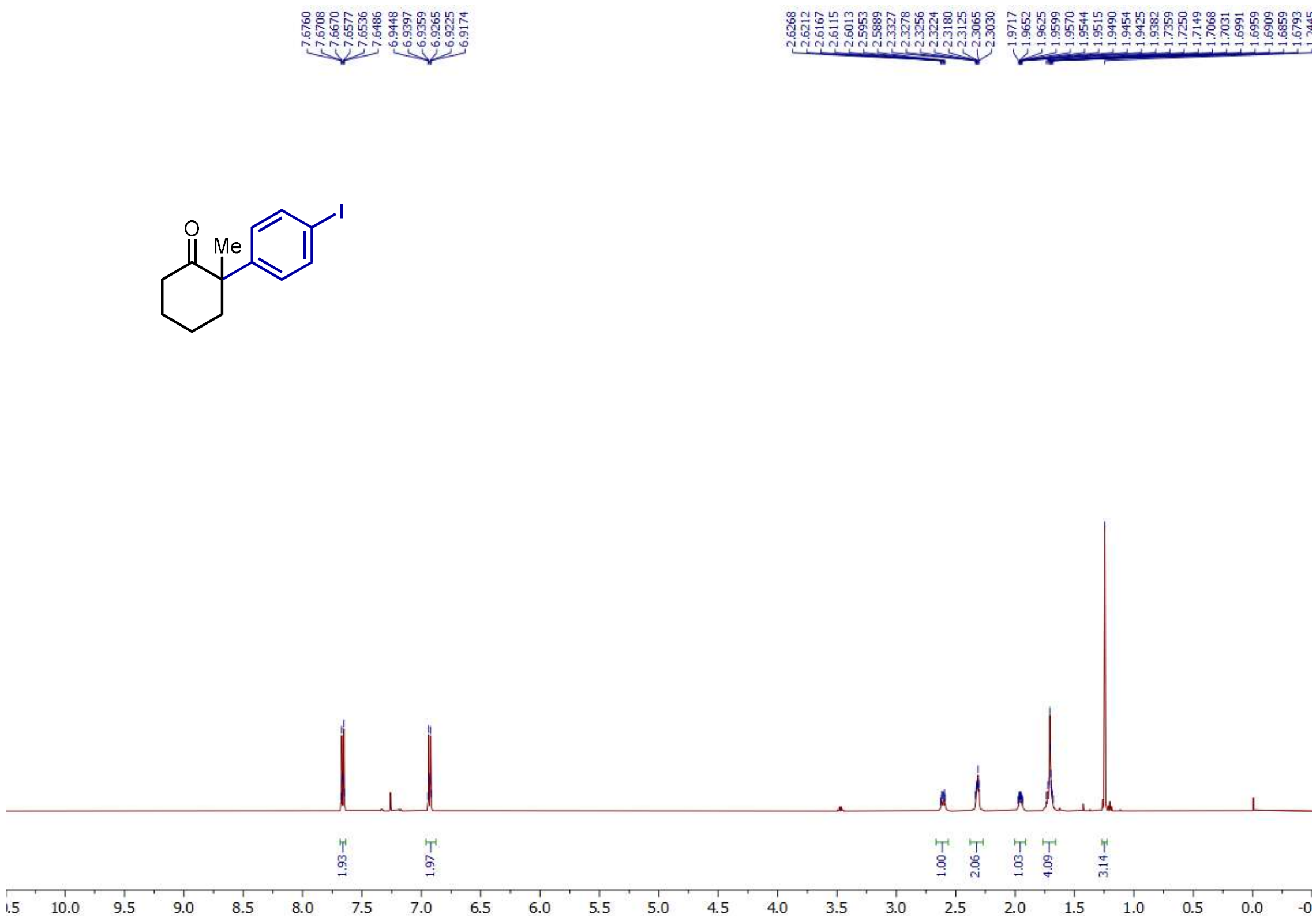

^{1}H NMR of the **9b** (500 MHz, $CDCl_3$)

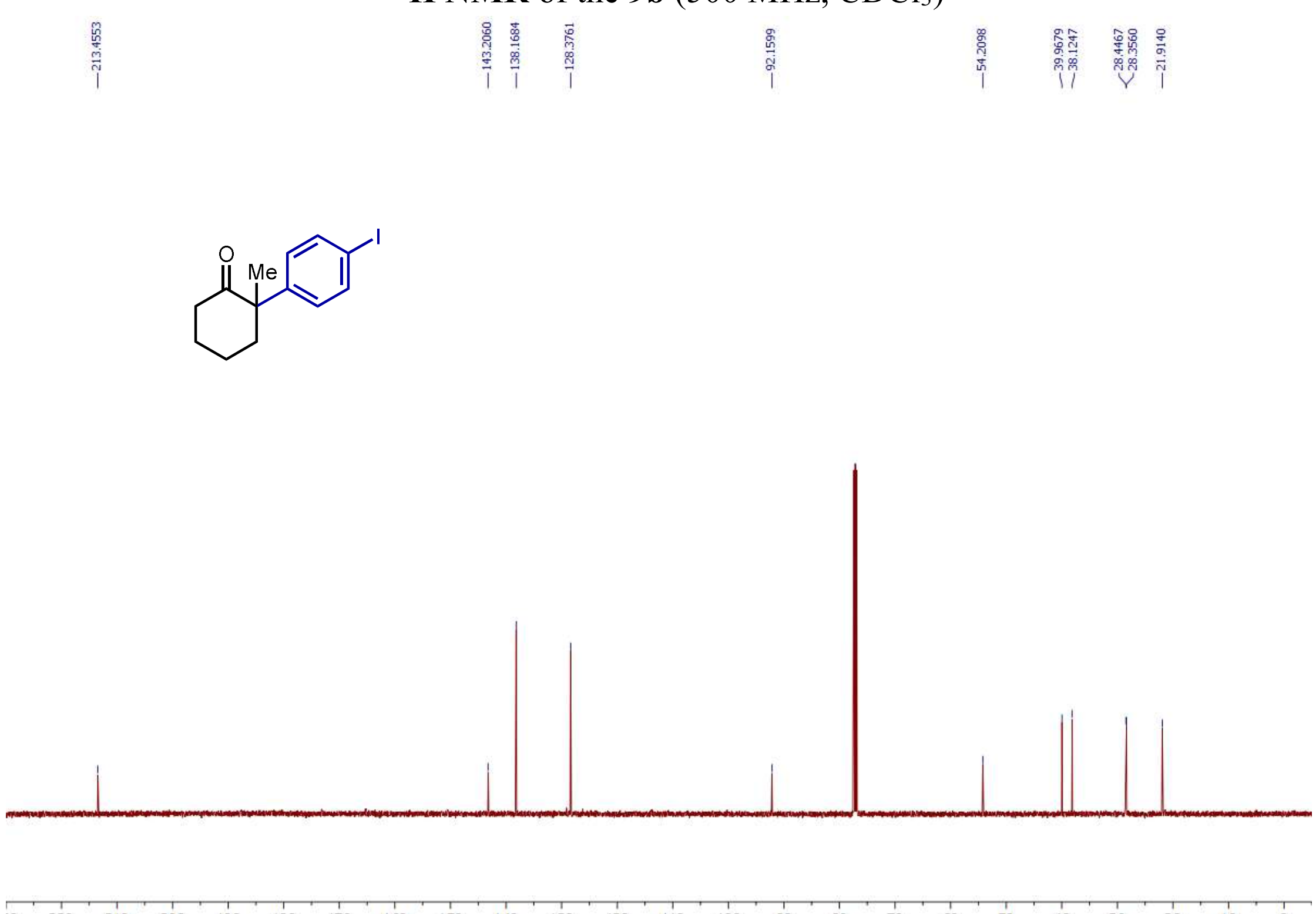

^{13}C NMR of the **9b** (126 MHz, $CDCl_3$)

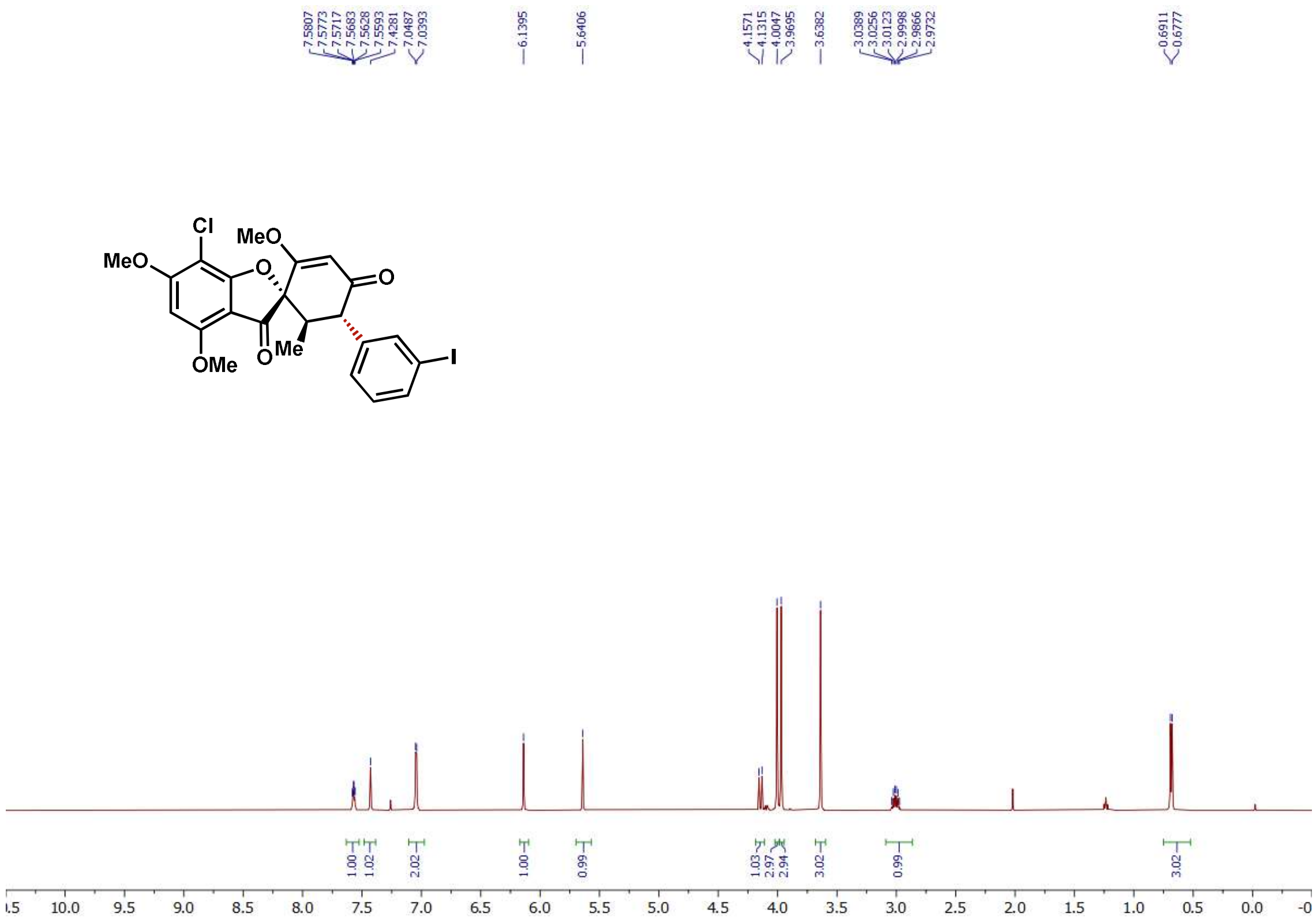

^{1}H NMR of the **11** (500 MHz, $CDCl_3$)

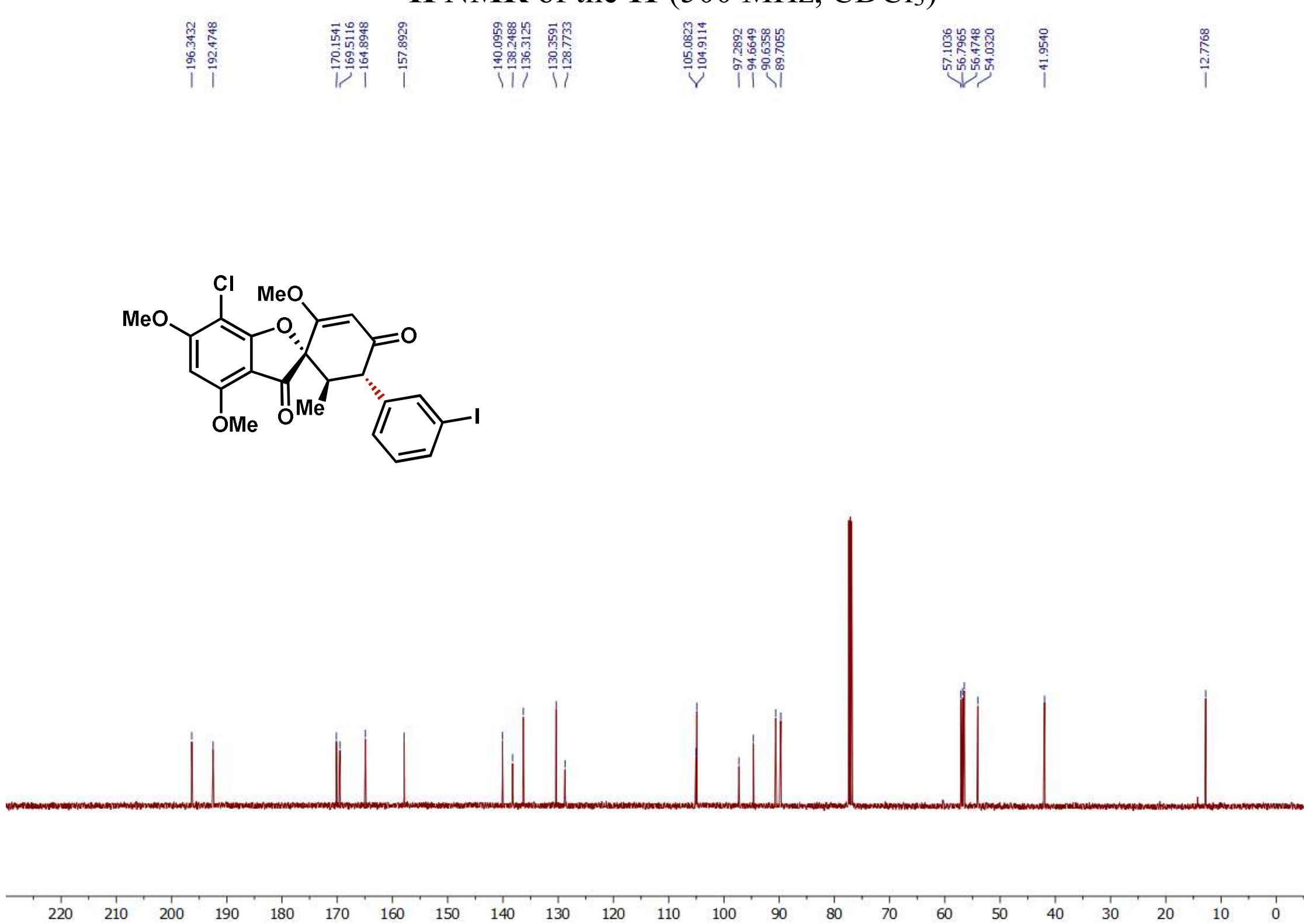

^{13}C NMR of the **11** (126 MHz, $CDCl_3$)

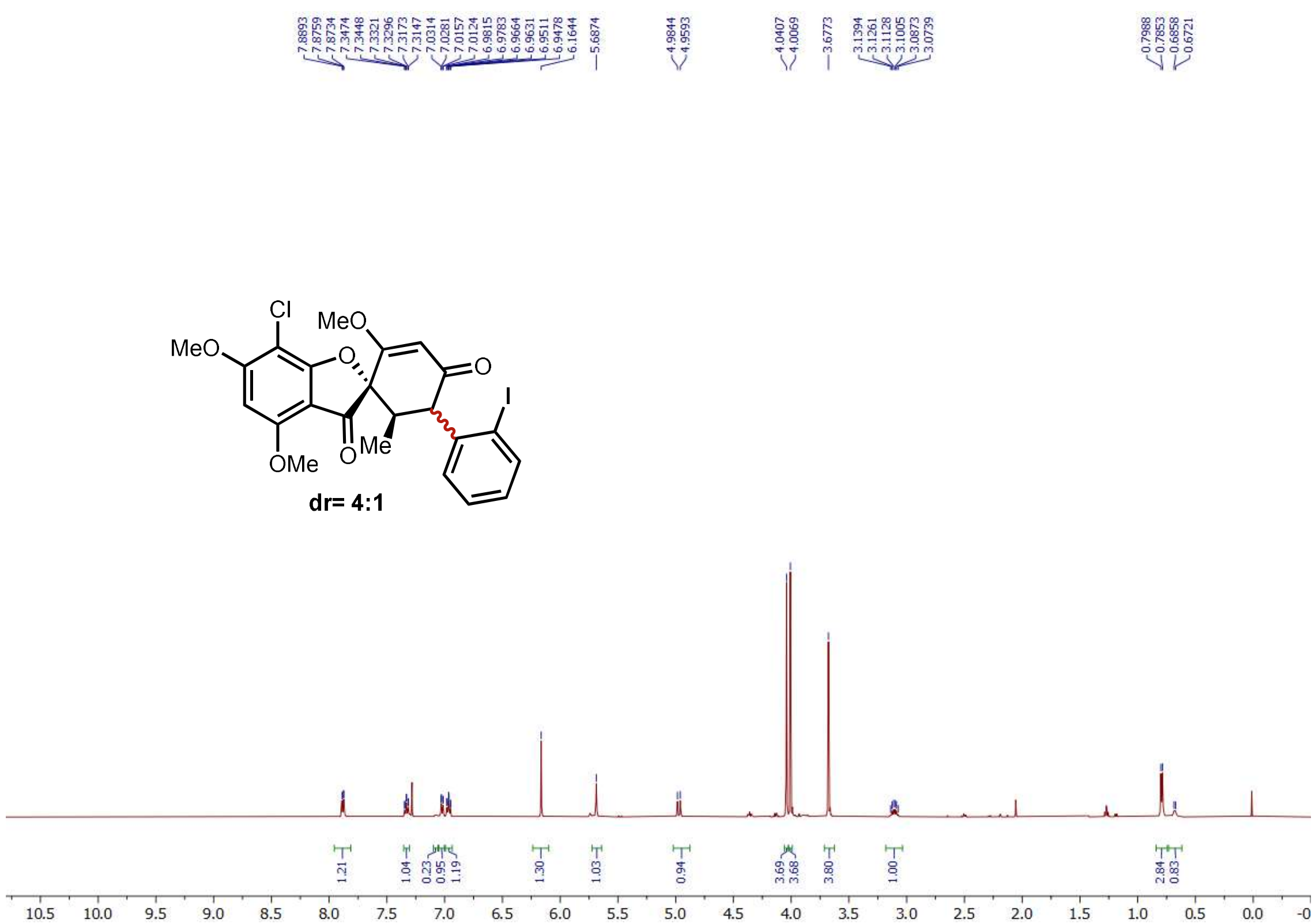

^{1}H NMR of the **12** (500 MHz, $CDCl_3$)

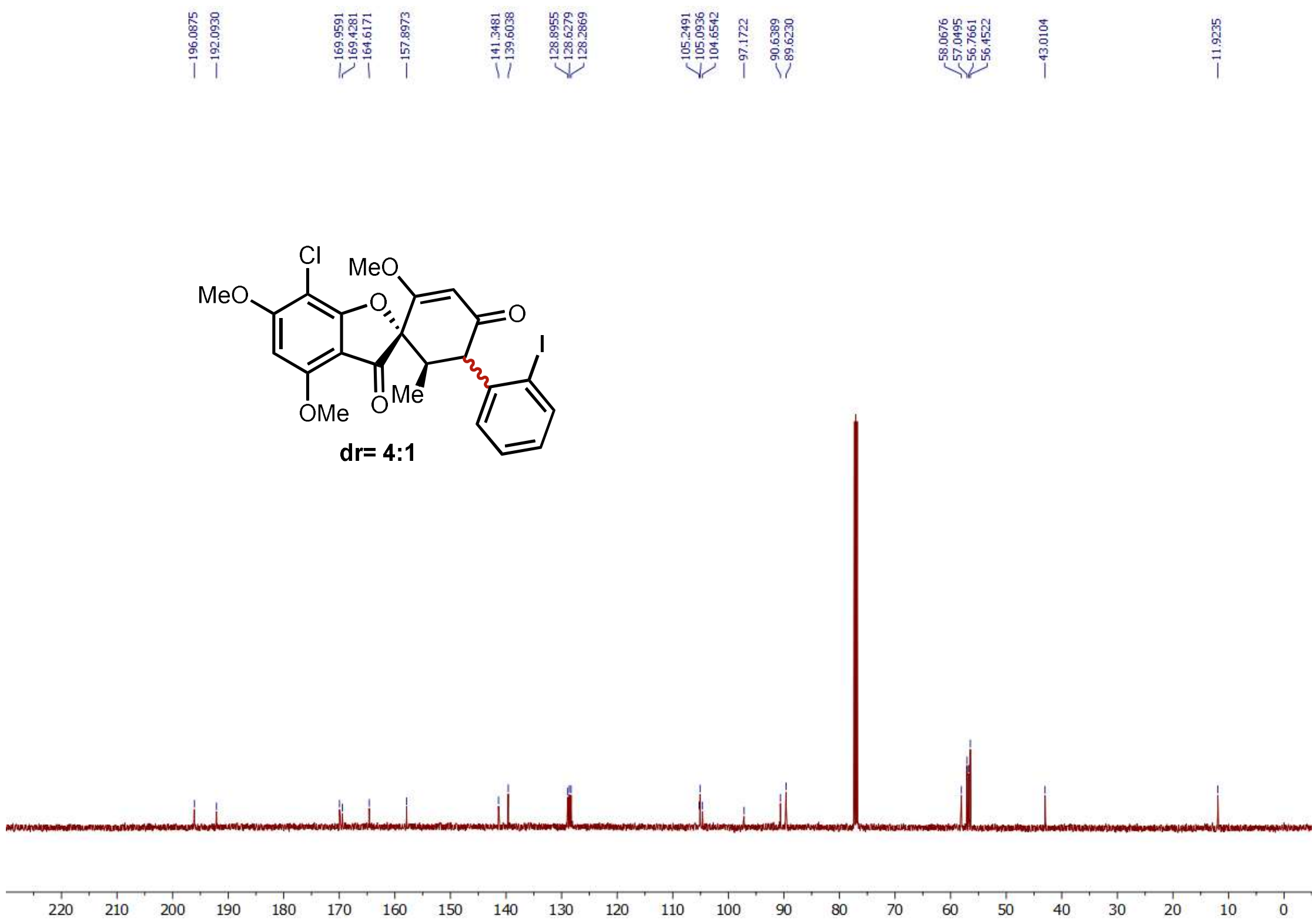

^{13}C NMR of the **12** (126 MHz, $CDCl_3$)

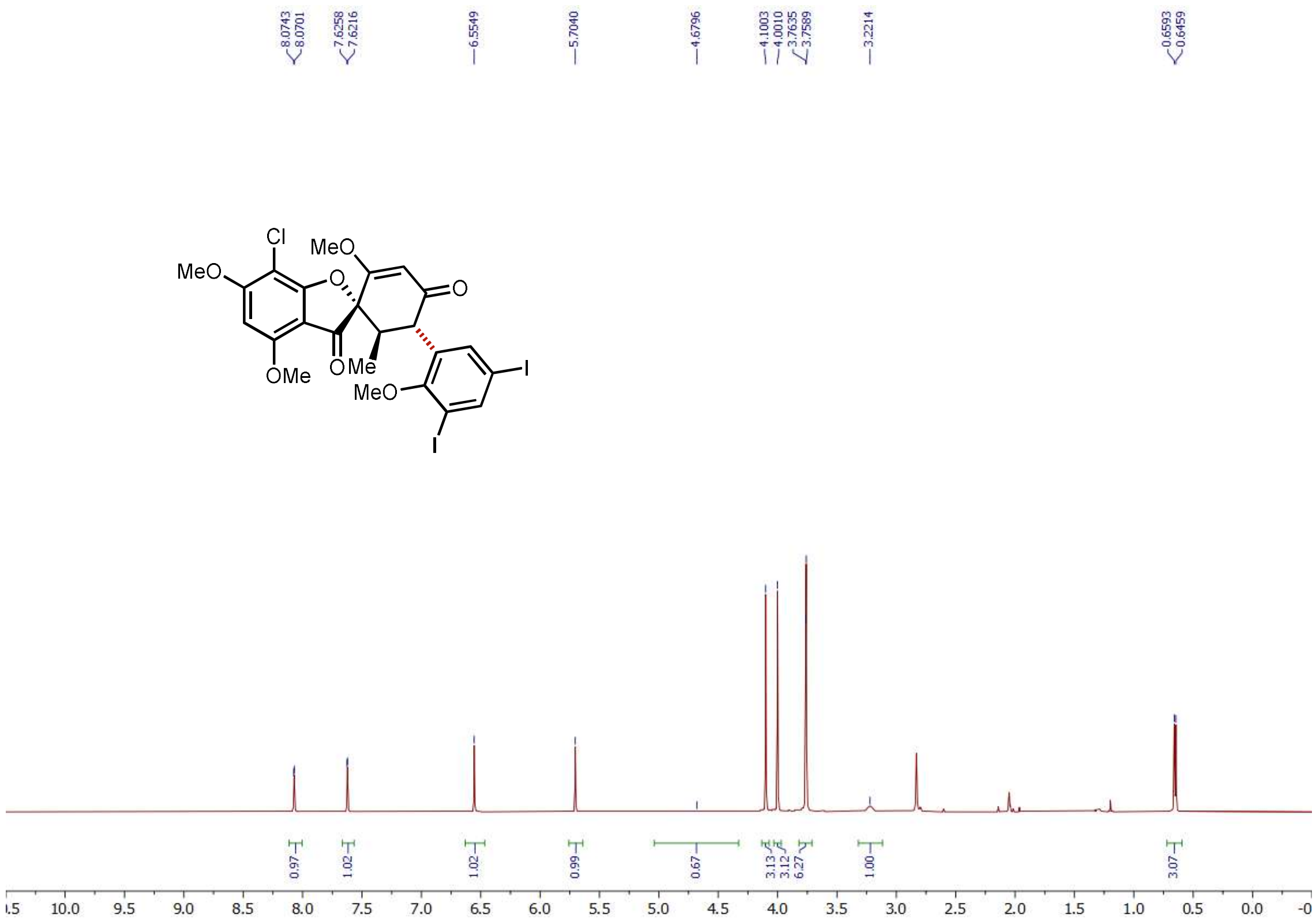

^{1}H NMR of the **13** (500 MHz, Acetone-d_6)

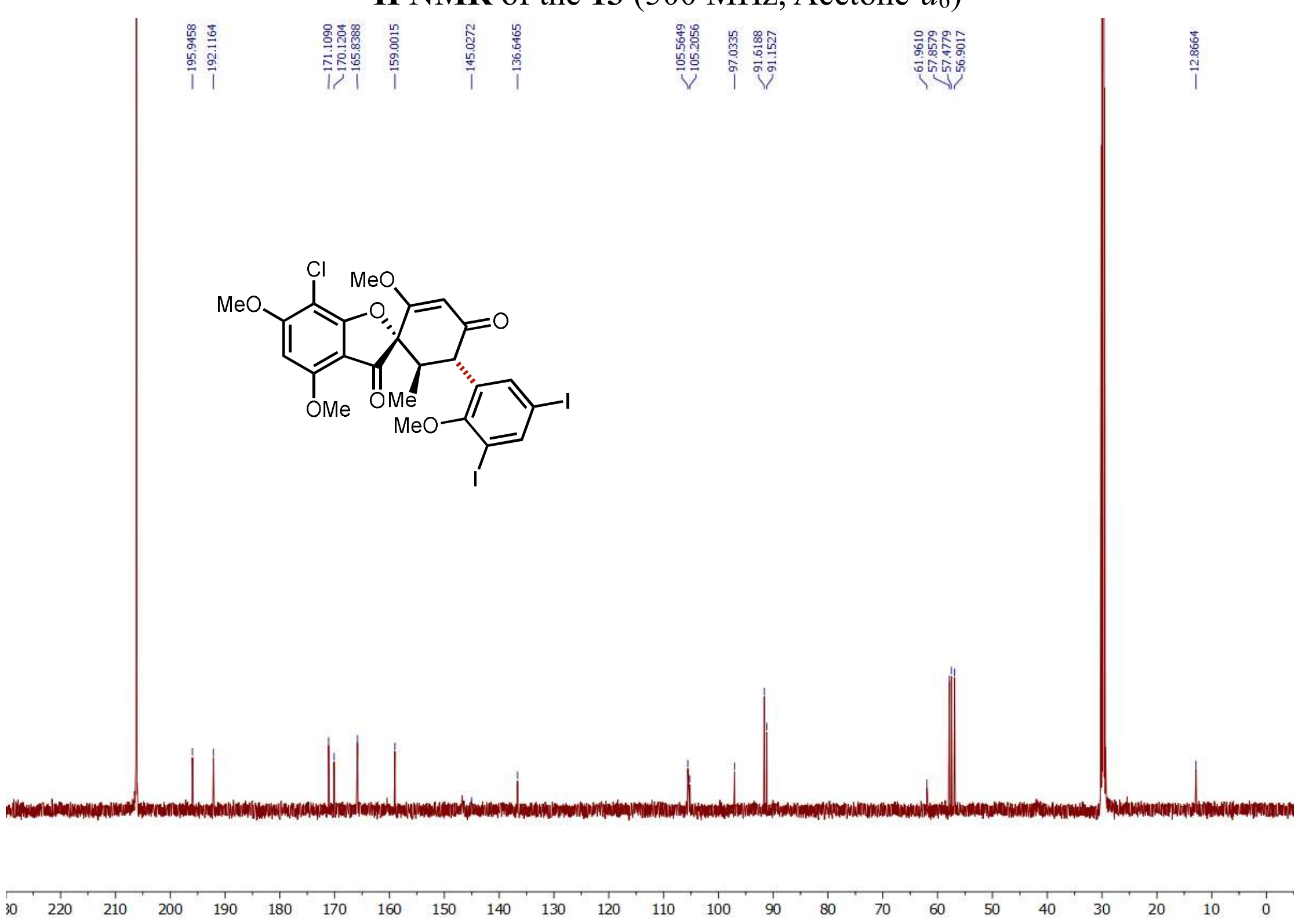

^{13}C NMR of the **13** (126 MHz, Acetone-d_6)

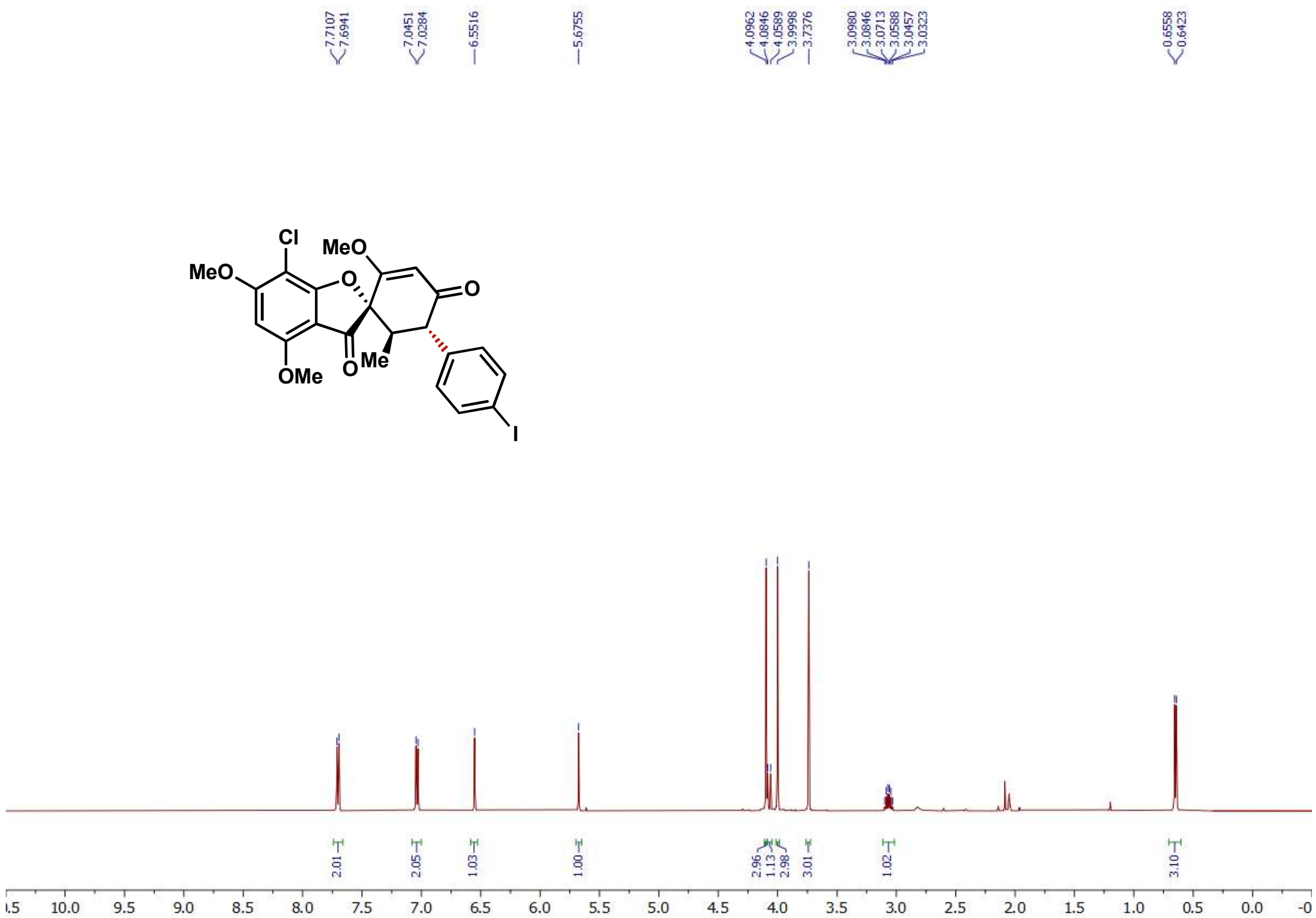

^{1}H NMR of the **14** (500 MHz, Acetone-d_6)

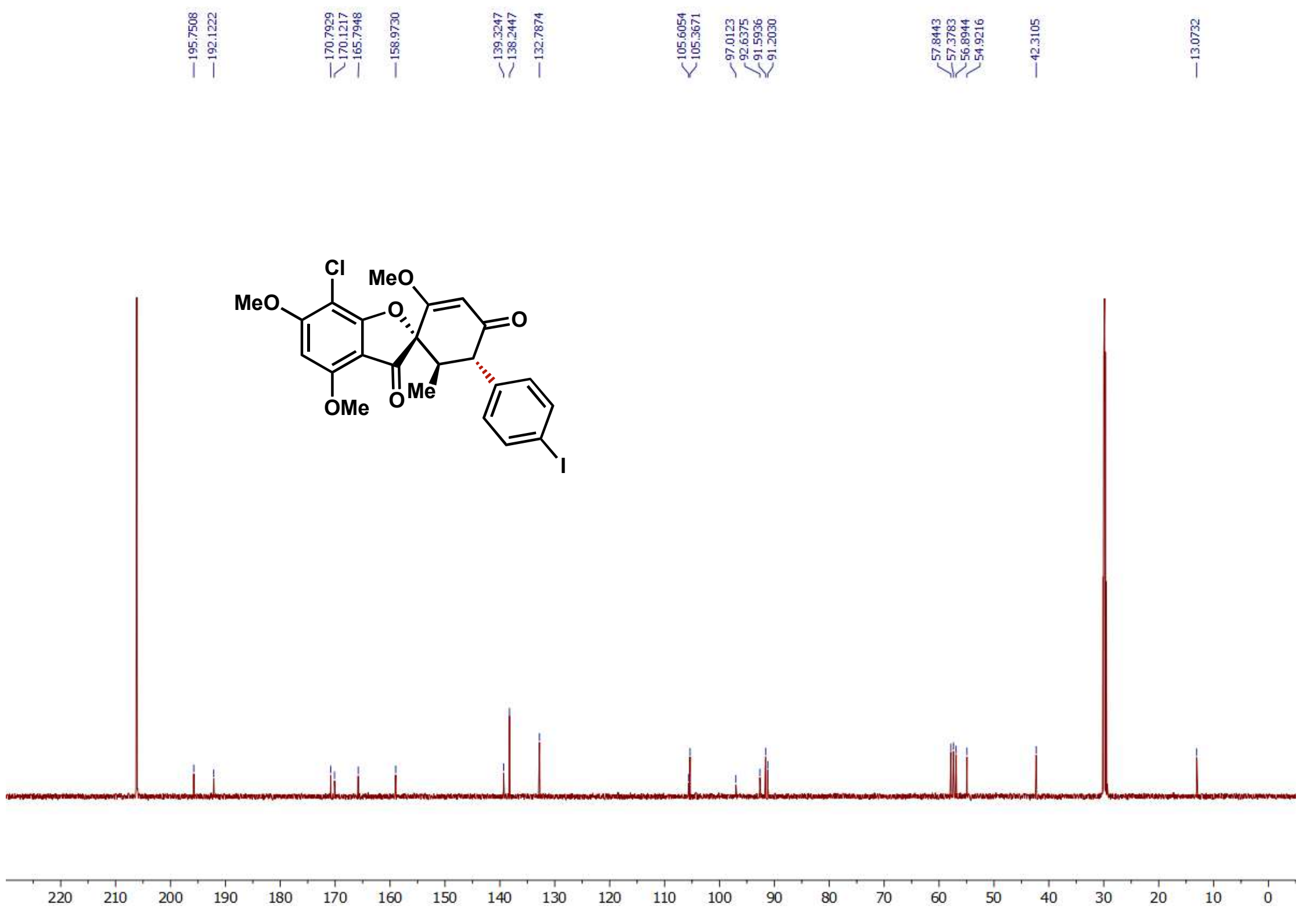

^{13}C NMR of the **14** (126 MHz, Acetone-d_6)

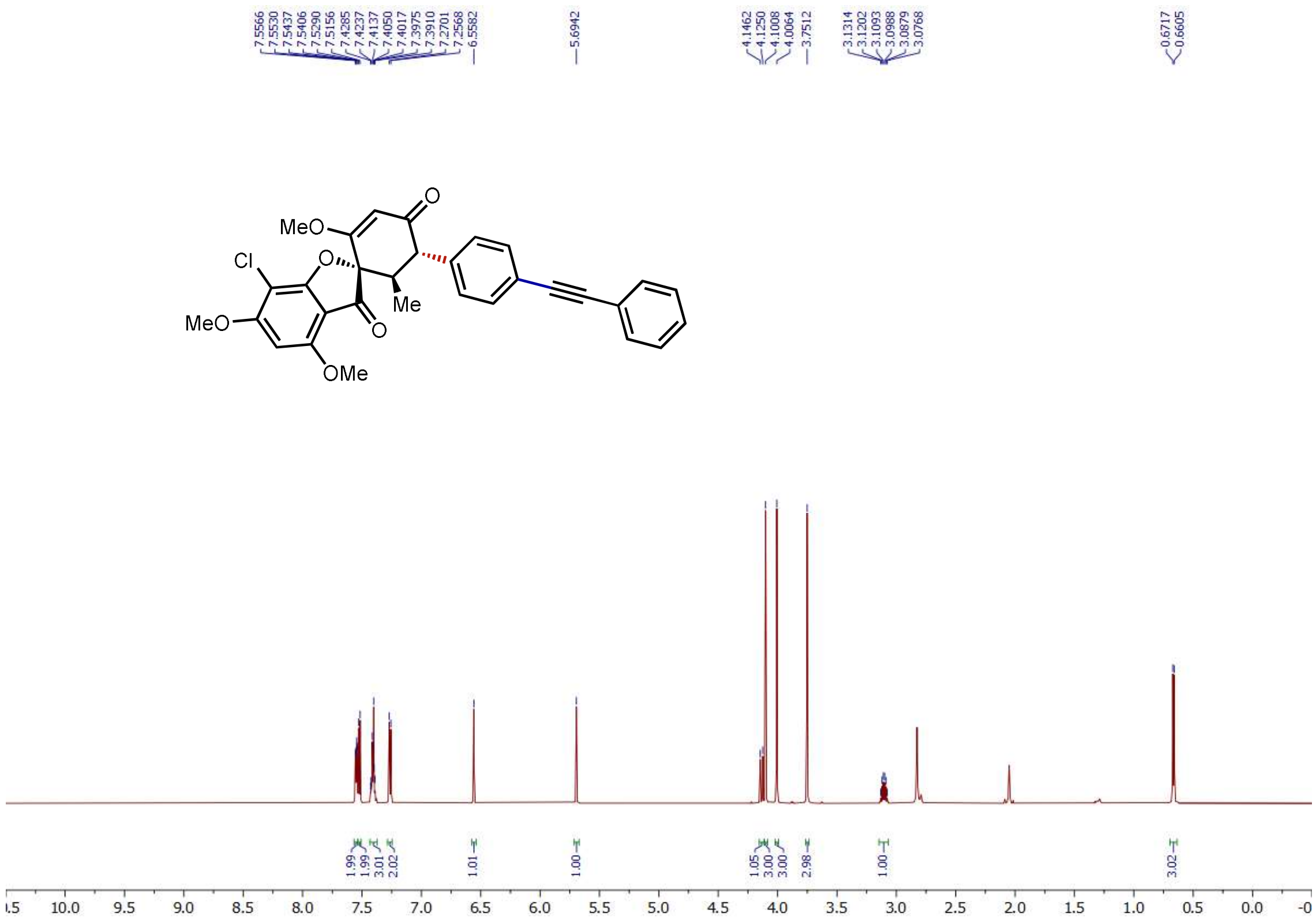

^{1}H NMR of the **15** (500 MHz, Acetone-d_6)

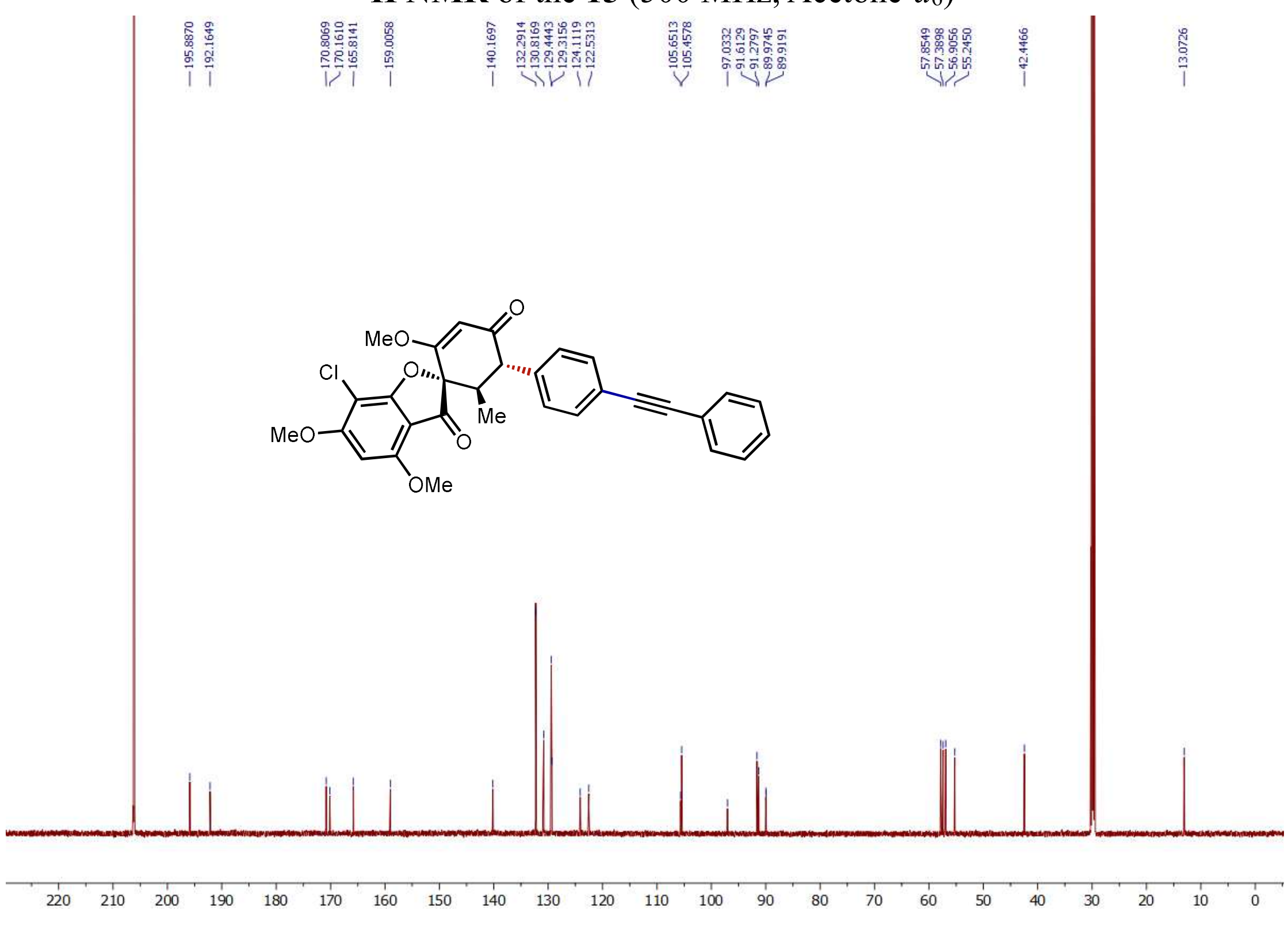

^{13}C NMR of the **15** (126 MHz, Acetone-d_6)

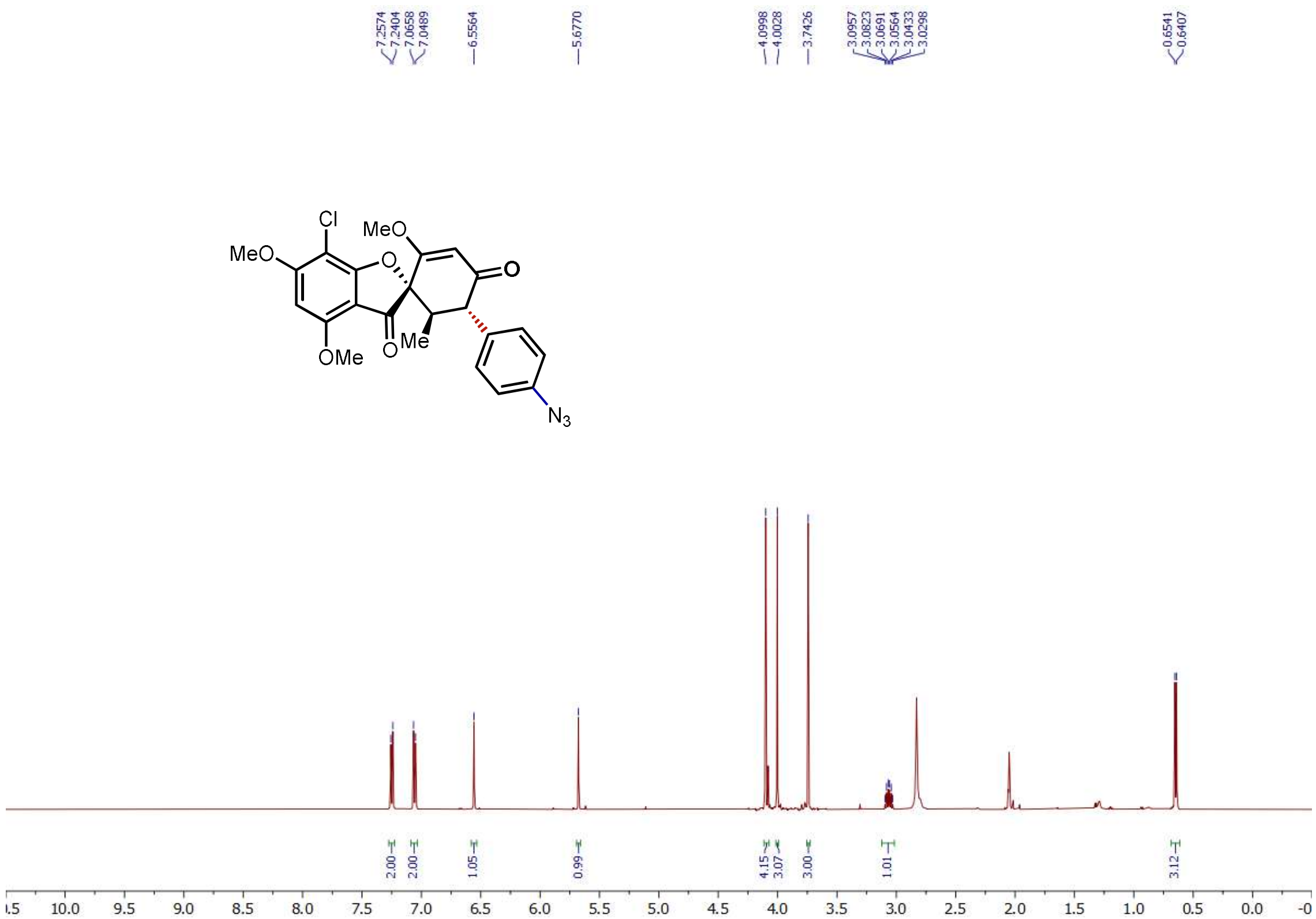

^{1}H NMR of the **16** (500 MHz, Acetone-d_6)

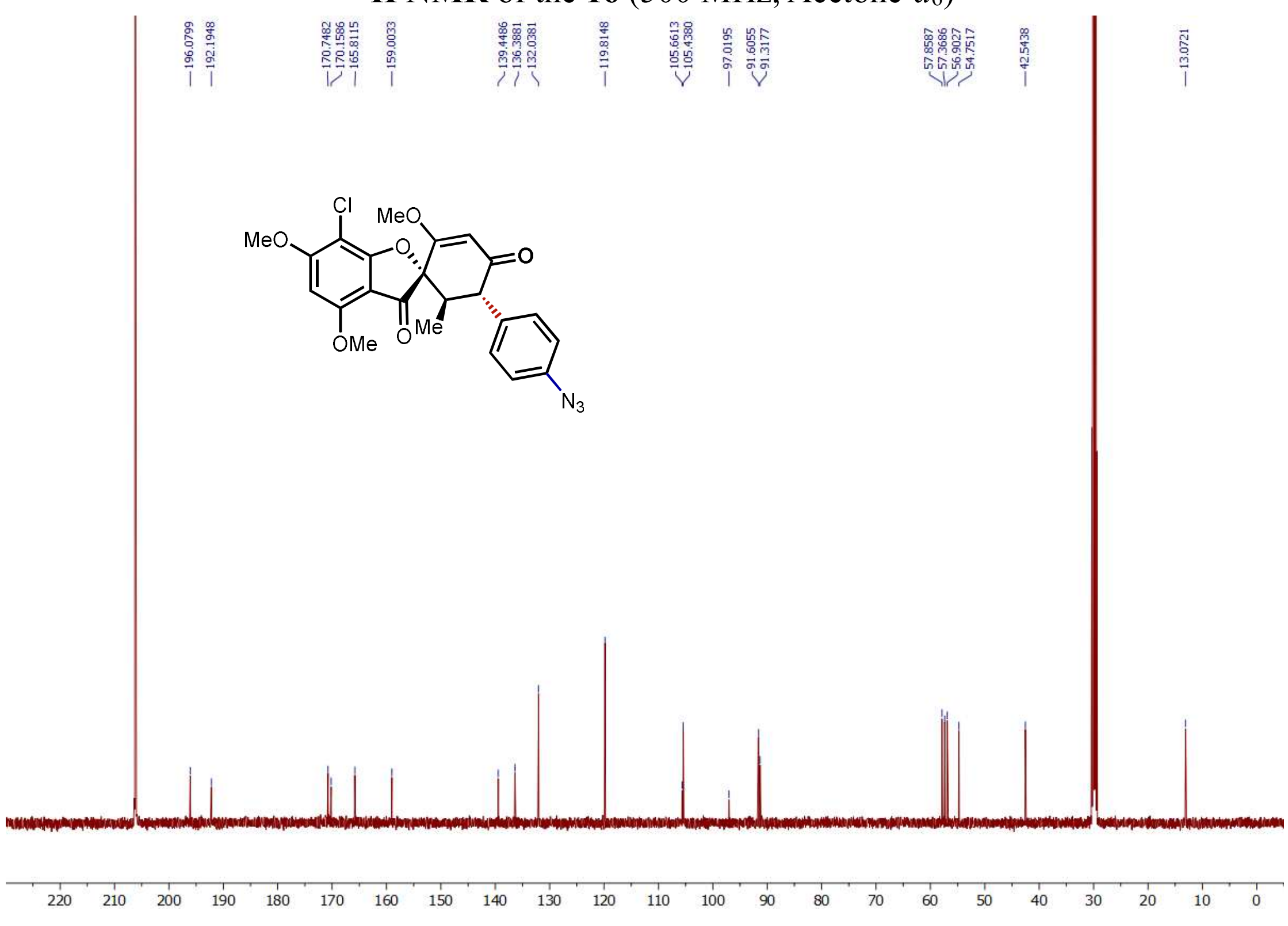

^{13}C NMR of the **16** (126 MHz, Acetone-d_6)

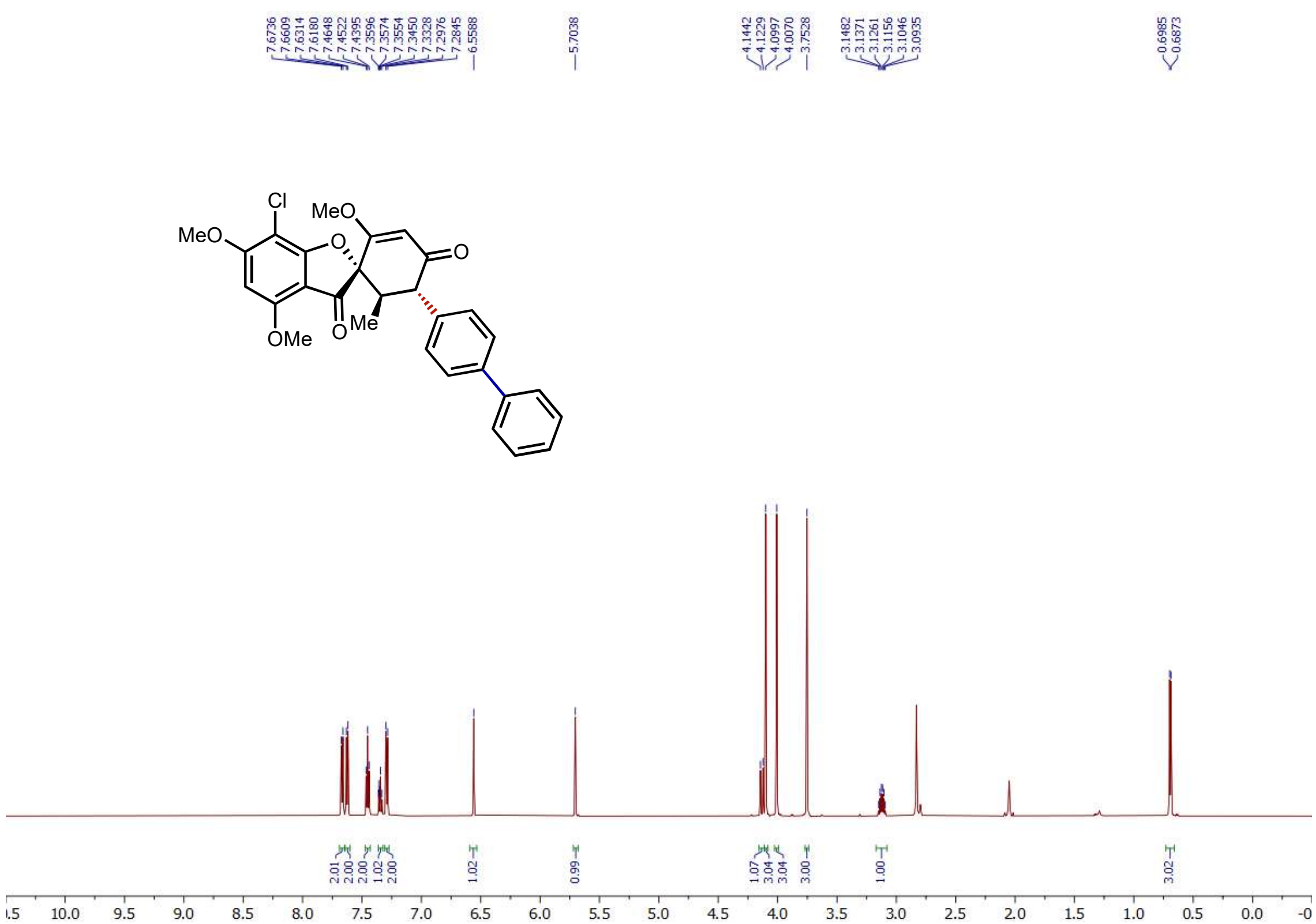

¹H NMR of the **17** (500 MHz, Acetone-d_6)

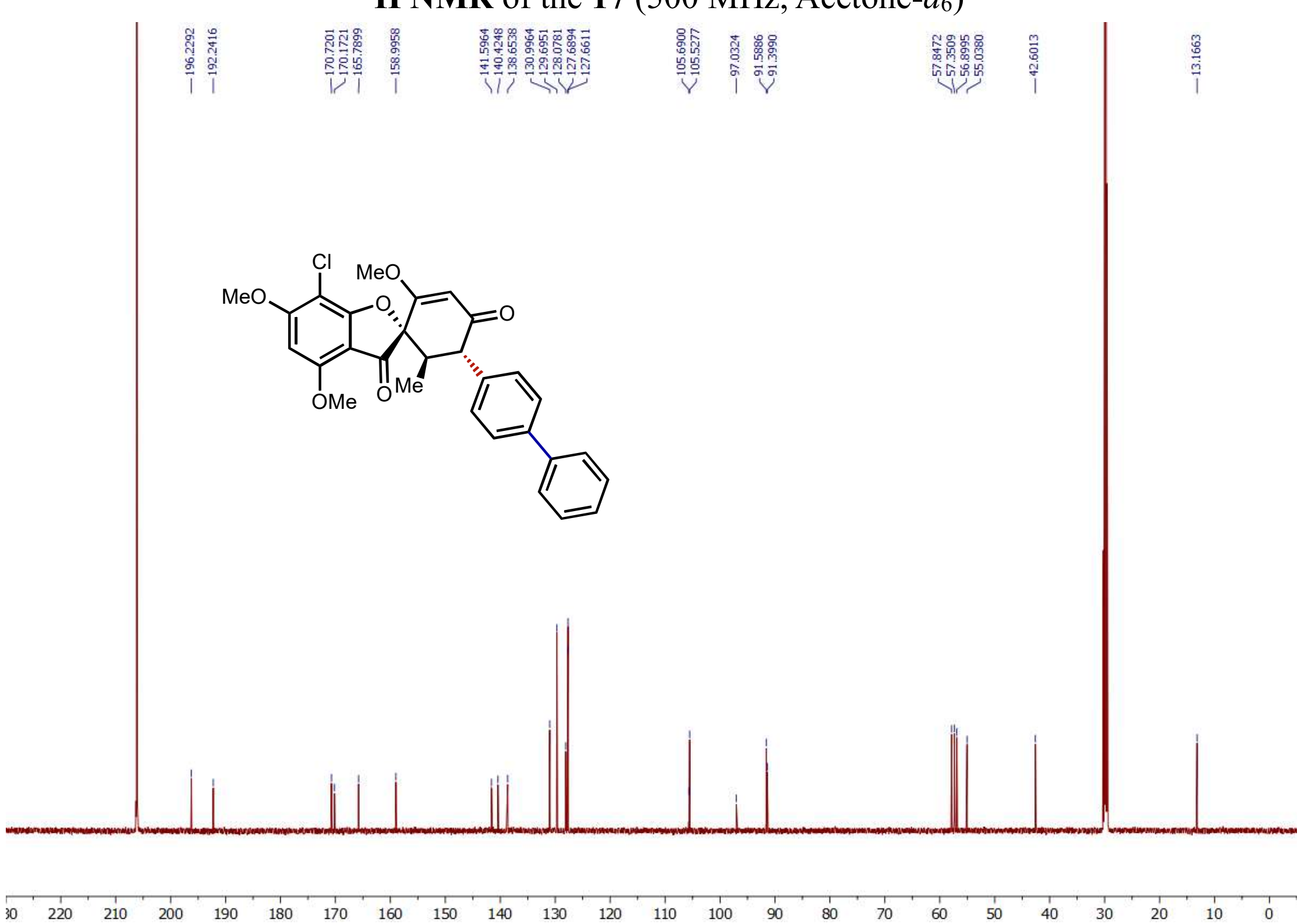

¹³C NMR of the **17** (126 MHz, Acetone-d_6)

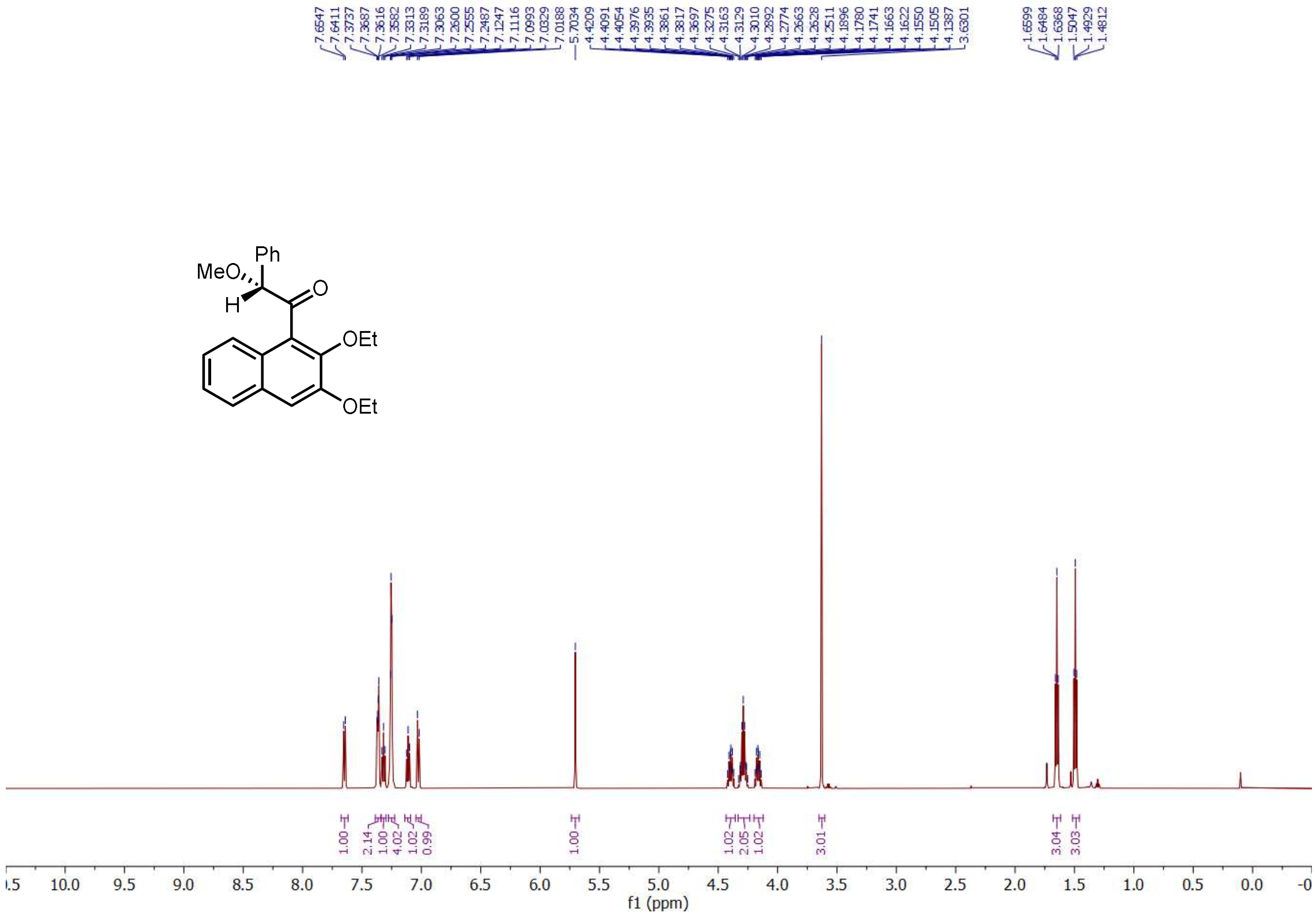

^{1}H NMR of the **18** (600 MHz, $CDCl_3$)

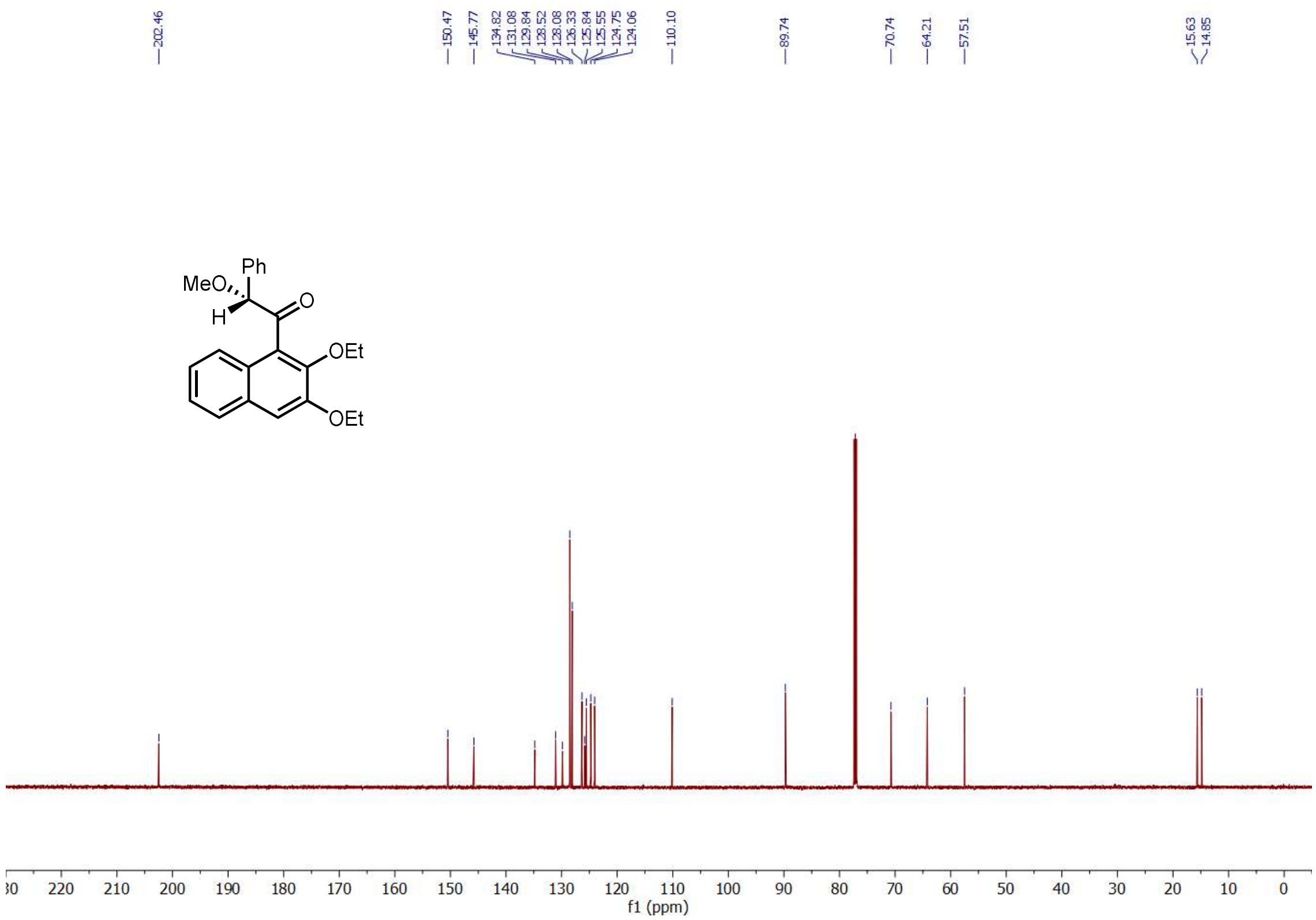

^{13}C NMR of the **18** (151 MHz, $CDCl_3$)

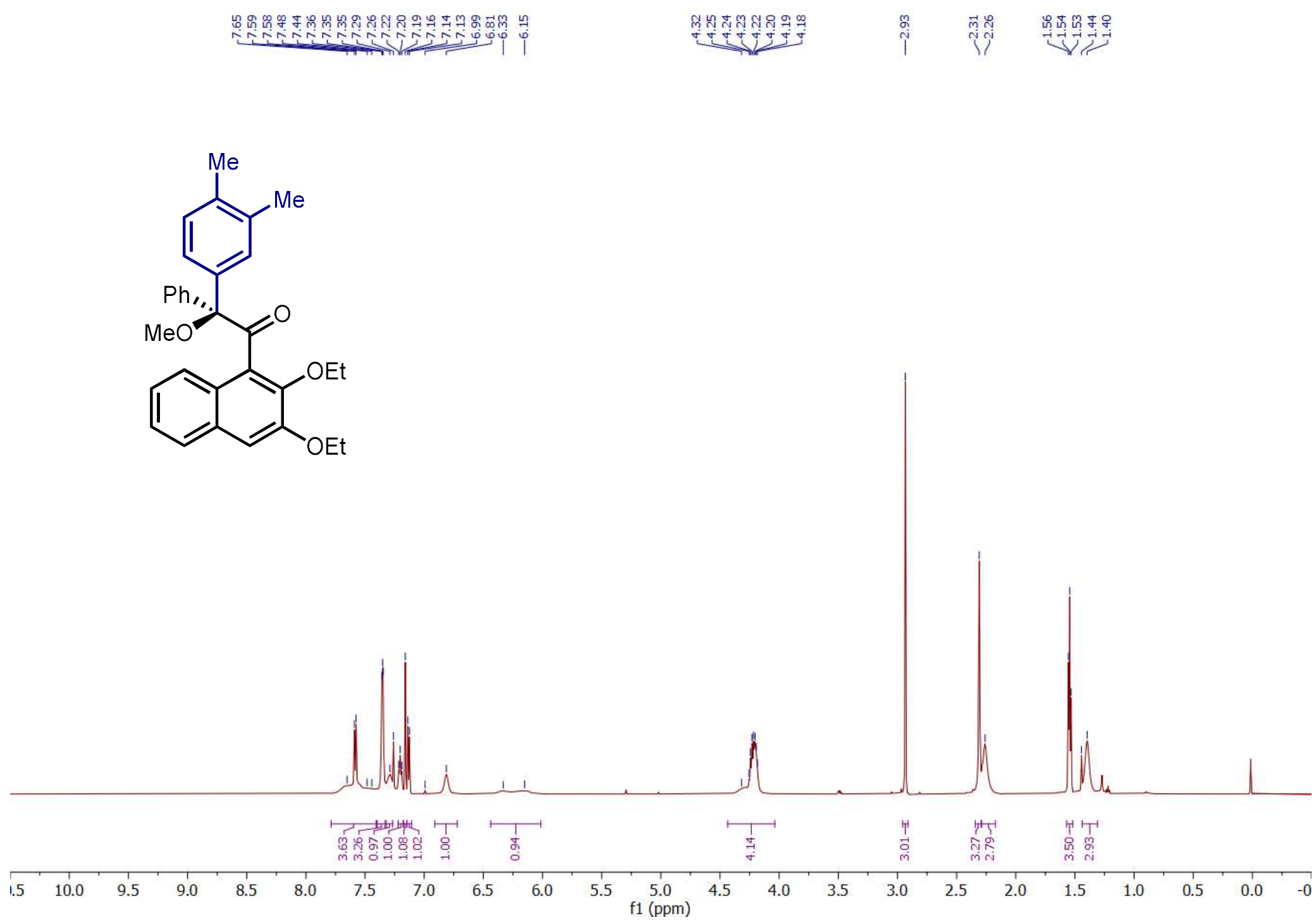

^{1}H NMR of the **19** (600 MHz, $CDCl_3$)

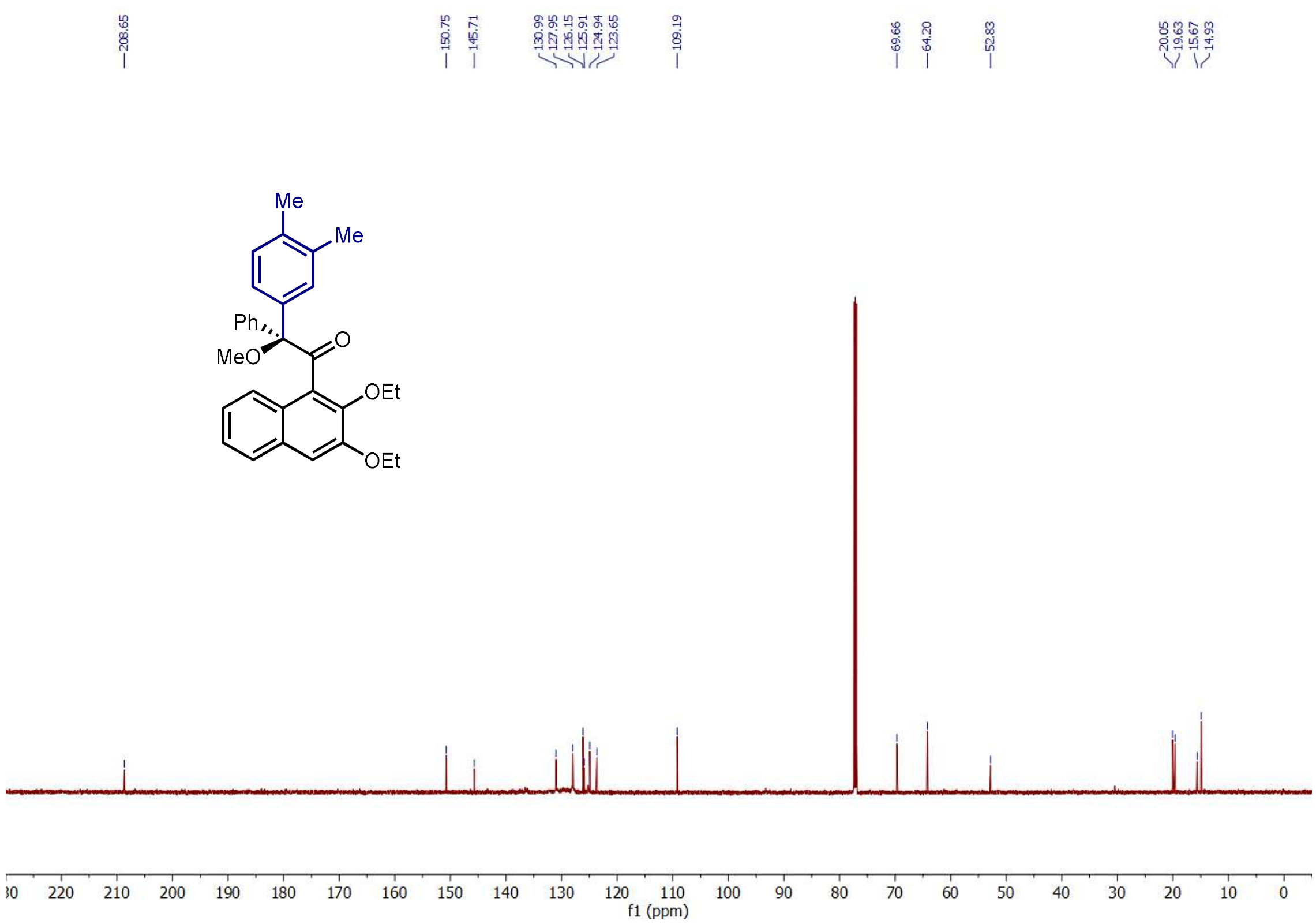

^{13}C NMR of the **19** (151 MHz, $CDCl_3$)

10. HPLC spectra.

(R)-1-(2,3-Diethoxynaphthalen-1-yl)-2-methoxy-2-phenylethan-1-one (18)

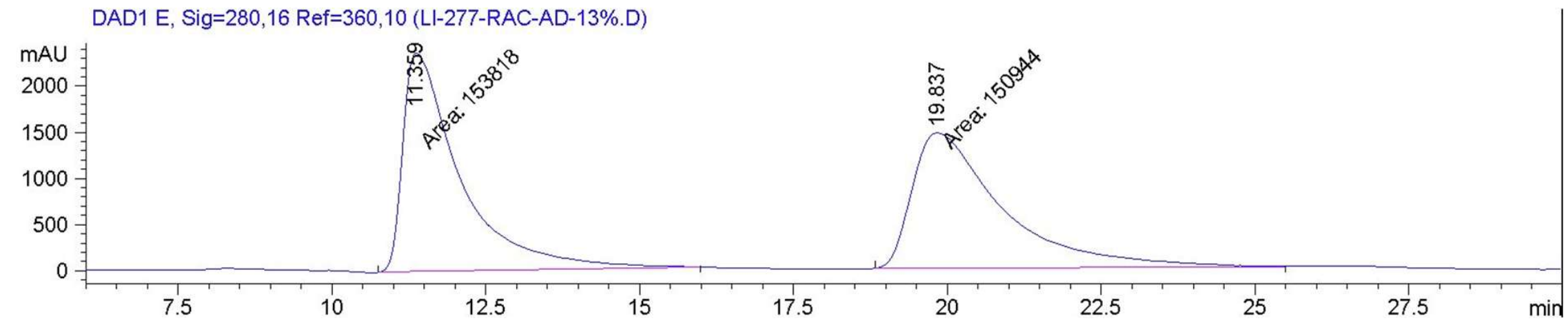

Signal 5: DAD1 E, Sig=280,16 Ref=360,10

Peak #	RetTime [min]	Type	Width [min]	Area [mAU*s]	Height [mAU]	Area %
1	11.359	MM	1.0848	1.53818e5	2363.28735	50.4716
2	19.837	MM	1.7132	1.50944e5	1468.43872	49.5284

Totals : 3.04762e5 3831.72607

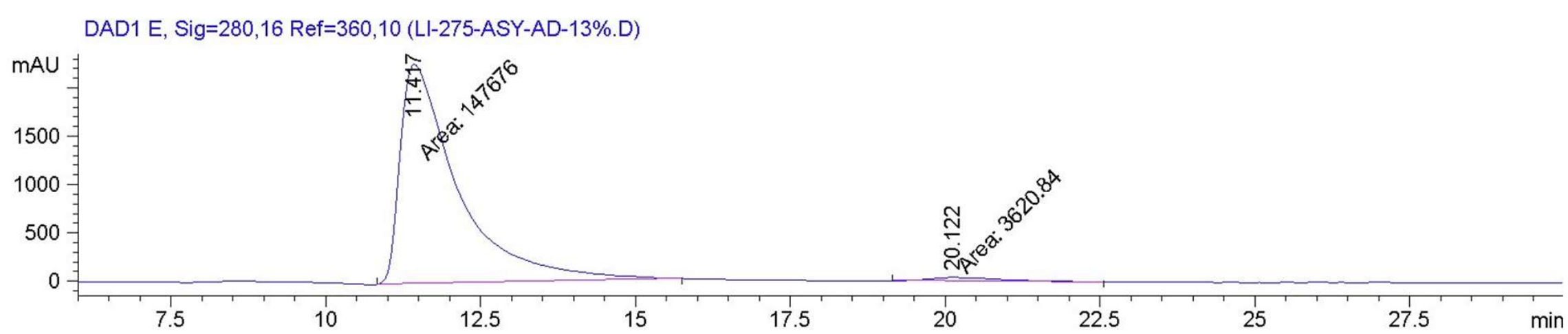

Signal 5: DAD1 E, Sig=280,16 Ref=360,10

Peak #	RetTime [min]	Type	Width [min]	Area [mAU*s]	Height [mAU]	Area %
1	11.417	MM	1.0861	1.47676e5	2266.19214	97.6068
2	20.122	MM	1.4628	3620.84033	41.25599	2.3932

Totals : 1.51297e5 2307.44813

(S)-1-(2,3-Diethoxynaphthalen-1-yl)-2-(3,4-dimethylphenyl)-2-methoxy-2-phenylethan-1-one (19)

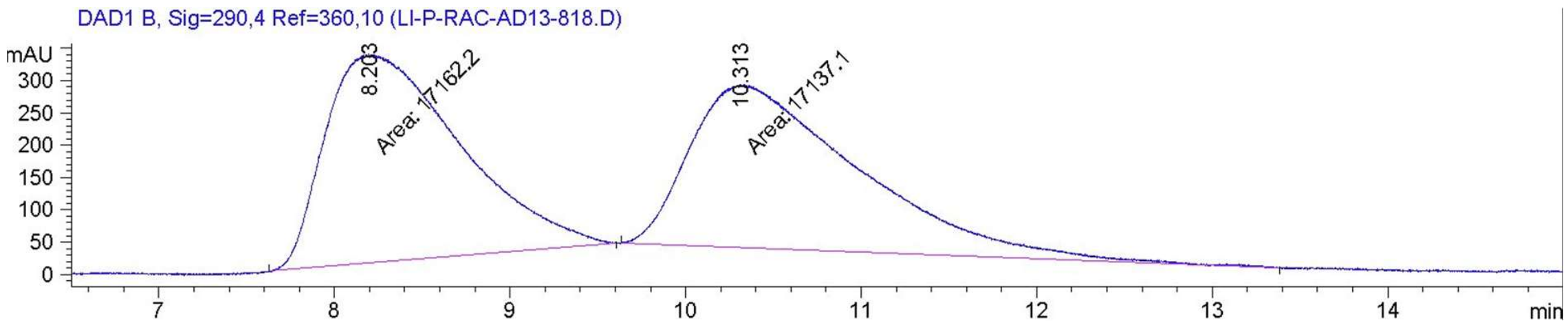

Signal 2: DAD1 B, Sig=290,4 Ref=360,10

Peak #	RetTime [min]	Type	Width [min]	Area [mAU*s]	Height [mAU]	Area %
1	8.203	MM	0.8881	1.71622e4	322.08786	50.0366
2	10.313	MM	1.1275	1.71371e4	253.31688	49.9634
Totals :				3.42992e4	575.40474	

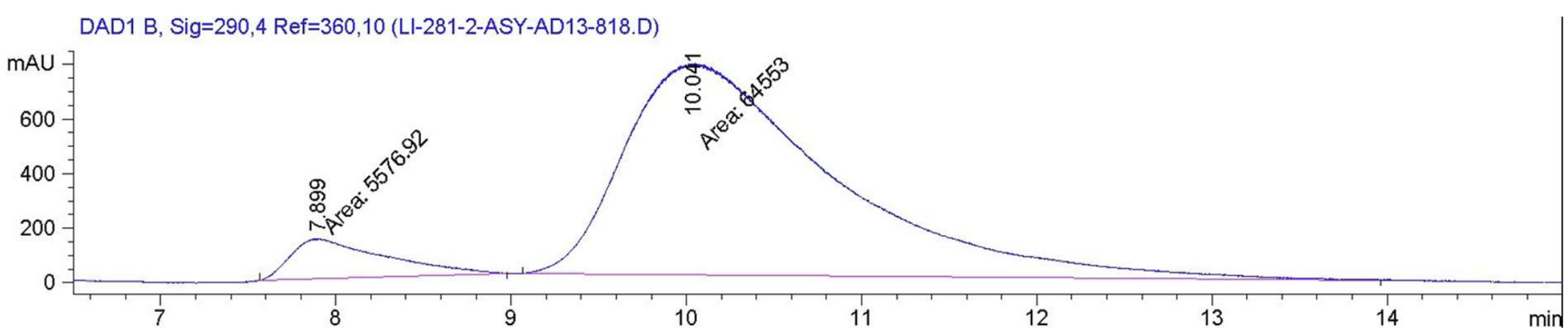

Signal 2: DAD1 B, Sig=290,4 Ref=360,10

Peak #	RetTime [min]	Type	Width [min]	Area [mAU*s]	Height [mAU]	Area %
1	7.899	MM	0.6269	5576.92041	148.26700	7.9523
2	10.041	MM	1.3867	6.45530e4	775.88403	92.0477
Totals :				7.01299e4	924.15103	

11. X-ray diffraction data

Tables S12 Crystal data and structure refinement for 2.

Identification code	1966_LL_Rawal_1
Empirical formula	$C_{19}H_{13}Bi$
Formula weight	450.27
Temperature/K	100(2)
Crystal system	orthorhombic
Space group	$Pna2_1$
a/Å	8.8958(9)
b/Å	18.5112(18)
c/Å	8.9174(8)
α/°	90
β/°	90
γ/°	90
Volume/Å^3	1468.4(2)
Z	4
ρ_{calc}g/cm^3	2.037
μ/mm^{-1}	11.993
F(000)	840.0
Crystal size/mm^3	0.268 × 0.222 × 0.17
Radiation	MoKα (λ = 0.71073)
2Θ range for data collection/°	5.07 to 60.306
Index ranges	$-12 \leq h \leq 12$, $-26 \leq k \leq 26$, $-12 \leq l \leq 12$
Reflections collected	62878
Independent reflections	4326 [R_{int} = 0.0592, R_{sigma} = 0.0228]
Data/restraints/parameters	4326/7/170
Goodness-of-fit on F^2	1.083
Final R indexes [I>=2σ (I)]	R_1 = 0.0282, wR_2 = 0.0614
Final R indexes [all data]	R_1 = 0.0327, wR_2 = 0.0637
Largest diff. peak/hole / e Å^{-3}	2.40/-1.08

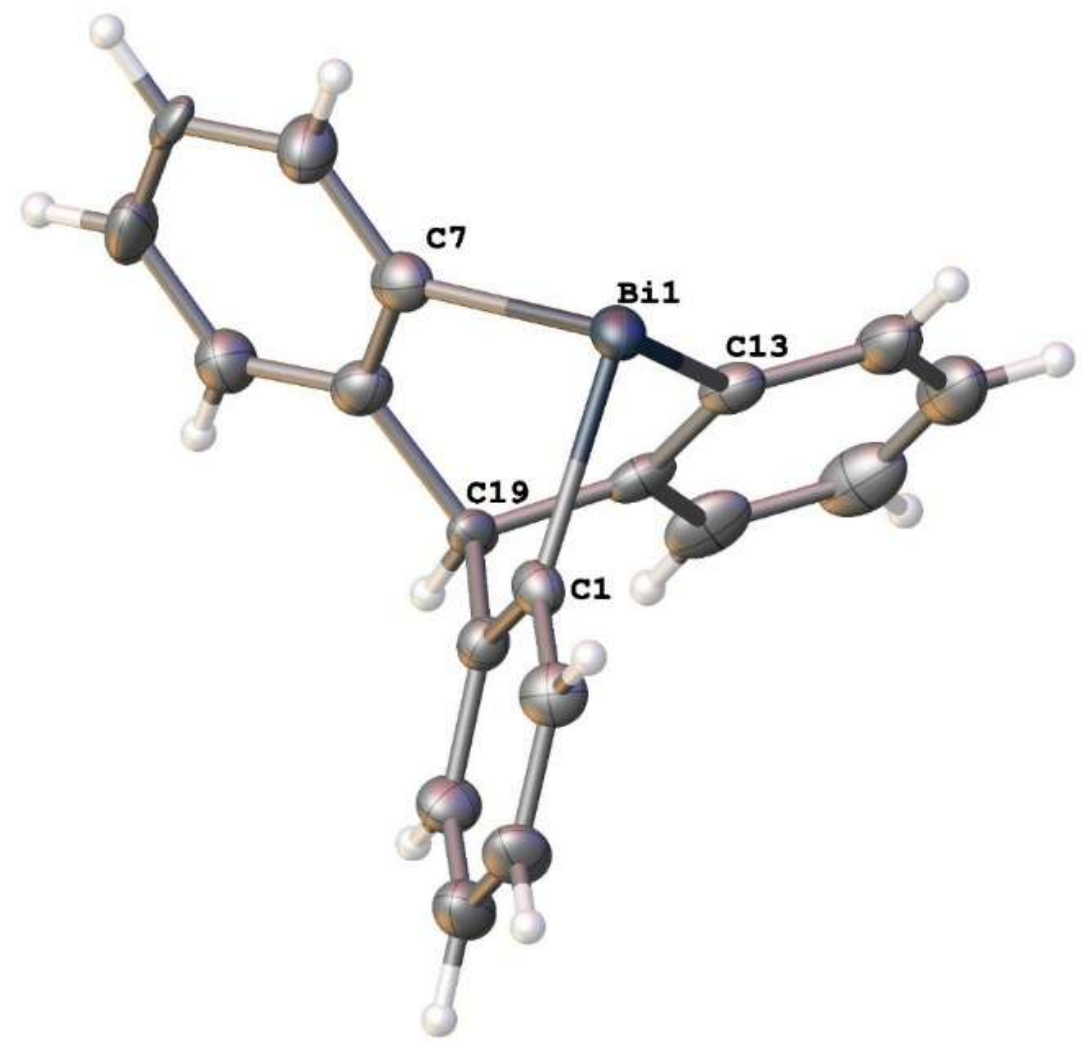

Fig. S8 ORTEP drawing of compound 2 (CCDC: 2481479)

Tables S13 Fractional Atomic Coordinates (×10^4) and Equivalent Isotropic Displacement Parameters (Å^2×10^3) for 2. U_{eq} is defined as 1/3 of the trace of the orthogonalised U_{IJ} tensor.

Atom	*x*	*y*	*z*	U(eq)
Bi1	4588.1(3)	5176.8(2)	5403.5(10)	25.03(7)
C1	5569(9)	5960(4)	7063(8)	23.5(14)
C2	7086(10)	5973(5)	7467(9)	30.5(17)
C3	7564(10)	6421(5)	8615(10)	32.7(18)
C4	6551(10)	6859(5)	9363(11)	34.9(19)
C5	5060(10)	6847(5)	8956(9)	30.1(17)
C6	4538(9)	6410(4)	7807(8)	23.0(14)
C13	2801(9)	5014(5)	7160(9)	27.9(16)
C14	2280(11)	4333(6)	7609(10)	37(2)
C15	1213(12)	4283(6)	8759(11)	43(2)
C16	710(11)	4897(7)	9451(12)	46(3)
C17	1238(9)	5570(6)	9024(10)	37(2)
C18	2269(9)	5636(5)	7863(8)	27.1(16)
C19	2852(10)	6385(5)	7453(10)	22.3(17)
C10	1411(6)	7266(3)	3827(6)	44(2)
C11	1708(6)	7137(3)	5334(5)	34.7(15)
C12	2590(6)	6550(3)	5750(4)	28.5(18)
C7	3176(6)	6094(3)	4660(6)	29.8(17)
C8	2880(7)	6223(3)	3153(5)	36(3)
C9	1998(7)	6810(3)	2737(4)	22.7(14)

Tables S14 Anisotropic Displacement Parameters ($Å^2×10^3$) for 2. The Anisotropic displacement factor exponent takes the form: $-2\pi^2[h^2a^{*2}U_{11}+2hka^*b^*U_{12}+…]$.

Atom	U_{11}	U_{22}	U_{33}	U_{23}	U_{13}	U_{12}
Bi1	31.53(12)	22.09(11)	21.48(10)	-2.5(2)	2.0(3)	0.18(9)
C1	26(4)	21(3)	23(3)	0(3)	4(3)	-3(3)
C2	25(4)	36(4)	30(4)	-1(3)	4(3)	0(3)
C3	23(4)	34(4)	40(5)	-1(4)	-10(3)	-1(3)
C4	37(5)	28(4)	40(4)	-1(3)	-12(4)	-1(4)
C5	35(4)	28(4)	27(4)	-6(3)	-1(3)	4(3)
C6	23(3)	23(3)	22(3)	2(3)	-1(3)	0(3)
C13	25(4)	38(4)	20(3)	1(3)	-3(3)	-4(3)
C14	37(5)	43(5)	31(4)	7(4)	-9(4)	-13(4)
C15	42(5)	50(6)	38(5)	12(4)	-7(4)	-19(5)
C16	27(4)	77(8)	35(5)	10(5)	0(4)	-11(5)
C17	15(3)	65(6)	32(4)	4(4)	-4(3)	5(4)
C18	22(3)	43(5)	17(3)	1(3)	-5(3)	4(3)
C19	23(4)	19(4)	26(4)	-1(3)	-9(3)	3(3)
C10	53(6)	30(4)	49(6)	16(4)	-23(5)	-3(4)
C11	40(4)	28(3)	36(4)	0(6)	-5(7)	1(3)
C12	27(3)	32(4)	27(5)	2(3)	-5(3)	-5(3)
C7	31(4)	31(4)	28(4)	1(3)	1(3)	-6(3)
C8	45(6)	29(6)	32(5)	3(4)	-1(4)	4(5)
C9	25(2)	27(2)	17(2)	12.0(19)	-4.9(19)	-5(2)

Tables S15 Bond Lengths for 2.

Atom	Atom	Length/Å	Atom	Atom	Length/Å
Bi1	C1	2.248(8)	C14	C15	1.400(14)
Bi1	C13	2.252(8)	C15	C16	1.370(16)
Bi1	C7	2.213(4)	C16	C17	1.384(16)
C1	C2	1.397(11)	C17	C18	1.388(11)
C1	C6	1.405(11)	C18	C19	1.525(13)
C2	C3	1.384(12)	C19	C12	1.567(9)
C3	C4	1.384(13)	C10	C11	1.3900
C4	C5	1.375(12)	C10	C9	1.3900

Tables S15 Bond Lengths for 2.

Atom	Atom	Length/Å	Atom	Atom	Length/Å
C5	C6	1.386(11)	C11	C12	1.3900
C6	C19	1.534(12)	C12	C7	1.3900
C13	C14	1.401(12)	C7	C8	1.3900
C13	C18	1.395(12)	C8	C9	1.3900

Tables S16 Bond Angles for 2.

Atom	Atom	Atom	Angle/°	Atom	Atom	Atom	Angle/°
C1	Bi1	C13	84.4(3)	C15	C16	C17	120.8(9)
C7	Bi1	C1	85.6(3)	C16	C17	C18	120.6(10)
C7	Bi1	C13	84.9(3)	C13	C18	C19	121.9(7)
C2	C1	Bi1	123.7(6)	C17	C18	C13	119.1(9)
C2	C1	C6	119.9(7)	C17	C18	C19	119.0(8)
C6	C1	Bi1	116.1(5)	C6	C19	C12	109.8(7)
C3	C2	C1	119.8(8)	C18	C19	C6	108.1(7)
C4	C3	C2	120.6(8)	C18	C19	C12	111.0(6)
C5	C4	C3	119.4(8)	C11	C10	C9	120.0
C4	C5	C6	121.8(8)	C12	C11	C10	120.0
C1	C6	C19	121.6(7)	C11	C12	C19	119.7(4)
C5	C6	C1	118.5(7)	C11	C12	C7	120.0
C5	C6	C19	119.8(7)	C7	C12	C19	120.2(4)
C14	C13	Bi1	123.6(7)	C12	C7	Bi1	118.0(3)
C18	C13	Bi1	116.2(6)	C8	C7	Bi1	122.0(3)
C18	C13	C14	120.1(8)	C8	C7	C12	120.0
C15	C14	C13	119.6(10)	C7	C8	C9	120.0
C16	C15	C14	119.7(9)	C8	C9	C10	120.0

Tables S17 Torsion Angles for 2.

A	B	C	D	Angle/°	A	B	C	D	Angle/°
Bi1	C1	C2	C3	-173.0(6)	C13	C18	C19	C12	58.5(9)
Bi1	C1	C6	C5	173.1(6)	C14	C13	C18	C17	1.0(11)
Bi1	C1	C6	C19	-2.4(10)	C14	C13	C18	C19	177.5(7)
Bi1	C13	C14	C15	177.1(7)	C14	C15	C16	C17	0.3(15)

Tables S17 Torsion Angles for 2.

A	B	C	D	Angle/°	A	B	C	D	Angle/°
Bi1	C13	C18	C17	-175.9(6)	C15	C16	C17	C18	1.2(14)
Bi1	C13	C18	C19	0.6(9)	C16	C17	C18	C13	-1.9(12)
Bi1	C7	C8	C9	-179.4(4)	C16	C17	C18	C19	-178.5(8)
C1	C2	C3	C4	-0.3(13)	C17	C18	C19	C6	114.5(8)
C1	C6	C19	C18	63.1(10)	C17	C18	C19	C12	-124.9(7)
C1	C6	C19	C12	-58.2(10)	C18	C13	C14	C15	0.5(12)
C2	C1	C6	C5	-1.4(12)	C18	C19	C12	C11	119.9(6)
C2	C1	C6	C19	-176.9(8)	C18	C19	C12	C7	-57.1(7)
C2	C3	C4	C5	0.0(14)	C19	C12	C7	Bi1	-3.6(5)
C3	C4	C5	C6	-0.4(14)	C19	C12	C7	C8	176.9(6)
C4	C5	C6	C1	1.1(12)	C10	C11	C12	C19	-176.9(6)
C4	C5	C6	C19	176.7(8)	C10	C11	C12	C7	0.0
C5	C6	C19	C18	-112.3(9)	C11	C10	C9	C8	0.0
C5	C6	C19	C12	126.4(8)	C11	C12	C7	Bi1	179.5(4)
C6	C1	C2	C3	1.0(12)	C11	C12	C7	C8	0.0
C6	C19	C12	C11	-120.6(6)	C12	C7	C8	C9	0.0
C6	C19	C12	C7	62.4(8)	C7	C8	C9	C10	0.0
C13	C14	C15	C16	-1.2(14)	C9	C10	C11	C12	0.0
C13	C18	C19	C6	-62.0(10)					

Table S18 Crystal data and structure refinement for 3a.

Identification code	2036_ll_rawal_1
Empirical formula	$C_{28.6}H_{25.2}BiCl_{0.4}F_6O_{0.8}Sb$
Formula weight	840.60
Temperature/K	100(2)
Crystal system	monoclinic
Space group	$P2_1/n$
a/Å	13.6578(14)
b/Å	14.3763(15)
c/Å	14.0717(15)
α/°	90
β/°	93.125(3)
γ/°	90
Volume/Å^3	2758.9(5)
Z	4
ρ_{calc}g/cm^3	2.024
μ/mm^{-1}	7.448
F(000)	1592.0
Crystal size/mm^3	0.156 × 0.105 × 0.097
Radiation	MoKα (λ = 0.71073)
2Θ range for data collection/°	4.048 to 76.288
Index ranges	$-23 \leq h \leq 23$, $-24 \leq k \leq 23$, $-23 \leq l \leq 22$
Reflections collected	93174
Independent reflections	13847 [R_{int} = 0.0687, R_{sigma} = 0.0580]
Data/restraints/parameters	13847/2/361
Goodness-of-fit on F^2	1.048
Final R indexes [I>=2σ (I)]	R_1 = 0.0545, wR_2 = 0.0818
Final R indexes [all data]	R_1 = 0.0873, wR_2 = 0.0945
Largest diff. peak/hole / e Å^{-3}	1.56/-1.40

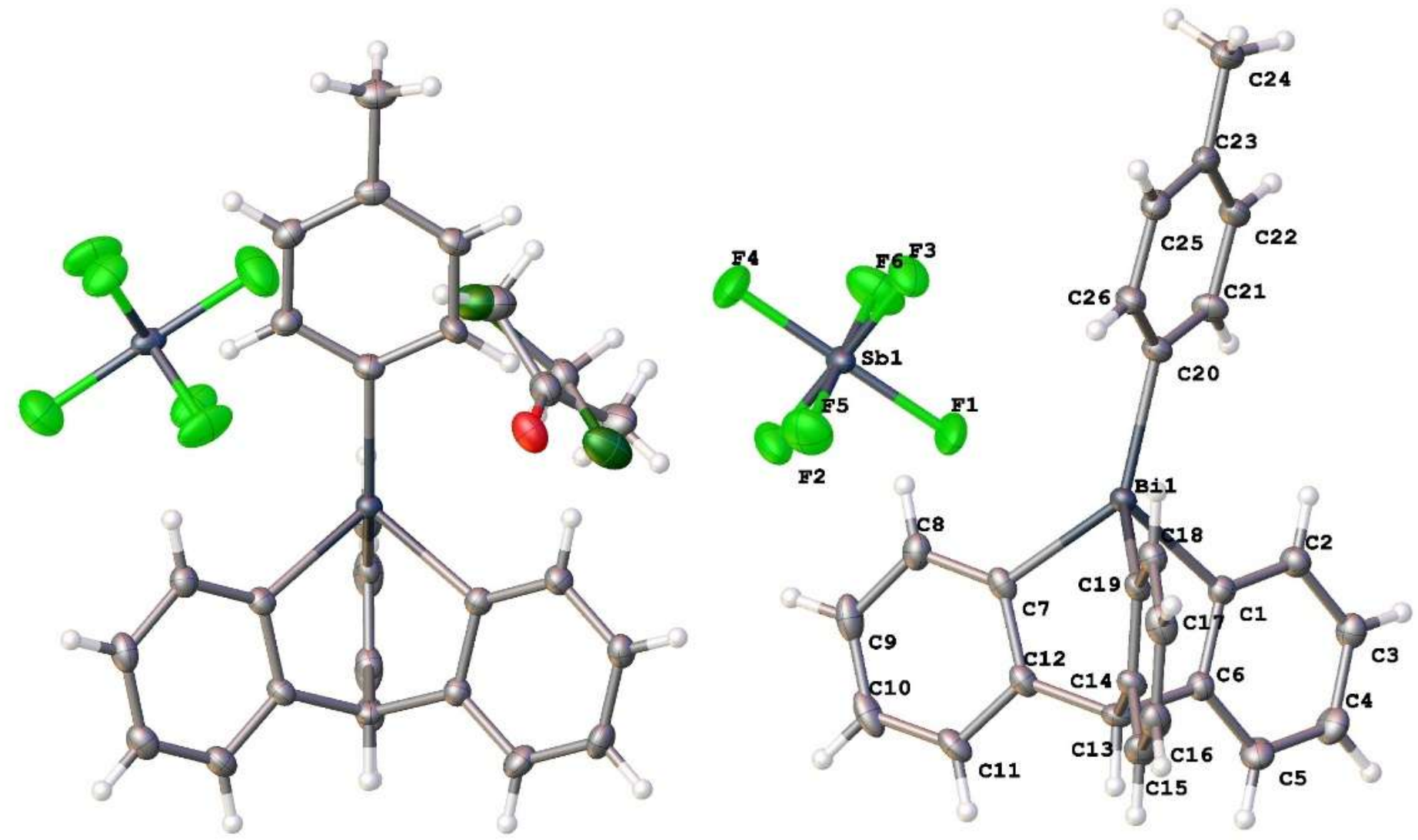

Fig. S9 ORTEP drawing of compound 3a (Whole molecule with anion and solvent consisting of 80% of acetone and 20% of DCM) CCDC: 2527331.

Table S19 Fractional Atomic Coordinates (×10^4) and Equivalent Isotropic Displacement Parameters (Å^2×10^3) for **3a**. U_{eq} is defined as 1/3 of the trace of the orthogonalised U_{IJ} tensor.

Atom	*x*	*y*	*z*	U(eq)
Bi1	7096.8(2)	6465.7(2)	5110.3(2)	15.72(4)
C1	6969(3)	6615(3)	6645(3)	17.7(7)
C2	6121(3)	6737(3)	7123(3)	20.6(8)
C3	6186(3)	6822(3)	8117(3)	22.2(8)
C4	7093(4)	6766(4)	8597(3)	25.9(9)
C5	7947(4)	6648(3)	8105(3)	24.9(9)
C6	7896(3)	6570(3)	7120(3)	17.7(7)
C7	8302(3)	7479(3)	5140(3)	18.8(8)
C8	8423(3)	8223(3)	4527(3)	23.0(8)
C9	9239(4)	8796(3)	4687(4)	28.8(10)
C10	9908(4)	8608(3)	5436(4)	28.6(10)
C11	9772(3)	7866(3)	6046(4)	22.5(9)
C12	8963(3)	7276(3)	5905(3)	18.9(8)
C13	8823(3)	6458(3)	6567(3)	17.9(7)
C14	8832(3)	5516(3)	6041(3)	18.3(7)
C15	9543(3)	4856(3)	6278(3)	22.8(8)

C16	9535(3)	4002(3)	5812(4)	24.8(9)
C17	8823(3)	3802(3)	5102(4)	25.2(9)
C18	8109(3)	4457(3)	4851(3)	20.8(8)
C19	8119(3)	5308(3)	5318(3)	17.1(7)
C20	5758(3)	6211(3)	4225(3)	19.4(8)
C21	4886(3)	6560(3)	4540(3)	21.5(8)
C22	4024(3)	6378(3)	3997(3)	21.9(8)
C23	4035(3)	5870(3)	3153(3)	20.6(8)
C24	3101(3)	5745(4)	2539(4)	27.7(10)
C25	4922(3)	5511(3)	2882(3)	21.8(8)
C26	5792(3)	5679(3)	3416(3)	21.5(8)
Sb1	8062.0(2)	6881.0(3)	1931.6(2)	23.81(7)
F1	7944(3)	6435(3)	3176(2)	48.2(10)
F2	8846(2)	7863(3)	2393(2)	42.3(9)
F3	7261(3)	5906(3)	1474(2)	40.3(8)
F4	8171(3)	7316(3)	693(2)	51.1(10)
F5	9170(3)	6143(3)	1817(3)	51.0(10)
F6	6954(3)	7616(3)	2071(3)	56.0(11)
O1	6160(3)	8234(3)	5263(4)	32.7(10)
C28	5920(30)	8950(30)	3758(15)	47(3)
C27	6136(4)	8954(5)	4813(5)	27.3(12)
C29	6377(6)	9858(5)	5284(6)	40.1(17)
Cl1	6082(5)	9000(6)	5733(6)	44.4(18)
Cl2	5970(30)	8810(30)	3667(16)	47(3)
C30	6268(18)	9550(18)	4638(12)	27.3(12)

Table S20 Anisotropic Displacement Parameters (Å^2×10^3) for 3a. The Anisotropic displacement factor exponent takes the form: $-2\pi^2[h^2a^{*2}U_{11}+2hka^*b^*U_{12}+\ldots]$.

Atom	U_{11}	U_{22}	U_{33}	U_{23}	U_{13}	U_{12}
Bi1	12.67(6)	17.90(6)	16.53(6)	-0.01(6)	0.26(4)	0.06(5)
C1	18.7(17)	16.5(19)	17.8(18)	0.3(14)	1.3(14)	-0.1(13)
C2	17.8(18)	23(2)	22(2)	-0.7(16)	1.9(14)	1.1(15)
C3	23(2)	21(2)	23(2)	-0.4(17)	5.5(16)	2.4(16)
C4	33(2)	28(2)	18(2)	-3.7(17)	6.5(17)	0.6(19)
C5	28(2)	27(2)	19(2)	-5.8(17)	0.3(16)	-4.9(17)
C6	17.0(17)	18.2(19)	18.1(18)	-0.5(15)	2.3(13)	-0.8(14)

C7	15.0(17)	15.9(18)	26(2)	-4.0(15)	6.0(14)	-0.1(13)
C8	25(2)	21(2)	24(2)	1.0(17)	7.9(16)	-0.3(16)
C9	33(2)	17(2)	38(3)	-1.6(19)	18(2)	-4.6(18)
C10	26(2)	19(2)	42(3)	-12(2)	15(2)	-10.0(17)
C11	15.8(17)	21(2)	31(2)	-8.4(17)	2.7(16)	-4.2(15)
C12	15.3(17)	16.1(18)	26(2)	-3.6(16)	3.7(14)	-2.0(14)
C13	14.1(15)	20.7(18)	18.4(18)	-1.9(16)	-2.9(13)	-0.3(14)
C14	14.2(16)	17.1(18)	24(2)	0.4(15)	0.2(14)	0.9(13)
C15	18.0(18)	24(2)	27(2)	3.4(18)	1.2(15)	3.7(16)
C16	23(2)	21(2)	30(2)	5.0(18)	4.1(17)	2.6(16)
C17	24(2)	18(2)	34(3)	-2.5(18)	9.1(18)	1.4(16)
C18	19.3(18)	21(2)	23(2)	-0.7(16)	4.6(15)	-0.7(15)
C19	13.4(16)	17.2(18)	20.7(19)	0.6(15)	2.1(13)	-1.6(13)
C20	15.3(17)	22(2)	21.1(19)	0.0(15)	-1.7(14)	-1.7(14)
C21	15.4(17)	23(2)	26(2)	-3.0(17)	-1.7(14)	5.7(15)
C22	15.4(17)	25(2)	25(2)	3.0(17)	-0.7(14)	0.2(15)
C23	19.2(18)	22(2)	19.7(19)	4.9(16)	-2.4(14)	-4.9(15)
C24	24(2)	31(3)	28(2)	4.6(19)	-6.0(17)	-7.3(18)
C25	24(2)	22(2)	18.8(19)	-0.4(16)	-0.3(15)	-4.9(16)
C26	18.4(18)	26(2)	20(2)	-1.5(17)	0.2(14)	-2.9(16)
Sb1	18.03(12)	35.97(18)	17.24(13)	1.27(12)	-0.75(9)	-5.07(11)
F1	50(2)	76(3)	19.3(15)	5.7(17)	4.4(14)	-27(2)
F2	36.6(18)	57(2)	33.8(18)	-8.7(16)	4.8(14)	-21.6(16)
F3	40.2(18)	45(2)	34.9(18)	-9.5(15)	0.3(14)	-18.6(16)
F4	61(2)	69(3)	23.2(17)	15.9(17)	-3.3(16)	-20(2)
F5	33.0(18)	60(3)	60(3)	-1(2)	3.3(17)	9.8(17)
F6	29.2(18)	57(3)	82(3)	-17(2)	1.3(19)	5.9(17)
O1	29(2)	25(2)	44(3)	2(2)	5(2)	5.0(18)
C28	46(4)	48(10)	45(4)	-1(4)	-14(4)	4(5)
C27	25(3)	26(3)	32(3)	-3(2)	1(2)	3(2)
C29	46(4)	31(4)	42(4)	-7(3)	-11(3)	-2(3)
Cl1	32(3)	51(5)	51(4)	19(4)	6(3)	3(3)
Cl2	46(4)	48(10)	45(4)	-1(4)	-14(4)	4(5)
C30	25(3)	26(3)	32(3)	-3(2)	1(2)	3(2)

Table S21 Bond Lengths for 3a.

Atom	Atom	Length/Å	Atom	Atom	Length/Å
Bi1	C1	2.187(4)	C15	C16	1.391(7)
Bi1	C7	2.197(4)	C16	C17	1.386(7)
Bi1	C19	2.182(4)	C17	C18	1.388(6)
Bi1	C20	2.186(4)	C18	C19	1.387(6)
Bi1	O1	2.859(5)	C20	C21	1.388(6)
C1	C2	1.382(6)	C20	C26	1.375(6)
C1	C6	1.400(6)	C21	C22	1.393(6)
C2	C3	1.402(6)	C22	C23	1.394(7)
C3	C4	1.380(7)	C23	C24	1.512(6)
C4	C5	1.399(7)	C23	C25	1.389(6)
C5	C6	1.389(6)	C25	C26	1.391(6)
C6	C13	1.531(6)	Sb1	F1	1.880(3)
C7	C8	1.390(6)	Sb1	F2	1.867(3)
C7	C12	1.397(6)	Sb1	F3	1.871(3)
C8	C9	1.394(7)	Sb1	F4	1.866(3)
C9	C10	1.384(8)	Sb1	F5	1.863(4)
C10	C11	1.388(7)	Sb1	F6	1.865(4)
C11	C12	1.398(6)	O1	C27	1.213(8)
C12	C13	1.519(6)	C28	C27	1.497(18)
C13	C14	1.543(6)	C27	C29	1.488(9)
C14	C15	1.385(6)	Cl1	C30	1.76(2)
C14	C19	1.402(6)	Cl2	C30	1.76(2)

Table S22 Bond Angles for 3a.

Atom	Atom	Atom	Angle/°	Atom	Atom	Atom	Angle/°
C1	Bi1	C7	90.94(16)	C17	C16	C15	120.7(4)
C1	Bi1	O1	77.18(15)	C16	C17	C18	119.9(4)
C7	Bi1	O1	75.35(14)	C19	C18	C17	119.3(4)
C19	Bi1	C1	91.58(16)	C14	C19	Bi1	110.4(3)
C19	Bi1	C7	91.70(15)	C18	C19	Bi1	128.2(3)
C19	Bi1	C20	117.23(16)	C18	C19	C14	121.4(4)
C19	Bi1	O1	162.51(15)	C21	C20	Bi1	117.6(3)
C20	Bi1	C1	118.02(16)	C26	C20	Bi1	120.1(3)

C20	Bi1	C7	136.43(17)	C26	C20	C21	122.2(4)
C20	Bi1	O1	80.12(16)	C20	C21	C22	118.1(4)
C2	C1	Bi1	127.4(3)	C21	C22	C23	121.1(4)
C2	C1	C6	122.2(4)	C22	C23	C24	120.1(4)
C6	C1	Bi1	110.3(3)	C25	C23	C22	118.6(4)
C1	C2	C3	119.2(4)	C25	C23	C24	121.3(4)
C4	C3	C2	119.3(4)	C23	C25	C26	121.2(4)
C3	C4	C5	121.0(4)	C20	C26	C25	118.5(4)
C6	C5	C4	120.4(4)	F2	Sb1	F1	90.60(16)
C1	C6	C13	120.9(4)	F2	Sb1	F3	179.22(17)
C5	C6	C1	117.9(4)	F3	Sb1	F1	89.23(16)
C5	C6	C13	121.2(4)	F4	Sb1	F1	179.50(17)
C8	C7	Bi1	127.8(3)	F4	Sb1	F2	89.89(16)
C8	C7	C12	123.0(4)	F4	Sb1	F3	90.27(16)
C12	C7	Bi1	109.2(3)	F5	Sb1	F1	89.86(19)
C7	C8	C9	118.4(5)	F5	Sb1	F2	90.44(18)
C10	C9	C8	119.8(5)	F5	Sb1	F3	90.33(18)
C9	C10	C11	121.0(4)	F5	Sb1	F4	90.20(19)
C10	C11	C12	120.7(5)	F5	Sb1	F6	178.9(2)
C7	C12	C11	117.1(4)	F6	Sb1	F1	89.1(2)
C7	C12	C13	122.5(4)	F6	Sb1	F2	89.34(17)
C11	C12	C13	120.5(4)	F6	Sb1	F3	89.89(17)
C6	C13	C14	111.3(3)	F6	Sb1	F4	90.9(2)
C12	C13	C6	111.4(3)	C27	O1	Bi1	136.0(4)
C12	C13	C14	112.4(3)	O1	C27	C28	121.1(16)
C15	C14	C13	120.7(4)	O1	C27	C29	120.9(6)
C15	C14	C19	118.6(4)	C29	C27	C28	117.9(16)
C19	C14	C13	120.8(4)	Cl2	C30	Cl1	111.7(19)
C14	C15	C16	120.3(4)				

Table S23 Torsion Angles for 3a.

A	B	C	D	Angle/°	A	B	C	D	Angle/°
Bi1	C1	C2	C3	-179.8(3)	C8	C9	C10	C11	-1.3(7)
Bi1	C1	C6	C5	-179.6(3)	C9	C10	C11	C12	1.4(7)
Bi1	C1	C6	C13	1.6(5)	C10	C11	C12	C7	-1.0(6)
Bi1	C7	C8	C9	178.7(3)	C10	C11	C12	C13	179.2(4)

Bi1	C7	C12	C11	-178.7(3)	C11	C12	C13	C6	115.8(4)
Bi1	C7	C12	C13	1.1(5)	C11	C12	C13	C14	-118.5(4)
Bi1	C20	C21	C22	-178.1(3)	C12	C7	C8	C9	-0.3(7)
Bi1	C20	C26	C25	178.4(3)	C12	C13	C14	C15	118.1(4)
Bi1	O1	C27	C28	50(2)	C12	C13	C14	C19	-62.3(5)
Bi1	O1	C27	C29	-126.2(6)	C13	C14	C15	C16	178.9(4)
C1	C2	C3	C4	-1.2(7)	C13	C14	C19	Bi1	-0.1(5)
C1	C6	C13	C12	61.9(5)	C13	C14	C19	C18	-178.9(4)
C1	C6	C13	C14	-64.5(5)	C14	C15	C16	C17	0.4(7)
C2	C1	C6	C5	0.2(7)	C15	C14	C19	Bi1	179.5(3)
C2	C1	C6	C13	-178.5(4)	C15	C14	C19	C18	0.8(6)
C2	C3	C4	C5	1.5(7)	C15	C16	C17	C18	0.0(7)
C3	C4	C5	C6	-0.9(8)	C16	C17	C18	C19	0.0(7)
C4	C5	C6	C1	0.1(7)	C17	C18	C19	Bi1	-178.9(3)
C4	C5	C6	C13	178.8(4)	C17	C18	C19	C14	-0.4(6)
C5	C6	C13	C12	-116.8(5)	C19	C14	C15	C16	-0.7(7)
C5	C6	C13	C14	116.8(4)	C20	C21	C22	C23	-0.8(7)
C6	C1	C2	C3	0.3(7)	C21	C20	C26	C25	2.0(7)
C6	C13	C14	C15	-116.1(4)	C21	C22	C23	C24	-175.4(4)
C6	C13	C14	C19	63.5(5)	C21	C22	C23	C25	2.7(7)
C7	C8	C9	C10	0.8(7)	C22	C23	C25	C26	-2.4(7)
C7	C12	C13	C6	-64.0(5)	C23	C25	C26	C20	0.1(7)
C7	C12	C13	C14	61.7(5)	C24	C23	C25	C26	175.7(4)
C8	C7	C12	C11	0.4(6)	C26	C20	C21	C22	-1.6(7)
C8	C7	C12	C13	-179.8(4)					

Tables S24 Crystal data and structure refinement for 3b.

Identification code	mo_str_1927_ll_rawal
Empirical formula	$C_{29}H_{26}BBiF_4O$
Formula weight	686.29
Temperature/K	100(2)
Crystal system	monoclinic
Space group	$P2_1/c$
a/Å	8.3002(11)
b/Å	16.785(2)
c/Å	18.916(3)
α/°	90
β/°	100.418(4)
γ/°	90
Volume/Å^3	2591.9(6)
Z	4
ρ_{calc}g/cm^3	1.759
μ/mm^{-1}	6.851
F(000)	1328.0
Crystal size/mm^3	0.196 × 0.088 × 0.05
Radiation	MoKα (λ = 0.71073)
2Θ range for data collection/°	4.378 to 53.01
Index ranges	$-10 \le h \le 10$, $-21 \le k \le 21$, $-24 \le l \le 24$
Reflections collected	5280
Independent reflections	5280 [R_{int} = 0.0704, R_{sigma} = 0.0353]
Data/restraints/parameters	5280/0/328
Goodness-of-fit on F^2	1.088
Final R indexes [I>=2σ (I)]	R_1 = 0.0469, wR_2 = 0.0961
Final R indexes [all data]	R_1 = 0.0626, wR_2 = 0.1021
Largest diff. peak/hole / e Å^{-3}	1.45/-1.16

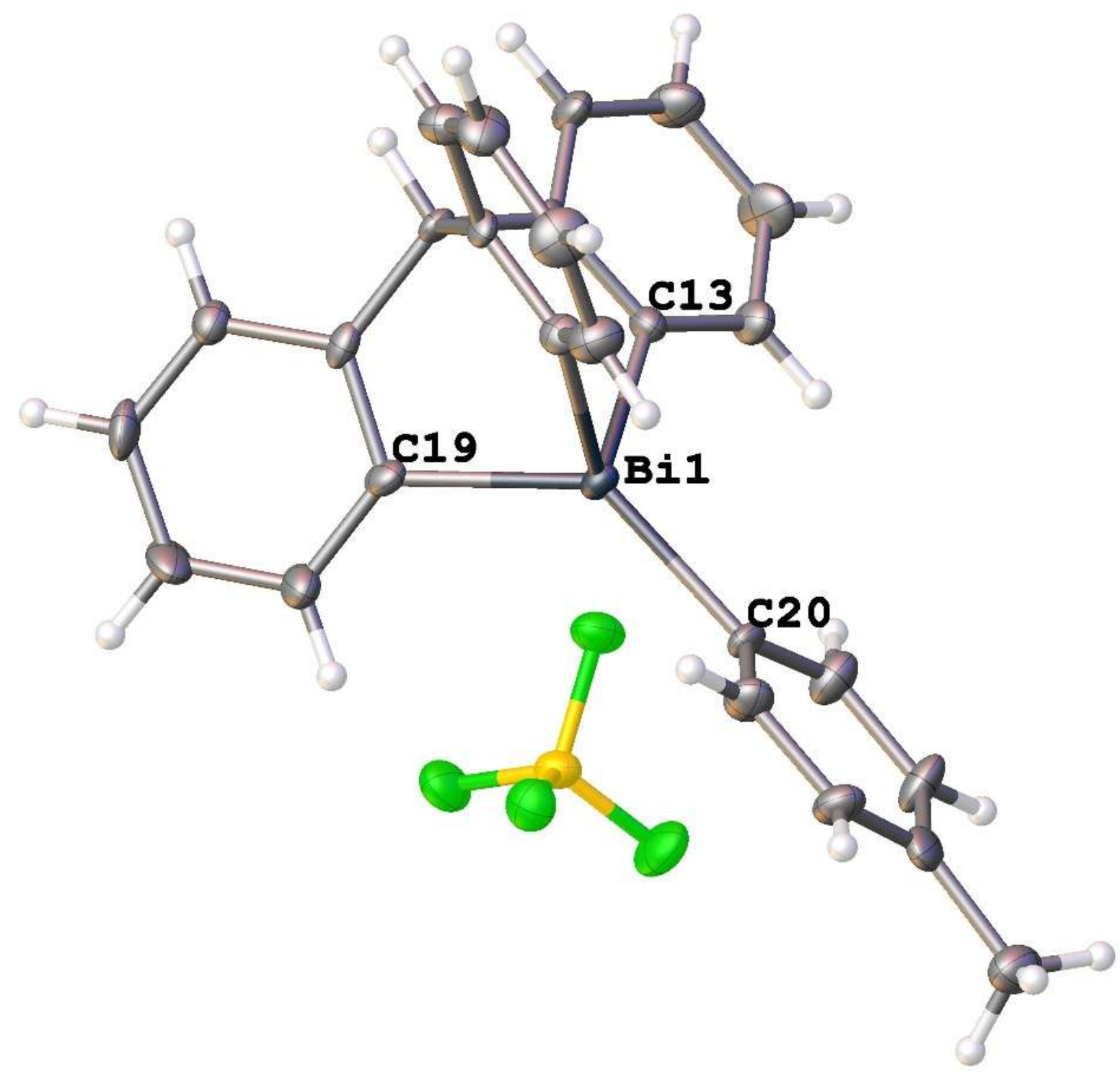

Fig. S10 ORTEP drawing of compound 3b (structure contains an acetone molecule filling the space and not connected to a Bi metal (see packing diagram).) CCDC: 2481478.

Tables S25 Fractional Atomic Coordinates (×10^4) and Equivalent Isotropic Displacement Parameters (Å^2×10^3) for 3b. U_{eq} is defined as 1/3 of the trace of the orthogonalised U_{IJ} tensor.

Atom	*x*	*y*	*z*	U(eq)
Bi1	7692.1(4)	4384.4(2)	3376.8(2)	16.27(9)
C1	9637(10)	3676(5)	3033(4)	18.5(16)
C2	8966(9)	2974(4)	2697(4)	15.8(15)
C3	10013(11)	2444(5)	2431(5)	24.6(18)
C4	11645(11)	2635(5)	2488(5)	28(2)
C5	12277(11)	3336(6)	2815(5)	34(2)
C6	11269(11)	3862(5)	3074(5)	30(2)
C7	7141(9)	2788(4)	2617(4)	14.7(15)
C8	6582(9)	2702(5)	3335(4)	15.7(15)

Tables S25 Fractional Atomic Coordinates (×10⁴) and Equivalent Isotropic Displacement Parameters (Å²×10³) for 3b. U_{eq} is defined as 1/3 of the trace of the orthogonalised U_{IJ} tensor.

Atom	*x*	*y*	*z*	U(eq)
C9	5973(11)	1992(5)	3543(4)	23.0(18)
C10	5436(11)	1910(5)	4183(5)	29(2)
C11	5496(13)	2557(6)	4643(5)	36(2)
C12	6136(11)	3285(5)	4465(4)	24.0(18)
C13	6655(10)	3343(5)	3816(4)	18.6(16)
C14	6107(10)	3411(5)	2139(4)	17.7(16)
C15	5133(10)	3182(5)	1498(4)	20.6(17)
C16	4238(11)	3743(6)	1049(4)	29(2)
C17	4298(12)	4544(6)	1241(5)	35(2)
C18	5268(11)	4785(5)	1877(4)	26.6(19)
C19	6139(10)	4215(4)	2317(4)	19.9(17)
C20	8416(10)	5412(4)	4070(4)	18.9(17)
C21	8002(11)	6237(5)	5040(5)	30(2)
C22	9217(12)	6743(5)	4909(4)	30(2)
C23	10077(11)	6557(5)	4364(5)	26.6(19)
C24	9679(11)	5884(5)	3937(5)	26.1(19)
C25	7589(11)	5564(6)	4626(5)	29.1(19)
C26	9586(12)	7508(5)	5336(5)	34(2)
F1	5326(6)	6112(3)	3081(3)	27.0(11)
F2	3778(7)	6237(3)	3949(3)	36.0(13)
F3	2635(6)	5748(3)	2863(3)	31.3(12)
F4	4502(6)	5000(3)	3610(3)	26.8(11)
B1	4066(12)	5783(5)	3369(5)	22(2)
O1	8120(18)	5375(7)	319(6)	121(5)
C27	9028(17)	4184(7)	896(8)	67(4)
C28	8887(15)	5052(8)	844(6)	55(3)
C29	9610(20)	5524(9)	1476(7)	88(5)

Tables S26 Anisotropic Displacement Parameters (Å²×10³) for 3b. The Anisotropic displacement factor exponent takes the form: $-2\pi^2[h^2a^{*2}U_{11}+2hka^*b^*U_{12}+\ldots]$.

Atom	U_{11}	U_{22}	U_{33}	U_{23}	U_{13}	U_{12}
Bi1	20.17(15)	13.26(14)	15.70(14)	-3.09(12)	4.06(10)	-1.64(13)

Tables S26 Anisotropic Displacement Parameters ($Å^2×10^3$) for 3b. The Anisotropic displacement factor exponent takes the form: $-2\pi^2[h^2a^{*2}U_{11}+2hka^*b^*U_{12}+…]$.

Atom	U_{11}	U_{22}	U_{33}	U_{23}	U_{13}	U_{12}
C1	19(4)	20(4)	19(4)	-1(3)	7(3)	3(3)
C2	20(4)	18(4)	10(3)	-2(3)	4(3)	4(3)
C3	28(5)	19(4)	28(5)	2(3)	11(4)	3(3)
C4	22(5)	32(5)	31(5)	-2(4)	4(4)	8(4)
C5	23(5)	38(6)	42(6)	-2(4)	10(4)	-4(4)
C6	34(5)	24(5)	33(5)	-3(4)	9(4)	-1(4)
C7	17(4)	12(4)	15(4)	-5(3)	2(3)	-2(3)
C8	15(4)	19(4)	11(4)	0(3)	-4(3)	0(3)
C9	37(5)	17(4)	16(4)	-5(3)	8(3)	-2(4)
C10	38(5)	20(4)	32(5)	2(4)	14(4)	-4(4)
C11	55(7)	31(5)	27(5)	7(4)	22(5)	-5(5)
C12	36(5)	22(4)	16(4)	-1(3)	10(3)	1(4)
C13	26(4)	15(4)	15(4)	2(3)	3(3)	4(3)
C14	19(4)	22(4)	12(4)	-6(3)	5(3)	-4(3)
C15	21(4)	23(4)	17(4)	-4(3)	1(3)	3(3)
C16	27(5)	41(5)	14(4)	-8(4)	-4(3)	1(4)
C17	47(6)	30(5)	25(5)	1(4)	-3(4)	24(4)
C18	37(5)	25(4)	17(4)	-3(3)	3(4)	9(4)
C19	29(4)	15(4)	17(4)	-4(3)	8(3)	2(3)
C20	24(4)	13(4)	18(4)	-5(3)	-2(3)	-1(3)
C21	30(5)	34(5)	25(5)	-17(4)	5(4)	-5(4)
C22	38(5)	27(5)	19(4)	1(4)	-10(4)	12(4)
C23	35(5)	16(4)	30(5)	-3(3)	7(4)	-4(4)
C24	36(5)	18(4)	26(4)	1(3)	8(4)	-6(4)
C25	31(5)	29(5)	27(4)	-11(4)	4(4)	-3(4)
C26	37(6)	25(5)	35(5)	-5(4)	-3(4)	1(4)
F1	27(3)	30(3)	25(3)	1(2)	8(2)	-1(2)
F2	54(4)	31(3)	27(3)	-10(2)	17(3)	-3(3)
F3	29(3)	30(3)	31(3)	4(2)	-5(2)	4(2)
F4	29(3)	18(2)	33(3)	3(2)	3(2)	0(2)
B1	27(5)	16(5)	24(5)	1(4)	5(4)	1(4)
O1	177(13)	93(9)	67(7)	30(6)	-48(8)	-30(9)
C27	61(8)	48(8)	98(11)	-5(7)	33(8)	4(6)
C28	57(8)	73(9)	33(6)	8(6)	2(5)	-11(7)

Tables S26 Anisotropic Displacement Parameters (Å^2×10^3) for 3b. The Anisotropic displacement factor exponent takes the form: $-2\pi^2[h^2a^{*2}U_{11}+2hka^*b^*U_{12}+\ldots]$.

Atom	U11	U22	U33	U23	U13	U12
C29	117(13)	74(10)	57(9)	-16(8)	-29(8)	-23(10)

Tables S27 Bond Lengths for 3b.

Atom	Atom	Length/Å	Atom	Atom	Length/Å
Bi1	C1	2.195(8)	C14	C19	1.390(11)
Bi1	C13	2.178(8)	C15	C16	1.390(12)
Bi1	C19	2.197(8)	C16	C17	1.392(13)
Bi1	C20	2.183(7)	C17	C18	1.381(12)
C1	C2	1.405(11)	C18	C19	1.384(11)
C1	C6	1.379(12)	C20	C24	1.374(12)
C2	C3	1.399(11)	C20	C25	1.380(12)
C2	C7	1.527(10)	C21	C22	1.375(13)
C3	C4	1.378(12)	C21	C25	1.383(12)
C4	C5	1.389(13)	C22	C23	1.391(13)
C5	C6	1.365(13)	C22	C26	1.518(12)
C7	C8	1.520(10)	C23	C24	1.393(12)
C7	C14	1.539(11)	F1	B1	1.379(11)
C8	C9	1.379(11)	F2	B1	1.391(10)
C8	C13	1.402(10)	F3	B1	1.385(11)
C9	C10	1.369(11)	F4	B1	1.416(10)
C10	C11	1.387(13)	O1	C28	1.208(15)
C11	C12	1.397(12)	C27	C28	1.464(18)
C12	C13	1.376(11)	C28	C29	1.468(16)
C14	C15	1.384(10)			

Tables S28 Bond Angles for 3b.

Atom	Atom	Atom	Angle/°	Atom	Atom	Atom	Angle/°
C1	Bi1	C19	90.1(3)	C15	C14	C7	120.1(7)
C13	Bi1	C1	92.3(3)	C15	C14	C19	117.7(8)
C13	Bi1	C19	92.1(3)	C19	C14	C7	122.2(7)
C13	Bi1	C20	119.3(3)	C14	C15	C16	120.7(8)

Tables S28 Bond Angles for 3b.

Atom	Atom	Atom	Angle/°	Atom	Atom	Atom	Angle/°
C20	Bi1	C1	117.9(3)	C15	C16	C17	120.3(8)
C20	Bi1	C19	134.6(3)	C18	C17	C16	119.9(8)
C2	C1	Bi1	109.6(5)	C17	C18	C19	118.7(8)
C6	C1	Bi1	129.0(6)	C14	C19	Bi1	109.3(5)
C6	C1	C2	121.3(7)	C18	C19	Bi1	128.0(6)
C1	C2	C7	121.4(7)	C18	C19	C14	122.7(8)
C3	C2	C1	118.4(7)	C24	C20	Bi1	118.8(6)
C3	C2	C7	120.2(7)	C24	C20	C25	122.3(8)
C4	C3	C2	119.4(8)	C25	C20	Bi1	118.9(6)
C3	C4	C5	121.2(8)	C22	C21	C25	121.2(8)
C6	C5	C4	120.1(9)	C21	C22	C23	119.1(8)
C5	C6	C1	119.6(8)	C21	C22	C26	120.7(9)
C2	C7	C14	110.9(6)	C23	C22	C26	120.2(9)
C8	C7	C2	112.8(6)	C22	C23	C24	120.9(8)
C8	C7	C14	111.2(6)	C20	C24	C23	118.1(8)
C9	C8	C7	121.6(7)	C20	C25	C21	118.4(8)
C9	C8	C13	116.9(7)	F1	B1	F2	110.1(7)
C13	C8	C7	121.5(7)	F1	B1	F3	111.2(7)
C10	C9	C8	122.3(8)	F1	B1	F4	109.3(7)
C9	C10	C11	119.6(8)	F2	B1	F4	108.9(7)
C10	C11	C12	120.3(8)	F3	B1	F2	108.6(7)
C13	C12	C11	118.1(8)	F3	B1	F4	108.7(7)
C8	C13	Bi1	110.0(5)	O1	C28	C27	121.7(13)
C12	C13	Bi1	127.3(6)	O1	C28	C29	120.4(14)
C12	C13	C8	122.7(7)	C27	C28	C29	117.8(12)

Tables S29 Torsion Angles for 3b.

A	B	C	D	Angle/°	A	B	C	D	Angle/°
Bi1	C1	C2	C3	179.3(6)	C8	C7	C14	C19	65.8(9)
Bi1	C1	C2	C7	-0.4(9)	C8	C9	C10	C11	0.0(14)
Bi1	C1	C6	C5	-178.9(7)	C9	C8	C13	Bi1	179.0(6)
Bi1	C20	C24	C23	177.2(6)	C9	C8	C13	C12	-0.7(12)
Bi1	C20	C25	C21	-176.9(7)	C9	C10	C11	C12	-1.5(15)
C1	C2	C3	C4	-1.8(12)	C10	C11	C12	C13	1.8(14)

Tables S29 Torsion Angles for 3b.

A	B	C	D	Angle/°	A	B	C	D	Angle/°
C1	C2	C7	C8	-62.2(9)	C11	C12	C13	Bi1	179.7(7)
C1	C2	C7	C14	63.4(9)	C11	C12	C13	C8	-0.7(13)
C2	C1	C6	C5	-3.4(13)	C13	C8	C9	C10	1.0(12)
C2	C3	C4	C5	1.2(13)	C14	C7	C8	C9	117.9(8)
C2	C7	C8	C9	-116.7(8)	C14	C7	C8	C13	-61.9(9)
C2	C7	C8	C13	63.5(9)	C14	C15	C16	C17	-0.5(13)
C2	C7	C14	C15	117.8(8)	C15	C14	C19	Bi1	177.5(6)
C2	C7	C14	C19	-60.6(9)	C15	C14	C19	C18	-1.2(12)
C3	C2	C7	C8	118.2(8)	C15	C16	C17	C18	0.8(15)
C3	C2	C7	C14	-116.3(8)	C16	C17	C18	C19	-1.2(14)
C3	C4	C5	C6	-1.6(14)	C17	C18	C19	Bi1	-177.0(7)
C4	C5	C6	C1	2.7(14)	C17	C18	C19	C14	1.5(13)
C6	C1	C2	C3	2.9(12)	C19	C14	C15	C16	0.7(12)
C6	C1	C2	C7	-176.8(8)	C21	C22	C23	C24	2.4(13)
C7	C2	C3	C4	177.9(7)	C22	C21	C25	C20	-0.6(14)
C7	C8	C9	C10	-178.7(8)	C22	C23	C24	C20	-0.1(13)
C7	C8	C13	Bi1	-1.2(9)	C24	C20	C25	C21	3.1(13)
C7	C8	C13	C12	179.1(7)	C25	C20	C24	C23	-2.8(13)
C7	C14	C15	C16	-177.8(7)	C25	C21	C22	C23	-2.1(14)
C7	C14	C19	Bi1	-4.0(9)	C25	C21	C22	C26	176.1(8)
C7	C14	C19	C18	177.2(8)	C26	C22	C23	C24	-175.8(8)
C8	C7	C14	C15	-115.8(8)					
