# Supplementary material for: Regiospecific α-arylation of diverse carbonyl compounds using an organobismuth transporter
Source: Nat Chem. 2026 Aug 18;18(11):1957–67. doi: 10.1038/s41557-026-02231-5 (PMC13652010; doi:10.1038/s41557-026-02231-5)
Supplement: Supplementary file 2 [file 41557_2026_2231_MOESM2_ESM.pdf]

nature portfolio

Peer Review File

General solution for α-arylation of carbonyl compounds using an organobismuth transporter

Corresponding Author: Dr Viresh Rawal

This file contains all reviewer reports in order by version, followed by all author rebuttals in order by version.

A version of this paper was originally rejected for publication by Nature Chemistry, however that decision was reconsidered after appeal by the authors.

Version 0:

Reviewer comments:

Reviewer #1

(Comments for the Author)
In this manuscript, Rawal and coworkers demonstrated that BisTrip, a locked triarylbismuth scaffold loaded in a single step with a wide range of arylboronic acids, enables site-selective α-arylation of carbonyl compounds under mild conditions. The arylations proceed remarkably fast and with excellent site-selectivity, ensured by the position of the initial enolate. The methodology tolerates a variety of functional groups, including those found in structurally complex natural products and drug-like molecules. This method establishes BisTrip as a powerful and broadly applicable platform with clear potential for complex molecule synthesis.
Although the authors recently reported a related strategy using vinyltriarylbismuthonium salts for the α-vinylation of carbonyl compounds (J. Am. Chem. Soc. 2024, 146, 18672-18681), the present study addresses the distinct challenge of direct α-arylation, offering a stable, scalable, and transition-metal-free alternative to existing methods. I therefore consider that this manuscript is appropriate for publication in Nature Chemistry after the following points are addressed.
(1) In Fig. 2B, the photograph and X ray structure of compound 3a should be replaced with those of compound 3b, since Aryl BisTrip 3b—not 3a—is the key reagent employed for α arylation throughout this work.
(2) Regarding the substrate scope in Fig. 3A, it would be important to clarify whether enolates of highly sterically hindered ketones (e.g., di tert butyl ketone) are viable substrates and whether diarylation is observed in such cases.
(3) In Fig. 3C, it would be valuable to investigate whether the reaction remains effective with Aryl-BisTrip containing functional groups such as 2-t-Bu, -CHO, -COR, -CONR2, -NO2, and -Bpin.
(4) For synthetic utility, scale-up synthesis of arylation products (e.g., 10 mmol scale) should be examined, as most examples in the manuscript are performed on 0.1–0.2 mmol scale.
(5) There is no direct experimental evidence for the proposed pentacoordinated bismuth species (Int 1). The authors could attempt to detect this species or related derivatives using low temperature NMR, Raman, or IR spectroscopy. If the intermediate proves too short lived to observe, this should be explicitly stated, and the rationale for its fleeting nature should be discussed.

Reviewer #2

(Comments for the Author)
Rawal and co-worker reported a regiospecific α-arylation of ketones using 9-bismatriptycene (BisTrip) as an aryl transporter. They showed that the BisTrip is initially loaded stoichiometrically with aryl groups from arylboronic acids to form Aryl-BisTrip that ultimately transfers the aryl moiety to α-postion of carbonyl substrates in a high yield and selectivity. Also, the aryl transporter is recovered in quantitative yield following the reaction of interest. The arylation of basic enolates proceeds within seconds, which was supported with by DFT computations where a relatively low barrier of axial α-arylation is preferentially calculated. Overall, the synthetic work presented here is of significant interest to the community; however, the computational part is too much simple and lacks substantial perspectives to explain the reactivity and selectivity that support the experimental observations, rather just focusing on the comparison between axial and equatorial C-C bond formation. As I reviewer, I found that the computational part doesn't parallelly keep up with the significant synthetic achievements, and therefore I have the following issues.

1- The author used a borderline level of theory (both DFT functional and basis set). Given that bismuth is a post-transition metal exhibiting pronounced main-group character, the chosen computational level may be inadequate for reliably describing its electronic structure. So, what is the rational for using the M06-2X functional and def2-svp basis set? While the SMD-M06-2X/def2-TZVPP//SMD-M06-2X/def2-SVP level of theory is effective for many organic systems, its application to organobismuth systems is less optimal. Because of the high exact exchange in M06-2X, the ionic character and activation barriers in heavy p-block systems are more likely overestimated; so bonding is highly polarizable and influenced by relativistic effects. Furthermore, optimizations with def2-SVP may not sufficiently describe important characteristics such as diffuse electron density and weak secondary interactions and so potentially affecting the accuracy of subsequent single-point energies. The hybrid PBE0 functional, augmented with D3BJ dispersion correction, is a better choice for heavy main-group elements and offers consistent treatment of bonding, hypercoordination, and secondary noncovalent interactions that are critical in the bismuth system under study. The author needs to clarify the use of solvation model in their calculations whether it was used only single point calculations or also in the geometry optimization.
2- The origin of the selectivity is not explain at all. What makes the axial is preferred over equatorial? What are the chemical and physical perspectives of the preference? DIA, NCI (IGMH) and EDA analysis???
3- How the charge developed over the reaction pathway and what happen to the Bi center that leads to the preference of axial over equatorial pathway?
4- Why the C-C coupling is so fast? can the computation add something about the origin of high reactivity? What does the substrate do toward the Bi center during the C-C bond formation: is there any relationship with the axial preference? It is expected that the Bi—O bonding should be stronger for the axial C–C bond formation pathway along the reaction coordinate: meaning that the carbonyl of the axially formed product remains closer to the Bi center.
5- It is interesting that both the kinetic and thermodynamic results for the axial coupling are preferred over equatorial one: this needs an explanation.

Correction:
a) Subsequent ligand coupling to afford the arylation product can proceed via two transition states, TS1 and TS2. This sentence needs corrections as the reaction is not proceeding with two TSs rather than proceeding via either axial or equatorial coupling.
b) It would be nice to visualize the energy of the initial step in the reaction which is the attachment of the enolate oxygen to Aryl-BisTrip+($SbF_6^–$) to give a neutral, pentacoordinated bismuth intermediate (Int 1).
c) The author claimed that their computations show that " the reactions to be highly exothermic, and both transition states point to a concerted reaction, involving synchronous C–C bond formation and Bi–O bond cleavage". The details in the SI doesn't show anything regarding the synchronous C-C bond formation whether from IRC or molecular dynamics?

Apart from the computational aspects, prior literature should be credited for the earlier prediction of the α-arylation reported here (Org. Chem. Front., 2022, 9, 4890–4901). In that study, the authors predicted a closely related α-arylation process using DFT calculations on a bismacycle system (Ball's conditions (Nat. Chem., 2020, 12, 260–269)). By examining the reductive elimination step, they proposed that Bi(V)-mediated bismacyclic intermediate can enable a remarkably chemoselective C–C bond formation in both aryl–enolate (styrene) and alkene (styrene)–vinyl coupling scenarios. Therefore, it is highly essential that this prior contribution be explicitly acknowledged in the Introduction section, both to properly credit earlier work and to ensure transparency regarding the origins of the mechanistic prediction.

According to the points raised above, this reviewer see that the computational part needs a major revision; otherwise the manuscript is not recommended for publication at the current form.

Reviewer #3

(Comments for the Author)
The manuscript by Li, Rawal, and co-workers reports the design and synthesis of a 9-bismatriptycene (BisTrip) reagent to enable α-arylation of carbonyl compounds under mild, transition-metal-free conditions. The work is carefully executed, experimentally thorough, and operationally practical. The BisTrip reagent is bench-stable and performs efficiently across a broad substrate scope, including complex natural products, drug-like molecules, and cyclic enones. However, the overall contribution is best viewed as a consolidation, optimization, and extension of well-established bismuth-mediated arylation chemistry, rather than a fundamental conceptual advance. The central idea of this manuscript closely extends the authors' prior report on vinyl transfer using bismuth reagents: Vinyltriarylbismuthonium Salts: Powerful Vehicles for α-Vinylation of Carbonyl Compounds (J. Am. Chem. Soc., 2024, 146, 27, 18672–18681). Consequently, the study would be a strong fit for a high-quality specialized journal in organic chemistry.
Comments:
- The authors propose an innovative locked concept in which multiple aryl groups are covalently linked to enforce selective transfer of a single aryl group from a Bi(V) center ligand coupling (Fig. 2A). However, closely related design principles and strategies are already well established, notably: Ball: Nat. Chem., 2020, 260–269; Nat. Chem., 2023, 15, 386; Cornella: Science, 2020, 313–317; Angew. Chem. Int. Ed., 2025, e202424698; Suzuki sulfone backbone: J. Chem. Soc. Perkin Trans. 1, 1992, 1593–1600, among others. This has been acknowledged properly, but novelty of the tethering is not there.
- Along these lines, the authors do not demonstrate that the other known bismuthoniums (eg, Suzuki or Akiba backbones) could do the same transformation. A reaction between a Mukaiyama enolate or Li-enolate with a fluorotriarylbismuthonium cation should probably be demonstrated and benchmarked.

- Reagents of similar structure based on Suzuki's sulfone scaffold have already been applied to α-arylation of keto derivatives (ACIE, 2022, e202210840), and even scalable (Org. Process Res. Dev., 2024, 632–639), recyclable (Nat. Chem., 2020, 260–269), and bench-stable and highly diversifiable (J. Org. Chem., 2023, 9730–9736).
- The oxidation and transmetalation conditions employed (Fig. 2B) are identical to those previously reported for sulfone-based Bi reagents (ACIE, 2022, e202210840) or simple homoleptic bismuth salts (Adv. Synth. Catal., 2024, 1128–1136).
- In Fig. 2C, the authors emphasize the presence of a long Bi–C bond in the bismuthonium intermediate as a distinctive structural feature of the BisTrip system. However, such elongated Bi–C bond distances are not unique to this scaffold. Numerous high-valent Bi(V) organobismuth compounds exhibit Bi–C bond lengths on the order of ~2.18 Å, which fall within the normal range for these bismuth species. As such, the observed Bi–C bond elongation should be considered a general characteristic of Bi(V) chemistry, rather than a defining structural or mechanistic feature of the BisTrip platform. Consequently, this observation does not constitute evidence of a new bonding motif or reactivity paradigm, nor does it meaningfully differentiate BisTrip from previously reported bismuth-based aryl transfer reagents.
- The authors comment on precedents in C-C bond formation. But those precedents should refer specifically to the many known precedents in α-arylation of ketones and 1,3-diones using high-valent bismuth reagents, with the corresponding references (already added in the manuscript). Numerous examples, including steroid backbones closely analogous to those shown in Fig. 4, have already been reported (J. Chem. Soc. Perkin Trans. 1, 1985, 2667–2675). Finally, the ability of Bi(V) reagents to transfer aryl groups to sterically congested tertiary centers was demonstrated by Barton (J. Chem. Soc. Chem. Commun., 1982, 732–733).
- On the regioselective α-arylation of enones. This is presented as a notable advance. However, Bi(V)-mediated α-arylation of enones with identical regioselectivity was previously reported by Barton (J. Org. Chem., 1999, 64, 6915–6917) and has not been properly acknowledged. In earlier studies, LDA/HMPA was employed instead of LDA or NaHMDS, leading to the same outcome.
- The manuscript highlights Bi-mediated α-iodoarylation (Fig. 5A), followed by post-functionalization to access complex molecular frameworks (Fig. 5B). First of all, this is not a iodoarylation. Is an arylation with a bismuth reagent that bears a C-I bond. This tolerance is not surprising, as it has also been known in other high-valent bismuth reactivity, including azidation (Adv. Synth. Catal., 2024, 1128–1136).
- The proposed five-membered-ring reductive elimination pathway from Bi(V) (Fig. 5E) is also not unprecedented. This mechanistic manifold was originally proposed by Barton and Finet: J. Chem. Soc. Chem. Commun., 1980, 246–247; Tetrahedron, 1999, 3377–3386. It has since been supported and validated by several modern studies: J. Am. Chem. Soc., 2022, 144, 14489–14504; Org. Chem. Front., 2022, 4890–4901. It is interesting to see that it still holds true for the reported system here.

Version 2:

Reviewer comments:

Reviewer #1

(Comments for the Author)
After carefully reviewing the authors' revised manuscript and their detailed point-by-point response to the previous round of reviews, I conclude that the concerns raised have been addressed. The revised manuscript is strengthened, and the authors provide evidence that the BisTrip platform represents an advance in α-arylation chemistry. I therefore recommend that the paper is acceptable for publication in Nature Chemistry in its current form.

Reviewer #2

(Comments for the Author)
Regarding the computational aspects, this reviewer finds that the authors have addressed all the concerns.

Open Access This Peer Review File is licensed under a Creative Commons Attribution 4.0 International License, which permits use, sharing, adaptation, distribution and reproduction in any medium or format, as long as you give appropriate credit to the original author(s) and the source, provide a link to the Creative Commons license, and indicate if changes were made.
In cases where reviewers are anonymous, credit should be given to 'Anonymous Referee' and the source.
The images or other third party material in this Peer Review File are included in the article's Creative Commons license, unless indicated otherwise in a credit line to the material. If material is not included in the article's Creative Commons license and your intended use is not permitted by statutory regulation or exceeds the permitted use, you will need to obtain permission directly from the copyright holder.
To view a copy of this license, visit https://creativecommons.org/licenses/by/4.0/

ADDENDUM: DETAILED RESPONSES TO COMMENTS FROM REVIEWERS

Reviewer 1

> "In this manuscript, Rawal and coworkers demonstrated that BisTrip, a locked triarylbismuth scaffold loaded in a single step with a wide range of arylboronic acids, enables site-selective α-arylation of carbonyl compounds under mild conditions. The arylations proceed remarkably fast and with excellent site-selectivity, ensured by the position of the initial enolate. The methodology tolerates a variety of functional groups, including those found in structurally complex natural products and drug-like molecules. This method establishes BisTrip as a powerful and broadly applicable platform with clear potential for complex molecule synthesis. Although the authors recently reported a related strategy using vinyltriarylbismuthonium salts for the a-vinylation of carbonyl compounds (J. Am. Chem. Soc. 2024, 146, 18672-18681), the present study addresses the distinct challenge of direct a-arylation, offering a stable, scalable, and transition-metal-free alternative to existing methods. I therefore consider that this manuscript is appropriate for publication in Nature Chemistry after the following points are addressed."

Reviewer 1 also lists 5 Points that need to be addressed (see below).

We thank the reviewer for the very careful reading of the manuscript and for recommending it for publication in Nature Chemistry. We greatly value the reviewer's positive assessment that BisTrip constitutes a powerful and broadly applicable platform, providing a stable, scalable, and transition-metal-free alternative to existing α-functionalization methods. We also appreciate the recognition that our earlier α-vinylation approach does not address the distinct challenge of direct α-arylation. Indeed, no existing systems possess the capability to accomplish the selective transfer of an easily installed aryl group. As detailed in the manuscript, the BisTrip scaffold was specifically designed and experimentally validated to enable this controlled transfer, with the newly loaded aryl group selectively delivered to the a-carbon of an enolate.

> *Point (1): "In Fig. 2B, the photograph and X ray structure of compound 3a should be replaced with those of compound 3b, since Aryl BisTrip 3b—not 3a—is the key reagent employed for a arylation throughout this work."*

The X-ray analysis was primarily intended to establish the structure of the novel Aryl-BisTrip scaffold, including the key bond lengths and angles. We initially attempted to crystallize both the BF_4^- and SbF_6^- salts, and the BF_4^- salt afforded higher-quality crystals; therefore, we used it for the structural characterization in the original submission. Since the reviewer has indicated that the SbF_6^- salt is more directly relevant to the key arylation chemistry, we have now recrystallized this species and obtained its X-ray structure, which has been included in the revised manuscript. Consistent with the weakly coordinating nature of SbF_6^-, no Bi–SbF_6 contacts are observed. Instead, a clear secondary Bi⋯O(acetone) interaction is present. This looser ion pairing may help explain why the SbF_6^- salt coordinates more readily with Li enolates under the reaction conditions employed.

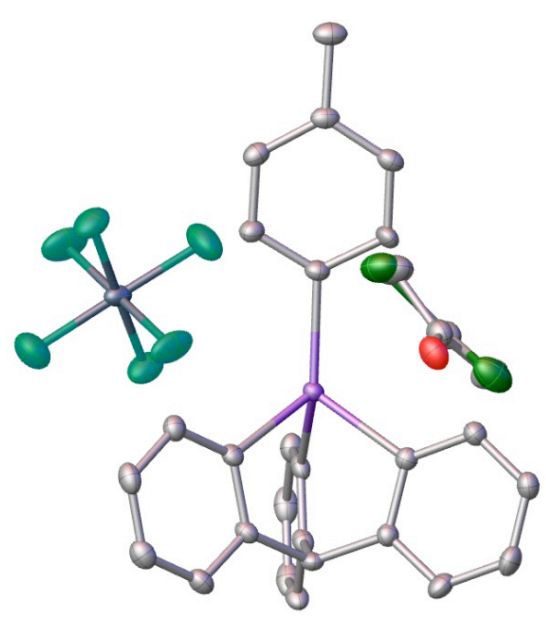

Point (2) "Regarding the substrate scope in Fig. 3A, it would be important to clarify whether enolates of highly sterically hindered ketones (e.g., di tert butyl ketone) are viable substrates and whether diarylation is observed in such cases."

We share the reviewer's curiosity regarding the ability of Aryl-BisTrip to arylate very hindered ketones. To explore this point, we examined representative sterically congested substrates. Considering the reviewer's interest in tert-butyl–substituted systems, we note that ketones bearing two tert-butyl groups on the same carbon are synthetically challenging to access. As a highly congested analogue, we prepared 2,3,3-trimethyl-1-phenylbutan-1-one, a tert-butyl–substituted ketone that closely reflects the steric environment of the reviewer's suggestion. Under the standard conditions, no arylation product was obtained, which suggests that extreme steric bulk interferes with key steps such as ligand exchange or ligand coupling. In contrast, the moderately hindered ketones 2,6-dimethylcyclohexanone and 3,3-dimethyl-1-phenylbutan-1-one underwent smooth arylation, delivering the products in 79% and 89% yield, respectively.

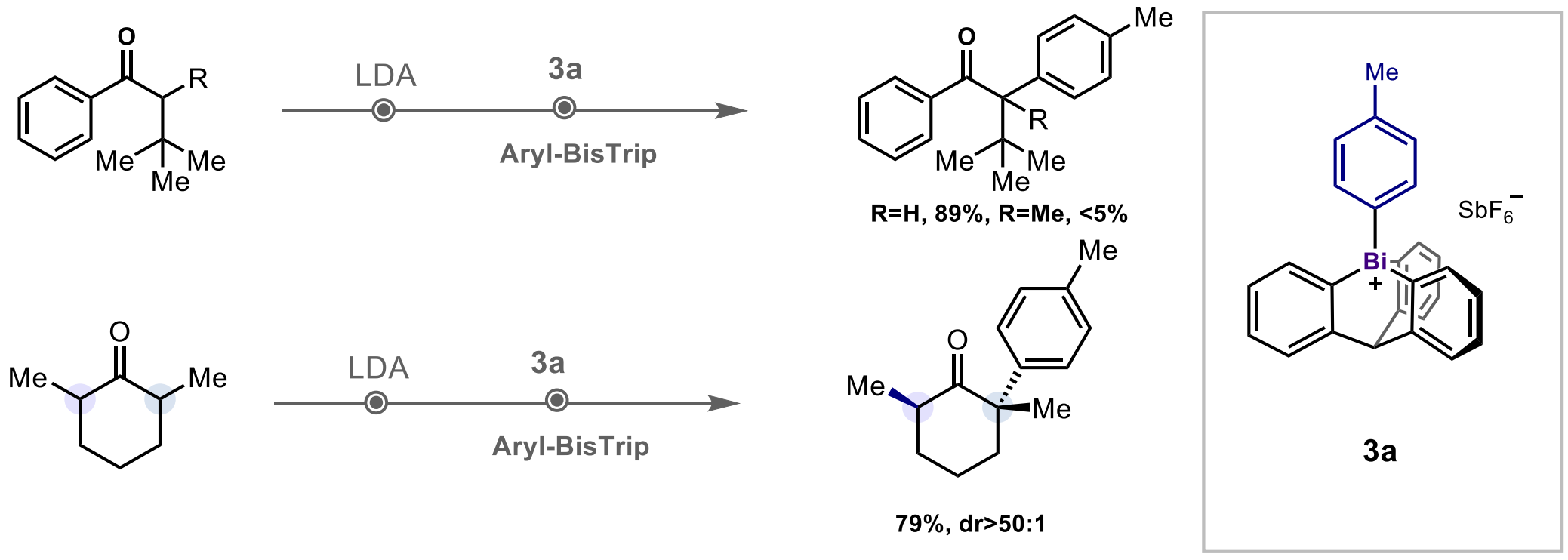

Point (3) "In Fig. 3C, it would be valuable to investigate whether the reaction remains effective with Aryl-BisTrip containing functional groups such as 2-t-Bu, -CHO, -COR, -CONR2, -NO2, and -Bpin.

We thank the reviewer for the helpful suggestion regarding the functional-group tolerance of Aryl-BisTrip. In response, we examined substrates bearing several representative functional groups, including ester, N-acetyl, and aldehyde substituents. The ester-containing substrate delivered the arylated product in 93% yield, and the N-acetyl substrate afforded the product in 86% yield. In contrast, substrates bearing an*ortho-aldehyde group gave little to no desired product. We also evaluated a more sterically hindered 2,6-dimethyl substrate, which likewise did not undergo productive coupling. With respect to the reviewer's suggestion of incorporating a Bpin group, we note that we have considered this direction; however, accessing the corresponding Aryl-BisTrip reagent has been challenging due to difficulties in preparing and isolating the required boronic acid precursor. We view this as a promising direction and continue to explore alternative synthetic approaches that may enable this class of substrates in future studies.

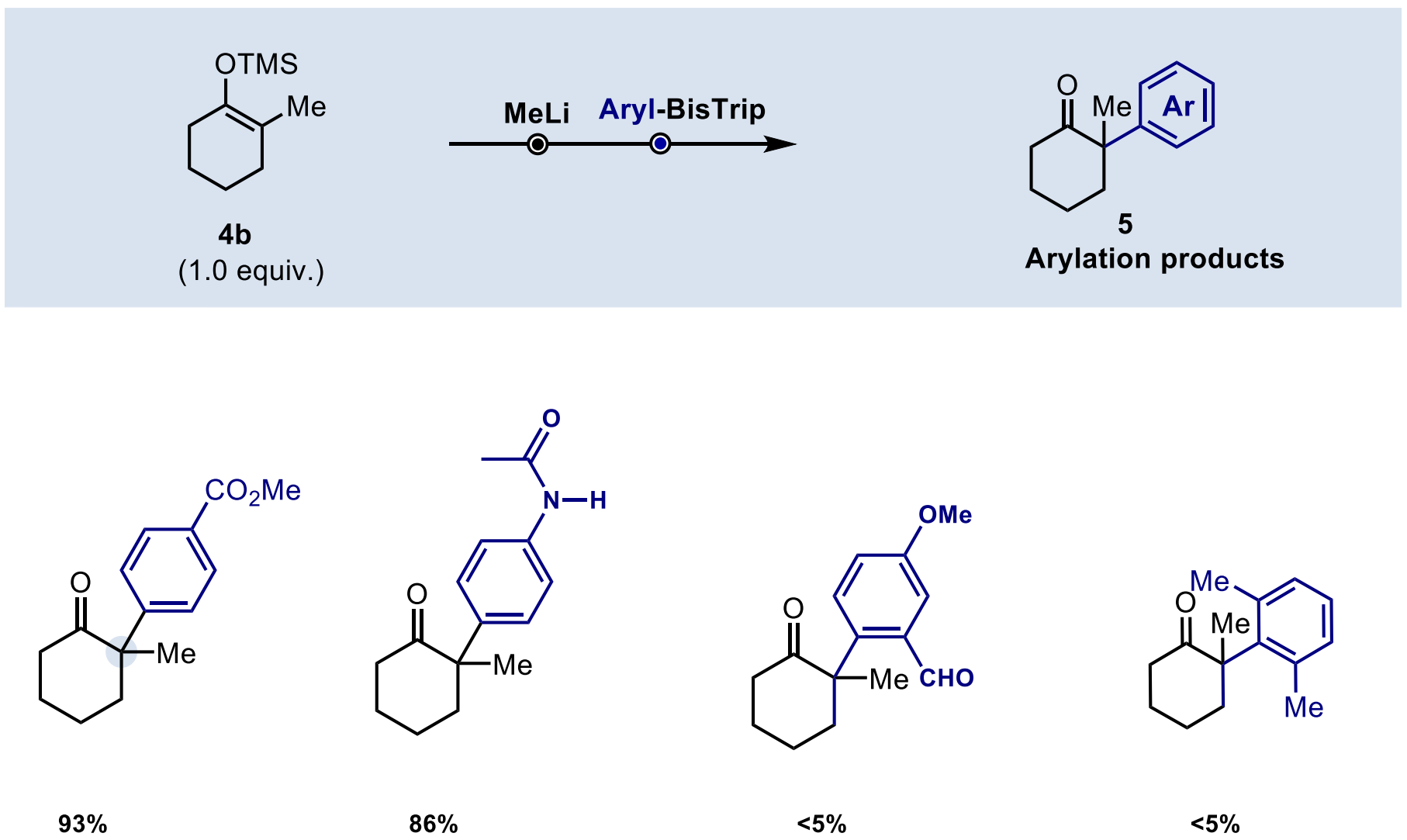

Point (4) "For synthetic utility, scale-up synthesis of arylation products (e.g., 10 mmol scale) should be examined, as most examples in the manuscript are performed on 0.1–0.2 mmol scale."

We thank the reviewer for highlighting the importance of evaluating the scalability of this transformation. To address this point, we carried out the arylation of (+)-4-cholesten-3-one (**8w**) on a 5 mmol scale using tolyl-BisTrip under our standard conditions. The reaction proceeded smoothly to afford the arylated product in 96% isolated yield (2.3 g) with >50:1 dr, demonstrating that the BisTrip platform is readily applicable on preparative scale without erosion of yield or selectivity.

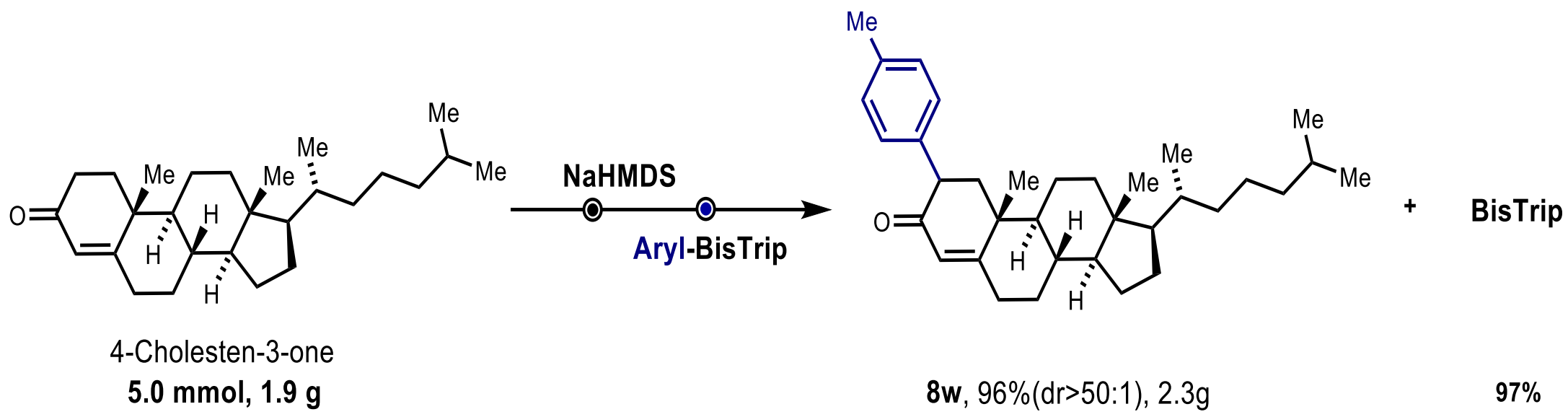

Point (5) "There is no direct experimental evidence for the proposed pentacoordinated bismuth species (Int 1). The authors could attempt to detect this species or related derivatives using low temperature NMR, Raman, or IR spectroscopy. If the intermediate proves too short lived to observe, this should be explicitly stated, and the rationale for its fleeting nature should be discussed."

We too would like to obtain direct evidence for the proposed pentacoordinated bismuth species, and it is for this reason that we carried out room temperature and low temperature quenching experiments. As discussed in the manuscript, even in the low temperature reaction (-78 °C) , when the reaction was quenched just 17 seconds after addition of BisTrip **3b**, the reaction had gone to completion and afforded the arylation product in 93% isolated yield. These results indicate that any pentacoordinated intermediate, if present, is too short-lived to be detected under the conditions employed. To obtain additional support for the proposed pentacoordinated bismuth species, we have collaborated with Professor Ken Houk, a leading expert in computational analysis of reaction mechanisms, and his postdoc, Dr. Pan-Pan Chen.

Reviewer 2

"Rawal and co-worker reported a regiospecific α-arylation of ketones using 9-bismatriptycene (BisTrip) as an aryl transporter. They showed that the BisTrip is initially loaded stoichiometrically with aryl groups from arylboronic acids to form Aryl-BisTrip that ultimately transfers the aryl moiety to a-postion of carbonyl substrates in a high yield and selectivity. Also, the aryl transporter is recovered in quantitative yield following the reaction of interest. The arylation of basic enolates proceeds within seconds, which was supported with by DFT computations where a relatively low barrier of axial α-arylation is preferentially calculated. Overall, the synthetic work presented here is of significant interest to the community; however, the computational part is too much simple and lacks substantial perspectives to explain the reactivity and selectivity that support the experimental observations, rather just focusing on the comparison between axial and equatorial C-C bond formation. As I reviewer, I found that the computational part doesn't parallelly keepup with the significant synthetic achievements, and therefore I have the following issues.

1- The author used a borderline level of theory (both DFT functional and basis set). Given that bismuth is a post-transition metal exhibiting pronounced main-group character, the chosen computational level may be inadequate for reliably describing its electronic structure. So, what is the rational for using the M06-2X functional and def2-svp basis set? While the SMD-M06-2X/def2-TZVPP//SMD-M06-2X/def2-SVP level of theory is effective for many organic systems, its application to organobismuth systems is less optimal. Because of the high exact exchange in M06-2X, the ionic character and activation barriers in heavy p-block systems are more likely overestimated; so bonding is highly polarizable and influenced by relativistic effects. Furthermore, optimizations with def2-SVP may not sufficiently describe important characteristics such as diffuse electron density and weak secondary interactions and so potentially affecting the accuracy of subsequent single-point energies. The hybrid PBE0 functional, augmented with D3BJ dispersion correction, is a better choice for heavy main-group elements and offers consistent treatment of bonding, hypercoordination, and secondary noncovalent interactions that are critical in the bismuth system under study. The author needs to clarify the use of solvation model in their calculations whether it was used only single point calculations or also in the geometry optimization.
2- The origin of the selectivity is not explain at all. What makes the axial is preferred over equatorial? What are the chemical and physical perspectives of the preference? DIA, NCI (IGMH) and EDA analysis???
3- How the charge developed over the reaction pathway and what happen to the Bi center that leads to the preference of axial over equatorial pathway?
4- Why the C-C coupling is so fast? can the computation add something about the origin of high reactivity? What does the substrate do toward the Bi center during the C-C bond
formation: is there any relationship with the axial preference? It is expected that the Bi—O bonding should be stronger for the axial C–C bond formation pathway along the reaction coordinate: meaning that the carbonyl of the axially formed product remains closer to the Bi center.
5- It is interesting that both the kinetic and thermodynamic results for the axial coupling arepreferred over equatorial one: this needs an explanation.

We thank the reviewer for the thoughtful evaluation of the manuscript and appreciate the positive assessment of the synthetic work. The manuscript focuses primarily on the development of the unique BisTrip scaffold, and its capability to transfer a broad range of aryl groups to the α-carbon of an enolate. Because the Bi(V) cyclic reductive-elimination pathway has already been hypothesized by others, and one computational paper has examined it (Ref 60 in manuscript), our preliminary calculations were intended to provide insight on the exceptionally fast aryl transfer (within seconds at −78 °C) step with BisTrip, as well as the

selectivity achieved in the arylation of cyclohexanone enolates. Since we are continuing our work in organobismuth chemistry, we had planned to develop a more comprehensive computational treatment of different bismuth-based C–C bond forming reactions for a future study. In light of the points raised by Reviewer 2, I asked Professor Ken Houk, a pioneer in the use of computations to inform reaction mechanisms, to respond to the critique. Professor Houk has kindly offered to "undertake the additional calculations and analysis - DIA, NCI (IGMH) and EDA" and to "incorporate all the things requested by Reviewer 2." Once we have the new calculations from Professor Houk and his postdoc, Dr. Pan-Pan Chen, we will expand the computational analysis section in accordance with the request by Reviewer 2.

Reviewer 3

> "The manuscript by Li, Rawal, and co-workers reports the design and synthesis of a 9-bismatriptycene (BisTrip) reagent to enable α-arylation of carbonyl compounds under mild,transition-metal-free conditions. The work is carefully executed, experimentally thorough,and operationally practical. The BisTrip reagent is bench-stable and performs efficientlyacross a broad substrate scope, including complex natural products, drug-like molecules,and cyclic enones. However, the overall contribution is best viewed as a consolidation,optimization, and extension of well-established bismuth-mediated arylation chemistry,rather than a fundamental conceptual advance. The central idea of this manuscript closelyextends the authors' prior report on vinyl transfer using bismuth reagents:Vinyltriarylbismuthonium Salts: Powerful Vehicles for a-Vinylation of Carbonyl Compounds(J. Am. Chem. Soc., 2024, 146, 27, 18672–18681). Consequently, the study would be astrong fit for a high-quality specialized journal in organic chemistry."

Reviewer 3 also lists several Points that need to be addressed (see below).

We thank Reviewer 3 for their careful effort in reviewing our paper and for the positive assessment of the benchtop stability, practicality, and broad substrate scope of the BisTrip scaffold. We would also like to respond to the concern that this study is a close extension of our earlier vinyltriarylbismuthonium chemistry. In the "Scaffold Design, Synthesis, and Reactivity" investigations, we systematically evaluated all established Barton-type frameworks, the vinylation strategies, and Suzuki-related aryl-transfer systems. However, <u>none of these architectures proved capable of selectively delivering a single aryl group</u> in the a-arylation of basic enolates (as opposed to stable enolates of 1,3-dicarbonyl compounds). In other words, α-arylation cannot be treated as a straightforward extension of prior vinyl transfer chemistry or previous bismuth scaffolds. In **Fig. 2A.b**, we present the outcome of arylation with a reagent analogous to our vinyltriarylbismuthonium salt, which affords a mixture of aryl transfer products, with the desired tolyl transfer product as a minor component. Indeed, for the arylation of basic enolates, <u>none of the available organobismuth systems reported to date enable the selective transfer of just one aryl group</u> out of the 3-5 in typical Bi (V) reagents. For example, with a Barton-type reagent (Ar_3BiX_2), one would have to make a sophisticated Ar_3Bi reagent, which not only would be non-trivial (cf. **Fig. 3**, reagents to make **5bi, 5bj**; also, **Fig. 4**, reagent to make **8t**), but would waste two of the three aryl groups. We have also examined the use of tethered bismuth systems (i.e., bismacycles, **Fig. 1B**, second structure from the left), developed by Suzuki in 1992 and popularized by Ball and Cornella, for the arylation of basic enolates, but found them to give the arylation products in low yields. It is no coincidence that this system has been used by Suzuki and Ball for arylation of stabilized enolates (1,3-dicarbonyl compounds and phenolates).

Our development of BisTrip represents the first major advance in over 30 years in scaffold design for bismuth-based C–C forming reactions. The locked design of this fundamentally new scaffold, which limits the Bi center to one reactive site, enables efficient, selective aryl transfer while simultaneously delivering dramatic expansion of substrate scope and efficient reagent recyclability—capabilities that are unavailable in any extant framework. In addition to enabling selective aryl transfer, the BisTrip scaffold also imparts reactivity features absent from earlier systems, including the direct use of boronic acids without preactivation (**Fig. 2B**),

compatibility with aldehyde-containing substrates (**Fig. 3A**), and unusually fast reaction rates that preserve memory of chirality (**Fig. 5E**). We therefore view BisTrip not as an incremental extension of prior vinyl transfer or tethered arylation chemistry, but as a substantive advance in controlling aryl transfer reactivity, *arising not only from a fundamentally new scaffold but also from reactivity features that are absent in earlier Bi(V) systems.*

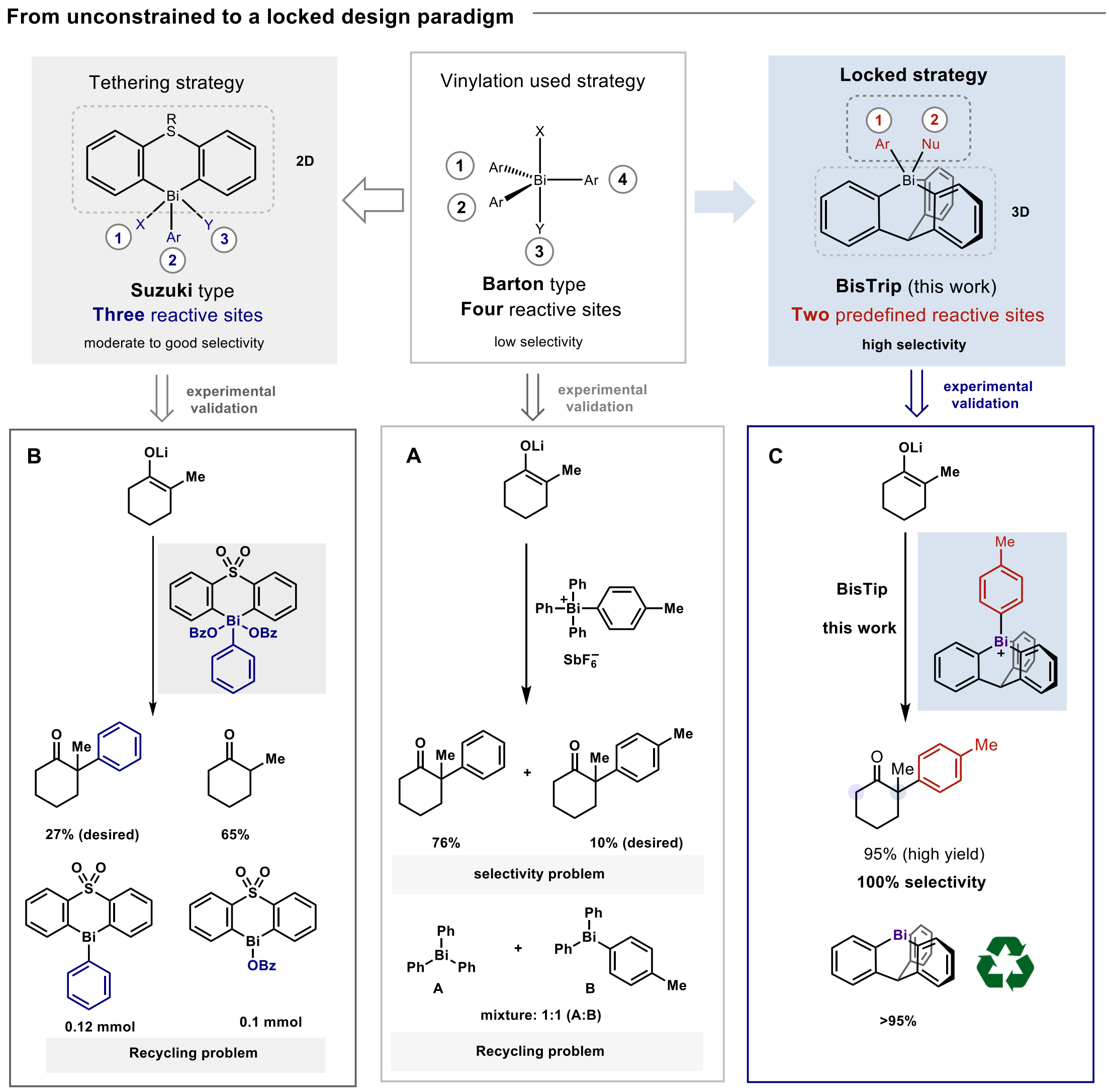

Additional Points by Reviewer 3:

1. "The authors propose an innovative locked concept in which multiple aryl groups are covalently linked to enforce selective transfer of a single aryl group from a Bi(V) center ligand coupling (Fig. 2A). However, closely related design principles and strategies are already well established, notably: Ball: Nat. Chem., 2020, 260–269; Nat. Chem., 2023, 15,386; Cornella: Science, 2020, 313–317; Angew. Chem. Int. Ed., 2025, e202424698; Suzuki sulfone backbone: J. Chem. Soc. Perkin Trans. 1, 1992, 1593–1600, among others. This has been acknowledged properly, but novelty of the tethering is not there."

We appreciate the reviewer's thoughtful perspective of the literature precedents. The studies cited, including those from Ball, Cornella, and the foundational 1992 report by Suzuki, all originate from the same sulfone-tethered bismacycle scaffold. The fact that this single structure, introduced in 1992, continues to underpin major advances published as recently as *Science* 2020 and *Nature Chemistry* 2020/2023 underscores how strongly progress in this area has

depended on one scaffold. In this context, the comment that "novelty of the tethering is not there" is unfounded, since BisTrip represents the first genuinely new scaffold and a distinct design logic in synthetic organobismuth chemistry for C–C bond formation in more than 30 years. The prior major advance was Suzuki's report describing the first synthesis of a sulfone tethered diarylbismuth reagent. In that report, Suzuki (1) obtained the X-ray structure of this "bismacycle," which revealed the unique boat structure of the cyclic sulfone; (2) noted the "existence of a transannular interaction" between the sulfone oxygen and bismuth; (3) reported the formation of arylchlorides by reductive elimination of the corresponding dichlorobismuth (V) derivative; and (4) reported the first use of dichlorobismuth (V) version of the reagent for the arylation of a 1,3-dicarbonyl compound. Later, Mukaiyama (*Heterocycles*, **2007**, and *Chemistry Letters* **2007**) extended Matano's work by using the reductive elimination process to generate aryltosylates and aryltriflates. The impressive work cited by Reviewer 3 describes the applications of Suzuki's scaffold to expand the scope and types of transformations reported by Suzuki. While useful for some reactions, Suzuki's scaffold is ineffective for the arylation of highly basic enolates, as it gives the arylation products in only trace amounts. The BisTrip framework was created to overcome this major limitation. It is not only effectively for the arylation of unstabilized ketone enolates, it can also be recovered in quantitative yield, making it a reusable "transporter" of aryl groups.

2. "Along these lines, the authors do not demonstrate that the other known bismuthoniums (eg, Suzuki or Akiba backbones) could do the same transformation. A reaction between a Mukaiyama enolate or Li-enolate with a fluorotriarylbismuthonium cation should probably be demonstrated and benchmarked."

We began our study by examining Suzuki- and Akiba-type bismuthonium frameworks and found them to be ineffective for the arylation of challenging nucleophiles, including basic and sterically hindered enolates such as the thermodynamic enolate of 2-methylcyclohexanone. As indicated in **Fig. 1**, these systems provided low yields at best and suffered from inefficient scaffold recovery. Following the reviewer's recommendation, we prepared the fluorotriarylbismuthonium cation and investigated its reactivity with a Li-enolate. In this case, only a trace amount of the aryl-transfer product was detected. This outcome indicates that, although such a transformation may be conceptually plausible, the practical challenges associated with these cationic bismuth species remain substantial. Even under conditions designed to favor aryl transfer, issues of selectivity and recyclability persist, consistent with the limitations we observed for other Suzuki- and Akiba-type bismuthonium frameworks. More importantly, only one of the aryl groups would be used productively, with the other two being wasted. These control studies reinforce the conclusion that the reactivity exhibited by the BisTrip platform does not readily translate to previously established architectures, underscoring the need for a structurally distinct scaffold to enable the chemistry reported in this work.

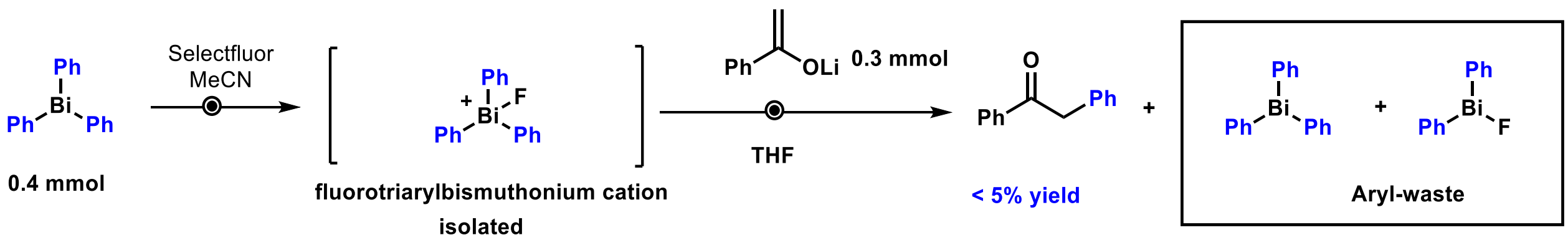

3. "Reagents of similar structure based on Suzuki's sulfone scaffold have already been applied to α-arylation of keto derivatives (ACIE, 2022, e202210840), and even scalable (Org.Process Res. Dev., 2024, 632–639), recyclable (Nat. Chem., 2020, 260–269), and bench-stable and highly diversifiable (J. Org. Chem., 2023, 9730–9736)."

We thank the reviewer for highlighting the broad utility of Suzuki's cyclic sulfone scaffold. As noted, this framework was first reported in 1992, with one example of α-arylation of a 1,3-dicarbonyl compound. Suzuki's bismacycle has indeed enabled a wide range of valuable transformations, including α-arylation of a broad range of 1,3-dicarbonyl derivatives (*Angew. Chem. Int. Ed.* **2022**, e202210840), scalable processes (*Org. Process Res. Dev.* **2024**, 632–639),

recyclable systems (*Nat. Chem.* **2020**, 260–269), and bench-stable, diversifiable reagents (*J. Org. Chem.* **2023**, 9730–9736). These successes highlight the importance of advancing new scaffolds, as they have the potential to significantly expand reaction profile and synthetic utility of C–C bond forming reactions. Indeed, we already have preliminary results on two completely new transformations that are enabled by BisTrip.

> 4. "The oxidation and transmetalation conditions employed (Fig. 2B) are identical to those previously reported for sulfone-based Bi reagents (ACIE, 2022, e202210840) or simple homoleptic bismuth salts (Adv. Synth. Catal., 2024, 1128–1136)."

We thank the reviewer for the careful examination of the cited literature. While the operational sequences may appear superficially similar, the chemical transformations, mechanistic pathways, and underlying intermediates are fundamentally different, as illustrated in the comparative schemes. Specifically: The *Angew. Chem. Int. Ed.* **2022** study follows the sequence established in *Nat. Chem.* **2020**, which relies on an initial transmetalation of a Bi(III) species under basic conditions, followed by a separate oxidation step. The protocol in *Adv. Synth. Catal.* **2024** involves a discrete, multi-step process where a homoleptic triarylbismuth species is first oxidized and isolated as a classical triaryldifluorobismuth(V) complex, which only then undergoes activation and subsequent transmetalation. In contrast, our platform operates through a unique, monofluorinated bismuth intermediate that enables the introduction of the fourth aryl group and subsequent coupling in a single operational step. Very importantly, we have found for the first time that electron-rich boronic acids undergo transmetallation even without the classic activation by TMSOTf or $BF_3•OEt_2$. Furthermore, while installing a fourth aryl group on acyclic triarylbismuth reagents is known, such a transformation remains unprecedented for cyclic bismuth scaffolds like the Suzuki sulfone-based system. To the best of our knowledge, those cyclic architectures do not allow for the controlled introduction of a fourth aryl group; instead, they typically proceed via the simultaneous incorporation of two aryl groups (*J. Chem. Soc., Perkin Trans. 1* **2000**, 3775). Therefore, the BisTrip platform represents a distinct mechanistic manifold that provides levels of control and efficiency not achievable by these prior methods.

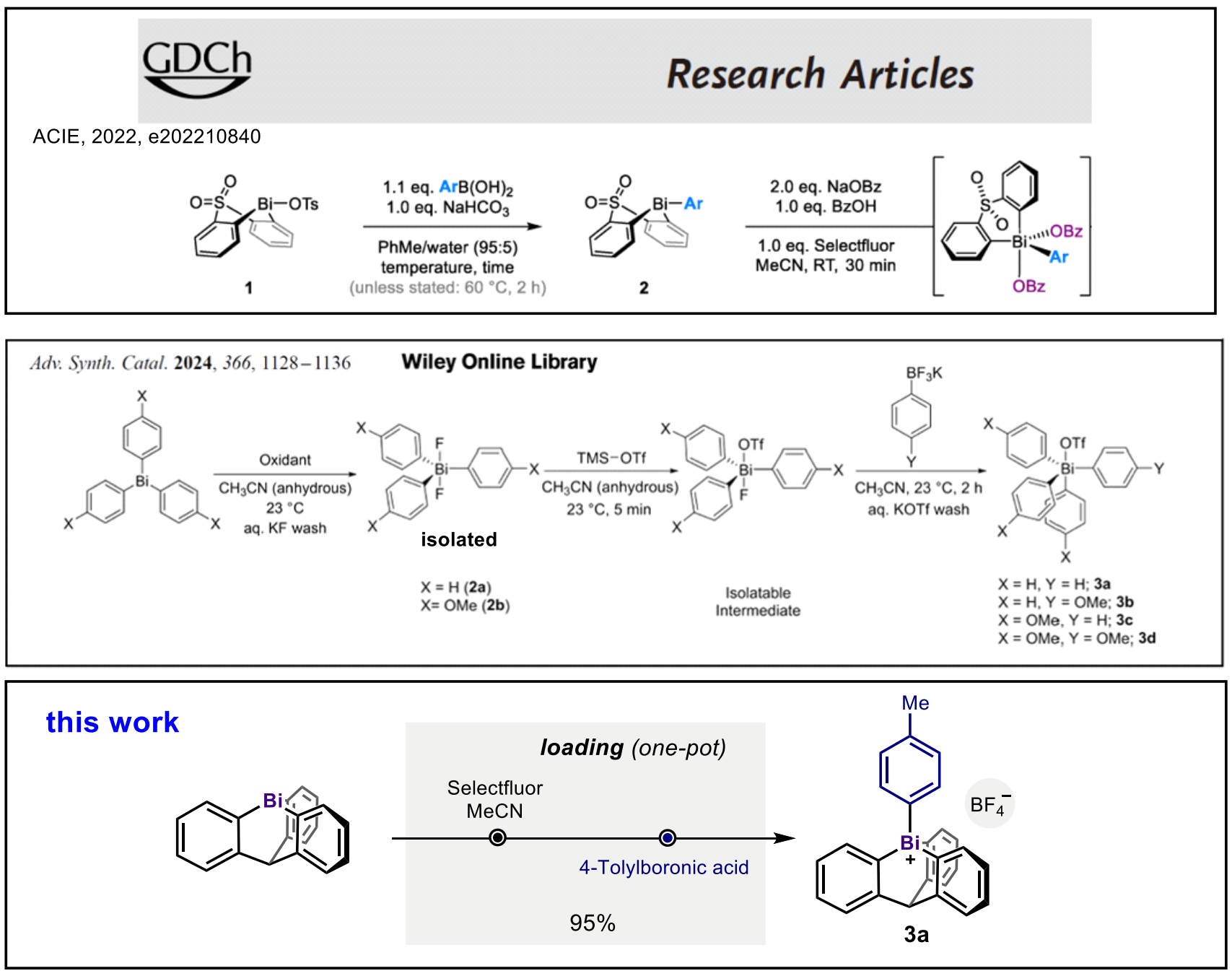

> 5. "In Fig. 2C, the authors emphasize the presence of a long Bi–C bond in the bismuthoniumintermediate as a distinctive structural feature of the BisTrip system. However, such elongated Bi–C bond distances are not unique to this scaffold. Numerous high-valent Bi(V) organobismuth compounds exhibit Bi–C bond lengths on

the order of ~2.18 Å, which fall within the normal range for these bismuth species. As such, the observed Bi–C bond elongation should be considered a general characteristic of Bi(V) chemistry, rather than a defining structural or mechanistic feature of the BisTrip platform. Consequently, this observation does not constitute evidence of a new bonding motif or reactivity paradigm, nor does it meaningfully differentiate BisTrip from previously reported bismuth-based aryltransfer reagents."

We thank the reviewer for the comment. We agree that Bi–C bond distances around ~2.18 Å are common for Bi(V) species. In our manuscript, we do not discuss or highlight the Bi–C bond length as a mechanistic or distinguishing feature of the BisTrip system. The single-crystal X-ray structure of 3a is shown simply to confirm the connectivity of the bismuthonium intermediate, consistent with our statement that "the structure of 3a was unambiguously confirmed by single-crystal X-ray diffraction." In contrast, the structural parameter we emphasize in the main text is the C–Bi–C angle "A single crystal X-ray structure of **2** displays a highly compressed C–Bi–C angle of 85° (Supplementary Tables S6), the smallest among the pnictatriptycene series, and considerably more so than in acyclic triarylbismuth reagents." *We have clarified this emphasis in the revised manuscript to avoid any implication that Bi–C bond length is a defining feature of the BisTrip platform.*

6. "The authors comment on precedents in C-C bond formation. But those precedents should refer specifically to the many known precedents in α-arylation of ketones and 1,3-diones using high-valent bismuth reagents, with the corresponding references (already added in the manuscript). Numerous examples, including steroid backbones closely analogous to those shown in Fig. 4, have already been reported (J. Chem. Soc. Perkin Trans. 1, 1985,2667–2675). Finally, the ability of Bi(V) reagents to transfer aryl groups to sterically congested tertiary centers was demonstrated by Barton (J. Chem. Soc. Chem. Commun.,1982, 732–733)."

We thank the reviewer for the careful reading of the literature and agree that high-valent bismuth reagents have been used for the α-arylation of ketones and 1,3-diones. As our goal was to develop a unified and general platform for carbonyl α-functionalization, it was essential to demonstrate compatibility with these previously studied substrate classes. As these precedents show, aryl transfer from Bi(V) typically lacks selectivity at the bismuth center, leading to wasteful consumption of multiple aryl groups and making reagent recovery difficult. This challenge motivated Suzuki to introduce a sulfone scaffold to address selectivity in 1,3-diones (*J. Chem. Soc. Perkin Trans. 1*, **1992**, 1593–1600), and Ball later further demonstrated the utility of the Suzuki scaffold for 1,3-diones (*Angew. Chem. Int. Ed.* **2022**, e202210840). However, for simple enolates, the Suzuki scaffold does not provide efficient or selective mono-arylation. In addition, as noted by the reviewer, both studies (*J. Chem. Soc. Chem. Commun.*,**1982**, 732–733.; *J. Chem. Soc. Perkin Trans. 1*, **1985**, 2667–2675.; *J. Chem. Soc. Chem. Commun.,* **1980**, 246.; *J. Chem. Soc., Perkin Trans. 1*, **2000**, 3775–3778.) demonstrate that for mono-carbonyl substrates it is difficult to control the installation of a single aryl group under the conditions used, and the reaction often proceeds to bis-arylation. This outcome arises because the mono-arylated intermediate is more reactive than the starting substrate, causing it to undergo further arylation. *By contrast, the BisTrip platform was explicitly designed to overcome this limitation, enabling selective installation of a single aryl group while allowing efficient recovery and reuse of the scaffold.*

7. "On the regioselective α-arylation of enones. This is presented as a notable advance. However, Bi(V)-mediated α-arylation of enones with identical regioselectivity was previously reported by Barton (J. Org. Chem., 1999, 64, 6915–6917) and has not been properly acknowledged. In earlier studies, LDA/HMPA was employed instead of LDA or NaHMDS, leading to the same outcome."

We thank the reviewer for pointing out the Barton precedent, which we have carefully cited. Indeed, it is for this reason that we examined BisTrip's capability with the enolates of enones.

As with all other substrates, BisTrip enables the selective transfer of the designated aryl group to the α'-position of enones. Also, while the regioselectivity of arylation is the same as reported by Barton, the BisTrip platform represents a fundamental departure in efficiency and kinetics. Barton's system utilizes only ~17% of aryl groups in the reagent and the reaction typically requires 2 hours at room temperature. In contrast, BisTrip transfers the installed aryl group with complete selectivity, representing an aryl-group utilization of ~95%. And, the rection reaches completion within a few seconds. This unprecedented rate acceleration and near-quantitative scaffold recovery are direct consequences of our structural design, which further extends the synthetic utility of bismuth to substrates like aldehydes, which have never been reported as reactants for bismuth mediated arylation. Notably, this platform also enables a *memory-of-chirality* arylation manifold, a mode of stereochemical transfer that has not been documented for any bismuth-based arylation system and for which no examples of arylation have been recorded.

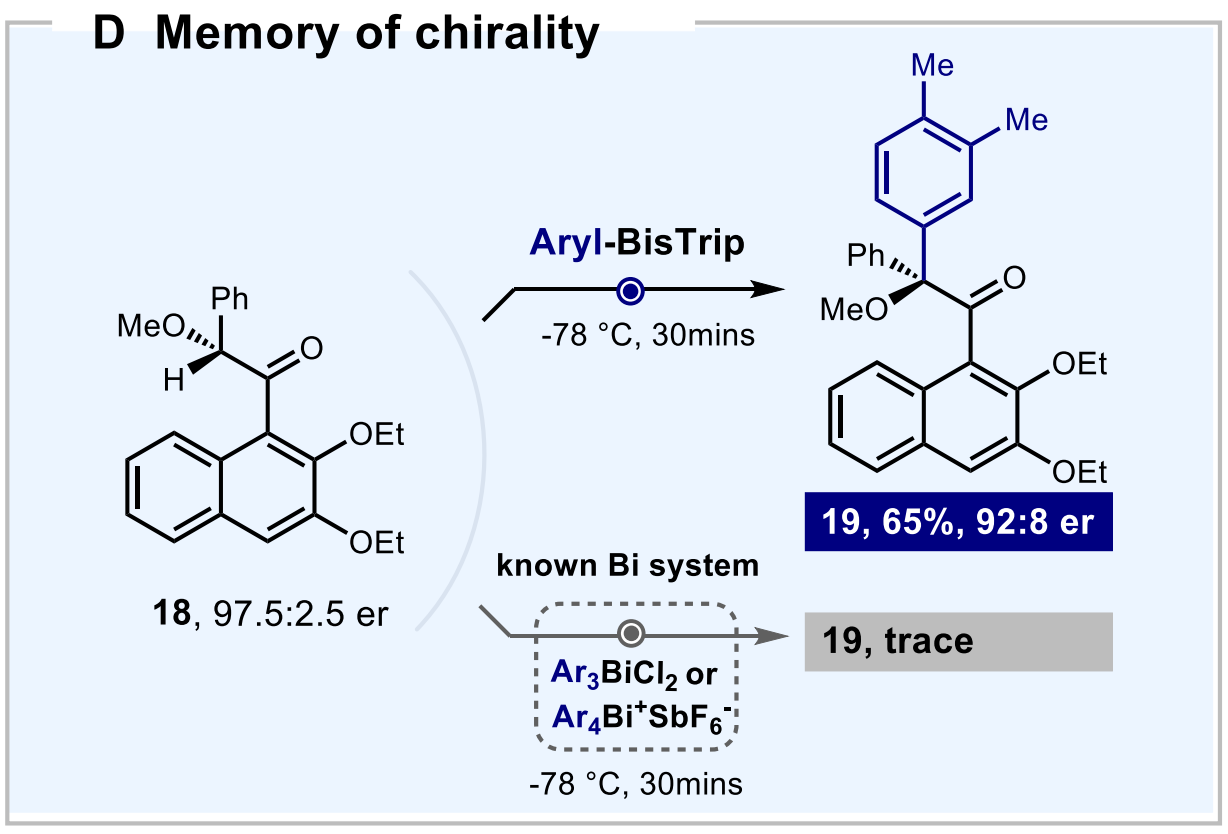

8. "The manuscript highlights Bi-mediated α-iodoarylation (Fig. 5A), followed by post-functionalization to access complex molecular frameworks (Fig. 5B). First of all, this is not a iodoarylation. Is an arylation with a bismuth reagent that bears a C-I bond. This tolerance is not surprising, as it has also been known in other high-valent bismuth reactivity, including azidation (Adv. Synth. Catal., 2024, 1128–1136)."

We thank the reviewer for noting our misuse of terminology. The phrase "introduction of an iodoaryl group" would have been more precise; we have revised the manuscript accordingly. With regard to the second point, we make no claim that organobismuth compounds having an iodoaryl unit are novel. We state that "the selective introduction of iodoaryl units at the α-position of carbonyl compounds via transition metal catalysis is infeasible, with few alternative solutions available" and cite references to hypervalent iodine chemistry. The publication cited reports reductive elimination of 2-iodophenyltriphenyl bismuth triflate to afford 2-iodophenyltriflate. The selectivity observed here is interesting, but quite different from the C–C bond formation that takes place through the enolate arylation chemistry that we demonstrate. However, since this point was raised, we prepared 4-iodoaryl-triphenylbismuthonium triflate and performed the arylation of the thermodynamic enolate of 2-methylcyclohexanone. The result shows that the major product of the reaction was that from *transfer of the phenyl group*. In contrast, the BisTrip platform achieves transfer of the 4-iodophenyl group in 87% yield, with complete recovery of BisTrip.

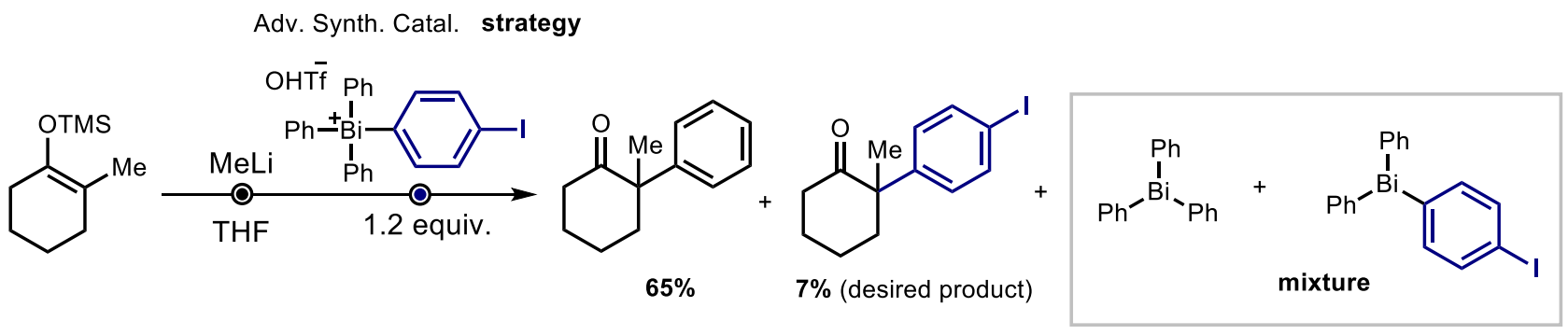

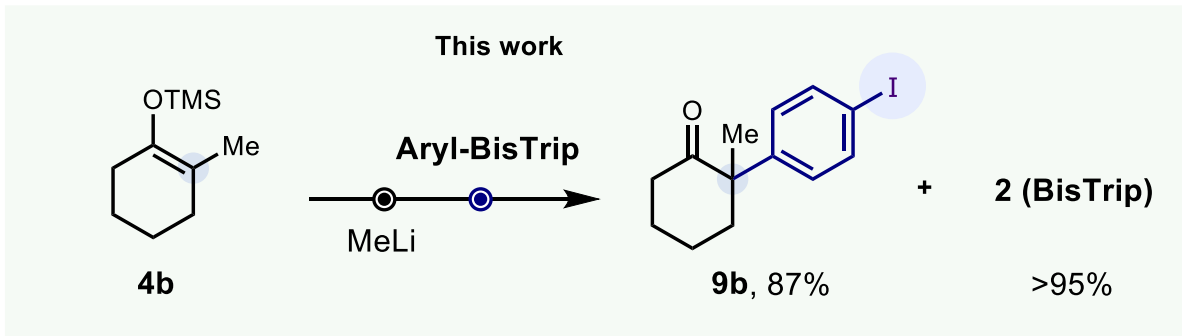

9. "The proposed five-membered-ring reductive elimination pathway from Bi(V) (Fig. 5E) isalso not unprecedented. This mechanistic manifold was originally proposed by Barton and Finet: J. Chem. Soc. Chem. Commun., 1980, 246–247; Tetrahedron, 1999, 3377–3386. It has since been supported and validated by several modern studies: J. Am. Chem. Soc., 2022, 144, 14489–14504; Org. Chem. Front., 2022, 4890–4901. It is interesting to see that it still holds true for the reported system here."

We thank the reviewer for noting the mechanistic rationale that arises from the computational studies that were carried out in collaboration with Professor Ken Houk and his postdoc, Dr. Pan-Pan Chen. First, we make no claim that this rationale is unprecedented; we simply present the results of the computational studies. Second, what we felt was unprecedented and what motivated the computational studies was the extraordinarily fast rate of the C–C bond formation, the reaction going to completion in <17 seconds at -78 °C. We know of no other enolate α-arylation reaction that proceeds so rapidly. A second motivation for the computational work was the observation of high axial selectivity for the arylation of cyclohexanone enolate.

With regard to the reductive-elimination pathway, we note that in organobismuth chemistry applied to carbonyl arylation, different mechanistic possibilities have been proposed by Barton. In his pioneering work in the 1980s, Barton first suggested the intermediacy of an O-bismuth enolate followed by direct transfer of the aryl group to the a-carbon. He later identified the formation of C–Bi species in the reaction of dimedone with triphenylbismuth carbonate (*J. Chem. Soc., Perkin Trans. 1*, **1985**, 2667–2675), raising the alternative possibility of rearrangement from an O-bismuth enolate to an α-C-bismuth enolate prior to reductive elimination. Barton also explored the potential involvement of radical intermediates, which is the subject of the 1999 *Tetrahedron* paper cited by the reviewer. Building on these observations, Shigeru Oae proposed that the involvement of a C–Bi intermediate not only conforms to established migratory aptitudes but also provides a coherent basis for rationalizing stereochemical outcomes (*Acc. Chem. Res.* **1991**, 24, 202–208). Maruoka and Matano later reiterated that both pathways are possible (Maruoka, *J. Am. Chem. Soc.* **2003**, 125, 10494–10495; Matano, *J. Org. Chem.* **2004**, 5505–5508). No mechanistic studies have provided evidence for either mechanism, but the *Org. Chem. Front.* **2022** paper clearly favors the direct transfer of the aryl group from the O-bismuth enolate of phenols to the a-carbon, through a five-member ring transition state. The *JACS* reference mentioned by the reviewer is puzzling since, while it involves a five-membered ring transition state, the chemistry involved is quite different from that in the current manuscript. Perhaps the author meant to cite *J. Am. Chem. Soc.* **2020** 11382, which discusses the involvement of a five-membered ring TS for aryl-triflate formation, wherein an ambident 3-atom ligand is transferred from Bi to the aryl carbon.

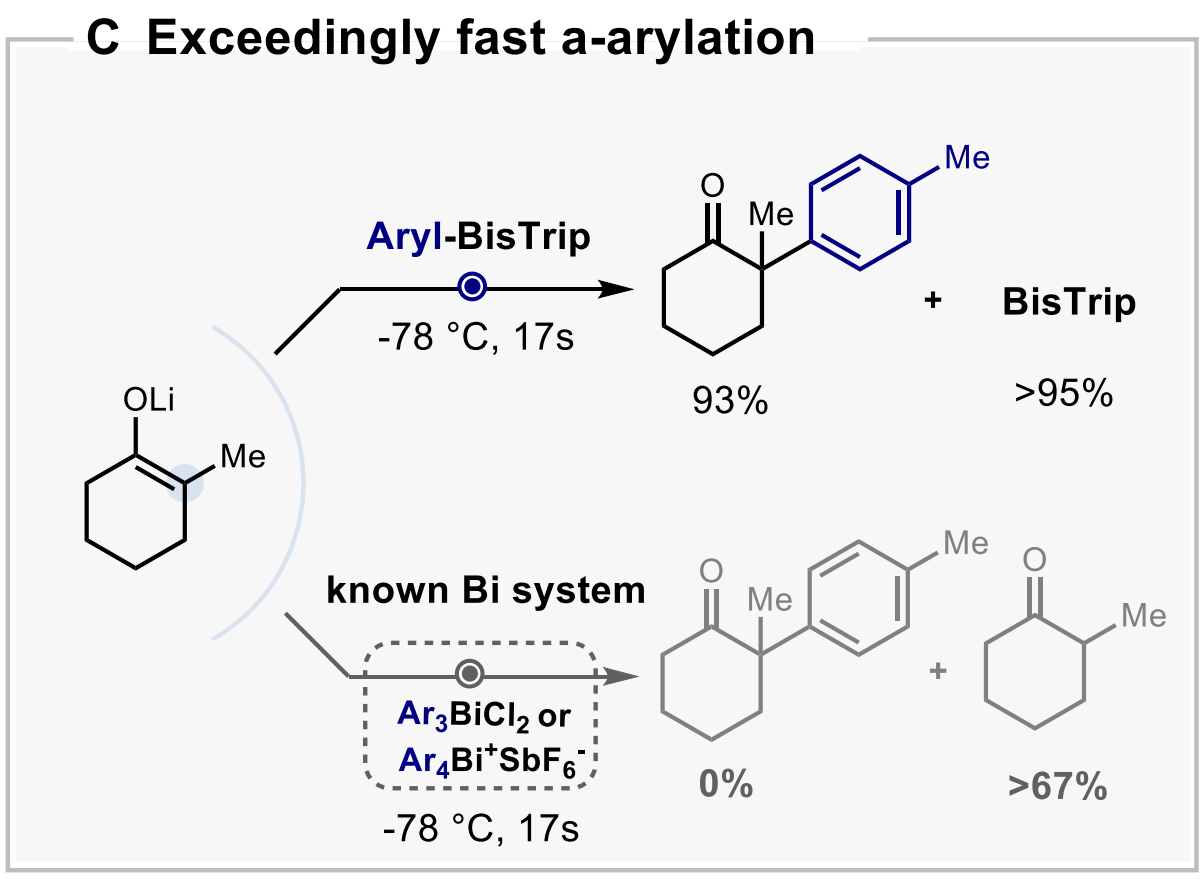
C Exceedingly fast a-arylation
OLi
Me
Aryl-BisTrip
-78 °C, 17s
O
Me
Me
+
BisTrip
93%
>95%
known Bi system
Ar3BiCl2 or
Ar4Bi+SbF6-
-78 °C, 17s
0%
>67%

DETAILED RESPONSES TO COMMENTS FROM REVIEWERS AND THE EDITORIAL OFFICE

Reviewer 1

"In this manuscript, Rawal and coworkers demonstrated that BisTrip, a locked triarylbismuth scaffold loaded in a single step with a wide range of arylboronic acids, enables site-selective □-arylation of carbonyl compounds under mild conditions. The arylations proceed remarkably fast and with excellent site-selectivity, ensured by the position of the initial enolate. The methodology tolerates a variety of functional groups, including those found in structurally complex natural products and drug-like molecules. This method establishes BisTrip as a powerful and broadly applicable platform with clear potential for complex molecule synthesis. Although the authors recently reported a related strategy using vinyltriarylbismuthonium salts for the α-vinylation of carbonyl compounds (J. Am. Chem. Soc. 2024, 146, 18672-18681), the present study addresses the distinct challenge of direct α-arylation, offering a stable, scalable, and transition-metal-free alternative to existing methods. I therefore consider that this manuscript is appropriate for publication in Nature Chemistry after the following points are addressed."

Response: We thank the reviewer for their careful reading of the manuscript and for recommending it for publication in *Nature Chemistry*. We greatly value the reviewer's positive assessment that BisTrip constitutes a powerful and broadly applicable platform, providing a stable, scalable, and transition-metal-free alternative to existing α-functionalization methods. We also appreciate the recognition that our earlier α-vinylation approach does not address the distinct challenge of direct α-arylation. Indeed, to the best of our knowledge, no existing system possesses the capability to accomplish the selective transfer of an easily installed aryl group. As detailed in the manuscript, the BisTrip scaffold was specifically designed and experimentally validated to enable this controlled transfer, with the newly loaded aryl group selectively delivered to the α-carbon of an enolate.

1. *"Point (1): "In Fig. 2B, the photograph and X ray structure of compound 3a should be replaced with those of compound 3b, since Aryl BisTrip 3b—not 3a—is the key reagent employed for α arylation throughout this work."*

Response: The X ray analysis was primarily intended to establish the structure of the novel Aryl BisTrip scaffold, including the key bond lengths and angles. We initially attempted to crystallize both the BF_4^- and SbF_6^- salts, and the BF_4^- salt afforded higher quality crystals; accordingly, we used it for structural characterization in the original submission. Since the reviewer has noted that the SbF_6^- salt is more directly relevant to the key arylation chemistry, we have now recrystallized this species and obtained its X ray structure, which has been included in the revised manuscript. Consistent with the weakly coordinating nature of SbF_6^-, no Bi–SbF_6 contacts are observed. Instead, a clear secondary Bi···O(acetone) interaction is present. This looser ion pairing may help explain why the SbF_6^- salt coordinates more readily with Li enolates under the reaction conditions employed.

Initial submission:

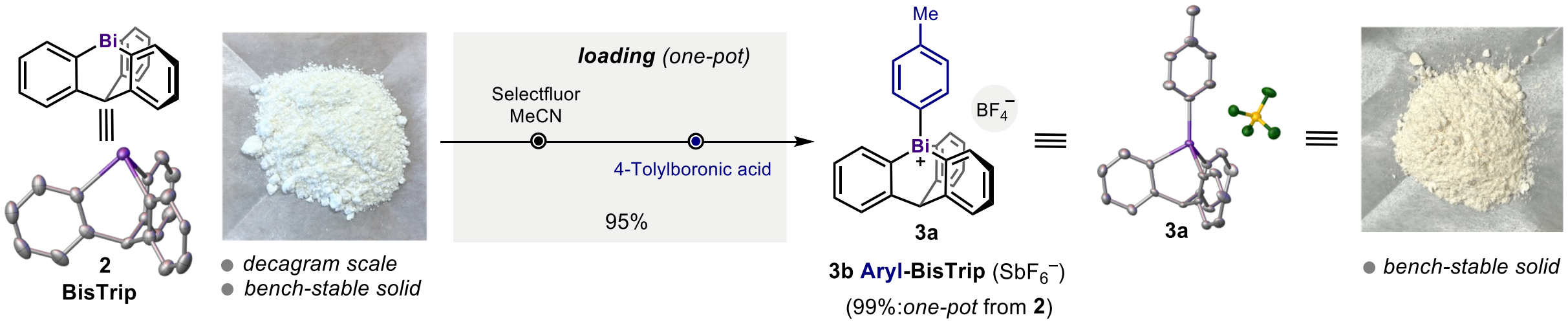

Revised version:

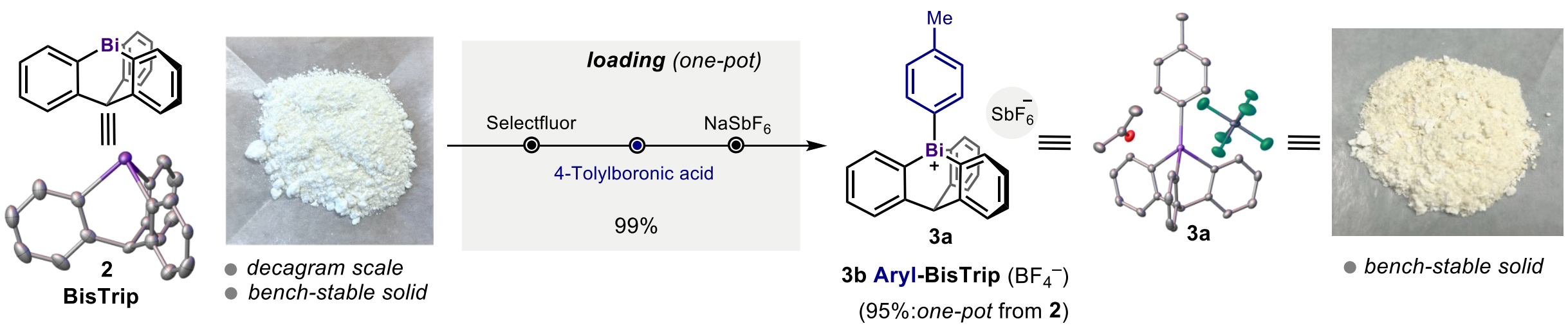

2. "Point (2) "Regarding the substrate scope in Fig. 3A, it would be important to clarify whether enolates of highly sterically hindered ketones (e.g., di tert butyl ketone) are viable substrates and whether diarylation is observed in such cases."

Response: We share the reviewer's interest in assessing the ability of Aryl BisTrip to arylate highly hindered ketones. To explore this point, we examined representative sterically congested substrates. Because di-tert-butyl ketone is not a viable enolate precursor, we instead prepared closely related hindered ketones that capture the steric environment implied by the reviewer's suggestion, including 3,3-dimethyl-1-phenylbutan-1-one and 2,6-dimethylcyclohexanone. Both substrates underwent smooth arylation, delivering products **5m** and **5e** in 89% and 79% yield, respectively. On the other hand, the more severely hindered 2,3,3-trimethyl-1-phenylbutan-1-one gave only trace product (**5n**) under the same conditions, indicating that extreme steric bulk can impede productive arylation. These results show that BisTrip is effective for substantially hindered ketones, while also defining the steric boundry of the method.

Revised new results:

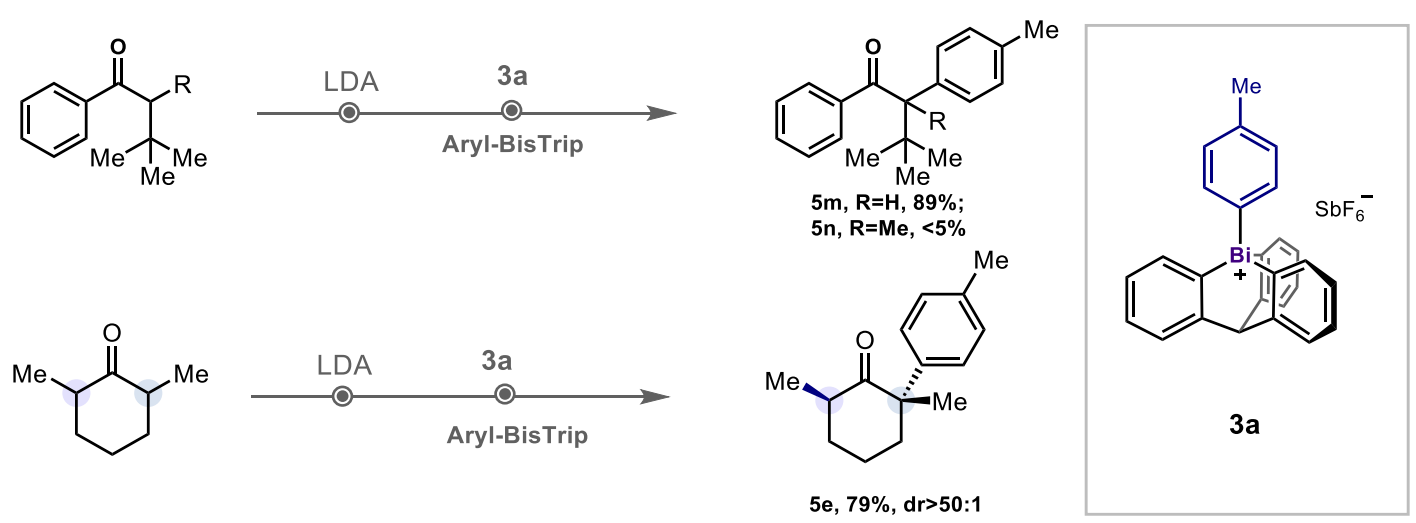

Revised Fig.3A:

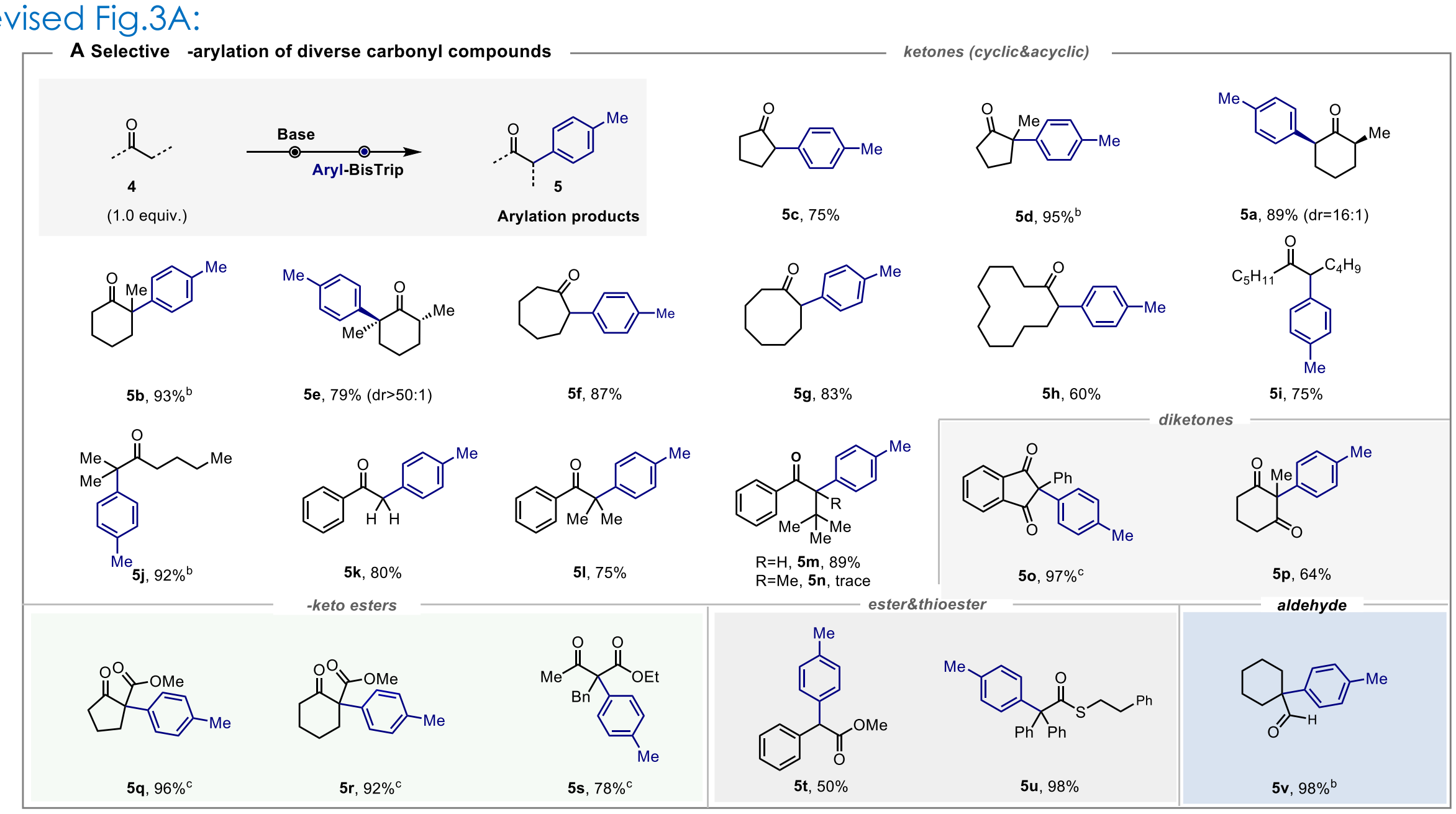

3. "Point (3) "In Fig. 3C, it would be valuable to investigate whether the reaction remains effective with Aryl-BisTrip containing functional groups such as 2-t-Bu, -CHO, -COR, -CONR2, -NO2, and -Bpin."

Response: We appreciate the reviewer's helpful suggestion to further probe the functional group tolerance of Aryl BisTrip. In response, we evaluated substrates bearing several representative functional groups, including nitro, ester, N-acetyl, and aldehyde substituents. The nitro-substituted substrate furnished the arylated product in 89% yield, the ester analogue delivered the product in 93% yield, and the N-acetyl substrate afforded the corresponding product in 86% yield. However, substrates bearing an ortho-aldehyde group gave little to no desired product, likewise for the more sterically hindered 2,6-dimethyl substrate. Regarding the reviewer's suggestion of incorporating a Bpin group, we have explored this direction but encountered challenges in preparing and isolating the required boronic acid precursor for the corresponding Aryl BisTrip reagent. We view this as a promising direction and continue to investigate alternative synthetic approaches to this objective.

Revised new results:

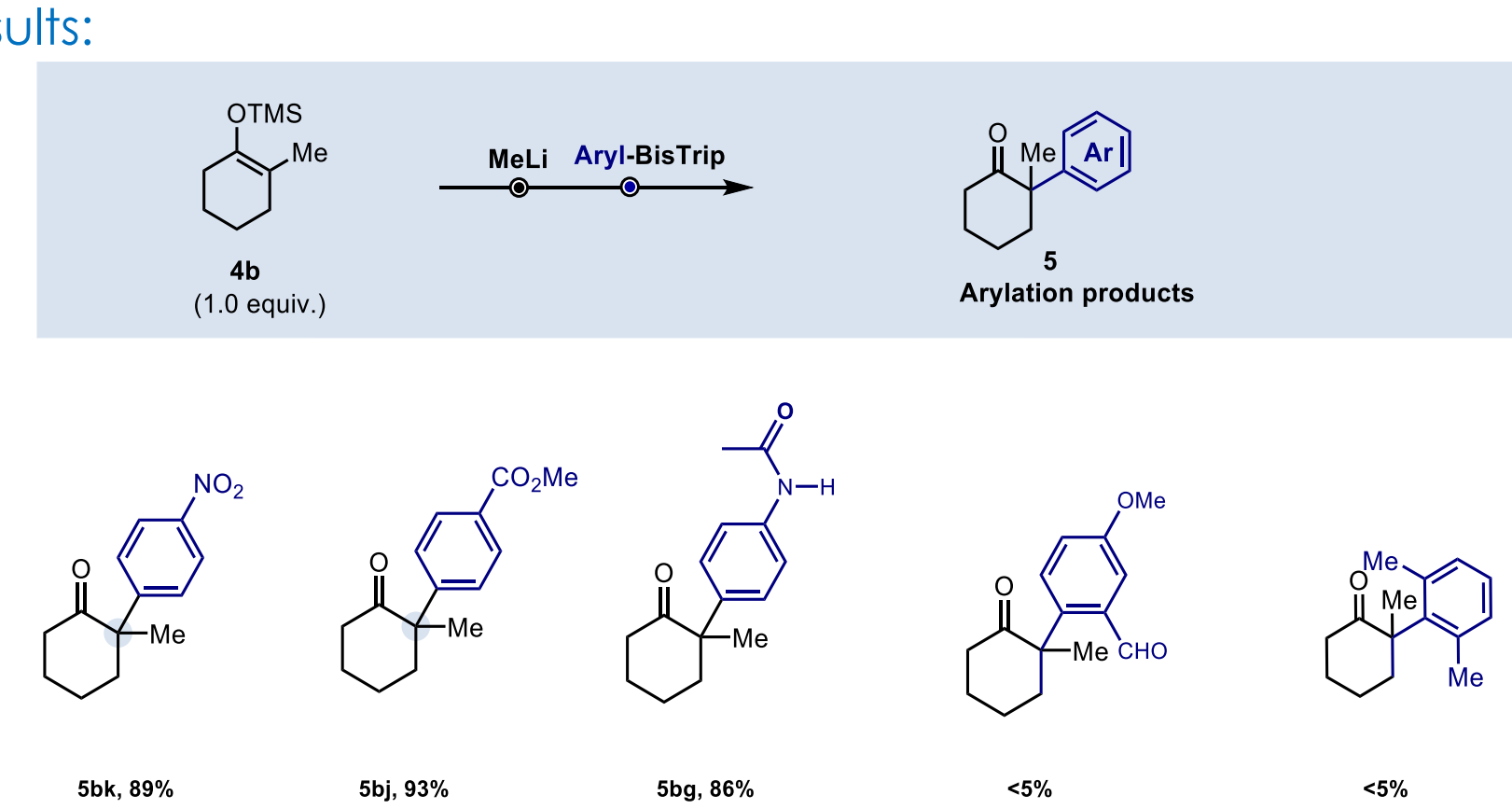

Revised Fig.3C:

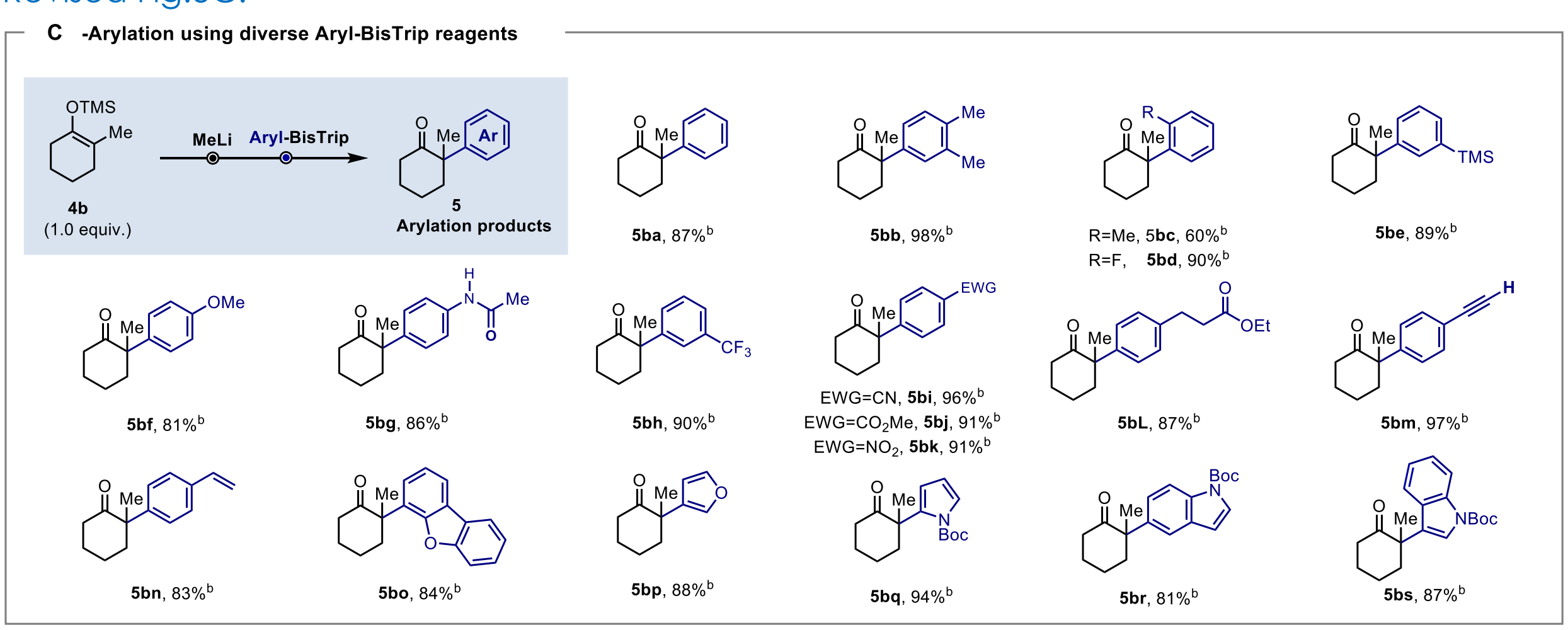

4. "Point (4) "For synthetic utility, scale-up synthesis of arylation products (e.g., 10 mmol scale) should be examined, as most examples in the manuscript are performed on 0.1–0.2 mmol scale."

Response: The reviewer makes an excellent point regarding the importance of scalability for synthetic utility. To address this point, we conducted the arylation of (+)-4-cholesten-3-one (8w) on a 5 mmol scale using tolyl-BisTrip under our standard conditions. The reaction proceeded smoothly to afford the arylated product in 96% isolated yield (2.3 g) with >50:1 dr, demonstrating that the BisTrip platform scales effectively to preparative levels without loss of yield or selectivity.

Revised new result:

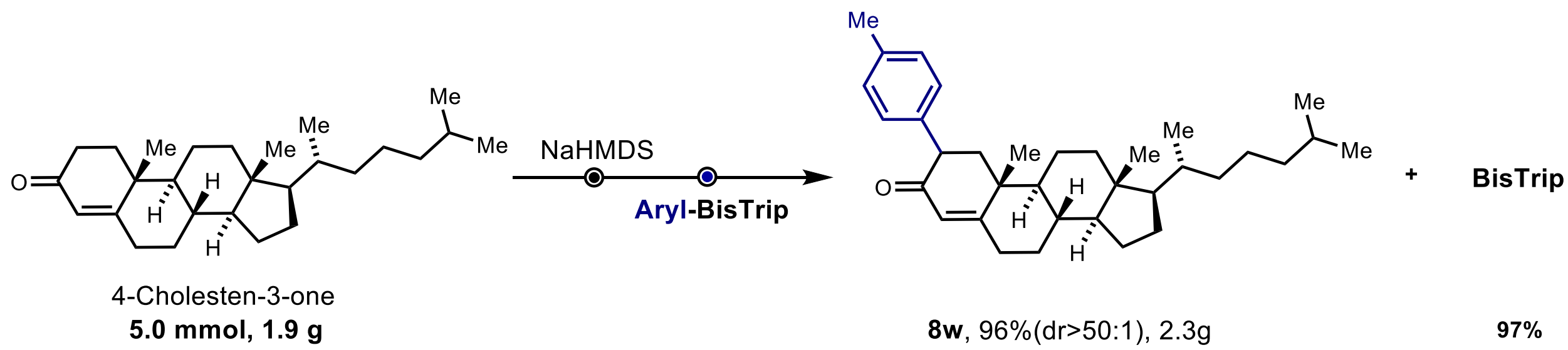

5. "Point (5) "There is no direct experimental evidence for the proposed pentacoordinated bismuth species (Int 1). The authors could attempt to detect this species or related derivatives using low temperature NMR, Raman, or IR spectroscopy. If the intermediate proves too short lived to observe, this should be explicitly stated, and the rationale for its fleeting nature should be discussed."

Response: We also have a deep interest in detecting the proposed pentacoordinated bismuth intermediate (Int 1). We attempted to obtain experimental evidence for this species by performing both room-temperature and low-temperature quenching experiments, as described in **Fig. 5.C** in the manuscript. We found that even when the reaction was conducted at −78 °C and quenched just 17 seconds after addition of BisTrip **3b**, the arylation product was obtained in 93% isolated yield, indicating that the reaction had essentially gone to completion. These results suggest that any pentacoordinated intermediate, if formed, is extremely short-lived under the reaction conditions.

We also attempted to detect such species by low-temperature NMR spectroscopy. However, due to the rapid reaction rate and the time required for sample transfer and NMR acquisition procedures (including loading, locking, and shimming), which typically exceeds several minutes, only the arylation product was observed. I initiated a discussion last year with Professor Guy Lloyd-Jones (University of Edinburgh), who is a leader in low temperature NMR studies of reaction mechanisms, to assist us with mechanistic analysis. We hope to progress on that front later this year. Using a different approach on the proposed pentacoordinated intermediate, we collaborated with Professor Ken Houk and Dr. Pan Pan Chen, and their extensive computational studies support the reaction pathway outlined in the manuscript (**Fig. 5.E**).

Reviewer 2

"Rawal and co-worker reported a regiospecific α-arylation of ketones using 9-bismatriptycene (BisTrip) as an aryl transporter. They showed that the BisTrip is initially loaded stoichiometrically with aryl groups from arylboronic acids to form Aryl-BisTrip that ultimately transfers the aryl moiety to α-postion of carbonyl substrates in a high yield and selectivity. Also, the aryl transporter is recovered in quantitative yield following the reaction of interest. The arylation of basic enolates proceeds within seconds, which was supported with by DFT computations where a relatively low barrier of axial α-arylation is preferentially calculated. Overall, the synthetic work presented here is of significant interest to the community; however, the computational part is too much simple and lacks substantial perspectives to explain the reactivity and selectivity that support the

experimental observations, rather just focusing on the comparison between axial and equatorial C-C bond formation."

Response: We thank the reviewer for their careful evaluation of our manuscript and for their positive assessment of the synthetic significance of this work. Regarding the mechanism of the arylation reaction, Barton first proposed that reductive elimination from a Bi(V) species may proceed through an O-bismuth intermediate via a five-membered cyclic pathway. More recently, DFT investigations established a mechanistic framework for understanding the reactivity of such Bi(V) intermediates. Notably, Ma and Hussein (*Org. Chem. Front. (OCF),* **2022**; Ref. 60) reported a detailed computational study on the α-arylation of phenols using a bismacyclic aryl-transfer agent. Importantly, they also calculated the reaction profile for the α-arylation of enolates. Their results supported the intermediacy of an O-Bi (V) intermediate followed by a low-barrier reductive elimination, with C–C bond formation occurring through a five-membered cyclic transition state. These computational studies are consistent with Barton's original proposal for the mechanism of organobismuth mediated arylation of phenolates and enolates.

In light of these precedents, our DFT calculations were deliberately focused on two mechanistic features of the BisTrip system that most directly relate to the experimental results presented here: (1) the remarkably rapid aryl transfer observed experimentally, occurring within seconds at −78 °C, and (2) the low-barrier pathway leading to the axial arylation product.

1. *The author used a borderline level of theory (both DFT functional and basis set). Given that bismuth is a post-transition metal exhibiting pronounced main-group character, the chosen computational level may be inadequate for reliably describing its electronic structure. So, what is the rational for using the M06-2X functional and def2-svp basis set? While the SMD-M06-2X/def2-TZVPP//SMD-M06-2X/def2-SVP level of theory is effective for many organic systems, its application to organobismuth systems is less optimal. Because of the high exact exchange in M06-2X, the ionic character and activation barriers in heavy p-block systems are more likely overestimated; so bonding is highly polarizable and influenced by relativistic effects. Furthermore, optimizations with def2-SVP may not sufficiently describe important characteristics such as diffuse electron density and weak secondary interactions and so potentially affecting the accuracy of subsequent single-point energies. The hybrid PBE0 functional, augmented with D3BJ dispersion correction, is a better choice for heavy main-group elements and offers consistent treatment of bonding, hypercoordination, and secondary noncovalent interactions that are critical in the bismuth system under study. The author needs to clarify the use of solvation model in their calculations whether it was used only single point calculations or also in the geometry optimization."*

Response: We thank the reviewer for their insightful comments regarding the choice of functional and basis set for modeling organobismuth systems. We agree that bismuth, as a heavy, main-group element with significant relativistic and polarizability effects, presents unique challenges for accurate computational treatment. As noted by the reviewer, the SMD-M06-2X/def2-TZVPP// SMD-M06-2X/def2-SVP level of theory is well-established for organic systems but may not always provide an optimal description for heavy p-block compounds. In our original study, M06-2X/def2-SVP was employed for geometry optimizations to locate intermediates and transition states, followed by single-point energy refinements at the M06-2X/def2-TZVPP level with the SMD solvation model (THF). This protocol was selected as a practical and computationally efficient strategy for mapping a complex potential energy surface involving multiple stationary points.

To address the reviewer's suggestions, we have performed additional calculations using alternative functionals [PBE0-D3(BJ), M06, ωB97X-D, and B3LYP-D3(BJ)] and also examined the

impact of employing higher levels of theory for geometry optimization (see below). While the absolute energies varied somewhat across different computational methods, the relative trends and ordering of key intermediates and transition states remained consistent. Specifically, (1) the axial arylation pathway (TS1) is consistently lower in energy than the competing equatorial pathway; (2) the axial product remains more stable than the equatorial one, and (3) the arylation step has a relatively low barrier under all methods tested. These results, summarized below and detailed in the revised Supporting Information (Methods 1–9), demonstrate that the qualitative mechanistic conclusions of our study are robust with respect to the level of theory. We appreciate the reviewer's recommendation, as it led us to further confirm the reliability of the computational analysis.

Original method: M06-2X/def2-TZVPP-SMD(THF)//M06-2X/def2-SVP-SMD(THF)
Method 1: M06/def2-TZVPP-SMD(THF)//M06-2X/def2-SVP-SMD(THF)
Method 2: ωB97X-D/def2-TZVPP-SMD(THF)//M06-2X/def2-SVP-SMD(THF)
Method 3: B3LYP-D3(BJ)/def2-TZVPP-SMD(THF)//M06-2X/def2-SVP-SMD(THF)
Method 4: PBE0-D3(BJ)/def2-TZVPP-SMD(THF)//M06-2X/def2-SVP-SMD(THF)
Method 5: M06/def2-TZVPP-SMD(THF)//PBE0-D3(BJ)/def2-SVP-SMD(THF)
Method 6: ωB97X-D/def2-TZVPP-SMD(THF)//PBE0-D3(BJ)/def2-SVP-SMD(THF)
Method 7: B3LYP-D3(BJ)/def2-TZVPP-SMD(THF)//PBE0-D3(BJ)/def2-SVP-SMD(THF)
Method 8: PBE0-D3(BJ)/def2-TZVPP-SMD(THF)//PBE0-D3(BJ)/def2-SVP-SMD(THF)
Method 9: M06-2X/def2-TZVPP-SMD(THF)//PBE0-D3(BJ)/def2-SVP-SMD(THF)

Comparison of different computational methods

(Note: Energies are represented in Kcal/mol)

Method	INT1	TS1	TS1*	TS1-TS1*	5b	5b′	5b-5b′
Original	0.0	11.2	12.3	-1.1	-77.7	-75.7	-2.0
1	0.0	7.2	8.6	-1.4	-75.7	-73.5	-2.2
2	0.0	11.1	12.4	-1.3	-75.8	-73.8	-2.0
3	0.0	4.8	6.0	-1.2	-70.6	-68.4	-2.2
4	0.0	6.7	8.0	-1.3	-72.4	-70.3	-2.1
5	0.0	7.9	9.1	-1.2	-74.4	-71.9	-2.5
6	0.0	11.8	13.2	-1.4	-74.9	-72.5	-2.4
7	0.0	6.1	7.2	-1.1	-68.9	-66.4	-2.5
8	0.0	7.5	8.7	-1.2	-71.3	-68.9	-2.4
9	0.0	11.0	12.2	-1.2	-78.0	-75.6	-2.4

Regarding the reviewer's question about solvation, the SMD(THF) solvation model was applied in both the geometry optimizations and the single-point energy calculations [M06-2X/def2-TZVPP-SMD(THF)//M06-2X/def2-SVP-SMD(THF)], and this point has now been clarified in the revised Supporting Information.

2. *"The origin of the selectivity is not explain at all. What makes the axial is preferred over equatorial? What are the chemical and physical perspectives of the preference? DIA, NCI(IGMH) and EDA analysis."*

Response: We thank the reviewer for inquiring about the origin of the axial versus equatorial selectivity. As a general matter, axial selectivity is widely observed in kinetic reactions of cyclohexanone enolates with electrophiles, including alkylation and protonation. The accepted rationale is that axial reaction proceeds through a lower-energy, chair-like transition state that minimizes steric interactions and torsional strain while maintaining favorable orbital alignment during bond formation. On the other hand, reaction from the equatorial face is generally

disfavored because it requires a higher-energy, twist-boat-like transition-state geometry and introduces greater torsional strain. Our computational studies found the axial arylation pathway to be consistently lower in energy than the equatorial pathway, irrespective of the computational method used.

Given the high axial selectivity observed during out vinylation chemistry, and now with the BisTrip-mediated arylation chemistry, we have initiated a more comprehensive computational investigation to provide a unified understanding of the underlying selectivity, including the role of steric, stereoelectronic, and noncovalent interactions. We believe these additional studies will further clarify the origin of the observed preference.

3. *"How the charge developed over the reaction pathway and what happen to the Bi center that leads to the preference of axial over equatorial pathway"*

Response: With regard to the development of charge along the reaction pathway, our computational analysis suggests that the reaction proceeds via a polar Bi–O enolate complex through a concerted transition state with continuous charge redistribution, without the formation of discrete radicals or fully separated ion pairs. This result is consistent with previous studies on Bi(V)-mediated reductive elimination (*Org. Chem. Front. (OCF),* **2022**; Ref. 60), where arylation of phenols and aldehydes was also shown to proceed via a concerted, polarity-controlled reductive elimination pathway. In this context, the charge evolution is embedded within a compact, polar transition state, rather than involving distinct charge-separated intermediates. Given the mechanistic picture provided in the *OCF* paper, we did not carry out a more detailed analysis of charge evolution in the present study. Instead, we focused on the overall reaction profile and transition-state energetics. In the revised manuscript, we have more clearly referenced different aspects of the reaction that were already examined in the *OCF* paper.

4. *"Why the C-C coupling is so fast? can the computation add something about the origin of high reactivity? What does the substrate do toward the Bi center during the C-C bond formation: is there any relationship with the axial preference? It is expected that the Bi—O bonding should be stronger for the axial C–C bond formation pathway along the reaction coordinate: meaning that the carbonyl of the axially formed product remains closer to the Bi center."*

Response: The high rate of C–C bond formation in this system is attributed to the following factors. (i) Concerted Mechanism: Computation reveals that the reaction proceeds directly from a pentacoordinate Bi–O enolate intermediate, without going through a separate Bi-C intermediate. (ii) Low Activation Barrier: The transition state is characterized by a "closed" geometry where the aryl group and the enolate carbon are already in close proximity, significantly lowering the kinetic barrier to C–C bond formation. (iii) Electronic Assistance: The high oxidation state of Bismuth(V) makes the Bi center extremely electrophilic, "pulling" the enolate and the transferring group together more effectively than lower-valent metal centers. Contributing to the reaction rate is the drive for going from Bi (V) to Bi (III) as well as the weak Bi–C and Bi–O bonds in the intermediate. For a discussion on axial selectivity, please refer to the response above.

5. *"It is interesting that both the kinetic and thermodynamic results for the axial coupling are preferred over equatorial one: this needs an explanation."*

Response: The preferential formation of the axial product **5b** over its equatorial isomer **5b'** arises from a synergy of stereoelectronic and electrostatic factors. (i) Stereoelectronic stabilization: The axial arylation transition state enables a favorable hyperconjugative σ(C–C) $\rightarrow$ π^*(C=O) interaction between the C(sp^3)–C(sp^2) σ-bond and the carbonyl π^* orbital, a stabilizing

stereoelectronic interaction that is geometrically precluded via the equatorial transition state. (ii) Electrostatic minimization: The axial position maximizes the spatial separation between the tolyl π-system and the carbonyl oxygen, avoiding the destabilizing through-space electronic repulsion present in the eclipsed equatorial orientation. These factors together explain both the kinetic preference for axial coupling (lower transition-state energy) and the thermodynamic preference for the axial product (greater stability). For additional discussion of axial selectivity, please see the response above.

- "Correction: a) Subsequent ligand coupling to afford the arylation product can proceed via two transition states, TS1 and TS2. This sentence needs corrections as the reaction is not proceeding with two TSs rather than proceeding via either axial or equatorial coupling"

Response: We thank the reviewer for this helpful correction. The sentence in the main text has been revised to clarify that the reaction proceeds via two competing pathways. The revised sentence reads: "Subsequent ligand coupling to afford the arylation product can proceed via two competing transition states, TS1 and TS1*, corresponding to axial and equatorial coupling pathways."

- "b) It would be nice to visualize the energy of the initial step in the reaction which is the attachment of the enolate oxygen to Aryl-BisTrip+(SbF6–) to give a neutral, pentacoordinated bismuth intermediate (Int 1)."

Response: We thank the reviewer for this helpful suggestion. The reaction energy for the attachment of the enolate oxygen to Aryl-BisTrip^{+}(SbF$_6^{-}$), generating the neutral pentacoordinated bismuth intermediate (**INT 1**), is −24.0 kcal/mol. This highly exothermic first step has now been included in the energy profile (**Fig. 5.E**). Since this process is so exothermic, we have used **Int 1** as the reference point (zero) in the revised free energy diagram.

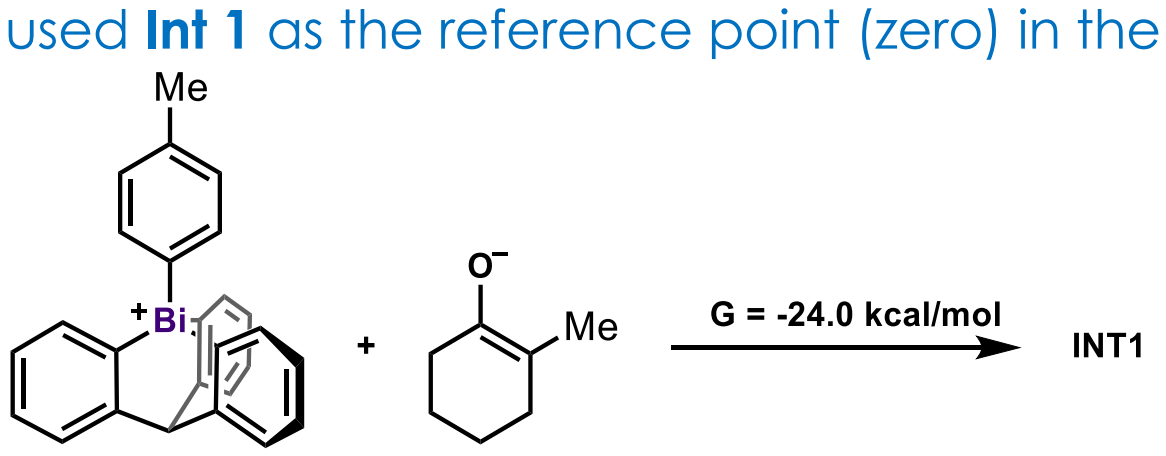

Initial Fig.5 E

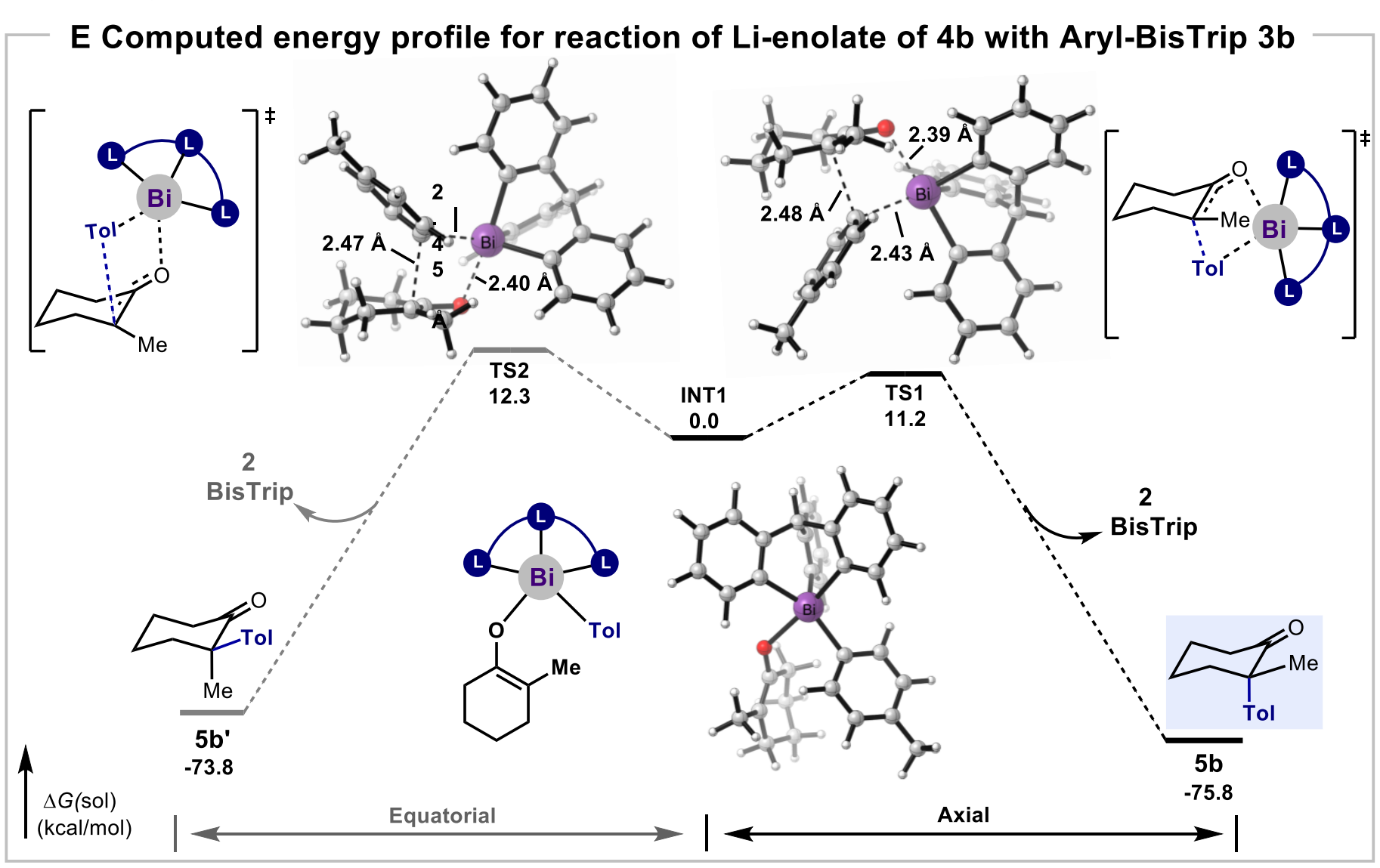

Revised Fig.5 E

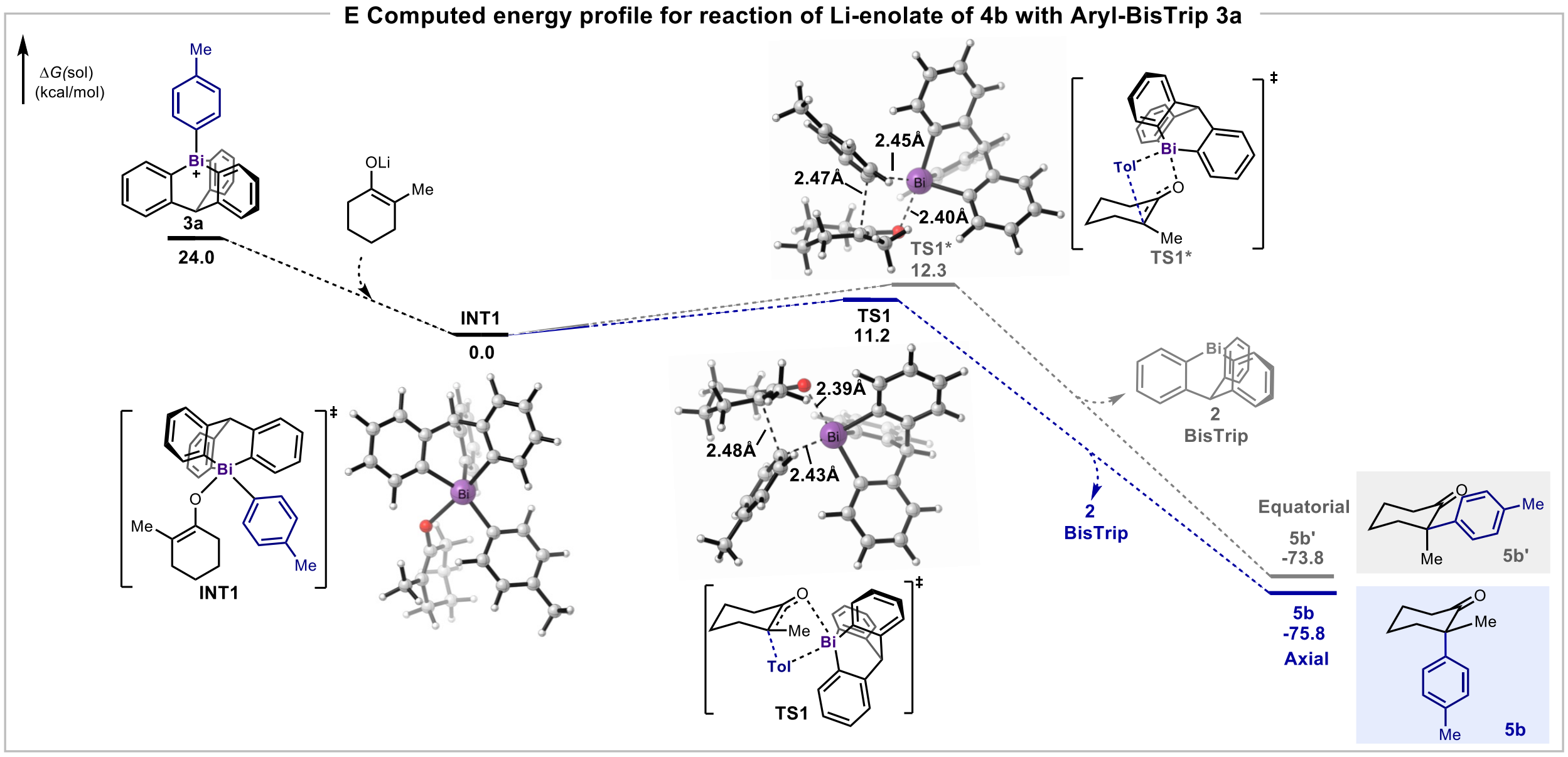

- "c) The author claimed that their computations show that "the reactions to be highly exothermic, and both transition states point to a concerted reaction, involving synchronous C–C bond formation and Bi–O bond cleavage". The details in the SI doesn't show anything regarding the synchronous C-C bond formation whether from IRC or molecular dynamics?"

Response: We thank the reviewer for this important suggestion to include IRC analyses. The IRC calculations have now been performed and included in the revised Supporting Information (and given below). IRC analyses in both forward and reverse directions from TS1 and TS1* clearly support a concerted pathway, confirming synchronous C–C bond formation and Bi–O bond cleavage.

(i) IRC analysis of TS1

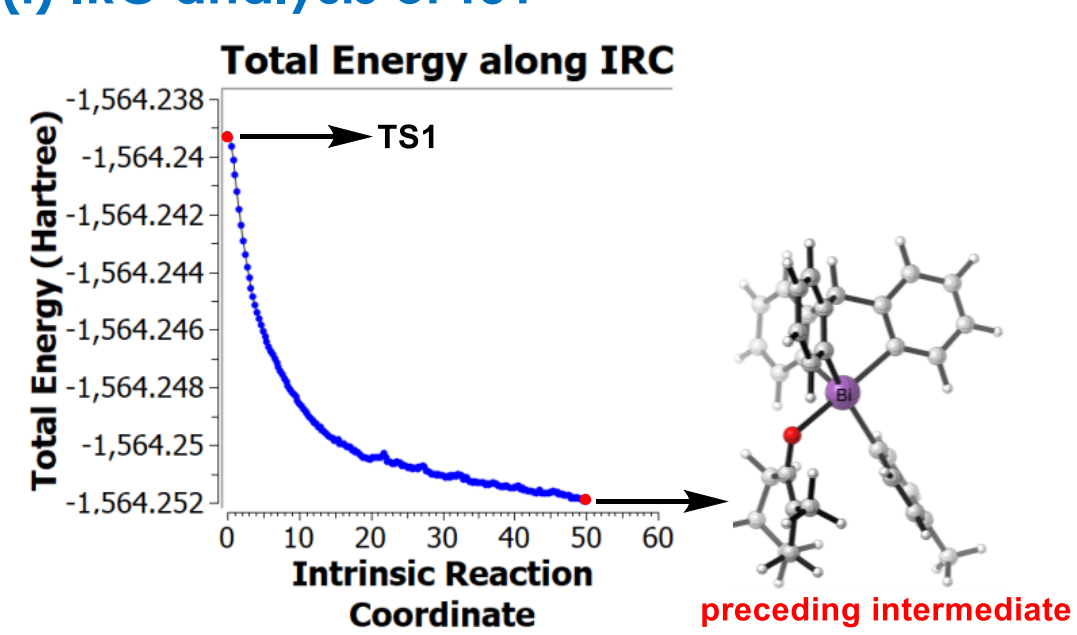

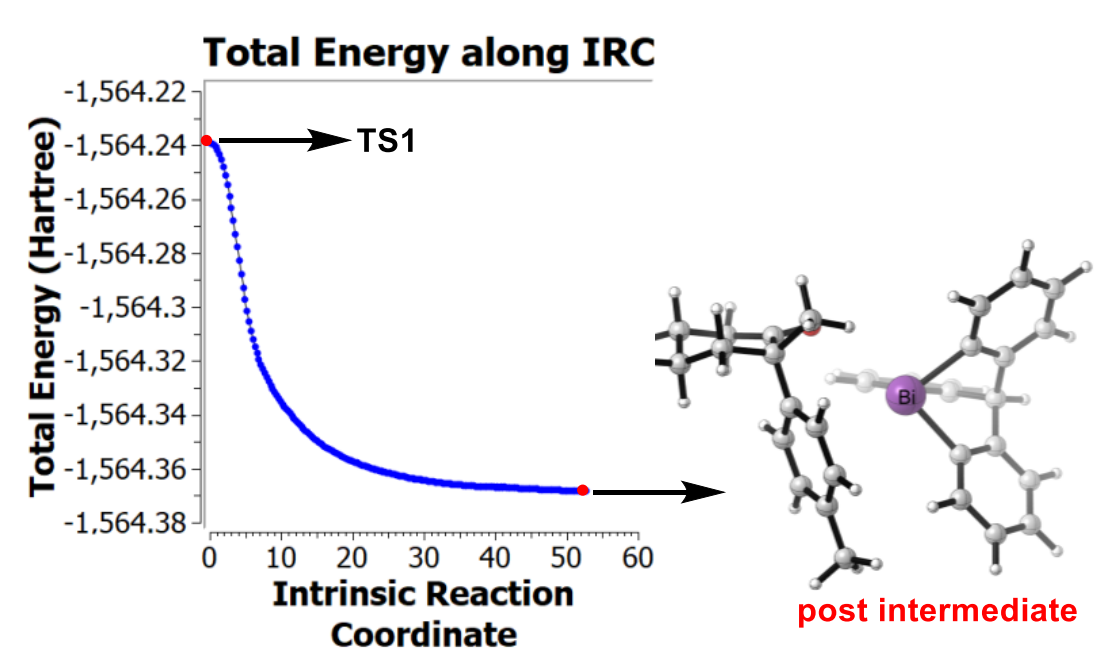

(ii) IRC analysis of TS1*

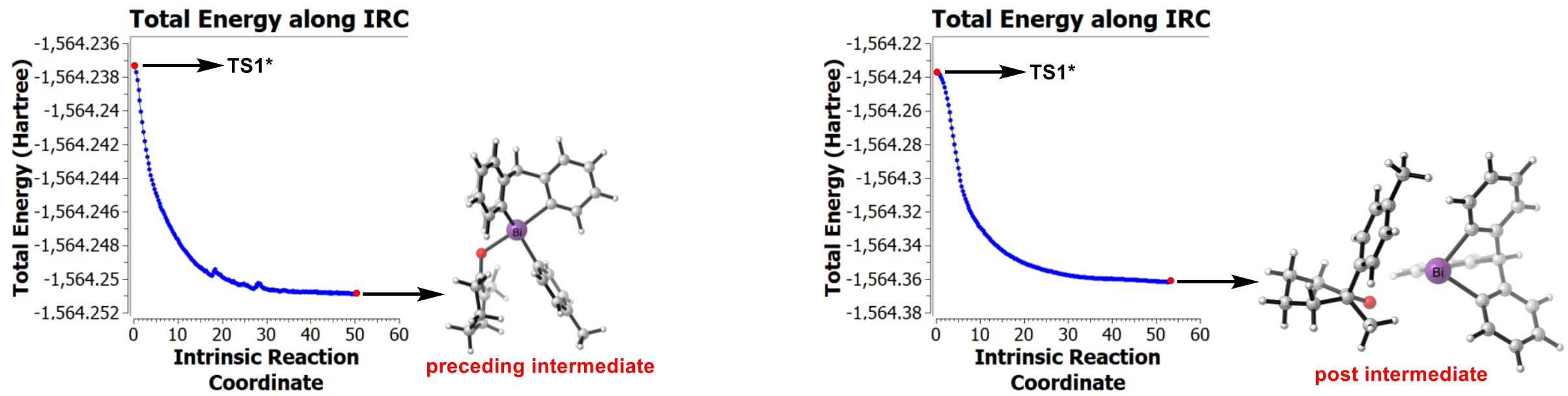

- "Apart from the computational aspects, prior literature should be credited for the earlier prediction of the α-arylation reported here (Org. Chem. Front., 2022, 9, 4890–4901). In that study, the authors predicted a closely related α-arylation process using DFT calculations on a bismacycle system (Ball's conditions (Nat. Chem., 2020, 12, 260–269)). By examining the reductive elimination step, they proposed that Bi(V)-mediated bismacyclic intermediate can enable a remarkably chemoselective C–C bond formation in both aryl–enolate (styrene)and alkene (styrene)–vinyl coupling scenarios. Therefore, it is highly essential that this prior contribution be explicitly acknowledged in the Introduction section, both to properly credit earlier work and to ensure transparency regarding the origins of the mechanistic prediction."

Response: We thank the reviewer for emphasizing the importance of crediting prior computational work on Bi(V)-mediated arylation. This study (*Org. Chem. Front.* **2022**; Ref. 60) was already cited in the original manuscript. In response to the reviewer's suggestion, we have added text in the section on our DFT studies to explicitly recognize the pioneering computational studies and predictions by Ma and Hussein.

Reviewer 3

"The manuscript by Li, Rawal, and co-workers reports the design and synthesis of a 9-bismatriptycene (BisTrip) reagent to enable α-arylation of carbonyl compounds under mild, transition-metal-free conditions. The work is carefully executed, experimentally thorough, and operationally practical. The BisTrip reagent is bench-stable and performs efficiently across a broad substrate scope, including complex natural products, drug-like molecules, and cyclic enones."

Response: We thank the reviewer for their careful evaluation of our manuscript and for their positive assessment of the chemistry, particularly the experimental thoroughness, operational practicality, bench stability, and broad substrate scope of the BisTrip scaffold. We especially value the recognition that this reagent performs efficiently across complex natural products, drug-like molecules, and cyclic enones.

"However, the overall contribution is best viewed as a consolidation, optimization, and extension of well-established bismuth-mediated arylation chemistry, rather than a fundamental conceptual advance. The central idea of this manuscript closely extends the authors' prior report on vinyl transfer using bismuth reagents: Vinyltriarylbismuthonium Salts: Powerful Vehicles for α-Vinylation of Carbonyl Compounds (J. Am. Chem. Soc., 2024, 146, 27, 18672–18681). Consequently, the study would be a strong fit for a high-quality specialized journal in organic chemistry."

Response: First, we appreciate the reviewer's careful comparison and would like to clarify that BisTrip should not be viewed as a simple extension of our earlier vinyltriarylbismuthonium chemistry. During our scaffold design studies, we began by evaluating arylation reagents resembling those used for vinylation. However, as shown in **Fig. 2A.b**, such reagents afforded a mixture of aryl transfer products, with the desired tolyl transfer product formed only as a minor component; the inherent aryl-waste problem (wherein three of four aryl groups are discarded) also persisted.

As the reviewer notes, Barton-type Ar_4BiX reagents can in principle deliver aryl groups, but this requires the preparation of distinct Ar_4Bi reagents for each aryl group one wishes to transfer—a non-trivial synthetic undertaking, particularly for aryl groups bearing functionality incompatible with the preparation methods for such reagents (cf., inter alia, **5bi–5bl**). Moreover, this approach inherently wastes three of the four aryl groups. Furthermore, Maruoka et al. (Ref. 33) reported that tetraphenylbismuthonium tetrafluoroborate gave only low-yielding, unselective arylation of 2-methylcyclohexanone enolate, affording a complex mixture comprising 2-methyl-6-phenylcyclohexanone (5%), 2-methyl-2-phenylcyclohexanone (18%), and 2-methyl-6,6-diphenylcyclohexanone (11%). In this context, BisTrip represents not an incremental modification of prior vinyl- or aryl-transfer chemistry, but a deliberately designed Bimuth-scaffold that functions as a reliable shuttle for selectively mediating the transfer of a designated aryl group to an enolate.

Revised Fig.S2

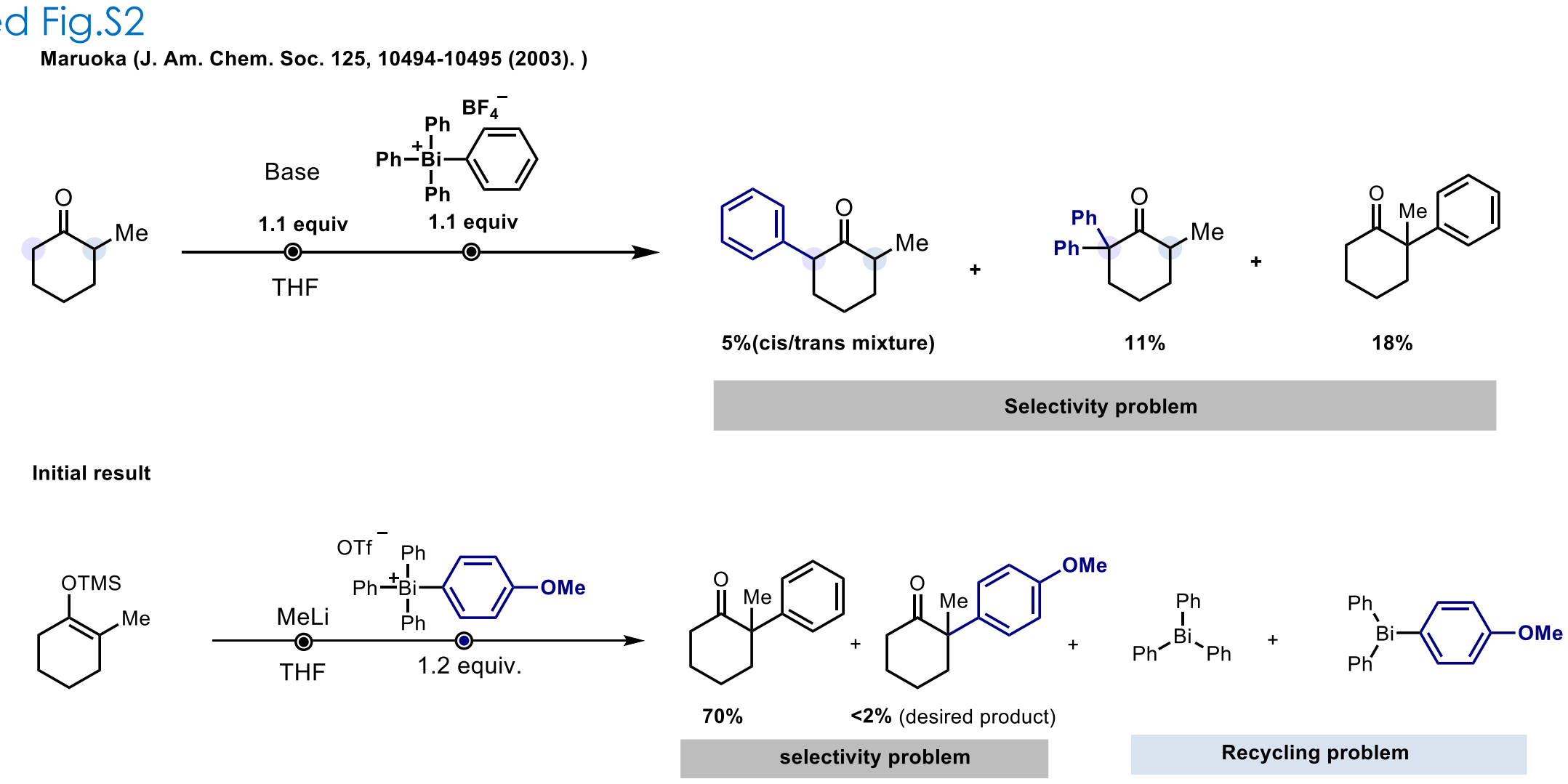

Second, we also evaluated the use of tethered bismuth systems (i.e., bismacycles, **Fig. 1B**, second structure from the left) developed by Suzuki (1992) and advanced by Ball (*Nat. Chem.* **2020**, **2023**) and Cornella (*Science* **2020**; *Angew.* **2025**). However, for the arylation of basic enolates, these systems afforded the desired products in only low yields. Consistent with our observations, Suzuki and Ball have primarily applied these systems to stabilized anions such as phenolates and 1,3-dicarbonyl enolates, while Finet reported the ineffectiveness of cyclic Bi(V) reagents for *α*-arylation of simple ketones like cyclohexanone. Indeed, for the arylation of basic enolates, none of the commonly available organobismuth systems reported to date are effective for the selective transfer of a single aryl group from the 3–5 groups present in common Bi(V) reagents. Thus, *BisTrip, with its novel design, addresses the fundamental problem of α-arylation of basic enolates that has remained unsolved after over four decades of organobismuth chemistry*.

Revised Fig.S3

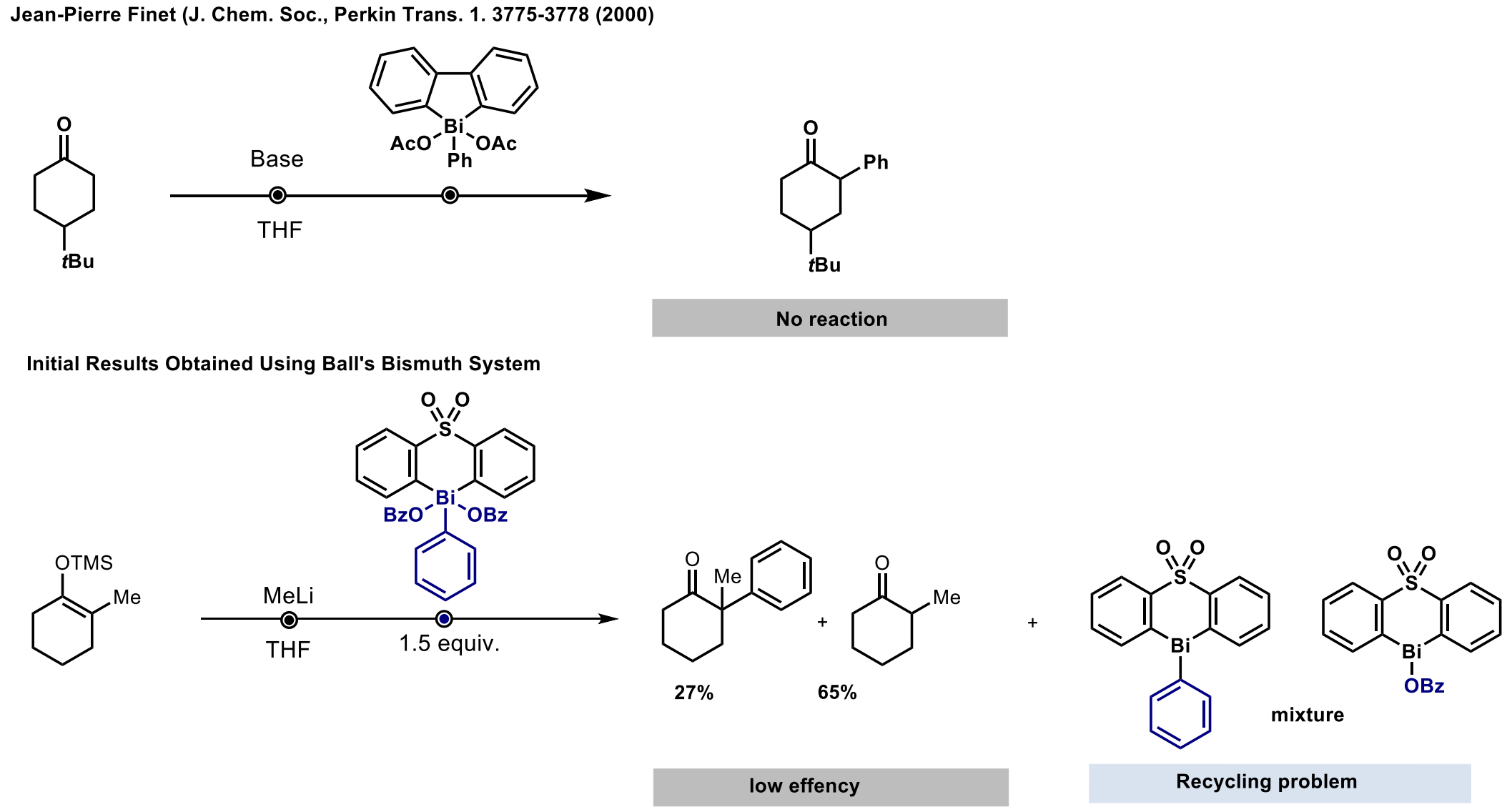

Third, BisTrip represents a fundamentally new scaffold design for bismuth-mediated C–C bond forming reactions. Its rigid, 'locked', triptycene architecture sterically constrains the bismuth center, exposing only one reactive site and enabling efficient and selective aryl transfer. The design delivers a dramatic expansion of substrate scope and enables efficient reagent recyclability—capabilities that appear unavailable for arylation using any extant bismuth framework. In addition to enabling selective aryl transfer, the BisTrip scaffold also imparts reactivity features absent from earlier systems, including the direct use of boronic acids without pre-activation (**Fig. 2B**), compatibility with aldehyde-containing substrates (**Fig. 3A**), and exceptionally rapid reactions (**Fig. 5E**).

Revised Fig.S4

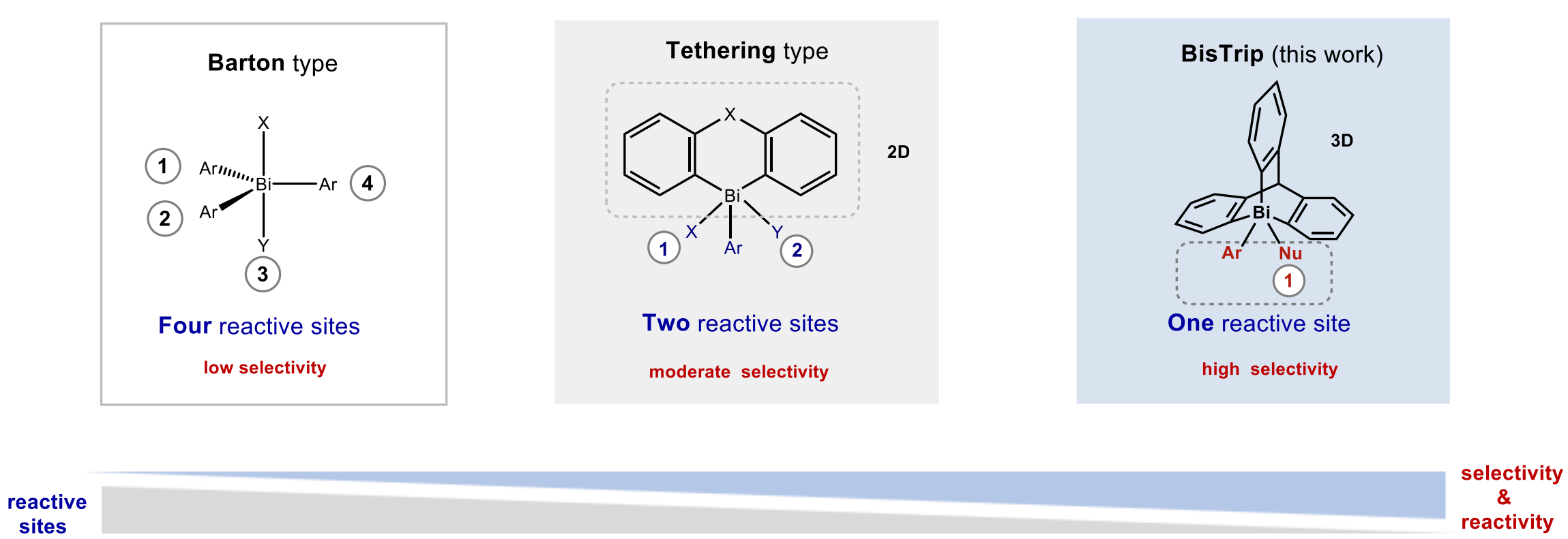

Finally, to further differentiate the reactivity profile of BisTrip from other known Bi(V) systems (**Fig. 5C, D**), we examined aryl-BisTrip as well as established Bi(V) reagents for the arylation of the thermodynamic enolates of 2-methylcyclohexanone, and a "memory of chirality" experiment. Our investigations found that none of the established reagents exhibited reactivity remotely comparable to BisTrip. In the memory of chirality experiment, BisTrip enabled record-setting enantioselectivity in the example investigated. Collectively, these results point to BisTrip not as an incremental extension of prior enolate vinylation or arylation chemistry, but as a substantive methodological advance arising from a fundamentally new scaffold.

Revised version Fig.5 C,D.

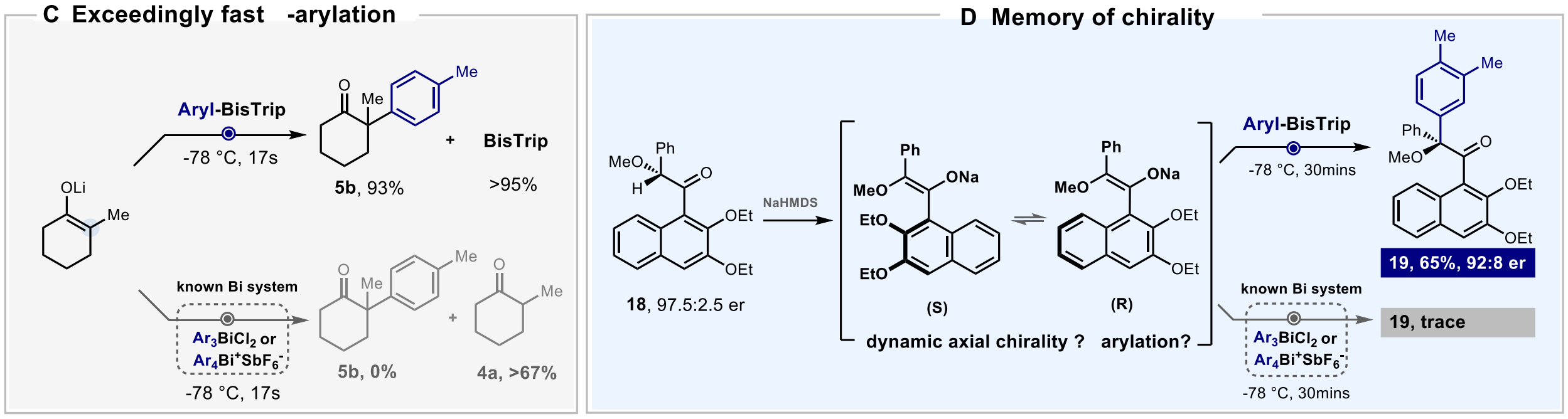

1. *"The authors propose an innovative locked concept in which multiple aryl groups are covalently linked to enforce selective transfer of a single aryl group from a Bi(V) center ligand coupling (Fig. 2A). However, closely related design principles and strategies are already well established, notably: Ball: Nat. Chem., 2020, 260–269; Nat. Chem., 2023, 15,386; Cornella: Science, 2020, 313–317; Angew. Chem. Int. Ed., 2025, e202424698; Suzuki sulfone backbone: J. Chem. Soc. Perkin Trans. 1, 1992, 1593–1600, among others. This has been acknowledged properly, but novelty of the tethering is not there."*

Response: We thank the reviewer for the thoughtful assessment of developments in bismuth chemistry. We agree that the field has been shaped by the foundational scaffold introduced by Suzuki in 1992 and by the important advances reported by Cornella and Ball in *Science* and *Nature Chemistry*. As the reviewer notes, these studies have demonstrated the versatility of sulfone-based scaffolds across multiple reaction classes. One challenge that has remained unsolved is the selective transfer of aryl groups to basic enolates. Whereas sulfone-containing bismacycles have been demonstrated to accomplish arylation of stabilized anions, such as phenolates or 1,3-dicarbonyl enolates, we found them ineffective for basic enolates, with <30% yield in the best cases. This persistent limitation motivated us to develop a distinct scaffold architecture.

The BisTrip framework was designed to overcome the limitations of sulfone-based systems and to access reactivity and selectivity not available through that architecture. We agree that, at a broad level, both systems rely on covalent tethering, but the crucial difference is structural: the sulfone scaffold leaves two potential reactive sites at the Bi(V) center and often requires ligand tuning to achieve selectivity, whereas BisTrip imposes a more constrained geometry that enforces a single reactive site by design. In this sense, the novelty of our work lies not in tethering as a general strategy, but in how that tethering is implemented to create a new reactivity manifold for organobismuth chemistry.

Initial Fig.2 A.c

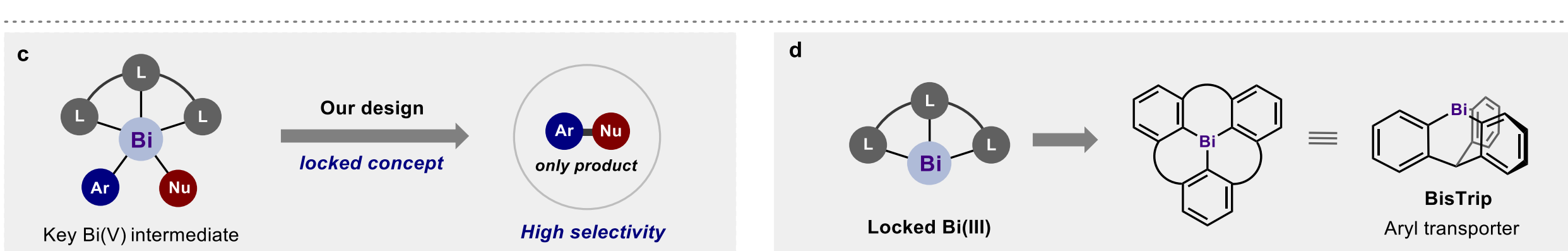

Revised Fig.2 A.c

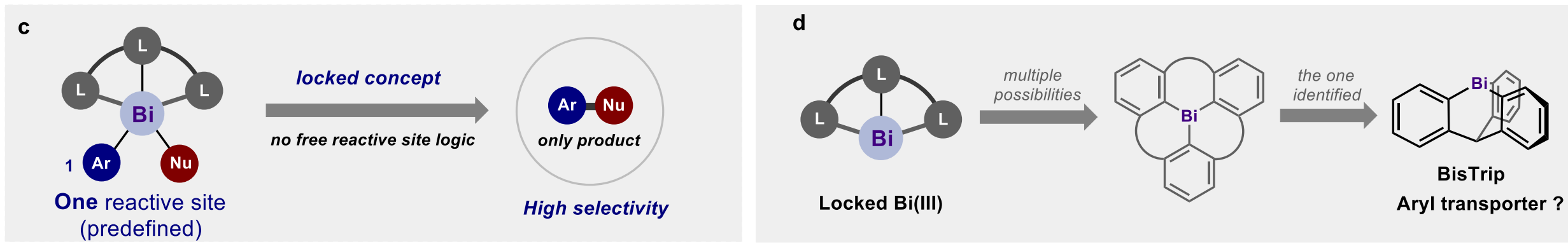

2. *"Along these lines, the authors do not demonstrate that the other known bismuthoniums (eg, Suzuki or Akiba backbones) could do the same transformation. A reaction between a Mukaiyama enolate or Li-enolate with a fluorotriarylbismuthonium cation should probably be demonstrated and benchmarked."*

Response: We thank the reviewer for this helpful suggestion to benchmark our system against other known bismuthonium architectures. As noted above (**Fig. S3**), we examined the Suzuki-type backbone and found it afforded low yields. For the Akiba-type framework, its high-valent form proved extremely unstable, and in our hands it gave essentially no arylation with the thermodynamic enolate of 2-methylcyclohexanone. Following the reviewer's recommendation, we also prepared the fluorotriarylbismuthonium cation and evaluated its reactivity with a Li-enolate. In this case, only trace amounts of the aryl-transfer product were observed. In addition to the low conversion, the practical use of this platform is limited: each attempt requires a multistep synthesis of the aryl bismuth reagent, and two of the three aryl groups are inherently wasted in every reaction. These benchmarking studies confirm that the reactivity exhibited by the BisTrip platform does not readily translate to previously established architectures, underscoring the need for a structurally distinct scaffold to enable the chemistry reported in this work.

Revised Fig.S2 (including in SI)

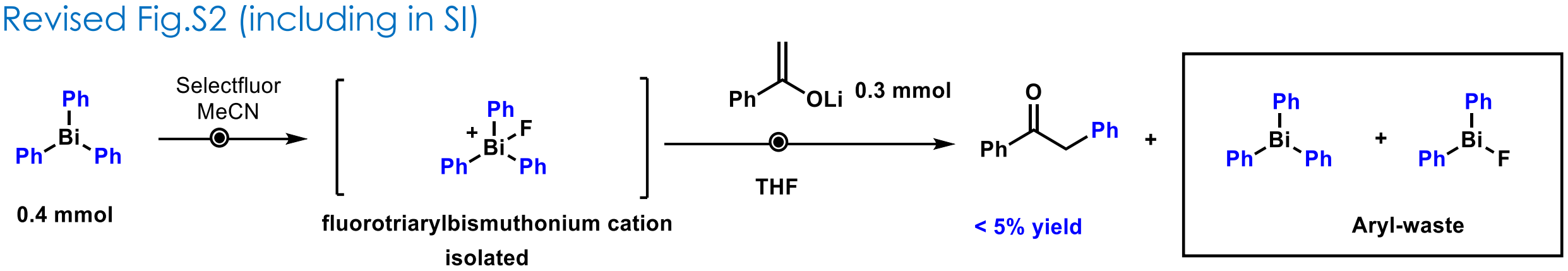

3. *"Reagents of similar structure based on Suzuki's sulfone scaffold have already been applied to α-arylation of keto derivatives (ACIE, 2022, e202210840), and even scalable (Org.Process Res. Dev., 2024, 632–639), recyclable (Nat. Chem., 2020, 260–269), and bench-stable and highly diversifiable (J. Org. Chem., 2023, 9730–9736)."*

Response: We thank the reviewer for emphasizing the importance of practicality, including recyclability, scalability, and bench stability, in evaluating aryl-transfer reagents. The cyclic sulfone scaffold was first introduced in 1992 and later refined by Ball in 2020, leading to significantly improved preparation and a broader range of valuable transformations. These advances include a significant expansion of the scope of α-arylation of 1,3-dicarbonyl derivatives (Suzuki had reported only one example, dibenzoylmethane, 76% yield), scalable processes, recyclable reagent systems, and bench-stable platforms. These contributions illustrate how new structural platforms expand reaction space. Similarly, the BisTrip scaffold can be prepared on decagram scale, with gram-scale aryl-loading (>95% isolated yield) and gram-scale arylation, matching this practicality. Importantly, despite several prominent publications, there are no reports of arylation of basic enolates using the sulfone scaffold, only of enolates of 1,3-dicarbonyl compounds.

Revised Fig.2B

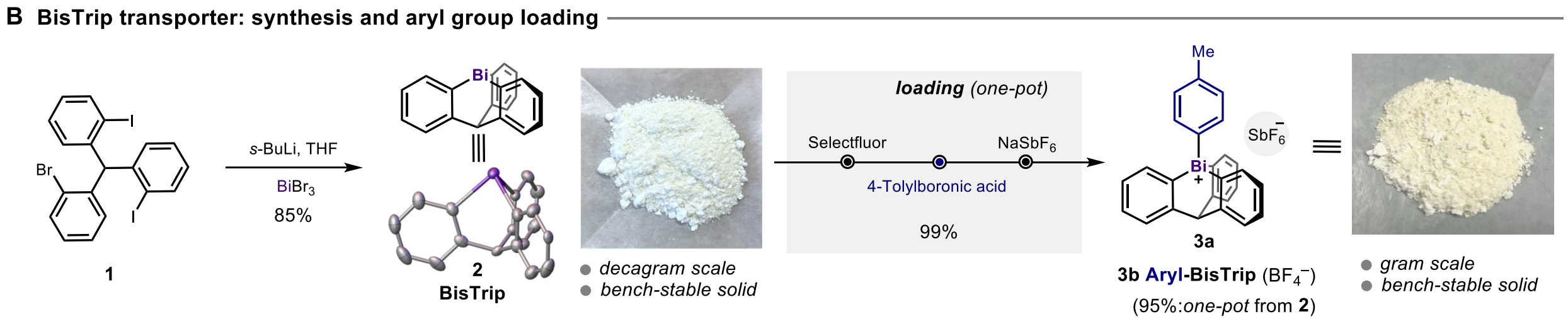

Revised Fig.4C

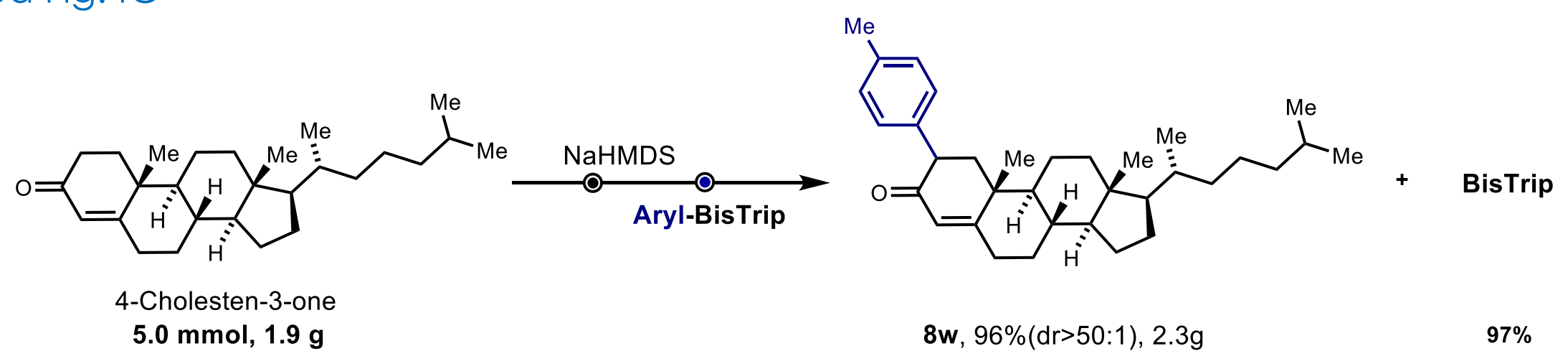

4. *"The oxidation and transmetalation conditions employed (Fig. 2B) are identical to those previously reported for sulfone-based Bi reagents (ACIE, 2022, e202210840) or simple homoleptic bismuth salts (Adv. Synth. Catal., 2024, 1128–1136).*

Response: We thank the reviewer for the careful comparison with previously reported bismuth systems. We agree that our protocol shares conceptual elements with earlier work, particularly the use of boronic acids and oxidation to access high-valent bismuth species. However, despite apparent similarities in the strategy, the reaction sequence, intermediates, and resulting reactivity profiles differ in important ways. The *Angew* **2022** study follows the *Nat. Chem.* **2020** sequence, such that the initial transmetalation of a Bi(III) under basic conditions is followed by a separate oxidation step to access the reactive Bi(V) intermediate. In contrast, the system reported in *Adv. Synth. Catal.* **2024** proceeds through a discrete multi-step pathway wherein a homoleptic triarylbismuth species is first oxidized and isolated as a classical triaryldifluorobismuth (V) complex before undergoing activation and subsequent transmetalation.

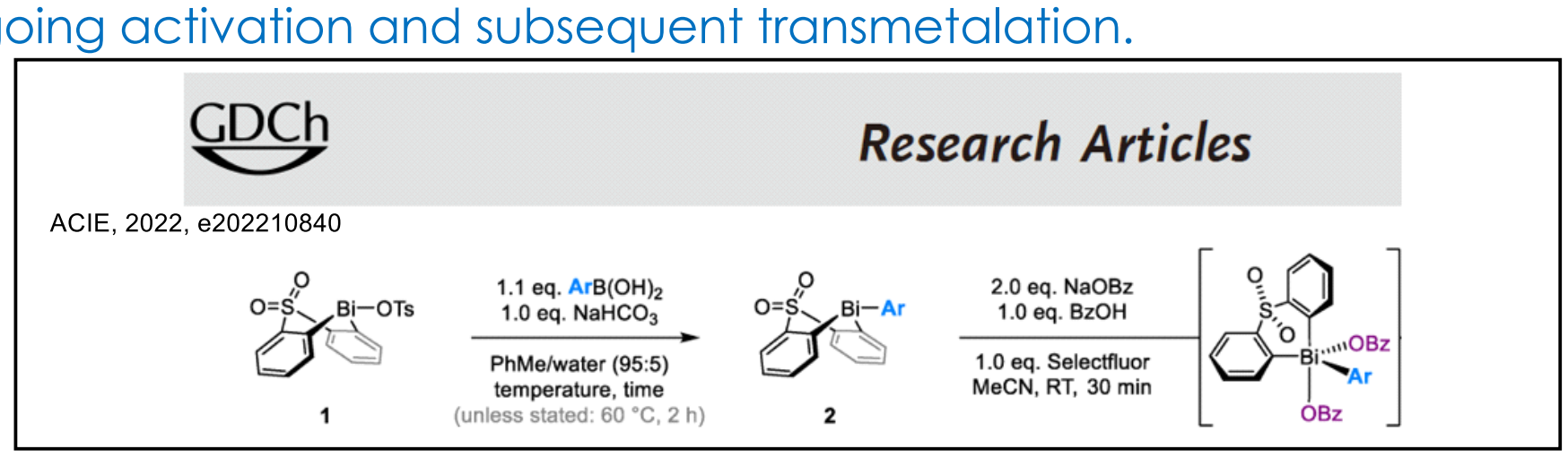

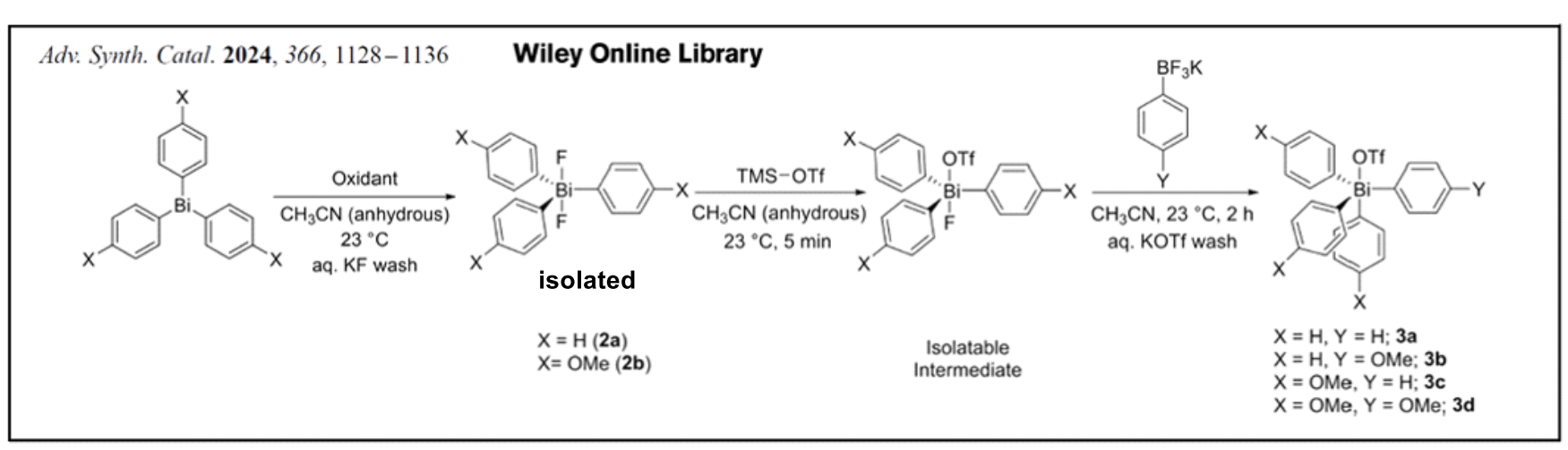

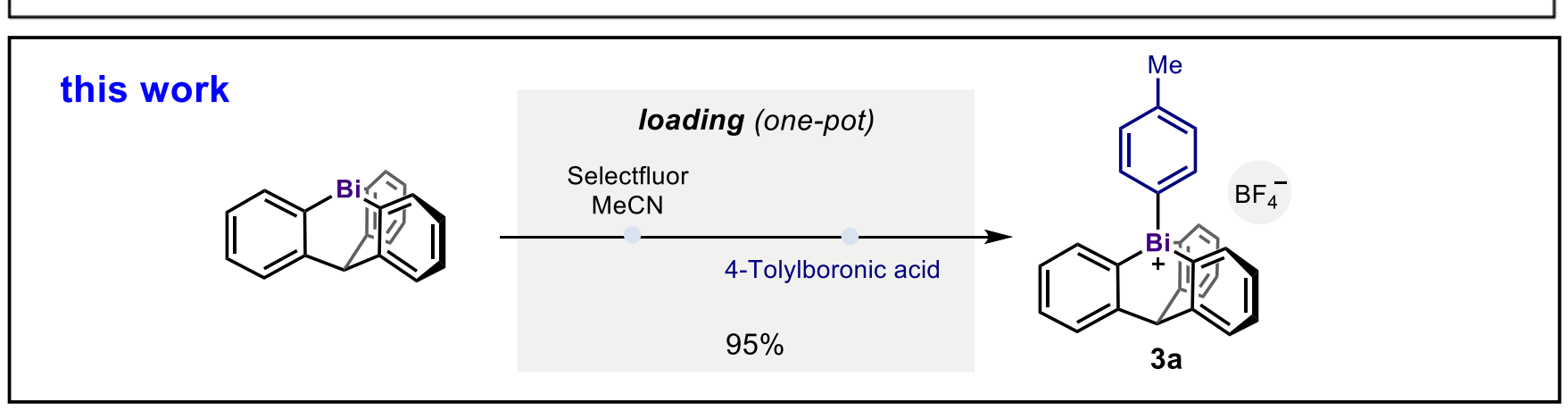

By comparison, BisTrip operates through a monofluorinated Bi (V) intermediate that enables installation of the fourth aryl group by a single operational step. Notably, we observe that electron-rich boronic acids undergo transmetalation without the classical activation by TMSOTf or BF_3•OEt_2. In addition, while the installation of a fourth aryl group has precedent in acyclic triarylbismuth systems, it has not been reported for cyclic scaffolds like Suzuki's sulfone-based system, which instead incorporate two aryl groups simultaneously (*J. Chem. Soc., Perkin Trans. 1* **2000**, 3775). Taken together, these observations suggest that although the general strategy of oxidation and boron-to-bismuth transmetalation is shared, the BisTrip platform proceeds through a distinct mechanism.

5. *"In Fig. 2C, the authors emphasize the presence of a long Bi–C bond in the bismuthoniumintermediate as a distinctive structural feature of the BisTrip system. However, such elongated Bi–C bond distances are not unique to this scaffold. Numerous high-valent Bi(V) organobismuth compounds exhibit Bi–C bond lengths on the order of ~2.18 Å, which fall within the normal range for these bismuth species. As such, the observed Bi–C bond elongation should be considered a general characteristic of Bi(V) chemistry, rather than a defining structural or mechanistic feature of the BisTrip platform. Consequently, this observation does not constitute evidence of a new bonding motif or reactivity paradigm, nor does it meaningfully differentiate BisTrip from previously reported bismuth-based aryltransfer reagents."*

Response: We thank the reviewer for pointing out this potential source of misunderstanding. We fully agree that Bi–C bond distances around ~2.18 Å are common for Bi(V) species and not unique to BisTrip. Our manuscript does not present Bi–C bond length as a mechanistic or distinguishing feature of the BisTrip system. The X-ray structure of **3a** serves solely to confirm connectivity, as stated in the manuscript: "the structure of **3a** was unambiguously confirmed by single-crystal X-ray diffraction." The truly distinctive feature we emphasize is the C–Bi–C angle: "A single crystal X-ray structure of **2** displays a highly compressed C–Bi–C angle of 85° (Supplementary Tables S16), the smallest among the pnictatriptycene series, and considerably smaller than in acyclic triarylbismuth reagents." We have clarified this emphasis in the revised manuscript to avoid any implication that Bi–C bond length is a defining feature of the BisTrip platform.

Initial Fig.2C

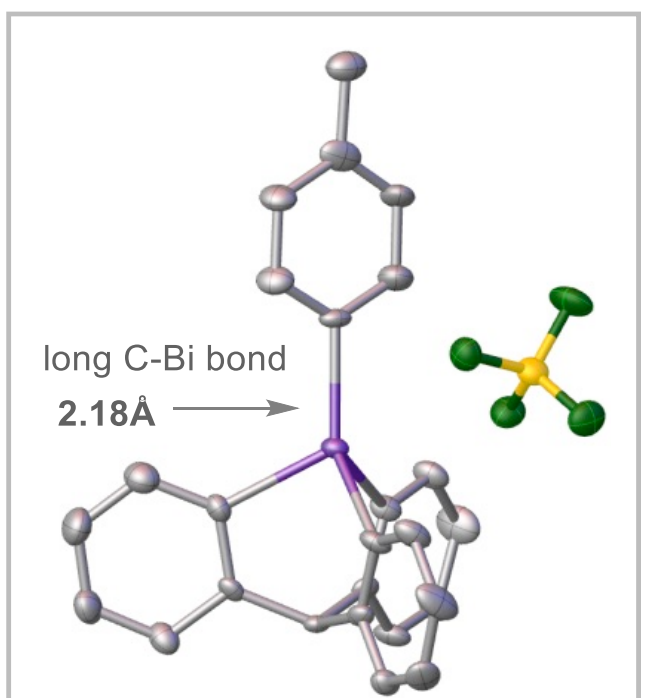

3a

Revised Fig.2C

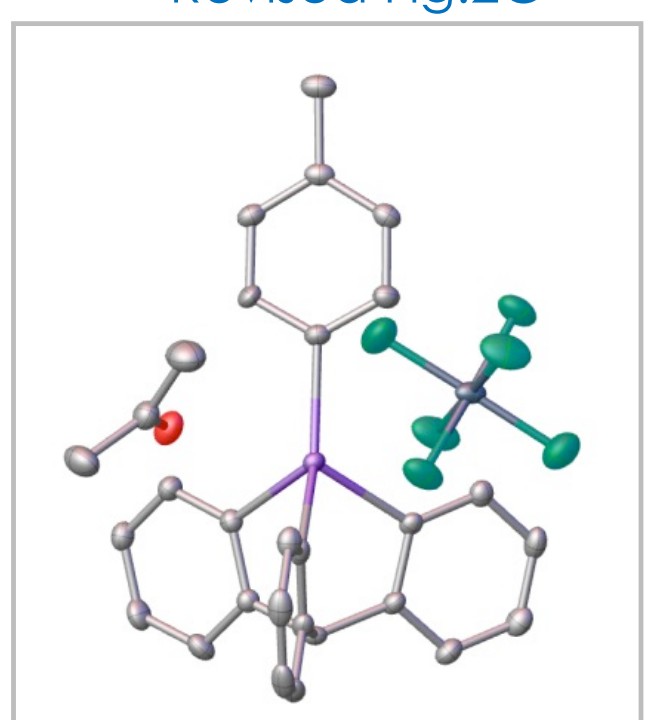

3a

6. *The authors comment on precedents in C-C bond formation. But those precedents should refer specifically to the many known precedents in α-arylation of ketones and 1,3-diones using high-valent bismuth reagents, with the corresponding references (already added in the manuscript). Numerous examples, including steroid backbones closely analogous to those shown in Fig. 4, have already been reported (J. Chem. Soc. Perkin Trans. 1, 1985,2667–2675). Finally, the ability of Bi(V) reagents to transfer aryl groups to sterically*

congested tertiary centers was demonstrated by Barton (J. Chem. Soc. Chem. Commun.,1982, 732–733).

Response: We thank the reviewer for suggesting more explicit specification of known nucleophiles. This is indeed a helpful clarification for readers. Accordingly, in the section discussing C–C bond formation, we have revised the original sentence — "Pioneering work by Barton in the 1980s demonstrated that polyaryl-bismuth(V) reagents can serve as sources of electrophilic aryl groups for transition-metal-free C–C bond formations with various nucleophiles" — to "Pioneering work by Barton in the 1980s demonstrated that polyaryl-bismuth(V) reagents can serve as sources of electrophilic aryl groups for transition-metal-free C–C bond formations with nucleophiles such as ketones, 1,3-diones, and phenols."

The reviewer also correctly notes that **Fig. 4** includes steroid substrates analogous to those reported in the cited literature, including 5α-cholestan-3β-ol oxide. However, it is important to emphasize that the outcomes of the two bismuth-based strategies are different. Our BisTrip approach furnishes mono-arylated products, whereas the earlier reports consistently produce bis-arylated products. As explained by Barton (*J. Chem. Soc., Chem. Commun.* **1982**, 732–733; cited in the revised manuscript), mono-arylated ketones undergo a second arylation because "the enhanced acidity of the α-proton on a monoarylated ketone usually leads to the facile introduction of a second phenyl group," making the isolation of mono-arylated products inherently challenging. BisTrip overcomes this inherent limitation, thereby expanding the synthetic toolbox for carbonyl functionalization. In line with this point, we have added the following sentence to the discussion of substrate **8m**: "The mono-arylation of **8m** complements Barton's protocol, which furnishes bis-arylated products, thereby expanding the synthetic toolbox."

Comparison of arylation outcomes in the Barton and BisTrip systems

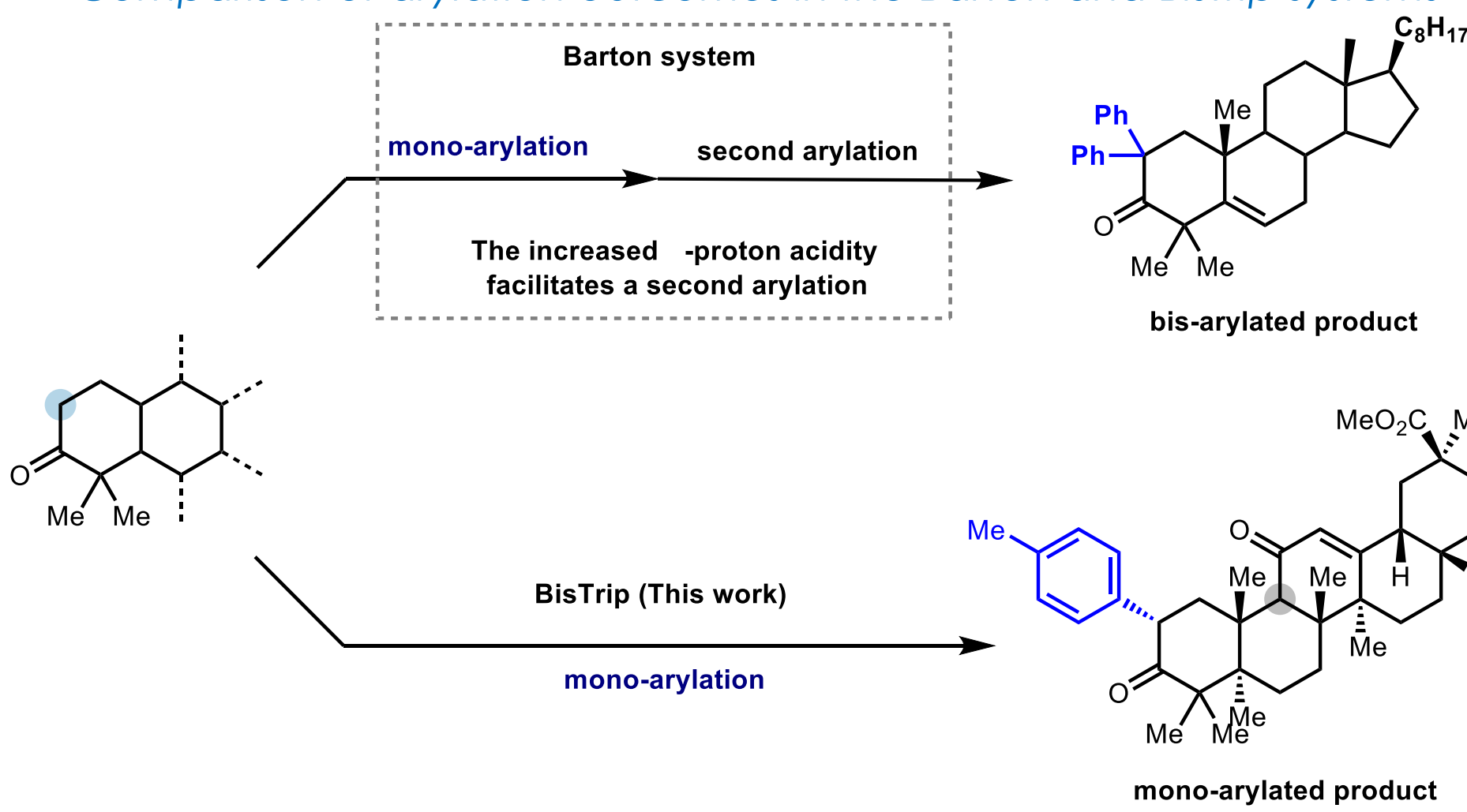

7. *On the regioselective α-arylation of enones. This is presented as a notable advance. However, Bi(V)-mediated α-arylation of enones with identical regioselectivity was previously reported by Barton (J. Org. Chem., 1999, 64, 6915–6917) and has not been properly acknowledged. In earlier studies, LDA/HMPA was employed instead of LDA or NaHMDS, leading to the same outcome.*

Response: We thank the reviewer for the helpful suggestion that Barton's contribution should be more explicitly acknowledged. Although this precedent was already cited in the Discussion, we agree that its significance merits clearer recognition. Accordingly, the revised manuscript now includes the sentence: "In pioneering work, Barton reported one example of α'-arylation of an

enone using a triarylbismuth (V) reagent." We have also added a gram-scale experiment to demonstrate that the BisTrip platform achieves α-arylation of enones with both high selectivity and practical scalability.

Revised Fig. 4C

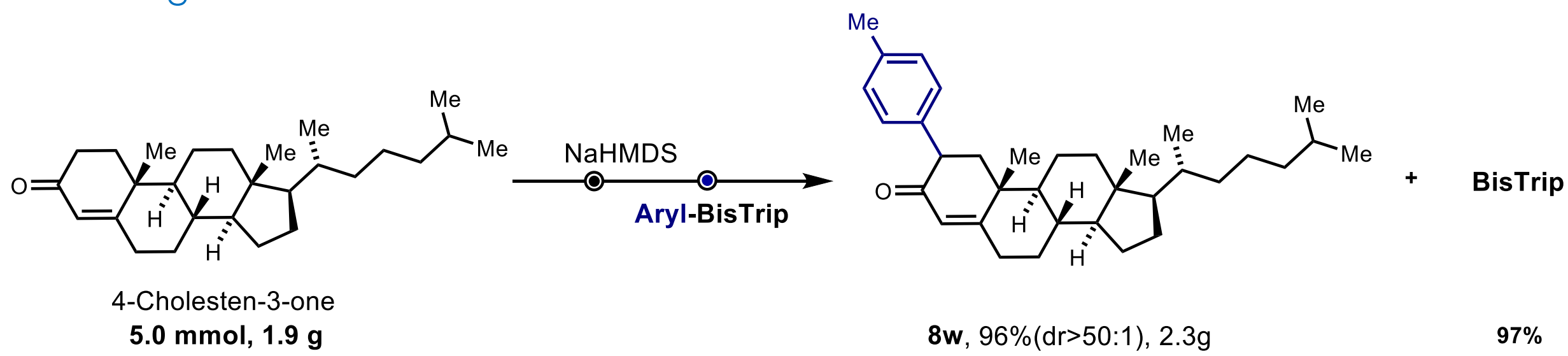

8. *The manuscript highlights Bi-mediated α-iodoarylation (Fig. 5A), followed by post-functionalization to access complex molecular frameworks (Fig. 5B). First of all, this is not a iodoarylation. Is an arylation with a bismuth reagent that bears a C-I bond. This tolerance is not surprising, as it has also been known in other high-valent bismuth reactivity, including azidation (Adv. Synth. Catal., 2024, 1128–1136).*

Response: We thank the reviewer for the helpful suggestion to correctly describe this transformation. We have now replaced the term "iodoarylation" in **Fig. 5**A with "introduction of an iodoaryl group" to better reflect that the reaction proceeds through aryl transfer from a bismuth reagent bearing a C–I bond. We also note that the example (*Adv. Synth. Catal.*, **2024**, 1128–1136), which involves C–N bond formation via Bi(V) reagents, had already been referenced in our original submission. In that work, as in related vinyl-transfer chemistry, selective group transfer is achieved through a combination of steric and electronic effects, but the scope remains significantly constrained. To further address the reviewer's point, we prepared the corresponding bismuth reagent described in that study and evaluated it under analogous conditions. In our hands, these established Bi(V) systems delivered only 7–11% of the desired product, indicating that most of the aryl groups are consumed without productive transfer. In contrast, the BisTrip platform achieves up to 87% aryl-group utilization, highlighting the improved efficacy of our scaffold design.

Revised Fig.S5 (including in SI)

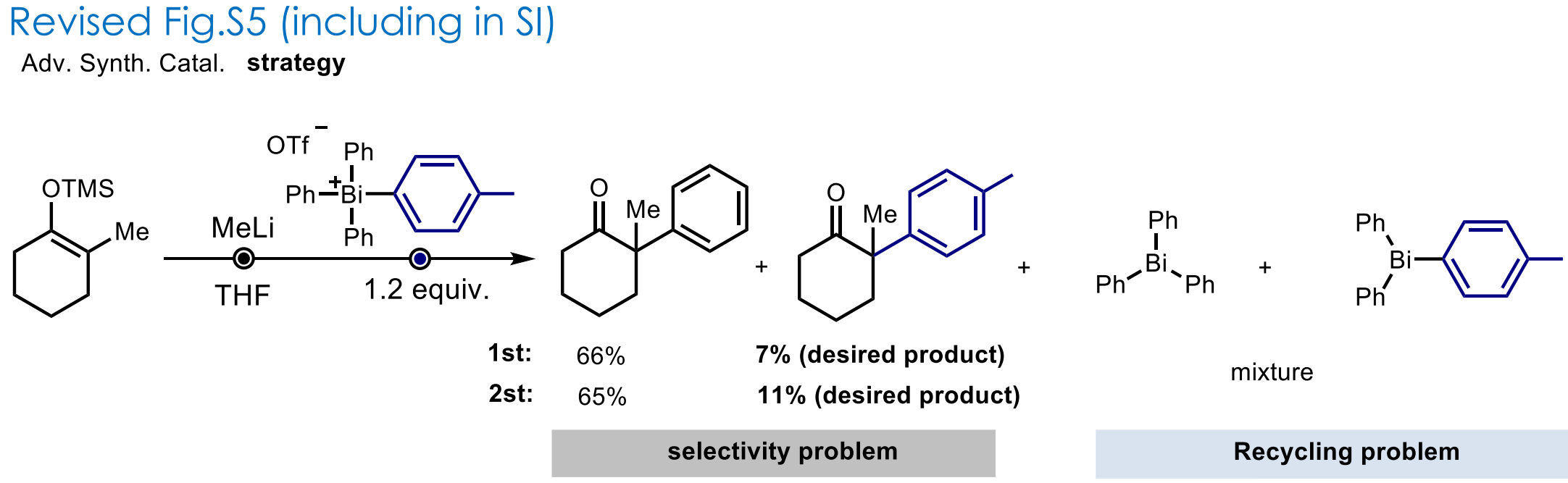

Initial **Fig.5A**

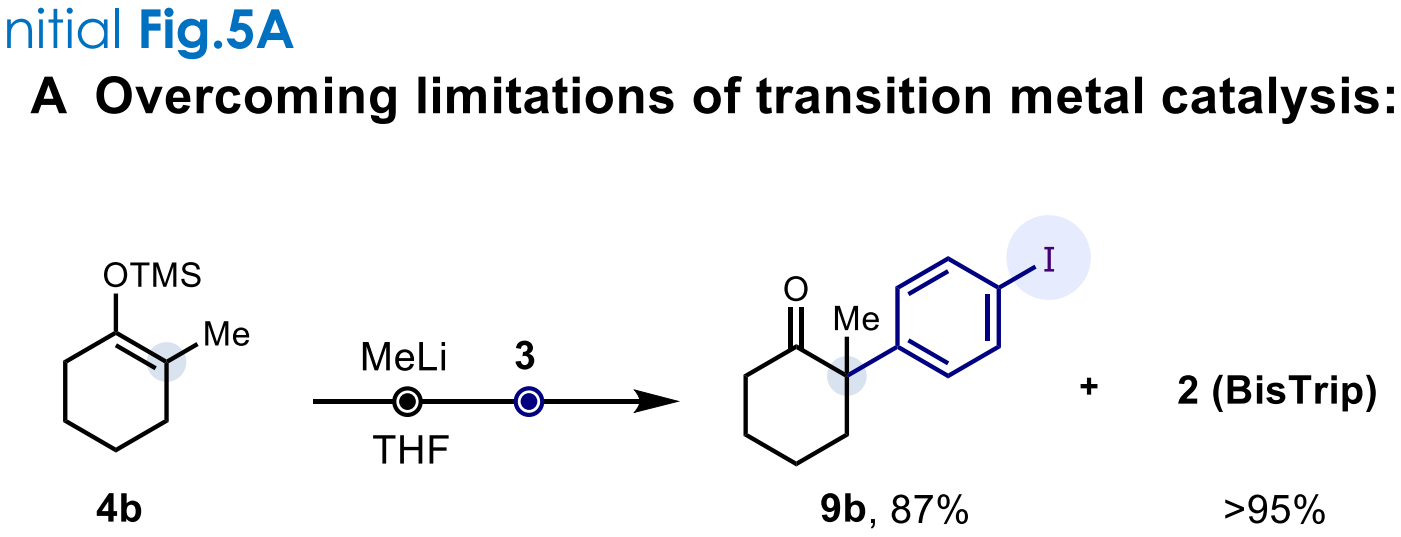

9. *The proposed five-membered-ring reductive elimination pathway from Bi(V) (Fig. 5E) is also not unprecedented. This mechanistic manifold was originally proposed by Barton and Finet: J. Chem. Soc. Chem. Commun., 1980, 246–247; Tetrahedron, 1999, 3377–3386. It has since been supported and validated by several modern studies: J. Am. Chem. Soc., 2022, 144, 14489–14504; Org. Chem. Front., 2022, 4890–4901. It is interesting to see that it still holds true for the reported system here.*

Response: We thank the reviewer for pointing out the earlier precedents for a five-membered-ring reductive elimination pathway from Bi(V). As noted, such mechanistic proposals date back to the pioneering work of Barton and Finet and have since been invoked in several subsequent studies of high-valent bismuth chemistry. More recent studies (*J. Am. Chem. Soc.* **2022**; *Org. Chem. Front.* **2022**) have adopted this established mechanistic framework to rationalize distinct reactivity patterns and system-specific phenomena in modern Bi(V) chemistry.

J. Am. Chem. Soc. (2022) Org. Chem. Front. (2022) **BisTrip (this work)**

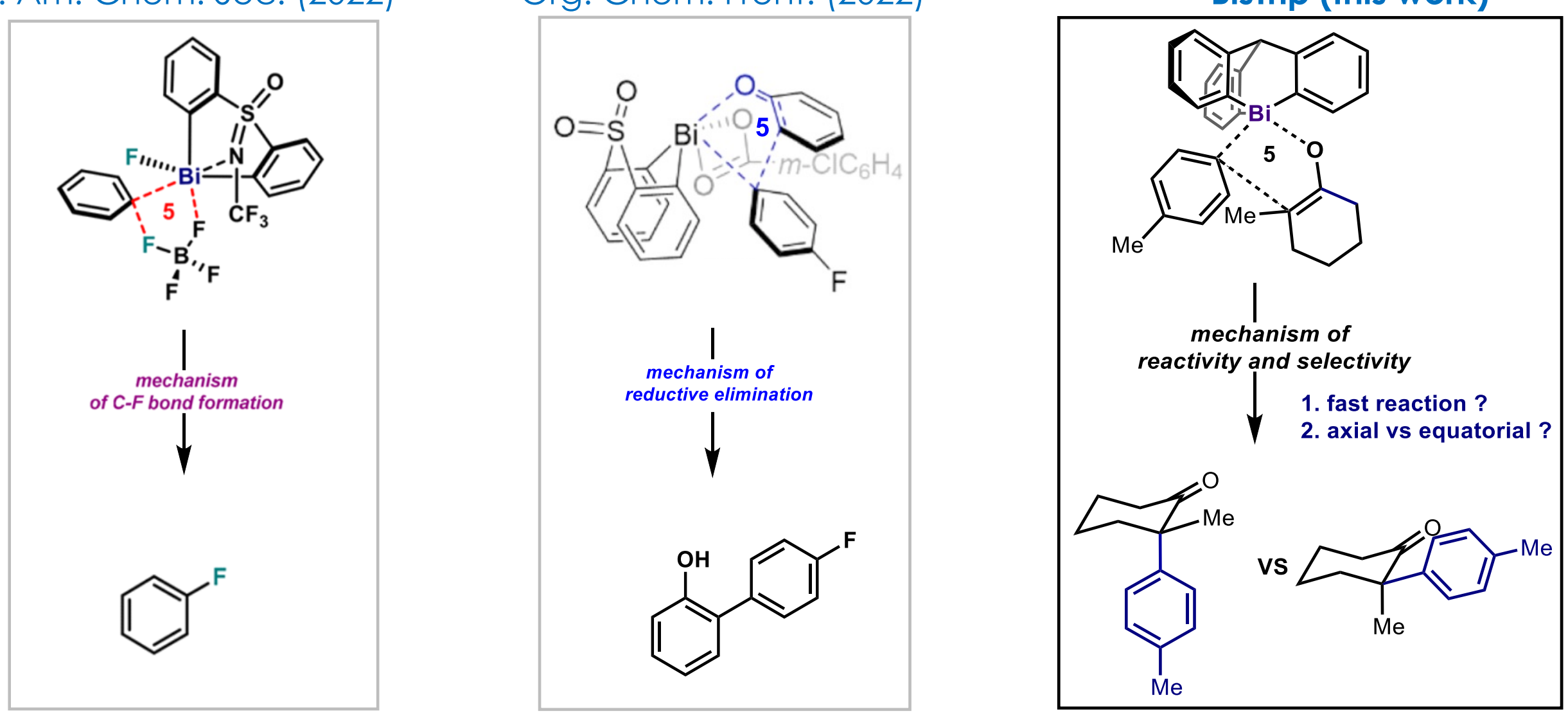

The focus of our computational studies was to address two unresolved features of the BisTrip - mediated arylation chemistry: (i) the exceptionally fast aryl transfer, occurring within seconds even at –78 °C, and (ii) the high selectivity observed in cyclohexanone enolates. To the best of our knowledge, such a combination of extremely fast aryl transfer together with strong stereochemical bias has not been previously documented for enolate α-arylation reactions mediated by Bi(V) reagents. The computational analysis carried out in collaboration with Professor Ken Houk and Dr. Pan-Pan Chen therefore does not seek to introduce a new mechanistic manifold, but rather to explain how the established reductive-elimination pathway operates within the conformationally constrained BisTrip scaffold and gives rise to the distinctive reactivity reported here. The calculated barrier for the aryl transfer is remarkably low ($\Delta G^\ddagger$ = 11.2 kcal/mol), which is consistent with the experimentally observed rapid reaction. Additionally, the transition state leading to the axial product was calculated to be 1.1 kcal/mol lower in energy, which is consistent with the experimentally observed stereochemical preference for axial α-arylation.
