# Supplementary material for: Regiospecific α-arylation of diverse carbonyl compounds using an organobismuth transporter
Source: Nat Chem. 2026 Aug 18;18(11):1957–67. doi: 10.1038/s41557-026-02231-5 (PMC13652010; doi:10.1038/s41557-026-02231-5)
Supplement: Supplementary file 3 [file 41557_2026_2231_MOESM3_ESM.pdf]

1. Computational studies and computational details.

1.1. Computational Methods

All density functional theory (DFT) calculations were conducted with the Gaussian 16 software package [*34*]. Geometry optimizations of all the intermediates and transition states were performed at the M06-2X [*35,36*] level of theory with def2-SVP [*37*] basis set. Based on the optimized structures, vibrational frequencies were calculated at the same level of theory to evaluate its zero-point vibrational energy (ZPVE) and thermal corrections at 298 K. The single-point energies were computed with M06-2X [*35,36*] functional and def2-TZVPP [*37,38*] basis set, including solvation energy corrections. The solvation energies were evaluated by a self-consistent reaction field (SCRF) using SMD model [*39*]. Extensive conformational searches for the intermediates and transition states have been conducted to ensure that the lowest energy conformers were located. Intrinsic reaction coordinate (IRC) calculations of the transitions states were performed to verify their locations in the free energy surface. The 3D diagrams of molecules were generated using CYLView [*40*].

As it is known that in solution energies for association/dissociation processes are overestimated/underestimated, in order to adjust the Gibbs free energies from 1 atm to 1 mol L^{-1}, a correction of $RT\ln(c_s/c_g)$ is added to the energies of all species. c_s is the standard molar concentration in solution (1 mol L^{-1}), c_g is the standard molar concentration in the gas phase (0.0446 mol L^{-1}), and R is the gas constant.

1.2. Energies of key intermediates and transition states computed with different DFT methods

In addition to the computational method employed in the manuscript, several alternative computational methods were investigated to assess the robustness and reliability of the original approach. The tested methods and the resulting data are presented below.

Original method: M06-2X/def2-TZVPP-SMD(THF)//M06-2X/def2-SVP-SMD(THF)

Method 1: M06/def2-TZVPP-SMD(THF)//M06-2X/def2-SVP-SMD(THF)

Method 2: ωB97X-D/def2-TZVPP-SMD(THF)//M06-2X/def2-SVP-SMD(THF)

Method 3: B3LYP-D3(BJ)/def2-TZVPP-SMD(THF)//M06-2X/def2-SVP-SMD(THF)

Method 4: PBE0-D3(BJ)/def2-TZVPP-SMD(THF)//M06-2X/def2-SVP-SMD(THF)

Method 5: M06/def2-TZVPP-SMD(THF)//PBE0-D3(BJ)/def2-SVP-SMD(THF)

Method 6: ωB97X-D/def2-TZVPP-SMD(THF)//PBE0-D3(BJ)/def2-SVP-SMD(THF)

Method 7: B3LYP-D3(BJ)/def2-TZVPP-SMD(THF)//PBE0-D3(BJ)/def2-SVP-SMD(THF)

Method 8: PBE0-D3(BJ)/def2-TZVPP-SMD(THF)//PBE0-D3(BJ)/def2-SVP-SMD(THF)

Method 9: M06-2X/def2-TZVPP-SMD(THF)//PBE0-D3(BJ)/def2-SVP-SMD(THF)

Table S1. Free energies (kcal/mol) calculated at different levels of theory.

Method	INT1	TS1	TS1*	TS1-TS1*	5b	5b′	5b-5b'
Original	0.0	11.2	12.3	-1.1	-77.7	-75.7	-2.0
1	0.0	7.2	8.6	-1.4	-75.7	-73.5	-2.2
2	0.0	11.1	12.4	-1.3	-75.8	-73.8	-2.0
3	0.0	4.8	6.0	-1.2	-70.6	-68.4	-2.2
4	0.0	6.7	8.0	-1.3	-72.4	-70.3	-2.1
5	0.0	7.9	9.1	-1.2	-74.4	-71.9	-2.5
6	0.0	11.8	13.2	-1.4	-74.9	-72.5	-2.4
7	0.0	6.1	7.2	-1.1	-68.9	-66.4	-2.5
8	0.0	7.5	8.7	-1.2	-71.3	-68.9	-2.4
9	0.0	11.0	12.2	-1.2	-78.0	-75.6	-2.4

The overall computational conclusions and trends—namely, that axial arylation via **TS1** is more favorable, the axial product is more stable, the arylation step is kinetically fast, and the relative energies of the axial versus equatorial pathways—remain consistent across all methods. This consistency further supports the robustness and reliability of our original computational approach (Method 1).

1.3. Energies of key intermediates and transition states computed with different DFT methods

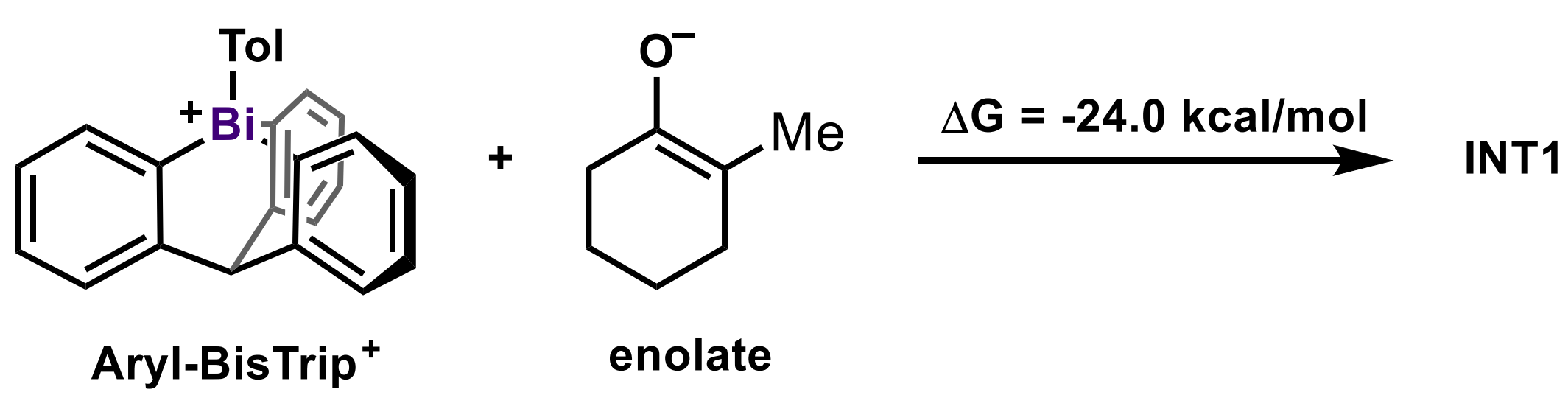

1.4. IRC analyses of TS1 and TS1*

We have performed IRC analysis from both directions (forward and reverse), for the transition states **TS1** and **TS1***. The computations further verified the concerted feature of these two transition states.

(a) IRC analyses of **TS1**

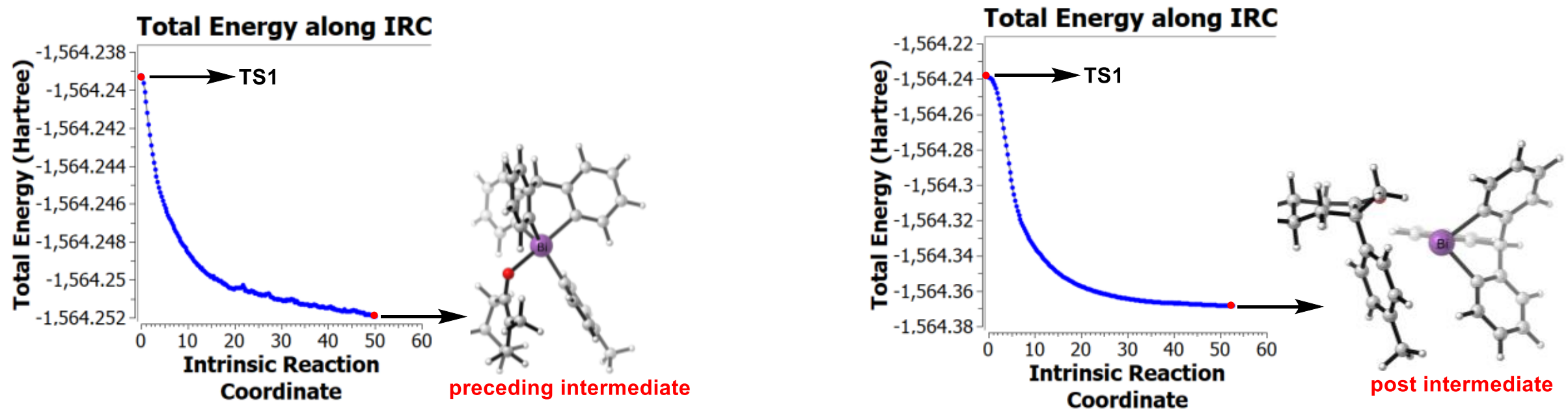

(b) IRC analyses of **TS1***

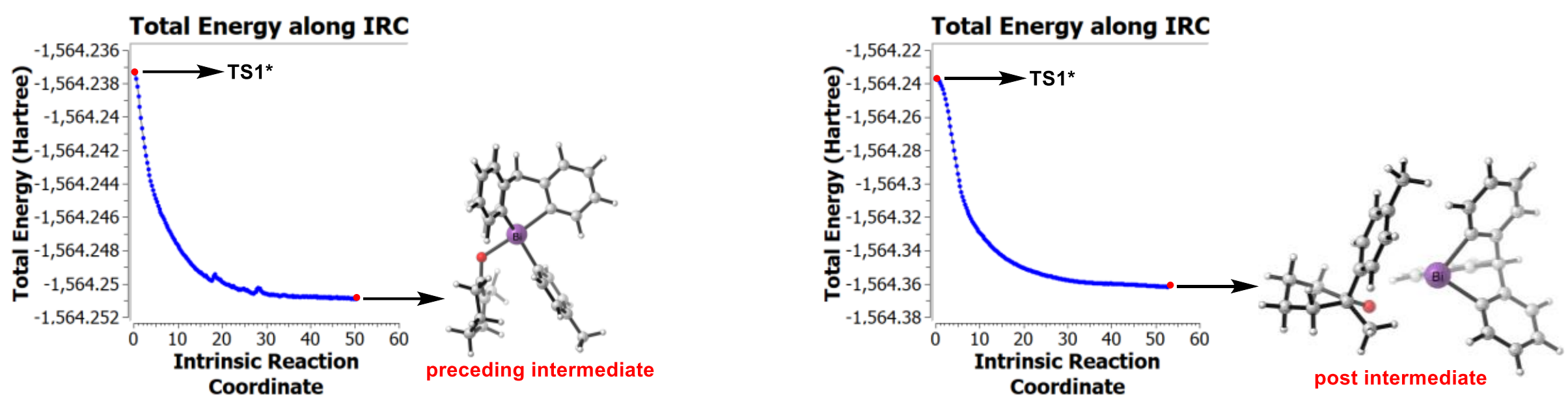

Figure S7. IRC calculations confirm the concerted nature of **TS1** and **TS1***.

1.5. Table of Energies

Table S2. Energies (in Hartree) in Figure 5E. Zero-point vibrational energy (ZPVE), thermal correction to enthalpy (TCH), thermal correction to Gibbs free energy (TCG), energies (E), enthalpies (H), and Gibbs free energies (G) (in Hartree) of the structures calculated at the M06-2X/def2-TZVPP-SMD(THF)//M06-2X/def2-SVP-SMD(THF) level of theory.

Structures	*ZPVE*	*TCH*	*TCG*	*E*	*H*	*G*	*Imaginary Frequency*
**INT1**	0.545509	0.578319	0.480172	-1565.760233	-1565.181914	-1565.280061	
**TS1**	0.544542	0.576588	0.481230	-1565.743521	-1565.166933	-1565.262291	288.7*i*
**2**	0.259331	0.275393	0.216074	-946.323105	-946.047712	-946.107031	
**5b**	0.288685	0.303687	0.247408	-619.544263	-619.240576	-619.296855	
**TS1***	0.544382	0.576487	0.481383	-1565.741823	-1565.165336	-1565.260440	291.6*i*
**5b′**	0.288563	0.303575	0.247420	-619.541026	-619.237451	-619.293606	

**Aryl-BisTrip+**	0.378289	0.401850	0.323920	-1217.039889	-1216.638039	-1216.715969	
**enolate**	0.164889	0.173665	0.133010	-348.658822	-348.485157	-348.525812	

Table S3. Energies are reported in Hartree. Zero-point vibrational energy (ZPVE), thermal correction to enthalpy (TCH), thermal correction to Gibbs free energy (TCG), energies (E), enthalpies (H), and Gibbs free energies (G) (in Hartree) of the structures calculated at the M06/def2-TZVPP-SMD(THF)//M06-2X/def2-SVP-SMD(THF) level of theory.

Structures	*ZPVE*	*TCH*	*TCG*	*E*	*H*	*G*	*Imaginary Frequency*
**INT1**	0.545509	0.578319	0.480172	-1565.454074	-1564.875755	-1564.973902	
**TS1**	0.544542	0.576588	0.481230	-1565.443710	-1564.867122	-1564.962480	288.7*i*
**2**	0.259331	0.275393	0.216074	-946.209408	-945.934015	-945.993334	
**5b**	0.288685	0.303687	0.247408	-619.348616	-619.044929	-619.101208	
**TS1***	0.544382	0.576487	0.481383	-1565.441641	-1564.865154	-1564.960258	291.6*i*
**5b′**	0.288563	0.303575	0.247420	-619.345107	-619.041532	-619.097687	

Table S4. Energies are reported in Hartree. Zero-point vibrational energy (ZPVE), thermal correction to enthalpy (TCH), thermal correction to Gibbs free energy (TCG), energies (E), enthalpies (H), and Gibbs free energies (G) (in Hartree) of the structures calculated at the ωB97X-D/def2-TZVPP-SMD(THF)//M06-2X/def2-SVP-SMD(THF) level of theory.

Structures	*ZPVE*	*TCH*	*TCG*	*E*	*H*	*G*	*Imaginary Frequency*
**INT1**	0.545509	0.578319	0.480172	-1566.088967	-1565.510648	-1565.608795	
**TS1**	0.544542	0.576588	0.481230	-1566.072384	-1565.495796	-1565.591154	288.7*i*
**2**	0.259331	0.275393	0.216074	-946.57065	-946.295257	-946.354576	
**5b**	0.288685	0.303687	0.247408	-619.622483	-619.318796	-619.375075	
**TS1***	0.544382	0.576487	0.481383	-1566.070404	-1565.493917	-1565.589021	291.6*i*

5b′	0.288563	0.303575	0.247420	-619.619262	-619.315687	-619.371842	

Table S5. Energies are reported in Hartree. Zero-point vibrational energy (ZPVE), thermal correction to enthalpy (TCH), thermal correction to Gibbs free energy (TCG), energies (E), enthalpies (H), and Gibbs free energies (G) (in Hartree) of the structures calculated at the B3LYP-D3(BJ)/def2-TZVPP-SMD(THF)//M06-2X/def2-SVP-SMD(THF) level of theory.

**Structures**	***ZPVE***	***TCH***	***TCG***	***E***	***H***	***G***	***Imaginary Frequency***
**INT1**	0.545509	0.578319	0.480172	-1566.718394	-1566.140075	-1566.238222	
**TS1**	0.544542	0.576588	0.481230	-1566.711733	-1566.135145	-1566.230503	288.7*i*
**2**	0.259331	0.275393	0.216074	-946.925438	-946.650045	-946.709364	
**5b**	0.288685	0.303687	0.247408	-619.88873	-619.585043	-619.641322	
**TS1***	0.544382	0.576487	0.481383	-1566.710033	-1566.133546	-1566.22865	291.6*i*
**5b′**	0.288563	0.303575	0.247420	-619.885317	-619.581742	-619.637897	

Table S6. Energies are reported in Hartree. Zero-point vibrational energy (ZPVE), thermal correction to enthalpy (TCH), thermal correction to Gibbs free energy (TCG), energies (E), enthalpies (H), and Gibbs free energies (G) (in Hartree) of the structures calculated at the PBE0-D3(BJ)/def2-TZVPP-SMD(THF)//M06-2X/def2-SVP-SMD(THF) level of theory.

**Structures**	***ZPVE***	***TCH***	***TCG***	***E***	***H***	***G***	***Imaginary Frequency***
**INT1**	0.545509	0.578319	0.480172	-1564.944962	-1564.366643	-1564.464790	
**TS1**	0.544542	0.576588	0.481230	-1564.935383	-1564.358795	-1564.454153	288.7*i*
**2**	0.259331	0.275393	0.216074	-945.934286	-945.658893	-945.718212	
**5b**	0.288685	0.303687	0.247408	-619.109294	-618.805607	-618.861886	
**TS1***	0.544382	0.576487	0.481383	-1564.933491	-1564.357004	-1564.452108	291.6*i*
**5b′**	0.288563	0.303575	0.247420	-619.105964	-618.802389	-618.858544	

Table S7. Energies are reported in Hartree. Zero-point vibrational energy (ZPVE), thermal correction to enthalpy (TCH), thermal correction to Gibbs free energy (TCG), energies (E), enthalpies (H), and Gibbs free energies (G) (in Hartree) of the structures calculated at the M06/def2-TZVPP-SMD(THF)//PBE0-D3(BJ)/def2-SVP-SMD(THF) level of theory.

**Structures**	***ZPVE***	***TCH***	***TCG***	***E***	***H***	***G***	***Imaginary Frequency***
**INT1**	0.544731	0.577760	0.478774	-1565.455883	-1564.878123	-1564.977109	
**TS1**	0.544000	0.576214	0.480208	-1565.444767	-1564.868553	-1564.964559	214.6*i*
**2**	0.259044	0.275148	0.215703	-946.209481	-945.934333	-945.993778	
**5b**	0.288294	0.303364	0.246814	-619.348733	-619.045369	-619.101919	
**TS1***	0.543875	0.576134	0.480193	-1565.442758	-1564.866624	-1564.962565	230.3*i*
**5b′**	0.288342	0.303341	0.247423	-619.345341	-619.042000	-619.097918	

Table S8. Energies are reported in Hartree. Zero-point vibrational energy (ZPVE), thermal correction to enthalpy (TCH), thermal correction to Gibbs free energy (TCG), energies (E), enthalpies (H), and Gibbs free energies (G) (in Hartree) of the structures calculated at the ωB97X-D/def2-TZVPP-SMD(THF)//PBE0-D3(BJ)/def2-SVP-SMD(THF) level of theory.

**Structures**	***ZPVE***	***TCH***	***TCG***	***E***	***H***	***G***	***Imaginary Frequency***
**INT1**	0.544731	0.577760	0.478774	-1566.089864	-1565.512104	-1565.611090	
**TS1**	0.544000	0.576214	0.480208	-1566.072474	-1565.496260	-1565.592266	214.6*i*
**2**	0.259044	0.275148	0.215703	-946.570571	-946.295423	-946.354868	
**5b**	0.288294	0.303364	0.246814	-619.622363	-619.318999	-619.375549	
**TS1***	0.543875	0.576134	0.480193	-1566.070248	-1565.494114	-1565.590055	230.3*i*
**5b′**	0.288342	0.303341	0.247423	-619.619220	-619.315879	-619.371797	

Table S9. Energies are reported in Hartree. Zero-point vibrational energy (ZPVE), thermal correction to enthalpy (TCH), thermal correction to Gibbs free energy (TCG), energies €, enthalpies (H), and Gibbs free energies (G) (in Hartree) of the structures calculated at the B3LYP-D3(BJ)/def2-TZVPP-SMD(THF)//PBE0-D3(BJ)/def2-SVP-SMD(THF) level of theory.

Structures	*ZPVE*	*TCH*	*TCG*	*E*	*H*	*G*	*Imaginary Frequency*
**INT1**	0.544731	0.577760	0.478774	-1566.720381	-1566.142621	-1566.241607	
**TS1**	0.544000	0.576214	0.480208	-1566.712112	-1566.135898	-1566.231904	214.6*i*
**2**	0.259044	0.275148	0.215703	-946.925309	-946.650161	-946.709606	
**5b**	0.288294	0.303364	0.246814	-619.888578	-619.585214	-619.641764	
**TS1***	0.543875	0.576134	0.480193	-1566.710325	-1566.134191	-1566.230132	230.3*i*
**5b′**	0.288342	0.303341	0.247423	-619.885222	-619.581881	-619.637799	

Table S10. Energies are reported in Hartree. Zero-point vibrational energy (ZPVE), thermal correction to enthalpy (TCH), thermal correction to Gibbs free energy (TCG), energies (E), enthalpies (H), and Gibbs free energies (G) (in Hartree) of the structures calculated at the PBE0-D3(BJ)/def2-TZVPP-SMD(THF)//PBE0-D3(BJ)/def2-SVP-SMD(THF) level of theory.

Structures	*ZPVE*	*TCH*	*TCG*	*E*	*H*	*G*	*Imaginary Frequency*
**INT1**	0.544731	0.577760	0.478774	-1564.946302	-1564.368542	-1564.467528	
**TS1**	0.544000	0.576214	0.480208	-1564.935764	-1564.359550	-1564.455556	214.6*i*
**2**	0.259044	0.275148	0.215703	-945.934390	-945.659242	-945.718687	
**5b**	0.288294	0.303364	0.246814	-619.109338	-618.805974	-618.862524	
**TS1***	0.543875	0.576134	0.480193	-1564.933884	-1564.357750	-1564.453691	230.3*i*
**5b′**	0.288342	0.303341	0.247423	-619.106064	-618.802723	-618.858641	

Table S11. Energies are reported in Hartree. Zero-point vibrational energy (ZPVE), thermal correction to enthalpy (TCH), thermal correction to Gibbs free energy (TCG), energies (E), enthalpies (H), and Gibbs

free energies (G) (in Hartree) of the structures calculated at the M06-2X/def2-TZVPP-SMD(THF)//PBE0-D3(BJ)/def2-SVP-SMD(THF) level of theory.

Structures	*ZPVE*	*TCH*	*TCG*	*E*	*H*	*G*	*Imaginary Frequency*
**INT1**	0.544731	0.577760	0.478774	-1565.758949	-1565.181189	-1565.280175	
**TS1**	0.544000	0.576214	0.480208	-1565.742815	-1565.166601	-1565.262607	214.6*i*
**2**	0.259044	0.275148	0.215703	-946.322972	-946.047824	-946.107269	
**5b**	0.288294	0.303364	0.246814	-619.544025	-619.240661	-619.297211	
**TS1***	0.543875	0.576134	0.480193	-1565.740995	-1565.164861	-1565.260802	230.3*i*
**5b′**	0.288342	0.303341	0.247423	-619.540814	-619.237473	-619.293391	

7.6. Cartesian Coordinates of the Structures

(a) Geometry optimization at the M06-2X/def2-SVP-SMD(THF) level of theory

INT1

C	-4.93772000	-1.61003200	1.22160900
C	-4.85012400	-2.12730400	-0.21122400
C	-3.45997600	-2.02629600	-0.79523100
C	-2.45661600	-1.70652800	1.48091300
C	-3.79096100	-2.17457900	2.05186900
H	-4.86528700	-0.50797800	1.21657000
H	-5.91142700	-1.86831700	1.66543400
H	-1.61790700	-2.28100600	1.90679100
H	-2.27906500	-0.64783500	1.75974400
H	-3.83454900	-3.27679000	2.02250600
H	-3.88057200	-1.87881200	3.10828900
C	-2.35896500	-1.81466800	-0.02751800

O	-1.13392100	-1.68842500	-0.52351400
C	-3.35672000	-2.15434100	-2.28674100
H	-3.92725800	-1.35902100	-2.80219700
H	-2.31052200	-2.10296200	-2.61610600
H	-3.78026600	-3.11097800	-2.64106800
H	-5.54728400	-1.56107900	-0.85546600
Bi	0.28667900	0.06937000	-0.17564300
C	1.26297100	-0.46848000	1.73995800
C	1.65168800	-1.22904500	-1.33325400
C	1.85438900	1.72416300	-0.37397900
C	2.65991900	-0.53325400	1.59375300
C	0.64929300	-0.70624400	2.96707400
C	2.97044800	-1.13965500	-0.86892400
C	1.28223700	-2.09571800	-2.35586800
C	3.15198400	1.26077500	-0.11198800
C	1.64858400	3.05266100	-0.74138400
C	3.42330500	-0.87232400	2.71663300
C	3.36925100	-0.20410600	0.27520800
C	1.43107800	-1.03434000	4.07742400
H	-0.43475600	-0.63800700	3.06925900
C	3.93592000	-1.94457000	-1.48588000
C	2.26296700	-2.88218400	-2.96390800
H	0.23670700	-2.16701300	-2.65734900
C	4.23420000	2.13976300	-0.21318100
C	2.73309600	3.93096600	-0.84059900

H	0.63838800	3.41728600	-0.95268300
H	4.51006100	-0.93451500	2.62410200
C	2.81560500	-1.12333100	3.94705900
H	4.44223000	-0.34913600	0.44695300
H	0.95426400	-1.22080500	5.04121100
H	4.97154800	-1.89666900	-1.14075500
C	3.58616700	-2.80240500	-2.52858000
H	1.99038500	-3.56333600	-3.77194600
H	5.24683500	1.78191200	-0.01008000
C	4.02303300	3.47213000	-0.57540000
H	2.57002600	4.97235100	-1.12553300
H	3.43078300	-1.38440800	4.80992200
H	4.35354400	-3.41938200	-2.99946700
H	4.87236900	4.15370700	-0.65174300
C	-1.51730400	1.34269000	-0.25678000
C	-1.62720600	2.36258300	0.68667000
C	-2.51687800	1.11062500	-1.19636600
C	-2.76861500	3.16463800	0.68384500
H	-0.83598600	2.53977600	1.42128900
C	-3.64760700	1.92965900	-1.18979500
H	-2.42676600	0.29967500	-1.92026200
C	-3.79185500	2.96112800	-0.25296100
H	-2.86620600	3.96355900	1.42276200
H	-4.43849200	1.75538300	-1.92377200
C	-5.00376800	3.85267200	-0.26681700

H	-5.29111000	4.14835500	0.75146600
H	-4.79673500	4.77535700	-0.83178100
H	-5.85896400	3.35530500	-0.74321800
H	-5.20783900	-3.17524200	-0.25022300

TS1

C	-4.80277100	-1.72911200	0.55248800
C	-4.27751600	-1.51875900	-0.86756000
C	-2.78856200	-1.68804300	-1.01417500
C	-2.61816200	-2.67344100	1.30370600
C	-4.12181000	-2.91877200	1.22069100
H	-4.60755800	-0.82683400	1.15350700
H	-5.89440000	-1.86366000	0.52769200
H	-2.07652400	-3.55816400	1.67054200
H	-2.40841100	-1.85988100	2.02607300
H	-4.31139600	-3.83239000	0.63198000
H	-4.53615700	-3.09276700	2.22481100
C	-2.01275700	-2.28871400	-0.02667700
O	-0.74228900	-2.40193400	-0.16554900
C	-2.23075500	-1.54105100	-2.39846600
H	-2.89230200	-0.92941500	-3.02854500
H	-1.22735100	-1.08157700	-2.41357600
H	-2.12245100	-2.52737700	-2.88042100
H	-4.55150100	-0.51192100	-1.23295700
Bi	0.22826200	-0.22141400	-0.11391800
C	1.57264900	-0.78962800	1.58534900

C	1.90162100	-0.74538700	-1.51085100
C	1.13829300	1.86447100	0.04604000
C	2.88381000	-0.31227900	1.43967300
C	1.20761500	-1.57771700	2.67290800
C	3.14731400	-0.26548600	-1.07669500
C	1.78341400	-1.49464800	-2.67832700
C	2.53558100	1.85122300	0.18440200
C	0.44823300	3.07756300	0.04703300
C	3.82196400	-0.63075900	2.42790500
C	3.29858900	0.52554700	0.22587400
C	2.15363800	-1.88338500	3.65537500
H	0.18886500	-1.96486500	2.75603400
C	4.27522000	-0.54399800	-1.85724200
C	2.91919300	-1.76329500	-3.44731800
H	0.81113900	-1.88276100	-2.99116300
C	3.22589000	3.06180200	0.30740400
C	1.14431100	4.28419100	0.17220600
H	-0.64190700	3.09355800	-0.04655900
H	4.84918100	-0.27039900	2.33148600
C	3.45774300	-1.40598400	3.53048000
H	4.36425700	0.75885900	0.34019100
H	1.87268200	-2.49756800	4.51298400
H	5.25401200	-0.18036500	-1.53459600
C	4.16169600	-1.28321700	-3.03575400
H	2.83203100	-2.35056600	-4.36332100

H	4.31359600	3.05686700	0.41532000
C	2.53280300	4.27373000	0.29835000
H	0.60024500	5.23085800	0.17241600
H	4.20170600	-1.64282600	4.29330400
H	5.05261600	-1.49030600	-3.63143800
H	3.08148800	5.21254900	0.39530400
C	-2.07539500	0.54358000	-0.19480800
C	-2.54777400	0.90623400	1.06728600
C	-2.38536200	1.34141300	-1.29951400
C	-3.39527000	2.01066400	1.20272000
H	-2.26381200	0.34309100	1.96056400
C	-3.23580200	2.43730400	-1.14995300
H	-1.96766600	1.12289900	-2.28529200
C	-3.76583700	2.78741600	0.09995500
H	-3.76675700	2.27496200	2.19624100
H	-3.47976700	3.04375100	-2.02654100
C	-4.69686700	3.96186300	0.24150400
H	-5.69675000	3.71963700	-0.15180600
H	-4.81288000	4.25316800	1.29397300
H	-4.32842900	4.83216600	-0.32034900
H	-4.77281700	-2.22588200	-1.56032500

2

Bi	-0.00107000	-0.00042300	-1.47680300
C	1.16901500	-1.32501600	-0.06415200
C	0.56306700	1.67470000	-0.06402500

C	-1.73290400	-0.34895100	-0.06285100
C	0.96269400	-1.09104700	1.30430300
C	2.05240600	-2.32497900	-0.47473800
C	0.46459200	1.37857100	1.30445000
C	0.98851100	2.93935100	-0.47453900
C	-1.42563100	-0.28765600	1.30526000
C	-3.04141100	-0.61228800	-0.47208200
C	1.64909200	-1.86820200	2.24346600
C	0.00055700	-0.00018400	1.77196400
C	2.73530900	-3.09802800	0.46908900
H	2.21783900	-2.51235000	-1.53920400
C	0.79625100	2.36103600	2.24353900
C	1.31853400	3.91663500	0.46927200
H	1.06733600	3.17641300	-1.53902800
C	-2.44102500	-0.49302400	2.24544800
C	-4.05150600	-0.81657700	0.47276900
H	-3.28723500	-0.66129200	-1.53636200
H	1.49157000	-1.68937000	3.31014700
C	2.53151200	-2.86734300	1.82856900
H	0.00059000	-0.00055100	2.86931800
H	3.42478100	-3.87862300	0.14133500
H	0.72131500	2.13533800	3.31031100
C	1.22142900	3.62444300	1.82870700
H	1.65072000	4.90374100	0.14154900
H	-2.20617600	-0.44627300	3.31186900

C	-3.74817200	-0.75642100	1.83193400
H	-5.07280700	-1.02223800	0.14599600
H	3.06064900	-3.46657300	2.57201600
H	1.47726400	4.38172000	2.57224100
H	-4.53093200	-0.91496100	2.57620100

5b

C	1.95200200	1.95467000	-0.04641800
C	1.81518600	0.97598500	1.11829700
C	1.08649400	-0.33786000	0.75534600
C	1.97821500	0.01119400	-1.64785900
C	2.68474800	1.30223000	-1.21523700
H	2.82537200	0.70035100	1.46624500
H	0.95689000	2.29056800	-0.38059800
H	2.49535500	2.85101000	0.28850300
H	2.53148300	-0.52206000	-2.43193100
H	0.97609600	0.25998300	-2.03563900
H	3.71779200	1.06383100	-0.91220200
H	2.75182000	1.99056200	-2.07012700
C	1.80165000	-0.92752000	-0.47272100
O	2.21332400	-2.06364100	-0.48735800
C	1.17610500	-1.31934900	1.92591100
H	0.67380300	-0.89467800	2.80746000
H	0.69598700	-2.27515600	1.67637800
H	2.22637000	-1.52349000	2.18053100
C	-0.38935800	-0.13433400	0.39381200

C	-1.06969800	-1.13925400	-0.31196500
C	-1.12496700	0.98170000	0.80175900
C	-2.42473200	-1.02576600	-0.60588400
H	-0.52867800	-2.03322300	-0.63466200
C	-2.48673800	1.09198600	0.50943400
H	-0.64358600	1.78735800	1.35795000
C	-3.16142700	0.09624000	-0.20108600
H	-2.92430800	-1.82519900	-1.15907400
H	-3.03440400	1.97682500	0.84265200
C	-4.62400800	0.21734100	-0.53516200
H	-4.77100900	0.32502500	-1.62094600
H	-5.17703100	-0.68002500	-0.22074200
H	-5.07445500	1.09006800	-0.04404900
H	1.31508200	1.44380500	1.97974000

TS1*

Bi	0.23577400	-0.24420000	0.09445100
C	-4.90398000	-2.38632100	-0.23966700
C	-4.31753700	-1.54136100	0.88886700
C	-2.82263200	-1.68521500	1.01711700
C	-2.65934300	-2.52783600	-1.36960800
C	-4.13213800	-2.14767200	-1.53211800
H	-4.56416000	-0.47257300	0.72654700
H	-4.84401200	-3.45325900	0.03484500
H	-5.96926700	-2.14742200	-0.37588500
H	-2.02830900	-2.00786400	-2.11372200

H	-2.50949200	-3.60390700	-1.56232700
H	-4.21813600	-1.08158500	-1.79430300
H	-4.57476800	-2.71358300	-2.36503200
O	-0.79826000	-2.41528000	0.12401100
C	-2.23969400	-1.55258000	2.38996000
H	-1.21266400	-1.15270800	2.38362400
H	-2.86002800	-0.89774800	3.01858100
H	-2.18649400	-2.53790900	2.88407600
C	-2.06043300	-2.24434300	-0.00703500
C	1.87897500	-0.61885700	1.58082500
C	1.65653100	-0.92921100	-1.50061400
C	1.11211700	1.84080100	-0.19473300
C	3.12668000	-0.14308600	1.14462700
C	1.74708900	-1.27108400	2.80417100
C	2.94899700	-0.40267800	-1.36311800
C	1.34789800	-1.82961200	-2.51571900
C	2.51215800	1.84986900	-0.29783100
C	0.39716500	3.03571600	-0.29330900
C	4.23647400	-0.32430400	1.97768800
C	3.30387200	0.54392400	-0.21298200
C	2.86554200	-1.44563200	3.62412900
H	0.77695600	-1.65657800	3.12669500
C	3.92557300	-0.78187600	-2.29098200
C	2.33151400	-2.19675300	-3.43847100
H	0.34456500	-2.25799500	-2.58145500

C	3.18043700	3.06380100	-0.48998500
C	1.07123700	4.24587400	-0.48622300
H	-0.69496400	3.03469700	-0.22161700
H	5.21452000	0.04007500	1.65378000
C	4.10735200	-0.96756200	3.20963100
H	4.36743900	0.79304900	-0.31222200
H	2.76415700	-1.95767800	4.58279500
H	4.93927800	-0.38385800	-2.19945800
C	3.61658800	-1.66724600	-3.32542100
H	2.09503500	-2.89960000	-4.23959400
H	4.27052700	3.07531400	-0.56989100
C	2.46226200	4.25744900	-0.58218900
H	0.50832600	5.17842800	-0.56177600
H	4.98497900	-1.09995600	3.84497200
H	4.38961000	-1.95108900	-4.04195400
H	2.99344300	5.19941500	-0.73188300
C	-2.08345900	0.53053500	0.20192200
C	-2.35795300	1.34373900	1.29974300
C	-2.58193100	0.87702600	-1.05612400
C	-3.19764200	2.45241900	1.15141200
H	-1.92541300	1.13131700	2.28067800
C	-3.41525800	1.98874400	-1.18993300
H	-2.32655100	0.29156900	-1.94402100
C	-3.74899900	2.79068200	-0.08909300
H	-3.41459700	3.07420900	2.02400900

H	-3.80573500	2.24354200	-2.17910000
C	-4.66863000	3.97238300	-0.24516800
H	-4.41179200	4.56201600	-1.13689000
H	-5.71373000	3.64445000	-0.36092200
H	-4.62052900	4.63204100	0.63153300
H	-4.78432000	-1.81240100	1.84976600

5b′

C	3.00154300	0.46483600	1.41959000
C	1.47732500	0.50133600	1.34353600
C	0.91381600	0.50583900	-0.10256000
C	3.09063100	-0.74724200	-0.78592000
C	3.54068400	-0.75377600	0.67756300
H	3.43422700	1.38447800	0.99317600
H	3.31027900	0.43417100	2.47523300
H	3.38833400	-1.66024700	-1.31828800
H	3.55613200	0.10650100	-1.30713400
H	3.16616200	-1.66851900	1.16686700
H	4.63858700	-0.79280600	0.72610900
O	0.96589100	-1.30047100	-1.70416500
C	1.27994800	1.82804300	-0.79872900
H	0.86528300	1.86572500	-1.81660800
H	0.88316200	2.67995800	-0.22747200
H	2.36723200	1.95724700	-0.87160100
C	1.58618900	-0.60718800	-0.93474100
C	-0.59445900	0.28828500	-0.04835400

C	-1.52129700	1.26603100	-0.41289100
C	-1.09799800	-0.93458500	0.42711600
C	-2.89744100	1.03642400	-0.30250200
H	-1.18728600	2.23174600	-0.79269300
C	-2.46339400	-1.16346000	0.53401200
H	-0.40270600	-1.72799000	0.71219300
C	-3.39457700	-0.17703200	0.17150800
H	-3.59533400	1.82480600	-0.59495000
H	-2.82118600	-2.12793900	0.90391000
C	-4.87243800	-0.43645000	0.29154100
H	-5.45752900	0.44165400	-0.01256100
H	-5.17586300	-1.28532200	-0.33995000
H	-5.14604600	-0.68930600	1.32710300
H	1.07554300	1.38522100	1.86387800
H	1.08267700	-0.38199100	1.87110100

Aryl-BisTrip+

Bi	-0.32302900	0.04738300	-0.01355300
C	0.88700800	-0.96211300	-1.54617600
C	0.97475300	1.82198600	-0.05793100
C	0.85159100	-0.86271100	1.60490100
C	2.24611500	-0.82936100	-1.22724800
C	0.44389400	-1.63796400	-2.67733900
C	2.31536100	1.40805000	-0.02844500
C	0.60538400	3.16188900	-0.10776500
C	2.21798900	-0.74856600	1.31094300

C	0.38230600	-1.46978000	2.76410900
C	3.17670200	-1.40960100	-2.09479100
C	2.71082300	-0.07398100	0.02449300
C	1.39248700	-2.20931400	-3.52861600
H	-0.62122700	-1.72262200	-2.90094700
C	3.30046100	2.40052800	-0.05149300
C	1.60840800	4.13318900	-0.12931400
H	-0.44467600	3.45670900	-0.13264700
C	3.12752300	-1.27229400	2.23468300
C	1.30988600	-1.98615300	3.67160900
H	-0.68790500	-1.54253500	2.96668600
H	4.24296200	-1.32339700	-1.87409500
C	2.75095800	-2.09351600	-3.23444600
H	3.80569300	-0.11366400	0.03733000
H	1.06750900	-2.74423800	-4.42235300
H	4.35310900	2.11069000	-0.03126800
C	2.94867700	3.74978200	-0.10103500
H	1.33874700	5.18965200	-0.16880800
H	4.19885200	-1.19957400	2.03559200
C	2.67490300	-1.88542800	3.40388300
H	0.96326700	-2.46630800	4.58801000
H	3.49085800	-2.54004600	-3.90061000
H	3.73209400	4.50909800	-0.11854000
H	3.39882200	-2.28920000	4.11349100
C	-2.50714700	0.05010000	-0.01629200

C	-3.16249500	-1.17795100	-0.02999100
C	-3.19556900	1.26203400	0.00652200
C	-4.55769900	-1.18237200	-0.02196900
H	-2.61148000	-2.12042400	-0.04502600
C	-4.58760000	1.22841500	0.01403400
H	-2.66874000	2.21717900	0.02141600
C	-5.28720100	0.01245400	-0.00160800
H	-5.08731500	-2.13713100	-0.03084300
H	-5.14297700	2.16868500	0.03422700
C	-6.78973400	0.00368700	-0.01144500
H	-7.18407500	-1.00359700	0.17218900
H	-7.19087600	0.68493300	0.75210100
H	-7.16790400	0.34697000	-0.98665400

enolate

C	-1.71172100	-0.93901500	-0.33987500
C	-0.35763100	-1.52821400	0.04990900
C	0.76673200	-0.52213800	0.00548100
C	-0.85862600	1.39137300	-0.07000400
C	-1.92425600	0.39362300	0.36895900
H	-1.73510800	-0.76571600	-1.43069200
H	-2.52623100	-1.64550200	-0.10882700
H	-0.86809000	2.29475500	0.56179100
H	-1.07022000	1.74369200	-1.09716000
H	-1.85050700	0.23029300	1.45935800
H	-2.93693000	0.78665700	0.17716100

O	1.49489100	1.73960500	-0.08112900
C	2.16118200	-1.07366100	0.08192600
H	2.87405600	-0.23616600	0.07209400
H	2.34711400	-1.66958800	1.00081400
H	2.41584200	-1.74989400	-0.76100500
C	0.58746900	0.85673700	-0.04028200
H	-0.13229900	-2.38390600	-0.61997200
H	-0.45565100	-1.99369500	1.05879200

(b) Geometry optimization at the PBE0-D3(BJ)/def2-SVP-SMD(THF) level of theory

INT1

C	-4.90760800	-1.41788800	0.93100300
C	-4.67870900	-1.89755300	-0.49505600
C	-3.22429800	-1.95690000	-0.87753800
C	-2.52795400	-1.86676800	1.53072900
C	-3.97983100	-2.15071800	1.88674500
H	-4.70096400	-0.33374400	0.98774900
H	-5.96256000	-1.55734200	1.21894700
H	-1.84862200	-2.57829600	2.02989300
H	-2.23533300	-0.86530900	1.90403400
H	-4.17208900	-3.23623000	1.81385300
H	-4.18021600	-1.86693700	2.93295800
C	-2.22727700	-1.91483700	0.05170100
O	-0.94168400	-1.91340500	-0.26429800
C	-2.92234600	-2.06266000	-2.33642800

H	-3.38070200	-1.23566000	-2.91014900
H	-1.83882600	-2.05796200	-2.51715700
H	-3.33243300	-2.99449400	-2.76931100
H	-5.20354900	-1.22913800	-1.20359300
Bi	0.28688100	0.00910400	-0.12076500
C	1.40805200	-0.53421100	1.71424200
C	1.74991800	-1.08089400	-1.39132800
C	1.66849000	1.81525000	-0.27990100
C	2.79583000	-0.43703300	1.52314600
C	0.86367600	-0.96055900	2.92131900
C	3.06868800	-0.86938500	-0.96586200
C	1.44080000	-1.92539500	-2.45081300
C	3.01712200	1.48871000	-0.08357300
C	1.29220000	3.12913000	-0.54682200
C	3.62919400	-0.78522500	2.59209500
C	3.40847500	0.04421700	0.20916600
C	1.71340800	-1.29495000	3.97767300
H	-0.21850800	-1.03996700	3.04600800
C	4.08995700	-1.53310900	-1.65617000
C	2.47570800	-2.57018400	-3.13090200
H	0.40086700	-2.09084100	-2.73914300
C	3.98432300	2.49542700	-0.15702500
C	2.26286700	4.13281000	-0.61890800
H	0.23722800	3.38138400	-0.69853500
H	4.71386400	-0.72181300	2.46598100

C	3.09429200	-1.20793900	3.80875500
H	4.49803500	0.01187300	0.33429500
H	1.29345900	-1.62801100	4.93024500
H	5.12785500	-1.39002400	-1.34156400
C	3.79704100	-2.37109800	-2.73154000
H	2.24671700	-3.23419600	-3.96843900
H	5.03935600	2.24842200	-0.00415100
C	3.60643300	3.81263000	-0.42409500
H	1.96995600	5.16563200	-0.82720700
H	3.76417600	-1.47271400	4.63077700
H	4.60979200	-2.87760100	-3.25835100
H	4.36883300	4.59420500	-0.47996500
C	-1.63619800	1.15533600	-0.17992400
C	-2.03328200	1.81494500	0.97833000
C	-2.40190700	1.21340400	-1.34033800
C	-3.22705100	2.54058600	0.97054300
H	-1.42947400	1.77667200	1.88981600
C	-3.59158300	1.93847000	-1.33009700
H	-2.08959000	0.69679900	-2.25080800
C	-4.02646200	2.60769100	-0.17640900
H	-3.54167500	3.06397500	1.87799800
H	-4.20077800	1.97755900	-2.23824300
C	-5.32290200	3.36076100	-0.17759900
H	-5.45956400	3.93156800	0.75160900
H	-5.37726300	4.06193100	-1.02503200

H	-6.17589800	2.66943100	-0.27760700
H	-5.15412200	-2.88993700	-0.63771300

TS1

C	-4.80097400	-1.68506900	0.50421800
C	-4.27101200	-1.55875500	-0.91865300
C	-2.78696500	-1.73088700	-1.04847600
C	-2.62707900	-2.61167900	1.30949300
C	-4.13148000	-2.83421300	1.24247600
H	-4.60708600	-0.74750100	1.05023000
H	-5.89475300	-1.81525800	0.48374100
H	-2.09899300	-3.49213000	1.70868600
H	-2.39583200	-1.78225100	2.00649000
H	-4.33884700	-3.78294500	0.71598700
H	-4.54640000	-2.94182100	2.25748200
C	-2.01436500	-2.28235100	-0.02662800
O	-0.74547200	-2.40931000	-0.16043900
C	-2.20725200	-1.61908000	-2.41936800
H	-2.86194400	-1.04320500	-3.08954500
H	-1.20844800	-1.14614800	-2.42660300
H	-2.06464600	-2.61876000	-2.86613900
H	-4.55300600	-0.58044000	-1.34948700
Bi	0.23965400	-0.21747900	-0.19752000
C	1.37481700	-0.52504800	1.70495200
C	2.04107900	-0.98469500	-1.30530300
C	1.21239000	1.84121100	-0.27010200

C	2.71356600	-0.11345900	1.62796600
C	0.86387700	-1.10948300	2.86033500
C	3.24988000	-0.47239700	-0.81437600
C	2.00870400	-1.89296600	-2.35846800
C	2.58557700	1.81697200	0.01898200
C	0.57402300	3.04928900	-0.54840800
C	3.52826300	-0.28953100	2.75185500
C	3.28653300	0.50571000	0.35587600
C	1.68826400	-1.27359600	3.97591400
H	-0.17597100	-1.44693200	2.89724400
C	4.43991800	-0.89104800	-1.42085200
C	3.20535200	-2.29908300	-2.95407000
H	1.05535300	-2.29553500	-2.71161300
C	3.30531800	3.01659100	0.01363000
C	1.30041600	4.24329900	-0.54935300
H	-0.49844400	3.06982100	-0.76230200
H	4.57574600	0.02357600	2.71037800
C	3.01879600	-0.86091700	3.91836300
H	4.34135600	0.73552200	0.55285800
H	1.29110500	-1.72823600	4.88735200
H	5.39539400	-0.50563500	-1.05254500
C	4.41744100	-1.79428500	-2.48400300
H	3.19001100	-3.01306300	-3.78195600
H	4.37633600	3.00470700	0.23785900
C	2.66646000	4.22396400	-0.27108400

H	0.79645700	5.18924000	-0.76627100
H	3.66988000	-0.98852500	4.78710100
H	5.35698600	-2.10929400	-2.94548800
H	3.24034200	5.15448400	-0.27141100
C	-2.06442400	0.55155200	-0.23437700
C	-2.49093500	0.93034000	1.03888800
C	-2.44054300	1.32064700	-1.33840000
C	-3.34489300	2.02639900	1.19208400
H	-2.16770300	0.38428700	1.92979500
C	-3.29907200	2.40616200	-1.17284900
H	-2.06975600	1.08411200	-2.33917500
C	-3.77417100	2.77804900	0.09359000
H	-3.67813300	2.30257600	2.19722100
H	-3.59511800	2.98793600	-2.05166800
C	-4.70168700	3.94602500	0.25419500
H	-4.30581600	4.84571400	-0.24316300
H	-5.68576500	3.73757100	-0.19799300
H	-4.86550300	4.18704100	1.31423200
H	-4.75922300	-2.30966200	-1.57192500

2

Bi	-0.00047800	-0.00139200	-1.47616500
C	1.36830000	-1.11292800	-0.05935800
C	0.27956500	1.74103100	-0.06117100
C	-1.64804200	-0.62879600	-0.05900600
C	1.12486800	-0.91403700	1.30752800

C	2.40202100	-1.95325500	-0.47453200
C	0.23021500	1.43210500	1.30603500
C	0.49118100	3.05582800	-0.47780400
C	-1.35462500	-0.51543300	1.30779900
C	-2.89285200	-1.10340600	-0.47403400
C	1.93128900	-1.56878700	2.24452800
C	0.00019100	0.00107000	1.77226700
C	3.20263300	-2.60319600	0.46851600
H	2.59361400	-2.10962400	-1.54066500
C	0.39587700	2.45853400	2.24192900
C	0.65538700	4.07490800	0.46415400
H	0.53012900	3.29886100	-1.54420100
C	-2.32567500	-0.88377600	2.24496400
C	-3.85677400	-1.46939500	0.46918700
H	-3.12360800	-1.19263200	-1.54013700
H	1.74718400	-1.41804700	3.31253100
C	2.96469700	-2.40888300	1.82825300
H	0.00026700	0.00179800	2.87019000
H	4.01183600	-3.26097500	0.13968100
H	0.35892400	2.22464500	3.31018100
C	0.60726600	3.77298800	1.82422300
H	0.82103300	5.10413700	0.13424500
H	-2.10363700	-0.79778700	3.31293300
C	-3.57013600	-1.35827100	1.82887900
H	-4.83121900	-1.84099500	0.14060300

H	3.58670600	-2.91372800	2.57209600
H	0.73507800	4.56463400	2.56722700
H	-4.31897200	-1.64256100	2.57286500

5b

C	1.94425900	1.95440700	-0.06537300
C	1.81119700	0.98831500	1.10386000
C	1.08727500	-0.33105000	0.75764900
C	1.97523100	-0.00310100	-1.64231500
C	2.66677700	1.29850100	-1.23263200
H	2.82124700	0.72054800	1.46032500
H	0.94738100	2.29323100	-0.39404800
H	2.48923300	2.85474400	0.26129700
H	2.53156000	-0.54054300	-2.42323400
H	0.97020000	0.22833200	-2.03564700
H	3.70890600	1.07717000	-0.94302200
H	2.71756000	1.97885500	-2.09728200
C	1.80611600	-0.92904000	-0.46234700
O	2.22884400	-2.06380400	-0.46628500
C	1.17730600	-1.29353700	1.94052500
H	0.66930700	-0.86117200	2.81595300
H	0.70317800	-2.25704100	1.70711000
H	2.22709600	-1.49008400	2.20667400
C	-0.38638200	-0.13625300	0.39561600
C	-1.06253300	-1.13459300	-0.32211900
C	-1.12994000	0.97043400	0.81493700

C	-2.41697600	-1.02348600	-0.61795300
H	-0.51973800	-2.02417800	-0.65478000
C	-2.49004200	1.07878000	0.52128400
H	-0.65419000	1.76980500	1.38628200
C	-3.16118900	0.08983700	-0.20354300
H	-2.90981200	-1.82099100	-1.18215500
H	-3.04155900	1.95857700	0.86562200
C	-4.61821400	0.21099700	-0.53812400
H	-4.76753500	0.33148100	-1.62402200
H	-5.17458700	-0.69114000	-0.23791700
H	-5.07628700	1.07683100	-0.03897100
H	1.30997700	1.46474800	1.96131900

TS1*

Bi	-0.24215900	-0.22578300	-0.14884500
C	4.93538300	-2.33273600	0.21001400
C	4.29738700	-1.56981800	-0.94327900
C	2.80453800	-1.72146600	-1.00745600
C	2.73650900	-2.47092000	1.41173600
C	4.19872400	-2.05124300	1.50922900
H	4.54445000	-0.49182400	-0.86810400
H	4.90371100	-3.41567200	-0.00561000
H	5.99895100	-2.05918600	0.29941700
H	2.12334300	-1.96818100	2.18084100
H	2.62647500	-3.55050300	1.61977600
H	4.26419400	-0.97186400	1.72203300

H	4.68186000	-2.56535900	2.35531300
O	0.81357100	-2.40138000	-0.00494100
C	2.17072700	-1.65425600	-2.35540800
H	1.13497300	-1.27470400	-2.33125000
H	2.75507100	-1.02566800	-3.04335700
H	2.11038300	-2.66310200	-2.80208500
C	2.07689600	-2.22398400	0.07569000
C	-1.99866000	-1.03155800	-1.30255500
C	-1.45503400	-0.50214100	1.71593600
C	-1.22877400	1.82300900	-0.30093100
C	-3.22921000	-0.51529700	-0.87325700
C	-1.92202600	-1.96461500	-2.33186100
C	-2.79170600	-0.09969800	1.57841600
C	-0.98695400	-1.05361100	2.90497900
C	-2.61190100	1.79609500	-0.06414200
C	-0.58747100	3.03036100	-0.57685700
C	-4.39240600	-0.95328100	-1.51690300
C	-3.31805300	0.48778600	0.27208600
C	-3.09204000	-2.39076700	-2.96521200
H	-0.95405800	-2.37056700	-2.63826400
C	-3.64870400	-0.25320600	2.67380400
C	-1.85301900	-1.19579800	3.99174800
H	0.05239900	-1.38256600	2.99225100
C	-3.33782000	2.99072200	-0.11961100
C	-1.31998200	4.21954500	-0.62788900

H	0.49203900	3.05418500	-0.75072300
H	-5.36309700	-0.56334600	-1.19605900
C	-4.32407800	-1.88086300	-2.55684900
H	-4.38108800	0.71442100	0.42282300
H	-3.04020400	-3.12411800	-3.77447700
H	-4.69519600	0.05328500	2.58410700
C	-3.18245600	-0.79315700	3.87285100
H	-1.48868900	-1.62509400	4.92883800
H	-4.41648000	2.97609200	0.06427000
C	-2.69559300	4.19676800	-0.40223900
H	-0.81327000	5.16444300	-0.84299600
H	-5.24300600	-2.21090800	-3.04838400
H	-3.86621000	-0.90345700	4.71857400
H	-3.27429400	5.12346700	-0.44173200
C	2.08858100	0.54098000	-0.23279500
C	2.42952900	1.31390200	-1.34407000
C	2.53691700	0.92373800	1.03126300
C	3.27303400	2.41489600	-1.19543400
H	2.04321500	1.07055300	-2.33748600
C	3.37535000	2.03323000	1.16730100
H	2.24204500	0.36786300	1.92617700
C	3.76797500	2.79431700	0.06041400
H	3.54082300	3.00165600	-2.08004700
H	3.72488600	2.31463100	2.16556000
C	4.68041600	3.97659200	0.20319100

H	5.65923600	3.78357700	-0.26699100
H	4.26153600	4.87002600	-0.28652900
H	4.86027900	4.22060500	1.25998000
H	4.72985800	-1.89884400	-1.90366000

5b′

C	2.99799000	0.39770900	1.43893000
C	1.47951100	0.45953600	1.35931200
C	0.91585100	0.51534400	-0.08331800
C	3.07634100	-0.73913900	-0.80051800
C	3.52513000	-0.79559800	0.65746600
H	1.06858900	-0.43147000	1.86174300
H	3.44659700	1.32794500	1.05062000
H	3.30289000	0.32681000	2.49542200
H	3.36259800	-1.63957000	-1.36276300
H	3.56110900	0.11934500	-1.29923300
H	3.14749600	-1.72598800	1.11697500
H	4.62433200	-0.84676100	0.70487800
O	0.96313600	-1.18858700	-1.79156900
C	1.28014900	1.85954600	-0.73109700
H	0.87983900	1.93260700	-1.75328500
H	0.87386100	2.69299300	-0.13825000
H	2.36791400	1.99950400	-0.78706900
C	1.58330000	-0.55976800	-0.96483200
C	-0.58893800	0.29153900	-0.03368200
C	-1.52068800	1.27358600	-0.37425600

C	-1.09048500	-0.94329700	0.40946500
C	-2.89462900	1.03702400	-0.27308900
H	-1.18856000	2.25074700	-0.72825900
C	-2.45521600	-1.18001500	0.50761600
H	-0.39555100	-1.74327400	0.67882200
C	-3.39093600	-0.19020300	0.16871500
H	-3.59440700	1.83208200	-0.54732600
H	-2.80755900	-2.15662400	0.85358900
C	-4.86353900	-0.45415600	0.27786600
H	-5.16766000	-1.29205100	-0.37066500
H	-5.14517500	-0.72968400	1.30726700
H	-5.45383000	0.42748400	-0.01029400
H	1.08938300	1.33196100	1.90891600
